# Copper-catalyzed asymmetric C(sp^2^)–H arylation for the synthesis of P- and axially chiral phosphorus compounds

**DOI:** 10.1038/s41467-023-37987-8

**Published:** 2023-04-20

**Authors:** Shao-Bai Yan, Rui Wang, Zha-Gen Li, An-Na Li, Chuanyong Wang, Wei-Liang Duan

**Affiliations:** 1 College of Chemistry and Chemical Engineering, Yangzhou University, 180 Siwangting Road, 225002 Yangzhou, China; 2 College of Chemistry and Chemical Engineering, Inner Mongolia University, 235 West University Street, 010021 Hohhot, China; 3 School of Chemistry and Chemical Engineering, Shaanxi Normal University, 620 Xi Changan Street, 710119 Xi’an, China

**Keywords:** Asymmetric synthesis, Synthetic chemistry methodology

## Abstract

Transition metal-catalyzed C–H bond functionalization is an important method in organic synthesis, but the development of methods that are lower cost and have a less environmental impact is desirable. Here, a Cu-catalyzed asymmetric C(sp^2^)–H arylation is reported. With diaryliodonium salts as arylating reagents, a range of *ortho*-arylated P*-*chiral phosphonic diamides were obtained in moderate to excellent yields with high enantioselectivities (up to 92% ee). Meanwhile, enantioselective C-3 arylation of diarylphosphine oxide indoles was also realized under similar conditions to construct axial chirality.

## Abstract (web summary)

Although transition metal-catalyzed C–H bond functionalization is a widely used method in organic synthesis, many methods rely on metals of low abundance. Here, the authors report a copper-catalyzed, asymmetric C–H arylation using diaryliodonium salts.

## Introduction

Transition metal-catalyzed C–H bond functionalization has become an important methodology for organic synthesis in terms of atom economy and step economy^1–4^. Over the past several decades, elegant and efficient palladium-, rhodium-, iridium-, and ruthenium-catalyzed transformations for the stereoselective construction of C–C and/or C–X bonds through C–H activation have been well exploited^5–8^. In these well-established methodologies, two general strategies have been followed to achieve enantiocontrol: (1) enantio-determining activation of enantiotopic C–H bonds by chiral transition metal complexes; (2) unbiased C–H bond scission followed by stereochemistry-generating incorporation of coupling partners. To realize those transformations, many chiral ligands and catalysts have been developed to achieve excellent selectivity^9–13^. On the other hand, a cost-effective and environmentally benign method to selectively activate inert C–H bonds is still desirable in this field.

**Fig. 1:**
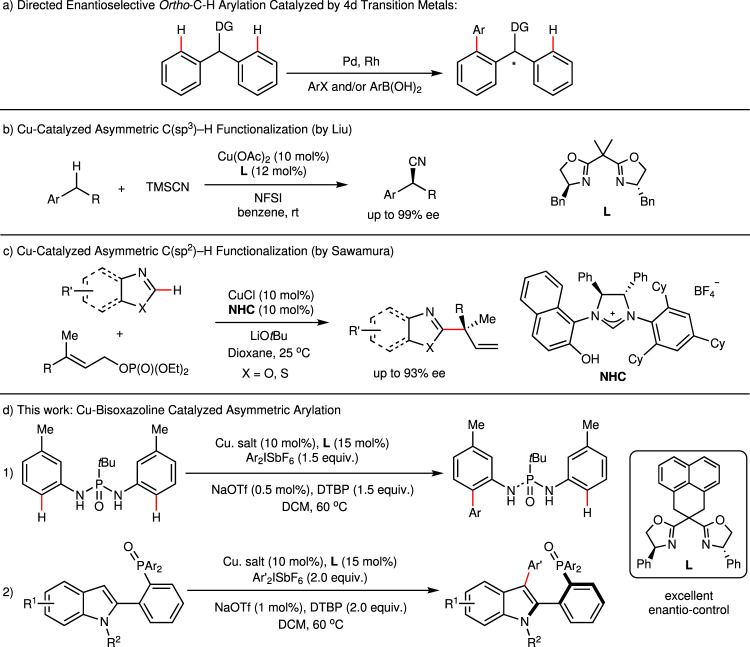
Metal-catalyzed asymmetric C–H functionalization. **a** Directed enantioselective o*rtho*-C–H arylation catalyzed by 4d transition metals. **b** Cu-catalyzed asymmetric C(sp^3^)–H functionalization. **c** Cu-catalyzed asymmetric C(sp^2^)–H functionalization. **d** This work: Cu-bisoxazoline-catalyzed asymmetric arylation. NFSI N-fluorobenzenesulfonimide, DTBP 2,6-di-*tert*-butylpyridine.

First-row metals are more earth-abundant and less toxic than precious 4d and 5d metals. Recently, the utilization of these 3d metals to activate omnipresent C–H bonds, especially enantioselectively, has gained increasing interest from chemists^14–28^. Hou and coworkers developed chiral cyclopentadienyl scandium complexes to catalyze enantioselective transformations, including C–H addition of pyridines to olefins^16^, C–H alkylation of imidazoles with 1,1-disubstituted alkenes^17^ and C–H alkenylation of ferrocenes with alkynes^18^. In 2017, Butenschön revealed an iron-catalyzed arylation of ferrocenamide with moderate enantioselectivities using (*R*,*R*)-chiraphos as the ligand^19^. Cramer and coworkers reported nickel-catalyzed asymmetric cyclization of pyridone derivatives with N-heterocyclic carbenes (NHCs) as the chiral source^20^. Despite significant achievements in the literature, inexpensive 3d metal-catalyzed asymmetric C–H activation is still in its infancy. Furthermore, alkenes and alkynes are the most commonly used coupling partners in reports to date, and examples of C–H arylation are quite scarce. Palladium, rhodium, and iridium are commonly used transition metals in directed enantioselective C–H arylation reactions (Fig. 1a).

Copper, which constitutes approximately 0.01% of the Earth’s crust, has three main oxidation states ranging from 0 to +3, making it more properly a 3d metal in transformations involving radicals or two-electron transfer^29,30^. Recently, Liu and others developed a Cu-catalyzed asymmetric C(sp^3^)–H functionalization involving radicals with impressive enantioselectivities (Fig. 1b)^31,32^. Cu-catalyzed C–H arylation of heteroarenes with aryl iodides was first reported by Daugulis, Miura, and Ackermann^33–36^. Since then, Cu-catalyzed C–H functionalization has made considerable progress in the construction of C–C and C–X bonds^37–43^. For example, Yu reported a Cu-catalyzed *ortho*-arylation of carboxamides directed by oxazoline-based bidentate groups in 2014^44^. Although research on Cu-catalyzed C–H functionalization, including arylation, alkylation, nitrogenation, halogenation, and so on, has surged in recent years, asymmetric transformations remain poorly developed. In 2016, Sawamura’s group published their work on Cu-catalyzed asymmetric allyl phosphate substitution by azoles to construct all-carbon quaternary stereocenters (Fig. 1c)^45^. A designed chiral NHC ligand was used to generate excellent branch regioselectivity and enantiocontrol, but C–H bond cleavage was not the enantio-determining step. Dai and Yu attempted Cu-mediated diastereoselective C–H thiolation of ferrocenes utilizing a chiral oxazoline bidentate directing group in 2018^46^.

In 2008, Gaunt found that diaryliodonium salts could serve as arylating reagents in the copper-catalyzed C–H arylation of indoles, and an acetyl moiety on the N atom switched the regioselectivity from C3 to C2^47^. The author proposed that a highly electrophilic aryl–Cu(III) intermediate was essential to the reactivity. Several groups extended this methodology, and diaryliodonium salts were proven to be powerful arylating reagents for the C–H bond functionalization of arenes^48–55^. While chiral bisoxazolines (BOXs) are common ligands in copper-catalyzed transformations^56–58^, we questioned whether asymmetric Cu-catalyzed C–H bond arylation could be achieved by the combination of chiral bisoxazolines and diaryliodonium salts.

In this work, we develop a Cu/bisoxazoline-catalyzed asymmetric C(sp^2^)–H arylation with diaryliodonium salts, and high stereoselectivities were obtained for *P*-chiral phosphonic diamides and axially chiral phosphorus-containing indoles (Fig. 1d).

## Results and discussion

### Optimization of reaction conditions

We commenced our investigations with symmetric phosphonic diamide **a1** and [PhIMes]SbF_6_ with CuI as the catalyst (Table 1; see the SuppIementary Information for more details). Initially, various bisoxazoline ligands were screened, and commercially available chiral BOX **L1** generated desired *ortho*-arylated product **b1** in 50% yield with weak enantiocontrol (10% ee, Entry 1). Next, phenyl-substituted spiro-BOXs **L2–L5** were examined (Entries 2–5). Cyclopropane- and cyclobutane-backboned ligands **L2** and **L3** yielded similar results, with moderate ee values, while cyclopentane-backboned spiro-BOX **L4** showed low reactivity. The enantioselectivity increased to 75% when the ring size was increased to a cyclohexane structure (Entry 5). A dramatic increase in enantioselectivity from 75 to 90% was observed when 2,3-dihydro-1*H*-phenalene-backboned BOX **L6** was used, which may be attributed to the additional rigidity of the backbone compared to that of **L5** (Entry 6). Unfortunately, modifications of the phenyl substituent of **L6** did not result in positive effects on selectivity (Entries 7–9). In addition, ligand **L10** with a seven-membered ring backbone was also tested and exhibited inferior enantioselectivity (69% ee, Entry 10). Under the same conditions, CuCl resulted in slightly better enantioselectivity than CuI (91%, Entry 11). 2,6-Di-*tert*-butylpyridine (DTBP) is a general acid side-product scavenger in Gaunt’s works with diaryliodonium salts;^47,51^ hence, it was also applied in this reaction to improve the yield, but to our surprise, the reaction was severely suppressed (Entry 12). We hypothesized that DTBP might abstract an anion, which facilitates H atom dissociation during C–H bond activation, from the system, so NaOTf as an external anion source was added (Entries 13–15). After investigating the molar ratio of NaOTf, 0.5 mol% was determined to be the best value, affording the product in 97% yield and 91% ee (Entry 14). Thus, we established an optimal catalyst system: CuCl (10 mol%)/**L6** (15 mol%) with 1.5 equiv. of DTBP and 0.5 mol% NaOTf in dichloromethane.

**Table 1:** Optimization of the enantioselective o*rtho*-C(sp^2^)–H arylation of a1^a^

Entry	Cu. salt	Ligand	Base	Yield (%)^b^	ee (%)^c^
1	CuI	**L1**	/	50	-10
2	CuI	**L2**	/	16	56
3	CuI	**L3**	/	15	52
4	CuI	**L4**	/	Trace	-
5	CuI	**L5**	/	45	75
6	CuI	**L6**	/	30	90
7	CuI	**L7**	/	25	80
8	CuI	**L8**	/	49	79
9	CuI	**L9**	/	Trace	-
10	CuI	**L10**	/	10	69
11	CuCl	**L6**	/	40	91
12	CuCl	**L6**	DTBP	Trace	-
13^d^	CuCl	**L6**	DTBP	87	85
14^e^	CuCl	**L6**	DTBP	97	91
15^f^	CuCl	**L6**	DTBP	64	84

*DTBP* 2,6-di-*tert*-butylpyridine.

^a^Reaction conditions unless otherwise noted: **1a** (0.10 mmol), [PhIMes]SbF_6_ (0.15 mmol), copper salt (0.01 mmol), ligand (0.015 mmol), Base (0.15 mmol), DCM (1 mL) in a sealed tube at 60 °C for 24 h.

^b^Isolated yields.

^c^Determined by chiral HPLC.

^d^With 1 mol% NaOTf.

^e^With 0.5 mol% NaOTf.

^f^With 0.1 mol% NaOTf.

### Substrate scope

**Fig. 2:**
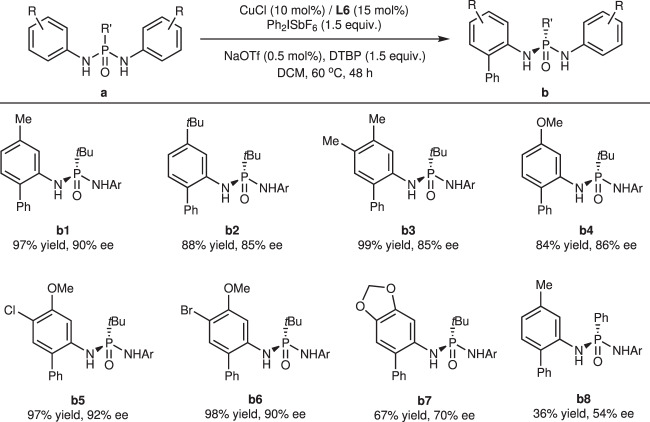
Substrate scope with phosphonic diamides. Reaction conditions unless otherwise noted: **a** (0.10 mmol), Ph_2_ISbF_6_ (0.15 mmol), CuCl (0.01 mmol), **L6** (0.015 mmol), NaOTf (0.0005 mmol), DTBP (0.15 mmol), DCM (2 mL) in a sealed tube at 60 °C for 48 h. DTBP 2,6-di-*tert*-butylpyridine.

With the optimal conditions in hand, we examined the substrate scope of this enantioselective *ortho*-C(sp^2^)-H arylation reaction (Fig. 2). Initially, unsubstituted *P*-*tert*-butyl-N,N’-diphenyl phosphonic diamine was subjected to the standard conditions, but only trace product was generated, and a bromo moiety *ortho* to the methyl group in **a1** completely inhibited the reactivity (see the SuppIementary Information for details). These findings suggest that the reaction might be controlled by electronics, necessitating electron-donating groups (EDGs) on the substrate. Several phosphonic diamides with different EDGs were tested. *Tert*-butyl and dimethyl groups exhibited similar inferior enantioselectivities (85%, **b2** and **b3**). A methoxy group at the *meta*-position reduced the ee value to 86% because of electronic effects (**b4**), while halogenation could alleviate the impact and lead to improved results (**b5**, **b6**). Substrate **a7**, with a strong electron-donating 3,4-methylenedioxy group, yielded much poorer results, which might be attributed to a faster background reaction. Replacing the butyl moiety on the P atom with a phenyl moiety resulted in only moderate enantioselectivity, which could be explained by the similar steric hindrance between phenyl and arylamine.

**Fig. 3:**
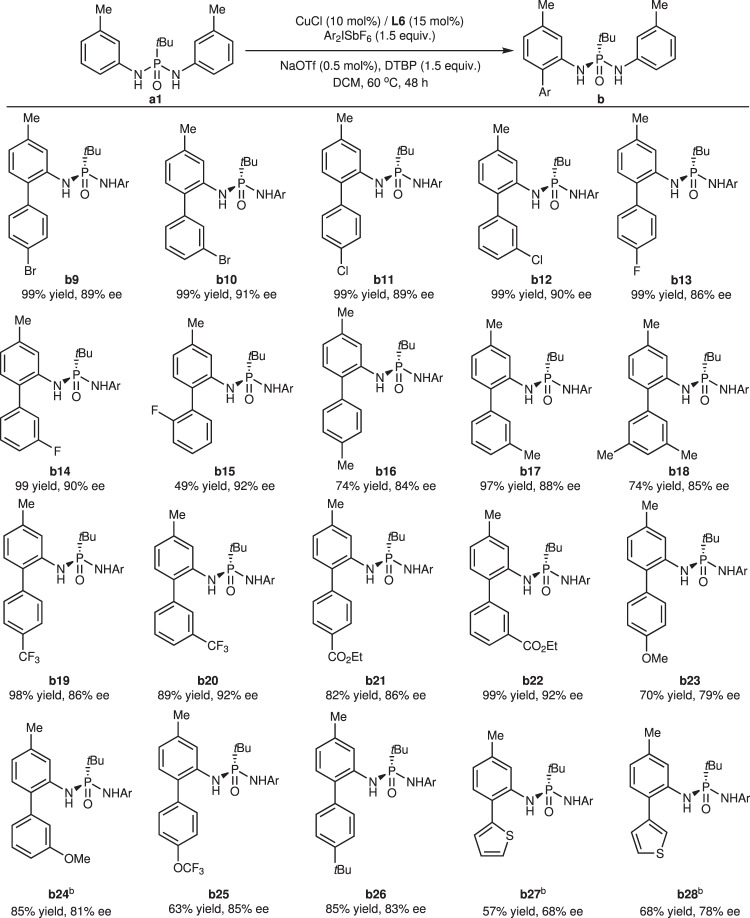
Substrate scope with diaryliodonium salts. Reaction conditions unless otherwise noted: **a1** (0.10 mmol), Ar_2_ISbF_6_ (0.15 mmol), CuCl (0.01 mmol), **L6** (0.015 mmol), NaOTf (0.0005 mmol), DTBP (0.15 mmol), DCM (2 mL) in a sealed tube at 60 °C for 48 h. ^b^[ArIMes]SbF_6_ was used. DTBP 2,6-di-*tert*-butylpyridine.

Next, a series of diaryliodonium salts were synthesized and tested in this reaction (Fig. 3). Halogenation at the *para* and *meta* positions of diaryliodonium salts has a negligible influence on the results, except *para*-fluorination resulted in slightly lower enantioselectivity due to the electron-withdrawing effect (**b9**-**b14**). *Ortho*-fluoro diaryliodonium salt produced the corresponding *ortho*-arylated product in moderate yield because of steric repulsion but did not affect the selectivity (**b15**). Other functional groups, such as methyl, trifluoromethyl, ethoxycarbonyl, methoxy, trifluoromethoxy, and *tert*-butyl groups, exert notable electronic effects on enantioselectivity. Strong electron-donating and electron-withdrawing abilities were both detrimental to stereo-induction, but substituents in the *para-*position had a more remarkable negative impact than those in the *meta*-position (**b16**-**b26**). The absolute configuration of product **b** was assigned to be *R* according to the X-ray crystal diffraction analysis (XRD) of product **b18**^59^. Finally, heterocyclic diaryliodonium salts were also used in this reaction, and thiophenyl groups were successfully introduced at the *ortho-*position of **a1** with moderate yields and enantioselectivities (**b27-b28**).

**Fig. 4:**
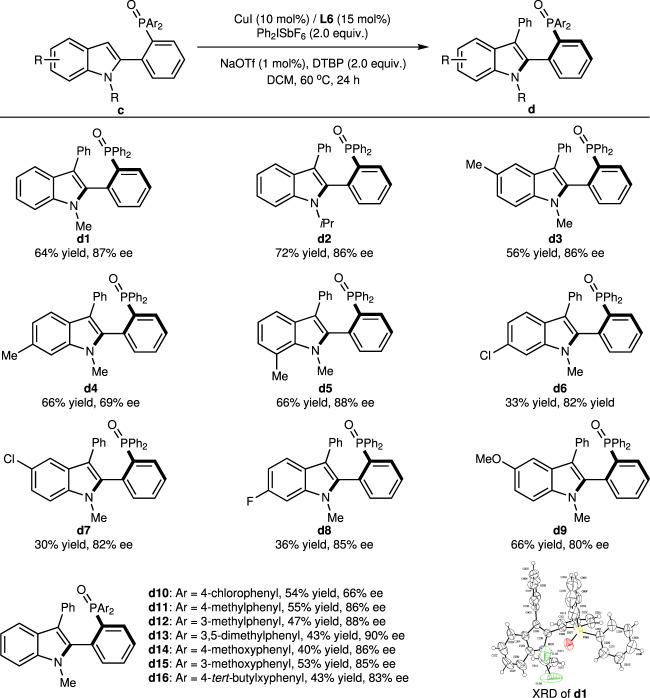
Substrate scope with diphenylphosphine oxide indoles. Reaction conditions unless otherwise noted: **c** (0.10 mmol), Ph_2_ISbF_6_ (0.2 mmol), CuI (0.01 mmol), **L6** (0.015 mmol), NaOTf (0.001 mmol), DTBP (0.2 mmol), DCM (2 mL) in a sealed tube at 60 °C for 24 h. DTBP 2,6-di-*tert*-butylpyridine, XRD X-ray crystal diffraction analysis.

Axially chiral biaryl monophosphines are an important class of ligands in asymmetric catalysis, and the development of the new synthetic protocol for these compounds has attracted much attention^60^. As phosphine oxides are common precursors of trivalent phosphines, we envisioned the developed phosphine oxide-directed C–H arylation reaction could be extended to the construction of axially chiral biaryl monophosphine oxides^61^. We chose diphenylphosphine oxide indole **c1** as the standard substrate under similar reaction conditions (Fig. 4, see SuppIementary Information for detailed condition optimization), and the C-3 phenylated product was obtained in 64% yield with 87% ee. XRD results of **d1** indicate the configuration to be *R* around the axis^62^.

The scope of diphenylphosphine oxide indoles was also investigated, and the isopropyl on the N atom of the indole ring gave similar results to methyl (**d2**), while results varied with sites of methyl on the indole ring. 5- and 7-methylated substrates produced approximate results (**d3** and **d5**), but methyl on the 6-position generates much poorer stereoselectivity (69% ee, **d4**). Such results could be explained as follows: EDGs *para* to C-3 increased the electron cloud density at the reaction site and accelerated the background reaction. Substrate **c8** with stronger EDG methoxy on the 5-position also validated this deduction (**d9**). Conversely, electron-withdrawing groups (EWGs) like chloro and fluoro on the indole ring severely hampered the reaction rate with slight erosion on ee values of products (**d6**-**d8**). Next, different functional groups on diphenylphosphine oxide were also screened. Generally, EDGs on phenyl rings benefit the stereocontrol, giving product in 83–90% ee with moderate yields (**d11**-**d16**). Unexpectedly, chloro on *para*-position of phenyl rings has distinct corruption on the stereoselectivity, generating the product in 66% ee (**d10**). We think this could be ascribed to the weaker directing ability of the electron-deficient diphenylphosphine oxide group.

**Fig. 5:**
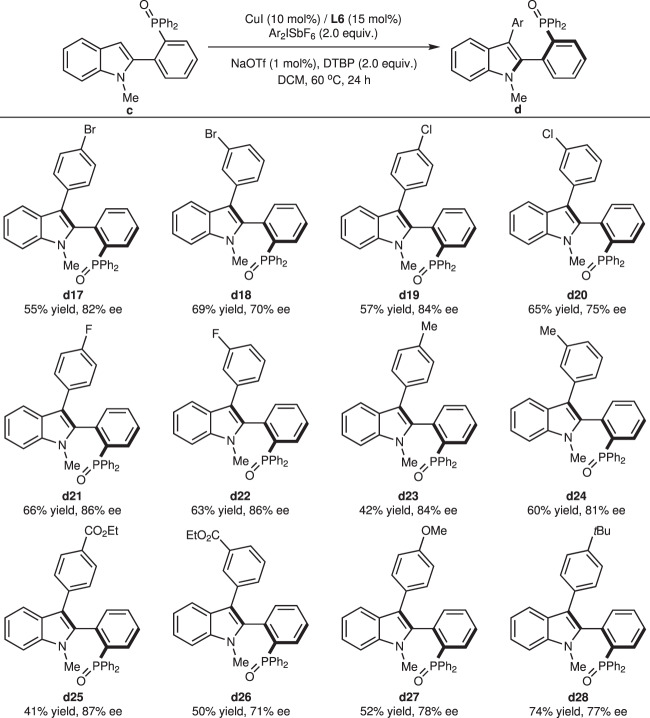
Substrate scope with diaryliodonium salts for diphenylphosphine oxide indoles. Reaction conditions unless otherwise noted: **c** (0.10 mmol), Ar_2_ISbF_6_ (0.2 mmol), CuI (0.01 mmol), **L6** (0.015 mmol), NaOTf (0.001 mmol), DTBP (0.2 mmol), DCM (2 mL) in a sealed tube at 60 °C for 24 h. DTBP 2,6-di-*tert*-butylpyridine.

Furthermore, the scope of diaryliodonium salts for asymmetric C-3 arylation of diphenylphosphine oxide indole **c1** was examined under the same conditions (Fig. 5). Groups with different electronic effects, like bromo, chloro, fluoro, methyl, ethoxycarbonyl, methoxy, and *tert*-butyl, were well tolerated and gave corresponding arylated products in 70–87% ee with moderate to good yields. As a whole, *meta*-position of substitutes on the phenyl ring of diaryliodonium salts renders low enantioselectivity than *para*-position, which is contrary to the tendency observed with phosphonic diamide **a1** in Fig. 3. We speculated that the steric effect plays a more important role than the electronic effect during the construction of axial chirality, opposite to *P*-central chirality.

### Synthetic applications

**Fig. 6:**
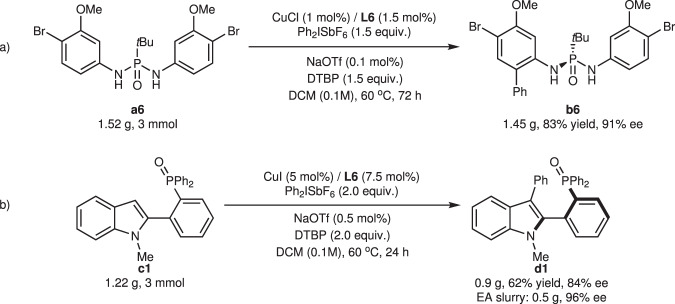
Scale-up experiment. **a** Scale up for **b6**. **b** Scale up for **d1**. DTBP 2,6-di-*tert*-butylpyridine, EA ethyl acetate.

To further demonstrate the robustness of the developed method, the two arylation reactions were performed at the gram scale under adjusted conditions. With catalyst loading reduced to 1 mol%, *ortho*-arylated product **b6** was isolated in 83% yield with 91% ee after 72 h of heating (Fig. 6a). C-3 arylation product of **c1** was obtained in 62% yield and 84% ee with 5 mol% catalyst loading (Fig. 6b). Meanwhile, slurry **d1** in ethyl acetate (EA) could afford the enantio-enriched product in 96% ee, which offers further possibilities for application of this axially chiral biaryl monophosphine oxides in catalysis.

### Mechanistic studies

**Fig. 7:**
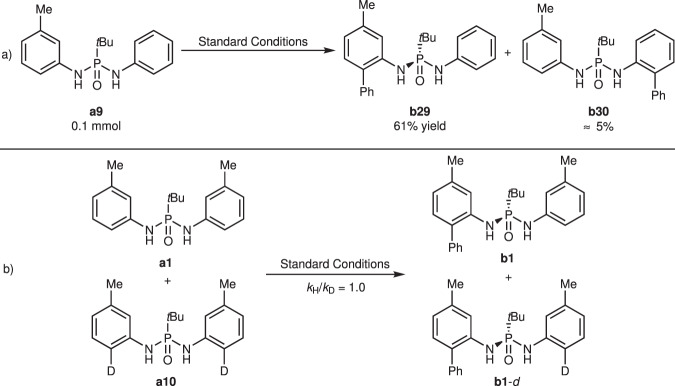
Mechanistic studies. **a** Intra-molecular competition experiment. **b** H/D Kinetic isotope effect (KIE) experiments.

As mentioned above, the electronic properties of the substrates had a substantial impact on the reactivities. To understand this reaction in more depth, unsymmetric phosphonic diamide **a9** was synthesized and subjected to standard conditions (Fig. 7a). As expected, arylation predominantly occurred on the *meta*-methyl-substituted phenyl ring, and **b29** was isolated in 61% yield, while arylated product **b30** with an unsubstituted phenyl ring was observed as a minor product. This differentiating behavior of the reaction system implies that the catalyst selectively interacts with the phenyl ring in an electrophilic activated manner. Additionally, this reaction represents a successful example of distinguishing between two similar phenyl rings with subtle differences in the electronic density, which is hard to achieve by other asymmetric catalytic systems.

**Fig. 8:**
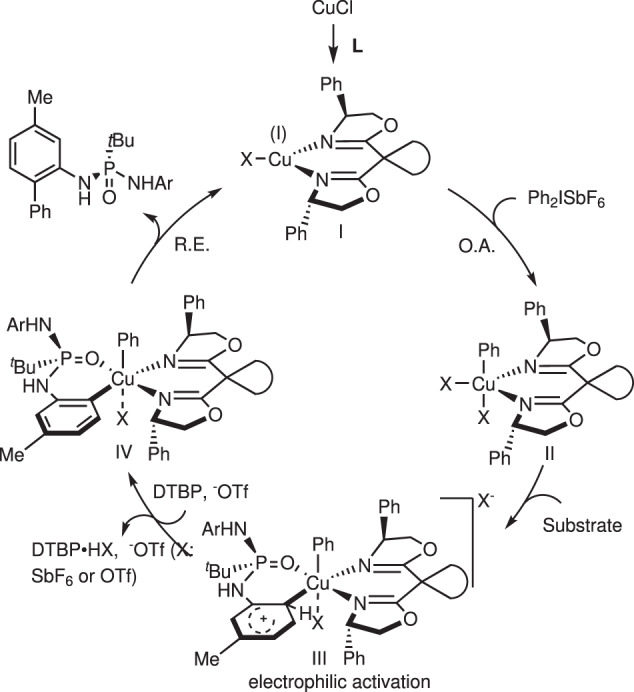
Proposed catalytic cycle. DTBP 2,6-di-*tert*-butylpyridine, O.A. oxidative addition, R.E. reductive elimination.

The kinetic isotope effect (KIE) was evaluated under standard conditions, and a value of 1.0 indicated that C–H bond abscission is not the rate-determining step in this reaction (Fig. 7b)^63^. Based on the observed results and literature reports^47,48^, we assumed that aryl–Cu(III) intermediate **II** was involved in the transformation, and this highly electrophilic complex, in a stereoselective and rate-limiting manner, activates an electron-rich phenyl ring with the aid of NaOTf to abstract H atoms and generate *ortho*-metalized intermediate **IV**, as depicted in Fig. 8. The whole C–H bond activation event determined the configuration of the product; however, more meticulous investigations are needed to elucidate the exact process of C–H bond activation and the origin of stereocontrol.

In summary, we have disclosed the Cu-catalyzed asymmetric arylation for the construction of *P*-chiral phosphonic diamines and axially chiral diarylphosphine oxide indoles with excellent enantioselectivities. Adequate but minimal NaOTf was found to be important for achieving the high conversion of substrates. Electronic effects are fairly obvious in this reaction, and an electrophilic aryl–Cu(III) intermediate was thereby assumed. The KIE experiment shows that C–H bond abscission is not the rate-determining step in this Cu-catalyzed arylation process. The gram-scale reaction proceeded smoothly when the catalyst loading was reduced to 1 mol%, implying potential industrial applications. Further investigations to fully understand the reaction and extension of this methodology to construct other optically active structures are underway in our lab.

## Methods

### General procedure for copper-bisoxazoline-catalyzed *ortho*-arylation

A dried Schlenk tube was charged with NaOTf/MeOH solution (c: 3.4 mg in 20 mL MeOH, 0.5 mL), and the solvent was removed under vacuum. CuCl (1.0 mg, 0.01 mmol), **L6** (6.9 mg, 0.015 mmol), and CH_2_Cl_2_ (2.0 mL) were added into the tube, and the mixture was stirred at room temperature for 0.5 h under N_2_. Substrate (0.1 mmol, 1.0 equiv.), diaryliodonium hexafluoroantimonate salt (0.15 mmol, 1.5 equiv.), and 2,6-di-*tert*-butylpyridine (33 μL, 0.15 mmol, 1.5 equiv.) were added into the mixture under N_2_. The reaction was heated to 60 °C and stirred for 48 h. After cooling to room temperature, the reaction mixture was filtrated, and the filtrate was concentrated under vacuum to remove all volatiles. The residue was purified via column chromatography on silica gel (petroleum ether/ethyl acetate = 5:1 to 2:1) to afford the product.

## Supplementary information

SUPPLEMENTARY INFO

## Data Availability

The data that support the findings of this study are available within the paper and its Supplementary Information files. Crystallographic parameters for compounds b18 and d1 are available free of charge from the Cambridge Crystallographic Data Centre under CCDC 2099084 and 2189868 (www.ccdc.cam.ac.uk/data_request/cif). All data are also available from authors upon request.
