# Supplementary material for: Copper-catalyzed asymmetric C(sp2)–H arylation for the synthesis of P- and axially chiral phosphorus compounds
Source: Nat Commun. 2023 Apr 20;14:2264. doi: 10.1038/s41467-023-37987-8 (PMC10119316; doi:10.1038/s41467-023-37987-8)
Supplement: Supplementary file 1 — SUPPLEMENTARY INFO [file 41467_2023_37987_MOESM1_ESM.pdf]

Supplementary Information

Copper-Catalyzed Asymmetric $C(sp^2)-H$ Arylation for the Synthesis of P- and Axially Chiral Phosphorus Compounds

Shao-Bai Yan[1], Rui Wang[1], Zha-Gen Li[1], An-Na Li[1], Chuanyong Wang[1] & Wei-Liang Duan*[,1,2,3]

[1]College of Chemistry and Chemical Engineering, Yangzhou University, 180 Siwangting Road, Yangzhou 225002, China
[2]College of Chemistry and Chemical Engineering, Inner Mongolia University, 235 West University Street, Hohhot 010021, China
[3]School of Chemistry and Chemical Engineering, Shaanxi Normal University, 620 Xi Changan Street, Xi'an 710119, China
*e-mail: duanwl@yzu.edu.cn

Table of Contents

1. Supplementary Methods **S3**

1.1 General Methods **S3**

1.2 Optimization of Reaction Conditions **S4**

1.3 Experimental Details and Characterization Data **S9**

1.4 Attempt on Utilization of Diphenylphosphine Oxide **S47**

1.5 Mechanistic Studies and Control Experiments **S50**

1.6 X-ray Structure of b18 and d1 **S55**

1.7 NMR spectra **S58**

1.8 HPLC charts **S171**

2. Supplementary References **S228**

1. Supplementary Methods

1.1 General Methods

All air- and moisture-sensitive manipulations were carried out with standard Schlenk techniques under nitrogen or in a glove box under nitrogen. ^{1}H, ^{13}C and ^{31}P NMR spectra were recorded on a Varian instrument (400 MHz and 100 MHz) or Bruker AVANCE 600 (600 MHz and 150 MHz). ^{1}H, ^{13}C NMR chemical shifts are reported vs tetramethylsilane signal or residual protio solvent signals. Data for ^{1}H NMR are recorded as follows: chemical shift (δ, ppm), multiplicity (s = singlet, d = doublet, t = triplet, sept = septet, br = broad singlet, coupling constant (*J*) in Hz, integration). Data for ^{13}C NMR are reported in terms of chemical shift (δ, ppm). Data for ^{31}P NMR are reported in terms of chemical shift (δ, ppm). High resolution mass spectra for new compounds were recorded at Mass Spectrometry Facilities of Yangzhou University. Enantiomeric excesses of products were determined on Waters HPLC system.

Bisoxazoline ligands were purchased from commercial sources or synthesized following literature procedures.[1] Substrates and diaryliodonium salts were prepared according to the literature procedures. All other chemicals with ≥ 98% purity were obtained from commercial suppliers (Aldrich, Alfa, J&K Scientific, shanghai Bidepharm and others) and used without further purification. All reactions were monitored by TLC or NMR analysis. Flash column chromatography was performed using 300–400 mesh silica gel.

1.2 Optimization of Reaction Conditions

Supplementary Table 1. Initial Screening of Arylation of Phosphonic Diamides[a]

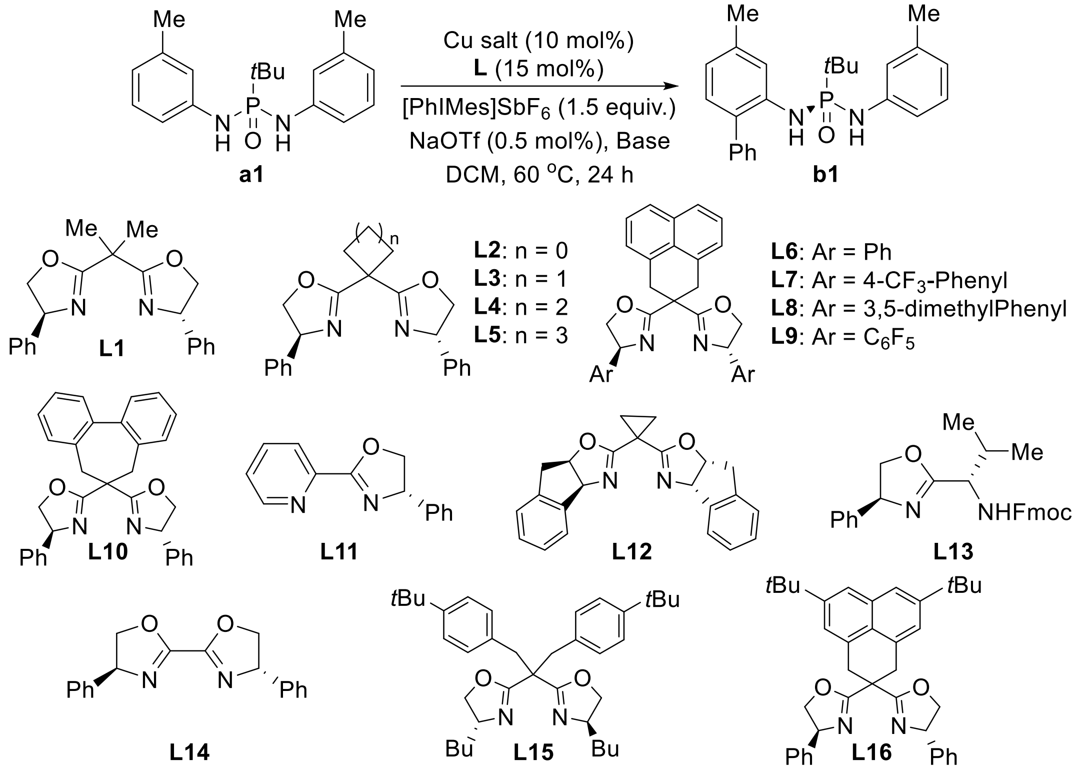

Entry	Cu. (10 mol%)	Ligand (mol%)	Base (1.5 equiv.)	Solvent (2 mL)	Additive	T (°C)	Yield[b] (%)	ee[c] (%)
1	CuI	**L1** (15)	/	DCM	/	60	50	10
2	CuI	**L2** (15)		DCM	/	60	16	56
3	CuI	**L3** (15)		DCM	/	60	15	52
4	CuI	**L4** (15)		DCM	/	60	trace	-
5	CuI	**L5** (15)		DCM	/	60	45	75
6	CuI	**L6** (15)		DCM	/	60	30	90
7	CuI	**L7** (15)		DCM	/	60	25	80
8	CuI	**L8** (15)		DCM	/	60	49	79
9	CuI	**L9** (15)		DCM	/	60	trace	-
10	CuI	**L10** (15)		DCM	/	60	10	69
11	CuI	**L11** (15)		DCM	/	60	15	20
12	CuI	**L12** (15)		DCM	/	60	n.r.	-
13	CuI	**L13** (15)		DCM	/	60	15	racemic
14	CuI	**L14** (15)		DCM	/	60	trace	-
15	CuI	**L15** (15)		DCM	/	60	n.r.	-
16	CuI	**L5** (15)	DTBP	DCM	/	60	n.r.	-

17	CuI	**L5** (15)	Na_2CO_3	DCM	/	60	17	8
18	CuI	**L5** (15)	DIPEA	DCM	/	60	n.r.	-
19	CuI	**L5** (15)	NaO*t*Bu	DCM	/	60	trace	-
20	CuI	**L5** (15)	Ph_2NMe	DCM	/	60	trace	-
21	CuI	**L5** (15)		DCE	/	60	trace	-
22	CuI	**L5** (15)		Toluene	/	60	30	57
23	CuI	**L5** (15)		EtOH	/	60	n.r.	
24	CuI	**L5** (15)		PhCl	/	60	43	73
25[d]	CuI	**L5** (15)		DCM	/	60	70	5
26[e]	CuI	**L5** (15)		DCM	/	60	15	-
27[f]	CuI	**L5** (15)		DCM	/	60	n.r.	5
28	CuI	**L5** (10)		DCM	/	60	30	73
29	CuI	**L5** (30)		DCM	/	60	25	77
30	CuI	**L5** (15)		DCM	/	80	47	70
31	CuI	**L5** (15)		DCM	/	40	20	74
32	CuI	**L6** (15)	Li_2CO_3	DCM	/	60	12	-
33	CuI	**L6** (15)	Cs_2CO_3	DCM	/	60	trace	-
34	CuI	**L6** (15)	DTBP	DCM	/	60	trace	-
35	CuOTf	**L6** (15)		DCM	/	60	44	35
36	$Cu(OTf)_2$	**L6** (15)		DCM	/	60	57	51
37	CuOTf	**L6** (15)	DTBP	DCM	/	60	23	85
38	CuOTf	**L6** (15)	K_2CO_3	DCM	/	60	22	82
39	$Cu(ACN)_4BF_4$	**L6** (15)		DCM	/	60	30	83
40	$Cu(ACN)_4PF_6$	**L6** (15)		DCM	/	60	10	-
41	$Cu(OAc)_2$	**L6** (15)		DCM	/	60	11	-
42	CuCl	**L6** (15)		DCM	/	60	40	91
43	CuCl	**L6** (15)	DTBP	DCM	/	60	trace	-
44	CuI	**L6** (15)	DTBP	DCM	CF_3CO_2H (20%)	60	trace	-
45	CuI	**L6** (15)	DTBP	DCM	*p*-TsOH (20%)	60	trace	-
46	CuI	**L6** (15)	DTBP	DCM	*p*-$TsNH_2$ (20%)	60	15	85
47	CuI	**L6** (15)	DTBP	DCM	$CF_3SO_2NH_2$ (20%)	60	10	-
48	CuI	**L6** (15)	DTBP	DCM	NaOTf (10%)	60	51	71
49	CuI	**L6** (15)	K_2CO_3	DCM	NaOTf (10%)	60	26	55
50	CuCl	**L6** (15)	DTBP	DCM	NaOTf (5%)	60	71	67
51	CuCl	**L6** (15)	DTBP	DCM	NaOTf (2%)	60	76	83
52	CuCl	**L6** (15)	DTBP	DCM	NaOTf (1%)	60	87	85

53	CuCl	**L6** (15)	DTBP	DCM	NaOTf (0.25%)	60	92	89
54	CuCl	**L6** (15)	DTBP	DCM	NaOTf (0.1%)	80	64	84
55	CuCl	**L6** (15)	DTBP	DCM	NaOTf (0.5%)	60	97	91
56[g]	CuCl	**L6** (15)	DTBP	DCM	NaOTf (0.5%)	60	97	90
57[g]	CuCl	**L16** (15)	DTBP	DCM	NaOTf (0.5%)	60	95	90

[a]Reaction conditions unless otherwise noted: **1a** (0.10 mmol), [PhIMes]SbF_6 (0.15 mmol), copper salt (0.01 mmol), ligand (0.015 mmol), Base (0.15 mmol), additives and DCM (2 mL) in a sealed tube at 60 °C for 24 h. [b]Isolated yields. [c]Determinded by chiral HPLC. [d][PhIMes]OTf used. [e][PhIMes]BF_4 used. [f][PhIMes]AsF_6 used. [g]Ph_2ISbF_6 used. DTBP: 2, 6-di-*tert*-butylpyridine. DIPEA: N, N-Diisopropylethylamine. n.r.: no reaction.

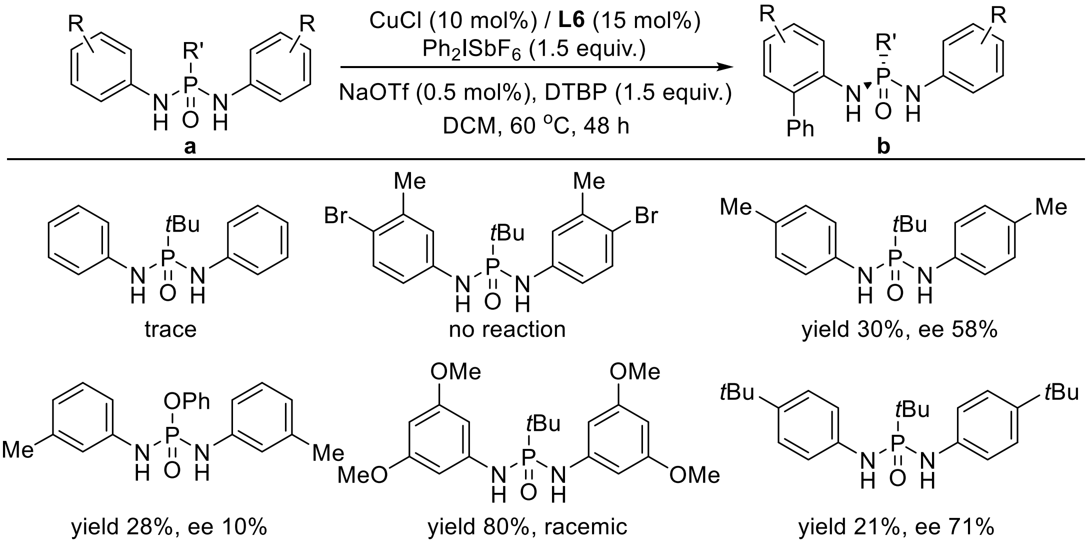

Supplementary Figure 1. Initial Screening of Substrates. Reaction conditions unless otherwise noted: **a** (0.10 mmol), Ph_2ISbF_6 (0.15 mmol), CuCl (0.01 mmol), **L6** (0.015 mmol), DTBP (0.15 mmol), NaOTf (0.5 mol%) and DCM (2 mL) in a sealed tube at 60 °C for 24 h. DTBP: 2, 6-di-*tert*-butylpyridine.

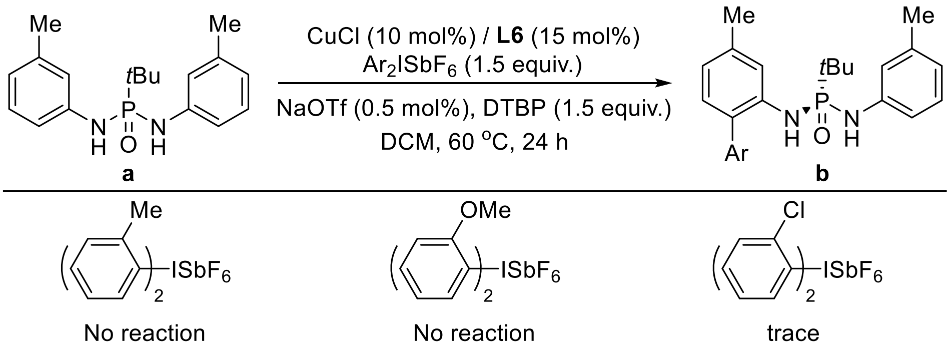

Supplementary Figure 2. Unsuccessful Diaryliodonium Salts. Reaction conditions: **a** (0.10 mmol), Ar_2ISbF_6 (0.15 mmol), CuCl (0.01 mmol), **L6** (0.015 mmol), DTBP (0.15 mmol), NaOTf (0.5 mol%) and DCM (2 mL) in a sealed tube at 60 °C for 24 h. DTBP: 2, 6-di-*tert*-butylpyridine.

Supplementary Table 2. Initial Screening of Arylation of biphenyl monophosphine oxides[a]

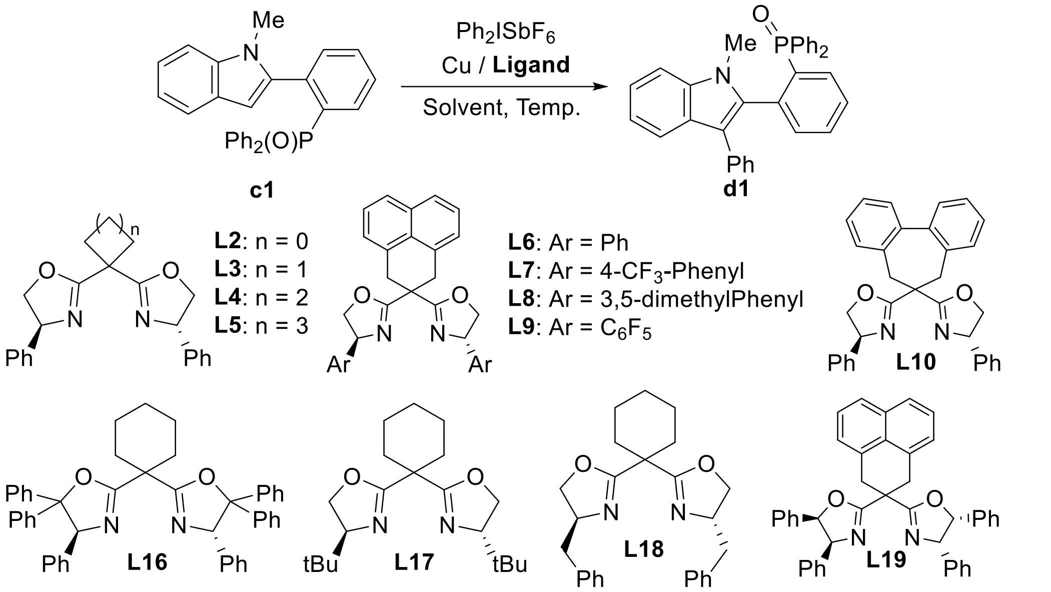

Entry	Cu. (10 mol%)	Ligand (mol%)	Base (equiv.)	Solvent (2 mL)	NaOTf (mol%)	T (°C)	Yield[b] (%)	ee[c] (%)
1	CuCl	**L6** (15)	DTBP/1.5	DCM	0.5%	60	18	88
2	CuI	**L2** (15)	DTBP/1.5	DCM	0.5%	60	80	28
3	CuI	**L3** (15)	DTBP/1.5	DCM	0.5%	60	85	60
4	CuI	**L4** (15)	DTBP/1.5	DCM	0.5%	60	trace	-
5	CuI	**L5** (15)	DTBP/1.5	DCM	0.5%	60	20	85
6	CuI	**L7** (15)	DTBP/1.5	DCM	0.5%	60	30	12
7	CuI	**L8** (15)	DTBP/1.5	DCM	0.5%	60	20	82
8	CuI	**L9** (15)	DTBP/1.5	DCM	0.5%	60	85	racemic
9	CuI	**L10** (15)	DTBP/1.5	DCM	0.5%	60	trace	-
10	CuI	**L6** (15)	DTBP/1.5	DCE	0.5%	60	50	81
11	CuI	**L6** (15)	DTBP/1.5	THF	0.5%	60	18	57
12	CuI	**L6** (15)	DTBP/1.5	EA	0.5%	60	15	68
13	CuI	**L6** (15)	DTBP/1.5	Toluene	0.5%	60	50	80
14[d]	CuI	**L6** (15)	DTBP/2	DCM	0.5%	60	68	88
15[d]	CuCl	**L6** (15)	DTBP/2	DCM	0.5%	60	64	87
16[d]	CuBr	**L6** (15)	DTBP/2	DCM	0.5%	60	68	86
17[d]	$Cu(OAc)_2$	**L6** (15)	DTBP/2	DCM	0.5%	60	68	88
18[d]	$Cu(ACN)_4BF_4$	**L6** (15)	DTBP/2	DCM	0.5%	60	54	85
19[d]	CuI	**L6** (15)	DTBP/2	DCM	1%	60	78	88
20[d]	CuI	**L6** (15)	DTBP/2	DCM	2%	60	62	85
21[e]	CuI	**L6** (15)	DTBP/2.5	DCM	1%	60	78	88

22[e]	CuI	**L6** (15)	DTBP/2.5	DCM	1%	50	74	88
23[d]	CuI	**L16** (15)	DTBP/2	DCM	1%	60	30	87
24[d]	CuI	**L17** (15)	DTBP/2	DCM	1%	60	n.r.	-
25[d]	CuI	**L18** (15)	DTBP/2	DCM	1%	60	70	27
26[d]	CuI	**L19** (15)	DTBP/2	DCM	1%	60	60	15

[a]Reaction conditions unless otherwise noted: **c1** (0.10 mmol), Ph_2ISbF_6 (0.15 mmol), copper salt (0.01 mmol), ligand (0.015 mmol), Base (0.15 mmol), additives and DCM (2 mL) in a sealed tube at 60 °C for 24 h. [b]Isolated yields. [c]Determinded by chiral HPLC. [d]2.0 equiv. Ph_2ISbF_6 used. [e]2.5 equiv. Ph_2ISbF_6 used. n.r.: no reaction. DTBP: 2, 6-di-*tert*-butylpyridine.

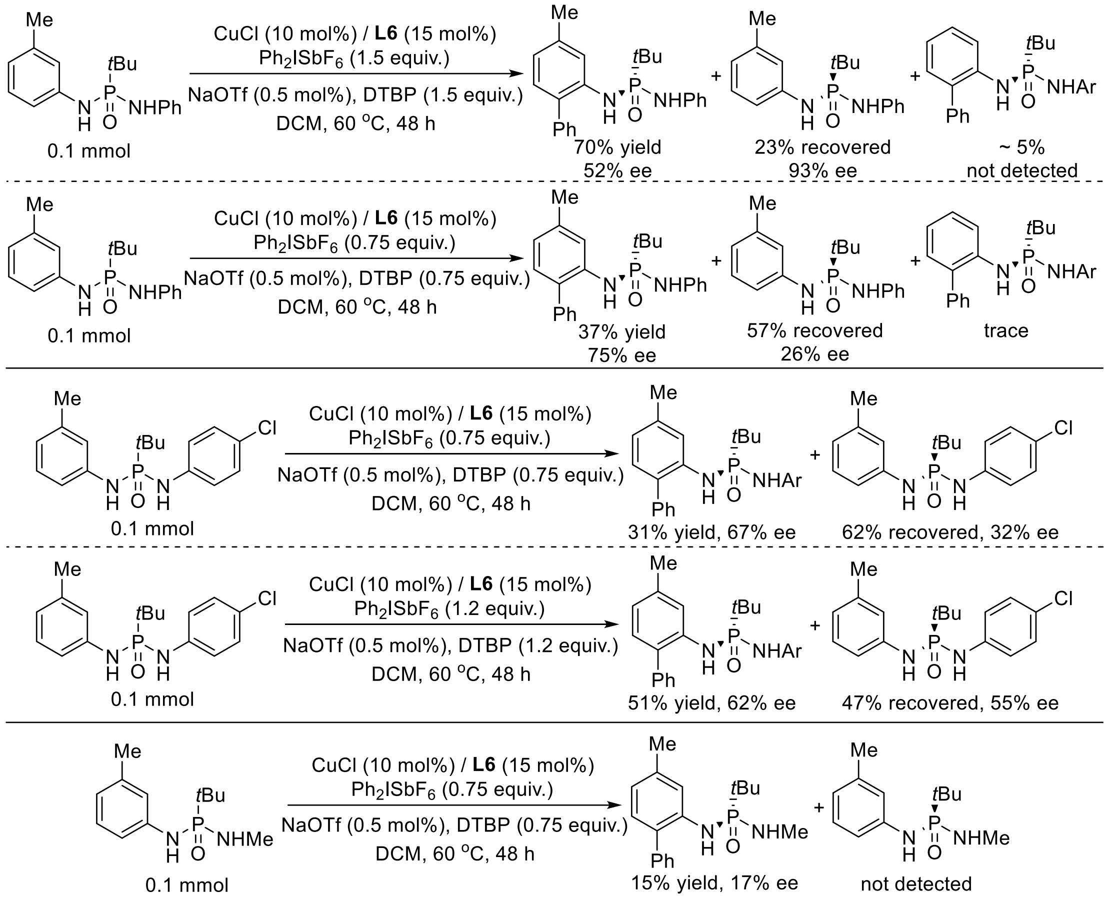

Supplementary Figure 3. Trials on Kinetic Resolution of Unsymmetric Phosphonic Diamides. DTBP: 2, 6-di-*tert*-butylpyridine.

1.3 Experimental Details and Characterization Data

Synthesis of Diaryliodonium Salts

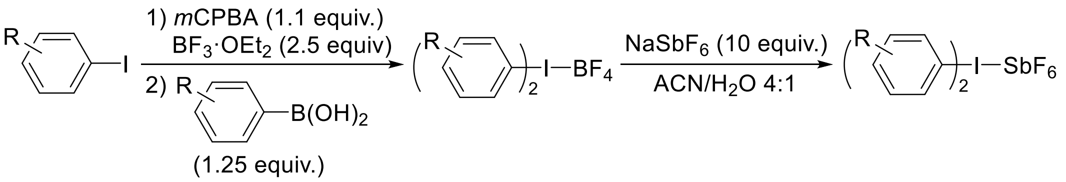

Procedure A: According to the literature.[2] 3-Chloroperoxybenzoic acid (81% active oxidant, 5.5 mmol) was dissolved in CH_2Cl_2 (10 mL). To the solution was added aryl iodide (5 mmol) followed by BF_3•OEt_2 (1.5 mL, 12.5 mmol) at room temperature. The resulting solution was stirred at rt for 30 min and then cooled to 0 °C, and arylboronic acid (6.25 mmol) was added. After 15 min of stirring at rt, the crude reaction mixture was applied on a silica plug and eluted with CH_2Cl_2 to remove unreacted ArI, followed by CH_2Cl_2/MeOH (60 mL, 20:1) to elute the product. The latter solution was concentrated, and diethyl ether was added to the residue to induce a precipitation of salt. The solution was allowed to stir for 30 min, and then the ether phase was decanted, and the solid was washed twice more with diethyl ether and then dried in vacuo to give diaryliodonium tetrafluoroborate salt.

Dissolve diaryliodonium tetrafluoroborate salt with CH_3CN/H_2O (4:1 v/v, 50 mL), then charge $NaSbF_6$ (10 equiv.) into the mixture and stir at rt for 24 h. Dilute with water and extract with CH_2Cl_2 for twice. The organic layer was dried over Na_2SO_4 and concentrate under vacuum. Diethyl ether was added to the residue to induce a precipitation of salt. The solution was allowed to stir for 30 min, and then the ether phase was decanted, and the solid was washed twice more with diethyl ether and then dried in vacuo to give diaryliodonium hexafluoroantimonate salt.

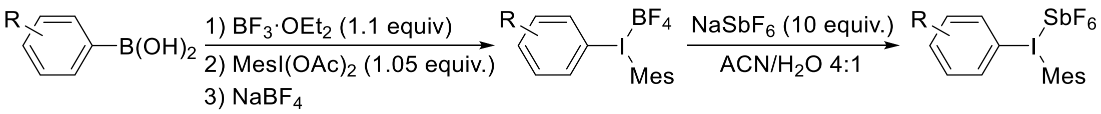

Procedure B: According to the literature.[3] BF_3•OEt_2 (6.95 mmol) was added to a stirred solution of the appropriate boronic acid (6.32 mmol) in CH_2Cl_2 (100 ml) at 0 °C and the solution stirred for 10 min before addition of a solution of 2-iodo-1,3,5-trimethylbenzene diacetate (6.64 mmol) in CH_2Cl_2 (20 ml) dropwise over 10 mins. The reaction was allowed to warm to rt over the course of 2 h and then 100 mL saturated aq. $NaBF_4$ solution was added with rapid stirring and the stirring continued for 30 mins. After this time the phases were separated, the aqueous layer extracted twice with CH_2Cl_2 and the combined organics dried over $MgSO_4$ and evaporated. The iodonium tetrafluoroborate was precipitated from the crude residue by addition of Et_2O. The solid was filtered, washed with Et_2O and dried under vacuum.

Dissolve the precipitate with CH_3CN/H_2O (4:1 v/v, 50 mL), then charge $NaSbF_6$ (10 equiv.) into the mixture and stir at rt for 24 h. Dilute with water and extract with CH_2Cl_2 for twice. The organic layer was dried over Na_2SO_4 and concentrate under vacuum. Diethyl ether was added to the residue to induce a precipitation of salt. The solution was allowed to stir for 30

min, and then the ether phase was decanted, and the solid was washed twice more with diethyl ether and then dried in vacuo to give corresponding diaryliodonium hexafluoroantimonate salt.

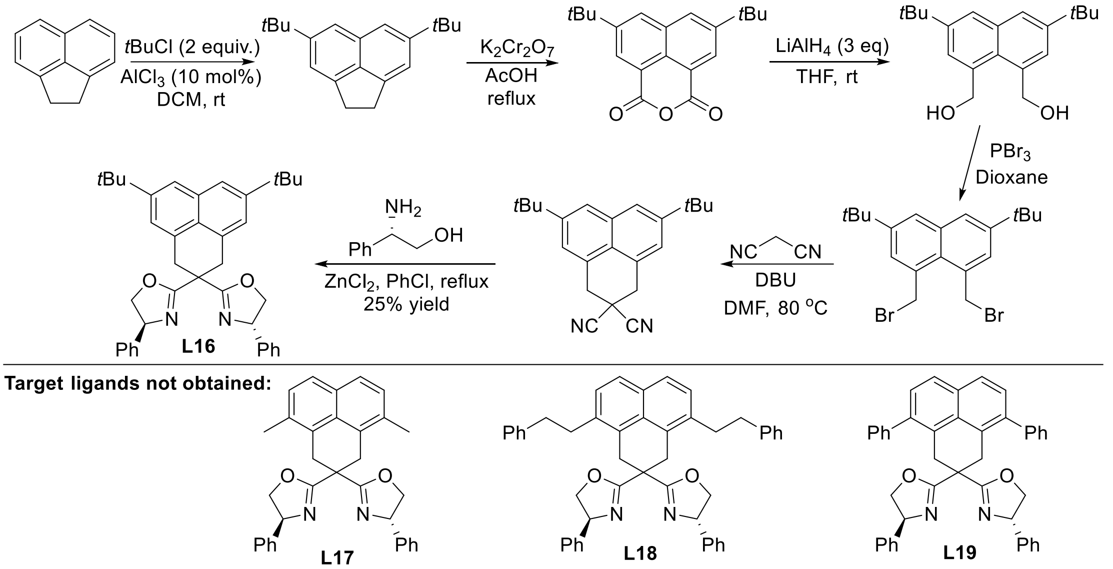

Supplementary Figure 4. Synthesis of Ligand **L16**. For synthetic steps 1-2 of **L16**, acid anhydride was prepared according to literature[4]; and for steps 3-5 general procedures in reference 1 were followed. Meanwhile, efforts on *ortho*-substituted ligands (**L17-19**) ended in failure.

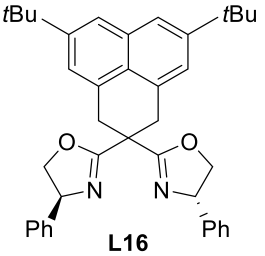

L16

White solid. 210 mg, 25% yield. **^{1}H NMR** (400 MHz, $CDCl_3$): δ 7.63 (d, J = 1.5 Hz, 2H), 7.35 (brs, 2H), 7.18 – 7.10 (m, 6H), 6.83 – 6.76 (m, 4H), 5.16 (dd, J = 10.1, 7.7 Hz, 2H), 4.59 (dd, J = 10.1, 8.3 Hz, 2H), 3.95 (t, J = 8.0 Hz, 2H), 3.83 (q, J = 15.6 Hz, 4H), 1.37 (s, 18H). **^{13}C NMR** (101 MHz, $CDCl_3$): δ 168.26, 148.58, 142.33, 133.31, 132.00, 128.51, 127.26, 126.43, 125.51, 122.85, 121.29, 75.49, 69.54, 43.01, 38.04, 34.76, 31.33.

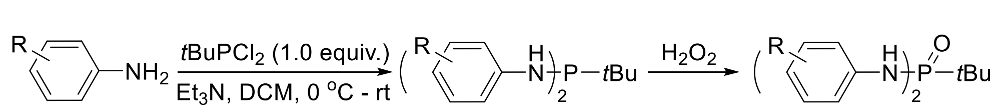

General Procedure for the Preparation of Substrate a. To a dried 100 mL round bottom flask was charged with *t*BuPCl$_2$ (0.7 mL, 5 mmol) and CH_2Cl_2 (25 mL), then cool the mixture to 0 ºC. Et_3N (2.1 mL, 3.0 equiv.) was added into the reaction at 0 ºC followed by arylamine (12.5 mmol, 2.5 equiv.). After stirring overnight at room temperature, water was added and the phases were separated. The aqueous layer extracted twice with CH_2Cl_2 and the combined organics dried over Na_2SO_4. After concentrated to about 30 mL, 30% H_2O_2 (0.65 mL, 1.1

equiv.) was added to the mixture at 0 °C followed by stirring at ambient temperature until disappear of the corresponding phosphine (Monitored by TLC). After quenched the reaction with $Na_2S_2O_3$, the mixture was extracted with DCM for three times. The solution was dried with Na_2SO_4 and the crude product was purified via flash chromatography (PE/EA 5:1 to 1:1, v/v) or recrystallization in CH_2Cl_2 to afford a white solid.

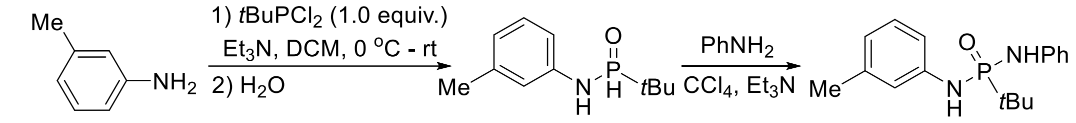

Procedure for the Preparation of Unsymmetric Substrate a10. To a dried 100 mL round bottom flask was charged with *t*BuPCl$_2$ (0.7 mL, 5 mmol) and CH_2Cl_2 (15 mL), then cool the mixture to 0 °C. Et_3N (1 mL, 1.5 equiv.) was added into the reaction at 0 °C followed by slow dropping of 3-toluidine (5.25 mmol, 1.05 equiv.) solution in CH_2Cl_2 (15 mL). After stirring at room temperature for 4-5 h, water (0.1 mL, 1.2 equiv.) was added and stirred for overnight. The reaction was quenched with water and the phases were separated. The aqueous layer was extracted twice with CH_2Cl_2 and dried over Na_2SO_4. The crude product was purified via flash chromatography (PE/EA 5:1 to 1:1, v/v) to afford a white solid in 50% yield. The solid was dissolved with CCl_4 (10 mL), then Et_3N (0.5 mL, 1.5 equiv.) and aniline (0.34 mL, 1.5 equiv.) were added into the mixture and stirred for overnight at room temperature. The mixture was concentrated under vacuum and the crude product was purified via flash chromatography (PE/EA 5:1 to 1:1, v/v) to afford a white solid in 73% yield.

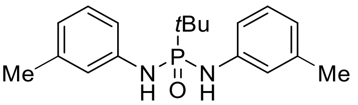

***P-tert*-butyl-*N, N*'-di(3-methylphenyl)phosphonic diamide (a1)**. White solid. 1.18 g, 75% yield. **^{1}H NMR** (400 MHz, $CDCl_3$): δ 7.10 (t, *J* = 8.0 Hz, 2H), 7.02 – 6.95 (m, 4H), 6.76 (d, *J* = 7.5 Hz, 2H), 4.74 (d, *J* = 11.7 Hz, 2H), 2.27 (s, 6H), 1.29 (d, *J* = 16.0 Hz, 9H). **^{13}C NMR** (101 MHz, DMSO-*d*$_6$): δ 143.60, 137.92, 128.80, 121.18, 119.05 (d, J_{CP} = 6.0 Hz), 115.61 (d, J_{CP} = 5.5 Hz), 34.38 (d, J_{CP} = 114.4 Hz), 25.08, 21.71. **^{31}P NMR** (162 MHz, DMSO-*d*$_6$): δ 28.6. **HRMS** (ESI) calcd for $C_{18}H_{25}N_2NaOP$ $(M+Na)^+$: 339.1597, found: 339.1588.

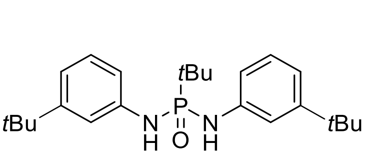

***P-tert*-butyl-*N, N*'-di(3-*tert*-butylphenyl)phosphonic diamide (a2)**. White solid. 1.36 g, 68% yield. **^{1}H NMR** (400 MHz, $CDCl_3$): δ 7.15 (t, *J* = 8.0 Hz, 2H), 7.12 (s, 2H), 7.07 (d, *J* = 8.1 Hz, 2H), 6.97 (d, *J* = 7.7 Hz, 2H), 4.76 (d, *J* = 11.6 Hz, 2H), 1.31 (d, *J* = 16.0 Hz, 9H), 1.24 (s, 18H). **^{13}C NMR** (101 MHz, $CDCl_3$): δ 152.34, 140.51 (d, J_{CP} = 1.7 Hz), 128.81, 118.84, 115.96 (d, J_{CP} = 5.8 Hz), 115.87 (d, J_{CP} = 5.0 Hz), 34.61, 34.36 (d, J_{CP} = 115.6 Hz), 31.23, 25.46. **^{31}P NMR** (162 MHz, $CDCl_3$): δ 27.8. **HRMS** (ESI) calcd for $C_{24}H_{37}N_2NaOP$ $(M+Na)^+$: 423.2536, found: 423.2522.

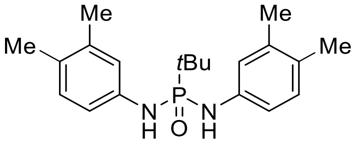

***P-tert*-butyl-*N*, *N*'-di(3, 4-dimethylphenyl)phosphonic diamide (a3)**. White solid. 1.29 g, 76% yield. **^{1}H NMR** (400 MHz, $CDCl_3$): δ 6.99 – 6.90 (m, 6H), 4.61 (d, *J* = 11.6 Hz, 2H), 2.18 (s, 3H), 2.16 (s, 3H), 1.28 (d, *J* = 15.9 Hz, 9H). **^{13}C NMR** (101 MHz, DMSO-d_6): δ 141.25, 136.27, 129.87, 127.74, 119.95 (d, J_{CP} = 6.0 Hz), 116.01 (d, J_{CP} = 5.3 Hz), 34.32 (d, J_{CP} = 114.4 Hz), 25.17, 20.12, 18.94. **^{31}P NMR** (162 MHz, DMSO-d_6): δ 28.5. **HRMS** (ESI) calcd for $C_{20}H_{29}N_2NaOP$ $(M+Na)^+$: 367.1910, found: 367.1900.

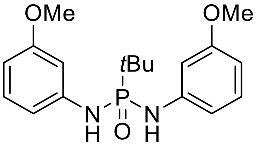

***P-tert*-butyl-*N*, *N*'-di(3-methoxyphenyl)phosphonic diamide (a4)**. White solid. 1.27 g, 73% yield. **^{1}H NMR** (400 MHz, $CDCl_3$): δ 7.11 (t, *J* = 8.2 Hz, 2H), 6.81 (s, 2H), 6.75 (d, *J* = 8.0 Hz, 2H), 6.51 (d, *J* = 8.2 Hz, 2H), 4.80 (d, *J* = 11.8 Hz, 2H), 3.73 (s, 6H), 1.30 (d, *J* = 16.1 Hz, 9H). **^{13}C NMR** (101 MHz, DMSO-d_6): δ 160.06, 144.85, 129.68, 111.01 (d, J_{CP} = 5.9 Hz), 105.81, 104.38 (d, J_{CP} = 5.8 Hz), 55.16, 34.32 (d, J_{CP} = 114.4 Hz), 24.96. **^{31}P NMR** (162 MHz, DMSO-d_6): δ 28.7. **HRMS** (ESI) calcd for $C_{18}H_{25}N_2NaO_3P$ $(M+Na)^+$: 371.1495, found: 371.1485.

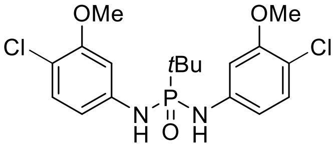

***P-tert*-butyl-*N*, *N*'-di(3-methoxy-4-chlorophenyl)phosphonic diamide (a5)**. White solid. 1.7 g, 80% yield. **^{1}H NMR** (400 MHz, $CDCl_3$): δ 7.18 (d, *J* = 8.4 Hz, 2H), 6.99 (d, *J* = 2.4 Hz, 2H), 6.61 (dd, *J* = 8.4, 2.4 Hz, 2H), 4.77 (d, *J* = 12.0 Hz, 2H), 3.81 (s, 6H), 1.31 (d, *J* = 16.3 Hz, 9H). **^{13}C NMR** (101 MHz, DMSO-d_6): δ 154.86, 143.96, 129.95, 112.33, 111.05 (d, J_{CP} = 5.7 Hz), 103.21 (d, J_{CP} = 6.1 Hz), 56.02, 34.28 (d, J_{CP} = 114.4 Hz), 24.79. **^{31}P NMR** (162 MHz, DMSO-d_6): δ 29.4. **HRMS** (ESI) calcd for $C_{18}H_{23}Cl_2N_2NaO_3P$ $(M+Na)^+$: 439.0716, found: 439.0703.

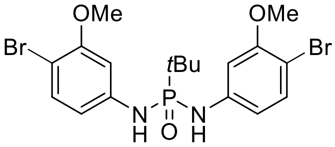

***P-tert*-butyl-*N*, *N*'-di(3-methoxy-4-bromophenyl)phosphonic diamide (a6)**. White solid. 1.9 g, 77% yield. **^{1}H NMR** (400 MHz, $CDCl_3$): δ 7.33 (d, *J* = 8.4 Hz, 2H), 6.97 (d, *J* = 2.4 Hz, 2H), 6.57 (dd, *J* = 8.4, 2.4 Hz, 2H), 4.82 (d, *J* = 11.9 Hz, 2H), 3.80 (s, 6H), 1.31 (d, *J* = 16.3 Hz, 9H). **^{13}C NMR** (101 MHz, DMSO-d_6): δ 155.77, 144.66, 132.89, 111.76 (d, J_{CP} = 5.7 Hz), 103.18 (d, J_{CP} = 6.5 Hz), 101.03, 56.14, 34.29 (d, J_{CP} = 114.3 Hz), 24.79. **^{31}P NMR** (162 MHz,

DMSO-d_6): δ 29.4. **HRMS** (ESI) calcd for $C_{18}H_{23}Br_2N_2NaO_3P$ $(M+Na)^+$: 526.9705, found: 526.9714.

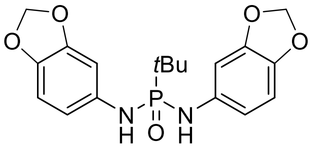

***P-tert*-butyl-*N, N*'-di(3,4-methylenedioxyphenyl)phosphonic diamide (a7)**. Brown solid. 1.24 g, 66% yield. **^{1}H NMR** (400 MHz, $CDCl_3$): δ 6.86 (d, J = 2.2 Hz, 2H), 6.66 (d, J = 8.2 Hz, 2H), 6.56 (dd, J = 8.2, 2.2 Hz, 2H), 5.90 (s, 4H), 4.60 (d, J = 11.5 Hz, 2H), 1.27 (d, J = 16.0 Hz, 9H). **^{13}C NMR** (101 MHz, DMSO-d_6): δ 147.53, 141.29, 138.03, 110.75 (d, J_{CP} = 6.3 Hz), 108.48, 100.90, 100.83 (d, J_{CP} = 5.2 Hz), 34.22 (d, J_{CP} = 113.9 Hz), 25.03. **^{31}P NMR** (243 MHz, $CDCl_3$): δ 33.3. **HRMS** (ESI) calcd for $C_{18}H_{21}N_2NaO_5P$ $(M+Na)^+$: 399.1080, found: 399.1066.

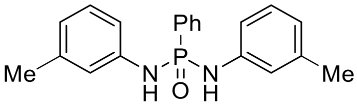

***P*-phenyl-*N, N*'-di(3-methylphenyl)phosphonic diamide (a8)**. Started with $PhPCl_2$. White solid. 1.2 g, 70% yield. **^{1}H NMR** (400 MHz, $CDCl_3$): δ 7.95 (d, J = 7.7 Hz, 1H), 7.92 (d, J = 8.0 Hz, 1H), 7.56 (t, J = 7.5 Hz, 1H), 7.51 – 7.43 (m, 2H), 7.08 (t, J = 7.7 Hz, 2H), 6.92 (s, 2H), 6.89 (d, J = 8.2 Hz, 2H), 6.77 (d, J = 7.5 Hz, 2H), 5.15 (d, J = 9.9 Hz, 2H), 2.25 (s, 6H). **^{13}C NMR** (101 MHz, DMSO-d_6): δ 142.61, 138.23, 133.43 (d, J_{CP} = 153.2 Hz), 132.17 (d, J_{CP} = 3.5 Hz), 132.10 (d, J_{CP} = 10.6 Hz), 129.02, 128.80 (d, J_{CP} = 13.6 Hz), 121.52, 118.84 (d, J_{CP} = 6.9 Hz), 115.48 (d, J_{CP} = 6.8 Hz), 21.70. **^{31}P NMR** (162 MHz, DMSO-d_6): δ 9.1. **HRMS** (ESI) calcd for $C_{20}H_{21}N_2NaOP$ $(M+Na)^+$: 359.1284, found: 359.1272.

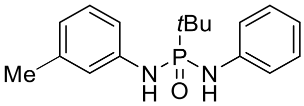

***P-tert*-butyl-*N*-(3-methylphenyl)-*N*'-phenylphosphonic diamide (a9)**. White solid. 0.55 g, 73% yield. **^{1}H NMR** (400 MHz, $CDCl_3$): δ 7.25 – 7.16 (m, 4H), 7.10 (t, J = 8.1 Hz, 1H), 7.02 – 6.97 (m, 2H), 6.95 (t, J = 7.0 Hz, 1H), 6.77 (d, J = 7.5 Hz, 1H), 4.77 (d, J = 12.7 Hz, 1H), 4.73 (d, J = 12.5 Hz, 1H), 2.27 (s, 3H), 1.30 (d, J = 16.1 Hz, 9H). **^{13}C NMR** (101 MHz, DMSO-d_6): δ 143.65, 143.59, 137.96, 128.96, 128.81, 121.20, 120.40, 119.00 (d, J_{CP} = 5.8 Hz), 118.39 (d, J_{CP} = 5.7 Hz), 115.56 (d, J_{CP} = 5.5 Hz), 34.37 (d, J_{CP} = 114.4 Hz), 25.04, 21.71. **^{31}P NMR** (162 MHz, DMSO-d_6): δ 28.6. **HRMS** (ESI) calcd for $C_{17}H_{23}N_2NaOP$ $(M+Na)^+$: 325.1440, found: 325.1438.

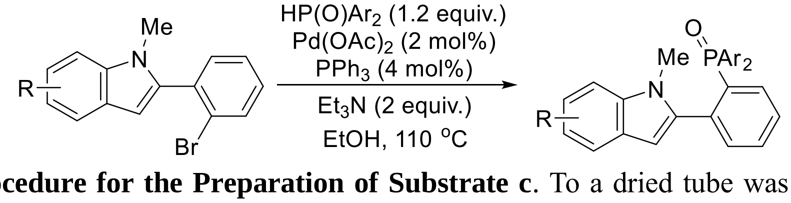

General Procedure for the Preparation of Substrate c. To a dried tube was charged with

2-(2-bromophenyl)-1-methyl-1*H*-indole (5 mmol, 1.0 equiv.), diarylphosphine oxide (6 mmol, 1.2 equiv.), $Pd(OAc)_2$ (22 mg, 2 mol%), PPh_3 (52 mg, 4 mol%), Et_3N (1.4 mL, 10 mmol, 2 equiv.) and EtOH (15 mL), then seal the tube and heat to 110 ºC for 20 - 24h. Dilute with CH_2Cl_2, wash with water and dried over Na_2SO_4. The crude product was purified via flash chromatography (PE/EA 5:1 to 1:1, v/v) to afford the product.

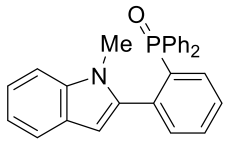

(2-(1-methyl-1*H*-indol-2-yl)phenyl)diphenylphosphine oxide (c1). Known compound.[5] White solid. 1.6 g, 77% yield. **^{1}H NMR** (400 MHz, $CDCl_3$) δ 8.02 (dd, J = 12.9, 7.8 Hz, 1H), 7.63 (t, J = 7.5 Hz, 1H), 7.56 (t, J = 7.6 Hz, 1H), 7.53 (s, 1H), 7.48 (s, 1H), 7.38 (d, J = 7.8 Hz, 2H), 7.34 (dd, J = 7.5, 4.4 Hz, 4H), 7.22 (s, 2H), 7.13 (t, J = 7.5 Hz, 3H), 7.02 (t, J = 7.4 Hz, 1H), 6.97 (d, J = 8.2 Hz, 1H), 6.19 (s, 1H), 2.98 (s, 3H).

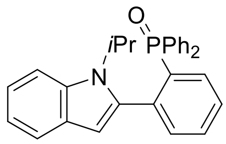

(2-(1-isopropyl-1*H*-indol-2-yl)phenyl)diphenylphosphine oxide (c2). White solid. 0.89 g, 41% yield. **^{1}H NMR** (400 MHz, $CDCl_3$) δ 7.79 (ddd, *J* = 13.2, 7.8, 1.4 Hz, 1H), 7.63 – 7.55 (m, 3H), 7.49 (ddd, *J* = 9.6, 5.6, 1.5 Hz, 1H), 7.44 (ddd, *J* = 7.3, 4.1, 1.3 Hz, 3H), 7.41 – 7.33 (m, 4H), 7.31 (d, *J* = 7.8 Hz, 1H), 7.20 (td, *J* = 7.7, 3.1 Hz, 2H), 7.09 (ddd, *J* = 8.4, 7.0, 1.3 Hz, 1H), 6.99 (ddd, *J* = 7.9, 7.0, 1.0 Hz, 1H), 5.80 (s, 1H), 4.15 (hept, *J* = 7.0 Hz, 1H), 1.45 (d, *J* = 7.0 Hz, 3H), 1.28 (d, *J* = 7.0 Hz, 3H). **^{13}C NMR** (101 MHz, $CDCl_3$) δ 137.77 (d, J_{CP} = 8.0 Hz), 137.29, 137.25, 134.46, 134.26 (d, J_{CP} = 10.7 Hz), 133.91 (d, J_{CP} = 102.0 Hz), 133.74 (d, J_{CP} = 102.6 Hz), 132.97 (d, J_{CP} = 9.5 Hz), 132.70 (d, J_{CP} = 102.8 Hz), 132.07 (d, J_{CP} = 9.6 Hz), 131.77 (d, J_{CP} = 9.7 Hz), 131.47 (d, J_{CP} = 2.8 Hz), 131.29 (d, J_{CP} = 2.7 Hz), 131.24 (d, J_{CP} = 2.4 Hz), 128.41, 128.30 (d, J_{CP} = 12.2 Hz), 127.99 (d, J_{CP} = 12.2 Hz), 127.90 (d, J_{CP} = 12.0 Hz), 120.97, 120.92, 118.91, 112.06, 105.13, 48.17, 21.94, 20.61. **^{31}P NMR** (162 MHz, $CDCl_3$): δ 28.2. **HRMS** (ESI) calcd for $C_{29}H_{26}NNaOP$ $(M+Na)^+$: 458.1644, found: 458.1639.

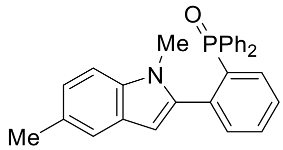

(2-(1,5-dimethyl-1*H*-indol-2-yl)phenyl)diphenylphosphine oxide (c3). Known compound.[5] White solid. 0.95 g, 45% yield. **^{1}H NMR** (400 MHz, $CDCl_3$) δ 8.08 (ddd, *J* = 13.0, 7.7, 1.2 Hz, 1H), 7.67 (ddd, *J* = 7.5, 4.5, 1.5 Hz, 1H), 7.60 (ddd, *J* = 7.8, 4.8, 1.8 Hz, 2H), 7.56–7.46 (m, 2H), 7.43–7.28 (m, 6H), 7.25 (s, 1H), 7.15 (s, 2H), 6.96 (d, *J* = 8.3 Hz, 1H), 6.86 (d, *J* = 8.3 Hz, 1H), 6.06 (s, 1H), 2.89 (s, 3H), 2.41 (s, 3H).

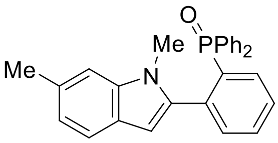

(2-(1,6-dimethyl-1*H*-indol-2-yl)phenyl)diphenylphosphine oxide (c4). White solid. 0.63 g, 30% yield. **^{1}H NMR** (400 MHz, $CDCl_3$) δ 8.00 (ddd, *J* = 13.0, 7.7, 1.5 Hz, 1H), 7.60 (tt, *J* = 7.5, 1.6 Hz, 1H), 7.54 (tt, *J* = 7.7, 1.7 Hz, 1H), 7.42 (s, 4H), 7.36 – 7.27 (m, 4H), 7.22 (s, 1H), 7.16 (s, 3H), 6.84 (dd, *J* = 8.1, 1.4 Hz, 1H), 6.76 (s, 1H), 6.09 (s, 1H), 2.92 (s, 3H), 2.44 (s, 3H). **^{13}C NMR** (101 MHz, $CDCl_3$) δ 137.04, 136.75 (d, J_{CP} = 8.8 Hz), 136.25 (d, J_{CP} = 4.6 Hz), 134.28 (d, J_{CP} = 10.1 Hz), 134.09 (d, J_{CP} = 102.3 Hz), 132.77 (d, *J* = 9.5 Hz), 131.61 (brs), 131.43 (d, *J* = 2.5 Hz), 131.41, 131.16 (d, *J* = 2.0 Hz), 128.16 (d, *J* = 11.7 Hz), 127.78 (d, *J* = 12.2 Hz), 125.07, 121.15, 120.23, 109.07, 105.46, 30.00, 21.96. **^{31}P NMR** (162 MHz, $CDCl_3$): δ 28.0. **HRMS** (ESI) calcd for $C_{28}H_{24}NNaOP$ $(M+Na)^+$: 444.1488, found: 444.1482.

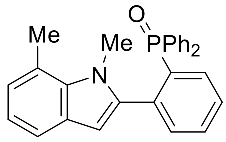

(2-(1,7-dimethyl-1*H*-indol-2-yl)phenyl)diphenylphosphine oxide (c5). Known compound.[5] White solid. 0.76 g, 36% yield. **^{1}H NMR** (400 MHz, $CDCl_3$) δ 8.11 (ddd, *J* = 12.9, 7.5, 1.7 Hz, 1H), 7.60 (ddt, *J* = 9.3, 7.3, 1.8 Hz, 2H), 7.51 (dd, *J* = 12.0, 10.5 Hz, 2H), 7.45 – 7.38 (m, 1H), 7.35 – 7.26 (m, 5H), 7.26 – 7.23 (m, 1H), 7.16 (d, *J* = 7.8 Hz, 1H), 7.03 (t, *J* = 7.5 Hz, 2H), 6.86 (t, *J* = 7.4 Hz, 1H), 6.80 (d, *J* = 7.0 Hz, 1H), 6.04 (s, 1H), 3.11 (s, 3H), 2.52 (s, 3H).

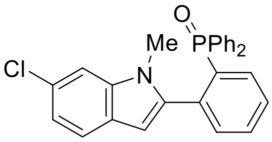

(2-(1-methyl-6-chloro-1*H*-indol-2-yl)phenyl)diphenylphosphine oxide (c6). Yellow solid. 1.1 g, 51% yield. **^{1}H NMR** (400 MHz, $CDCl_3$) δ 7.89 (ddd, *J* = 13.2, 7.6, 1.5 Hz, 1H), 7.64 (tt, *J* = 7.6, 1.5 Hz, 1H), 7.55 (tt, *J* = 7.8, 1.8 Hz, 1H), 7.46 (s, 4H), 7.40 – 7.32 (m, 3H), 7.29 (d, *J* = 8.9 Hz, 1H), 7.21 (s, 4H), 7.03 – 6.96 (m, 2H), 6.23 (s, 1H), 3.02 (s, 3H). **^{13}C NMR** (101 MHz, $CDCl_3$) δ 137.67 (d, J_{CP} =4.5 Hz), 137.05, 136.08 (d, J_{CP} = 8.4 Hz), 134.31 (d, J_{CP} = 10.3 Hz), 134.26 (d, J_{CP} = 102.0 Hz), 132.61 (d, J_{CP} = 9.3 Hz), 131.74 (brs), 131.56 (d, J_{CP} = 2.4 Hz), 131.29 (d, J_{CP} = 2.7 Hz), 128.45 (d, J_{CP} = 11.8 Hz), 127.85 (d, J_{CP} = 12.2 Hz), 127.44, 125.68, 121.50, 120.06, 109.09, 105.77, 30.33. **^{31}P NMR** (162 MHz, $CDCl_3$): δ 27.6. **HRMS** (ESI) calcd for $C_{27}H_{21}ClNNaOP$ $(M+Na)^+$: 464.0941, found: 464.0937.

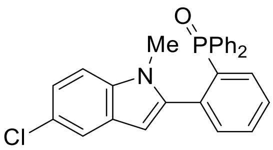

(2-(1-methyl-5-chloro-1*H*-indol-2-yl)phenyl)diphenylphosphine oxide (c7). Known comp-ound.[5] Yellow solid. 1.2 g, 55% yield. **^{1}H NMR** (400 MHz, $CDCl_3$) δ 8.01 – 7.90 (m,

1H), 7.71 – 7.61 (m, 2H), 7.60 – 7.52 (m, 2H), 7.49 – 7.44 (m, 3H), 7.42 – 7.31 (m, 6H), 7.17 (s, 2H), 7.08 (dd, J = 8.6, 2.0 Hz, 1H), 6.91 (d, J = 8.7 Hz, 1H), 6.11 (s, 1H), 3.01 (s, 3H).

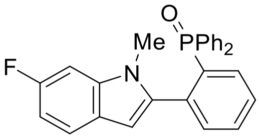

(2-(1-methyl-6-fluoro-1*H*-indol-2-yl)phenyl)diphenylphosphine oxide (c8). White solid. 0.85 g, 40% yield. **^{1}H NMR** (400 MHz, $CDCl_3$) δ 7.91 (dd, J = 13.1, 7.7 Hz, 1H), 7.62 (t, J = 7.7 Hz, 1H), 7.53 (t, J = 7.6 Hz, 1H), 7.47 (brs, 4H), 7.38 – 7.25 (m, 4H), 7.20 (brs, 4H), 6.78 (t, J = 9.2 Hz, 1H), 6.65 (d, J = 9.9 Hz, 1H), 6.26 (s, 1H), 2.98 (s, 3H). **^{13}C NMR** (101 MHz, $CDCl_3$) δ 159.75 (d, J_{CF} = 237.4 Hz), 137.35 (dd, J_{CP} = 4.5 Hz, J_{CF} = 4.0 Hz), 136.70 (d, J_{CP} = 12.2 Hz), 136.25 (d, J_{CP} = 8.5 Hz), 134.31 (d, J_{CP} = 10.5 Hz), 134.26 (d, J_{CP} = 102.4 Hz), 132.66 (d, J_{CP} = 9.4 Hz), 132.08 (d, J_{CF} = 10.0 Hz), 131.55 (brs), 131.54 (d, J_{CP} = 2.4 Hz), 131.21 (d, J_{CP} = 2.8 Hz), 128.36 (d, J_{CP} = 11.9 Hz), 127.81 (d, J_{CP} = 12.2 Hz), 123.63, 121.41 (d, J_{CF} = 10.1 Hz), 108.06 (d, J_{CF} = 24.5 Hz), 105.77, 95.37 (d, J_{CF} = 26.1 Hz), 30.34. **^{31}P NMR** (162 MHz, $CDCl_3$): δ 27.7. **HRMS** (ESI) calcd for $C_{27}H_{21}FNNaOP$ $(M+Na)^+$: 448.1237, found: 448.1235.

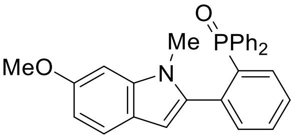

(2-(1-methyl-6-methoxy-1*H*-indol-2-yl)phenyl)diphenylphosphine oxide (c9). White solid. 1.3 g, 60% yield. **^{1}H NMR** (400 MHz, $CDCl_3$) δ 8.01 (ddd, J = 13.0, 7.7, 1.5 Hz, 1H), 7.65 – 7.60 (m, 1H), 7.58 – 7.52 (m, 1H), 7.44 (brs, 4H), 7.37 – 7.29 (m, 3H), 7.19 (brs, 4H), 6.86 (d, J = 4.2 Hz, 1H), 6.85 (d, J = 2.2 Hz, 1H), 6.79 (dd, J = 8.9, 2.2 Hz, 1H), 6.16 (s, 1H), 3.82 (s, 3H), 2.94 (s, 3H). **^{13}C NMR** (101 MHz, $CDCl_3$) δ 153.98, 137.39 (d, J_{CP} = 4.6 Hz), 136.60 (d, J_{CP} = 8.7 Hz), 134.29 (d, J_{CP} = 10.0 Hz), 134.09 (d, J_{CP} = 102.3 Hz), 132.60 (d, J_{CP} = 9.5 Hz), 132.08, 131.55 (brs), 131.48 (d, J_{CP} = 2.5 Hz), 131.13 (d, J_{CP} = 2.8 Hz), 128.23 (d, J_{CP} = 11.7 Hz), 127.77 (d, J_{CP} = 12.2 Hz), 127.41, 111.95, 109.85, 105.40, 102.18, 55.85, 30.17. **^{31}P NMR** (162 MHz, $CDCl_3$): δ 27.9. **HRMS** (ESI) calcd for $C_{28}H_{24}NNaO_2P$ $(M+Na)^+$: 460.1437, found: 460.1437.

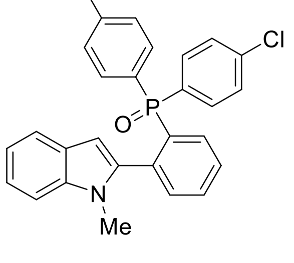

bis(4-chlorophenyl)(2-(1-methyl-1*H*-indol-2-yl)phenyl)phosphine oxide (c10). Yellow solid. 1.0 g, 43% yield. **^{1}H NMR** (400 MHz, $CDCl_3$) δ 7.95 (ddd, J = 13.3, 7.8, 1.4 Hz, 1H), 7.71 – 7.63 (m, 1H), 7.61 – 7.54 (m, 1H), 7.42 (dt, J = 7.9, 1.0 Hz, 1H), 7.38 (ddd, J = 7.5,

4.4, 1.4 Hz, 2H), 7.28 (brs, 12H), 7.18 (ddd, J = 8.2, 7.1, 1.2 Hz, 2H), 7.10 – 7.01 (m, 3H), 6.20 (s, 1H), 3.06 (s, 3H). **^{13}C NMR** (101 MHz, $CDCl_3$) δ 137.97 (d, J_{CP} = 3.4 Hz), 136.66, 136.55 (d, J_{CP} = 1.1 Hz), 136.48 (d, J_{CP} = 2.8 Hz), 134.17 (d, J_{CP} = 10.4 Hz), 134.40 (d, J_{CP} = 104.5 Hz), 132.78 (d, J_{CP} = 9.6 Hz), 132.79 (brs), 131.97 (d, J_{CP} = 2.5 Hz), 128.56 (d, J_{CP} = 12.0 Hz), 128.18 (d, J_{CP} = 12.7 Hz), 127.03, 122.20, 120.55, 119.81, 109.10, 105.62, 30.28. **^{31}P NMR** (162 MHz, $CDCl_3$): δ 26.6. **HRMS** (ESI) calcd for $C_{27}H_{20}Cl_2NNaOP$ $(M+Na)^+$: 498.0552, found: 498.0548.

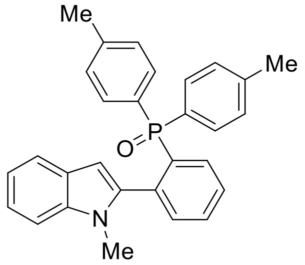

bis(4-methylphenyl)(2-(1-methyl-1*H*-indol-2-yl)phenyl)phosphine oxide (c11). White solid. 1.07 g, 49% yield. **^{1}H NMR** (400 MHz, $CDCl_3$) δ 8.02 (ddd, J = 13.0, 7.6, 1.5 Hz, 1H), 7.66 – 7.57 (tt, J = 7.5, 1.6 Hz, 1H), 7.55 (tt, J = 7.6, 1.8 Hz, 1H), 7.39 (d, J = 7.8 Hz, 1H), 7.32 (ddd, J = 7.4, 4.1, 1.4 Hz, 1H), 7.3 (brs, 4H), 7.17 – 7.08 (m, 1H), 7.06 – 7.00 (m, 1H), 7.01 (brs, 2H), 6.97 (d, J = 8.1 Hz, 1H), 6.87 (s, 2H), 6.19 (s, 1H), 3.00 (s, 3H), 2.26 (s, 6H). **^{13}C NMR** (101 MHz, $CDCl_3$) δ 141.58, 137.07 (d, J_{CP} = 4.4 Hz), 136.69, 136.42 (d, J_{CP} = 8.6 Hz), 134.58 (d, J_{CP} = 101.9 Hz), 134.27 (d, J_{CP} = 9.9 Hz), 132.61 (d, J_{CP} = 9.4 Hz), 131.52 (brs), 131.29 (d, J_{CP} = 2.4 Hz), 128.47 (d, J_{CP} = 12.6 Hz), 128.29 (d, J_{CP} = 11.5 Hz), 127.27, 121.56, 120.50, 119.34, 108.93, 105.27, 30.17, 21.45. **^{31}P NMR** (162 MHz, $CDCl_3$): δ 28.3. **HRMS** (ESI) calcd for $C_{29}H_{26}NNaOP$ $(M+Na)^+$: 458.1644, found: 458.1632.

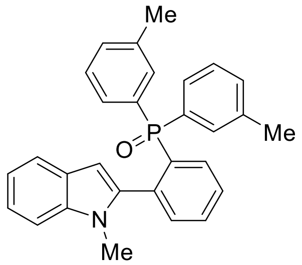

bis(3-methylphenyl)(2-(1-methyl-1*H*-indol-2-yl)phenyl)phosphine oxide (c12). White solid. 0.76 g, 35% yield. **^{1}H NMR** (400 MHz, $CDCl_3$) δ 7.99 (ddd, J = 13.0, 7.7, 1.5 Hz, 1H), 7.61 (tt, J = 7.5, 1.6 Hz, 1H), 7.55 (tt, J = 7.6, 1.8 Hz, 1H), 7.42 (d, J = 7.9 Hz, 1H), 7.37 (brs, 1H), 7.32 (dd, J = 7.6, 4.2 Hz, 1H), 7.22 (d, J = 10.2 Hz, 2H), 7.17 – 7.08 (m, 4H), 7.06 (brs, 2H), 7.01 (t, J = 7.4 Hz, 1H), 6.96 (d, J = 8.2 Hz, 1H), 6.26 (s, 1H), 2.99 (s, 3H), 2.10 (s, 3H), 2.02 (s, 3H). **^{13}C NMR** (101 MHz, $CDCl_3$) δ 137.93 (d, J_{CP} = 11.7 Hz), 136.95 (d, J_{CP} = 4.5 Hz), 136.63, 136.54, 134.39 (d, J_{CP} = 101.7 Hz), 134.20 (d, J_{CP} = 10.0 Hz), 132.67 (d, J_{CP} = 9.4 Hz), 132.20 (brs), 131.88 (d, J_{CP} = 2.7 Hz), 131.38 (d, J_{CP} = 2.4 Hz), 129.15 – 128.85, 128.23 (d, J_{CP} = 11.7 Hz), 127.60 (d, J_{CP} = 11.3 Hz), 127.28, 121.68, 120.70, 119.39, 109.06, 105.73, 30.16, 21.08. **^{31}P NMR** (162 MHz, $CDCl_3$): δ 27.8. **HRMS** (ESI) calcd for $C_{29}H_{26}NNaOP$ $(M+Na)^+$: 458.1644, found: 458.1640.

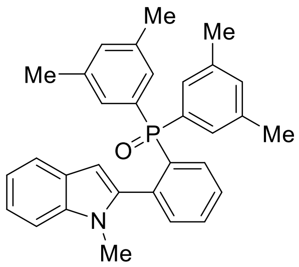

bis(3,5-dimethylphenyl)(2-(1-methyl-1*H*-indol-2-yl)phenyl)phosphine oxide (c13). White solid. 0.97 g, 42% yield. **^{1}H NMR** (400 MHz, $CDCl_3$) δ 8.03 (dd, *J* = 12.8, 7.5 Hz, 1H), 7.61 (t, *J* = 7.4 Hz, 1H), 7.56 (t, *J* = 7.7 Hz, 1H), 7.44 (d, *J* = 7.8 Hz, 1H), 7.30 (dd, *J* = 7.6, 4.2 Hz, 1H), 7.14 (t, *J* = 7.6 Hz, 1H), 7.07 (brs, 4H), 7.01 (t, *J* = 7.4 Hz, 1H), 6.97 (d, *J* = 8.2 Hz, 1H), 6.93 (s, 2H), 6.27 (s, 1H), 2.98 (s, 3H), 2.15 (s, 6H), 2.00 (s, 6H). **^{13}C NMR** (101 MHz, $CDCl_3$) δ 137.55 (d, J_{CP} = 12.7 Hz), 137.09 (d, J_{CP} = 4.0 Hz), 136.60, 136.48 (d, J_{CP} = 9.1 Hz), 134.51 (d, J_{CP} = 101.1 Hz), 134.23 (d, J_{CP} = 10.1 Hz), 132.78 (d, J_{CP} = 2.4 Hz), 132.62 (d, J_{CP} = 9.5 Hz), 131.23 (d, J_{CP} = 2.0 Hz), 129.50 – 128.90 (m), 128.24 (d, J_{CP} = 11.6 Hz), 127.33, 121.67, 120.72, 119.34, 108.98, 105.53, 30.09, 21.01. **^{31}P NMR** (162 MHz, $CDCl_3$): δ 28.3. **HRMS** (ESI) calcd for $C_{31}H_{31}NOP$ $(M+H)^+$: 464.2138, found: 464.2133.

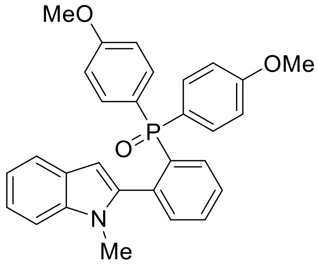

bis(4-methoxyphenyl)(2-(1-methyl-1*H*-indol-2-yl)phenyl)phosphine oxide (c14). White solid. 0.58 g, 25% yield. **^{1}H NMR** (400 MHz, $CDCl_3$) δ 8.11 (ddd, *J* = 12.8, 7.2, 1.9 Hz, 1H), 7.65 – 7.54 (m, 2H), 7.41 (d, *J* = 7.8 Hz, 1H), 7.39 (s, 2H), 7.31 (ddd, *J* = 7.0, 4.3, 1.6 Hz, 1H), 7.21 (s, 2H), 7.14 (t, *J* = 8.2 Hz, 1H), 7.04 (t, *J* = 7.4, 1H), 6.98 (d, *J* = 8.2 Hz, 1H), 6.77 (s, 2H), 6.51 (s, 2H), 6.15 (s, 1H), 3.72 (s, 6H), 2.97 (s, 3H). **^{13}C NMR** (101 MHz, $CDCl_3$) δ 161.84, 137.23 (d, J_{CP} = 4.4 Hz), 136.61, 136.18 (d, J_{CP} = 8.9 Hz), 134.69 (d, J_{CP} = 102.5 Hz), 134.25 (d, J_{CP} = 9.5 Hz), 133.47 (brs), 132.61 (d, J_{CP} = 9.5 Hz), 131.25 (d, J_{CP} = 2.4 Hz), 128.42 (d, J_{CP} = 11.4 Hz), 127.34, 121.43, 120.52, 119.33, 113.54 – 113.06 (m), 109.02, 104.88, 55.19, 30.11. **^{31}P NMR** (162 MHz, $CDCl_3$): δ 27.8. **HRMS** (ESI) calcd for $C_{29}H_{27}NO_3P$ $(M+H)^+$: 468.1723, found: 468.1717.

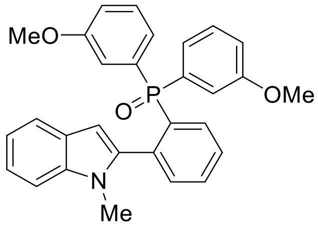

bis(3-methoxyphenyl)(2-(1-methyl-1*H*-indol-2-yl)phenyl)phosphine oxide (c15). White solid. 0.7 g, 30% yield. **^{1}H NMR** (400 MHz, $CDCl_3$) δ 7.84 (dd, *J* = 13.2, 7.8 Hz, 1H), 7.62 (t,

J = 7.5 Hz, 1H), 7.51 (t, J = 7.6 Hz, 1H), 7.43 (d, J = 7.8 Hz, 1H), 7.35 (dd, J = 7.4, 4.2 Hz, 1H), 7.18 – 7.11 (m, 3H), 7.09 (s, 1H), 7.07 – 6.99 (m, 5H), 6.85 (brs, 2H), 6.36 (s, 1H), 3.57 (s, 6H), 3.11 (s, 3H). **^{13}C NMR** (101 MHz, $CDCl_3$) δ 159.10 (d, J_{CP} = 14.9 Hz), 136.84 (d, J_{CP} = 8.5 Hz), 136.80 (d, J_{CP} = 4.6 Hz), 136.69, 134.40 (d, J_{CP} = 102.9 Hz), 134.05 (d, J_{CP} = 10.8 Hz), 132.7 (d, J_{CP} = 9.3 Hz), 131.46 (d, J_{CP} = 2.5 Hz), 129.22 – 128.77 (m), 128.13 (d, J_{CP} = 11.9 Hz), 124.3 – 123.0 (m), 127.19, 121.69, 120.64, 119.36, 117.57, 116.88 – 115.87 (m), 109.02, 106.04, 55.18, 30.29. **^{31}P NMR** (162 MHz, $CDCl_3$): δ 27.8. **HRMS** (ESI) calcd for $C_{29}H_{27}NO_3P$ $(M+H)^+$: 468.1723, found: 468.1717.

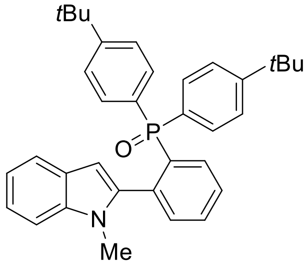

bis(4-*tert*butylphenyl)(2-(1-methyl-1*H*-indol-2-yl)phenyl)phosphine oxide (c16). White solid. 1.27 g, 49% yield. **^{1}H NMR** (400 MHz, $CDCl_3$) δ 8.07 (dd, J = 12.8, 7.5 Hz, 1H), 7.61 (t, J = 7.5 Hz, 1H), 7.55 (t, J = 7.6 Hz, 1H), 7.40 (s, 2H), 7.38 (d, J = 7.8 Hz, 1H), 7.30 (dd, J = 6.8, 4.1 Hz, 1H), 7.27 (s, 4H), 7.12 (t, J = 7.6 Hz, 1H), 7.08 (s, 2H), 7.02 (t, J = 7.4 Hz, 1H), 6.94 (d, J = 8.2 Hz, 1H), 6.22 (s, 1H), 2.91 (s, 3H), 1.24 (s, 18H). **^{13}C NMR** (101 MHz, $CDCl_3$) δ 154.47, 137.04 (d, J_{CP} = 4.5 Hz), 136.52, 136.38 (d, J_{CP} = 8.7 Hz), 134.69 (d, J_{CP} = 101.8 Hz), 134.26 (d, J_{CP} = 10.0 Hz), 132.59 (d, J_{CP} = 9.3 Hz), 131.76 (brs), 131.24 (d, J_{CP} = 2.6 Hz), 131.18 (brs), 128.19 (d, J_{CP} = 11.6 Hz), 127.26, 124.75 (d, J_{CP} = 12.5 Hz), 121.50, 120.56, 119.35, 109.17, 105.68, 34.79, 31.09, 30.01. **^{31}P NMR** (162 MHz, $CDCl_3$): δ 27.8. **HRMS** (ESI) calcd for $C_{29}H_{27}NO_3P$ $(M+H)^+$: 468.1723, found: 468.1717.

General Procedure for Copper-Bisoxazoline Catalyzed *ortho*-Arylation. NaOTf (3.4 mg, 0.02 mmol) was dissolved into MeOH (20 mL) and the NaOTf/MeOH solution was stored for use. To a dried Schlenk tube was charged with NaOTf/MeOH solution (0.5 mL) and solvent was removed under vacuum. CuCl (1 mg, 0.01 mmol), **L6** (6.9 mg, 0.015 mmol) and CH_2Cl_2 (2 mL) were added into the tube and the mixture was stirred at rt for 0.5 h under N_2. Substrate (0.1 mmol, 1.0 equiv.), diaryliodonium hexafluoroantimonate salt (0.15 mmol, 1.5 equiv.) and 2, 6-Di-*tert*-butylpyridine (DTBP, 33 μL, 1.5 equiv.) were added into the mixture under N_2. The reaction was heated to 60 °C and stirred for 48 h. After cooling to room temperature, the reaction mixture was filtrated, concentrated under vacuum. The crude product was purified via flash chromatography (PE/EA 5:1 to 2:1, v/v) to afford product. The absolute configuration of product was assigned to be ***R*** by XRD analysis of **b18**, as stated in part 1.6.

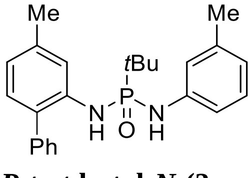

***P*-*tert*-butyl-*N*-(3-methylphenyl)-*N*'-[(3-methyl-6-phenyl)phenyl]phosphonic diamide (b**

1)**. White solid. 38 mg, 97% yield. **HPLC** conditions: Daicel CHIRALCEL® OD-H column with hexane/2-propanol = 95/05, flow = 1.0 mL/min, λ = 254 nm. Retention times: 14.0 min [(*R*)-enantiomer], 24.8 min [(*S*)-enantiomer]. $[\alpha]_D^{25}$: 96 (*c* 0.100, CH_2Cl_2, 90% ee). The absolute configuration was assigned by analogy with compound (*R*)-**b18**. **^{1}H NMR** (400 MHz, $CDCl_3$): δ 7.54 (s, 1H), 7.44 (t, *J* = 7.2 Hz, 2H), 7.40 – 7.35 (m, 1H), 7.34 – 7.32 (m, 2H), 7.13 – 7.05 (m, 2H), 6.93 -6.98 (m, 2H), 6.82 (d, *J* = 8.0 Hz, 1H), 6.77 (d, *J* = 7.5 Hz, 1H), 5.12 (d, *J* = 14.1 Hz, 1H), 4.61 (d, *J* = 9.1 Hz, 1H), 2.31 (s, 3H), 2.28 (s, 3H), 1.08 (d, *J* = 16.1 Hz, 9H). **^{13}C NMR** (101 MHz, $CDCl_3$): δ 141.33, 138.95, 138.88, 138.54, 137.26 (d, J_{CP} = 2.3 Hz), 129.77, 129.39, 129.12, 128.96, 128.00 (d, J_{CP} = 7.9 Hz), 127.72, 122.66, 122.37, 119.69 (d, J_{CP} = 5.3 Hz), 118.36 (d, J_{CP} = 2.4 Hz), 116.06 (d, J_{CP} = 4.9 Hz), 34.32 (d, J_{CP} = 114.4 Hz), 25.22, 21.48, 21.41. **^{31}P NMR** (162 MHz, $CDCl_3$): δ 27.7. **HRMS** (ESI) calcd for $C_{24}H_{29}N_2NaOP$ $(M+Na)^+$: 415.1910, found: 415.1897.

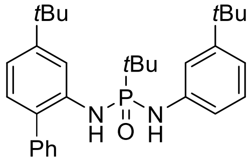

***P*-*tert*-butyl-*N*-(3-*tert*-butylphenyl)-*N*'-[(3-*tert*-butyl-6-phenyl)phenyl]phosphonic diami-de (b2)**. White solid. 42 mg, 88% yield. **HPLC** conditions: Daicel CHIRALCEL® OD-H column with hexane/2-propanol = 95/05, flow = 1.0 mL/min, λ = 254 nm. Retention times: 6.1 min [(*R*)-enantiomer], 15.0 min [(*S*)-enantiomer]. $[\alpha]_D^{25}$: 53 (*c* 0.100, CH_2Cl_2, 85% ee). The absolute configuration was assigned by analogy with compound (*R*)-**b18**. **^{1}H NMR** (400 MHz, $CDCl_3$): δ 7.76 (s, 1H), 7.44 (t, *J* = 7.3 Hz, 2H), 7.39 – 7.33 (m, 3H), 7.16 – 7.04 (m, 4H), 7.03 – 6.99 (dd, *J* = 8.0 and 1.3 Hz, 1H), 6.97 (d, *J* = 7.7 Hz, 1H), 5.16 (d, *J* = 13.9 Hz, 1H), 4.68 (d, *J* = 9.4 Hz, 1H), 1.24 (s, 9H), 1.23 (s, 9H), 1.12 (d, *J* = 16.0 Hz, 9H). **^{13}C NMR** (101 MHz, $CDCl_3$): δ 152.26, 152.00, 140.88, 138.52, 137.06 (d, J_{CP} = 2.4 Hz), 129.56, 129.35, 129.08, 128.77, 127.92 (d, J_{CP} = 8.0 Hz), 127.66, 118.96, 118.53, 116.32 (d, J_{CP} = 5.8 Hz), 116.14 (d, J_{CP} = 4.3 Hz), 115.33 (d, J_{CP} = 2.7 Hz), 34.68, 34.07 (d, J_{CP} = 108.8 Hz), 31.24, 31.20, 25.15. **^{31}P NMR** (162 MHz, $CDCl_3$): δ 28.0. **HRMS** (ESI) calcd for $C_{30}H_{41}N_2NaOP$ $(M+Na)^+$: 499.2849, found: 499.2838.

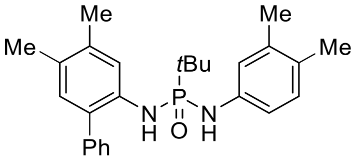

***P*-*tert*-butyl-*N*-(3, 5-dimethylphenyl)-*N*'-[(3, 5-dimethyl-6-phenyl)phenyl]phosphonic dia-mide (b3)**. White solid. 42 mg, 99% yield. **HPLC** conditions: Daicel CHIRALCEL® OD-H column with hexane/2-propanol = 90/10, flow = 1.0 mL/min, λ = 254 nm. Retention times: 12.0 min [(*R*)-enantiomer], 20.8 min [(*S*)-enantiomer]. $[\alpha]_D^{25}$: 52 (*c* 0.100, CH_2Cl_2, 85% ee). The absolute configuration was assigned by analogy with compound (*R*)-**b18**. **^{1}H NMR** (400 MHz, $CDCl_3$): δ 7.52 (s, 1H), 7.43 (t, *J* = 7.3 Hz, 2H), 7.37 (d, *J* = 7.3 Hz, 1H), 7.35 – 7.30 (m, 2H), 6.94 (m, 2H), 6.90 (m, 2H), 5.01 (d, *J* = 13.8 Hz, 1H), 4.54 (d, *J* = 9.1 Hz, 1H), 2.21 (s, 3H), 2.19 (s, 3H), 2.18 (s, 3H), 2.17 (s, 3H), 1.07 (d, *J* = 15.8 Hz, 9H). **^{13}C NMR** (101 MHz, $CDCl_3$): δ 138.99, 138.70, 137.24, 137.19, 134.99 (d, J_{CP} = 2.1 Hz), 130.99, 130.13,

129.90, 129.56, 129.36, 129.02, 128.43 (d, J_{CP} = 7.5 Hz), 127.56, 120.61 (d, J_{CP} = 5.0 Hz), 119.34 (d, J_{CP} = 2.1 Hz), 116.56 (d, J_{CP} = 4.6 Hz), 34.28 (d, J_{CP} = 114.6 Hz), 25.31, 19.87, 19.78, 18.90. ^{31}**P NMR** (162 MHz, $CDCl_3$): δ 27.7. **HRMS** (ESI) calcd for $C_{26}H_{33}N_2NaOP$ $(M+Na)^+$: 443.2223, found: 443.2212.

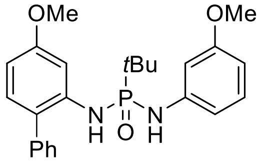

***P-tert*-butyl-*N*-(3-methoxyphenyl)-*N*'-[(3-methoxy-6-phenyl)phenyl]phosphonic diamide (b4)**. White solid. 36 mg, 84% yield. **HPLC** conditions: Daicel CHIRALCEL® OD-H column with hexane/2-propanol = 90/10, flow = 1.0 mL/min, λ = 254 nm. Retention times: 12.7 min [(*R*)-enantiomer], 16.0 min [(*S*)-enantiomer]. $[\alpha]_D^{25}$: - 14 (*c* 0.050, CH_2Cl_2, 86% ee). The absolute configuration was assigned by analogy with compound (*R*)-**b18**. ^{1}**H NMR** (400 MHz, $CDCl_3$): δ 7.44 (t, *J* = 7.4 Hz, 2H), 7.38 (d, *J* = 7.3 Hz, 1H), 7.35 – 7.30 (m, 3H), 7.10 (t, *J* = 8.5 Hz, 2H), 6.79 (s, 1H), 6.73 (d, *J* = 7.9 Hz, 1H), 6.56 (dd, *J* = 8.4, 2.4 Hz, 1H), 6.51 (dd, *J* = 8.2, 2.3 Hz, 1H), 5.20 (d, *J* = 14.2 Hz, 1H), 4.76 (d, *J* = 9.2 Hz, 1H), 3.74 (s, 3H), 3.72 (s, 3H), 1.10 (d, *J* = 16.1 Hz, 9H). ^{13}**C NMR** (101 MHz, $CDCl_3$): δ 160.37, 159.93, 142.55, 138.41 (d, J_{CP} = 2.5 Hz), 138.27, 130.70, 129.86, 129.50, 129.14, 127.60, 123.36 (d, J_{CP} = 7.9 Hz), 111.51 (d, J_{CP} = 5.3 Hz), 107.98, 107.58, 104.89 (d, J_{CP} = 5.1 Hz), 103.09 (d, J_{CP} = 2.6 Hz), 55.23, 55.15, 34.25 (d, J_{CP} = 114.2 Hz), 25.09. ^{31}**P NMR** (162 MHz, $CDCl_3$): δ 28.1. **HRMS** (ESI) calcd for $C_{24}H_{29}N_2NaO_3P$ $(M+Na)^+$: 447.1808, found: 447.1798.

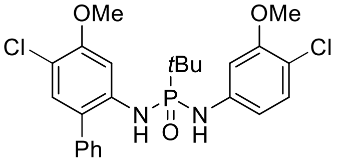

***P-tert*-butyl-*N*-(3-methoxy-4-chlorophenyl)-*N*'-[(3-methoxy-4-chloro-6-phenyl)phenyl]-phosphonic diamide (b5)**. White solid. 48 mg, 97% yield. **HPLC** conditions: Daicel CHIRALCEL® OD-H column with hexane/2-propanol = 90/10, flow = 1.0 mL/min, λ = 254 nm. Retention times: 10.0 min [(*R*)-enantiomer], 12.1 min [(*S*)-enantiomer]. $[\alpha]_D^{25}$: 56 (*c* 0.100, CH_2Cl_2, 92% ee). The absolute configuration was assigned by analogy with compound (*R*)-**b18**. ^{1}**H NMR** (400 MHz, $CDCl_3$): δ 7.49 – 7.35 (m, 4H), 7.30 – 7.27 (m, 2H), 7.19 (s, 1H), 7.16 (d, *J* = 8.5 Hz, 1H), 6.98 (d, *J* = 2.4 Hz, 1H), 6.60 (dd, *J* = 8.5, 2.3 Hz, 1H), 5.15 (d, *J* = 13.8 Hz, 1H), 4.74 (d, *J* = 9.7 Hz, 1H), 3.81 (s, 3H), 3.78 (s, 3H), 1.12 (d, *J* = 16.2 Hz, 9H). ^{13}**C NMR** (101 MHz, $CDCl_3$): δ 155.37, 154.83, 141.13, 137.11 (d, J_{CP} = 1.3 Hz), 136.89, 130.95, 130.23, 129.34, 129.22, 128.14, 123.84 (d, J_{CP} = 7.7 Hz), 115.51, 114.98, 111.59 (d, J_{CP} = 5.4 Hz), 103.58 (d, J_{CP} = 4.2 Hz), 102.29, 56.07, 56.02, 34.21 (d, J_{CP} = 114.2 Hz), 24.90. ^{31}**P NMR** (162 MHz, $CDCl_3$): δ 29.0. **HRMS** (ESI) calcd for $C_{24}H_{29}N_2NaOP$ $(M+Na)^+$: 330.1042, found: 330.1038.

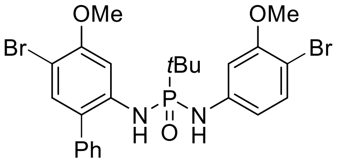

***P-tert*-butyl-*N*-(3-methoxy-4-bromophenyl)-*N*'-[(3-methoxy-4-bromo-6-phenyl)phenyl] phosphonic diamide (b6)**. White solid. 57 mg, 98% yield. **HPLC** conditions: Daicel CHIRALCEL® OD-H column with hexane/2-propanol = 90/10, flow = 1.0 mL/min, λ = 254 nm. Retention times: 10.5 min [(*R*)-enantiomer], 12.7 min [(*S*)-enantiomer]. $[\alpha]_D{}^{25}$: 77 (*c* 0.100, CH_2Cl_2, 91% ee). The absolute configuration was assigned by analogy with compound (*R*)-**b18**. **^{1}H NMR** (400 MHz, $CDCl_3$): δ 7.45 (t, *J* = 7.3 Hz, 2H), 7.40 (m, 2H), 7.35 (s, 1H), 7.32 (d, *J* = 8.3 Hz, 1H), 7.30 – 7.25 (m, 2H), 6.96 (s, 1H), 6.56 (d, *J* = 8.1 Hz, 1H), 5.16 (d, *J* = 14.0 Hz, 1H), 4.76 (d, *J* = 10.1 Hz, 1H), 3.80 (s, 3H), 3.77 (s, 3H), 1.12 (d, *J* = 16.2 Hz, 9H). **^{13}C NMR** (101 MHz, $CDCl_3$): δ 156.31, 155.79, 141.90, 137.87 (d, J_{CP} = 2.4 Hz), 136.80, 133.86, 133.26, 129.34, 129.23, 128.15, 124.41 (d, J_{CP} = 7.8 Hz), 112.15 (d, J_{CP} = 5.8 Hz), 103.91, 103.42 (d, J_{CP} = 2.1 Hz), 103.40 (d, J_{CP} = 2.6 Hz), 102.10 (d, J_{CP} = 2.5 Hz), 56.16, 56.11, 34.22 (d, J_{CP} = 114.1 Hz), 24.90. **^{31}P NMR** (162 MHz, $CDCl_3$): δ 28.9. **HRMS** (ESI) calcd for $C_{24}H_{27}Cl_2N_2NaO_3P$ $(M+Na)^+$: 515.1029, found: 515.1030.

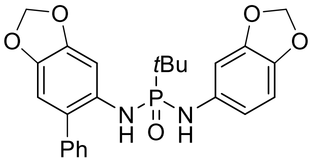

***P-tert*-butyl-*N*-(3, 4-methylenedioxyphenyl)-*N*'-[(3, 4-methylenedioxy-6-phenyl) phenyl] phosphonic diamide (b7)**. White solid. 30 mg, 67% yield. **HPLC** conditions: Daicel CHIRALCEL® OD-H column with hexane/2-propanol = 80/20, flow = 1.0 mL/min, λ = 254 nm. Retention times: 14.7 min [(*S*)-enantiomer], 17.9 min [(*R*)-enantiomer]. $[\alpha]_D{}^{25}$: 83 (*c* 0.100, CH_2Cl_2, 70% ee). The absolute configuration was assigned by analogy with compound (*R*)-**b18**. **^{1}H NMR** (400 MHz, $CDCl_3$):δ 7.45 (t, *J* = 7.3 Hz, 2H), 7.38 (d, *J* = 7.0 Hz, 1H), 7.35 (s, 1H), 7.32 – 7.28 (m, 2H), 6.80 (d, *J* = 2.2 Hz, 1H), 6.68 (s, 1H), 6.64 (d, *J* = 8.2 Hz, 1H), 6.49 (dd, *J* = 8.3, 2.2 Hz, 1H), 5.92 (s, 2H), 5.90 (s, 2H), 4.95 (d, *J* = 13.6 Hz, 1H), 4.48 (d, *J* = 9.5 Hz, 1H), 1.05 (d, *J* = 15.9 Hz, 9H). **^{13}C NMR** (101 MHz, $CDCl_3$): δ 148.01, 147.61, 142.93, 142.49, 138.54, 135.31, 131.59 (d, J_{CP} = 2.5 Hz), 129.53, 129.18, 127.76, 123.76 (d, J_{CP} = 7.9 Hz), 112.26 (d, J_{CP} = 5.6 Hz), 109.61, 108.23, 102.20 (d, J_{CP} = 4.2 Hz), 101.09, 101.01, 100.65 (d, J_{CP} = 2.4 Hz), 34.14 (d, J_{CP} = 113.6 Hz), 25.15. **^{31}P NMR** (162 MHz, $CDCl_3$): δ 28.7. **HRMS** (ESI) calcd for $C_{24}H_{25}N_2NaO_5P$ $(M+Na)^+$: 475.1393, found: 475.1386.

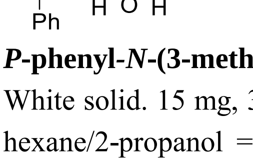

***P*-phenyl-*N*-(3-methylphenyl)-*N*'-[(3-methyl-6-phenyl)phenyl]phosphonic diamide (b8)**. White solid. 15 mg, 36% yield. **HPLC** conditions: Daicel CHIRALPAK® AD-H column with hexane/2-propanol = 80/20, flow = 1.0 mL/min, λ = 254 nm. Retention times: 10.3 min

[(*R*)-enantiomer], 20.1 min [(*S*)-enantiomer]. $[\alpha]_D^{25}$: 15 (*c* 0.050, CH_2Cl_2, 54% ee). The absolute configuration was assigned by analogy with compound (*R*)-**b18**. **^{1}H NMR** (400 MHz, $CDCl_3$): δ 7.82 (d, *J* = 7.6 Hz, 1H), 7.79 (d, *J* = 7.6 Hz, 1H), 7.53 (t, *J* = 7.5 Hz, 1H), 7.47 – 7.40 (m, 2H), 7.38 (s, 1H), 7.35 – 7.27 (m, 5H), 7.10 – 7.02 (m, 2H), 6.86 (s, 1H), 6.83 (d, *J* = 7.6 Hz, 1H), 6.78 (d, *J* = 7.8 Hz, 2H), 5.33 (d, *J* = 10.0 Hz, 1H), 5.00 (d, *J* = 9.9 Hz, 1H), 2.29 (s, 3H), 2.25 (s, 3H). **^{13}C NMR** (150 MHz, $CDCl_3$): δ 139.64, 139.20, 138.74, 138.34, 136.93, 132.43 (d, J_{CP} = 3.0 Hz), 131.51 (d, J_{CP} = 158.4 Hz), 131.41 (d, J_{CP} = 10.3 Hz), 130.18, 129.24, 129.11, 129.00, 128.77 (d, J_{CP} = 14.1 Hz), 128.42 (d, J_{CP} = 8.9 Hz), 127.55, 123.12, 122.88, 119.53 (d, J_{CP} = 6.2 Hz), 118.77 (d, J_{CP} = 2.5 Hz), 115.94 (d, J_{CP} = 6.1 Hz), 21.42, 21.40. **^{31}P NMR** (162 MHz, $CDCl_3$): δ 10.6. **HRMS** (ESI) calcd for $C_{26}H_{25}N_2NaOP$ $(M+Na)^+$: 435.1597, found: 435.1586.

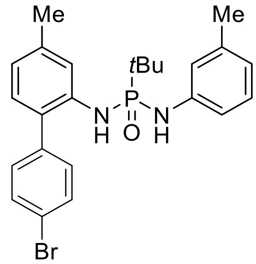

***P*-*tert*-butyl-*N*-(3-methylphenyl)-*N*'-{[3-methyl-6-(4-bromophenyl)phenyl]}phosphonic diamide (b9)**. White solid. 47 mg, 99% yield. **HPLC** conditions: Daicel CHIRALCEL® OD-H column with hexane/2-propanol = 90/10, flow = 1.0 mL/min, λ = 254 nm. Retention times: 11.6 min [(*R*)-enantiomer], 14.1 min [(*S*)-enantiomer]. $[\alpha]_D^{25}$: 154 (*c* 0.100, CH_2Cl_2, 89% ee). The absolute configuration was assigned by analogy with compound (*R*)-**b18**. **^{1}H NMR** (400 MHz, $CDCl_3$): δ 7.57 (s, 1H), 7.54 (d, *J* = 8.3 Hz, 2H), 7.18 (d, *J* = 8.3 Hz, 2H), 7.11 (t, *J* = 7.7 Hz, 1H), 7.01 (d, *J* = 7.8 Hz, 1H), 6.98 (d, *J* = 9.0 Hz, 1H), 6.95 (s, 1H), 6.80 (t, *J* = 6.9 Hz, 2H), 4.95 (d, *J* = 13.2 Hz, 1H), 4.63 (d, *J* = 9.9 Hz, 1H), 2.31 (s, 3H), 2.29 (s, 3H), 1.14 (d, *J* = 16.1 Hz, 9H). **^{13}C NMR** (101 MHz, $CDCl_3$): δ 141.01, 139.29, 139.03, 137.49, 137.33 (d, J_{CP} = 1.9 Hz), 132.22, 131.07, 129.80, 129.02, 126.62 (d, J_{CP} = 8.0 Hz), 122.89, 122.49, 121.80, 119.85 (d, J_{CP} = 5.3 Hz), 118.58 (d, J_{CP} = 2.3 Hz), 116.23 (d, J_{CP} = 4.9 Hz), 34.33 (d, J_{CP} = 114.6 Hz), 25.30, 21.47, 21.46. **^{31}P NMR** (162 MHz, $CDCl_3$): δ 27.9. **HRMS** (ESI) calcd for $C_{24}H_{28}BrN_2NaOP$ $(M+Na)^+$: 493.1015, found: 493.1005.

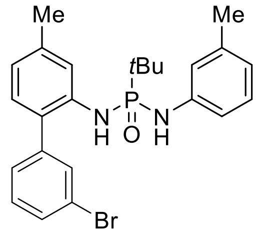

***P*-*tert*-butyl-*N*-(3-methylphenyl)-*N*'-{[3-methyl-6-(3-bromophenyl)phenyl]}phosphonic diamide (b10)**. White solid. 47 mg, 99% yield. **HPLC** conditions: Daicel CHIRALCEL® OD-H column with hexane/2-propanol = 90/10, flow = 1.0 mL/min, λ = 254 nm. Retention times: 11.7 min [(*R*)-enantiomer], 14.1 min [(*S*)-enantiomer]. $[\alpha]_D^{25}$: 31 (*c* 0.100, CH_2Cl_2, 91% ee). The absolute configuration was assigned by analogy with compound (*R*)-**b18**. **^{1}H NMR**

(400 MHz, $CDCl_3$): δ 7.56 (s, 1H), 7.54 - 7.48 (m, 2H), 7.30 (t, *J* = 7.6 Hz, 1H), 7.27 - 7.23 (m, 1H), 7.10 (t, *J* = 8.1 Hz, 1H), 7.04 (d, *J* = 7.7 Hz, 1H), 6.99 – 6.94 (m, 2H), 6.82 (d, *J* = 7.8 Hz, 1H), 6.78 (d, *J* = 7.5 Hz, 1H), 5.02 (d, *J* = 13.7 Hz, 1H), 4.64 (d, *J* = 9.4 Hz, 1H), 2.30 (s, 3H), 2.28 (s, 3H), 1.14 (d, *J* = 16.0 Hz, 9H). **^{13}C NMR** (101 MHz, $CDCl_3$): δ 141.12, 140.71, 139.51, 139.04, 137.17 (d, J_{CP} = 2.4 Hz), 132.44, 130.73, 130.61, 129.74, 129.02, 127.99, 126.42 (d, J_{CP} = 8.0 Hz), 123.13, 122.83, 122.58, 119.71 (d, J_{CP} = 5.3 Hz), 118.64 (d, J_{CP} = 2.5 Hz), 116.09 (d, J_{CP} = 4.9 Hz), 34.37 (d, *J* = 114.5 Hz), 25.27, 21.50, 21.45. **^{31}P NMR** (162 MHz, $CDCl_3$): δ 27.8. **HRMS** (ESI) calcd for $C_{24}H_{28}BrN_2NaOP$ $(M+Na)^+$: 493.1015, found: 493.1000.

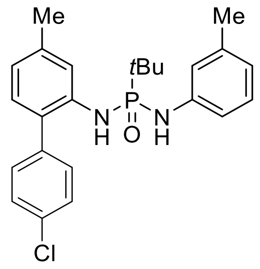

***P-tert*-butyl-*N*-(3-methylphenyl)-*N*'-{[3-methyl-6-(4-chlorophenyl)phenyl]}phosphonic diamide (b11)**. White solid. 42 mg, 99% yield. **HPLC** conditions: Daicel CHIRALCEL® OD-H column with hexane/2-propanol = 90/10, flow = 1.0 mL/min, λ = 254 nm. Retention times: 11.1 min [(*R*)-enantiomer], 13.9 min [(*S*)-enantiomer]. $[\alpha]_D^{25}$: 54 (*c* 0.100, CH_2Cl_2, 89% ee). The absolute configuration was assigned by analogy with compound (*R*)-**b18**. **^{1}H NMR** (400 MHz, $CDCl_3$): δ 7.56 (s, 1H), 7.39 (dd, *J* = 8.2, 1.4 Hz, 2H), 7.24 (dd, *J* = 8.3, 1.4 Hz, 2H), 7.11 (t, *J* = 7.6 Hz, 1H), 7.03 – 6.98 (m, 1H), 6.96 (s, 2H), 6.80 (t, *J* = 7.5 Hz, 2H), 4.95 (d, *J* = 13.4 Hz, 1H), 4.62 (d, *J* = 10.0 Hz, 1H), 2.31 (s, 3H), 2.29 (s, 3H), 1.13 (d, *J* = 16.1 Hz, 9H). **^{13}C NMR** (101 MHz, $CDCl_3$): δ 141.01, 139.26, 139.04, 137.38 (d, J_{CP} = 2.1 Hz), 137.01, 133.68, 130.75, 129.84, 129.26, 129.02, 126.64 (d, J_{CP} = 7.9 Hz), 122.89, 122.48, 119.83 (d, J_{CP} = 5.3 Hz), 118.57 (d, J_{CP} = 2.4 Hz), 116.22 (d, J_{CP} = 4.9 Hz), 34.32 (d, J_{CP} = 114.6 Hz), 25.29, 21.45, 21.45. **^{31}P NMR** (162 MHz, $CDCl_3$): δ 27.9. **HRMS** (ESI) calcd for $C_{24}H_{28}ClN_2NaOP$ $(M+Na)^+$: 449.1520, found: 449.1513.

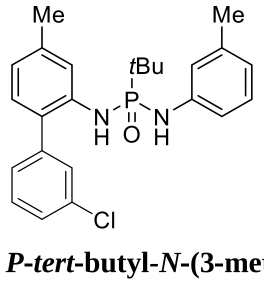

***P-tert*-butyl-*N*-(3-methylphenyl)-*N*'-{[3-methyl-6-(3-chlorophenyl)phenyl]}phosphonic diamide (b12)**. White solid. 42 mg, 99% yield. **HPLC** conditions: Daicel CHIRALCEL® OD-H column with hexane/2-propanol = 90/10, flow = 1.0 mL/min, λ = 254 nm. Retention times: 10.9 min [(*R*)-enantiomer], 13.1 min [(*S*)-enantiomer]. $[\alpha]_D^{25}$: 17 (*c* 0.100, CH_2Cl_2, 90% ee). The absolute configuration was assigned by analogy with compound (*R*)-**b18**. **^{1}H NMR** (400 MHz, $CDCl_3$): δ 7.56 (s, 1H), 7.38 – 7.32 (m, 3H), 7.23 – 7.17 (m, 1H), 7.10 (t, *J* = 7.9 Hz, 1H), 7.04 (d, *J* = 7.7 Hz, 1H), 6.99 – 6.93 (m, 2H), 6.82 (d, *J* = 7.7 Hz, 1H), 6.78 (d, *J* =

7.4 Hz, 1H), 5.02 (d, J = 13.7 Hz, 1H), 4.63 (d, J = 9.4 Hz, 1H), 2.31 (s, 3H), 2.28 (s, 3H), 1.13 (d, J = 16.1 Hz, 9H). **^{13}C NMR** (101 MHz, $CDCl_3$): δ 141.09, 140.43, 139.49, 139.05, 137.18 (d, J_{CP} = 1.4 Hz), 134.99, 130.34, 129.73, 129.57, 129.02, 127.81, 127.50, 126.52 (d, J_{CP} = 7.7 Hz), 122.85, 122.58, 119.70 (d, J_{CP} = 5.4 Hz), 118.63 (d, J_{CP} = 1.7 Hz), 116.09 (d, J_{CP} = 4.9 Hz), 34.36 (d, J_{CP} = 114.4 Hz), 25.26, 21.48, 21.46. **^{31}P NMR** (162 MHz, $CDCl_3$): δ 27.8. **HRMS** (ESI) calcd for $C_{24}H_{28}ClN_2NaOP$ $(M+Na)^+$: 449.1520, found: 449.1510.

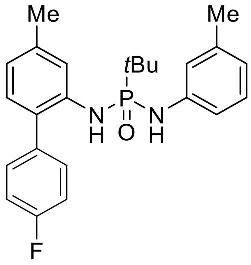

***P-tert*-butyl-*N*-(3-methylphenyl)-*N*'-{[3-methyl-6-(4-fluorophenyl)phenyl]}phosphonic diamide (b13)**. White solid. 41 mg, 99% yield. **HPLC** conditions: Daicel CHIRALCEL® OD-H column with hexane/2-propanol = 90/10, flow = 1.0 mL/min, λ = 254 nm. Retention times: 10.9 min [(*R*)-enantiomer], 14.7 min [(*S*)-enantiomer]. $[\alpha]_D{}^{25}$: 57 (*c* 0.100, CH_2Cl_2, 86% ee). The absolute configuration was assigned by analogy with compound (*R*)-**b18**. **^{1}H NMR** (400 MHz, $CDCl_3$): δ 7.55 (s, 1H), 7.31 – 7.27 (m, 2H), 7.16 – 7.06 (m, 3H), 7.02 (d, J = 7.7 Hz, 1H), 7.00 – 6.93 (m, 2H), 6.80 (t, J = 8.2 Hz, 2H), 4.96 (d, J = 13.5 Hz, 1H), 4.66 (d, J = 9.7 Hz, 1H), 2.30 (s, 3H), 2.28 (s, 3H), 1.11 (d, J = 16.1 Hz, 9H). **^{13}C NMR** (101 MHz, $CDCl_3$): δ 162.23 (d, J_{CF} = 247.6 Hz), 141.19, 139.08, 138.98, 137.48 (d, J_{CF} = 1.3 Hz), 134.46 (d, J_{CP} = 2.9 Hz), 131.14 (d, J_{CF} = 8.0 Hz), 129.91, 128.98, 126.86 (d, J_{CP} = 8.0 Hz), 122.78, 122.38, 119.82 (d, J_{CP} = 5.4 Hz), 118.43 (d, J_{CP} = 1.9 Hz), 116.17 (d, J_{CP} = 4.3 Hz), 116.05 (d, J_{CF} = 21.1 Hz), 34.32 (d, J_{CP} = 114.4 Hz), 25.26, 21.46, 21.41. **^{31}P NMR** (162 MHz, $CDCl_3$): δ 27.8. **HRMS** (ESI) calcd for $C_{24}H_{28}FN_2NaOP$ $(M+Na)^+$: 433.1815, found: 433.1803.

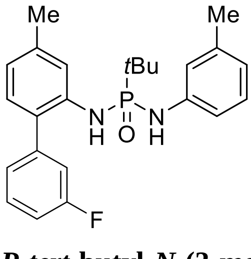

***P-tert*-butyl-*N*-(3-methylphenyl)-*N*'-{[3-methyl-6-(3-fluorophenyl)phenyl]}phosphonic diamide (b14)**. White solid. 41 mg, 99% yield. **HPLC** conditions: Daicel CHIRALCEL® OD-H column with hexane/2-propanol = 90/10, flow = 1.0 mL/min, λ = 254 nm. Retention times: 10.4 min [(*R*)-enantiomer], 13.1 min [(*S*)-enantiomer]. $[\alpha]_D{}^{25}$: 142 (*c* 0.100, CH_2Cl_2, 90% ee). The absolute configuration was assigned by analogy with compound (*R*)-**b18**. **^{1}H NMR** (400 MHz, $CDCl_3$): δ 7.56 (s, 1H), 7.45 – 7.34 (m, 1H), 7.13 – 7.02 (m, 5H), 6.99 – 6.94 (m, 2H), 6.82 (d, J = 7.6 Hz, 1H), 6.78 (d, J = 7.5 Hz, 1H), 5.06 (d, J = 13.7 Hz, 1H), 4.66 (d, J = 9.5 Hz, 1H), 2.30 (s, 3H), 2.28 (s, 3H), 1.12 (d, J = 16.1 Hz, 9H). **^{13}C NMR** (101 MHz, $CDCl_3$): δ 163.10 (d, J_{CF} = 248.0 Hz), 141.09, 140.85 (d, J_{CF} = 7.7 Hz), 139.41, 139.04

137.20 (d, J_{CP} = 2.2 Hz), 130.64 (d, J_{CF} = 8.4 Hz), 129.70, 129.01, 126.67 (dd, J_{CP} = 7.6, J_{CF} = 1.6 Hz), 124.98 (d, J_{CF} = 3.0 Hz), 122.84, 122.50, 119.71 (d, J_{CP} = 5.2 Hz), 118.56 (d, J_{CP} = 2.4 Hz), 116.49 (d, J_{CF} = 21.2 Hz), 116.10 (d, J_{CP} = 4.8 Hz), 114.64 (d, J_{CF} = 21.0 Hz), 34.33 (d, J_{CP} = 114.4 Hz), 25.24, 21.46, 21.45. **^{31}P NMR** (162 MHz, $CDCl_3$): δ 27.8. **HRMS** (ESI) calcd for $C_{24}H_{28}FN_2NaOP$ $(M+Na)^+$: 433.1815, found: 433.1804.

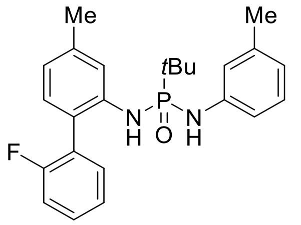

***P-tert*-butyl-*N*-(3-methylphenyl)-*N*'-{[3-methyl-6-(2-fluorophenyl)phenyl]}phosphonic diamide (b15)**. White solid. 20 mg, 49% yield. **HPLC** conditions: Daicel CHIRALPAK® AD-H column with hexane/2-propanol = 90/10, flow = 1.0 mL/min, λ = 254 nm. Retention times: 10.8 min [(*R*)-enantiomer], 28.4 min [(*S*)-enantiomer]. $[\alpha]_D$ ^{25}: 16 (*c* 0.100, CH_2Cl_2, 92% ee). The absolute configuration was assigned by analogy with compound (*R*)-**b18**. **^{1}H NMR** (400 MHz, $CDCl_3$): δ 7.60 (s, 1H), 7.45 – 7.34 (m, 1H), 7.30 (brs, 1H), 7.19 (t, *J* = 9.1 Hz, 1H), 7.12 – 7.03 (m, 2H), 6.96 (brs, 2H), 6.87 (d, *J* = 7.7 Hz, 1H), 6.75 (d, *J* = 7.4 Hz, 1H), 4.75 (d, *J* = 15.4 Hz, 1H), 4.67 (brs, 1H), 2.32 (s, 3H), 2.27 (s, 3H), 1.09 (d, *J* = 16.0 Hz, 9H). **^{13}C NMR** (101 MHz, $CDCl_3$): 159.72 (d, J_{CF} = 245.6 Hz), 139.70, 138.97, 137.71 (d, J_{CP} = 1.8 Hz), 132.29 (d, J_{CF} = 2.6 Hz), 130.31, 130.05 (d, J_{CP} = 8.1 Hz), 128.95, 125.78 (d, J_{CF} = 17.5 Hz), 124.98 (d, J_{CP} = 2.6 Hz), 123.00, 122.61, 119.51 (d, J_{CP} = 5.2 Hz), 116.02 (d, J_{CF} = 21.2 Hz), 115.89 (d, J_{CP} = 3.0 Hz), 34.28 (d, J_{CP} = 114.7 Hz), 25.18, 21.48, 21.45. **^{31}P NMR** (243 MHz, $CDCl_3$): δ 27.1 (brs). **HRMS** (ESI) calcd for $C_{24}H_{28}FN_2NaOP$ $(M+Na)^+$: 433.1815, found: 433.1800.

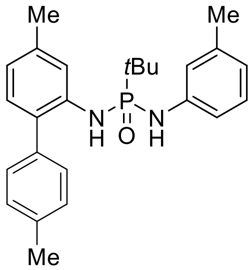

***P-tert*-butyl-*N*-(3-methylphenyl)-*N*'-{[3-methyl-6-(4-methylphenyl)phenyl]}phosphonic diamide (b16)**. White solid. 30 mg, 74% yield. **HPLC** conditions: Daicel CHIRALCEL® OD-H column with hexane/2-propanol = 90/10, flow = 1.0 mL/min, λ = 254 nm. Retention times: 6.8 min [(*R*)-enantiomer], 10.7 min [(*S*)-enantiomer]. $[\alpha]_D$ ^{25}: 25 (*c* 0.100, CH_2Cl_2, 84% ee). The absolute configuration was assigned by analogy with compound (*R*)-**b18**. **^{1}H NMR** (400 MHz, $CDCl_3$): δ 7.53 (s, 1H), 7.26 – 7.18 (m, 4H), 7.08 (t, *J* = 7.7 Hz, 1H), 7.04 (d, *J* = 7.7 Hz, 1H), 6.96 (d, *J* = 7.6 Hz, 2H), 6.79 (d, *J* = 7.8 Hz, 1H), 6.76 (d, *J* = 7.5 Hz, 1H), 5.16 (d, *J* = 14.2 Hz, 1H), 4.60 (d, *J* = 9.1 Hz, 1H), 2.39 (s, 3H), 2.29 (s, 3H), 2.27 (s, 3H), 1.09 (d, *J* = 16.0 Hz, 9H). **^{13}C NMR** (101 MHz, $CDCl_3$): δ 141.30, 138.96, 138.62, 137.43, 137.29 (d, J_{CP} = 2.3 Hz), 135.47, 129.89, 129.78, 129.20, 128.97, 127.92 (d, J_{CP} = 8.1 Hz), 122.69,

122.34, 119.70 (d, J_{CP} = 5.2 Hz), 118.29 (d, J_{CP} = 2.6 Hz), 116.11 (d, J_{CP} = 4.8 Hz), 34.32 (d, J_{CP} = 114.4 Hz), 25.25, 21.47, 21.45, 21.20. **^{31}P NMR** (162 MHz, $CDCl_3$): δ 27.7. **HRMS** (ESI) calcd for $C_{25}H_{31}N_2NaOP$ $(M+Na)^+$: 429.2066, found: 429.2055.

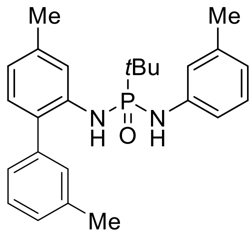

***P-tert*-butyl-*N*-(3-methylphenyl)-*N*'-{[3-methyl-6-(3-methylphenyl)phenyl]}phosphonic diamide (b17)**. White solid. 39 mg, 97% yield. **HPLC** conditions: Daicel CHIRALCEL® OD-H column with hexane/2-propanol = 90/10, flow = 1.0 mL/min, λ = 254 nm. Retention times: 7.1 min [(*R*)-enantiomer], 11.0 min [(*S*)-enantiomer]. $[\alpha]_D^{25}$: - 58 (*c* 0.100, CH_2Cl_2, 88% ee). The absolute configuration was assigned by analogy with compound (*R*)-**b18**. **^{1}H NMR** (400 MHz, $CDCl_3$): δ 7.53 (s, 1H), 7.33 (t, *J* = 7.5 Hz, 1H), 7.21 – 7.1 (m, 3H), 7.09 – 7.05 (m, 2H), 6.98 – 6.93 (m, 2H), 6.81 (d, *J* = 7.7 Hz, 1H), 6.77 (d, *J* = 7.5 Hz, 1H), 5.20 (d, *J* = 14.4 Hz, 1H), 4.62 (d, *J* = 9.1 Hz, 1H), 2.36 (s, 3H), 2.30 (s, 3H), 2.28 (s, 3H), 1.10 (d, *J* = 16.0 Hz, 9H). **^{13}C NMR** (101 MHz, $CDCl_3$): δ 141.26, 139.00, 138.82, 138.76, 138.41, 137.17 (d, J_{CP} = 2.2 Hz), 130.07, 129.72, 129.02, 128.99, 128.41, 128.06 (d, J_{CP} = 8.0 Hz), 126.33, 122.69, 122.36, 119.56 (d, J_{CP} = 5.3 Hz), 118.28 (d, J_{CP} = 2.6 Hz), 115.98 (d, J_{CP} = 4.9 Hz), 34.31 (d, J_{CP} = 114.7 Hz), 25.21, 21.48, 21.46, 21.37. **^{31}P NMR** (162 MHz, $CDCl_3$): δ 27.6. **HRMS** (ESI) calcd for $C_{25}H_{31}N_2NaOP$ $(M+Na)^+$: 429.2066, found: 429.2055.

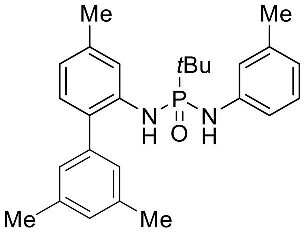

***P-tert*-butyl-*N*-(3-methylphenyl)-*N*'-{[3-methyl-6-(3, 5-dimethylphenyl)phenyl]}phospho-nic diamide (b18)**. White solid. 31 mg, 74% yield. **HPLC** conditions: Daicel CHIRALCEL® OD-H column with hexane/2-propanol = 90/10, flow = 1.0 mL/min, λ = 254 nm. Retention times: 6.7 min [(*R*)-enantiomer], 10.9 min [(*S*)-enantiomer]. $[\alpha]_D^{25}$: 30 (*c* 0.100, CH_2Cl_2, 85% ee). **^{1}H NMR** (400 MHz, $CDCl_3$): δ 7.52 (s, 1H), 7.13 – 7.04 (m, 2H), 7.01 (s, 1H), 6.99 – 6.93 (m, 4H), 6.80 (d, *J* = 7.8 Hz, 1H), 6.76 (d, *J* = 7.3 Hz, 1H), 5.28 (d, *J* = 15.0 Hz, 1H), 4.65 (d, *J* = 8.4 Hz, 1H), 2.32 (s, 6H), 2.29 (s, 3H), 2.27 (s, 3H), 1.11 (d, *J* = 16.0 Hz, 9H). **^{13}C NMR** (101 MHz, $CDCl_3$): δ 141.30, 138.98, 138.71, 138.62, 138.34, 137.12 (d, J_{CP} = 2.3 Hz), 129.65, 129.22, 128.97, 128.13 (d, J_{CP} = 8.0 Hz), 127.07, 122.66, 122.33, 119.52 (d, J_{CP} = 5.3 Hz), 118.24 (d, J_{CP} = 2.5 Hz), 115.96 (d, J_{CP} = 4.9 Hz), 34.32 (d, J_{CP} = 114.7 Hz), 25.19, 21.46, 21.44, 21.24, 21.24. **^{31}P NMR** (162 MHz, $CDCl_3$): δ 27.5. **HRMS** (ESI) calcd for $C_{26}H_{33}N_2NaOP$ $(M+Na)^+$: 443.2223, found: 443.2212. The absolute configuration of **b18** was determined to be *R* by XRD analysis, see supplementary figure 5 below:

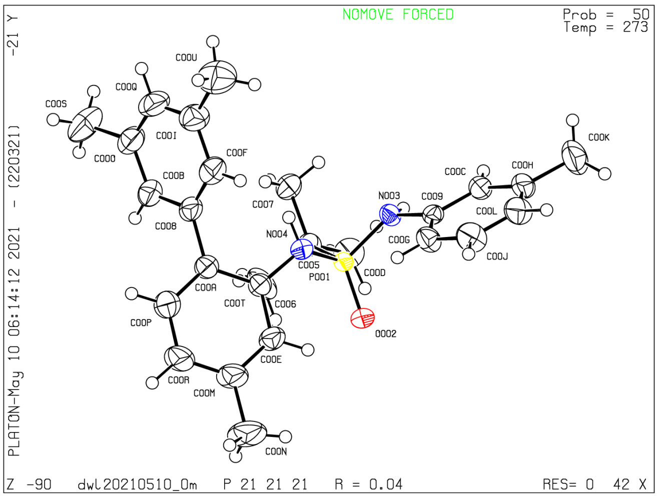

Supplementary Figure 5. XRD analysis result of **b18**

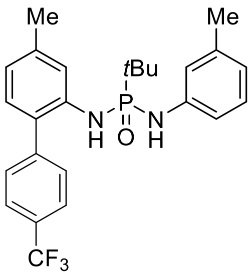

***P-tert*-butyl-*N*-(3-methylphenyl)-*N*'-{[3-methyl-6-(4-trifluoromethylphenyl)phenyl]}phosphonic diamide (b19)**. White solid. 45 mg, 98% yield. **HPLC** conditions: Daicel CHIRALCEL® OD-H column with hexane/2-propanol = 90/10, flow = 1.0 mL/min, λ = 254 nm. Retention times: 9.8 min [(*R*)-enantiomer], 12.6 min [(*S*)-enantiomer]. $[\alpha]_D{}^{25}$: 108 (*c* 0.100, CH_2Cl_2, 86% ee). The absolute configuration was assigned by analogy with compound (*R*)-**b18**. **^{1}H NMR** (400 MHz, $CDCl_3$): δ 7.66 (d, *J* = 7.9 Hz, 2H), 7.60 (s, 1H), 7.42 (d, *J* = 7.9 Hz, 2H), 7.11 (t, *J* = 7.7 Hz, 1H), 7.03 (d, *J* = 7.7 Hz, 1H), 6.98 (d, *J* = 8.4 Hz, 1H), 6.95 (s, 1H), 6.84 (d, *J* = 7.7 Hz, 1H), 6.81 (d, *J* = 7.5 Hz, 1H), 4.87 (d, *J* = 12.7 Hz, 1H), 4.65 (d, *J* = 10.4 Hz, 1H), 2.33 (s, 3H), 2.29 (s, 3H), 1.13 (d, *J* = 16.0 Hz, 9H). **^{13}C NMR** (101 MHz, $CDCl_3$): δ 142.47 (q, J_{CF} = 1.1 Hz), 140.86, 140.85, 139.68, 139.07, 137.41 (d, J_{CP} = 1.9 Hz), 129.82, 129.79, 129.78 (q, J_{CF} = 33.0 Hz), 129.05, 126.51 (d, J_{CP} = 8.0 Hz), 125.96 (q, J_{CF} = 3.7 Hz), 124.0 (q, J_{CF} = 273.5 Hz), 123.01, 122.62, 119.96 (d, J_{CP} = 5.4 Hz), 118.81 (d, J_{CP} = 2.3 Hz), 116.33 (d, J_{CP} = 4.9 Hz), 34.29 (d, J_{CP} = 114.5 Hz), 25.24, 21.46, 21.40. **^{31}P NMR** (162 MHz, $CDCl_3$): δ 28.0. **HRMS** (ESI) calcd for $C_{25}H_{28}F_3N_2NaOP$ $(M+Na)^+$: 483.1784, found: 483.1777.

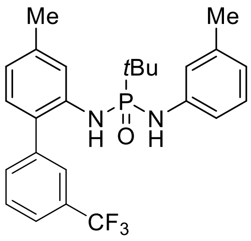

***P-tert*-butyl-*N*-(3-methylphenyl)-*N*'-{[3-methyl-6-(3-trifluoromethylphenyl)phenyl]}pho-**

sphonic diamide (b20). White solid. 41 mg, 89% yield. **HPLC** conditions: Daicel CHIRALPAK® AD-H column with hexane/2-propanol = 90/10, flow = 1.0 mL/min, λ = 254 nm. Retention times: 7.6 min [(*R*)-enantiomer], 10.6 min [(*S*)-enantiomer]. $[\alpha]_D{}^{25}$: - 22 (*c* 0.100, CH_2Cl_2, 92% ee). The absolute configuration was assigned by analogy with compound (*R*)-**b18**. **^{1}H NMR** (400 MHz, $CDCl_3$): δ 7.67 – 7.62 (m, 2H), 7.60 – 7.48 (m, 3H), 7.12 – 7.02 (m, 2H), 6.99 – 6.93 (m, 2H), 6.84 (d, *J* = 7.7 Hz, 1H), 6.78 (d, *J* = 7.5 Hz, 1H), 4.91 (d, *J* = 13.6 Hz, 1H), 4.65 (d, *J* = 9.4 Hz, 1H), 2.31 (s, 3H), 2.28 (s, 3H), 1.11 (d, *J* = 16.1 Hz, 9H). **^{13}C NMR** (101 MHz, $CDCl_3$): δ 140.96, 139.71, 139.48, 139.09, 137.15 (d, J_{CP} = 2.3 Hz), 132.91 (q, J_{CF} = 0.9 Hz), 131.48 (q, J_{CF} = 32.4 Hz), 129.89, 129.70, 129.03, 126.39 (d, J_{CP} = 7.9 Hz), 126.11 (q, J_{CF} = 3.7 Hz), 124.45 (q, J_{CF} = 3.9 Hz), 123.86 (q, J_{CF} = 274.1 Hz), 122.96, 122.72, 119.76 (d, J_{CP} = 5.3 Hz), 118.76 (d, J_{CP} = 2.5 Hz), 116.14 (d, J_{CP} = 4.8 Hz), 34.28 (d, J_{CP} = 114.6 Hz), 25.16, 21.48, 21.43. **^{31}P NMR** (162 MHz, $CDCl_3$): δ 27.9. **HRMS** (ESI) calcd for $C_{25}H_{28}F_3N_2NaOP$ $(M+Na)^+$: 483.1784, found: 483.1781.

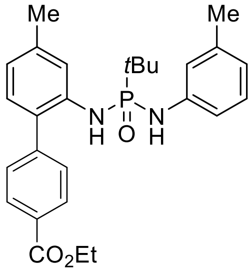

***P-tert*-butyl-*N*-(3-methylphenyl)-*N*'-{[3-methyl-6-(4-ethoxycarbonylphenyl)phenyl]}phosphonic diamide (b21)**. White solid. 45 mg, 98% yield. **HPLC** conditions: Daicel CHIRALPAK® IC column with hexane/2-propanol = 80/20, flow = 1.0 mL/min, λ = 254 nm. Retention times: 10.2 min [(*S*)-enantiomer], 16.6 min [(*R*)-enantiomer]. $[\alpha]_D{}^{25}$: 197 (*c* 0.100, CH_2Cl_2, 86% ee). The absolute configuration was assigned by analogy with compound (*R*)-**b18**. **^{1}H NMR** (400 MHz, $CDCl_3$): δ 8.09 (d, *J* = 8.0 Hz, 2H), 7.59 (s, 1H), 7.39 (d, *J* = 8.0 Hz, 2H), 7.11 (t, *J* = 7.7 Hz, 1H), 7.05 (d, *J* = 7.7 Hz, 1H), 6.98 (d, *J* = 8.3 Hz, 1H), 6.95 (s, 1H), 6.83 (d, *J* = 7.8 Hz, 1H), 6.80 (d, *J* = 7.5 Hz, 1H), 5.01 (d, *J* = 13.4 Hz, 1H), 4.62 (d, *J* = 9.9 Hz, 1H), 4.41 (q, *J* = 7.1 Hz, 2H), 2.32 (s, 3H), 2.29 (s, 3H), 1.43 (t, *J* = 7.1 Hz, 3H), 1.12 (d, *J* = 16.1 Hz, 9H). **^{13}C NMR** (101 MHz, $CDCl_3$): δ 166.24, 143.39, 141.01, 139.57, 139.05, 137.26 (d, J_{CP} = 1.2 Hz), 130.30, 129.78, 129.66, 129.35, 129.04, 126.86 (d, J_{CP} = 7.6 Hz), 122.92, 122.56, 119.83 (d, J_{CP} = 5.0 Hz), 118.63, 116.22 (d, J_{CP} = 5.0 Hz), 61.18, 34.34 (d, J_{CP} = 114.6 Hz), 25.28, 21.50, 21.47, 14.34. **^{31}P NMR** (162 MHz, $CDCl_3$): δ 28.0. **HRMS** (ESI) calcd for $C_{27}H_{33}N_2NaO_3P$ $(M+Na)^+$: 487.2121, found: 487.2108.

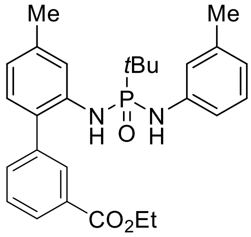

***P-tert*-butyl-*N*-(3-methylphenyl)-*N*'-{[3-methyl-6-(3-ethoxycarbonylphenyl)phenyl]}phosphonic diamide (b22)**. White solid. 41 mg, 89% yield. **HPLC** conditions: Daicel

CHIRALPAK® AD-H column with hexane/2-propanol = 90/10, flow = 1.0 mL/min, λ = 254 nm. Retention times: 16.5 min [(*R*)-enantiomer], 23.7 min [(*S*)-enantiomer]. $[\alpha]_D^{25}$: 91 (*c* 0.100, CH_2Cl_2, 92% ee). The absolute configuration was assigned by analogy with compound (*R*)-**b18**. **^{1}H NMR** (400 MHz, $CDCl_3$): δ 8.10 – 8.03 (m, 2H), 7.58 (s, 1H), 7.52 (d, *J* = 4.8 Hz, 2H), 7.13 – 7.05 (m, 2H), 6.99 – 6.94 (m, 2H), 6.84 (d, *J* = 7.8 Hz, 1H), 6.77 (d, *J* = 7.5 Hz, 1H), 5.03 (d, *J* = 14.0 Hz, 1H), 4.68 (d, *J* = 9.2 Hz, 1H), 4.39 (q, *J* = 7.1 Hz, 2H), 2.31 (s, 3H), 2.28 (s, 3H), 1.38 (t, *J* = 7.1 Hz, 3H), 1.10 (d, *J* = 16.1 Hz, 9H). **^{13}C NMR** (101 MHz, $CDCl_3$): δ 166.11, 141.26, 139.40, 138.98, 138.83, 137.16 (d, J_{CP} = 2.5 Hz), 133.87, 131.29, 130.25, 129.91, 129.29, 128.98, 128.85, 126.85 (d, J_{CP} = 8.0 Hz), 122.72, 122.59, 119.68 (d, J_{CP} = 5.2 Hz), 118.60 (d, J_{CP} = 2.6 Hz), 116.05 (d, J_{CP} = 4.9 Hz), 61.23, 34.34 (d, J_{CP} = 114.5 Hz), 25.24, 21.47, 21.43, 14.31. **^{31}P NMR** (162 MHz, $CDCl_3$): δ 27.8. **HRMS** (ESI) calcd for $C_{27}H_{33}N_2NaO_3P$ $(M+Na)^+$: 487.2121, found: 487.2111.

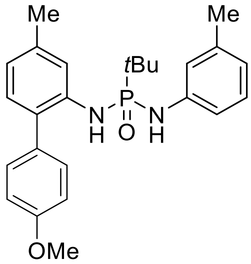

***P*-*tert*-butyl-*N*-(3-methylphenyl)-*N*'-{[3-methyl-6-(4-methoxyphenyl)phenyl]} phosphonic diamide (b23)**. White solid. 41 mg, 98% yield. **HPLC** conditions: Daicel CHIRALCEL® OD-H column with hexane/2-propanol = 90/10, flow = 1.0 mL/min, λ = 254 nm. Retention times: 8.6 min [(*R*)-enantiomer], 13.5 min [(*S*)-enantiomer]. $[\alpha]_D^{25}$: 129 (*c* 0.100, CH_2Cl_2, 79% ee). The absolute configuration was assigned by analogy with compound (*R*)-**b18**. **^{1}H NMR** (400 MHz, $CDCl_3$): δ 7.53 (s, 1H), 7.24 (d, *J* = 8.7 Hz, 2H), 7.09 (t, *J* = 8.0 Hz, 1H), 7.03 (d, *J* = 7.7 Hz, 1H), 6.99 – 6.93 (m, 4H), 6.82 – 6.74 (m, 2H), 5.13 (d, *J* = 14.2 Hz, 1H), 4.63 (s, 1H), 3.85 (s, 3H), 2.30 (s, 3H), 2.28 (s, 3H), 1.11 (d, *J* = 16.1 Hz, 9H). **^{13}C NMR** (101 MHz, $CDCl_3$): δ 159.08, 141.25, 138.98, 138.57, 137.58 (d, J_{CP} = 1.9 Hz), 130.62, 130.52, 129.98, 128.98, 127.60 (d, J_{CP} = 7.9 Hz), 122.71, 122.30, 119.68 (d, J_{CP} = 5.3 Hz), 118.24 (d, J_{CP} = 2.2 Hz), 116.08 (d, J_{CP} = 4.9 Hz), 114.48, 55.33, 34.31 (d, J_{CP} = 114.6 Hz), 25.28, 21.47, 21.43. **^{31}P NMR** (162 MHz, $CDCl_3$): δ 27.7. **HRMS** (ESI) calcd for $C_{25}H_{31}N_2NaO_2P$ $(M+Na)^+$: 445.2015, found: 445.2006.

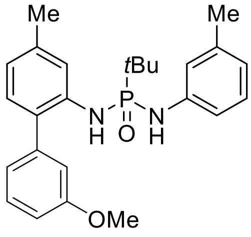

***P*-*tert*-butyl-*N*-(3-methylphenyl)-*N*'-{[3-methyl-6-(3-methoxyphenyl)phenyl]} phosphonic diamide (b24)**. White solid. 38 mg, 89% yield. **HPLC** conditions: Daicel CHIRALPAK® AD-H column with hexane/2-propanol = 80/20, flow = 1.0 mL/min, λ = 254 nm. Retention times: 5.7 min [(*R*)-enantiomer], 9.1 min [(*S*)-enantiomer]. $[\alpha]_D^{25}$: 92 (*c* 0.050,

CH_2Cl_2, 81% ee). The absolute configuration was assigned by analogy with compound (*R*)-**b18**. **^{1}H NMR** (400 MHz, $CDCl_3$): δ 7.55 (s, 1H), 7.35 (t, *J* = 7.9 Hz, 1H), 7.12 – 7.05 (m, 2H), 6.99 – 6.94 (m, 2H), 6.94 – 6.89 (m, 2H), 6.87 (s, 1H), 6.81 (d, *J* = 7.7 Hz, 1H), 6.76 (d, *J* = 7.5 Hz, 1H), 5.23 (d, *J* = 14.2 Hz, 1H), 4.61 (d, *J* = 9.2 Hz, 1H), 3.79 (s, 3H), 2.31 (s, 3H), 2.28 (s, 3H), 1.10 (d, *J* = 16.1 Hz, 9H). **^{13}C NMR** (101 MHz, $CDCl_3$):δ 160.20, 141.23, 139.94, 139.00, 138.93, 137.21 (d, J_{CP} = 2.1 Hz), 130.11, 129.65, 128.98, 127.84 (d, J_{CP} = 8.0 Hz), 122.71, 122.33, 121.42, 119.63 (d, J_{CP} = 5.3 Hz), 118.31 (d, J_{CP} = 2.7 Hz), 116.01 (d, J_{CP} = 4.8 Hz), 114.70, 113.53, 55.25, 34.32 (d, J_{CP} = 114.5 Hz), 25.23, 21.46. **^{31}P NMR** (162 MHz, $CDCl_3$): δ 27.7. **HRMS** (ESI) calcd for $C_{25}H_{31}N_2NaO_2P$ $(M+Na)^+$: 445.2015, found: 445.2006.

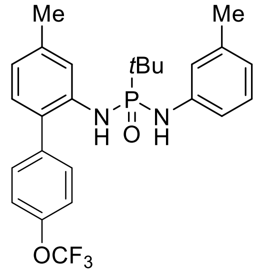

***P*-*tert*-butyl-*N*-(3-methylphenyl)-*N*'-{[3-methyl-6-(4-trifluoromethoxyphenyl)phenyl]} phosphonic diamide (b25)**. White solid. 47 mg, 98% yield. **HPLC** conditions: Daicel CHIRALPAK® AD-H column with hexane/2-propanol = 90/10, flow = 1.0 mL/min, λ = 254 nm. Retention times: 7.2 min [(*R*)-enantiomer], 10.2 min [(*S*)-enantiomer]. $[\alpha]_D^{25}$: 7 (*c* 0.050, CH_2Cl_2, 85% ee). The absolute configuration was assigned by analogy with compound (*R*)-**b18**. **^{1}H NMR** (400 MHz, $CDCl_3$): δ 7.56 (s, 1H), 7.33 (d, *J* = 8.7 Hz, 2H), 7.26 (d, *J* = 8.7 Hz, 2H), 7.10 (t, *J* = 7.7 Hz, 1H), 7.03 (d, *J* = 7.7 Hz, 1H), 7.00 – 6.93 (m, 2H), 6.82 (d, *J* = 8.5 Hz, 1H), 6.80 (d, *J* = 7.5 Hz, 1H), 4.88 (d, *J* = 13.2 Hz, 1H), 4.63 (d, *J* = 10.1 Hz, 1H), 2.31 (s, 3H), 2.29 (s, 3H), 1.11 (d, *J* = 16.1 Hz, 9H). **^{13}C NMR** (101 MHz, $CDCl_3$): δ 148.64 (q, J_{CF} = 1.8 Hz), 140.92, 139.39, 139.09, 137.39 (d, J_{CP} = 1.8 Hz), 137.29, 130.93, 129.79, 129.05, 126.58 (d, J_{CP} = 7.9 Hz), 122.95, 122.54, 121.54, 120.44 (q, J_{CF} = 258.9 Hz), 119.80 (d, J_{CP} = 5.3 Hz), 118.64 (d, J_{CP} = 2.3 Hz), 116.18 (d, J_{CP} = 4.8 Hz), 34.28 (d, J_{CP} = 114.7 Hz), 25.22, 21.49, 21.44. **^{31}P NMR** (162 MHz, $CDCl_3$): δ 27.9. **HRMS** (ESI) calcd for $C_{25}H_{28}F_3N_2NaO_2P$ $(M+Na)^+$: 499.1733, found: 499.1724.

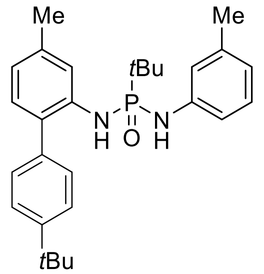

***P*-*tert*-butyl-*N*-(3-methylphenyl)-*N*'-{[3-methyl-6-(4-*tert*-butylphenyl)phenyl]} phosphon-ic diamide (b26)**. White solid. 36 mg, 85% yield. **HPLC** conditions: Daicel CHIRALCEL® OD-H column with hexane/2-propanol = 90/10, flow = 1.0 mL/min, λ = 254 nm. Retention times: 5.1 min [(*R*)-enantiomer], 7.9 min [(*S*)-enantiomer]. $[\alpha]_D^{25}$: - 55 (*c*

0.100, CH_2Cl_2, 83% ee). The absolute configuration was assigned by analogy with compound (*R*)-**b18**. **^{1}H NMR** (400 MHz, $CDCl_3$): δ 7.51 (s, 1H), 7.46 (d, J = 8.0 Hz, 2H), 7.26 (d, J = 8.0 Hz, 2H), 7.12 – 7.05 (m, 2H), 6.98 – 6.93 (m, 2H), 6.81 (d, J = 7.7 Hz, 1H), 6.77 (d, J = 7.5 Hz, 1H), 5.14 (d, J = 14.1 Hz, 1H), 4.61 (d, J = 9.2 Hz, 1H), 2.30 (s, 3H), 2.28 (s, 3H), 1.35 (s, 9H), 1.08 (d, J = 16.0 Hz, 9H). **^{13}C NMR** (101 MHz, $CDCl_3$): δ 150.75, 141.34, 138.94, 138.64, 137.38 (d, J_{CP} = 2.3 Hz), 135.46, 129.71, 129.06, 128.96, 128.09 (d, J_{CP} = 7.9 Hz), 125.97, 122.64, 122.38, 119.66 (d, J_{CP} = 5.3 Hz), 118.41 (d, J_{CP} = 2.5 Hz), 116.04 (d, J_{CP} = 4.9 Hz), 34.62, 34.30 (d, J_{CP} = 115.1 Hz), 31.31, 25.19, 21.49, 21.43. **^{31}P NMR** (162 MHz, $CDCl_3$): δ 27.7. **HRMS** (ESI) calcd for $C_{28}H_{37}N_2NaOP$ $(M+Na)^+$: 471.2536, found: 471.2525.

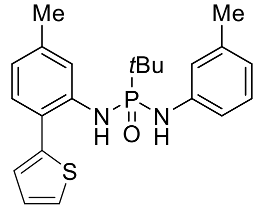

***P-tert*-butyl-*N*-(3-methylphenyl)-*N*'-{[3-methyl-6-(thiophen-2-ylphenyl)phenyl]} phosph-onic diamide (b27)**. Brown solid. 23 mg, 57% yield. **HPLC** conditions: Daicel CHIRALPAK® AD-H column with hexane/2-propanol = 90/10, flow = 1.0 mL/min, λ = 254 nm. Retention times: 10.4 min [(*R*)-enantiomer], 17.8 min [(*S*)-enantiomer]. $[\alpha]_D^{25}$: - 25 (*c* 0.050, CH_2Cl_2, 68% ee). The absolute configuration was assigned by analogy with compound (*R*)-**b18**. **^{1}H NMR** (400 MHz, $CDCl_3$): δ 7.57 (s, 1H), 7.39 (d, J = 5.1 Hz, 1H), 7.18 (d, J = 7.7 Hz, 1H), 7.14 – 7.06 (m, 2H), 7.01 – 6.94 (m, 3H), 6.82 – 6.73 (m, 2H), 5.50 (d, J = 14.3 Hz, 1H), 4.67 (d, J = 8.9 Hz, 1H), 2.29 (s, 3H), 2.28 (s, 3H), 1.16 (d, J = 16.1 Hz, 9H). **^{13}C NMR** (101 MHz, $CDCl_3$): δ 141.21, 139.92, 139.72, 139.02, 138.22 (d, J_{CP} = 2.1 Hz), 130.88, 129.00, 127.68, 126.69, 126.33, 122.77, 122.29, 120.00 (d, J_{CP} = 8.0 Hz), 119.71 (d, J_{CP} = 5.3 Hz), 118.47 (d, J_{CP} = 2.6 Hz), 116.11 (d, J_{CP} = 4.9 Hz), 34.39 (d, J_{CP} = 114.4 Hz), 25.25, 21.50, 21.48. **^{31}P NMR** (162 MHz, $CDCl_3$): δ 27.9. **HRMS** (ESI) calcd for $C_{22}H_{27}N_2NaOPS$ $(M+Na)^+$: 421.1474, found: 421.1468.

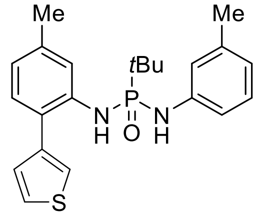

***P-tert*-butyl-*N*-(3-methylphenyl)-*N*'-{[3-methyl-6-(thiophen-3-ylphenyl)phenyl]} phosph-onic diamide (b28)**. White solid. 27 mg, 68% yield. **HPLC** conditions: Daicel CHIRALPAK® AD-H column with hexane/2-propanol = 90/10, flow = 1.0 mL/min, λ = 254 nm. Retention times: 11.2 min [(*R*)-enantiomer], 17.1 min [(*S*)-enantiomer]. $[\alpha]_D^{25}$: 145 (*c* 0.050, CH_2Cl_2, 78% ee). The absolute configuration was assigned by analogy with compound (*R*)-**b18**. **^{1}H NMR** (400 MHz, $CDCl_3$): δ 7.54 (s, 1H), 7.46 (dd, J = 4.9, 2.9 Hz, 1H), 7.20 (dd, J = 2.9, 1.3 Hz, 1H), 7.15 – 7.06 (m, 3H), 7.01 – 6.94 (m, 2H), 6.79 (d, J = 6.8 Hz, 1H), 6.78 (d, J = 8.3 Hz, 1H), 5.25 (d, J = 14.0 Hz, 1H), 4.64 (d, J = 9.5 Hz, 1H), 2.30 (s, 3H), 2.28 (s,

3H), 1.14 (d, J = 16.1 Hz, 9H). **^{13}C NMR** (101 MHz, $CDCl_3$): δ 141.11, 139.08, 139.05, 138.84, 137.78 (d, J_{CP} = 2.0 Hz), 129.83, 129.04, 128.51, 127.03, 123.12, 122.87, 122.71 (d, J_{CP} = 7.9 Hz), 122.29, 119.76 (d, J_{CP} = 5.1 Hz), 118.34 (d, J_{CP} = 2.1 Hz), 116.15 (d, J_{CP} = 4.9 Hz), 34.30 (d, J_{CP} = 114.8 Hz), 25.27, 21.51, 21.48. **^{31}P NMR** (162 MHz, $CDCl_3$): δ 27.9. **HRMS** (ESI) calcd for $C_{22}H_{27}N_2NaOPS$ $(M+Na)^+$: 421.1474, found: 421.1465.

General Procedure for Copper-Bisoxazoline Catalyzed C-3 Arylation. NaOTf (6.8 mg, 0.04 mmol) was dissolved into MeOH (20 mL) and the NaOTf/MeOH solution was stored for use. To a dried Schlenk tube was charged with NaOTf/MeOH solution (0.5 mL) and solvent was removed under vacuum. CuI (1.9 mg, 0.01 mmol), **L6** (6.9 mg, 0.015 mmol) and CH_2Cl_2 (2 mL) were added into the tube and the mixture was stirred at rt for 0.5 h under N_2. Substrate (0.1 mmol, 1.0 equiv.), diaryliodonium hexafluoroantimonate salt (0.2 mmol, 2.0 equiv.) and 2, 6-Di-*tert*-butylpyridine (DTBP, 45 µL, 2.0 equiv.) were added into the mixture under N_2. The reaction was heated to 60 °C and stirred for 24 h. After cooling to room temperature, the reaction mixture was filtrated, concentrated under vacuum. The crude product was purified via flash chromatography (PE/EA 5:1 to 2:1, v/v) to afford product. The absolute configuration of product was assigned to be ***R*** by XRD analysis of **d1**, as stated in part 1.6.

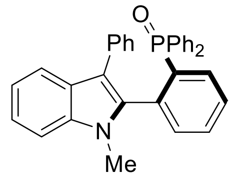

(2-(1-methyl-3-phenyl-1*H*-indol-2-yl)phenyl)diphenylphosphine oxide (d1). Known comp-ound.[5] White solid. 31 mg, 64% yield. **HPLC** conditions: Daicel CHIRALCEL® OD-H column with hexane/2-propanol = 90/10, flow = 1.0 mL/min, λ = 254 nm. Retention times: 8.3 min [(*R*)-enantiomer], 10.3 min [(*S*)-enantiomer]. $[\alpha]_D{}^{25}$: 0.0 (*c* 0.100, CH_2Cl_2, 85% ee). **^{1}H NMR** (400 MHz, $CDCl_3$): δ 8.02 (dd, J = 12.7, 7.8 Hz, 1H), 7.71 – 7.64 (m, 1H), 7.63 – 7.58 (m, 2H), 7.57 – 7.50 (m, 1H), 7.32 (tt, J = 7.1, 1.4 Hz, 1H), 7.24 – 7.15 (m, 4H), 7.14 – 7.05 (m, 8H), 7.04 (d, J = 7.5 Hz, 2H), 6.85 (td, J = 7.8, 2.9 Hz, 2H), 3.15 (s, 3H). The absolute configuration of **d1** was determined to be ***R*** by XRD analysis, see supplementary figure 6 below:

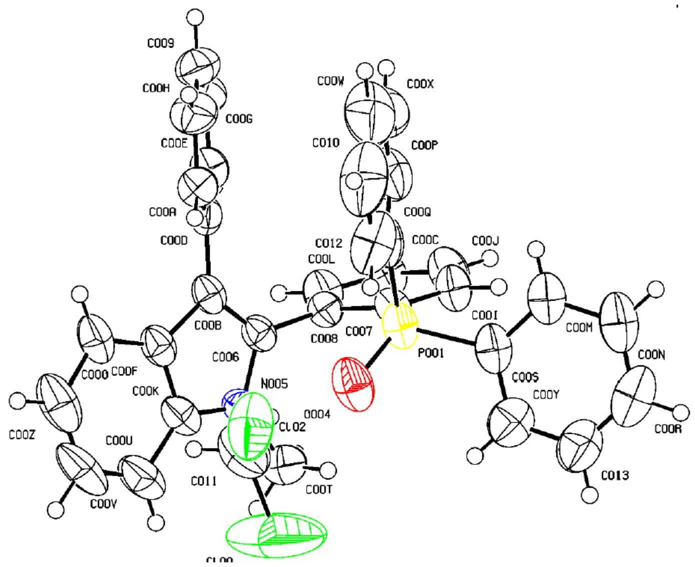

Supplementary Figure 6. XRD analysis result of **d1**

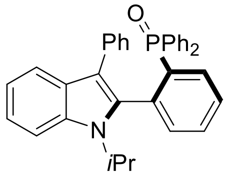

(2-(1-isopropyl-3-phenyl-1*H*-indol-2-yl)phenyl)diphenylphosphine oxide (d2). White solid. 37 mg, 72% yield. **HPLC** conditions: Daicel CHIRALCEL® OD-H column with hexane/2-propanol = 97/03, flow = 1.0 mL/min, λ = 254 nm. Retention times: 11.7 min [(*R*)-enantiomer], 13.9 min [(*S*)-enantiomer]. $[\alpha]_D^{25}$: 77 (*c* 0.100, CH_2Cl_2, 86% ee). The absolute configuration was assigned by analogy with compound (*R*)-**d1**. **^{1}H NMR** (400 MHz, $CDCl_3$): δ 7.67 – 7.55 (m, 4H), 7.48 – 7.38 (m, 3H), 7.36 – 7.26 (m, 4H), 7.22 (dd, *J* = 7.4, 1.5 Hz, 1H), 7.19 – 7.08 (m, 3H), 7.06 – 6.98 (m, 3H), 6.97 – 6.87 (m, 5H), 4.25 (p, *J* = 6.9 Hz, 1H), 1.72 (d, *J* = 6.9 Hz, 3H), 1.48 (d, *J* = 6.9 Hz, 3H). **^{13}C NMR** (101 MHz, $CDCl_3$): δ 137.90 (d, J_{CP} = 6.9 Hz), 135.29 (t, J_{CP} = 3.0 Hz), 135.19 (d, J_{CP} = 1.4 Hz), 134.91 (d, J_{CP} = 9.8 Hz), 134.61 (d, J_{CP} = 104.5 Hz), 134.58, 134.46, 133.23 (d, J_{CP} = 100.5 Hz), 131.82 (d, J_{CP} = 9.5 Hz), 131.45 (d, J_{CP} = 2.3 Hz), 131.33 (d, J_{CP} = 10.1 Hz), 131.30 (d, J_{CP} = 105.0 Hz), 131.10 (d, J_{CP} = 2.3 Hz), 130.95 (t, J_{CP} = 2.3 Hz), 129.33, 128.33 (d, J_{CP} = 12.1 Hz), 128.15 (d, J_{CP} = 4.1 Hz), 127.98 (d, J_{CP} = 12.5 Hz), 127.85, 124.80, 121.36, 119.79, 119.30, 115.40, 112.34, 48.86, 22.06, 20.77. **^{31}P NMR** (162 MHz, $CDCl_3$): δ 29.3. **HRMS** (ESI) calcd for $C_{35}H_{30}NNaOP$ $(M+Na)^+$: 534.1957, found: 534.1953.

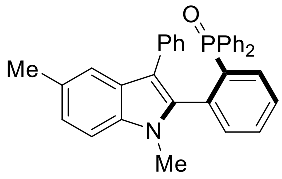

(2-(1, 5-dimethyl-3-phenyl-1*H*-indol-2-yl)phenyl)diphenylphosphine oxide (d3). White solid. 28 mg, 56% yield. **HPLC** conditions: Daicel CHIRALCEL® OD-H column with hexane/2-propanol = 90/10, flow = 1.0 mL/min, λ = 254 nm. Retention times: 7.2 min

[(*R*)-enantiomer], 8.9 min [(*S*)-enantiomer]. $[\alpha]_D^{25}$: 0.0 (*c* 0.100, CH_2Cl_2, 86% ee). The absolute configuration was assigned by analogy with compound (*R*)-**d1**. **^{1}H NMR** (400 MHz, $CDCl_3$): δ 8.02 (ddd, *J* = 12.8, 7.9, 1.5 Hz, 1H), 7.68 – 7.64 (m, 1H), 7.63 – 7.57 (m, 1H), 7.55 – 7.50 (m, 1H), 7.38 (s, 1H), 7.32 (td, *J* = 7.1, 1.5 Hz, 1H), 7.20 (d, *J* = 7.2 Hz, 1H), 7.18 – 7.13 (m, 2H), 7.13 – 7.08 (m, 6H), 7.07 – 7.00 (m, 4H), 6.93 (d, *J* = 8.3 Hz, 1H), 6.88 (td, *J* = 7.7, 3.0 Hz, 2H), 3.08 (s, 3H), 2.42 (s, 3H). **^{13}C NMR** (101 MHz, $CDCl_3$): δ 135.87 (d, J_{CP} = 8.3 Hz), 135.11, 134.85 (d, J_{CP} = 9.9 Hz), 134.65 (d, J_{CP} = 10.2 Hz), 134.51, 134.15 (d, J_{CP} = 100.7 Hz), 132.86 (d, J_{CP} = 105.9 Hz), 131.49 (d, J_{CP} = 4.1 Hz), 131.39 (d, J_{CP} = 4.1 Hz), 131.24 (d, J_{CP} = 3.8 Hz), 131.21 (d, J_{CP} = 3.9 Hz), 130.83 (d, J_{CP} = 2.9 Hz), 130.51 (d, J_{CP} = 106.0 Hz), 129.11, 129.01, 128.85 (d, J_{CP} = 11.4 Hz), 128.16, 128.04, 127.30 (d, J_{CP} = 12.5 Hz), 126.50, 125.08, 123.66, 118.97, 115.04, 109.21, 30.45, 21.54. **^{31}P NMR** (162 MHz, $CDCl_3$): δ 29.7. **HRMS** (ESI) calcd for $C_{34}H_{28}NNaOP$ $(M+Na)^+$: 520.1801, found: 520.1796.

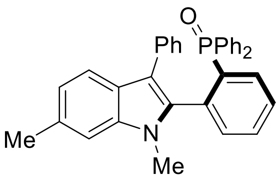

(2-(1, 6-dimethyl-3-phenyl-1*H*-indol-2-yl)phenyl)diphenylphosphine oxide (d4). White solid. 33 mg, 66% yield. **HPLC** conditions: Daicel CHIRALCEL® OD-H column with hexane/2-propanol = 90/10, flow = 1.0 mL/min, λ = 254 nm. Retention times: 7.5 min [(*R*)-enantiomer], 9.8 min [(*S*)-enantiomer]. $[\alpha]_D^{25}$: 0.0 (*c* 0.100, CH_2Cl_2, 69% ee). The absolute configuration was assigned by analogy with compound (*R*)-**d1**. **^{1}H NMR** (400 MHz, $CDCl_3$): δ 7.97 (dd, *J* = 12.7, 7.8 Hz, 1H), 7.63 (t, *J* = 7.4 Hz, 1H), 7.57 (t, *J* = 7.6 Hz, 1H), 7.51 (d, *J* = 4.1 Hz, 1H), 7.47 (d, *J* = 8.4 Hz, 1H), 7.30 (t, *J* = 7.3 Hz, 1H), 7.23 – 7.13 (m, 4H), 7.12 – 7.01 (m, 8H), 6.90 (d, *J* = 8.2 Hz, 1H), 6.87 (dd, *J* = 7.7, 2.9 Hz, 2H), 6.84 (s, 1H), 3.11 (s, 3H), 2.48 (s, 3H). **^{13}C NMR** (101 MHz, $CDCl_3$): δ 136.94, 135.99 (d, J_{CP} = 8.8 Hz), 135.27, 134.89 (d, J_{CP} = 9.5 Hz), 134.71 (d, J_{CP} = 9.3 Hz), 134.48 (d, J_{CP} = 101.3 Hz), 133.91 (d, J_{CP} = 2.7 Hz), 133.04 (d, J_{CP} = 105.4 Hz), 132.01, 131.62 (d, J_{CP} = 9.9 Hz), 131.46 (d, J_{CP} = 10.7 Hz), 131.36 (d, J_{CP} = 8.2 Hz), 130.89, 130.72 (d, J_{CP} = 105.1 Hz), 129.02, 128.92, 128.28, 128.14, 127.32 (d, J_{CP} = 12.1 Hz), 125.22, 124.34, 121.70, 119.25, 115.43, 109.53, 30.55, 21.99. **^{31}P NMR** (162 MHz, $CDCl_3$): δ 29.6. **HRMS** (ESI) calcd for $C_{34}H_{28}NNaOP$ $(M+Na)^+$: 520.1801, found: 520.1795.

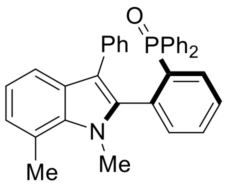

(2-(1, 7-dimethyl-3-phenyl-1*H*-indol-2-yl)phenyl)diphenylphosphine oxide (d5). White solid. 30 mg, 60% yield. **HPLC** conditions: Daicel CHIRALCEL® OD-H column with hexane/2-propanol = 90/10, flow = 1.0 mL/min, λ = 254 nm. Retention times: 7.9 min [(*R*)-enantiomer], 10.6 min [(*S*)-enantiomer]. $[\alpha]_D^{25}$: 0.0 (*c* 0.100, CH_2Cl_2, 88% ee). The absolute configuration was assigned by analogy with compound (*R*)-**d1**. **^{1}H NMR** (400 MHz,

CDCl$_3$): δ 8.06 (ddd, *J* = 12.7, 7.5, 1.7 Hz, 1H), 7.67 – 7.56 (m, 2H), 7.50 (ddd, *J* = 7.1, 4.5, 1.6 Hz, 1H), 7.41 (d, *J* = 7.8 Hz, 1H), 7.31 (td, *J* = 7.0, 1.7 Hz, 1H), 7.28 – 7.22 (m, 2H), 7.16 – 7.01 (m, 10H), 6.95 – 6.83 (m, 4H), 3.31 (s, 3H), 2.58 (s, 3H). **^{13}C NMR** (101 MHz, CDCl$_3$): δ 135.71 (d, J_{CP} = 8.6 Hz), 135.37, 135.24 (d, J_{CP} = 3.5 Hz), 134.89 (d, J_{CP} = 9.2 Hz), 134.59 (d, J_{CP} = 9.7 Hz), 134.43 (d, J_{CP} = 101.4 Hz), 132.92 (d, J_{CP} = 105.6 Hz), 131.50 (d, J_{CP} = 10.2 Hz), 131.44 (d, J_{CP} = 10.3 Hz), 131.27 (d, J_{CP} = 2.5 Hz), 131.16 (d, J_{CP} = 2.8 Hz), 130.58 (d, J_{CP} = 3.0 Hz), 130.56 (d, J_{CP} = 105.7 Hz), 129.25, 128.93 (d, J_{CP} = 11.4 Hz), 128.17, 128.13 (d, J_{CP} = 12.4 Hz), 127.26, 127.17 (d, J_{CP} = 12.5 Hz), 125.29, 125.06, 121.09, 119.88, 117.50, 115.75, 33.88, 20.30. **^{31}P NMR** (162 MHz, CDCl$_3$): δ 28.3. **HRMS** (ESI) calcd for $C_{34}H_{28}NNaOP$ (M+Na)$^+$: 520.1801, found: 520.1796.

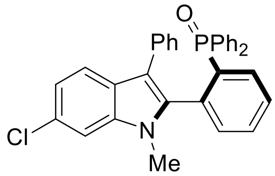

(2-(6-chloro-1-methyl-3-phenyl-1*H*-indol-2-yl)phenyl)diphenylphosphine oxide (d6). Wh-ite solid. 17 mg, 33% yield. **HPLC** conditions: Daicel CHIRALCEL® OD-H column with hexane/2-propanol = 90/10, flow = 1.0 mL/min, λ = 254 nm. Retention times: 8.2 min [(*R*)-enantiomer], 11.7 min [(*S*)-enantiomer]. $[\alpha]_D^{25}$: 0.0 (*c* 0.100, CH_2Cl_2, 82% ee). The absolute configuration was assigned by analogy with compound (*R*)-**d1**. **^{1}H NMR** (400 MHz, CDCl$_3$): δ 7.91 (dd, *J* = 12.5, 7.5 Hz, 1H), 7.73 – 7.64 (m, 2H), 7.63 – 7.57 (m, 1H), 7.54 (dd, *J* = 7.3, 4.5 Hz, 1H), 7.49 (d, *J* = 8.5 Hz, 1H), 7.46 (td, *J* = 7.4, 2.6 Hz, 1H), 7.35 (td, *J* = 7.2, 1.7 Hz, 1H), 7.22 – 7.17 (m, 2H), 7.1 – 7.15 (m, 2H), 7.14 (d, *J* = 2.9 Hz, 1H), 7.13 – 7.09 (m, 3H), 7.08 (d, *J* = 1.8 Hz, 1H), 7.06 – 7.00 (m, 3H), 6.92 (td, *J* = 7.7, 3.0 Hz, 2H), 3.18 (s, 3H). **^{13}C NMR** (101 MHz, CDCl$_3$): δ 136.90, 135.30 (d, J_{CP} = 8.1 Hz), 135.23 (d, J_{CP} = 3.8 Hz), 134.76 (d, J_{CP} = 9.9 Hz), 134.47, 134.38, 134.26 (d, J_{CP} = 100.5 Hz), 134.38, 132.94 (d, J_{CP} = 105.5 Hz), 132.08 (d, J_{CP} = 9.9 Hz), 131.94 (d, J_{CP} = 2.9 Hz), 131.53 (d, J_{CP} = 10.0 Hz), 131.33 (d, J_{CP} = 4.6 Hz), 131.32, 131.22, 131.00 (d, J_{CP} = 2.9 Hz), 130.76 (d, J_{CP} = 105.8 Hz), 129.05 (d, J_{CP} = 11.4 Hz), 128.92, 128.50 (d, J_{CP} = 12.0 Hz), 128.26, 128.15 (d, J_{CP} = 12.3 Hz), 127.86, 127.37 (d, J_{CP} = 12.4 Hz), 125.48, 124.96, 120.45, 120.40, 115.66, 109.46, 30.73. **^{31}P NMR** (162 MHz, CDCl$_3$): δ 29.4. **HRMS** (ESI) calcd for $C_{33}H_{26}ClNOP$ (M+H)$^+$: 518.1435, found: 518.1428.

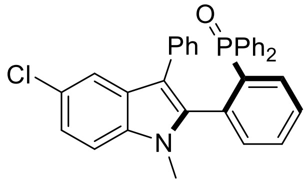

(2-(5-chloro-1-methyl-3-phenyl-1*H*-indol-2-yl)phenyl)diphenylphosphine oxide (d7). Wh-ite solid. 16 mg, 30% yield. **HPLC** conditions: Daicel CHIRALCEL® OD-H column with hexane/2-propanol = 90/10, flow = 1.0 mL/min, λ = 254 nm. Retention times: 8.4 min [(*R*)-enantiomer], 11.4 min [(*S*)-enantiomer]. $[\alpha]_D^{25}$: + 1 (*c* 0.100, CH_2Cl_2, 82% ee). The absolute configuration was assigned by analogy with compound (*R*)-**d1**. **^{1}H NMR** (400 MHz,

CDCl3): δ 7.89 (dd, *J* = 12.8, 7.7 Hz, 1H), 7.66 (t, *J* = 7.4 Hz, 1H), 7.59 (dt, *J* = 7.6, 1.7 Hz, 1H), 7.57 – 7.51 (m, 2H), 7.34 (td, *J* = 7.3, 1.7 Hz, 1H), 7.23 – 7.08 (m, 11H), 7.03 – 6.98 (m, 3H), 6.92 (td, *J* = 7.7, 2.9 Hz, 2H), 3.20 (s, 3H). **^{13}C NMR** (101 MHz, $CDCl_3$): δ 135.88 (d, J_{CP} = 3.8 Hz), 135.29 (d, J_{CP} = 8.0 Hz), 134.89, 134.77 (d, J_{CP} = 9.8 Hz), 134.36 (d, J_{CP} = 9.5 Hz), 134.16 (d, J_{CP} = 100.8 Hz), 132.87 (d, J_{CP} = 105.8 Hz), 134.24, 131.51 (d, J_{CP} = 10.0 Hz), 131.33, 131.30 (d, J_{CP} = 13.0 Hz), 130.98 (d, J_{CP} = 2.9 Hz), 130.64 (d, J_{CP} = 105.6 Hz), 129.07 (d, J_{CP} = 11.6 Hz), 128.87, 128.31, 128.17 (d, J_{CP} = 12.2 Hz), 127.40 (d, J_{CP} = 12.5 Hz), 127.31, 125.58, 125.48, 122.26, 118.86, 115.20, 110.59, 30.79. **^{31}P NMR** (162 MHz, $CDCl_3$): δ 29.4. **HRMS** (ESI) calcd for $C_{33}H_{25}ClNNaOP$ $(M+Na)^+$: 540.1254, found: 540.1256.

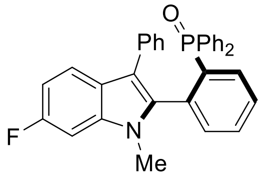

(2-(6-fluoro-1-methyl-3-phenyl-1*H*-indol-2-yl)phenyl)diphenylphosphine oxide (d8). Wh-ite solid. 18 mg, 36%yield. **HPLC** conditions: Daicel CHIRALCEL® OD-H column with hexane/2-propanol = 90/10, flow = 1.0 mL/min, λ = 254 nm. Retention times: 8.1 min [(*R*)-enantiomer], 11.2 min [(*S*)-enantiomer]. $[\alpha]_D^{25}$: 56 (*c* 0.100, CH_2Cl_2, 85% ee). The absolute configuration was assigned by analogy with compound (*R*)-**d1**. **^{1}H NMR** (400 MHz, $CDCl_3$): δ 7.93 (dd, *J* = 12.8, 7.7 Hz, 1H), 7.66 (t, *J* = 7.4 Hz, 1H), 7.59 (t, *J* = 7.5 Hz, 1H), 7.56 – 7.47 (m, 2H), 7.33 (t, *J* = 7.2 Hz, 1H), 7.23 – 7.09 (m, 10H), 7.07 – 7.01 (m, 2H), 6.91 (td, *J* = 7.7, 2.9 Hz, 2H), 6.83 (td, *J* = 9.2, 2.3 Hz, 1H), 6.74 (dd, *J* = 9.9, 2.4 Hz, 1H), 3.13 (s, 3H). **^{13}C NMR** (101 MHz, $CDCl_3$): δ 160.02 (d, J_{CF} = 237.7 Hz), 136.53 (d, J_{CF} = 12.0 Hz), 135.39 (d, J_{CP} = 8.3 Hz), 134.81 (d, J_{CP} = 9.6 Hz), 134.56, 134.47, 134.34 (d, J_{CP} = 94.3 Hz), 132.93 (d, J_{CP} = 106.2 Hz), 131.50 (d, J_{CP} = 10.0 Hz), 131.33 (d, J_{CF} = 10.2 Hz), 131.28 (d, J_{CP} = 2.6 Hz), 130.89 (d, J_{CF} = 2.7 Hz), 130.77 (d, J_{CP} = 109.5 Hz), 129.02 (d, J_{CP} = 11.5 Hz), 128.93, 128.26, 128.12 (d, J_{CP} = 12.2 Hz), 127.29 (d, J_{CP} = 12.3 Hz), 125.44, 122.91, 120.45, 120.35, 115.59, 108.38 (d, J_{CF} = 24.3 Hz), 95.70 (d, J_{CF} = 26.1 Hz), 30.72. **^{31}P NMR** (162 MHz, $CDCl_3$): δ 29.6. **HRMS** (ESI) calcd for $C_{33}H_{25}FNNaOP$ $(M+Na)^+$: 524.1550, found: 524.1542.

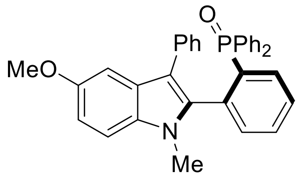

(2-(5-methoxy-1-methyl-3-phenyl-1*H*-indol-2-yl)phenyl)diphenylphosphine oxide (d9). White solid. 34 mg, 66% yield. **HPLC** conditions: Daicel CHIRALPAK® AD-H column with hexane/2-propanol = 85/15, flow = 1.0 mL/min, λ = 254 nm. Retention times: 9.1 min [(*R*)-enantiomer], 17.1 min [(*S*)-enantiomer]. $[\alpha]_D^{25}$: 162 (*c* 0.100, CH_2Cl_2, 80% ee). The absolute configuration was assigned by analogy with compound (*R*)-**d1**. **^{1}H NMR** (400 MHz, $CDCl_3$): δ 7.99 (ddd, *J* = 12.8, 7.8, 1.6 Hz, 1H), 7.66 (tt, *J* = 7.5, 1.6 Hz, 1H), 7.60 (tt, *J* = 7.5,

1.7 Hz, 1H), 7.53 (ddd, J = 7.4, 4.3, 1.4 Hz, 1H), 7.32 (td, J = 7.1, 1.7 Hz, 1H), 7.24 – 7.03 (m, 13H), 6.96 – 6.82 (m, 4H), 3.80 (s, 3H), 3.11 (s, 3H). **^{13}C NMR** (101 MHz, $CDCl_3$): δ 154.59, 135.67 (d, J_{CP} = 8.0 Hz), 135.08, 134.79 (d, J_{CP} = 9.3 Hz), 134.44 (d, J_{CP} = 9.4 Hz), 133.89 (d, J_{CP} = 102.2 Hz), 131.47 (d, J_{CP} = 8.3 Hz), 131.37 (d, J_{CP} = 8.2 Hz), 131.34 (d, J_{CP} = 107.0 Hz), 131.27 (d, J_{CP} = 2.3 Hz), 131.20 (d, J_{CP} = 2.7 Hz), 130.78, 129.50 (d, J_{CP} = 104.6 Hz), 128.85, 128.28, 128.10 (d, J_{CP} = 12.3 Hz), 127.23 (d, J_{CP} = 12.5 Hz), 126.50, 125.15, 115.20, 112.30, 110.27, 101.20, 56.05, 30.59. **^{31}P NMR** (162 MHz, $CDCl_3$): δ 29.7. **HRMS** (ESI) calcd for $C_{34}H_{28}NNaO_2P$ $(M+Na)^+$: 536.1750, found: 536.1749.

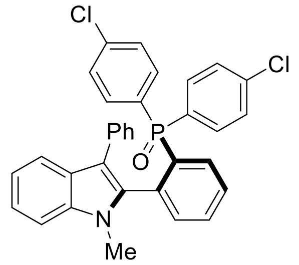

bis(4-chlorophenyl)(2-(1-methyl-3-phenyl-1*H*-indol-2-yl)phenyl)phosphine oxide (d10). Yellow solid. 30 mg, 54% yield. **HPLC** conditions: Daicel CHIRALCEL® OD-H column with hexane/2-propanol = 90/10, flow = 1.0 mL/min, λ = 254 nm. Retention times: 8.2 min [(*R*)-enantiomer], 11.4 min [(*S*)-enantiomer]. $[\alpha]_D^{25}$: - 1 (*c* 0.100, CH_2Cl_2, 66% ee). The absolute configuration was assigned by analogy with compound (*R*)-**d1**. **^{1}H NMR** (400 MHz, $CDCl_3$): δ 8.02 (dd, J = 12.9, 7.7 Hz, 1H), 7.74 (t, J = 7.4 Hz, 1H), 7.69 – 7.59 (m, 3H), 7.25 (t, J = 7.7 Hz, 1H), 7.18 – 7.08 (m, 5H), 7.07 – 6.95 (m, 8H), 6.82 – 6.77 (m, 2H), 3.10 (s, 3H). **^{13}C NMR** (101 MHz, $CDCl_3$): δ 138.05 (d, J_{CP} = 3.3 Hz), 137.88 (d, J_{CP} = 3.5 Hz), 136.44, 135.60 (d, J_{CP} = 8.7 Hz), 134.78, 134.69 (d, J_{CP} = 2.0 Hz), 134.61, 133.92, 133.39 (d, J_{CP} = 99.1 Hz), 132.67 (d, J_{CP} = 11.1 Hz), 132.52 (d, J_{CP} = 11.0 Hz), 131.67 (d, J_{CP} = 2.5 Hz), 130.78 (d, J_{CP} = 107.3 Hz), 129.21 (d, J_{CP} = 11.5 Hz), 128.69 (d, J_{CP} = 107.5 Hz), 128.75, 128.52 (d, J_{CP} = 13.0 Hz), 128.32, 127.72 (d, J_{CP} = 13.0 Hz), 126.04, 125.40, 122.75, 120.26, 119.44, 115.45, 109.41, 30.43. **^{31}P NMR** (162 MHz, $CDCl_3$): δ 28.3. **HRMS** (ESI) calcd for $C_{33}H_{24}Cl_2NNaOP$ $(M+Na)^+$: 574.0865, found: 574.0869.

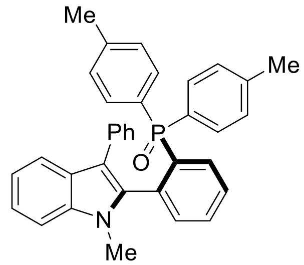

bis(4-methylphenyl)(2-(1-methyl-3-phenyl-1*H*-indol-2-yl)phenyl)phosphine oxide (d11). White solid. 28 mg, 55% yield. **HPLC** conditions: Daicel CHIRALCEL® OD-H column with hexane/2-propanol = 90/10, flow = 1.0 mL/min, λ = 254 nm. Retention times: 7.8 min [(*R*)-enantiomer], 10.6 min [(*S*)-enantiomer]. $[\alpha]_D^{25}$: 61 (*c* 0.100, CH_2Cl_2, 86% ee). The absolute configuration was assigned by analogy with compound (*R*)-**d1**. **^{1}H NMR** (400 MHz, $CDCl_3$): δ 8.03 (ddd, J = 12.8, 7.3, 1.2 Hz, 1H), 7.69 – 7.56 (m, 3H), 7.54 – 7.46 (m, 1H), 7.18

(t, J = 7.5 Hz, 1H), 7.14 – 6.98 (m, 11H), 6.88 (dd, J = 8.2, 2.5 Hz, 2H), 6.64 (dd, J = 8.0, 2.8 Hz, 2H), 3.15 (s, 3H), 2.28 (s, 3H), 2.08 (s, 3H). **^{13}C NMR** (101 MHz, $CDCl_3$): δ 141.50 (d, J_{CP} = 2.9 Hz), 136.55, 135.43 (d, J_{CP} = 8.4 Hz), 135.05, 134.79 (d, J_{CP} = 100.9 Hz), 134.78 (d, J_{CP} = 9.4 Hz), 134.56 (d, J_{CP} = 3.5 Hz), 134.37 (d, J_{CP} = 9.6 Hz), 131.49 (d, J_{CP} = 10.6 Hz), 131.30 (d, J_{CP} = 10.3 Hz), 131.10 (d, J_{CP} = 1.9 Hz), 129.80 (d, J_{CP} = 108.1 Hz), 129.05, 128.98, 128.87, 128.74, 128.07, 127.96, 127.58 (d, J_{CP} = 108.1 Hz), 126.36, 125.11, 122.10, 119.84, 119.39, 115.32, 109.30, 30.54, 21.53, 21.40. **^{31}P NMR** (162 MHz, $CDCl_3$): δ 30.0. **HRMS** (ESI) calcd for $C_{35}H_{30}NNaOP$ $(M+Na)^+$: 534.1957, found: 534.1955.

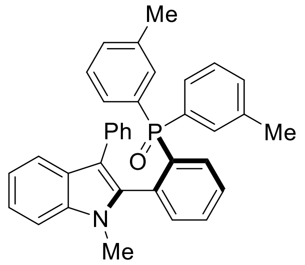

bis(3-methylphenyl)(2-(1-methyl-3-phenyl-1*H*-indol-2-yl)phenyl)phosphine oxide (d12). White solid. 24 mg, 47% yield. **HPLC** conditions: Daicel CHIRALCEL® OD-H column with hexane/2-propanol = 90/10, flow = 1.0 mL/min, λ = 254 nm. Retention times: 7.0 min [(*R*)-enantiomer], 8.0 min [(*S*)-enantiomer]. $[\alpha]_D$ ^{25}: - 117 (*c* 0.100, CH_2Cl_2, 88% ee). The absolute configuration was assigned by analogy with compound (*R*)-**d1**. **^{1}H NMR** (400 MHz, $CDCl_3$): δ 7.96 (dd, J = 12.8, 7.7 Hz, 1H), 7.66 – 7.55 (m, 3H), 7.51 – 7.46 (m, 1H), 7.24 – 7.17 (m, 2H), 7.16 – 7.02 (m, 10H), 6.97 (td, J = 7.5, 3.3 Hz, 1H), 6.90 – 6.83 (m, 2H), 6.79 (td, J = 7.6, 3.1 Hz, 1H), 3.22 (s, 3H), 2.18 (s, 3H), 1.90 (s, 3H). **^{13}C NMR** (101 MHz, $CDCl_3$): δ 138.05 (d, J_{CP} = 12.0 Hz), 137.34 (d, J_{CP} = 12.2 Hz), 136.57, 135.65 (d, J_{CP} = 8.4 Hz), 134.91, 134.81 (d, J_{CP} = 9.8 Hz), 134.66 (d, J_{CP} = 3.8 Hz), 134.64 (d, J_{CP} = 100.4 Hz), 134.35 (d, J_{CP} = 9.6 Hz), 132.92 (d, J_{CP} = 104.7 Hz), 132.12 (d, J_{CP} = 2.7 Hz), 132.04 (d, J_{CP} = 9.3 Hz), 131.71 (d, J_{CP} = 12.5 Hz), 131.68, 131.23 (d, J_{CP} = 2.6 Hz), 130.58 (d, J_{CP} = 104.6 Hz), 129.04, 128.81 (d, J_{CP} = 11.4 Hz), 128.72, 128.61, 128.50, 128.01, 127.85 (d, J_{CP} = 13.0 Hz), 127.15 (d, J_{CP} = 12.9 Hz), 126.44, 125.24, 122.09, 119.85, 119.55, 115.49, 109.44, 30.70, 21.42, 21.02. **^{31}P NMR** (162 MHz, $CDCl_3$): δ 29.3. **HRMS** (ESI) calcd for $C_{35}H_{30}NNaOP$ $(M+Na)^+$: 534.1957, found: 534.1955.

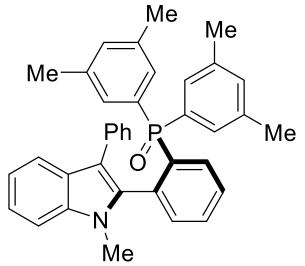

bis(3,5-dimethylphenyl)(2-(1-methyl-3-phenyl-1*H*-indol-2-yl)phenyl)phosphine oxide (d 13). White solid. 23 mg, 43% yield. **HPLC** conditions: Daicel CHIRALCEL® OD-H column with hexane/2-propanol = 97/03, flow = 1.0 mL/min, λ = 254 nm. Retention times: 12.0 min [(*R*)-enantiomer], 14.5 min [(*S*)-enantiomer]. $[\alpha]_D$ ^{25}: 0.0 (*c* 0.100, CH_2Cl_2, 90% ee). The

absolute configuration was assigned by analogy with compound (*R*)-**d1**. **^{1}H NMR** (400 MHz, $CDCl_3$): δ 7.91 (dd, *J* = 12.8, 7.6 Hz, 1H), 7.69 – 7.51 (m, 3H), 7.43 (dd, *J* = 6.7, 4.9 Hz, 1H), 7.20 (t, *J* = 7.5 Hz, 1H), 7.16 – 7.03 (m, 7H), 7.02 – 6.95 (m, 3H), 6.92 (d, *J* = 12.4 Hz, 2H), 6.65 (s, 1H), 3.29 (s, 3H), 2.15 (s, 6H), 1.89 (s, 6H). **^{13}C NMR** (101 MHz, $CDCl_3$): δ 137.74 (d, J_{CP} = 12.8 Hz), 137.15 (d, J_{CP} = 13.0 Hz), 136.72, 135.70 (d, J_{CP} = 8.5 Hz), 134.84 (d, J_{CP} = 100.3 Hz), 134.81 (d, J_{CP} = 9.8 Hz), 134.81, 134.15 (d, J_{CP} = 9.5 Hz), 133.22 (d, J_{CP} = 2.9 Hz), 132.97 (d, J_{CP} = 104.5 Hz), 132.70 (d, J_{CP} = 2.8 Hz), 131.25 (d, J_{CP} = 2.3 Hz), 130.31 (d, J_{CP} = 103.9 Hz), 129.29 (d, J_{CP} = 9.7 Hz), 128.95, 128.86, 128.66 (d, J_{CP} = 11.6 Hz), 127.78, 126.53, 125.19, 122.08, 119.83, 119.61, 115.31, 109.45, 30.90, 21.36, 20.93. **^{31}P NMR** (162 MHz, $CDCl_3$): δ 28.9. **HRMS** (ESI) calcd for $C_{37}H_{35}NOP$ $(M+H)^+$: 540.2451, found: 540.2448.

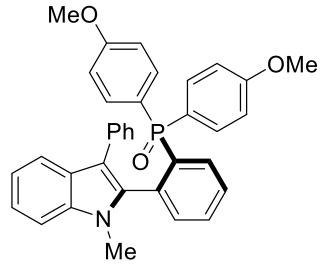

bis(4-methoxyphenyl)(2-(1-methyl-3-phenyl-1*H*-indol-2-yl)phenyl)phosphine oxide (d14). White solid. 22 mg, 40% yield. **HPLC** conditions: Daicel CHIRALCEL® OD-H column with hexane/2-propanol = 85/15, flow = 1.0 mL/min, λ = 254 nm. Retention times: 9.8 min [(*R*)-enantiomer], 13.2 min [(*S*)-enantiomer]. $[\alpha]_D{}^{25}$: 0.0 (*c* 0.100, CH_2Cl_2, 86% ee). The absolute configuration was assigned by analogy with compound (*R*)-**d1**. **^{1}H NMR** (400 MHz, $CDCl_3$): δ 8.11 (ddd, *J* = 12.6, 6.9, 2.3 Hz, 1H), 7.68 – 7.57 (m, 3H), 7.50 (ddd, *J* = 7.0, 4.4, 1.8 Hz, 1H), 7.21 – 7.15 (m, 1H), 7.15 – 7.05 (m, 8H), 7.03 (d, *J* = 8.0 Hz, 1H), 6.98 (dd, *J* = 11.8, 8.7 Hz, 2H), 6.53 (dd, *J* = 8.7, 1.9 Hz, 2H), 6.31 (dd, *J* = 8.7, 2.1 Hz, 2H), 3.73 (s, 3H), 3.54 (s, 3H), 3.08 (s, 3H). **^{13}C NMR** (101 MHz, $CDCl_3$): δ 161.70 (d, J_{CP} = 2.6 Hz), 161.57 (d, J_{CP} = 3.1 Hz), 136.49, 135.15 (d, J_{CP} = 8.6 Hz), 135.09, 134.92 (d, J_{CP} = 101.1 Hz), 134.79 (d, J_{CP} = 8.8 Hz), 134.37 (d, J_{CP} = 9.5 Hz), 133.30 (d, J_{CP} = 11.7 Hz), 133.23 (d, J_{CP} = 11.8 Hz), 131.07 (d, J_{CP} = 2.3 Hz), 129.05 (d, J_{CP} = 10.6 Hz), 129.02, 128.20, 126.41, 125.26, 124.27 (d, J_{CP} = 111.7 Hz), 121.99, 121.93 (d, J_{CP} = 113.1 Hz), 119.79, 119.36, 115.28, 113.57 (d, J_{CP} = 13.3 Hz), 112.72 (d, J_{CP} = 13.5 Hz), 109.25, 55.13, 54.92, 30.43. **^{31}P NMR** (162 MHz, $CDCl_3$): δ 29.4. **HRMS** (ESI) calcd for $C_{35}H_{30}NNaO_3P$ $(M+Na)^+$: 566.1856, found: 566.1851.

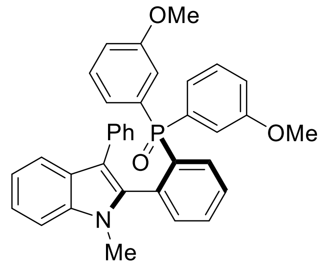

bis(3-methoxyphenyl)(2-(1-methyl-3-phenyl-1*H*-indol-2-yl)phenyl)phosphine oxide (d15). White solid. 29 mg, 53% yield. **HPLC** conditions: Daicel CHIRALCEL® OD-H column with

hexane/2-propanol = 90/10, flow = 1.0 mL/min, λ = 254 nm. Retention times: 9.4 min [(*R*)-enantiomer], 11.0 min [(*S*)-enantiomer]. $[\alpha]_D$ ^{25}: - 116 (*c* 0.100, CH_2Cl_2, 85% ee). The absolute configuration was assigned by analogy with compound (*R*)-**d1**. **^{1}H NMR** (400 MHz, $CDCl_3$): δ 7.88 (ddd, *J* = 12.9, 7.7, 1.5 Hz, 1H), 7.61 (d, *J* = 7.7 Hz, 2H), 7.58 – 7.47 (m, 2H), 7.24 – 7.17 (m, 1H), 7.15 – 7.03 (m, 7H), 7.03 – 6.95 (m, 2H), 6.89 – 6.74 (m, 4H), 6.67 – 6.53 (m, 2H), 3.64 (s, 3H), 3.47 (s, 3H), 3.27 (s, 3H). **^{13}C NMR** (101 MHz, $CDCl_3$): δ 159.20 (d, J_{CP} = 14.9 Hz), 158.56 (d, J_{CP} = 15.6 Hz), 136.55, 135.85 (d, J_{CP} = 8.5 Hz), 134.91, 134.64 (d, J_{CP} = 10.0 Hz), 134.46 (d, J_{CP} = 9.5 Hz), 134.31 (d, J_{CP} = 101.3 Hz), 134.20 (d, J_{CP} = 105.1 Hz), 132.01 (d, J_{CP} = 105.2 Hz), 131.33 (d, J_{CP} = 2.3 Hz), 129.22 (d, J_{CP} = 14.8 Hz), 129.05, 128.83 (d, J_{CP} = 11.7 Hz), 128.53 (d, J_{CP} = 14.5 Hz), 128.04, 126.37, 125.16, 123.97 (d, J_{CP} = 10.3 Hz), 123.58 (d, J_{CP} = 9.8 Hz), 122.04, 119.80, 119.54, 117.62 (d, J_{CP} = 2.7 Hz), 116.70 (d, J_{CP} = 2.5 Hz), 116.43 (d, J_{CP} = 11.3 Hz), 116.23 (d, J_{CP} = 10.5 Hz), 115.62, 109.43, 55.20, 54.95. **^{31}P NMR** (162 MHz, $CDCl_3$): δ 29.3. **HRMS** (ESI) calcd for $C_{35}H_{30}NNaO_3P$ $(M+Na)^+$: 566.1856, found: 566.1847.

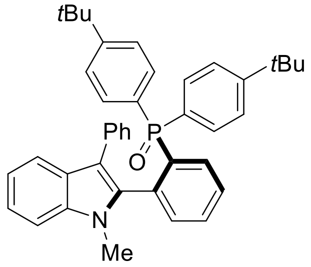

bis(4-tertbutylphenyl)(2-(1-methyl-3-phenyl-1*H*-indol-2-yl)phenyl)phosphine oxide (d16). White solid. 26 mg, 43% yield. **HPLC** conditions: Daicel CHIRALCEL® OD-H column with hexane/2-propanol = 90/10, flow = 1.0 mL/min, λ = 254 nm. Retention times: 5.2 min [(*R*)-enantiomer], 7.1 min [(*S*)-enantiomer]. $[\alpha]_D$ ^{25}: - 131 (*c* 0.100, CH_2Cl_2, 83% ee). The absolute configuration was assigned by analogy with compound (*R*)-**d1**. **^{1}H NMR** (400 MHz, $CDCl_3$): δ 8.22 – 8.16 (m, 1H), 7.69 – 7.63 (m, 2H), 7.62 (d, *J* = 8.0 Hz, 1H), 7.52 – 7.47 (m, 1H), 7.20 – 7.14 (m, 3H), 7.14 – 7.08 (m, 6H), 7.08 – 7.02 (m, 4H), 7.01 – 6.97 (m, 2H), 6.88 (dd, *J* = 8.4, 2.7 Hz, 2H), 3.03 (s, 3H), 1.25 (s, 9H), 1.14 (s, 9H). **^{13}C NMR** (101 MHz, $CDCl_3$): δ 154.22 (d, J_{CP} = 2.6 Hz), 154.19 (d, J_{CP} = 2.3 Hz), 136.38, 135.32 (d, J_{CP} = 8.6 Hz), 135.03, 134.94, 134.66 (d, J_{CP} = 3.8 Hz), 134.51 (d, J_{CP} = 100.4 Hz), 134.40 (d, J_{CP} = 9.5 Hz), 131.55 (d, J_{CP} = 10.4 Hz), 131.27 (d, J_{CP} = 10.4 Hz), 131.14 (d, J_{CP} = 2.6 Hz), 129.63 (d, J_{CP} = 108.5 Hz), 129.07, 128.96 (d, J_{CP} = 11.1 Hz), 128.16, 127.53 (d, J_{CP} = 108.2 Hz), 126.39, 125.24, 125.07 (d, J_{CP} = 12.5 Hz), 124.38 (d, J_{CP} = 12.6 Hz), 122.13, 119.87, 119.47, 115.50, 109.53, 34.76, 34.64, 31.05, 31.01, 30.33. **^{31}P NMR** (162 MHz, $CDCl_3$): δ 29.9. **HRMS** (ESI) calcd for $C_{41}H_{42}NNaOP$ $(M+Na)^+$: 618.2896, found: 618.2894.

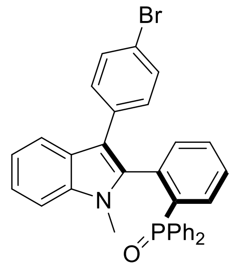

(2-(3-(4-bromophenyl)-1-methyl-1*H*-indol-2-yl)phenyl)diphenylphosphine oxide (d17). Known compound.[5] White solid. 31 mg, 55% yield. **HPLC** conditions: Daicel CHIRALCEL® OD-H column with hexane/2-propanol = 90/10, flow = 1.0 mL/min, λ = 254 nm. Retention times: 7.6 min [(*R*)-enantiomer], 9.8 min [(*S*)-enantiomer]. $[\alpha]_D^{25}$: - 93 (*c* 0.100, CH_2Cl_2, 82% ee). The absolute configuration was assigned by analogy with compound (*R*)-**d1**. **^{1}H NMR** (400 MHz, $CDCl_3$): δ 7.89 (dd, *J* = 12.6, 7.8 Hz, 1H), 7.68 (t, *J* = 7.6 Hz, 1H), 7.61 (d, *J* = 7.6 Hz, 1H), 7.57 – 7.51 (m, 2H), 7.42 – 7.36 (m, 1H), 7.25 – 7.06 (m, 12H), 6.92 (td, *J* = 7.7, 3.0 Hz, 2H), 6.86 (d, *J* = 8.3 Hz, 2H), 3.20 (s, 3H), 1.71 (s, 3H).

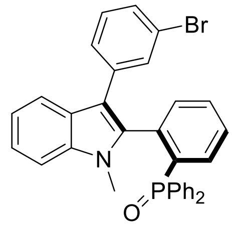

(2-(3-(3-bromophenyl)-1-methyl-1*H*-indol-2-yl)phenyl)diphenylphosphine oxide (d18). White solid. 39 mg, 69% yield. **HPLC** conditions: Daicel CHIRALCEL® OD-H column with hexane/2-propanol = 90/10, flow = 1.0 mL/min, λ = 254 nm. Retention times: 8.0 min [(*R*)-enantiomer], 10.0 min [(*S*)-enantiomer]. $[\alpha]_D^{25}$: 109 (*c* 0.100, CH_2Cl_2, 70% ee). The absolute configuration was assigned by analogy with compound (*R*)-**d1**. **^{1}H NMR** (400 MHz, $CDCl_3$): δ 7.91 (ddd, *J* = 12.7, 7.7, 1.4 Hz, 1H), 7.69 (tt, *J* = 7.5, 1.6 Hz, 1H), 7.61 (tt, *J* = 7.6, 1.8 Hz, 1H), 7.58 (d, *J* = 7.9 Hz, 1H), 7.53 (ddd, *J* = 7.5, 4.1, 1.4 Hz, 1H), 7.44 – 7.36 (m, 1H), 7.25 – 7.07 (m, 12H), 6.96 – 6.85 (m, 4H), 3.19 (s, 3H). **^{13}C NMR** (101 MHz, $CDCl_3$): δ 137.36, 136.63, 135.67 (d, J_{CP} = 8.2 Hz), 135.25 (d, J_{CP} = 3.4 Hz), 134.90 (d, J_{CP} = 9.8 Hz), 134.54 (d, J_{CP} = 9.2 Hz), 134.29 (d, J_{CP} = 100.4 Hz), 132.84 (d, J_{CP} = 105.2 Hz), 131.66, 131.65 (d, J_{CP} = 10.0 Hz), 131.51 (d, J_{CP} = 2.5 Hz), 131.37 (d, J_{CP} = 10.0 Hz), 131.23 (d, J_{CP} = 3.0 Hz), 130.48 (d, J_{CP} = 105.3 Hz), 129.64, 129.14 (d, J_{CP} = 11.5 Hz), 128.22 (d, J_{CP} = 12.2 Hz), 128.12, 127.50 (d, J_{CP} = 12.4 Hz), 127.33, 126.08, 122.44, 120.31, 119.22, 113.94, 109.88, 30.68. **^{31}P NMR** (162 MHz, $CDCl_3$): δ 29.1. **HRMS** (ESI) calcd for $C_{33}H_{25}BrNNaOP$ $(M+Na)^+$: 584.0749, found: 584.0741.

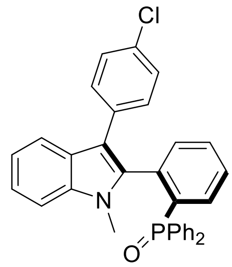

(2-(3-(4-chlorophenyl)-1-methyl-1*H*-indol-2-yl)phenyl)diphenylphosphine oxide (d19). Known compound.[5] White solid. 29 mg, 57% yield. **HPLC** conditions: Daicel CHIRALCEL® OD-H column with hexane/2-propanol = 90/10, flow = 1.0 mL/min, λ = 254 nm. Retention times: 7.5 min [(*R*)-enantiomer], 9.5 min [(*S*)-enantiomer]. $[\alpha]_D^{25}$: - 102 (*c* 0.100, CH_2Cl_2, 84% ee). The absolute configuration was assigned by analogy with compound (*R*)-**d1**. **^{1}H NMR** (400 MHz, $CDCl_3$): δ 7.91 (ddd, *J* = 12.8, 7.8, 1.4 Hz, 1H), 7.69 (tt, *J* = 7.5, 1.5 Hz, 1H), 7.61 (tt, *J* = 7.7, 1.7 Hz, 1H), 7.57 – 7.52 (m, 2H), 7.43 – 7.35 (m, 1H), 7.22 (ddd, *J* = 8.1, 6.8, 1.1 Hz, 1H), 7.20 – 7.07 (m, 9H), 7.04 – 6.99 (m, 2H), 6.95 – 6.88 (m, 4H), 3.19 (s, 3H).

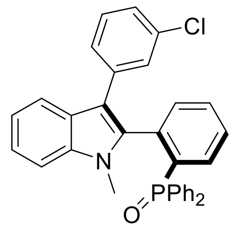

(2-(3-(3-chlorophenyl)-1-methyl-1*H*-indol-2-yl)phenyl)diphenylphosphine oxide (d20). White solid. 34 mg, 65% yield. **HPLC** conditions: Daicel CHIRALCEL® OD-H column with hexane/2-propanol = 90/10, flow = 1.0 mL/min, λ = 254 nm. Retention times: 7.8 min [(*R*)-enantiomer], 9.9 min [(*S*)-enantiomer]. $[\alpha]_D^{25}$: 1 (*c* 0.100, CH_2Cl_2, 75% ee). The absolute configuration was assigned by analogy with compound (*R*)-**d1**. **^{1}H NMR** (400 MHz, $CDCl_3$): δ 7.89 (dd, *J* = 12.8, 7.7 Hz, 1H), 7.69 (t, *J* = 7.5 Hz, 1H), 7.63 – 7.52 (m, 3H), 7.39 (t, *J* = 7.3 Hz, 1H), 7.25 – 7.09 (m, 10H), 7.04 (d, *J* = 7.8 Hz, 1H), 6.99 (t, *J* = 7.7 Hz, 1H), 6.95 – 6.85 (m, 4H), 3.20 (s, 3H). **^{13}C NMR** (101 MHz, $CDCl_3$): δ 136.93, 136.51, 135.58, 135.19 (d, J_{CP} = 95.9 Hz), 135.09, 134.81, 134.44 (d, J_{CP} = 9.4 Hz), 133.86, 132.73 (d, J_{CP} = 105.0 Hz), 131.54 (d, J_{CP} = 9.7 Hz), 131.52, 131.39 (d, J_{CP} = 2.2 Hz), 131.26 (d, J_{CP} = 10.2 Hz), 131.11 (d, J_{CP} = 3.3 Hz), 130.33 (d, J_{CP} = 106.8 Hz), 129.19, 129.00 (d, J_{CP} = 11.5 Hz), 128.68, 128.10 (d, J_{CP} = 12.3 Hz), 127.41 (d, J_{CP} = 12.3 Hz), 126.82, 126.00, 125.15, 122.31, 120.17, 119.13, 113.95, 109.74, 30.57. **^{31}P NMR** (162 MHz, $CDCl_3$): δ 29.2. **HRMS** (ESI) calcd for $C_{33}H_{25}ClNNaOP$ $(M+Na)^+$: 540.1254, found: 540.1256.

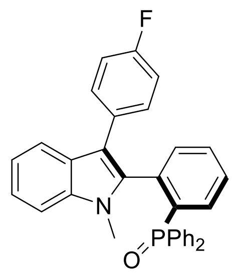

(2-(3-(4-fluorophenyl)-1-methyl-1*H*-indol-2-yl)phenyl)diphenylphosphine oxide (d21). Known compound.[5] White solid. 33 mg, 66% yield. **HPLC** conditions: Daicel CHIRALPAK® IC column with hexane/2-propanol = 80/20, flow = 1.0 mL/min, λ = 254 nm. Retention times: 21.6 min [(*R*)-enantiomer], 27.7 min [(*S*)-enantiomer]. $[\alpha]_D^{25}$: - 228 (*c* 0.100, CH_2Cl_2, 86% ee). The absolute configuration was assigned by analogy with compound (*R*)-**d1**. ^{1}**H NMR** (400 MHz, $CDCl_3$): δ 7.91 (dd, *J* = 12.8, 7.7 Hz, 1H), 7.67 (t, *J* = 7.5 Hz, 1H), 7.59 (t, *J* = 7.6 Hz, 1H), 7.56 – 7.49 (m, 2H), 7.36 (t, *J* = 7.0 Hz, 1H), 7.23 – 7.06 (m, 10H), 6.97 (dd, *J* = 8.5, 5.6 Hz, 2H), 6.92 (td, *J* = 7.6, 2.9 Hz, 2H), 6.76 (t, *J* = 8.7 Hz, 2H), 3.18 (s, 3H).

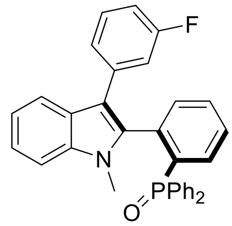

(2-(3-(3-fluorophenyl)-1-methyl-1*H*-indol-2-yl)phenyl)diphenylphosphine oxide (d22). White solid. 32 mg, 63% yield. **HPLC** conditions: Daicel CHIRALCEL® OD-H column with hexane/2-propanol = 90/10, flow = 1.0 mL/min, λ = 254 nm. Retention times: 7.8 min [(*R*)-enantiomer], 10.1 min [(*S*)-enantiomer]. $[\alpha]_D^{25}$: - 1 (*c* 0.100, CH_2Cl_2, 86% ee). The absolute configuration was assigned by analogy with compound (*R*)-**d1**. ^{1}**H NMR** (400 MHz, $CDCl_3$): δ 7.90 (dd, *J* = 12.8, 7.7 Hz, 1H), 7.69 (t, *J* = 7.5 Hz, 1H), 7.64 – 7.57 (m, 2H), 7.54 (dd, *J* = 7.9, 3.9 Hz, 1H), 7.37 (t, *J* = 7.0 Hz, 1H), 7.25 – 7.08 (m, 10H), 7.03 (td, *J* = 8.0, 6.3 Hz, 1H), 6.91 (td, *J* = 7.7, 2.9 Hz, 2H), 6.82 – 6.74 (m, 2H), 6.66 (dt, *J* = 10.8, 2.1 Hz, 1H), 3.20 (s, 3H). ^{13}**C NMR** (101 MHz, $CDCl_3$): δ 162.64 (d, J_{CF} = 244.1 Hz), 137.34 (d, J_{CP} = 8.5 Hz), 136.51, 135.66 (d, J_{CF} = 8.1 Hz), 135.06 (d, J_{CP} = 3.7 Hz), 134.76 (d, J_{CP} = 9.9 Hz), 134.41 (d, J_{CP} = 9.5 Hz), 134.17 (d, J_{CP} = 100.5 Hz), 132.85 (d, J_{CP} = 105.2 Hz), 131.54 (d, J_{CP} = 9.9 Hz), 131.41 (d, J_{CP} = 2.7 Hz), 131.28 (d, J_{CP} = 10.0 Hz), 131.01 (d, J_{CP} = 2.8 Hz), 130.43 (d, J_{CP} = 105.2 Hz), 129.30 (d, J_{CF} = 8.7 Hz), 129.00 (d, J_{CP} = 11.6 Hz), 128.14 (d, J_{CP} = 12.2 Hz), 127.43 (d, J_{CP} = 12.3 Hz), 126.07, 124.51 (d, J_{CF} = 2.6 Hz), 122.28, 120.13, 119.19, 115.48 (d, J_{CF} = 21.5 Hz), 114.21 (d, J_{CF} = 2.3 Hz), 111.92 (d, J_{CF} = 21.1 Hz), 109.71, 30.58. ^{31}**P NMR** (162 MHz, $CDCl_3$): δ 29.2. **HRMS** (ESI) calcd for $C_{33}H_{25}FNNaOP$ $(M+Na)^+$: 524.1550, found: 524.1545.

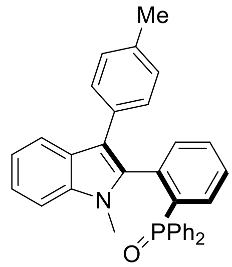

(2-(3-(4-methylphenyl)-1-methyl-1*H*-indol-2-yl)phenyl)diphenylphosphine oxide (d23). Known compound.[5] White solid. 21 mg, 42% yield. **HPLC** conditions: Daicel CHIRALCEL® OD-H column with hexane/2-propanol = 95/05, flow = 1.0 mL/min, λ = 254 nm. Retention times: 15.1 min [(*R*)-enantiomer], 21.4 min [(*S*)-enantiomer]. $[\alpha]_D^{25}$: 61 (*c* 0.100, CH_2Cl_2, 84% ee). The absolute configuration was assigned by analogy with compound (*R*)-**d1**. **^{1}H NMR** (400 MHz, $CDCl_3$): δ 8.04 (dd, *J* = 12.5, 7.9 Hz, 1H), 7.70 – 7.57 (m, 3H), 7.55 – 7.49 (m, 1H), 7.32 (td, *J* = 7.4, 1.8 Hz, 1H), 7.23 – 6.99 (m, 10H), 6.99 – 6.90 (m, 4H), 6.84 (td, *J* = 7.7, 2.9 Hz, 2H), 3.12 (s, 3H), 2.34 (s, 3H).

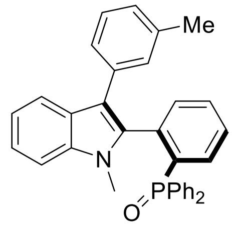

(2-(3-(3-methylphenyl)-1-methyl-1*H*-indol-2-yl)phenyl)diphenylphosphine oxide (d24). Known compound.[5] White solid. 30 mg, 60% yield. **HPLC** conditions: Daicel CHIRALCEL® OD-H column with hexane/2-propanol = 90/10, flow = 1.0 mL/min, λ = 254 nm. Retention times: 7.8 min [(*R*)-enantiomer], 9.5 min [(*S*)-enantiomer]. $[\alpha]_D^{25}$: - 133 (*c* 0.100, CH_2Cl_2, 81% ee). The absolute configuration was assigned by analogy with compound (*R*)-**d1**. **^{1}H NMR** (400 MHz, $CDCl_3$): δ 8.04 (dd, *J* = 12.7, 7.6 Hz, 1H), 7.67 (t, *J* = 7.5 Hz, 1H), 7.61 (t, *J* = 7.8 Hz, 1H), 7.60 (d, *J* = 7.9 Hz, 1H), 7.57 – 7.52 (m, 1H), 7.38 – 7.30 (m, 1H), 7.22 – 6.98 (m, 11H), 6.93 (d, *J* = 7.6 Hz, 1H), 6.89 – 6.81 (m, 4H), 3.11 (s, 3H), 2.19 (s, 3H).

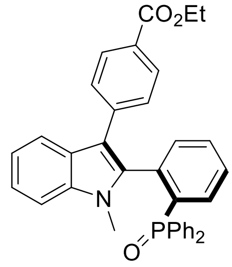

(2-(3-(4-ethoxycarbonylphenyl)-1-methyl-1*H*-indol-2-yl)phenyl)diphenylphosphine oxide (d25). Known compound.[5] White solid. 23 mg, 41% yield. **HPLC** conditions: Daicel CHIRALCEL® OD-H column with hexane/2-propanol = 85/15, flow = 1.0 mL/min, λ = 254 nm. Retention times: 6.9 min [(*R*)-enantiomer], 8.5 min [(*S*)-enantiomer]. $[\alpha]_D^{25}$: 78 (*c* 0.100, CH_2Cl_2, 87% ee). The absolute configuration was assigned by analogy with compound (*R*)-**d1**.

^{1}**H NMR** (400 MHz, $CDCl_3$): δ 7.85 (dd, *J* = 12.8, 7.7 Hz, 1H), 7.73 (d, *J* = 8.1 Hz, 2H), 7.70 – 7.65 (m, 1H), 7.64 – 7.56 (m, 2H), 7.53 (dd, *J* = 7.8, 3.8 Hz, 1H), 7.36 (t, *J* = 7.3 Hz, 1H), 7.26 – 7.09 (m, 10H), 7.07 (d, *J* = 8.1 Hz, 2H), 6.88 (td, *J* = 7.7, 2.9 Hz, 2H), 4.39 (q, *J* = 7.1 Hz, 2H), 3.26 (s, 3H), 1.41 (t, *J* = 7.1 Hz, 3H).

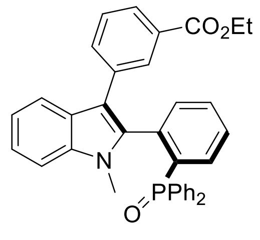

(2-(3-(3-ethoxycarbonylphenyl)-1-methyl-1*H*-indol-2-yl)phenyl)diphenylphosphine oxide (d26). Known compound.[5] White solid. 28 mg, 50% yield. **HPLC** conditions: Daicel CHIRALCEL® OD-H column with hexane/2-propanol = 85/15, flow = 1.0 mL/min, λ = 254 nm. Retention times: 6.7 min [(*R*)-enantiomer], 8.4 min [(*S*)-enantiomer]. $[\alpha]_D{}^{25}$: 66 (*c* 0.100, CH_2Cl_2, 71% ee). The absolute configuration was assigned by analogy with compound (*R*)-**d1**. ^{1}**H NMR** (400 MHz, $CDCl_3$): δ 7.89 (dd, *J* = 12.9, 7.7 Hz, 1H), 7.76 (dd, *J* = 7.7, 1.5 Hz, 1H), 7.72 – 7.67 (m, 2H), 7.64 – 7.54 (m, 3H), 7.33 (t, *J* = 7.2 Hz, 1H), 7.25 – 7.09 (m, 10H), 7.06 (t, *J* = 7.4 Hz, 1H), 6.85 (td, *J* = 7.8, 3.0 Hz, 2H), 4.34 (q, *J* = 7.2 Hz, 2H), 3.24 (s, 3H), 1.38 (t, *J* = 7.2 Hz, 3H).

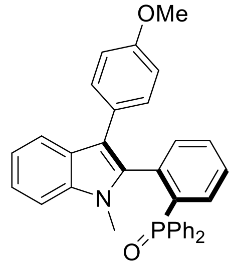

(2-(3-(4-methoxyphenyl)-1-methyl-1*H*-indol-2-yl)phenyl)diphenylphosphine oxide (d27). Known compound.[5] White solid. 27 mg, 52% yield. **HPLC** conditions: Daicel CHIRALCEL® OD-H column with hexane/2-propanol = 93/07, flow = 1.0 mL/min, λ = 254 nm. Retention times: 16.1 min [(*R*)-enantiomer], 19.6 min [(*S*)-enantiomer]. $[\alpha]_D{}^{25}$: 80 (*c* 0.100, CH_2Cl_2, 78% ee). The absolute configuration was assigned by analogy with compound (*R*)-**d1**. ^{1}**H NMR** (400 MHz, $CDCl_3$): δ 8.02 (dd, *J* = 12.7, 7.6 Hz, 1H), 7.67 (t, *J* = 7.5 Hz, 1H), 7.61 (t, *J* = 7.8 Hz, 1H), 7.58 – 7.50 (m, 2H), 7.33 (t, *J* = 7.2 Hz, 1H), 7.22 – 7.01 (m, 10H), 6.98 (d, *J* = 8.3 Hz, 2H), 6.87 (td, *J* = 8.0, 2.5 Hz, 2H), 6.67 (d, *J* = 8.3 Hz, 2H), 3.81 (s, 3H), 3.12 (s, 3H).

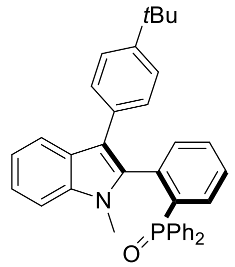

(2-(3-(4-tertbutylphenyl)-1-methyl-1*H*-indol-2-yl)phenyl)diphenylphosphine oxide (d28). Known compound.[5] White solid. 40 mg, 74% yield. **HPLC** conditions: Daicel CHIRALCEL® OD-H column with hexane/2-propanol = 90/10, flow = 1.0 mL/min, λ = 254 nm. Retention times: 6.1 min [(*R*)-enantiomer], 7.5 min [(*S*)-enantiomer]. $[\alpha]_D{}^{25}$: 124 (*c* 0.100, CH_2Cl_2, 77% ee). The absolute configuration was assigned by analogy with compound (*R*)-**d1**. **^{1}H NMR** (400 MHz, $CDCl_3$): δ 8.07 (ddd, *J* = 12.7, 7.6, 1.6 Hz, 1H), 7.71 – 7.63 (m, 2H), 7.62 (d, *J* = 8.1 Hz, 1H), 7.58 – 7.54 (m, 1H), 7.30 (td, *J* = 7.3, 1.5 Hz, 1H), 7.20 – 7.11 (m, 7H), 7.10 – 6.99 (m, 7H), 6.83 (td, *J* = 7.8, 3.0 Hz, 2H), 3.08 (s, 3H), 1.35 (s, 9H).

Scale-up for Copper-Bisoxazoline Catalyzed *ortho*-Arylation of Phosphonic Diamide: To a dried Schlenk tube was charged with NaOTf/MeOH solution (c: 3.4 mg in 20 mL MeOH, 3 mL, 0.003 mmol) and solvent was removed under vacuum. CuCl (3 mg, 0.03 mmol), **L6** (21 mg, 0.045 mmol) and CH_2Cl_2 (5 mL) were added into the tube and the mixture was stirred at rt for 0.5 h under N_2. Substrate **a6** (1.52g, 3 mmol, 1.0 equiv.), Ph_2ISbF_6 (2.33g, 4.5 mmol, 1.5 equiv.), 2,6-Di-*tert*-butylpyridine (1 mL, 4.5 mmol, 1.5 equiv.) and CH_2Cl_2 (25 mL) were added into the reactor under N_2. The reaction was heated to 60 °C and stirred for 72 h. After cooling to room temperature, the reaction mixture was filtered, concentrated under vacuum. The crude product was purified via flash chromatography on silica gel column (PE/EA 5:1 to 2:1, v/v) to afford product **b6** as off-white solid, 1.45 g, 83% yield.

Scale-up for Copper-Bisoxazoline Catalyzed C-3 Arylation of diphenylphosphine oxide indole: To a dried Schlenk tube was charged with NaOTf/MeOH solution (c: 6.8 mg in 20 mL MeOH, 7 mL, 0.03 mmol) and solvent was removed under vacuum. CuI (29 mg, 0.15 mmol), **L6** (103 mg, 0.45 mmol) and CH_2Cl_2 (5 mL) were added into the tube and the mixture was stirred at rt for 0.5 h under N_2. Substrate **c1** (1.22g, 3 mmol, 1.0 equiv.), Ph_2ISbF_6 (3.1g, 6 mmol, 2 equiv.), 2,6-Di-*tert*-butylpyridine (1.3 mL, 6 mmol, 2 equiv.) and CH_2Cl_2 (25 mL) were added into the reactor under N_2. The reaction was heated to 60 °C and stirred for 24 h. After cooling to room temperature, the reaction mixture was filtered, concentrated under vacuum. The crude product was purified via flash chromatography on silica gel column (PE/EA 5:1 to 2:1, v/v) to afford product **d1** as white solid, 0.9 g, 62% yield. Slurry the purified product (0.9 g) with EA (15 ml) at rt for 5-6 h, then filter the solution. After removing solvent from the mother liquor under vacuum, 0.5 g enantio-enriched product was obtained with 96% ee.

1.4 Attempt on Utilization of Diphenylphosphine Oxide

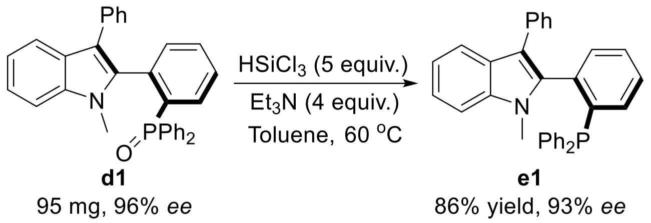

Following a reported procedure[6], reduction of diphenylphosphine oxide **d1** (95 mg, 96% ee) was performed in toluene at 60 °C with $HSiCl_3$ (5 equiv.) as reductant. The phosphine product **e1** was obtained in 86% yield with slight erosion on ee value (93% ee, determined by HPLC on Daicel CHIRALPAK IE column).

To demonstrate practical utility of the axial chiral phosphine product, **e1** was used as ligand in several palladium-catalyzed reactions, as showed in supplementary figure 7, but only disappointing results were observed in these reactions. More investigation would be needed to fulfill this purpose.

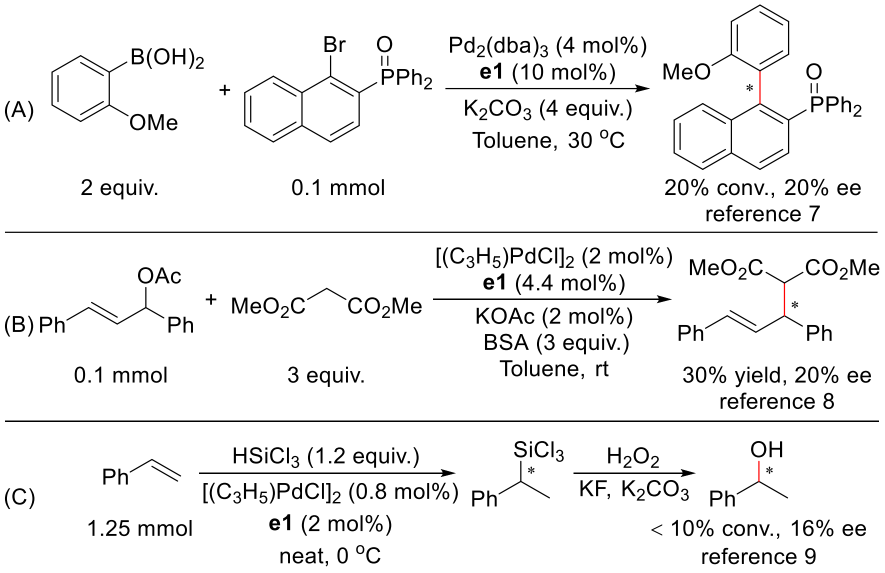

Supplementary Figure 7. Attempt on Utilization of Diphenylphosphine Oxide. **A** Palladium catalyzed suzuki-miyaura coupling. **B** Palladium catalyzed allylic substitution. **C** Palladium catalyzed olefin reduction.

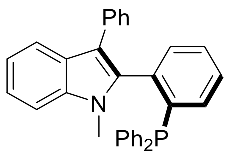

2-(2-(diphenylphosphanyl)phenyl)-1-methyl-3-phenyl-1*H*-indole (**e1**). White solid. 79 mg, 86% yield. **HPLC** conditions: Daicel CHIRALPAK IE column with hexane/2-propanol = 98/02, flow = 1.0 mL/min, λ = 254 nm. Retention times: 5.9 min [(*R*)-enantiomer], 6.4 min [(*S*)-enantiomer]. The absolute configuration was assigned by analogy with compound (*R*)-**d1**. **^{1}H NMR** (400 MHz, $CDCl_3$): δ 7.79 (d, J = 7.9 Hz, 1H), 7.44 – 7.42 (m, 2H), 7.39 – 7.34 (m,

1H), 7.32 (t, J = 8.5 Hz, 2H), 7.28 – 7.25 (m, 3H), 7.22 – 7.06 (m, 12H), 6.78 (t, J = 7.2 Hz, 2H), 3.31 (s, 3H). **^{13}C NMR** (101 MHz, $CDCl_3$): δ 140.34, 140.20, 138.42 (d, J_{CP} = 33.7 Hz), 137.50 (d, J_{CP} = 14.0 Hz), 137.01 (d, J_{CP} = 5.8 Hz), 136.88, 136.67 (d, J_{CP} = 13.4 Hz), 135.37, 134.54 (d, J_{CP} = 2.0 Hz), 133.88, 133.68, 133.31, 133.11, 132.57 (d, J_{CP} = 5.5 Hz), 129.51, 129.00, 128.80, 128.40 (d, J_{CP} = 9.5 Hz), 128.25 (d, J_{CP} = 8.5 Hz), 128.11 (d, J_{CP} = 5.6 Hz), 127.92, 126.75, 125.15, 121.87, 119.90, 119.79, 116.18 (d, J_{CP} = 2.8 Hz), 109.48, 30.49. **^{31}P NMR** (162 MHz, $CDCl_3$): δ - 13.8. **HRMS** (ESI) calcd for $C_{33}H_{26}NNaP$ $(M+Na)^+$: 490.1695, found: 490.1687.

1.5 Mechanistic Studies and Control Experiments

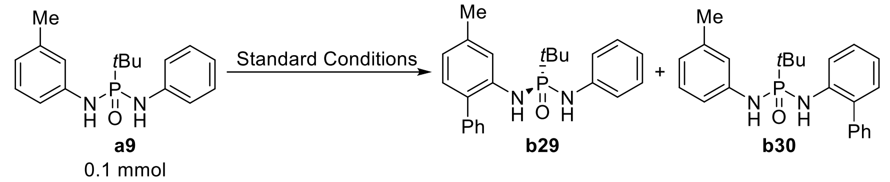

Intramolecular Competition Reaction: To a dried Schlenk tube was charged with NaOTf /MeOH solution (c: 3.4 mg in 20 mL MeOH, 0.5 mL) and solvent was removed under vacuum. CuCl (1 mg, 0.01 mmol), **L6** (6.9 mg, 0.015 mmol) and CH_2Cl_2 (2 mL) were added into the tube and the mixture was stirred at rt for 0.5 h under N_2. **a9** (0.1 mmol, 1.0 equiv.), diphenyliodonium hexafluoroantimonate salt (52 mg, 0.15 mmol, 1.5 equiv.) and 2,6-Di-*tert*-butylpyridine (33 μL, 0.15 mmol, 1.5 equiv.) were added into the mixture under N_2. The reaction was heated to 60 °C and stirred for 24 h. After cooling to room temperature, the reaction mixture was filtrated, concentrated under vacuum. The crude product was purified via flash chromatography (PE/EA 5:1 to 2:1, v/v) to afford a mixture. The ratio of **b29** : **b30** was determined to be 10:1 by ^{1}H NMR of crude product, as showed in supplementary figure 8.

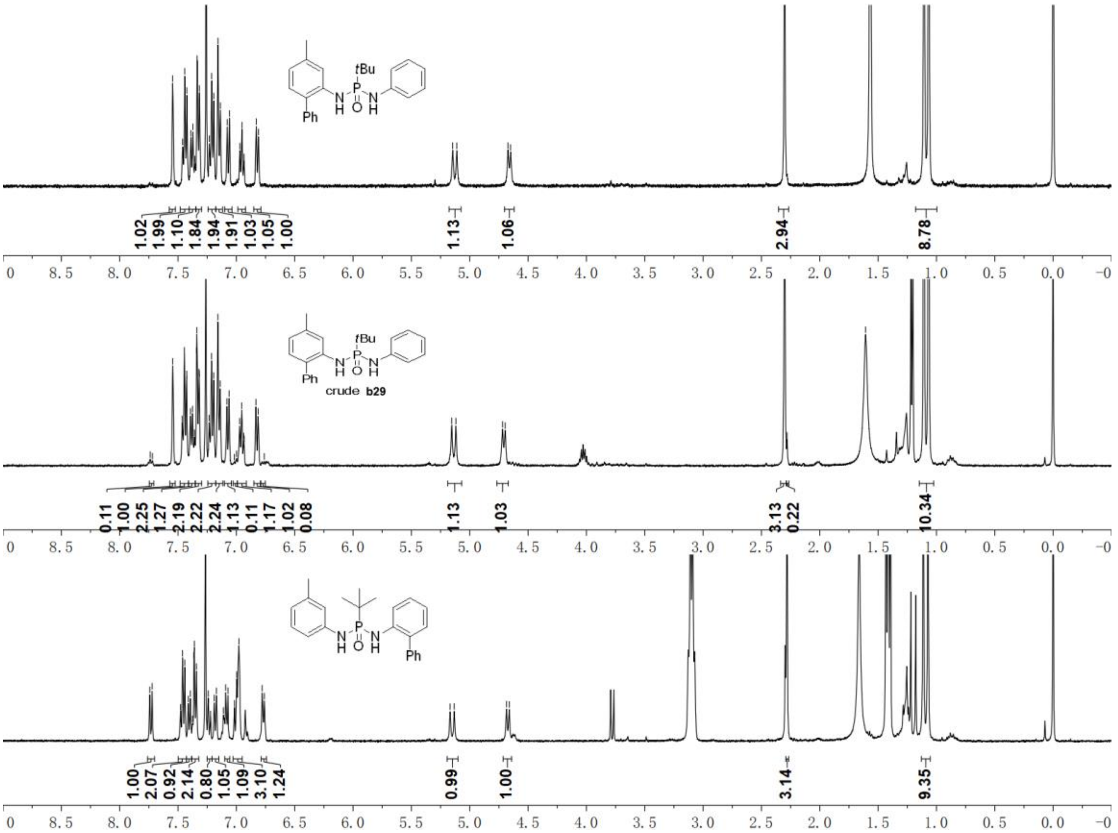

Supplementary Figure 8. ^{1}H NMR analysis of crude **b29/b30** mixture in $CDCl_3$

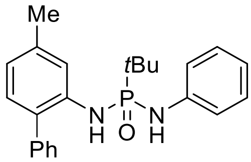

***P-tert*-butyl-*N*-phenyl-*N*'-[(3-methyl-6-phenyl)phenyl]phosphonic diamide (b29)**. White solid. 23 mg, 61% yield. **HPLC** conditions: Daicel CHIRALCEL® OD-H column with hexane/2-propanol = 95/05, flow = 1.0 mL/min, λ = 254 nm. Retention times: 14.8 min [(*R*)-enantiomer], 17.6 min [(*S*)-enantiomer]. The absolute configuration was assigned by analogy with compound (*R*)-**b18**. **^{1}H NMR** (400 MHz, $CDCl_3$): δ 7.55 (s, 1H), 7.44 (t, J = 7.4 Hz, 2H), 7.37 (t, J = 7.1 Hz, 1H), 7.33 (d, J = 7.8 Hz, 2H), 7.21 (t, J = 7.7 Hz, 2H), 7.15 (d, J = 8.0 Hz, 2H), 7.07 (d, J = 7.7 Hz, 1H), 6.95 (t, J = 7.2 Hz, 1H), 6.82 (d, J = 7.6 Hz, 1H), 5.13 (d, J = 14.2 Hz, 1H), 4.66 (d, J = 8.9 Hz, 1H), 2.30 (s, 3H), 1.09 (d, J = 16.1 Hz, 9H). **^{13}C NMR** (101 MHz, $CDCl_3$): δ 141.34, 138.91, 138.49, 137.14 (d, J_{CP} = 2.5 Hz), 129.79, 129.37, 129.17, 129.13, 128.05 (d, J_{CP} = 7.9 Hz), 127.73, 122.45, 121.86, 118.94 (d, J_{CP} = 5.0 Hz), 118.33 (d, J_{CP} = 2.6 Hz), 34.28 (d, J_{CP} = 114.5 Hz), 25.17, 21.42. **^{31}P NMR** (162 MHz, $CDCl_3$): δ 27.9. **HRMS** (ESI) calcd for $C_{23}H_{27}N_2NaOP$ $(M+Na)^+$: 401.1753, found: 401.1743.

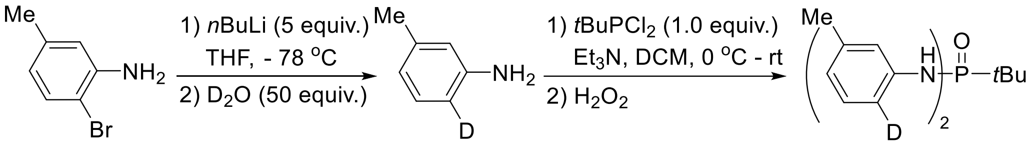

Deuterated-substrate preparation: 3-Toluidine-6-*d* was prepared according to the reported procedure in literature[10] and **a10** was obtained with approximately 75% enriched with deuterium at the 6-position, as determined by ^{1}H NMR (See supplementary figure 9).

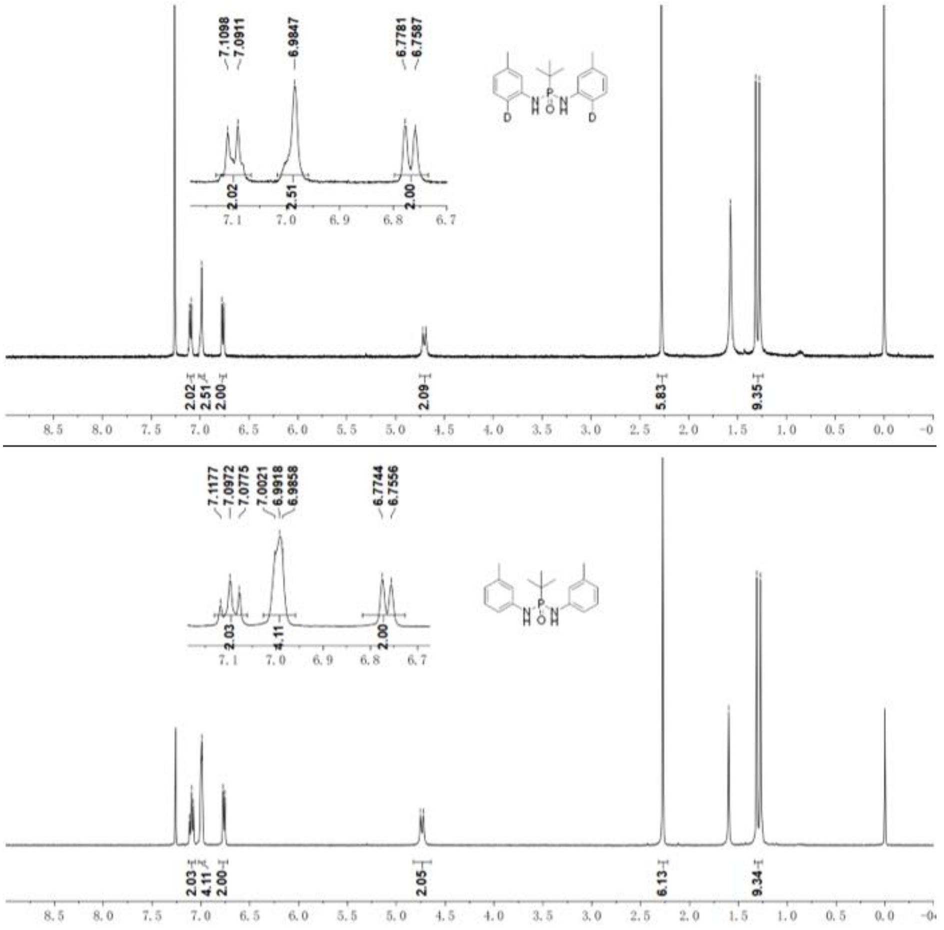

Supplementary Figure 9. ^{1}H NMR of **a10** and **a1** in $CDCl_3$

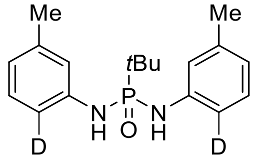

***P-tert*-butyl-*N, N*'-di(3-methyl-6-*d*-phenyl)phosphonic diamide (a10)**. White solid. 0.45 g, 70% yield (based on 2 mmol *t*BuPCl$_2$). **^{1}H NMR** (400 MHz, $CDCl_3$): δ 7.10 (d, J = 7.5 Hz, 2H), 6.98 (s, 2H), 6.77 (d, J = 7.5 Hz, 2H), 4.71 (d, J = 11.7 Hz, 2H), 2.28 (s, 6H), 1.30 (d, J = 16.0 Hz, 9H). **^{13}C NMR** (101 MHz, DMSO-d_6): δ 143.52, 137.92, 128.69, 121.17, 119.03 (d, J_{CP} = 6.0 Hz), 115.59 (d, J_{CP} = 5.3 Hz), 34.35 (d, J_{CP} = 114.4 Hz), 25.06, 21.71. **^{31}P NMR** (162 MHz, DMSO-d_6): δ 28.6. **HRMS** (ESI) calcd for $C_{18}H_{23}D_2N_2NaOP$ $(M+Na)^+$: 341.1722, found: 341.1713.

Preparation of materials for KIE experiments: A 1:1 mixture of **a1** and deuterated **a10** was prepared by careful blending the two molecules, and the exact ratio of **a1** and **a10** was determined by ^{1}H NMR, as showed in supplementary figure 10.

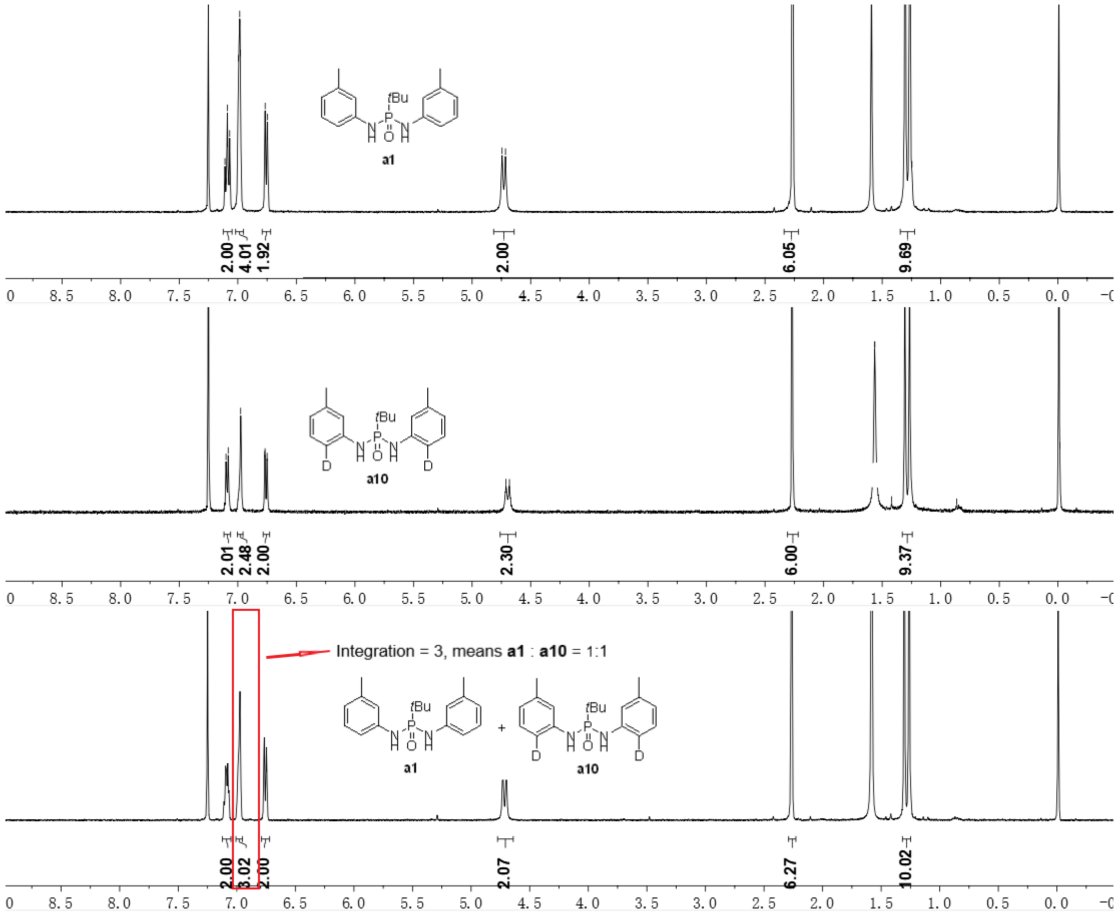

Supplementary Figure 10. ^{1}H NMR of 1:1 mixture of **a10** and **a1** in $CDCl_3$

General Procedure for Competition KIE Experiments: To a dried Schlenk tube was charged with NaOTf/MeOH solution (c: 3.4 mg in 20 mL MeOH, 0.5 mL) and solvent was removed under vacuum. CuCl (1 mg, 0.01 mmol), **L6** (6.9 mg, 0.015 mmol) and CH_2Cl_2 (2 mL) were added into the tube and the mixture was stirred at rt for 0.5 h under N_2. A 1:1 mixture of **a1** and **a10** (0.1 mmol, 1.0 equiv.), diphenyliodonium hexafluoroantimonate (0.15 mmol, 1.5 equiv.) and 2,6-Di-*tert*-butylpyridine (33 μL, 0.15 mmol, 1.5 equiv.) were added into the mixture under N_2. The reaction was heated to 60 °C and stirred for 1 h. The reaction was quenched by cooling it to – 40 °C. The product was isolated via flash chromatography (PE/EA 5:1 to 2:1, v/v) and the ratio of product **b1** and **b1**-*d* was determined to be 51:49 by ^{1}H NMR in supplementary figure 12. The kinetic isotope effect of this reaction was calculated: KIE = 51/49 ≈ 1.0.

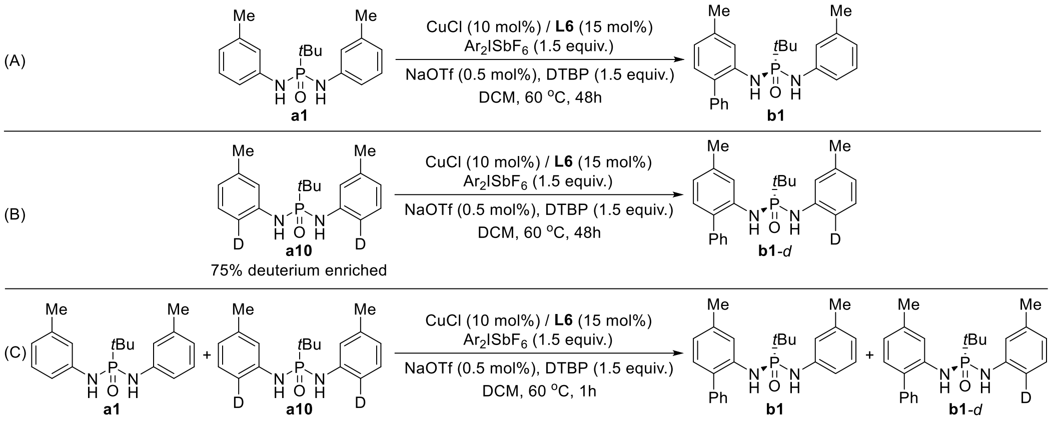

Supplementary Figure 11. Competition KIE Experiments. **A** Reaction of standard substrate. **B** Reaction of deuterated substrate. **C** Reaction of 1:1 mixture.

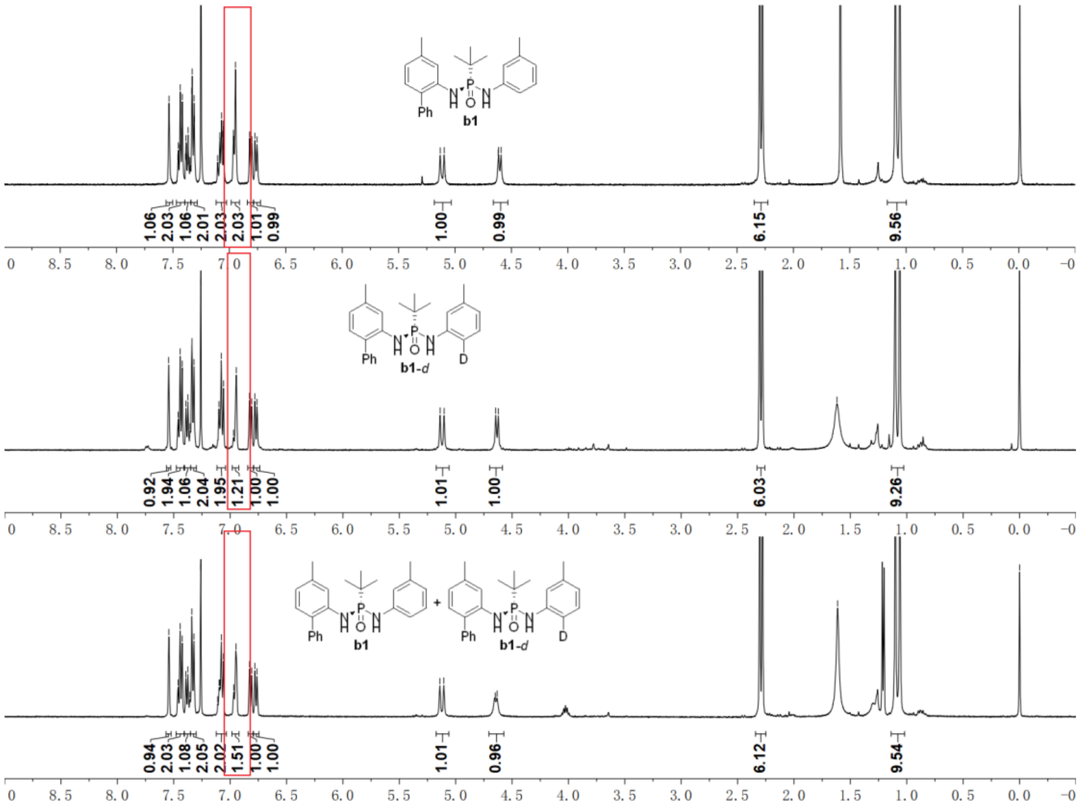

Supplementary Figure 12. ^{1}H NMR Results of KIE Experiments

1.8 X-ray Structure of b18 and d1

(a) X-ray Structure of b18

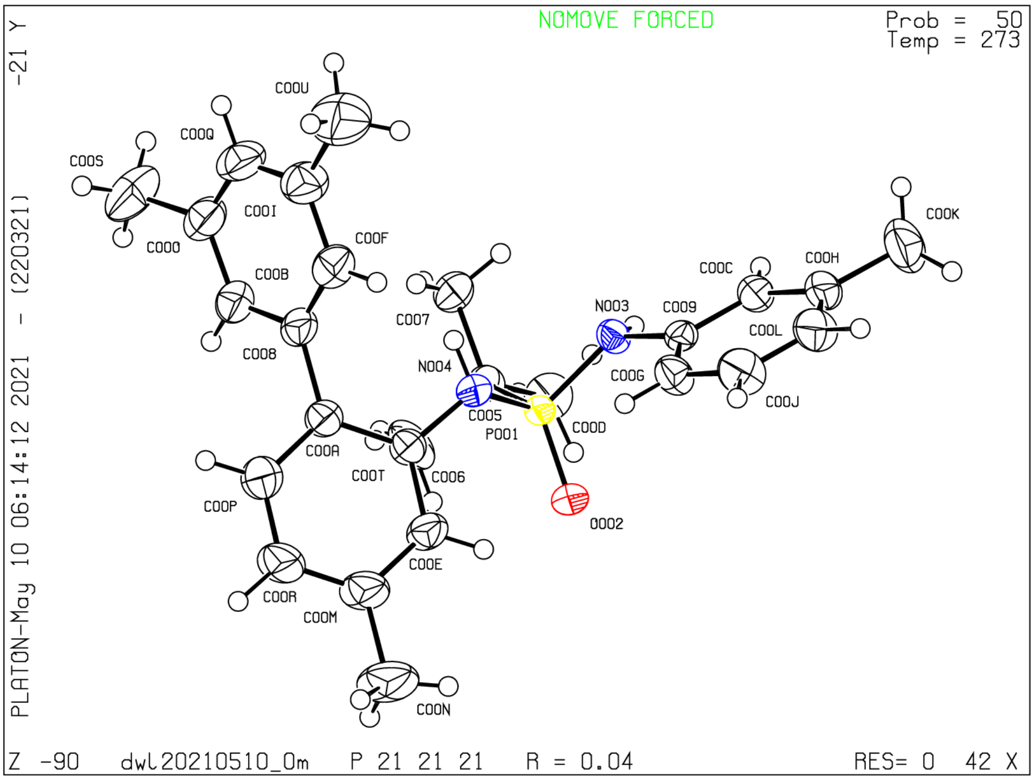

Supplementary Figure 13. ORTEP structure of **b18** with thermal ellipsoids at the 30% probability level (CCDC: 2099084)

Supplementary Table 3. Crystal data for mj21150_0m

Datablock: dwl20210510_0m

Bond precision: C-C = 0.0042 A Wavelength=0.71073

Cell: a=10.327(2) b=12.062(3) c=19.399(5)

alpha=90 beta=90 gamma=90

Temperature: 273 K

Calculated Reported

Volume 2416.4(10) 2416.5(9)

Space group P 21 21 21 P 21 21 21

Hall group P 2ac 2ab P 2ac 2ab

Moiety formula C26 H33 N2 O P C26 H33 N2 O P

Sum formula C26 H33 N2 O P C26 H33 N2 O P

Mr 420.51 420.51

Dx,g cm-3 1.156 1.156

Z 4 4

Mu (mm-1) 0.133 0.133

F000 904.0 904.0

F000’ 904.71
h,k,lmax 13,15,25 13,15,25
Nref 5548[3131] 5507
Tmin, Tmax 0.575,0.746
Tmin’
Correction method= # Reported T Limits: Tmin=0.575 Tmax=0.746
AbsCorr = NONE
Data completeness= 1.76/0.99 Theta(max)= 27.492
R(reflections)= 0.0424 (4827) wR2(reflections)= 0.111 4(5507)
S = 1.027 Npar= 278

(b) X-ray Structure of d1

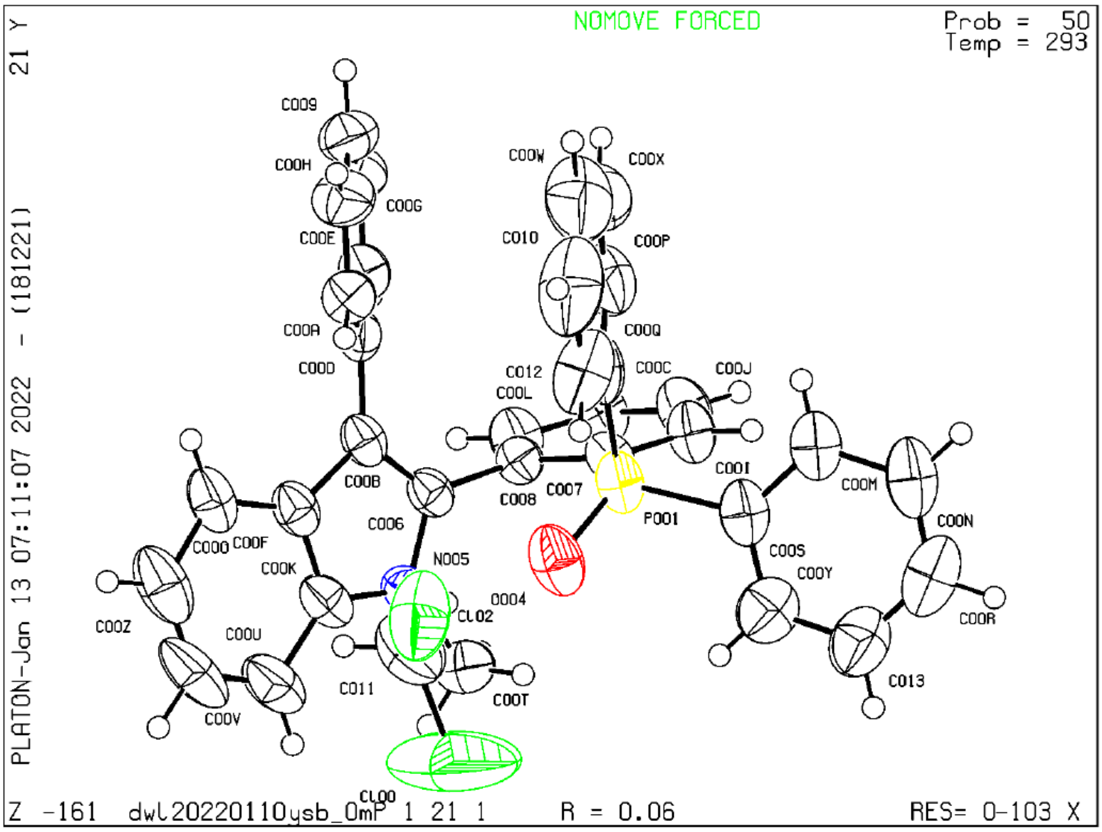

Supplementary Figure 14. ORTEP structure of **d1** with thermal ellipsoids at the 30% probability level (CCDC: 2189868)

Supplementary Table 4. Crystal data for dwl20220110ysb_0m
Datablock: dwl20220110ysb_0m
Bond precision: C-C = 0.0094 A Wavelength=0.71073
Cell: a=9.742(7) b=16.439(12) c=10.106(8)
alpha=90 beta=113.157(17) gamma=90
Temperature: 293 K
Calculated Reported
Volume 1488.1(19) 1488.1(19)

Space group P 21 P 1 21 1
Hall group P 2yb P 2yb
Moiety formula C33 H26 N O P, C H2 Cl2 C33 H26 N O P, C H2 Cl2
Sum formula C34 H28 Cl2 N O P C34 H28 Cl2 N O P
Mr 568.44 568.44
Dx,g cm-3 1.269 1.269
Z 2 2
Mu (mm-1) 0.299 0.299
F000 592.0 592.0
F000’ 592.99
h,k,lmax 12,21,13 12,21,13
Nref 6890[3566] 6858
Tmin,Tmax 0.679,0.746
Tmin’
Correction method= # Reported T Limits: Tmin=0.679 Tmax=0.746
AbsCorr = NONE
Data completeness= 1.92/1.00 Theta(max)= 27.572
R(reflections)= 0.0612(4434)
wR2(reflections)=
0.1750(6858)
S = 1.099 Npar= 353

1.7 NMR spectra

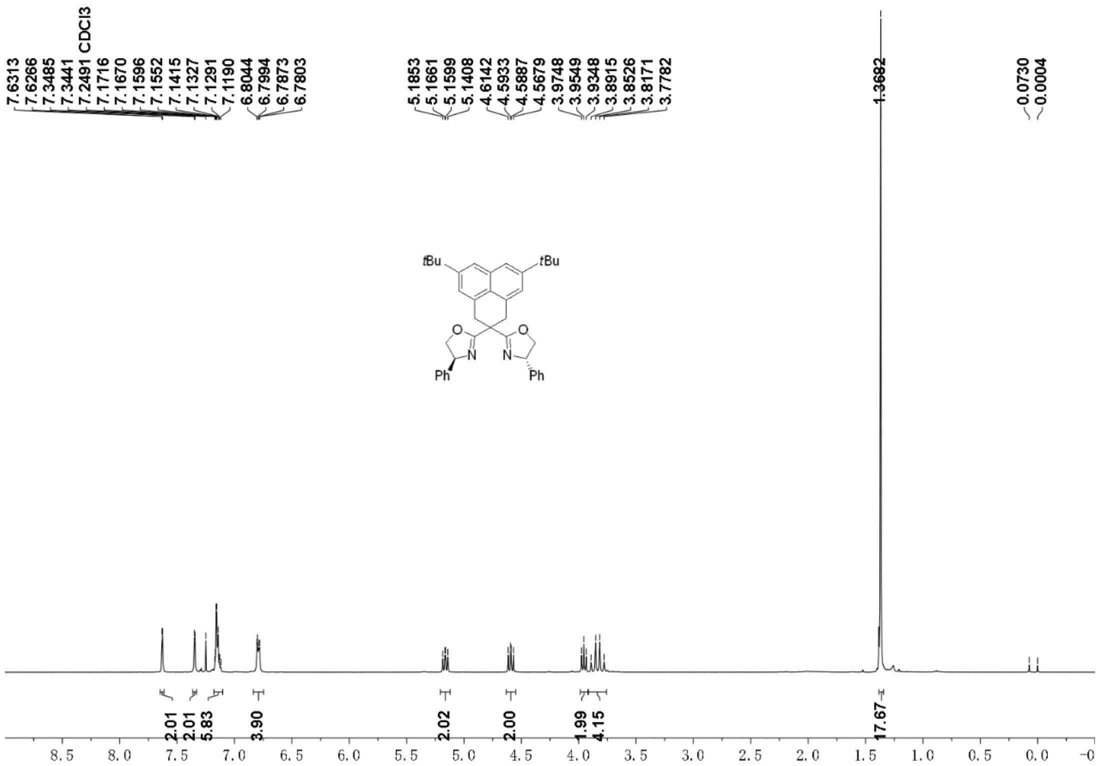

Supplementary Figure 15. ^{1}H NMR spectrum of **L16**

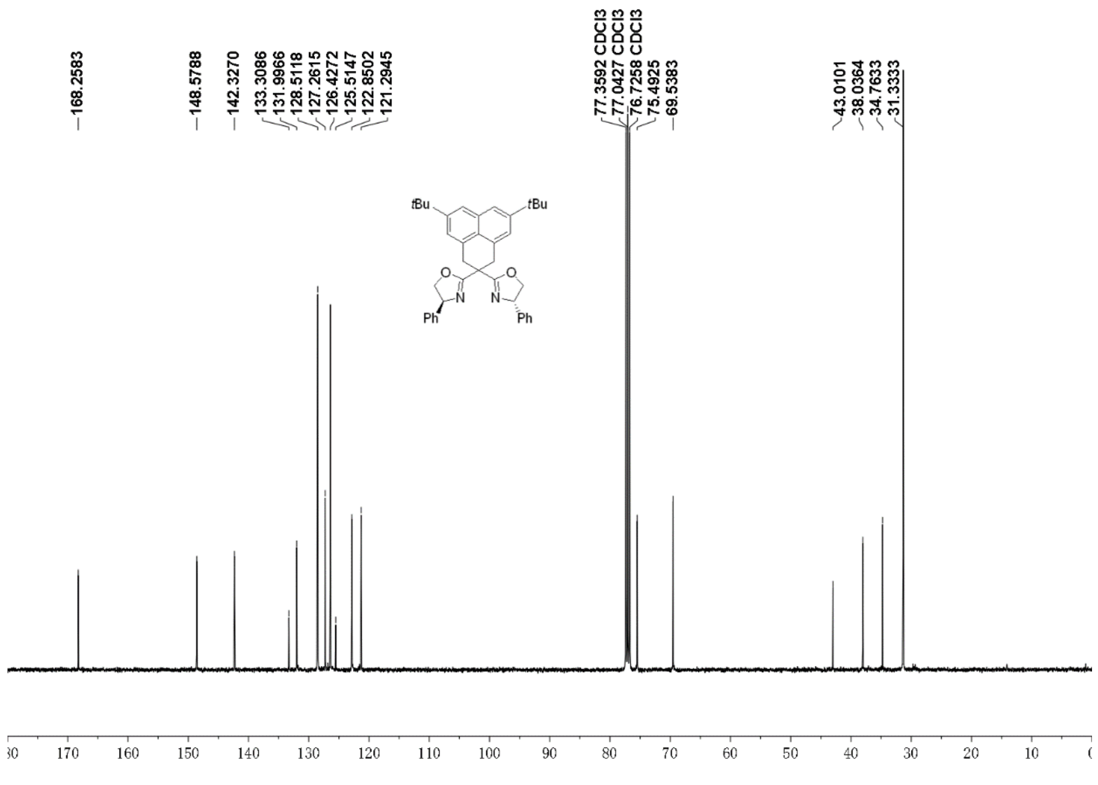

Supplementary Figure 16. ^{13}C NMR spectrum of **L16**

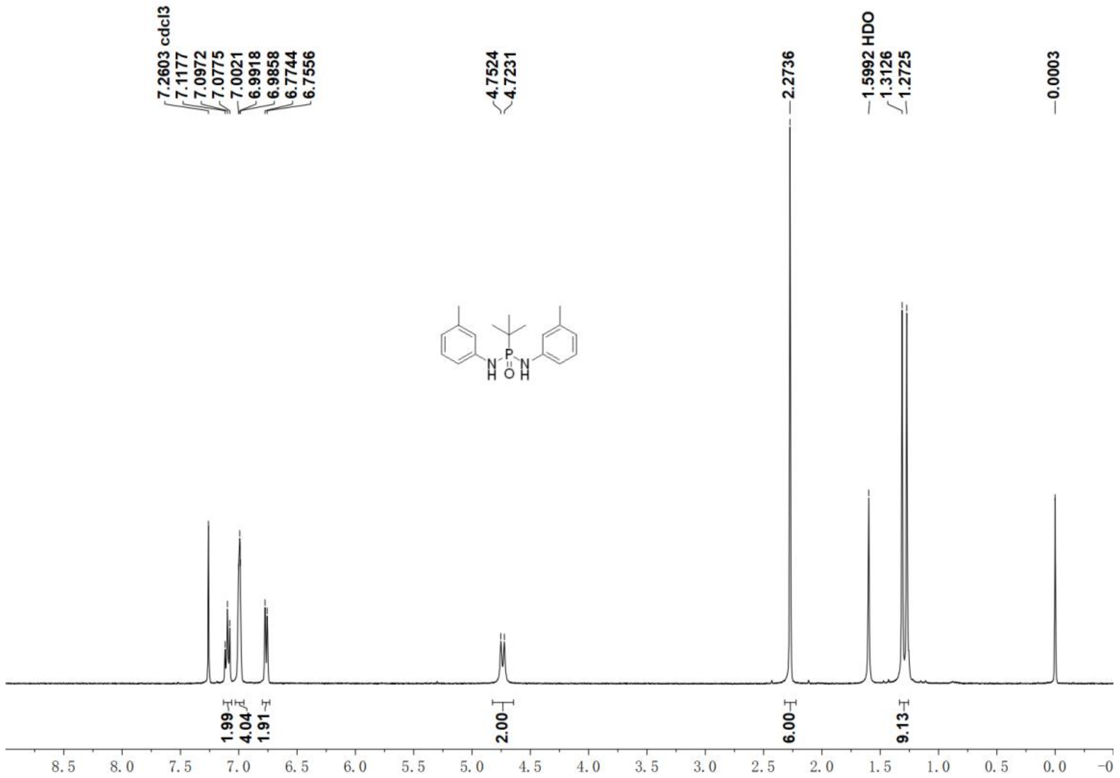

Supplementary Figure 17. ^{1}H NMR spectrum of **a1**

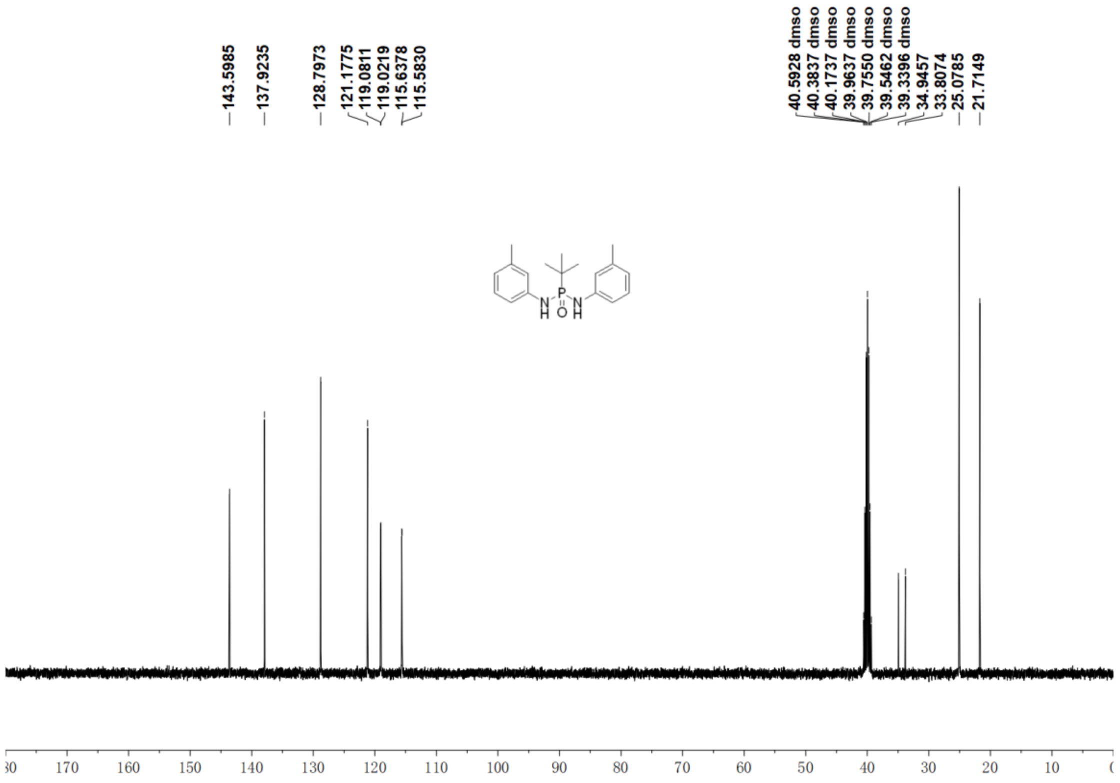

Supplementary Figure 18. ^{13}C NMR spectrum of **a1**

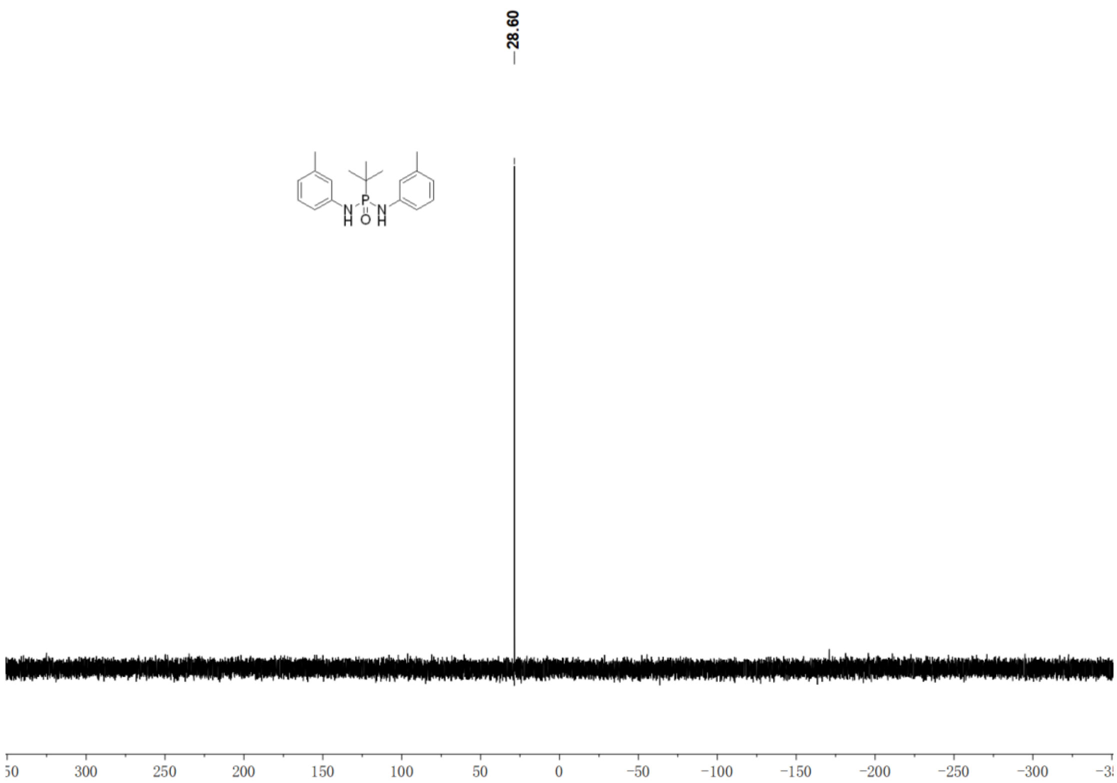

Supplementary Figure 19. ^{31}P NMR spectrum of **a1**

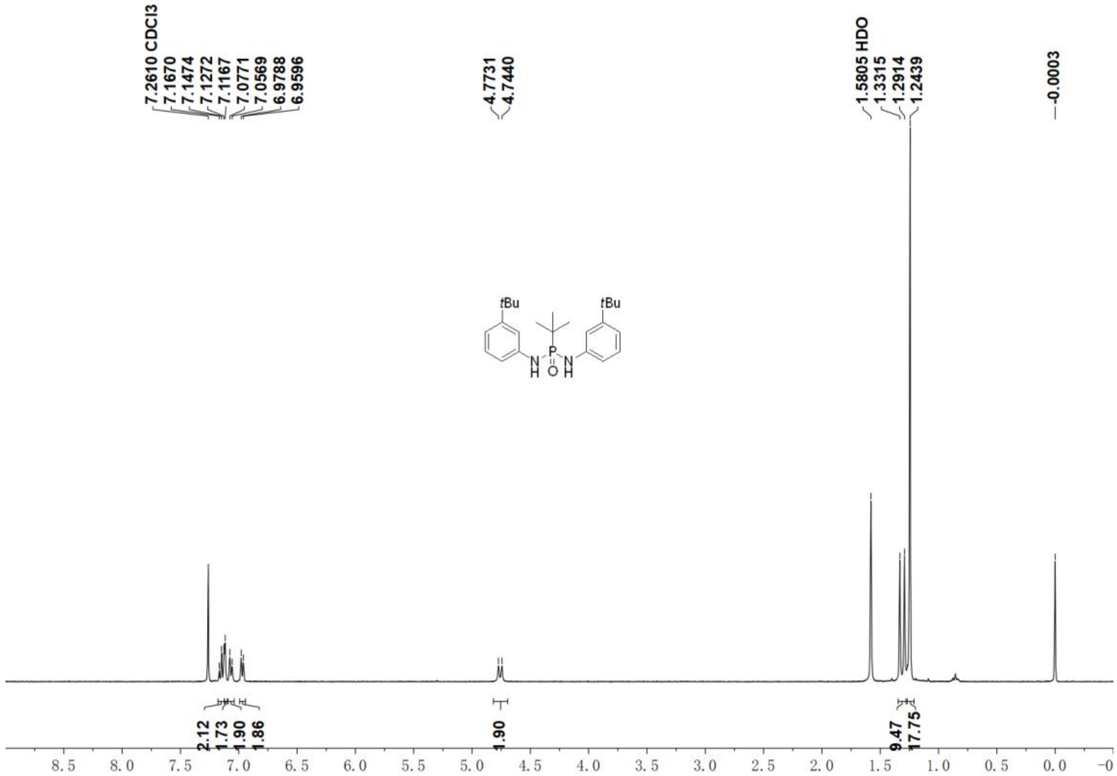

Supplementary Figure 20. ^{1}H NMR spectrum of **a2**

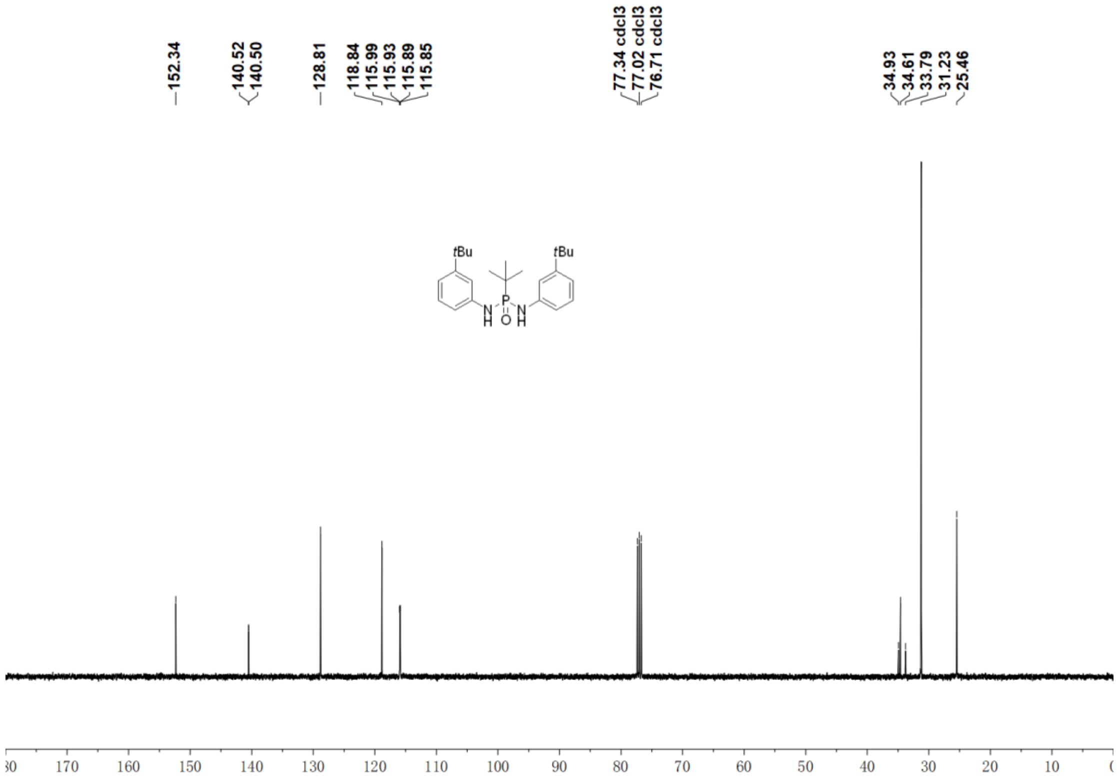

Supplementary Figure 21. ^{13}C NMR spectrum of **a2**

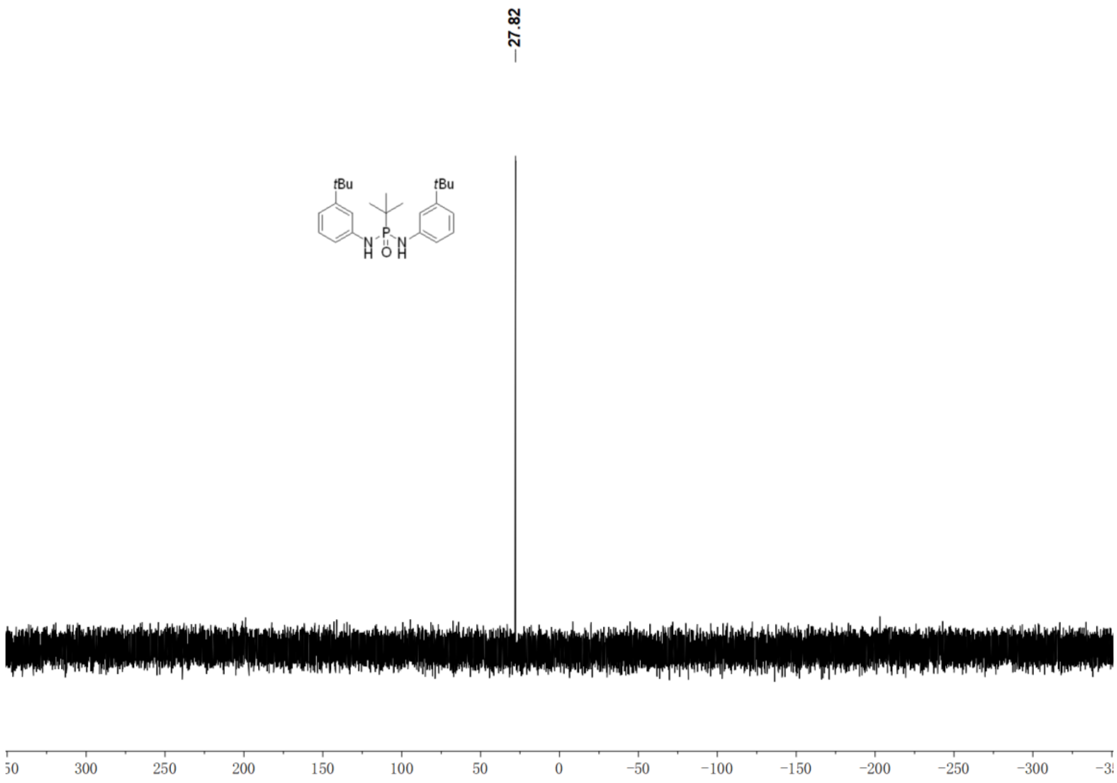

Supplementary Figure 22. ^{31}P NMR spectrum of **a2**

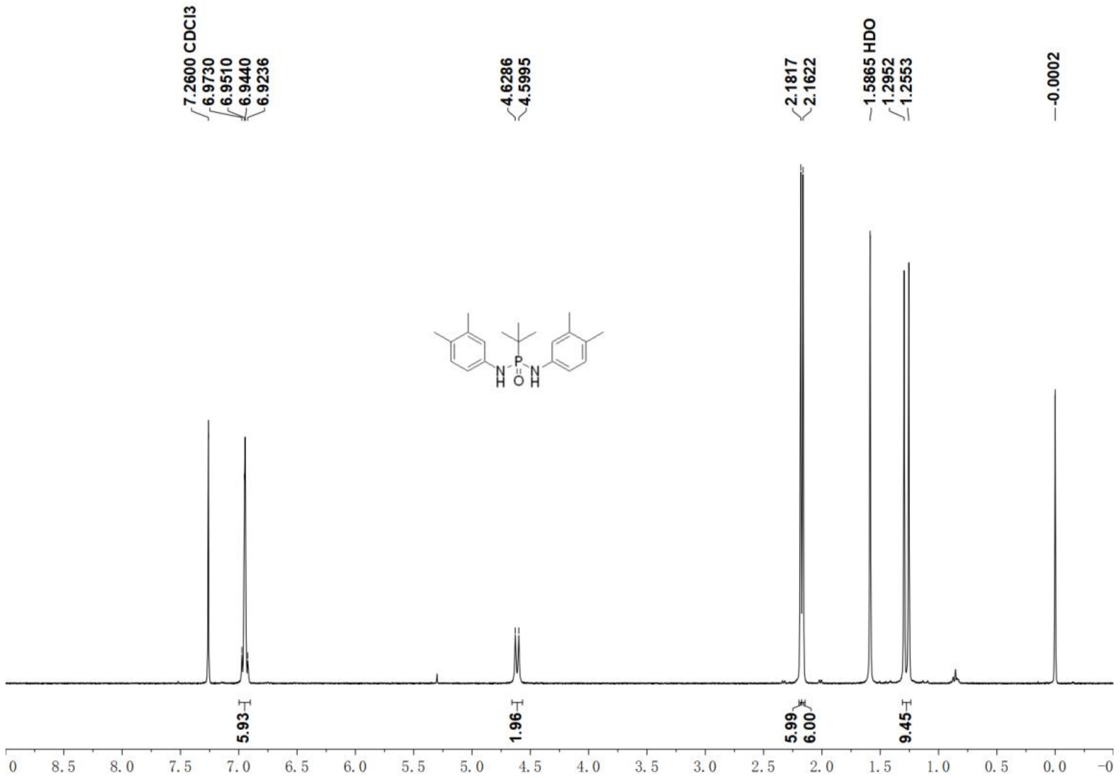

Supplementary Figure 23. ^{1}H NMR spectrum of **a3**

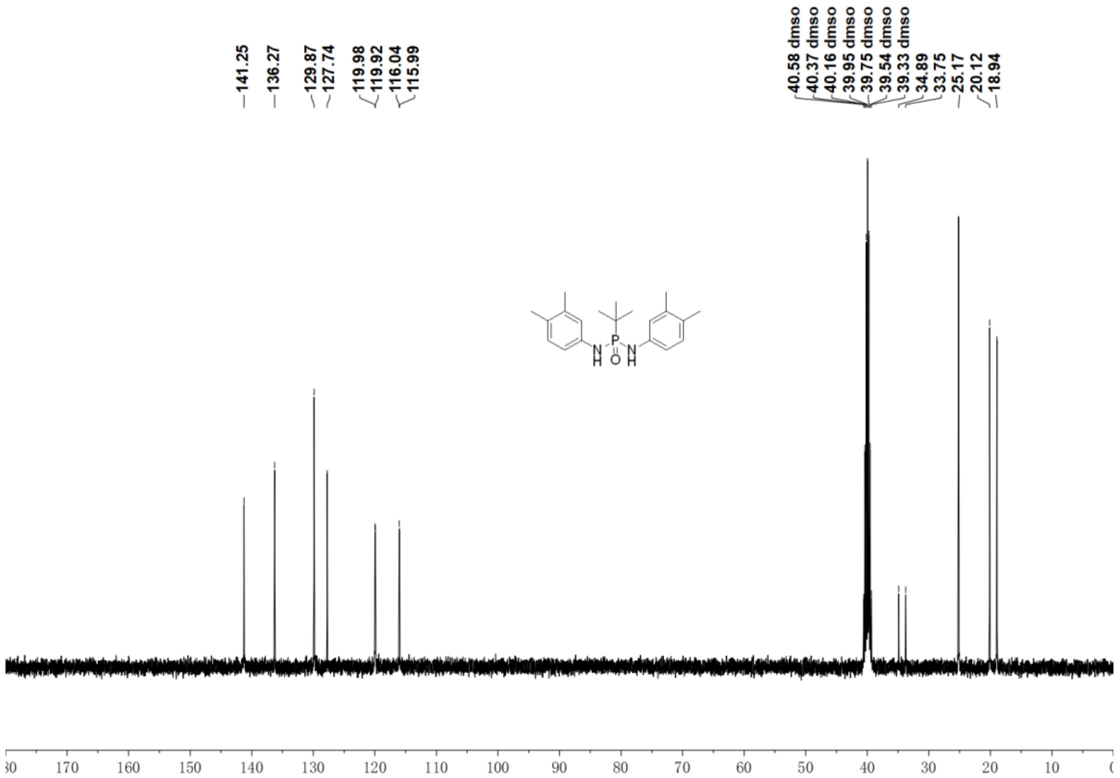

Supplementary Figure 24. ^{13}C NMR spectrum of **a3**

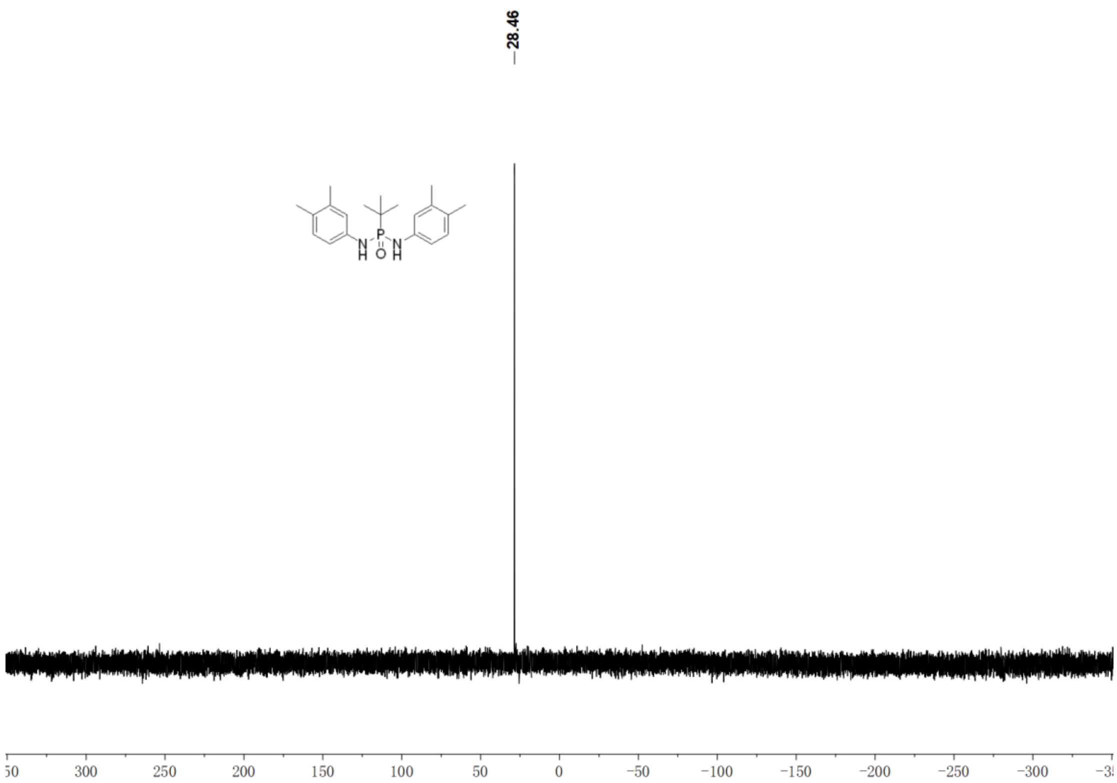

Supplementary Figure 25. ^{31}P NMR spectrum of **a3**

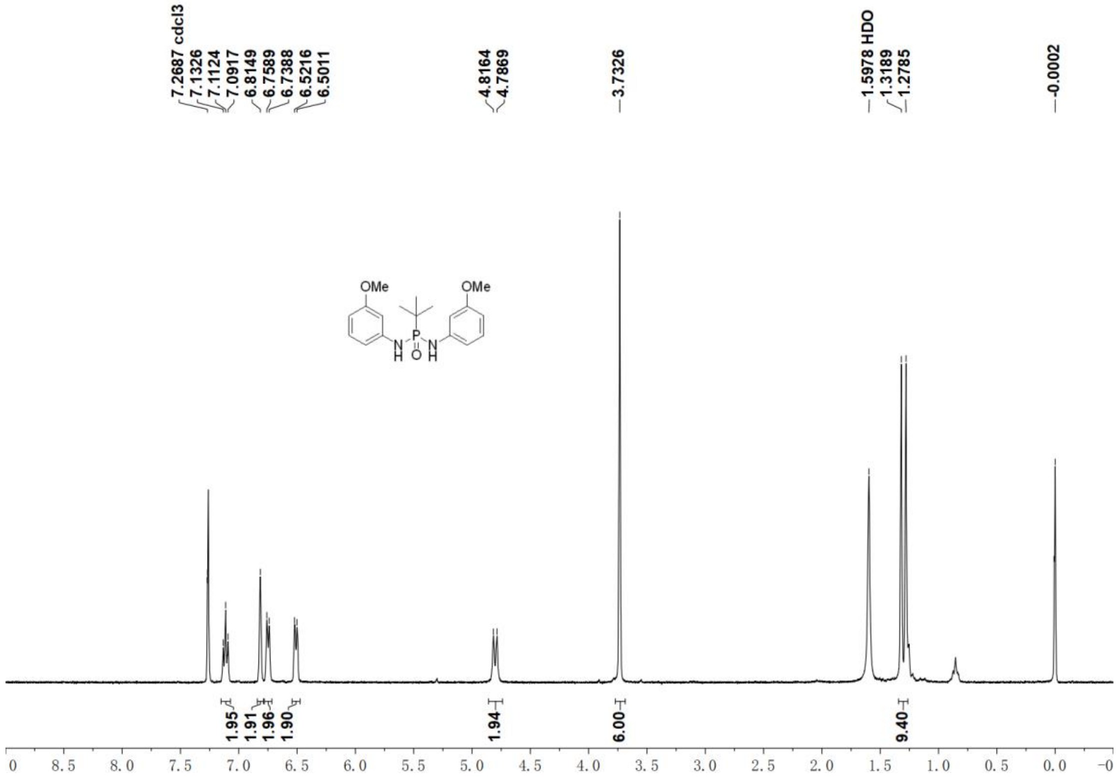

Supplementary Figure 26. ^{1}H NMR spectrum of **a4**

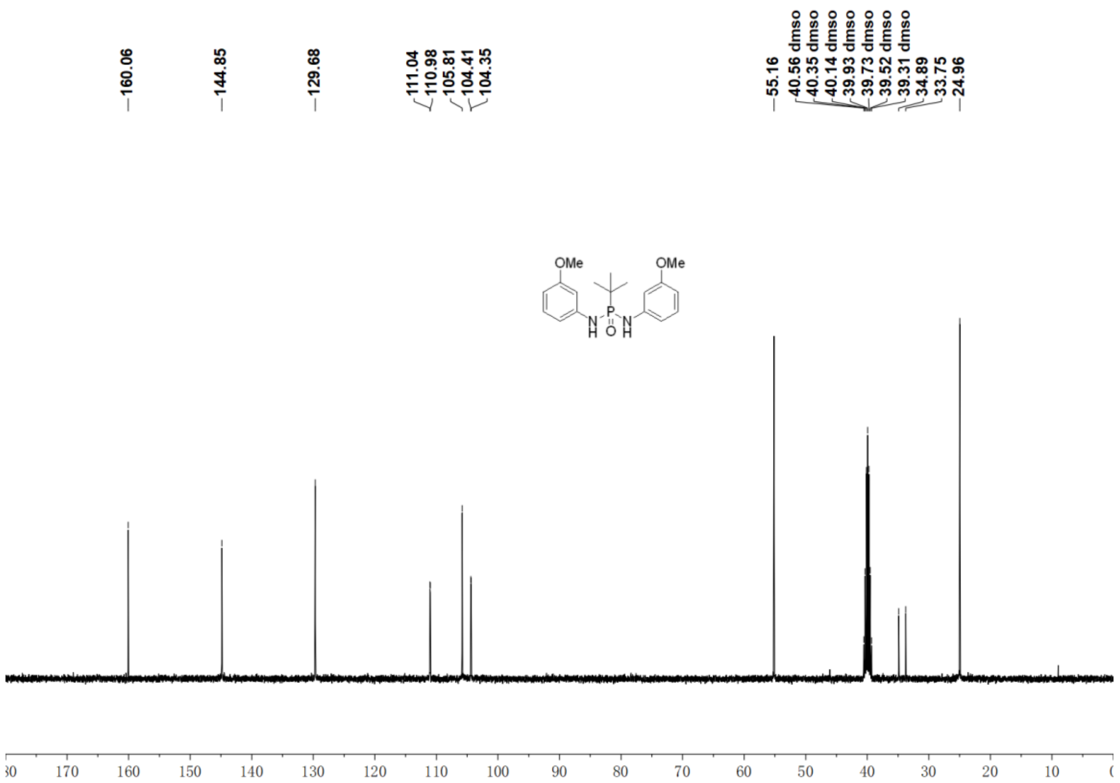

Supplementary Figure 27. ^{13}C NMR spectrum of **a4**

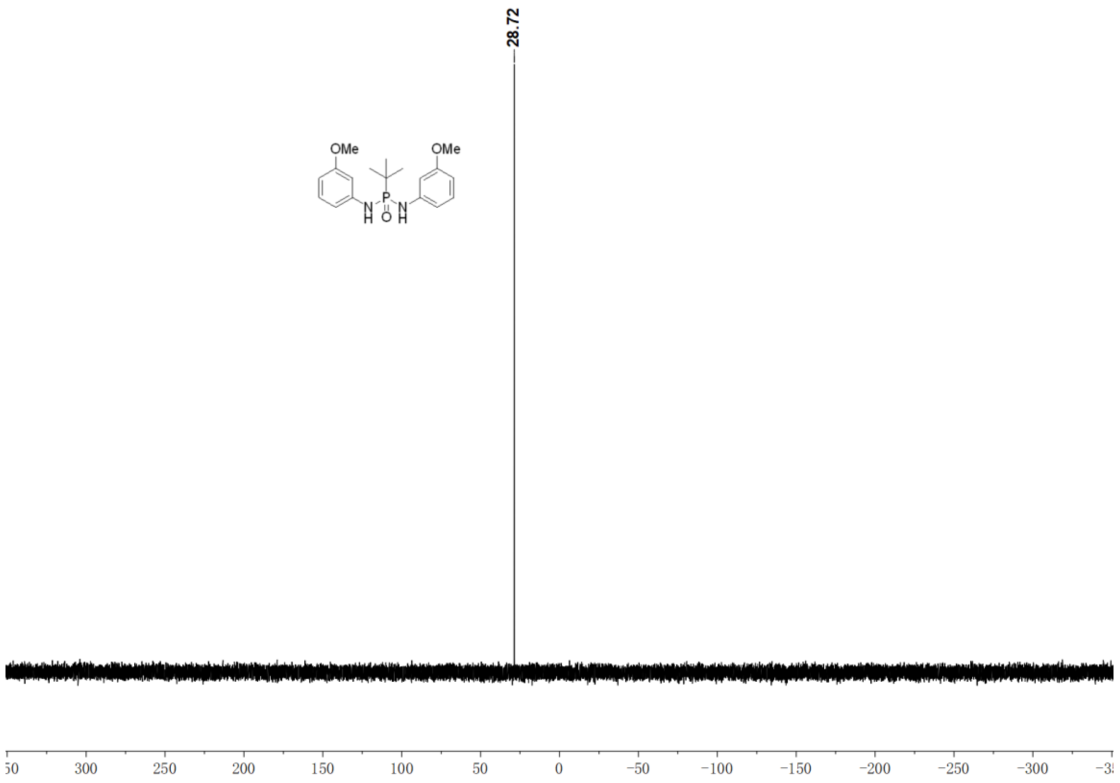

Supplementary Figure 28. ^{31}P NMR spectrum of **a4**

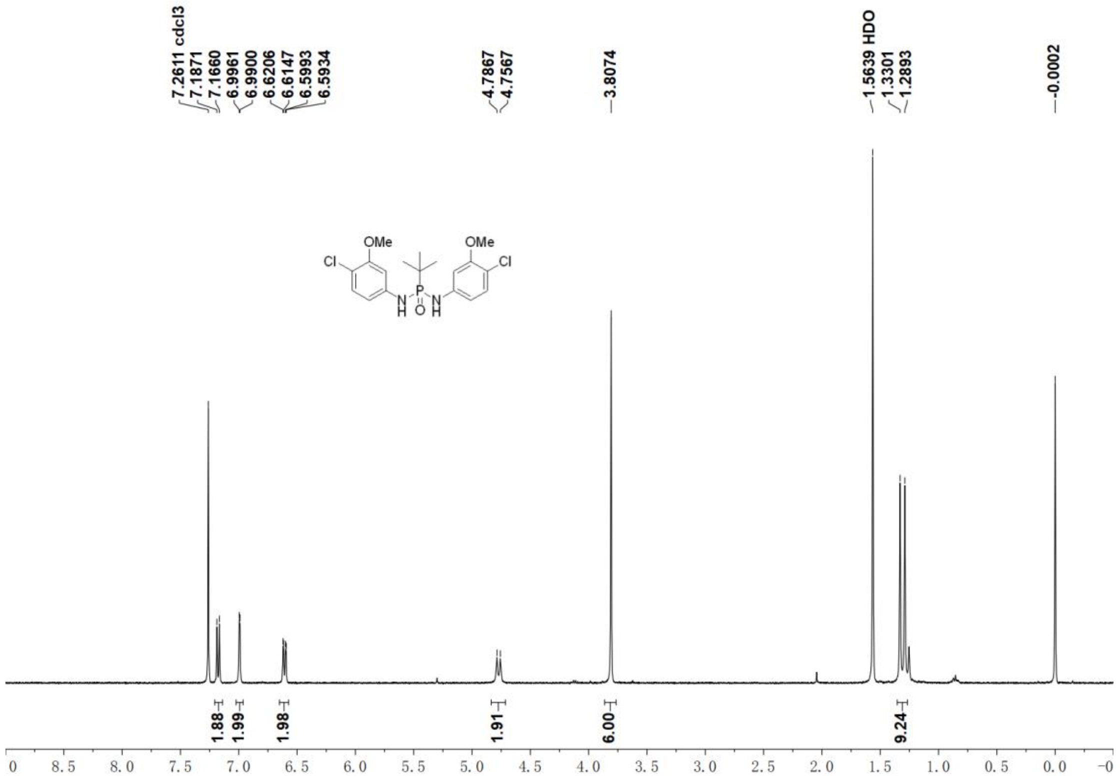

Supplementary Figure 29. ^{1}H NMR spectrum of **a5**

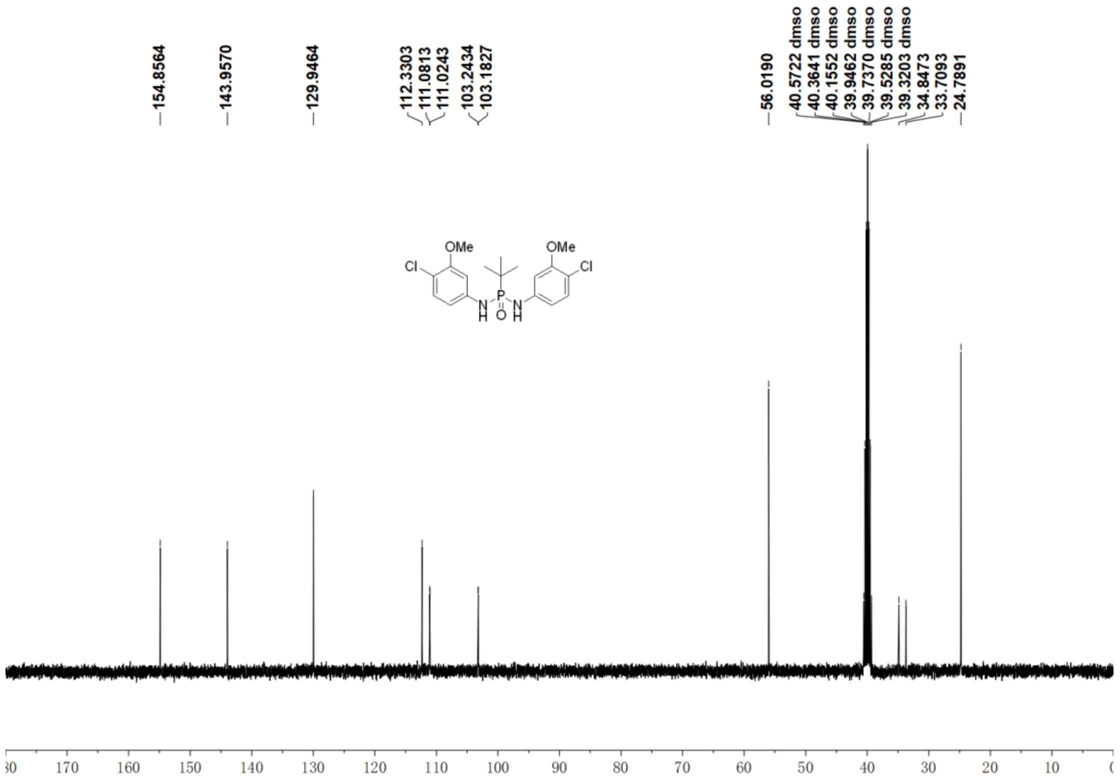

Supplementary Figure 30. ^{13}C NMR spectrum of **a5**

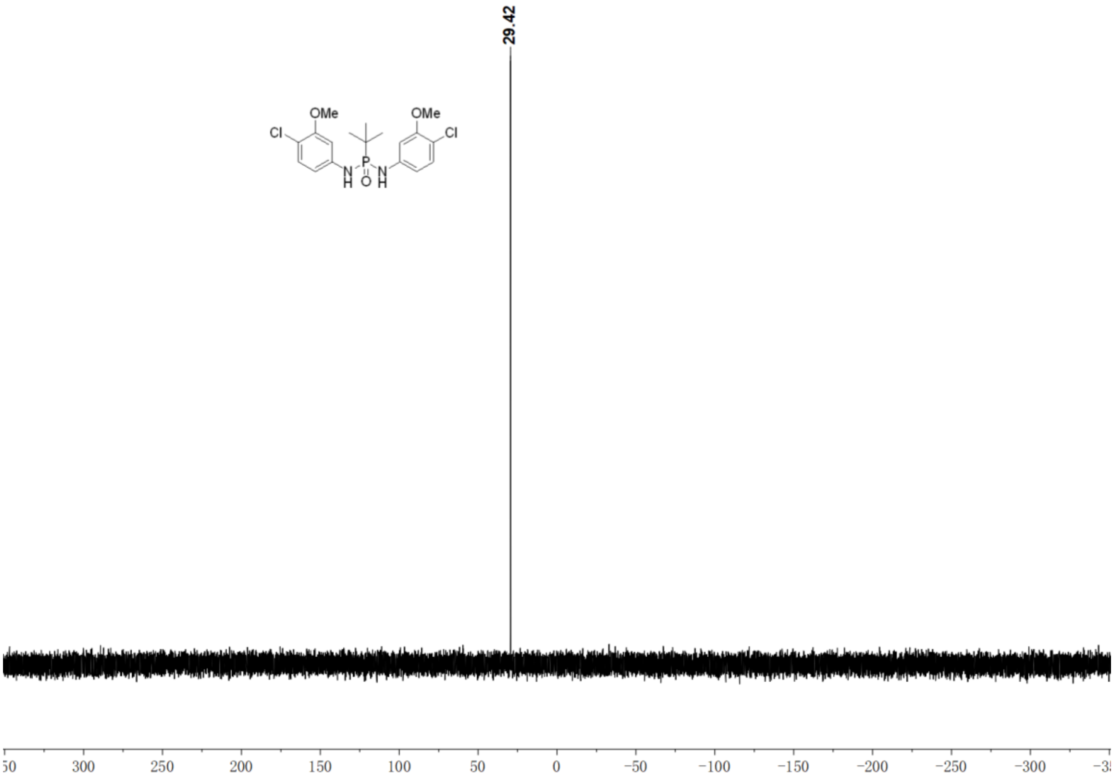

Supplementary Figure 31. ^{31}P NMR spectrum of **a5**

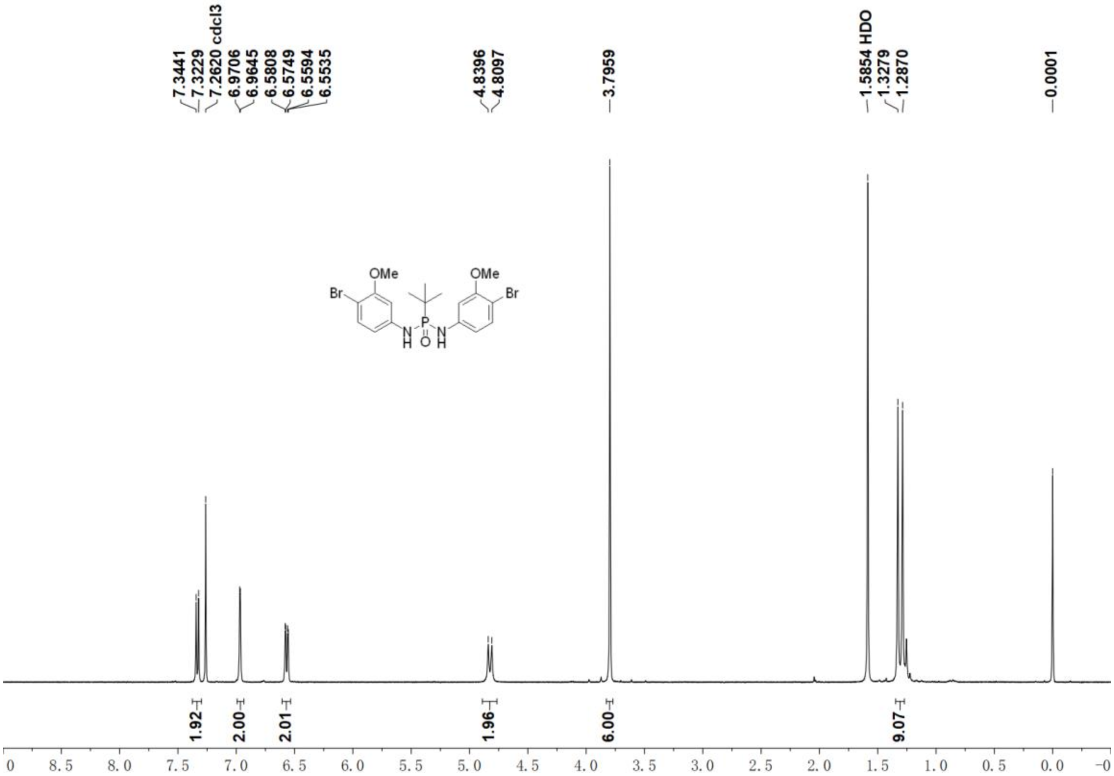

Supplementary Figure 32. ^{1}H NMR spectrum of **a6**

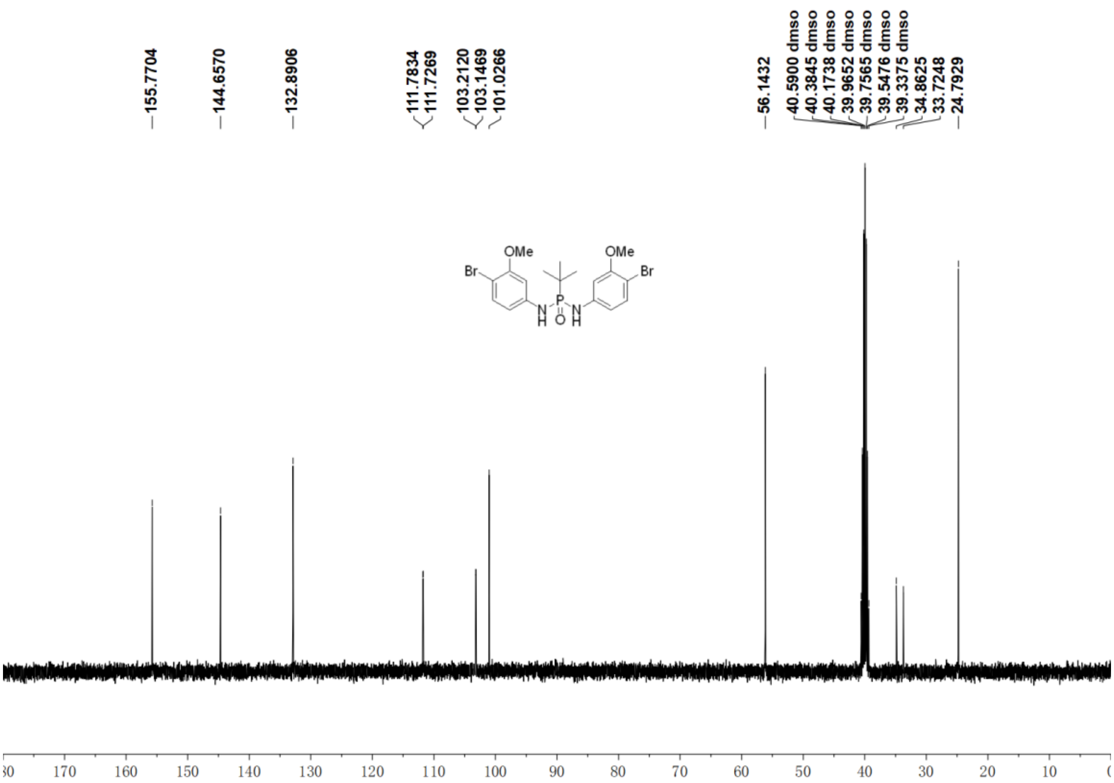

Supplementary Figure 33. ^{13}C NMR spectrum of **a6**

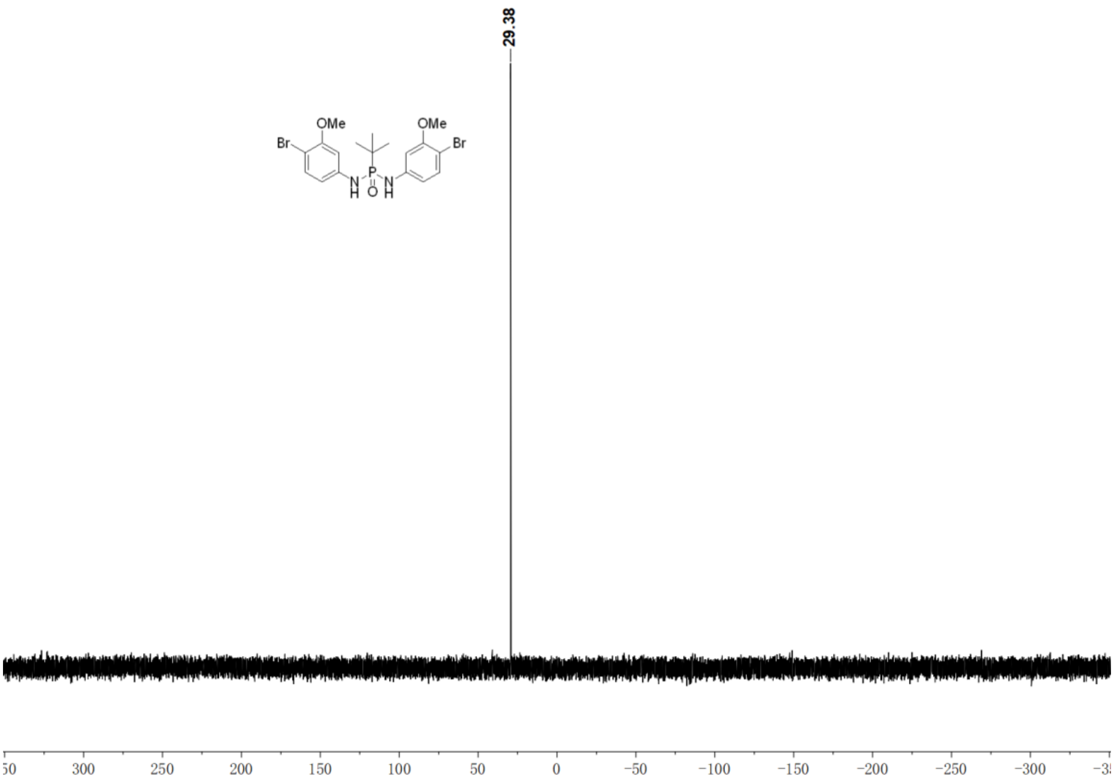

Supplementary Figure 34. ^{31}P NMR spectrum of **a6**

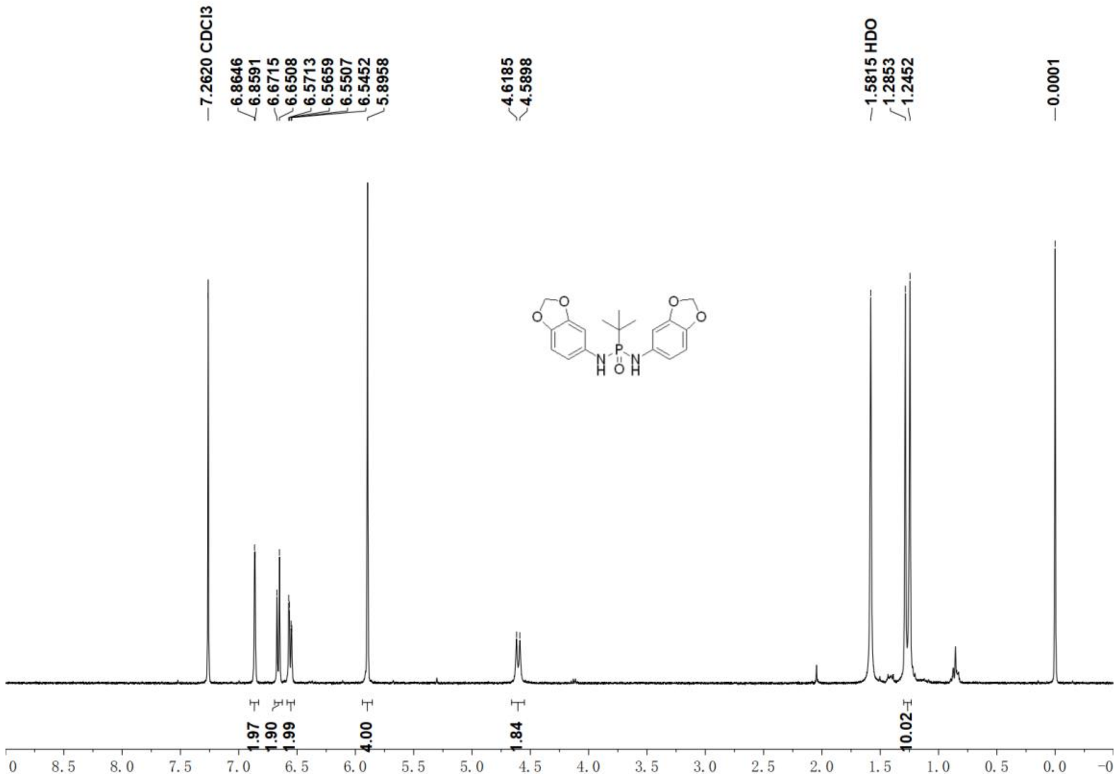

Supplementary Figure 35. ^{1}H NMR spectrum of **a7**

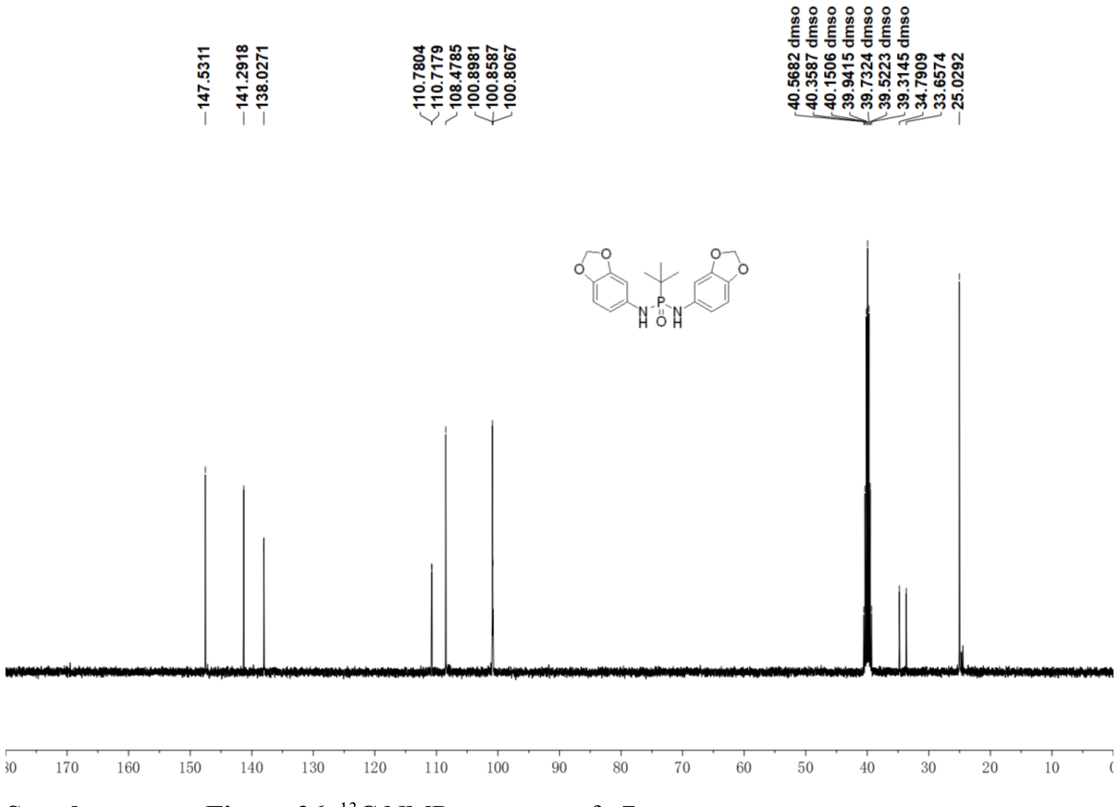

Supplementary Figure 36. ^{13}C NMR spectrum of **a7**

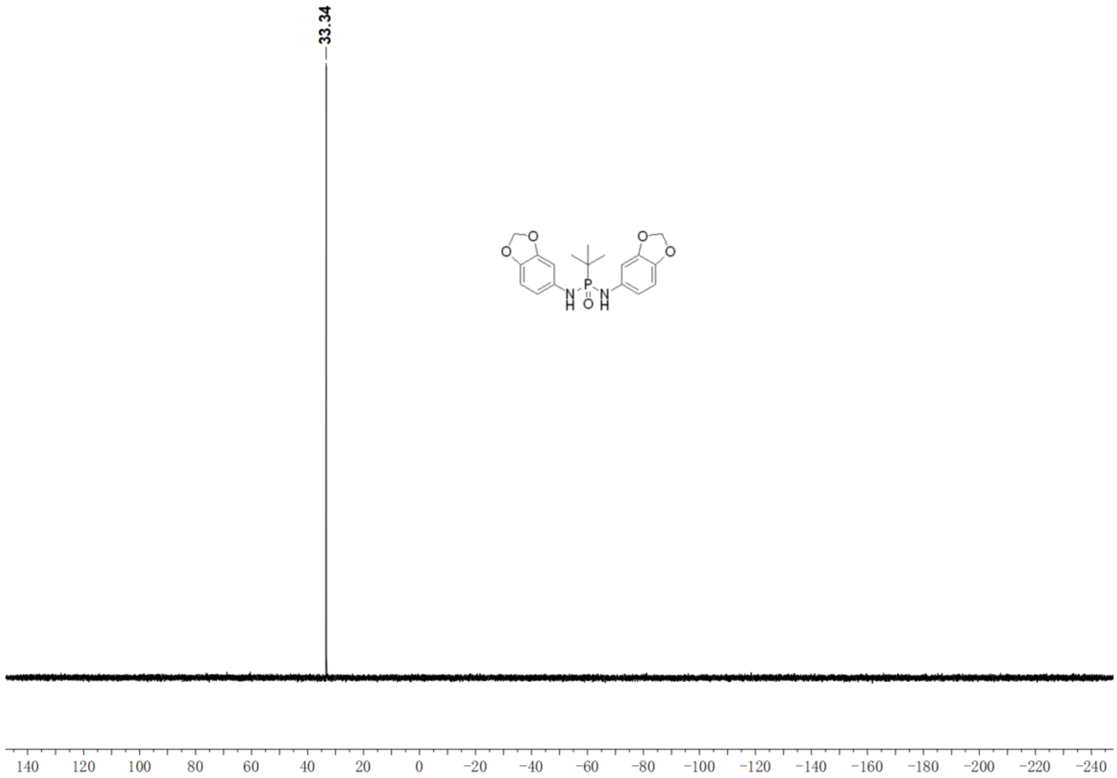

Supplementary Figure 37. ^{31}P NMR spectrum of **a7**

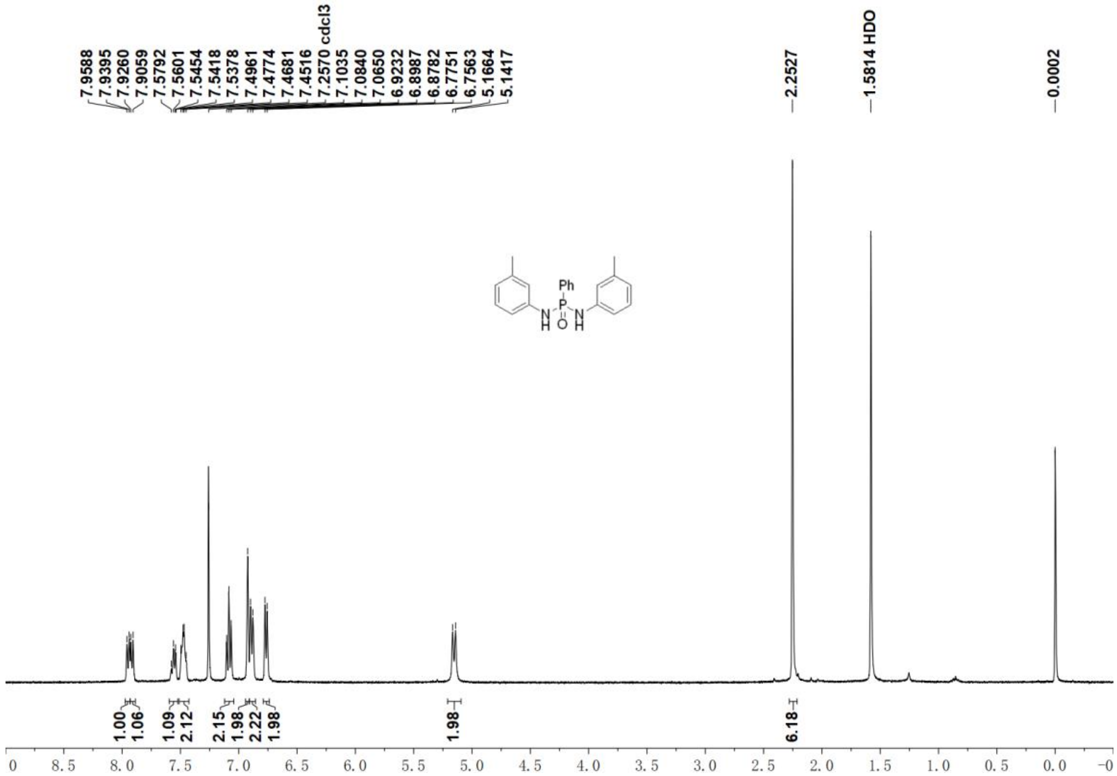

Supplementary Figure 38. ^{1}H NMR spectrum of **a8**

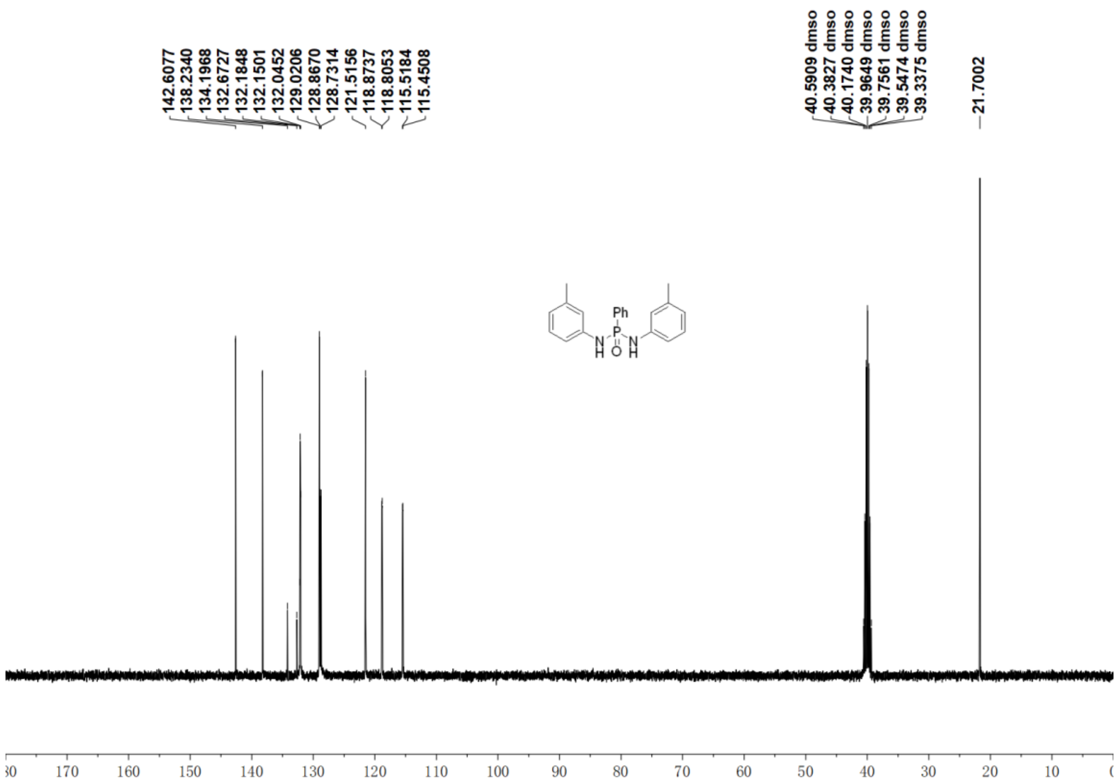

Supplementary Figure 39. ^{13}C NMR spectrum of **a8**

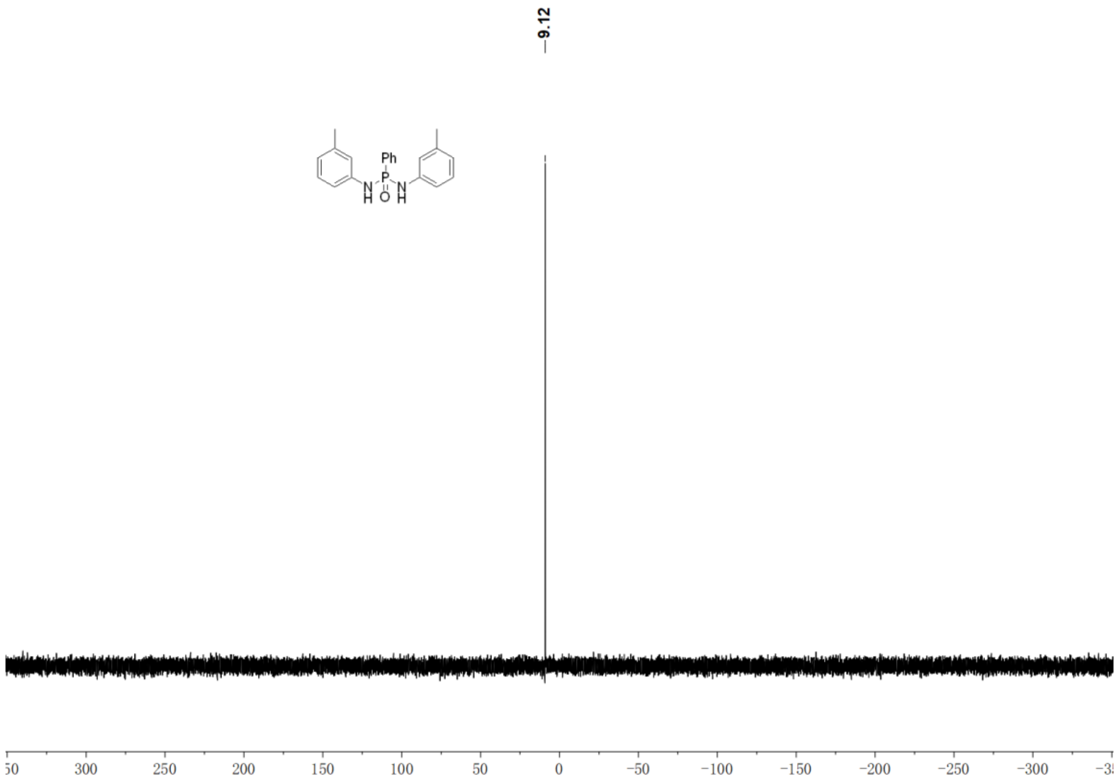

Supplementary Figure 40. ^{31}P NMR spectrum of **a8**

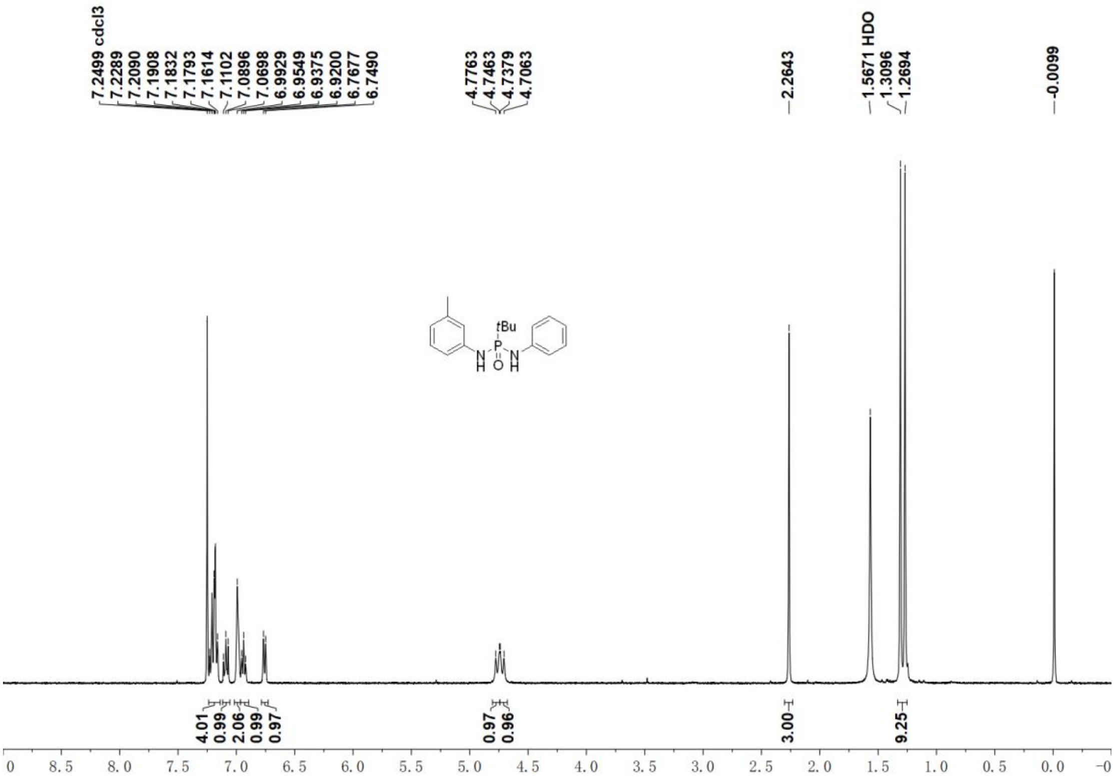

Supplementary Figure 41. ^{1}H NMR spectrum of **a9**

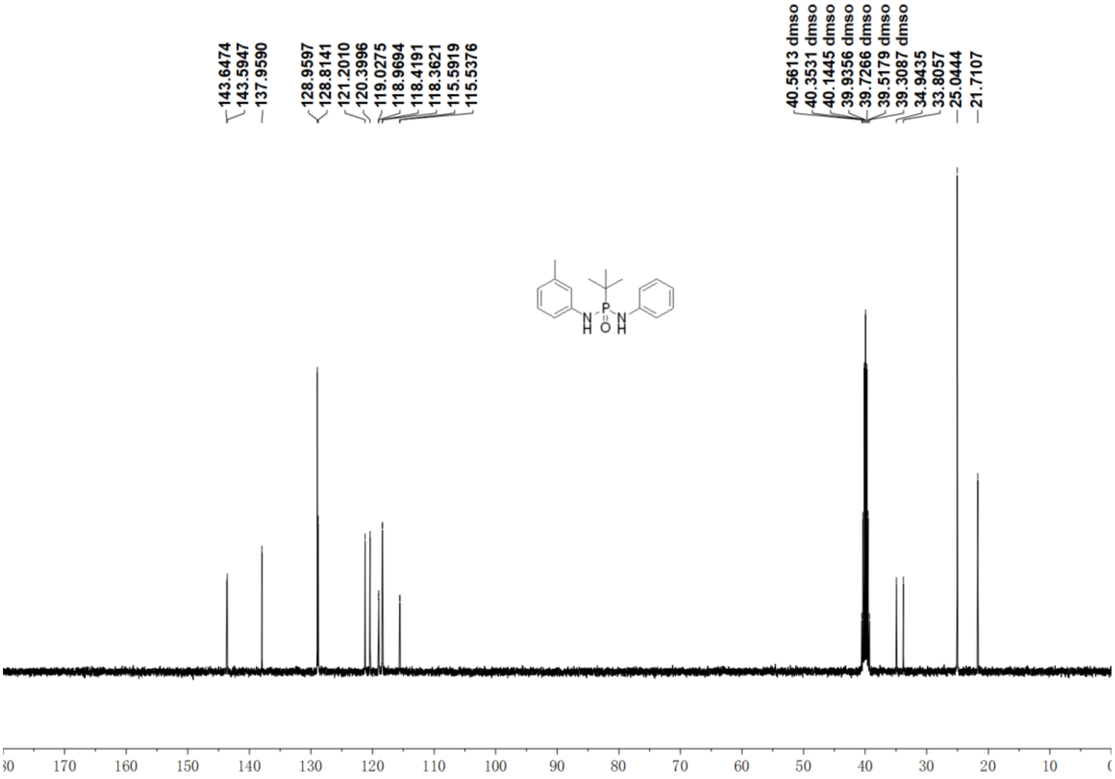

Supplementary Figure 42. ^{13}C NMR spectrum of **a9**

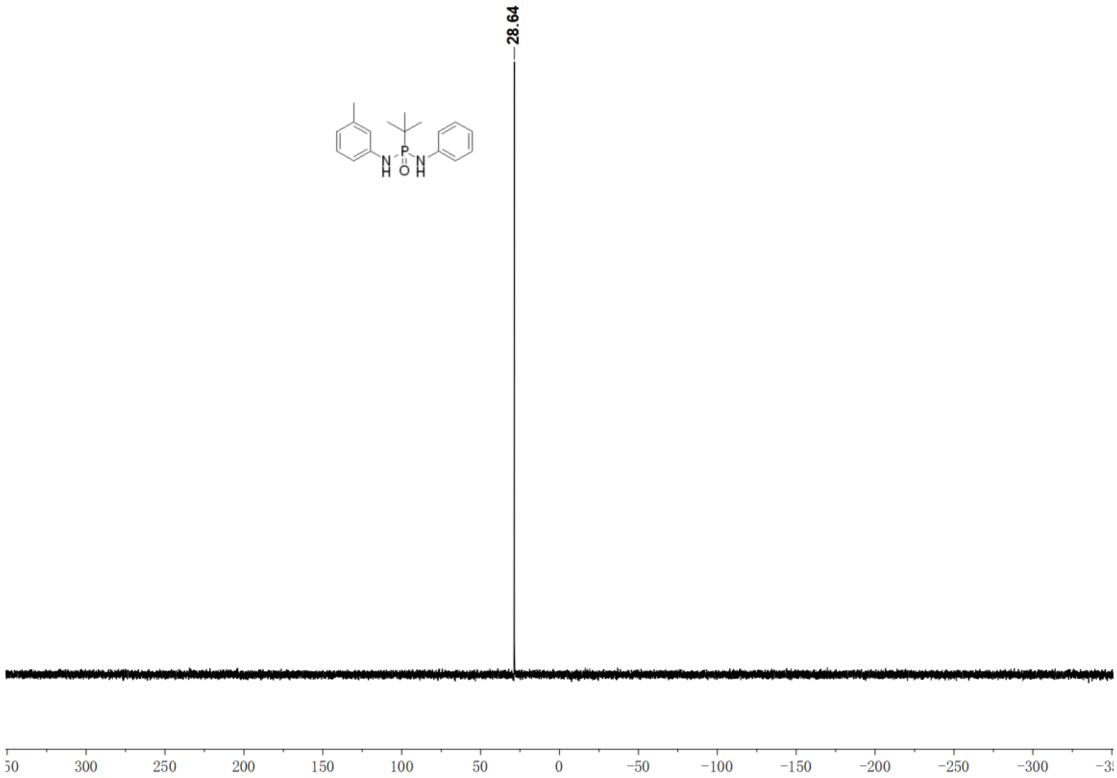

Supplementary Figure 43. ^{31}P NMR spectrum of **a9**

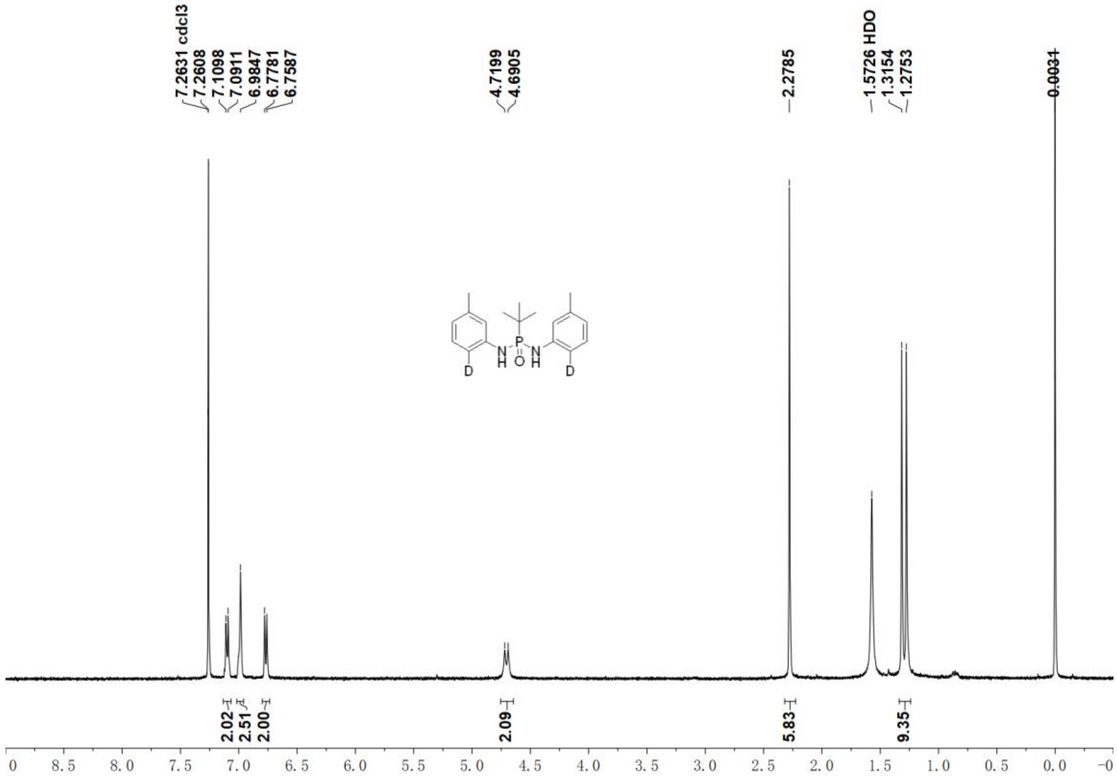

Supplementary Figure 44. ^{1}H NMR spectrum of **a10**

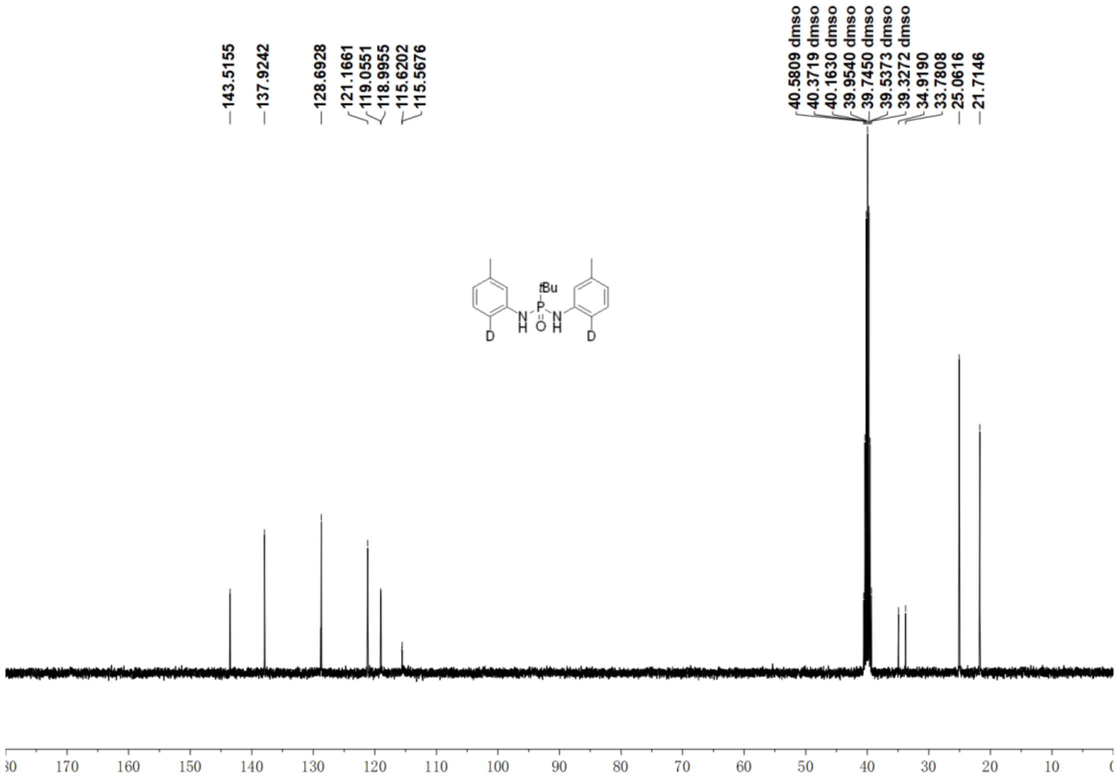

Supplementary Figure 45. ^{13}C NMR spectrum of **a10**

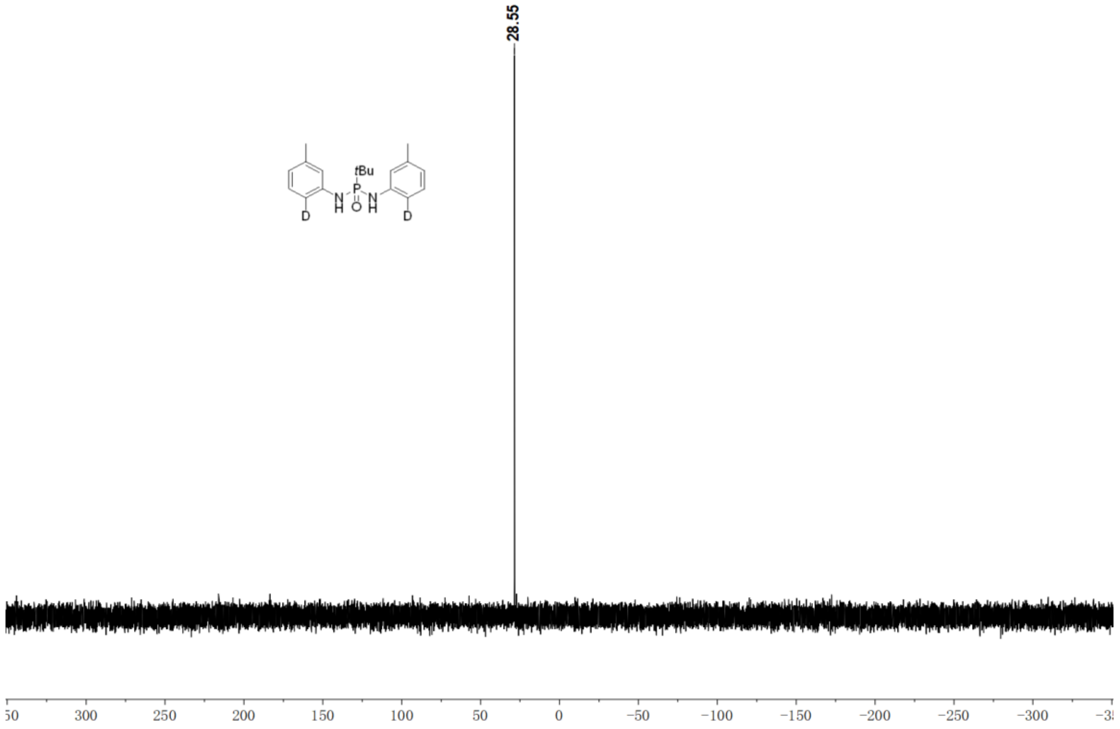

Supplementary Figure 46. ^{31}P NMR spectrum of **a10**

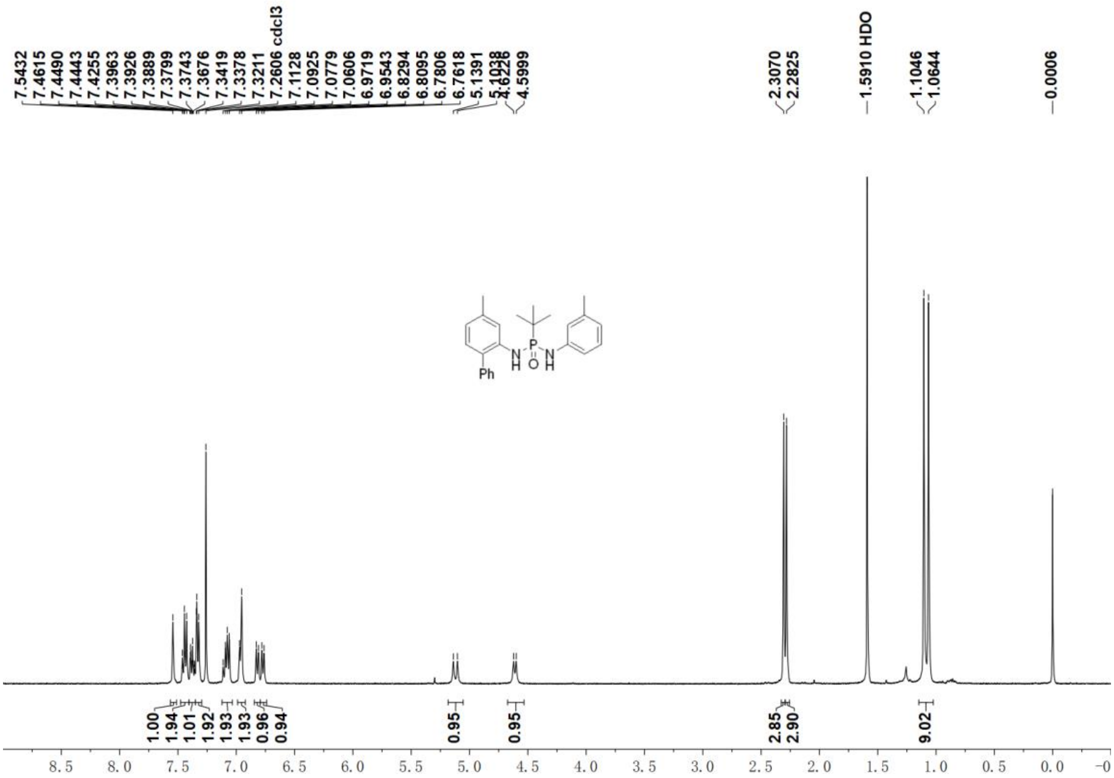

Supplementary Figure 47. ^{1}H NMR spectrum of **b1**

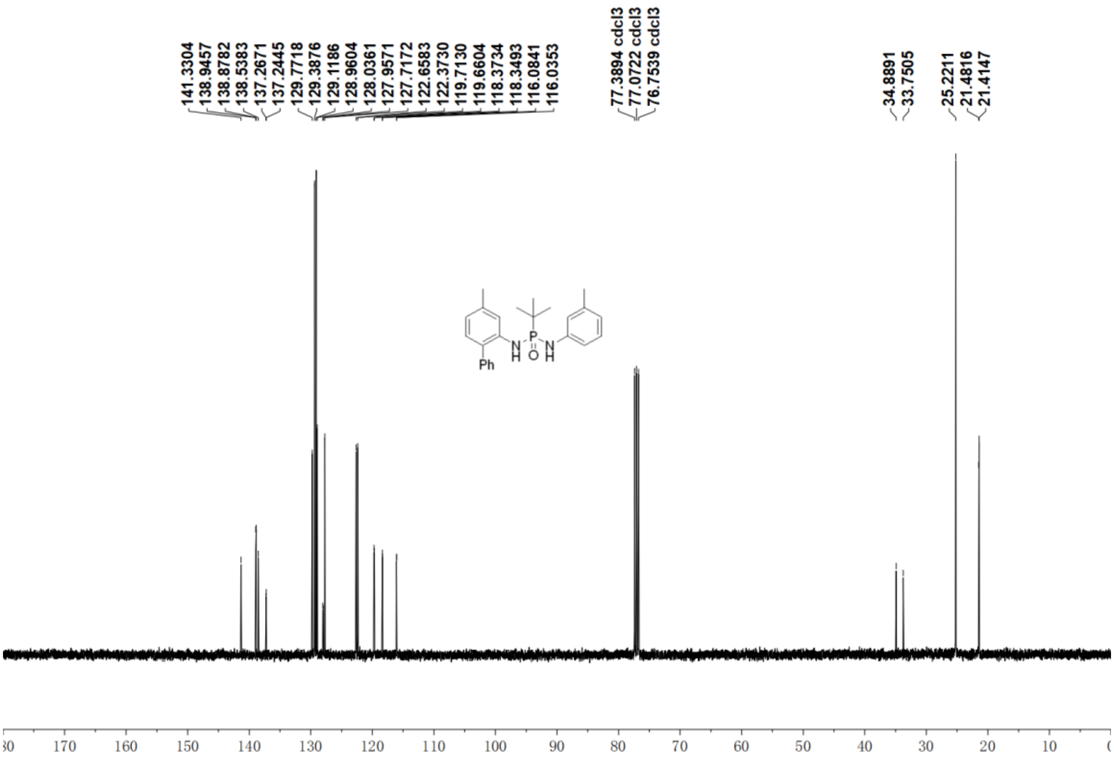

Supplementary Figure 48. ^{13}C NMR spectrum of **b1**

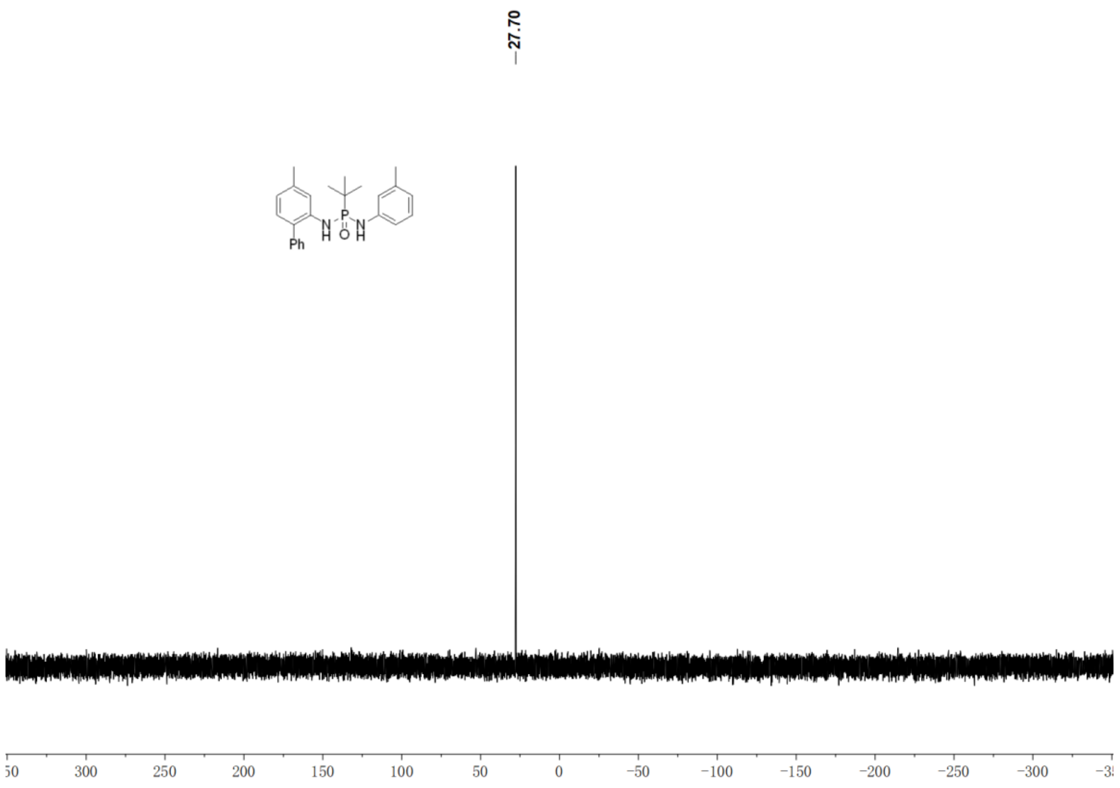

Supplementary Figure 49. ^{31}P NMR spectrum of **b1**

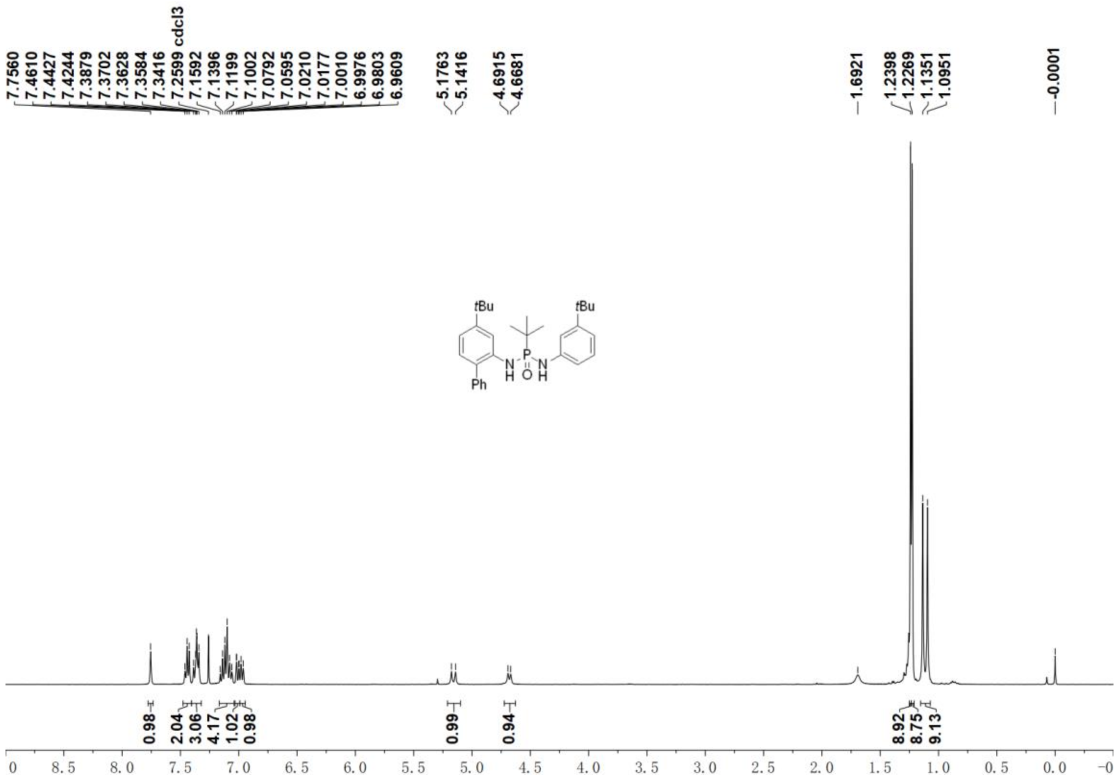

Supplementary Figure 50. ^{1}H NMR spectrum of **b2**

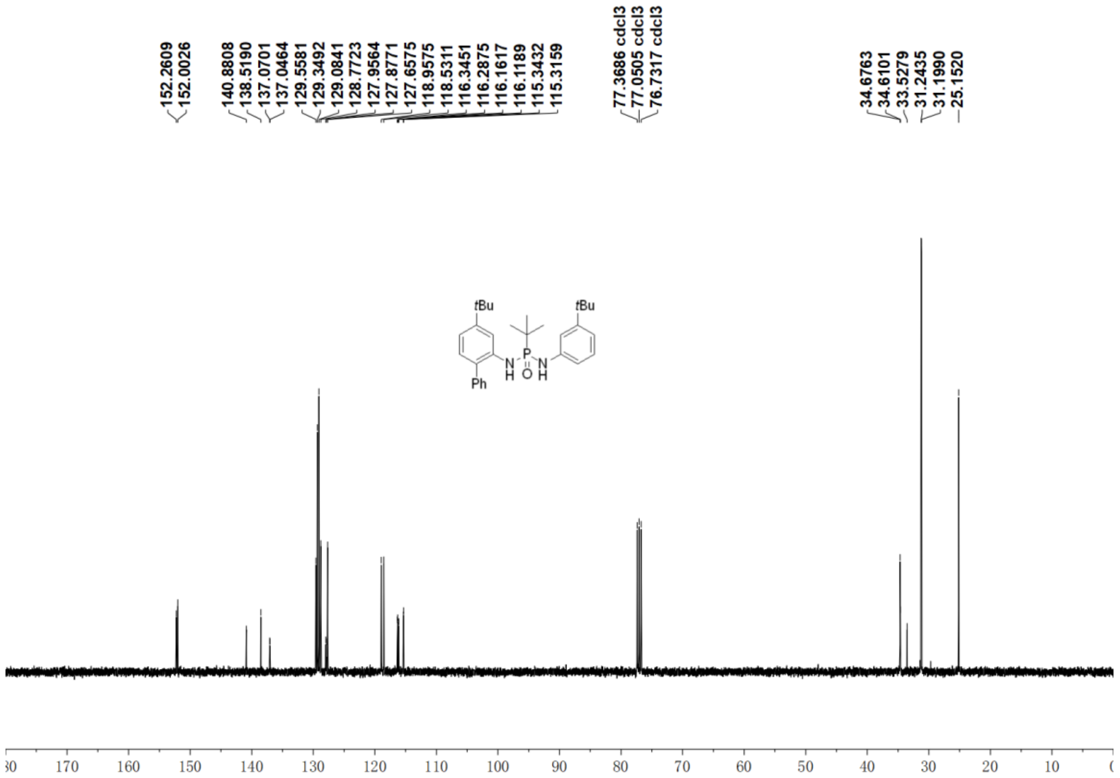

Supplementary Figure 51. ^{13}C NMR spectrum of **b2**

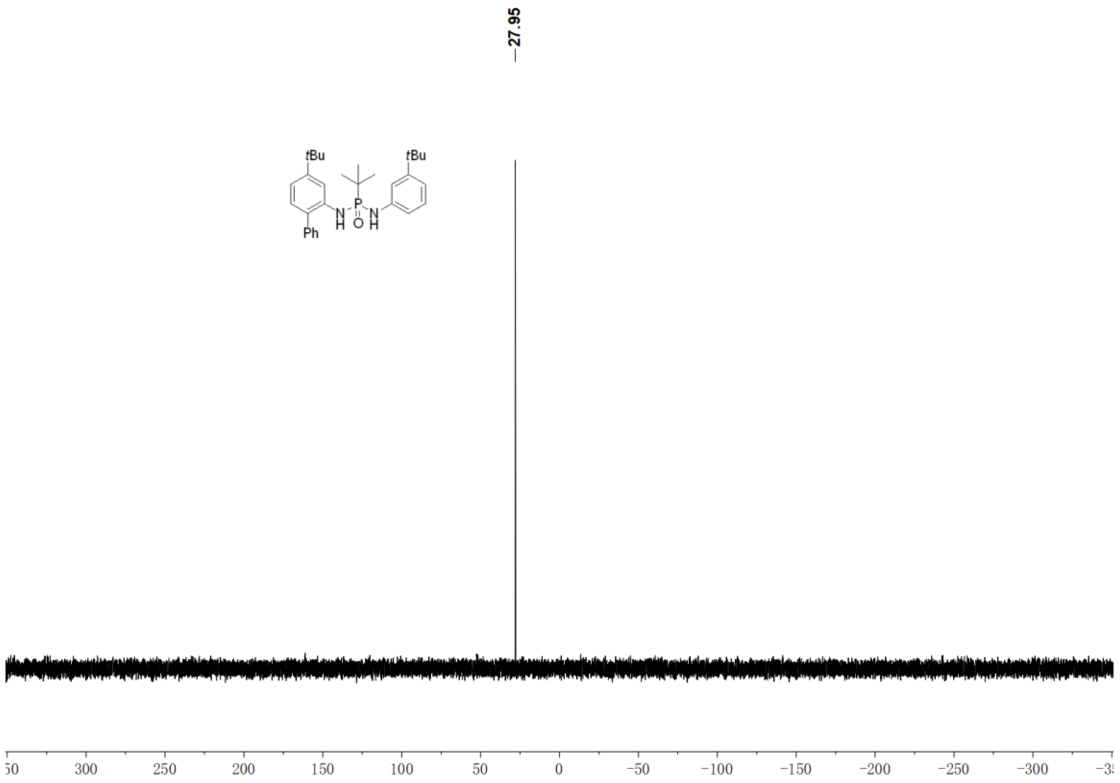

Supplementary Figure 52. ^{31}P NMR spectrum of **b2**

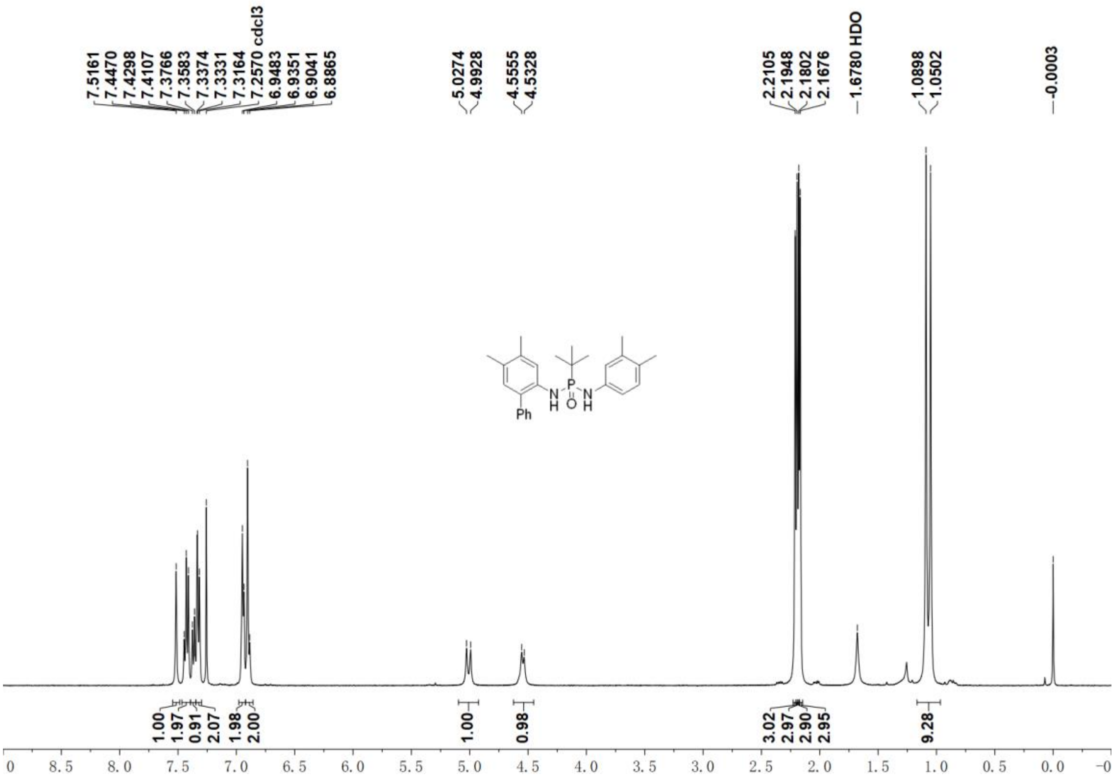

Supplementary Figure 53. ^{1}H NMR spectrum of **b3**

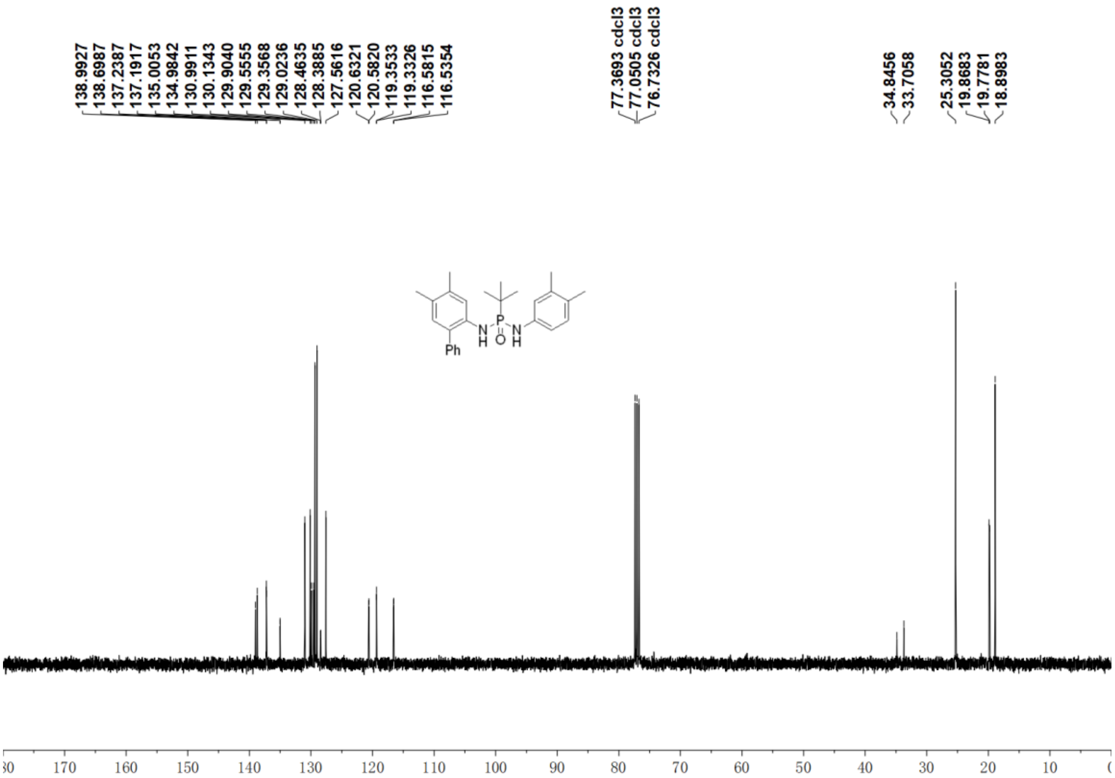

Supplementary Figure 54. ^{13}C NMR spectrum of **b3**

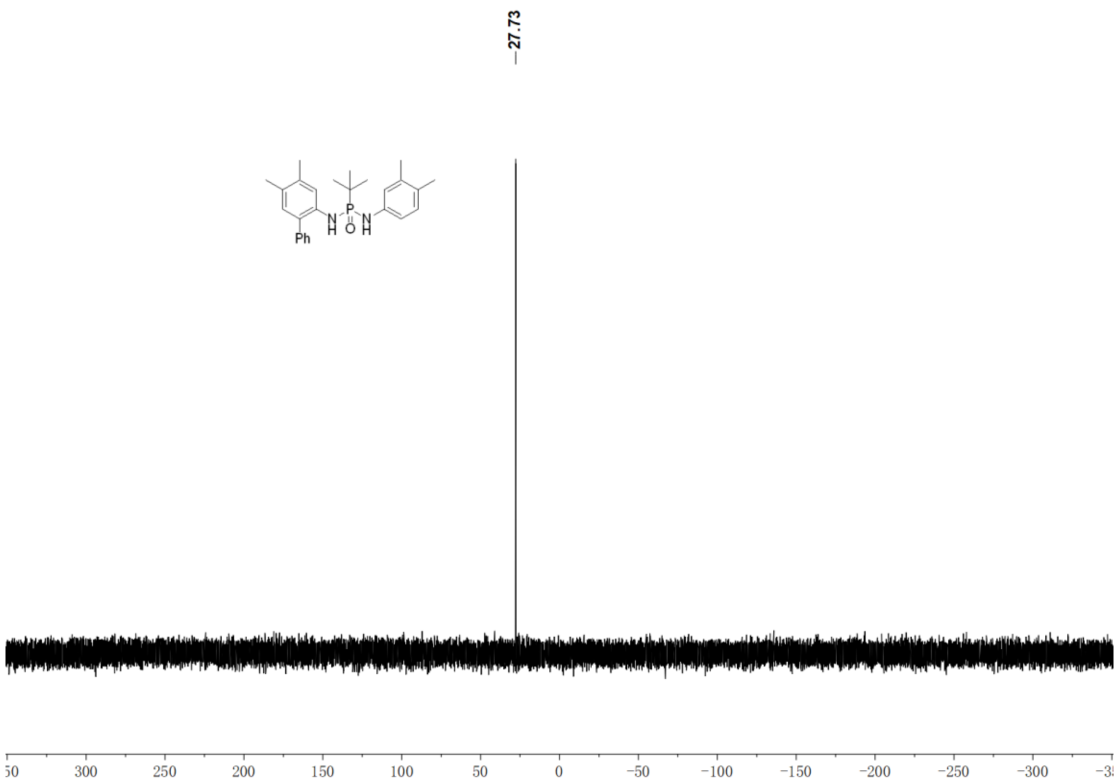

Supplementary Figure 55. ^{31}P NMR spectrum of **b3**

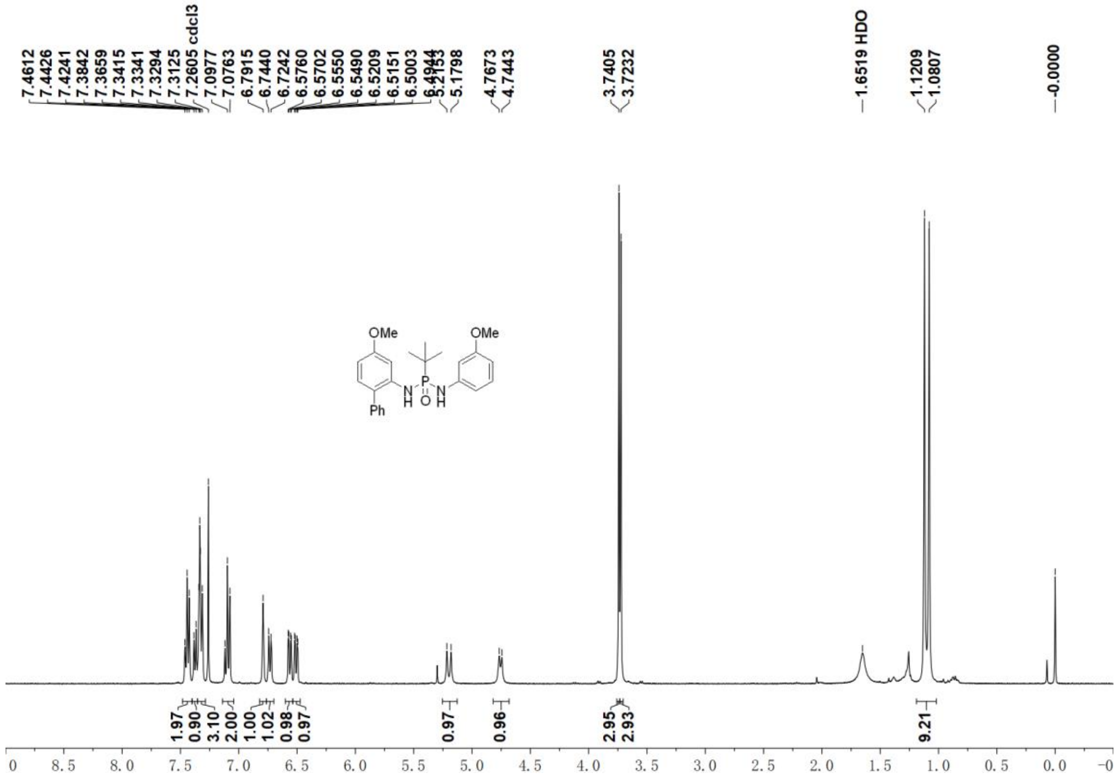

Supplementary Figure 56. ^{1}H NMR spectrum of **b4**

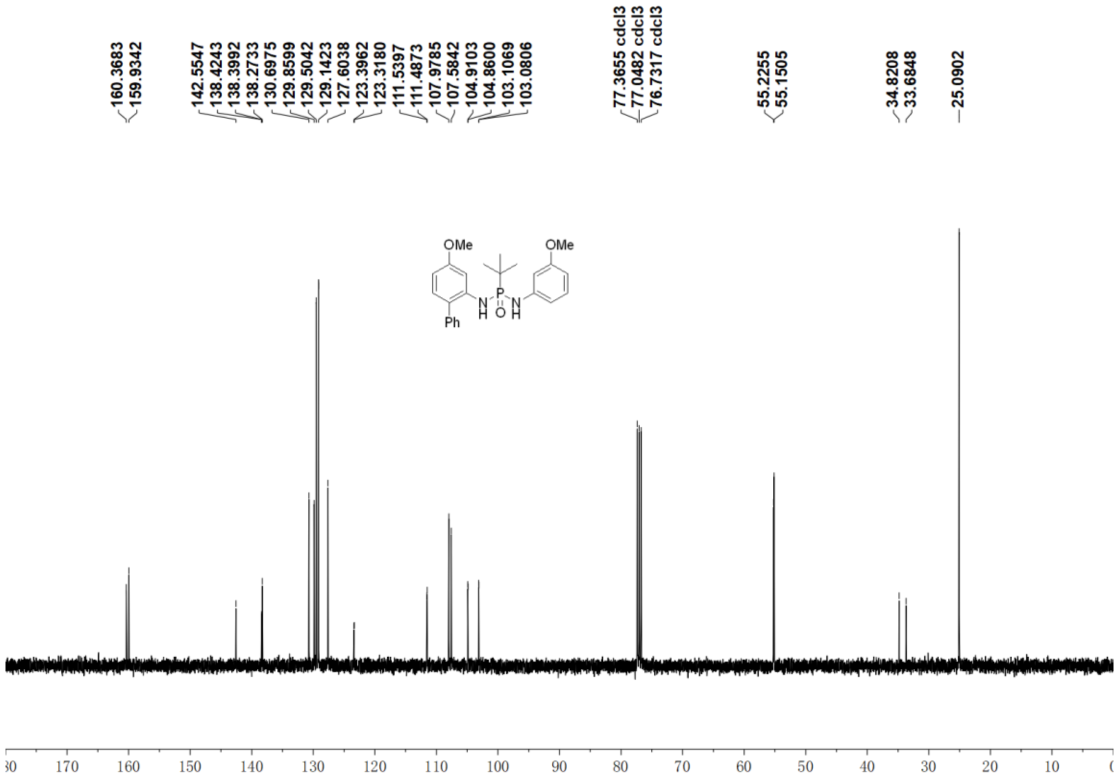

Supplementary Figure 57. ^{13}C NMR spectrum of **b4**

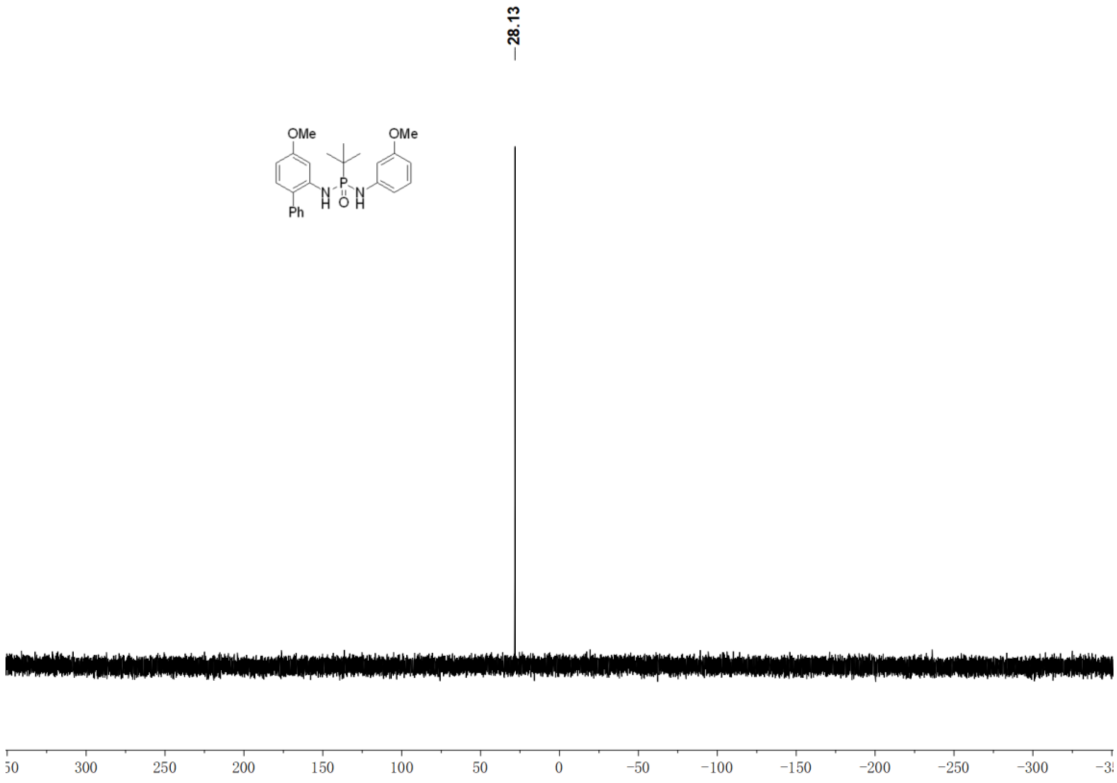

Supplementary Figure 58. ^{31}P NMR spectrum of **b4**

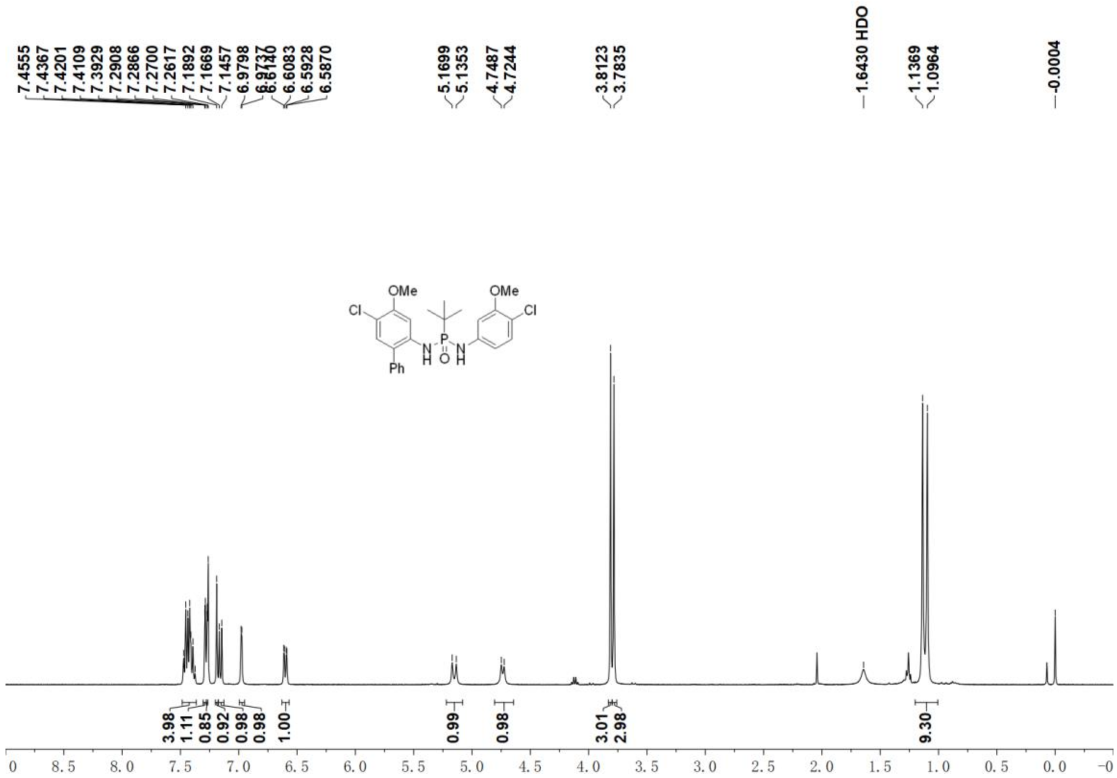

Supplementary Figure 59. ^{1}H NMR spectrum of **b5**

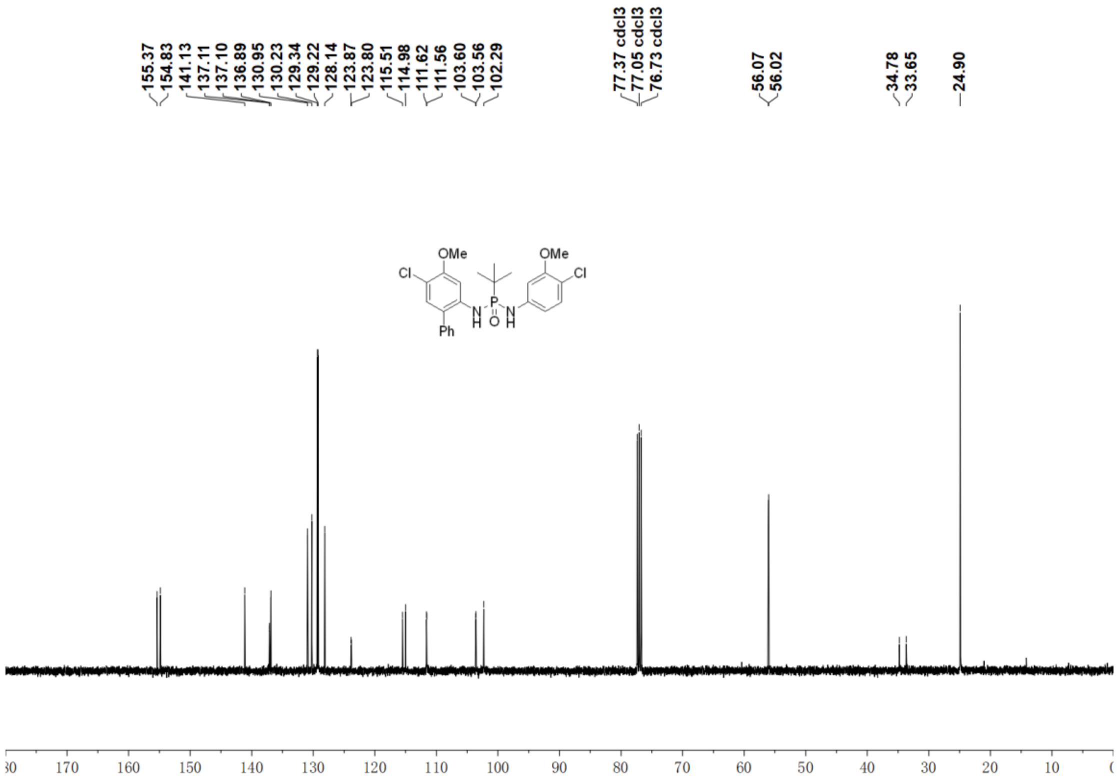

Supplementary Figure 60. ^{13}C NMR spectrum of **b5**

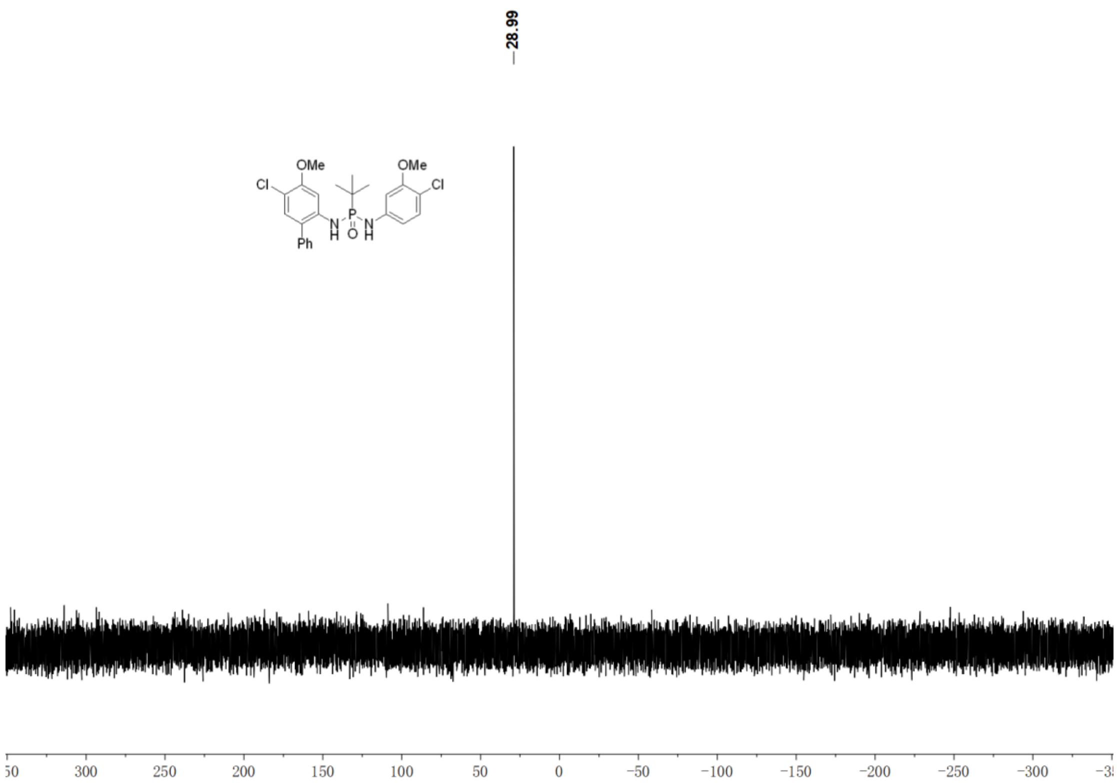

Supplementary Figure 61. ^{31}P NMR spectrum of **b5**

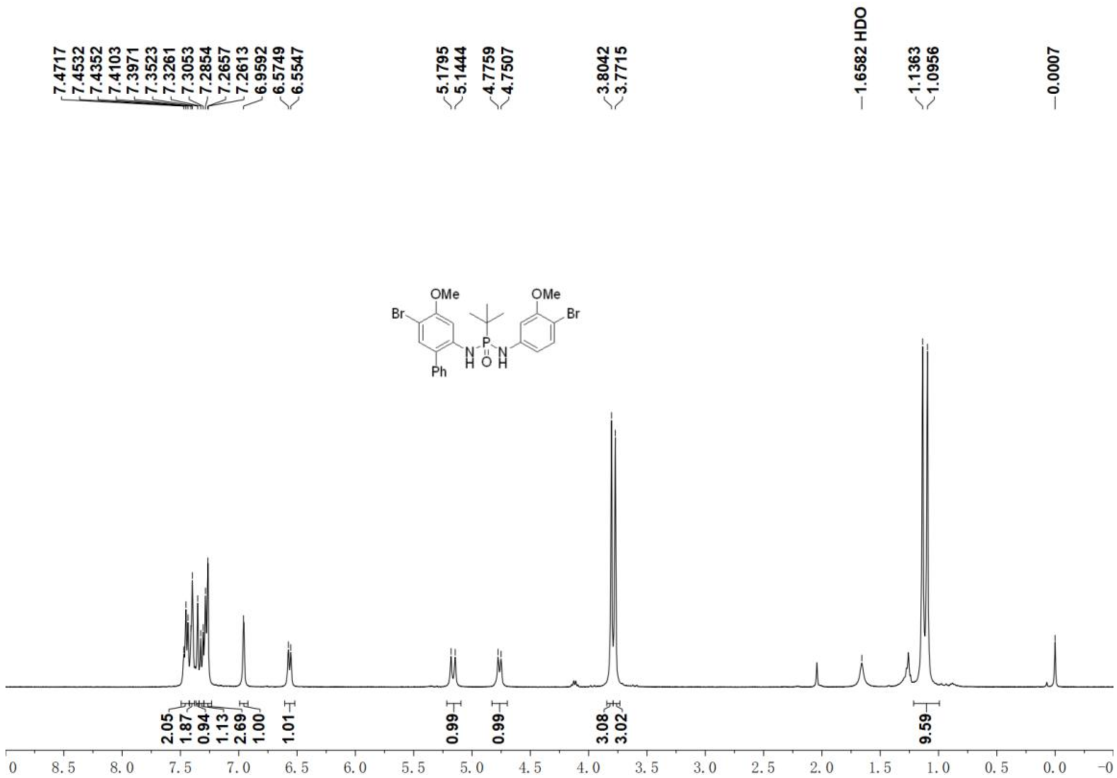

Supplementary Figure 62. ^{1}H NMR spectrum of **b6**

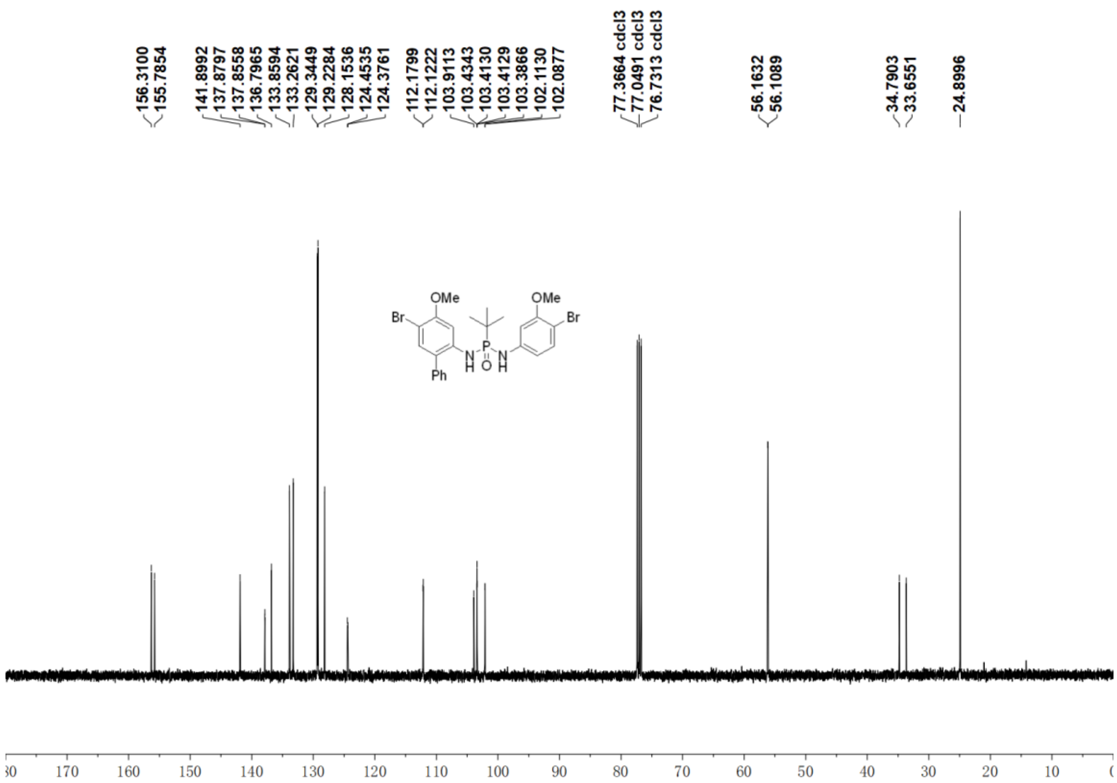

Supplementary Figure 63. ^{13}C NMR spectrum of **b6**

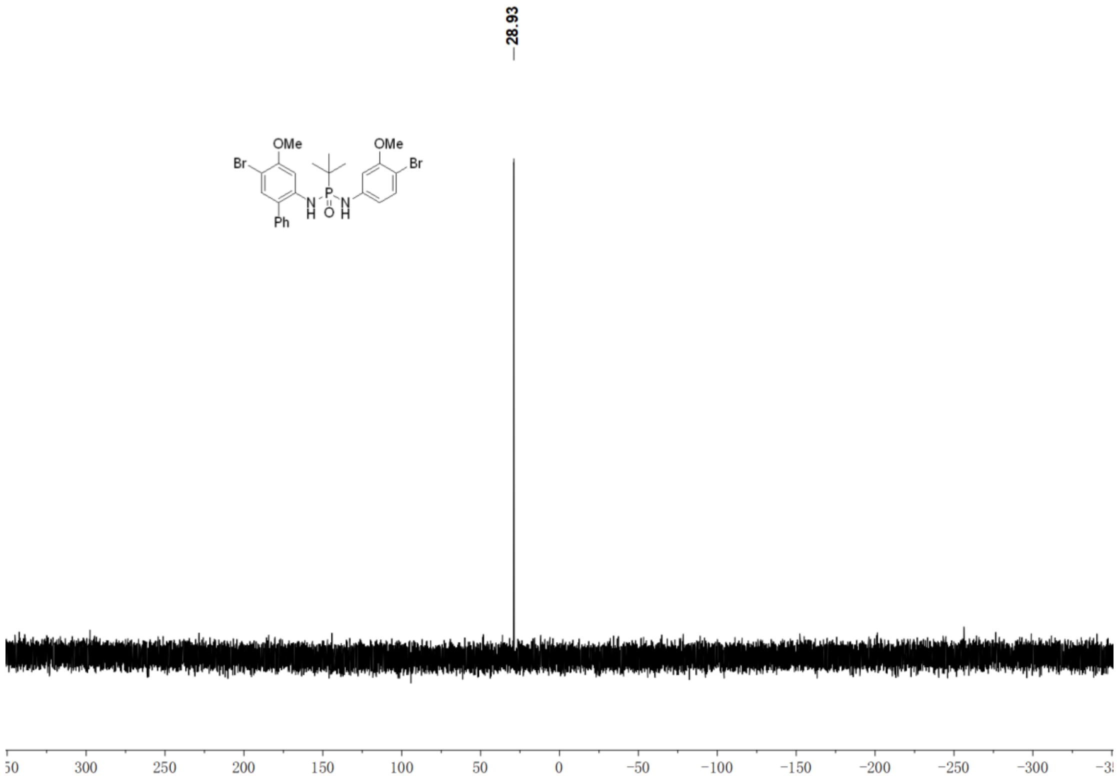

Supplementary Figure 64. ^{31}P NMR spectrum of **b6**

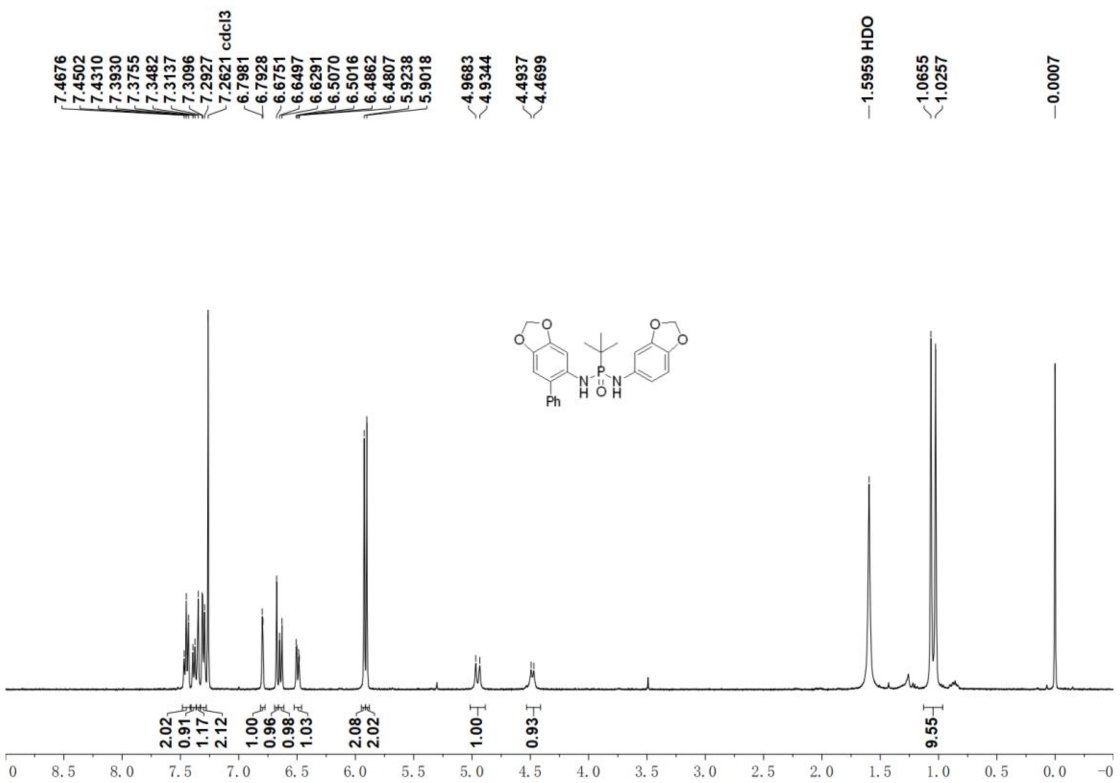

Supplementary Figure 65. ^{1}H NMR spectrum of **b7**

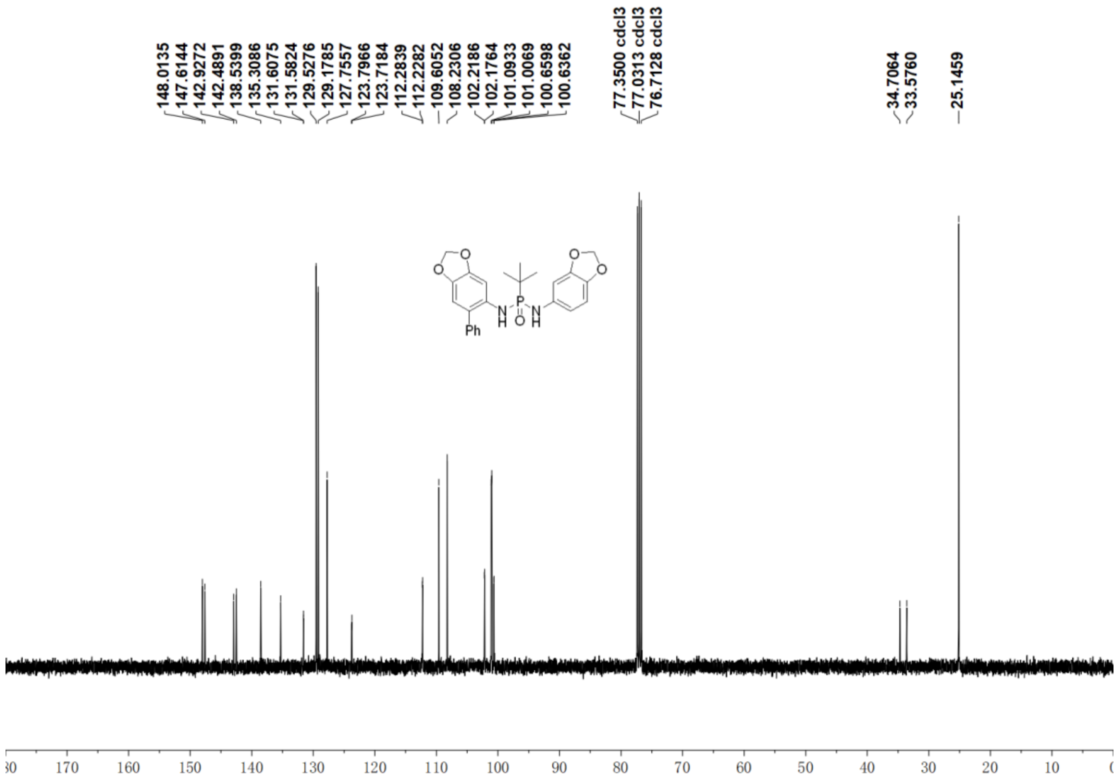

Supplementary Figure 66. ^{13}C NMR spectrum of **b7**

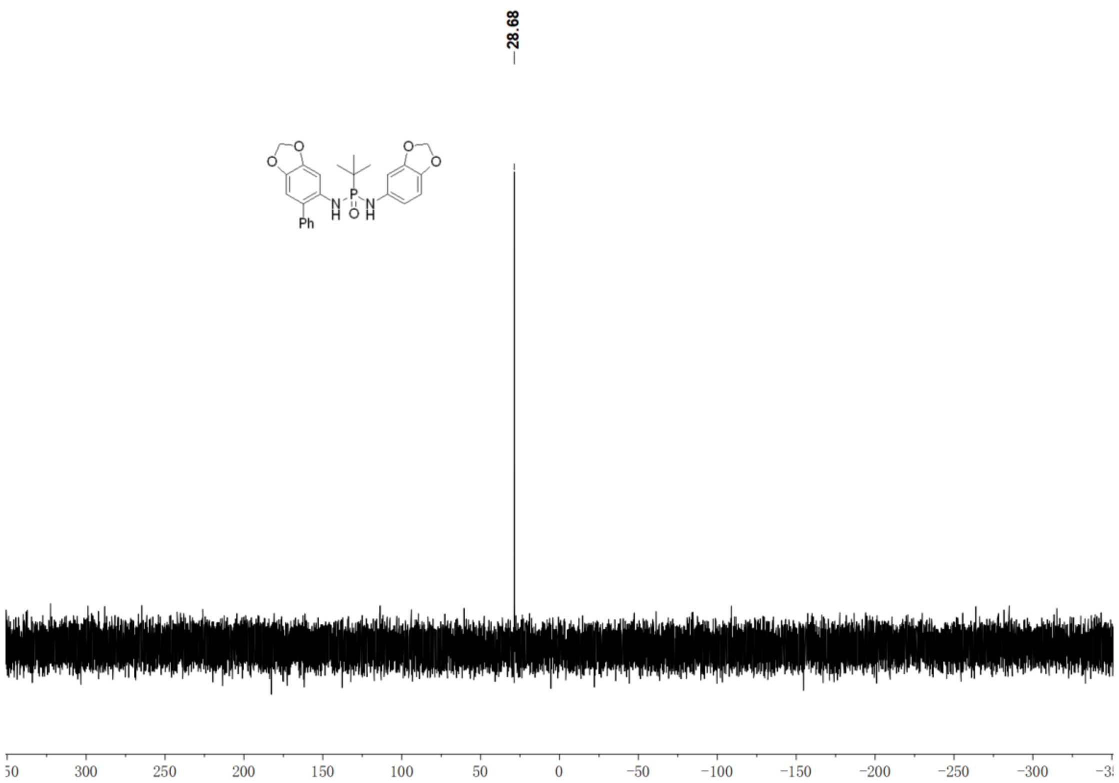

Supplementary Figure 67. ^{31}P NMR spectrum of **b7**

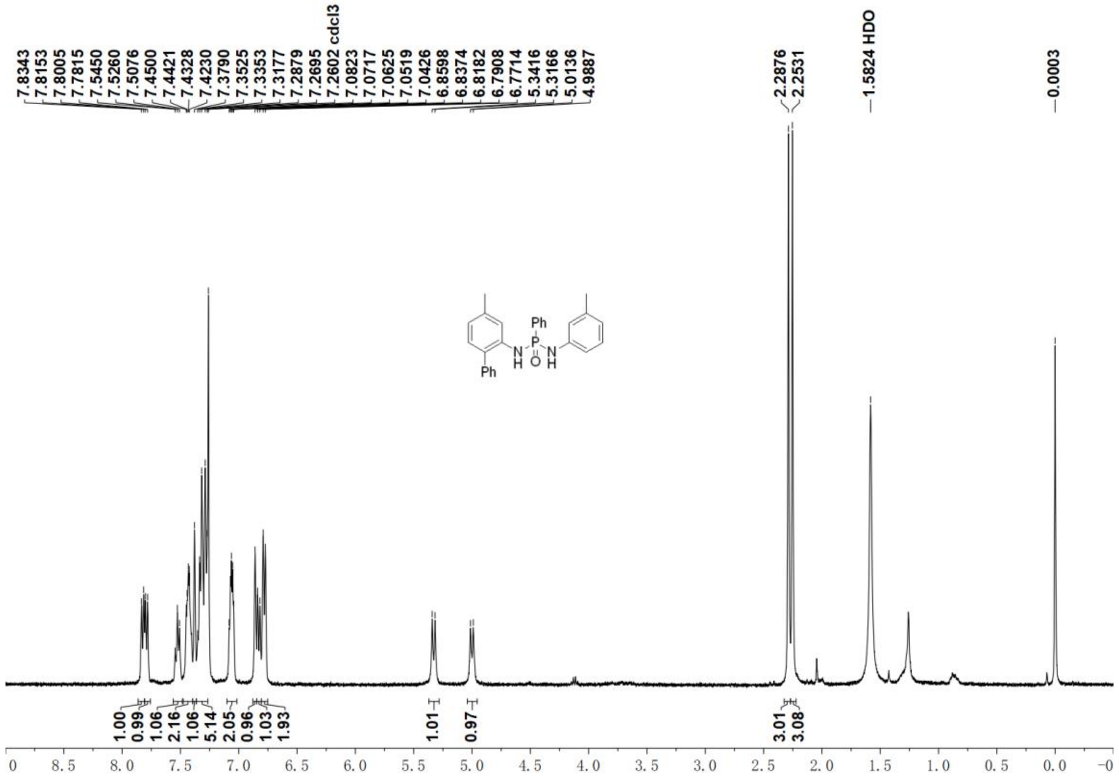

Supplementary Figure 68. ^{1}H NMR spectrum of **b8**

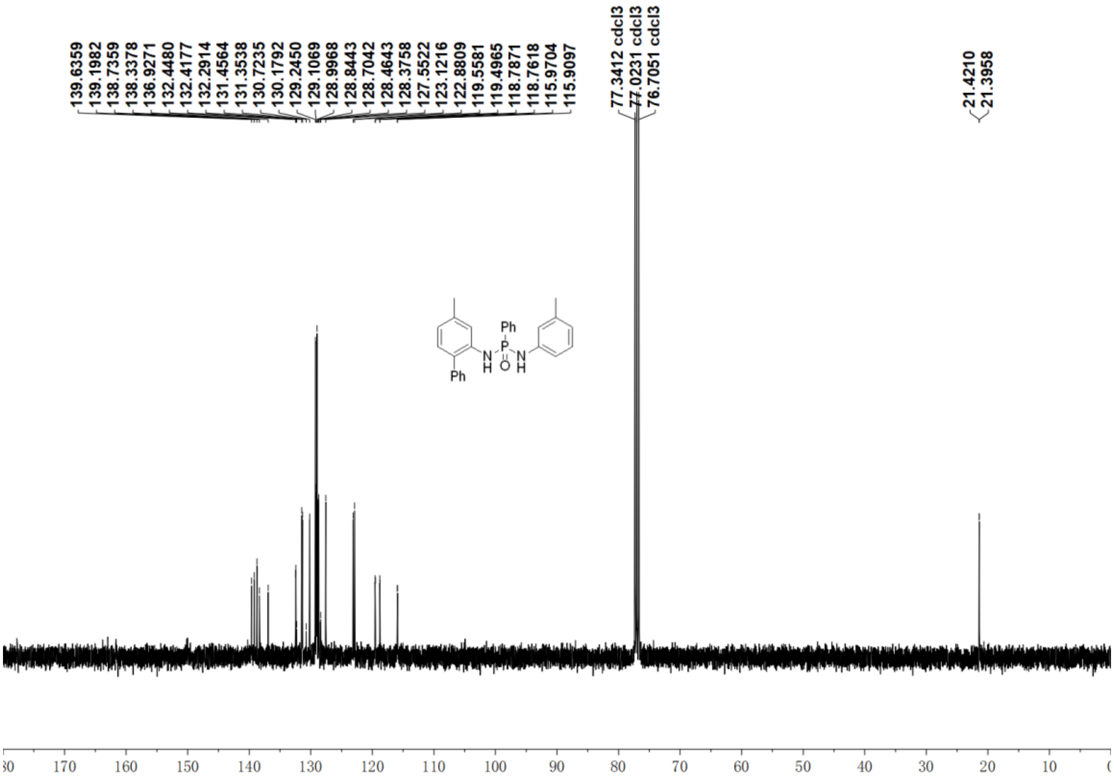

Supplementary Figure 69. ^{13}C NMR spectrum of **b8**

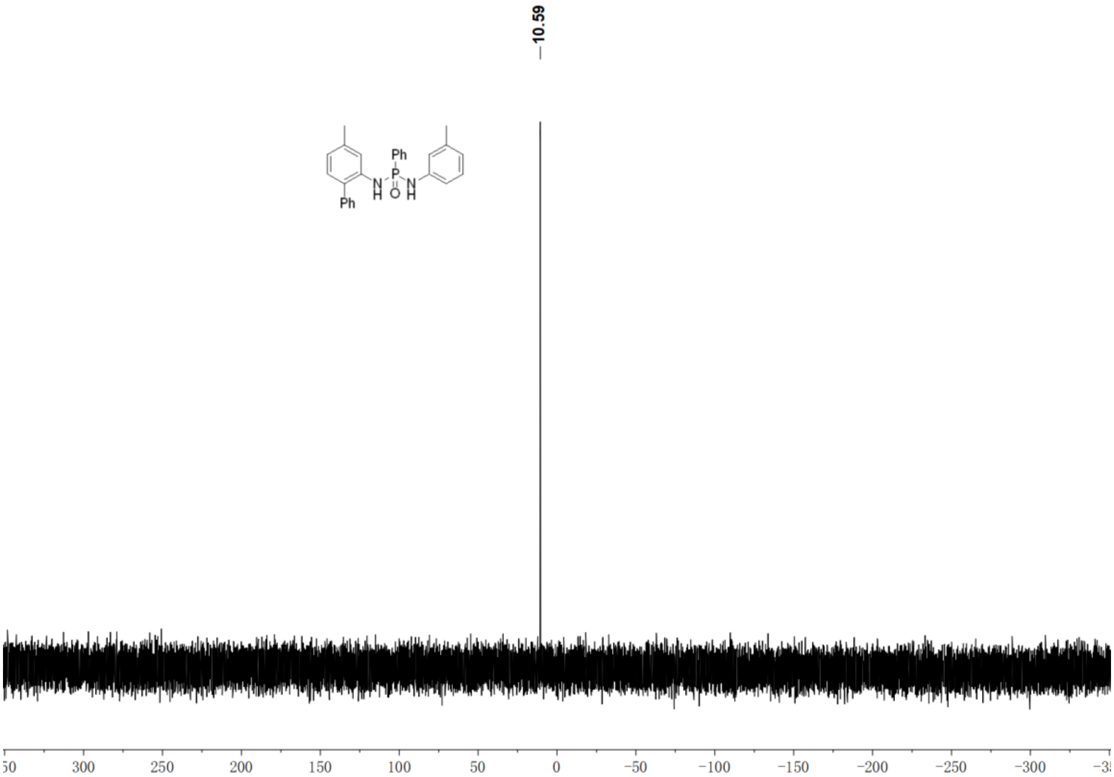

Supplementary Figure 70. ^{31}P NMR spectrum of **b8**

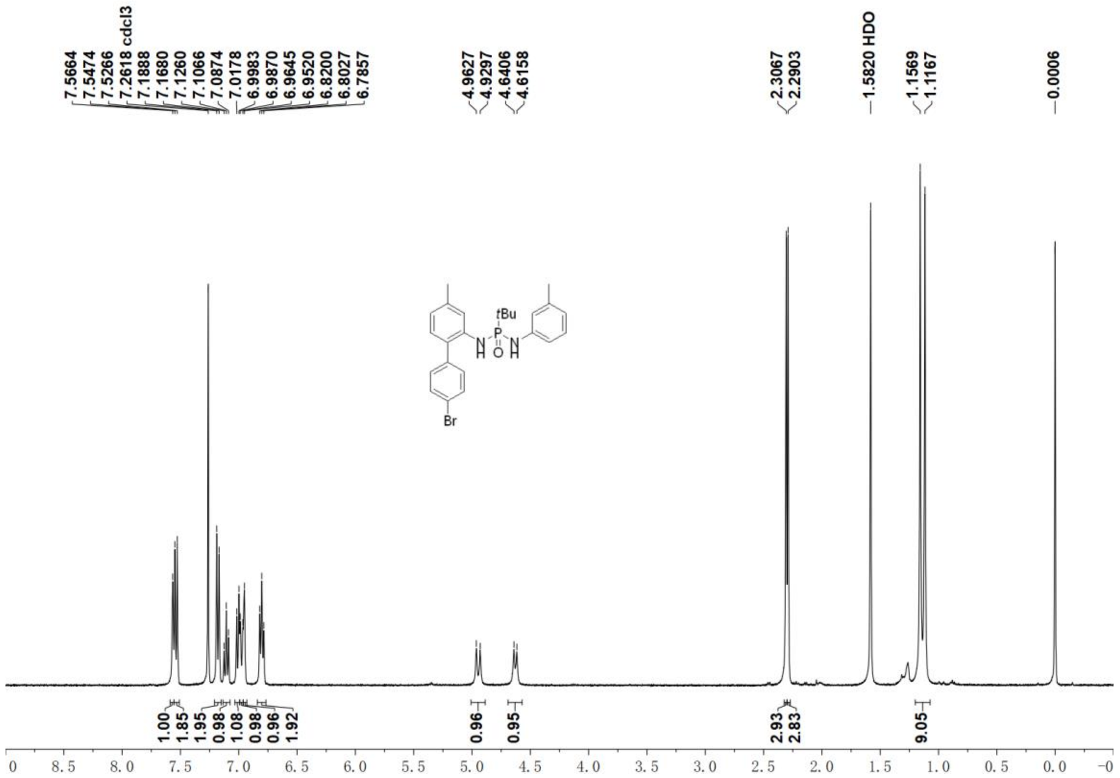

Supplementary Figure 71. ^{1}H NMR spectrum of **b9**

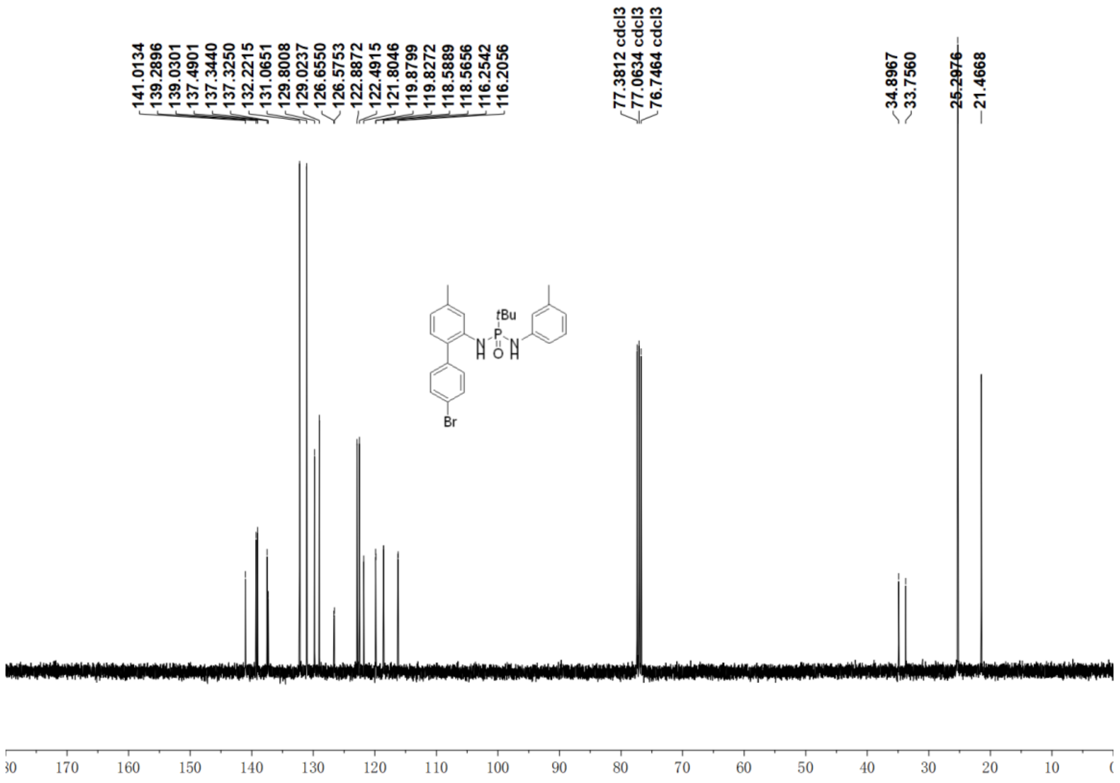

Supplementary Figure 72. ^{13}C NMR spectrum of **b9**

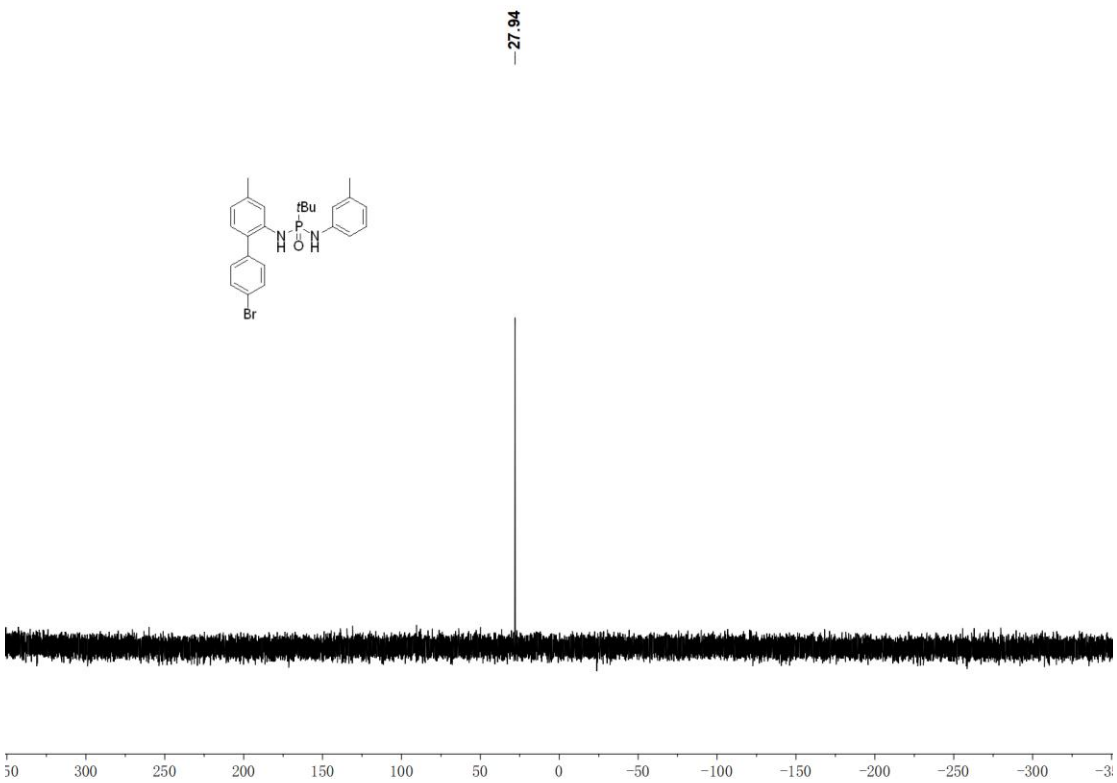

Supplementary Figure 73. ^{31}P NMR spectrum of **b9**

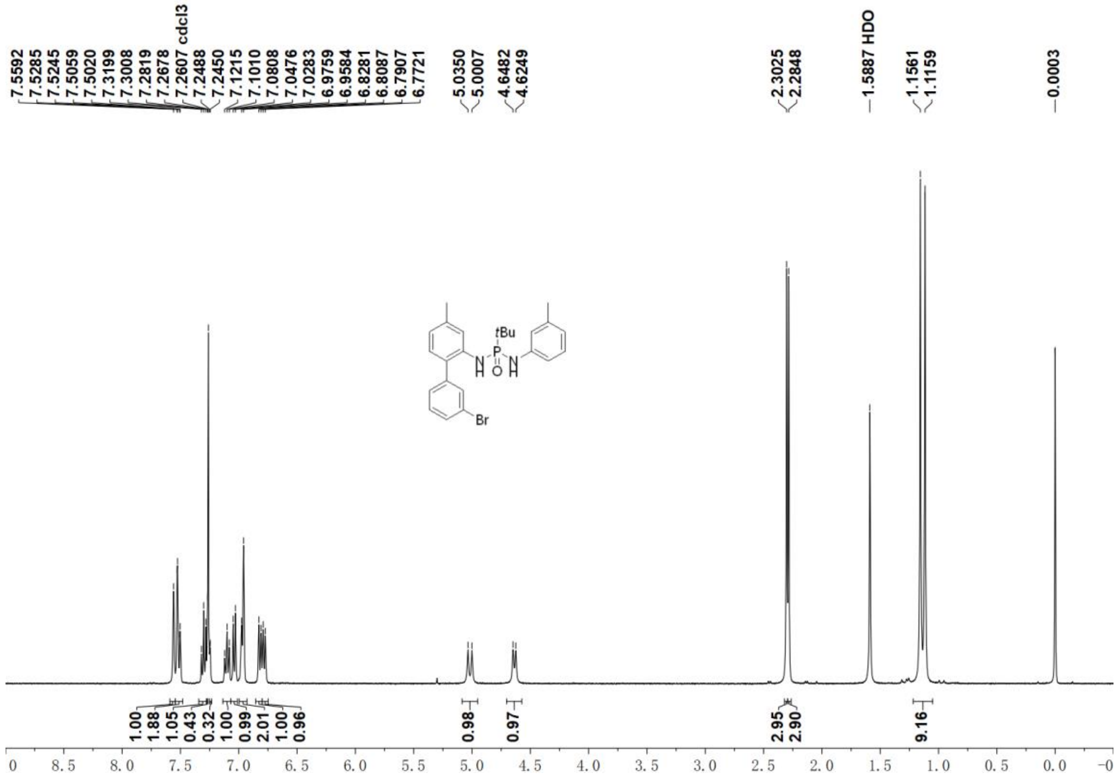

Supplementary Figure 74. ^{1}H NMR spectrum of **b10**

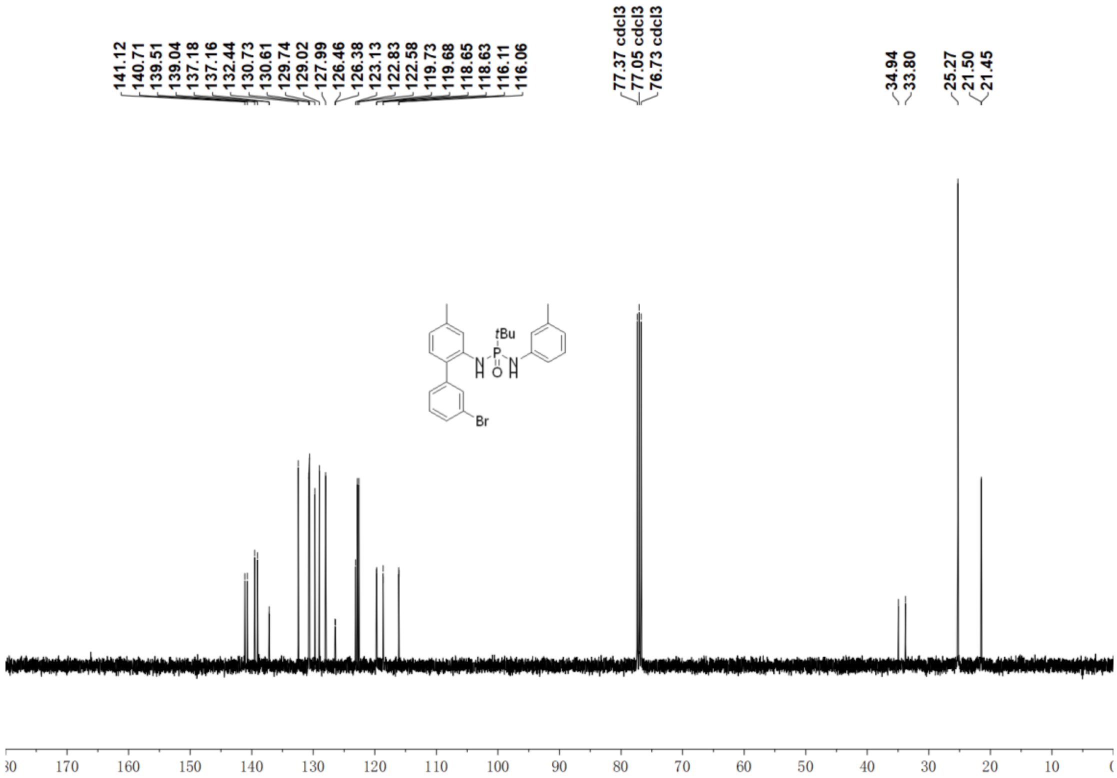

Supplementary Figure 75. ^{13}C NMR spectrum of **b10**

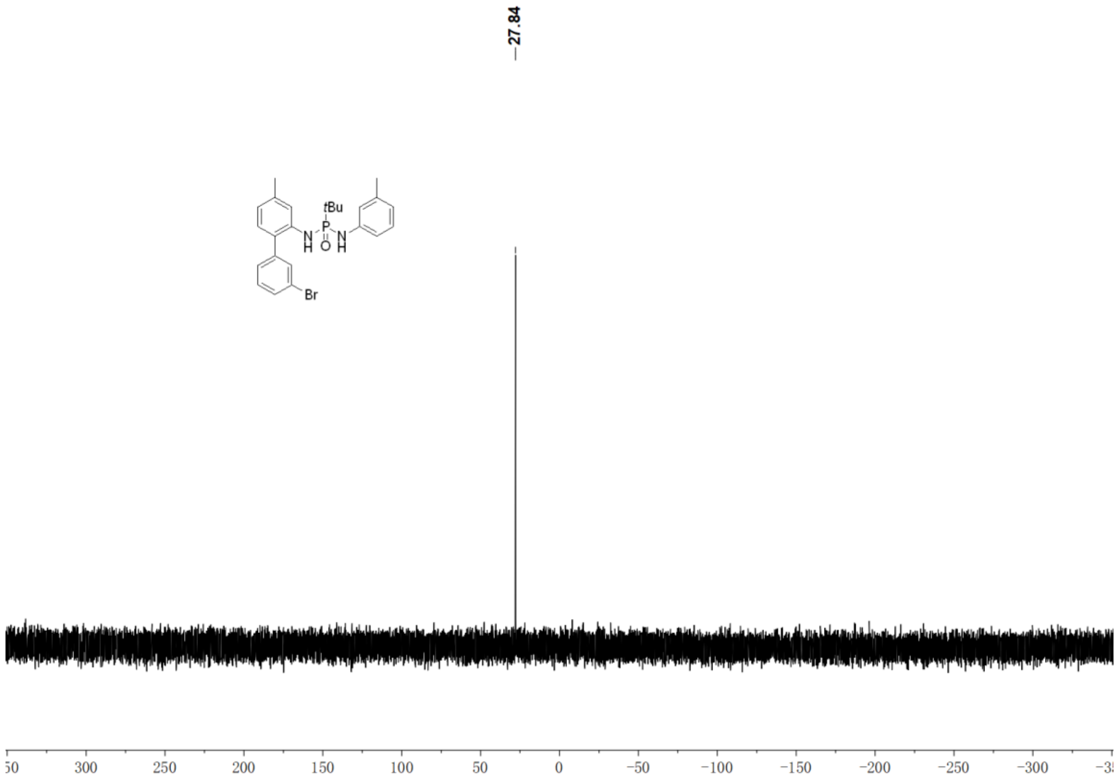

Supplementary Figure 76. ^{31}P NMR spectrum of **b10**

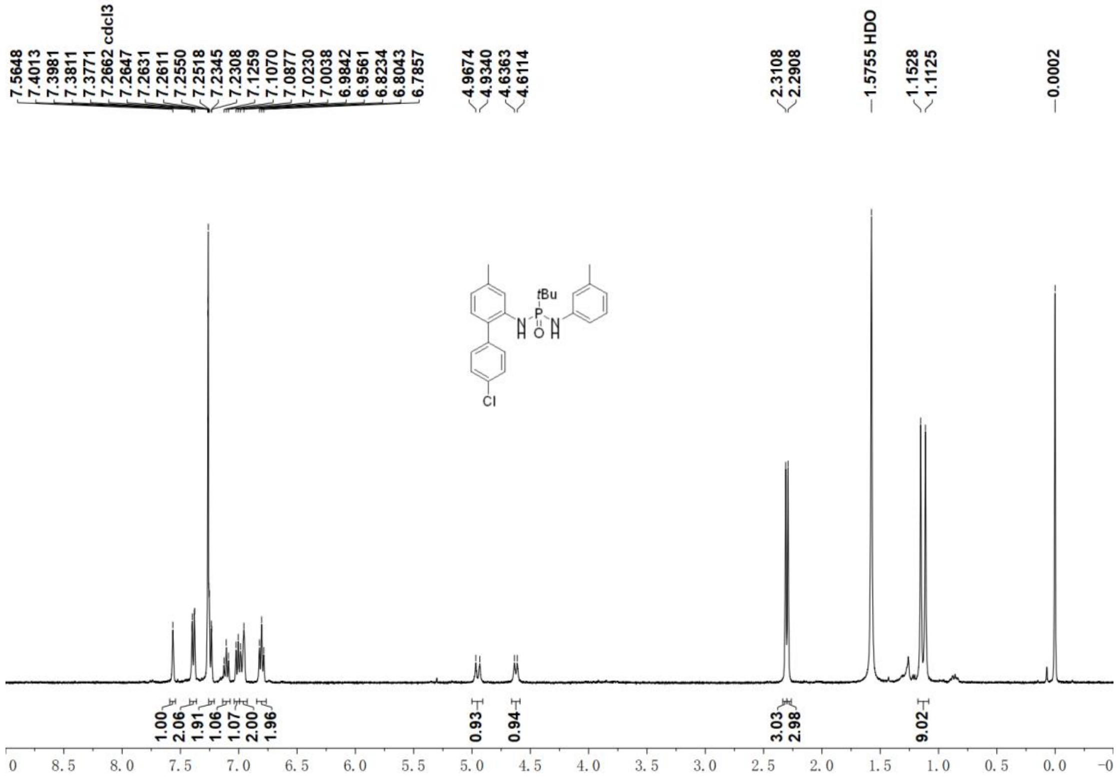

Supplementary Figure 77. ^{1}H NMR spectrum of **b11**

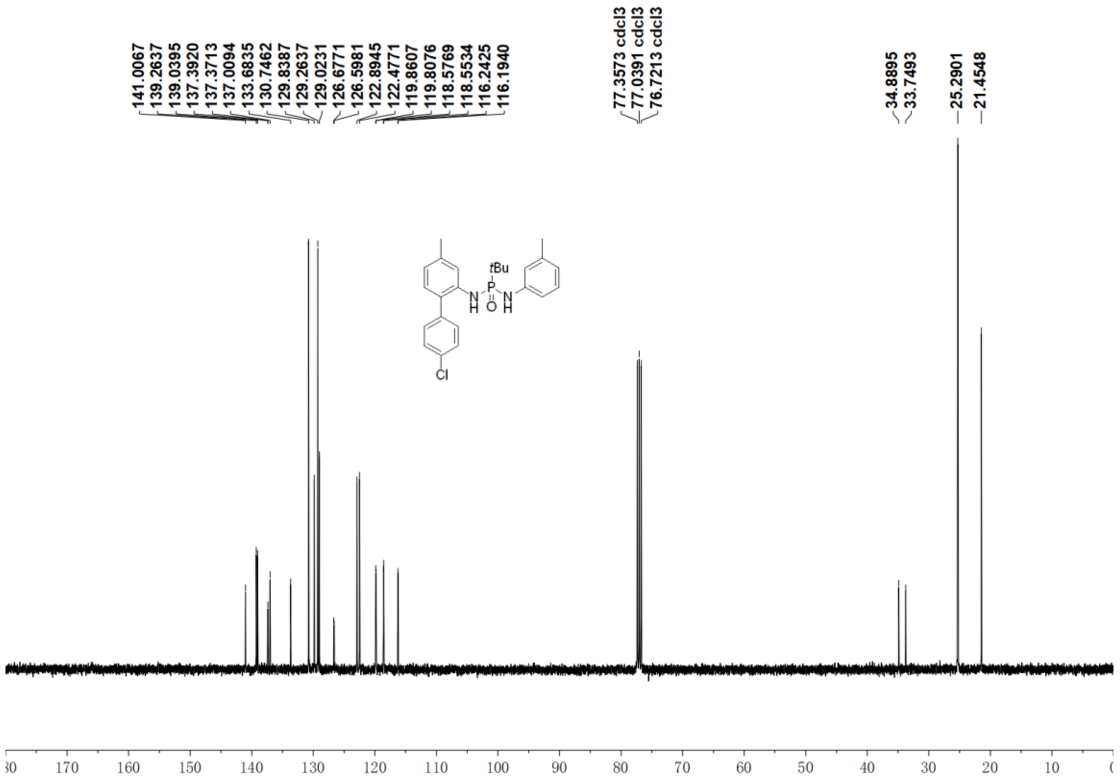

Supplementary Figure 78. ^{13}C NMR spectrum of **b11**

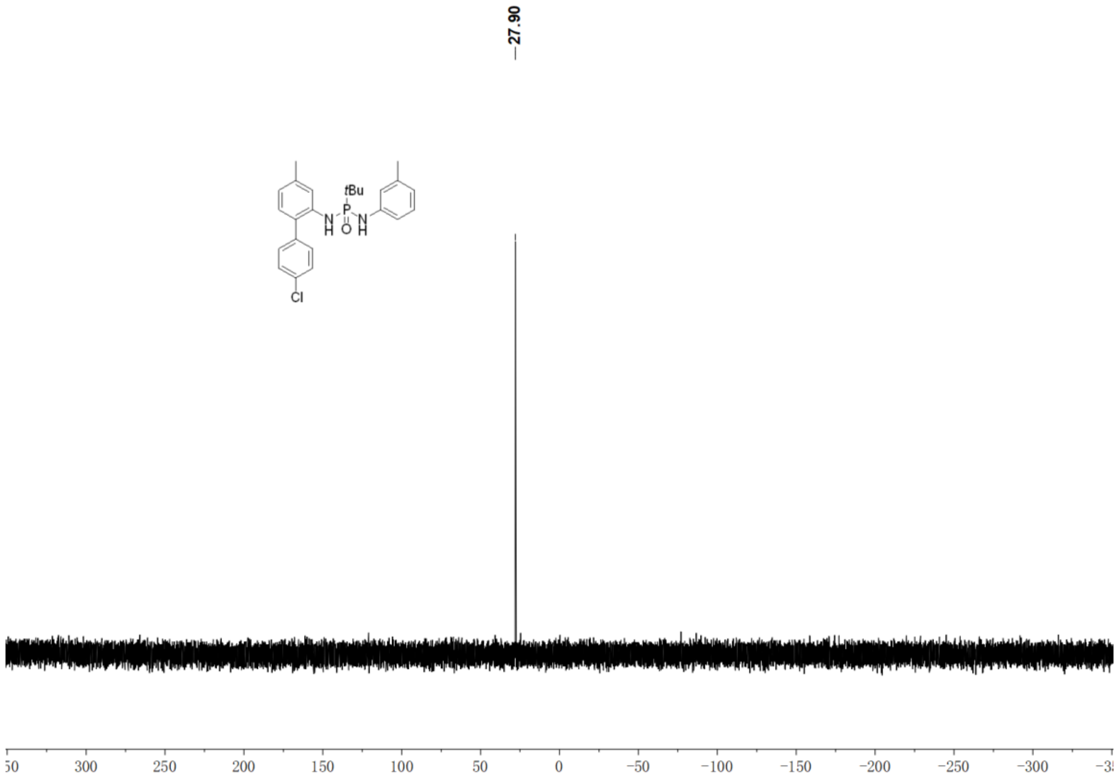

Supplementary Figure 79. ^{31}P NMR spectrum of **b11**

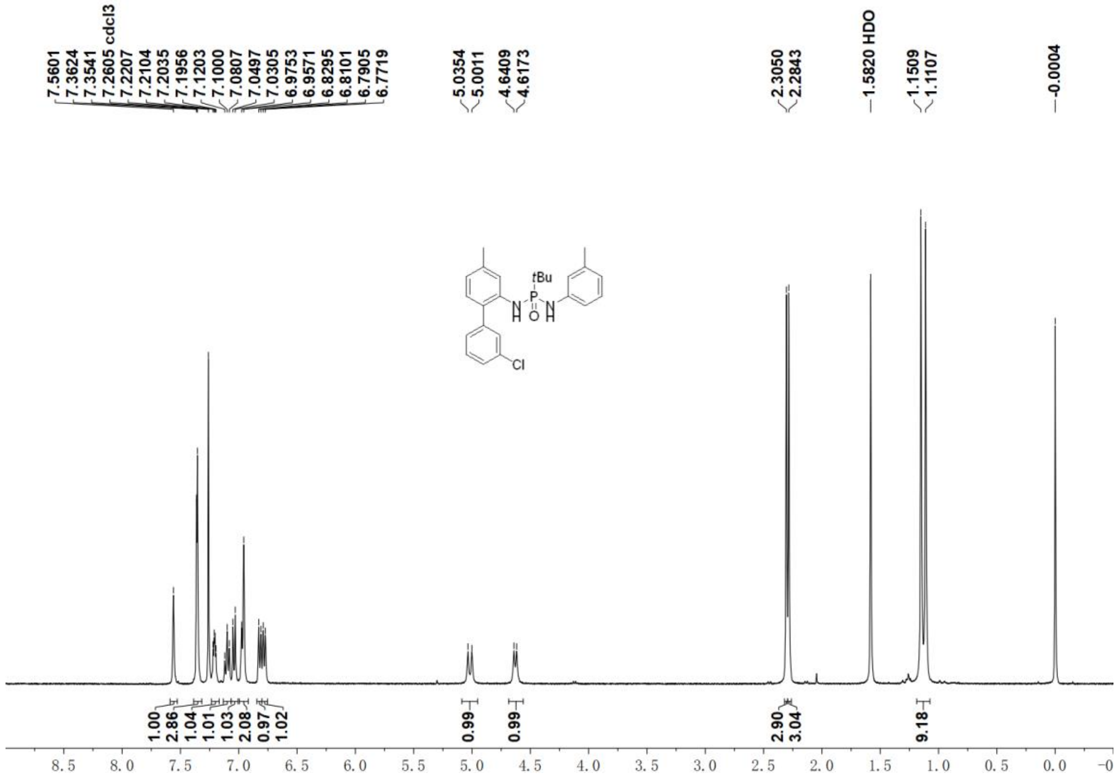

Supplementary Figure 80. ^{1}H NMR spectrum of **b12**

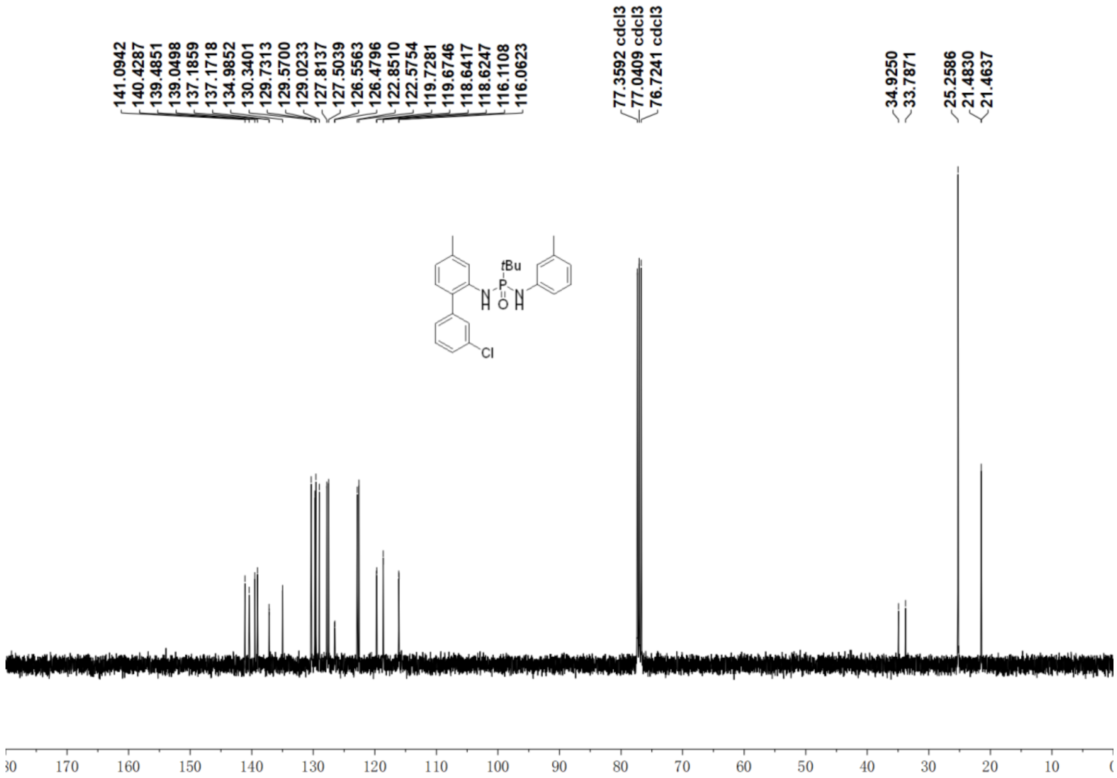

Supplementary Figure 81. ^{13}C NMR spectrum of **b12**

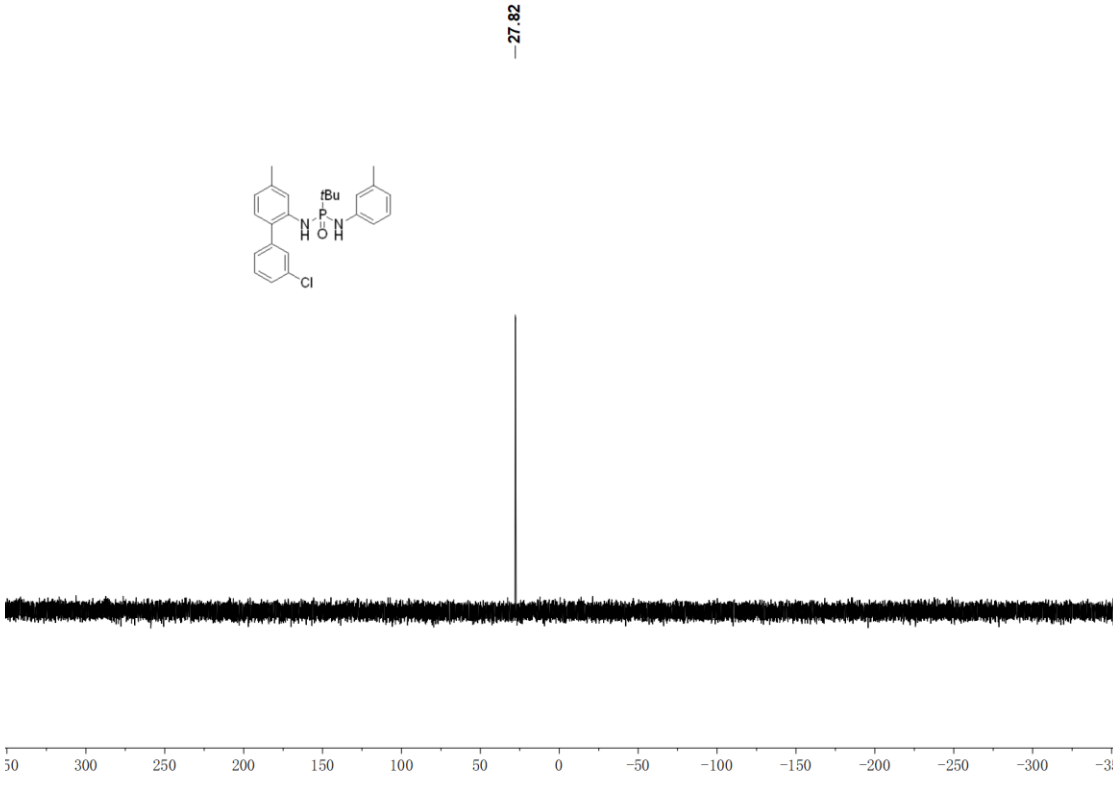

Supplementary Figure 82. ^{31}P NMR spectrum of **b12**

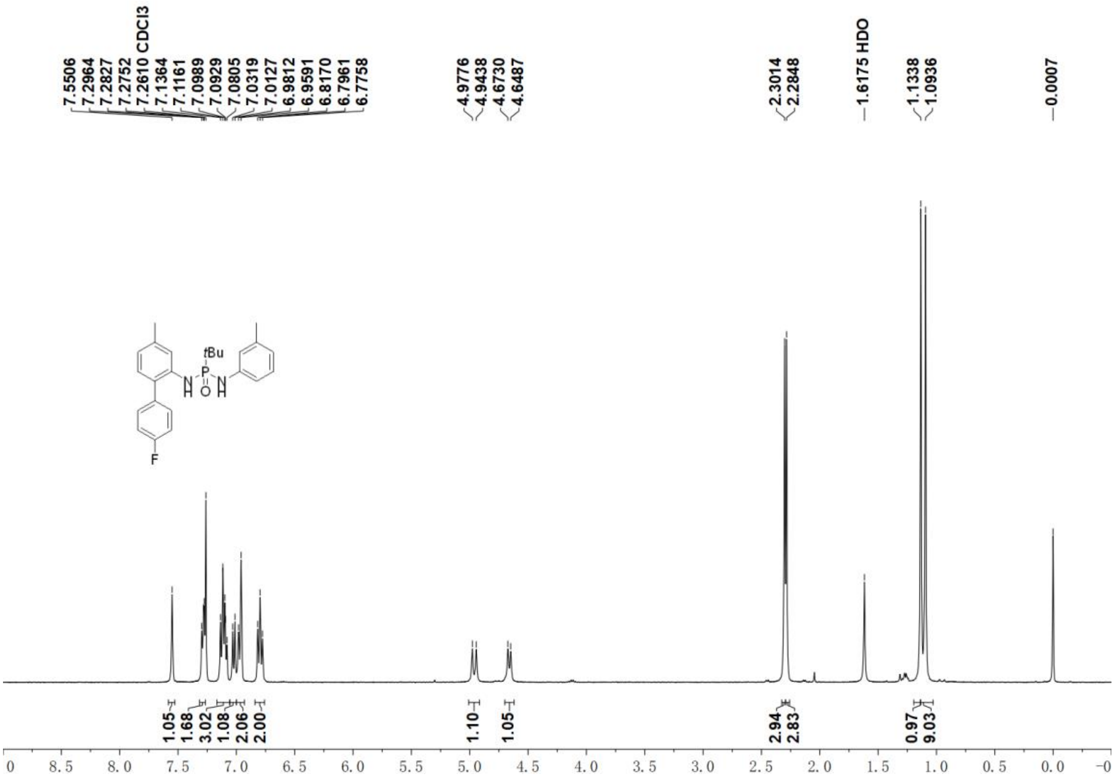

Supplementary Figure 83. ^{1}H NMR spectrum of **b13**

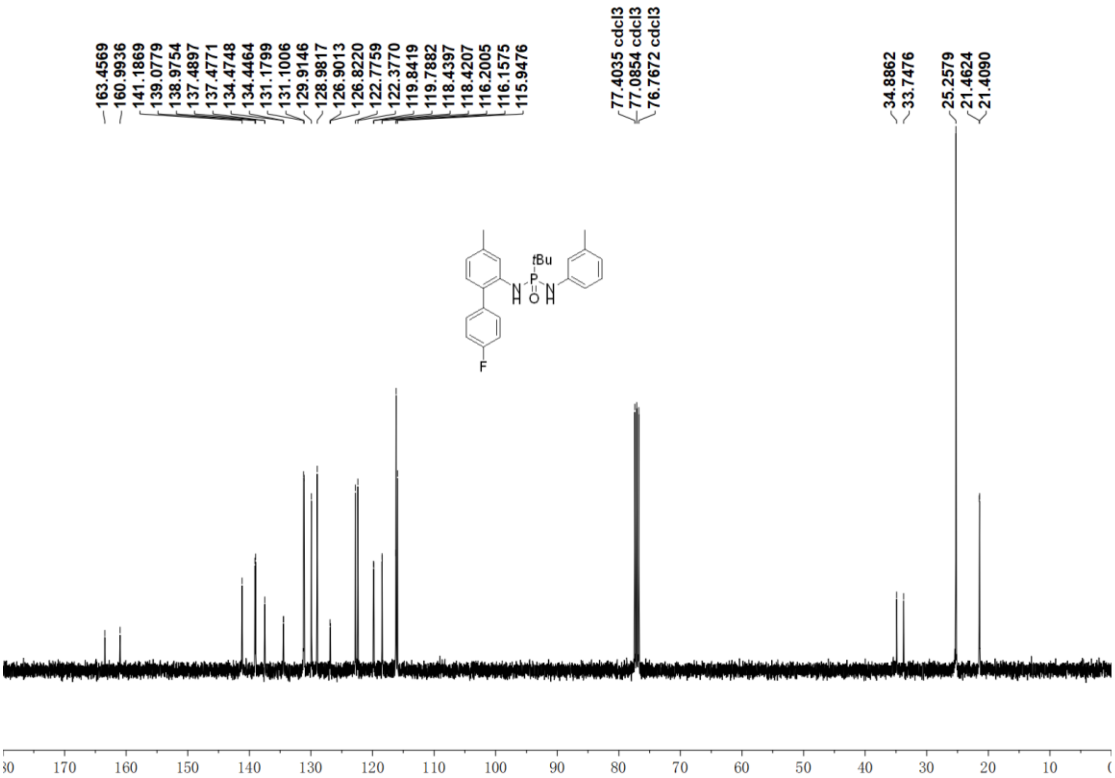

Supplementary Figure 84. ^{13}C NMR spectrum of **b13**

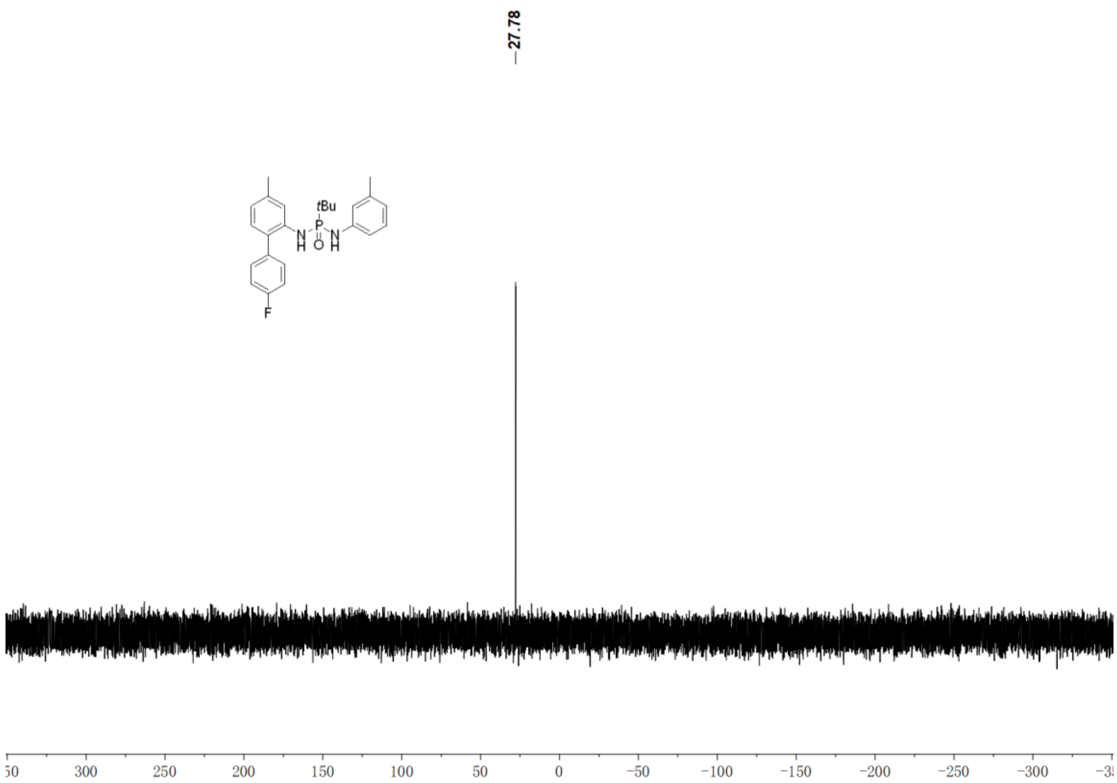

Supplementary Figure 85. ^{31}P NMR spectrum of **b13**

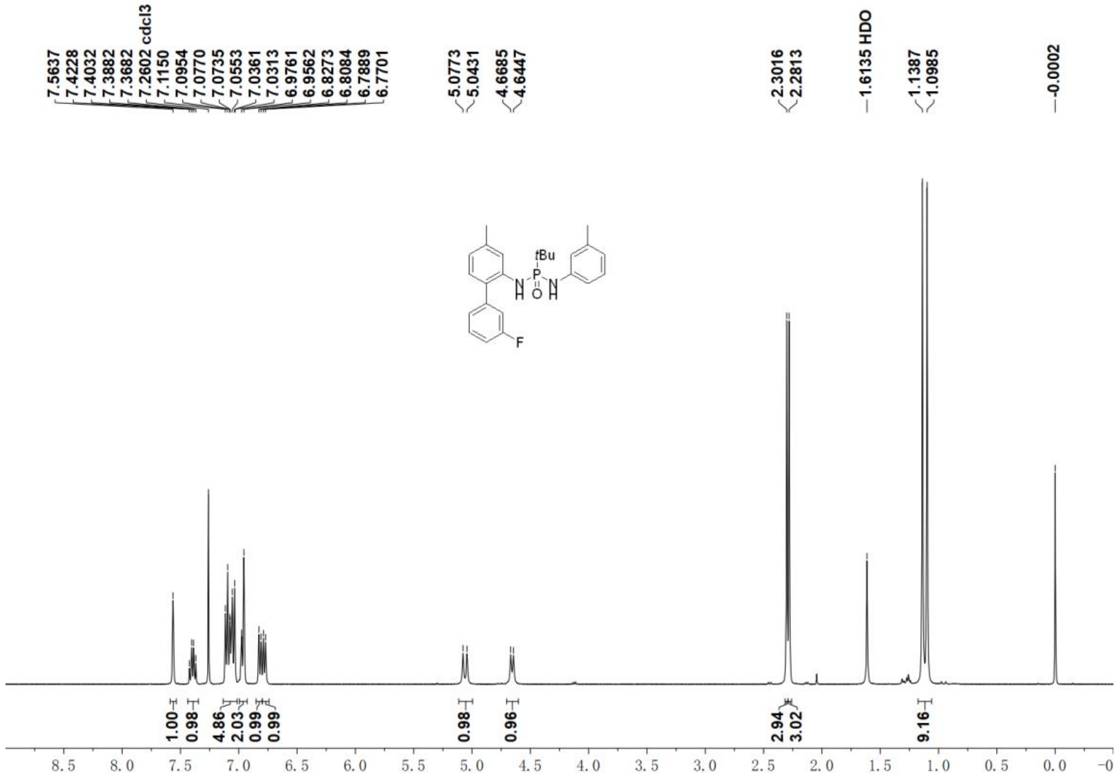

Supplementary Figure 86. ^{1}H NMR spectrum of **b14**

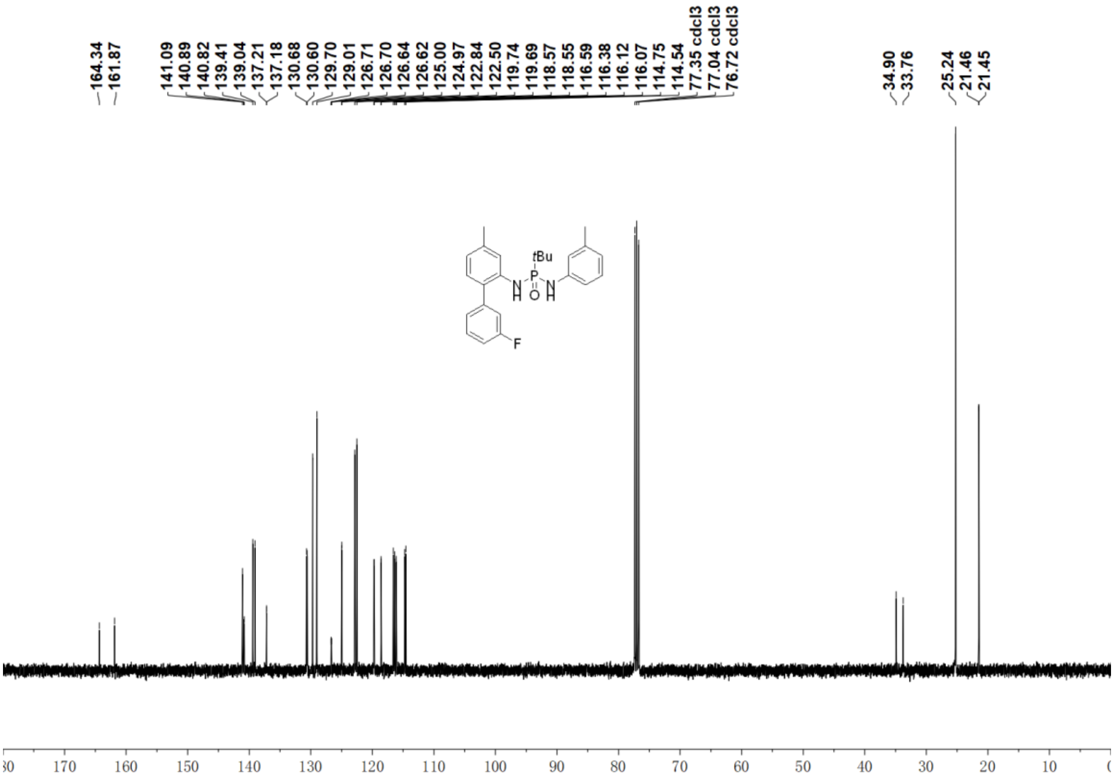

Supplementary Figure 87. ^{13}C NMR spectrum of **b14**

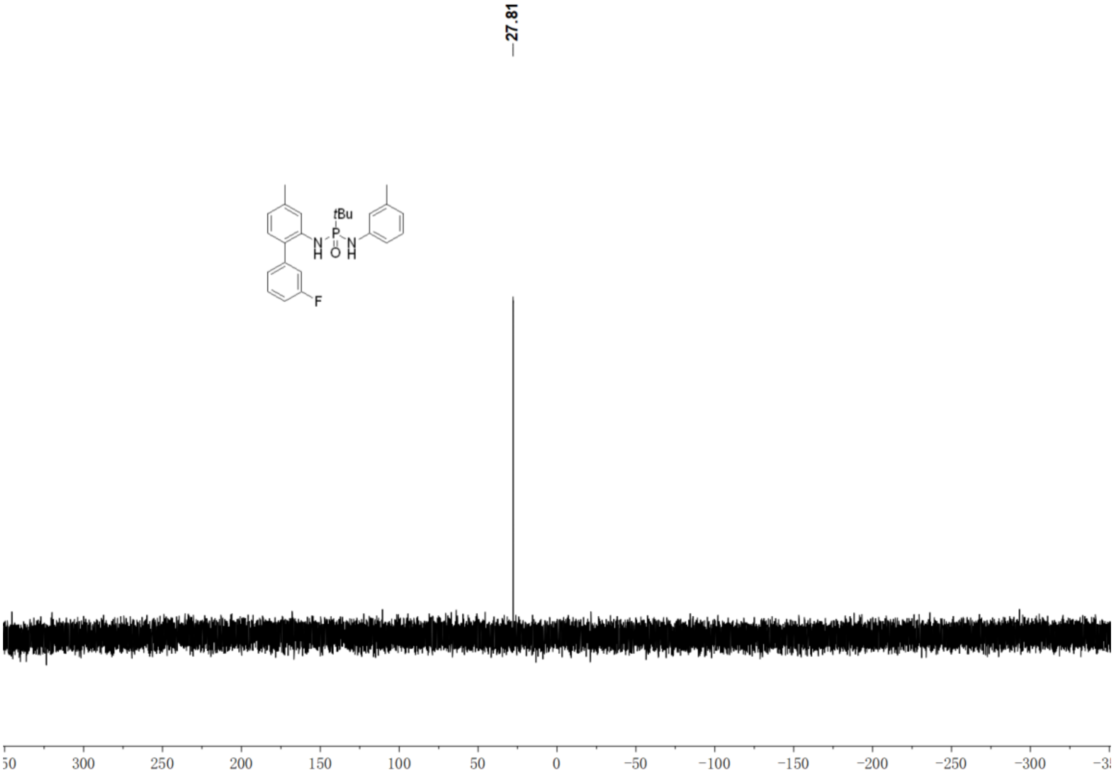

Supplementary Figure 88. ^{31}P NMR spectrum of **b14**

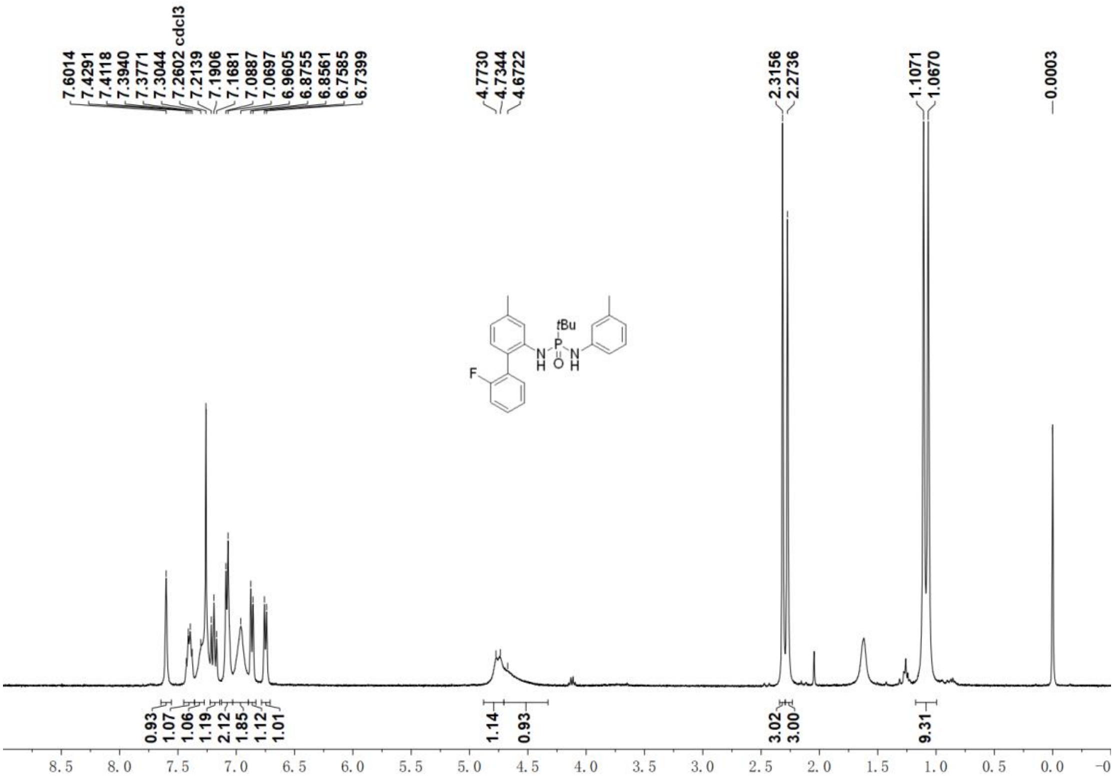

Supplementary Figure 89. ^{1}H NMR spectrum of **b15**

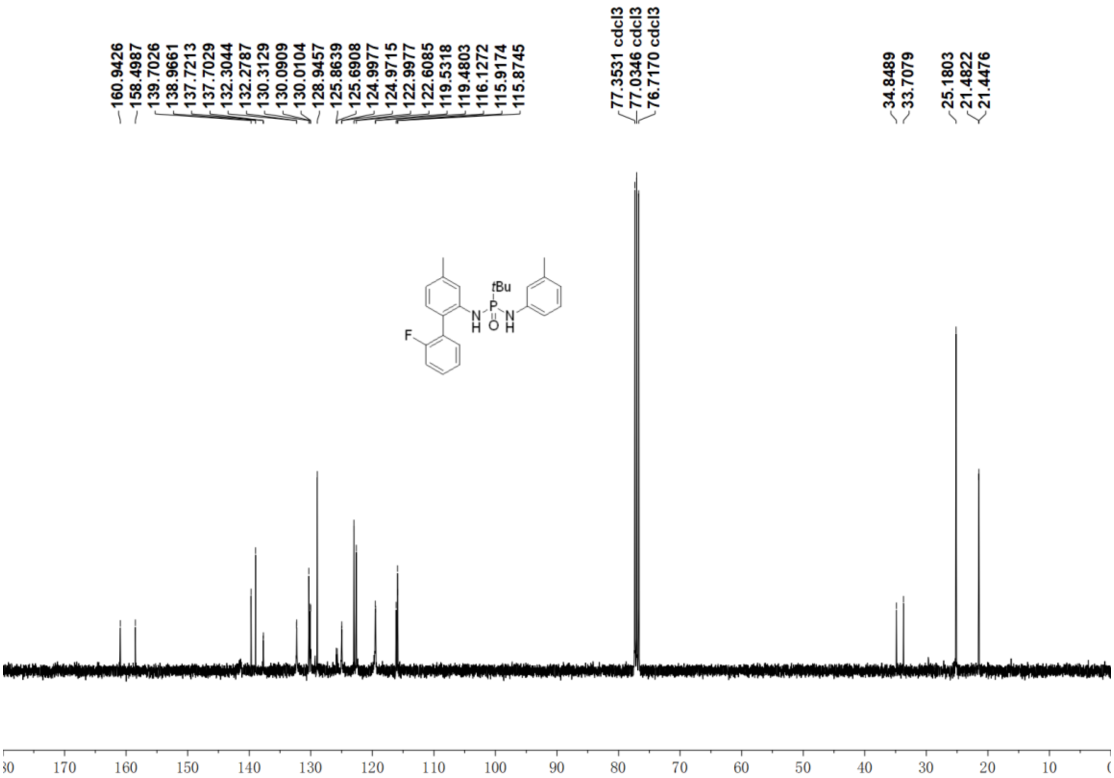

Supplementary Figure 90. ^{13}C NMR spectrum of **b15**

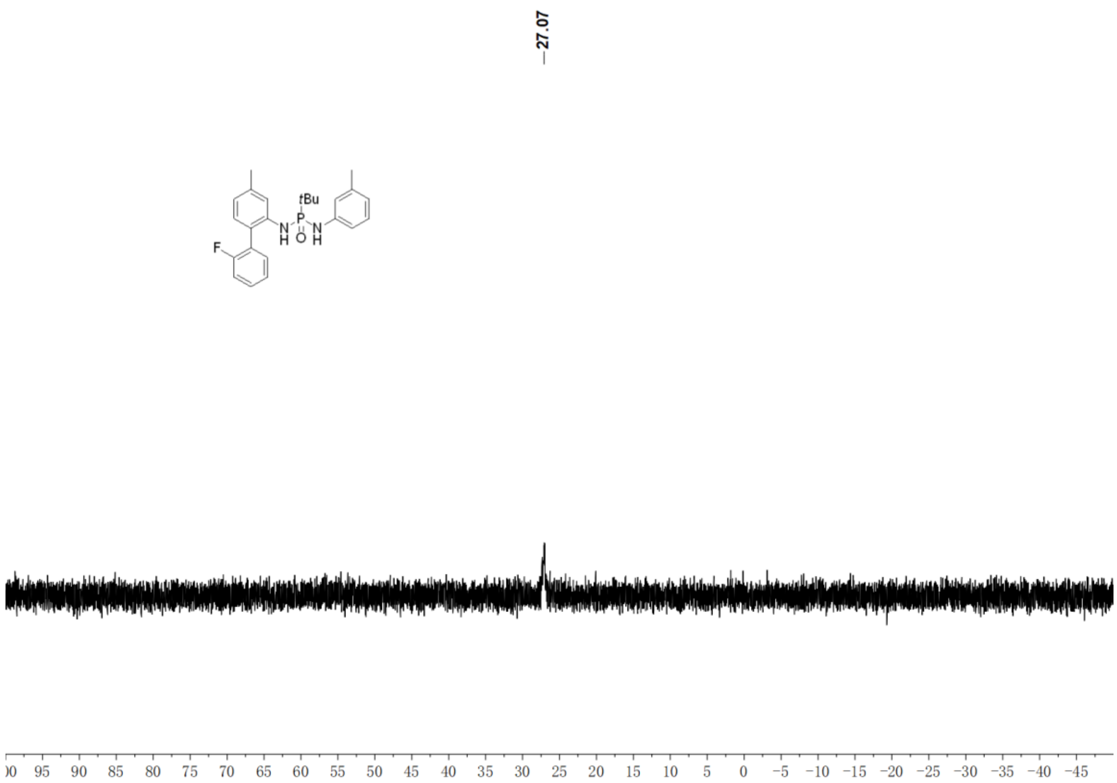

Supplementary Figure 91. ^{31}P NMR spectrum of **b15**

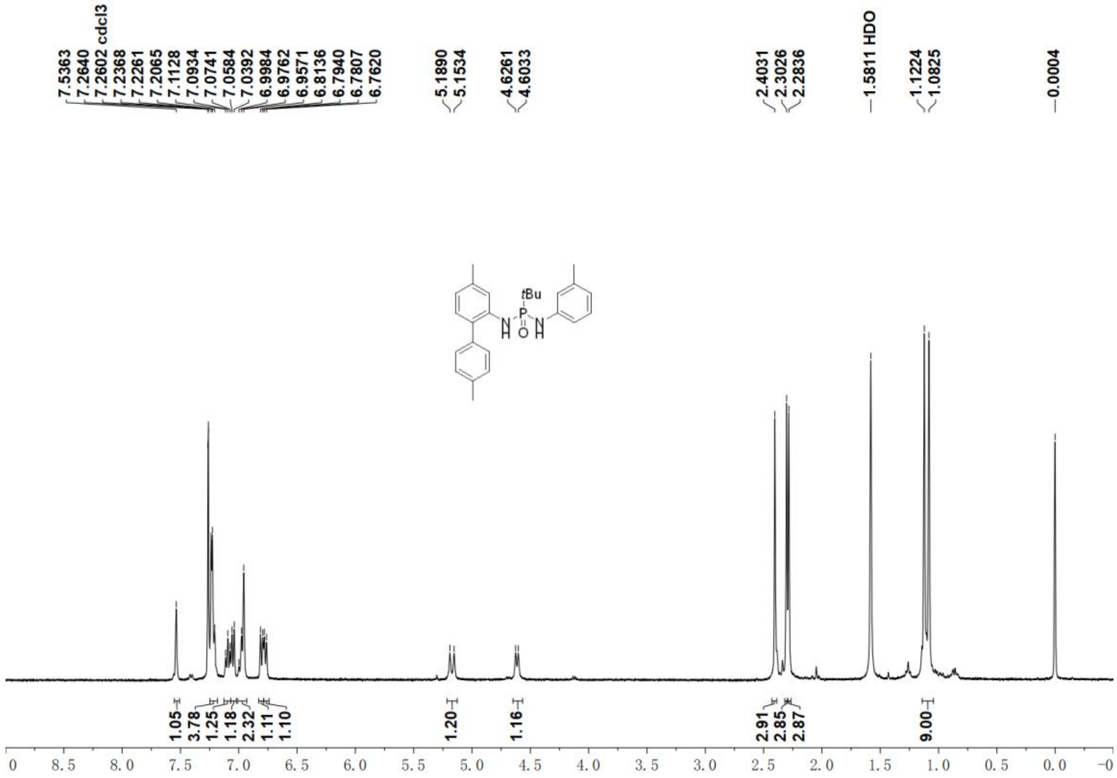

Supplementary Figure 92. ^{1}H NMR spectrum of **b16**

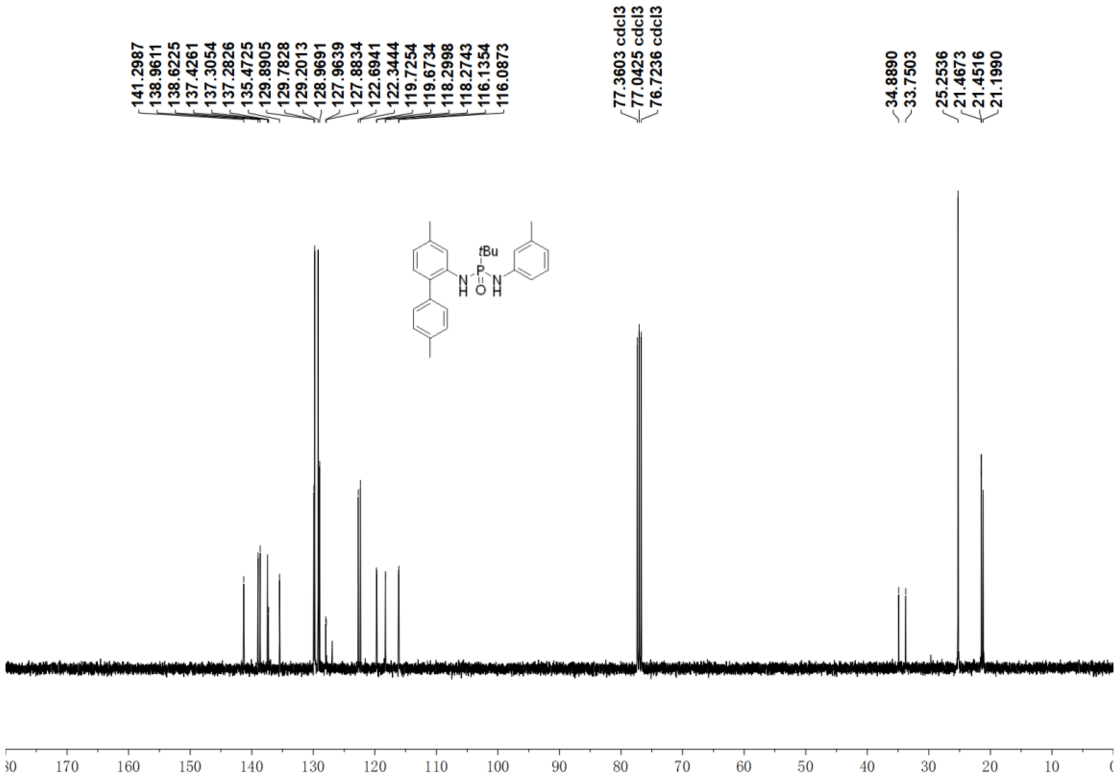

Supplementary Figure 93. ^{13}C NMR spectrum of **b16**

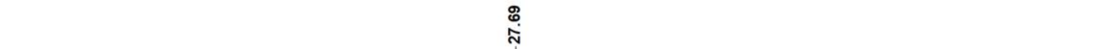

Supplementary Figure 94. ^{31}P NMR spectrum of **b16**

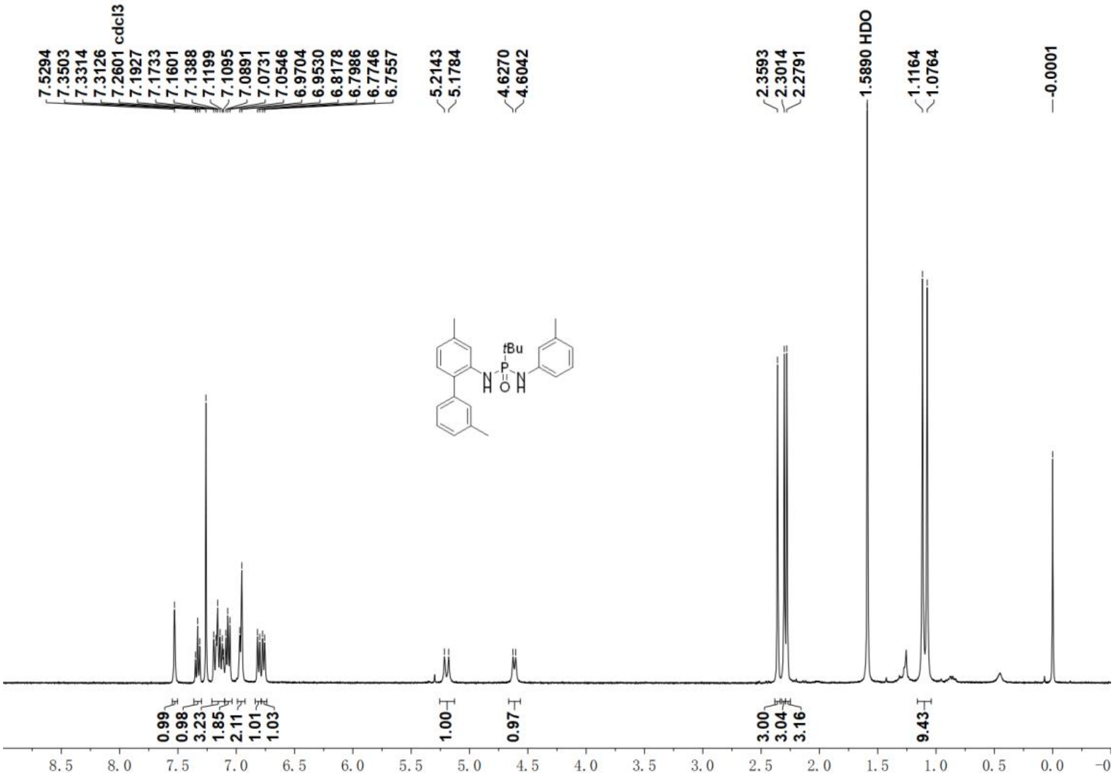

Supplementary Figure 95. ^{1}H NMR spectrum of **b17**

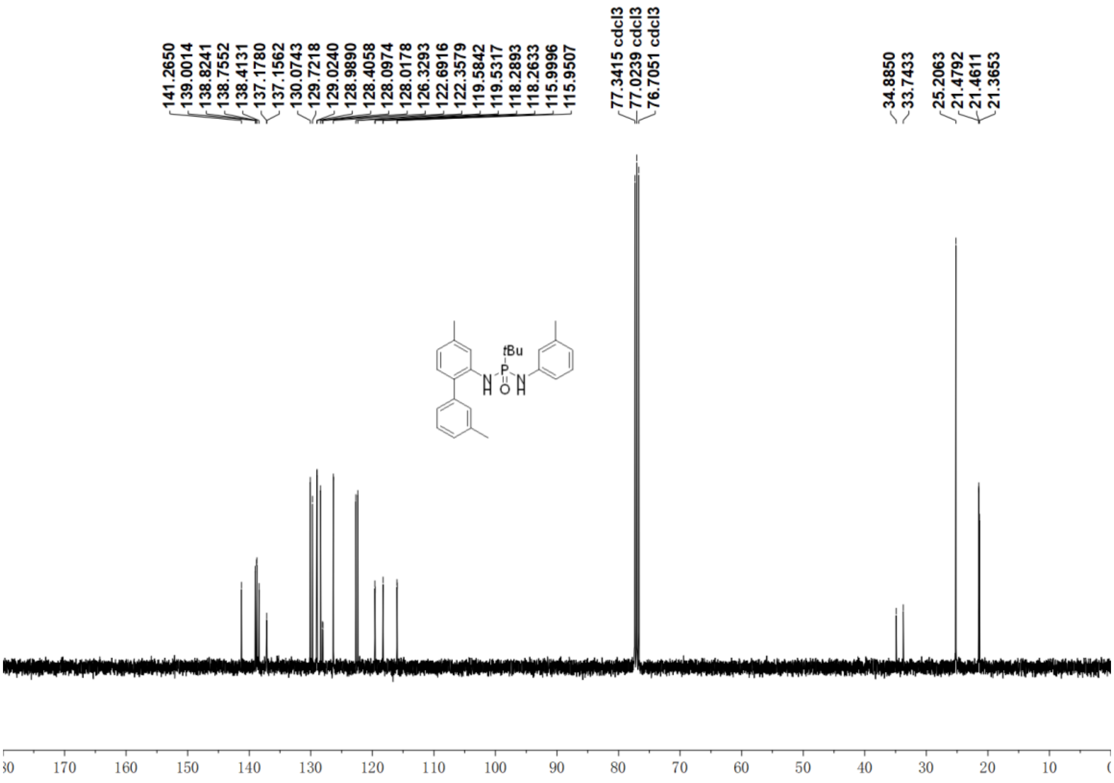

Supplementary Figure 96. ^{13}C NMR spectrum of **b17**

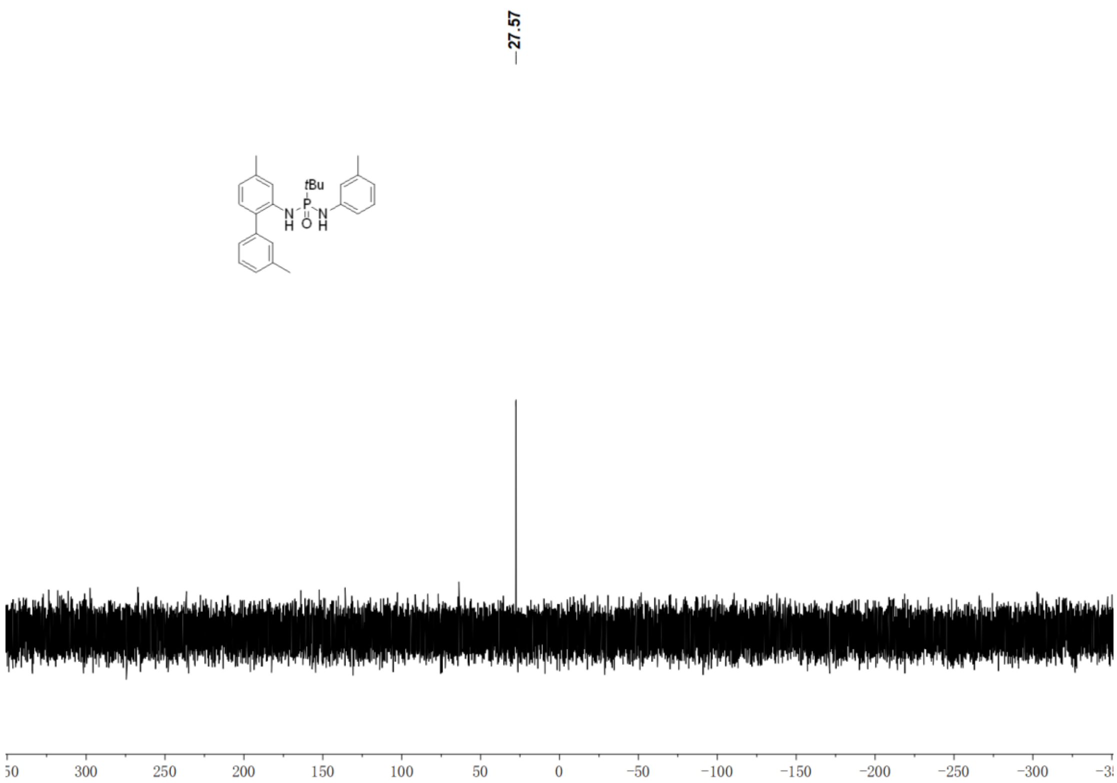

Supplementary Figure 97. ^{31}P NMR spectrum of **b17**

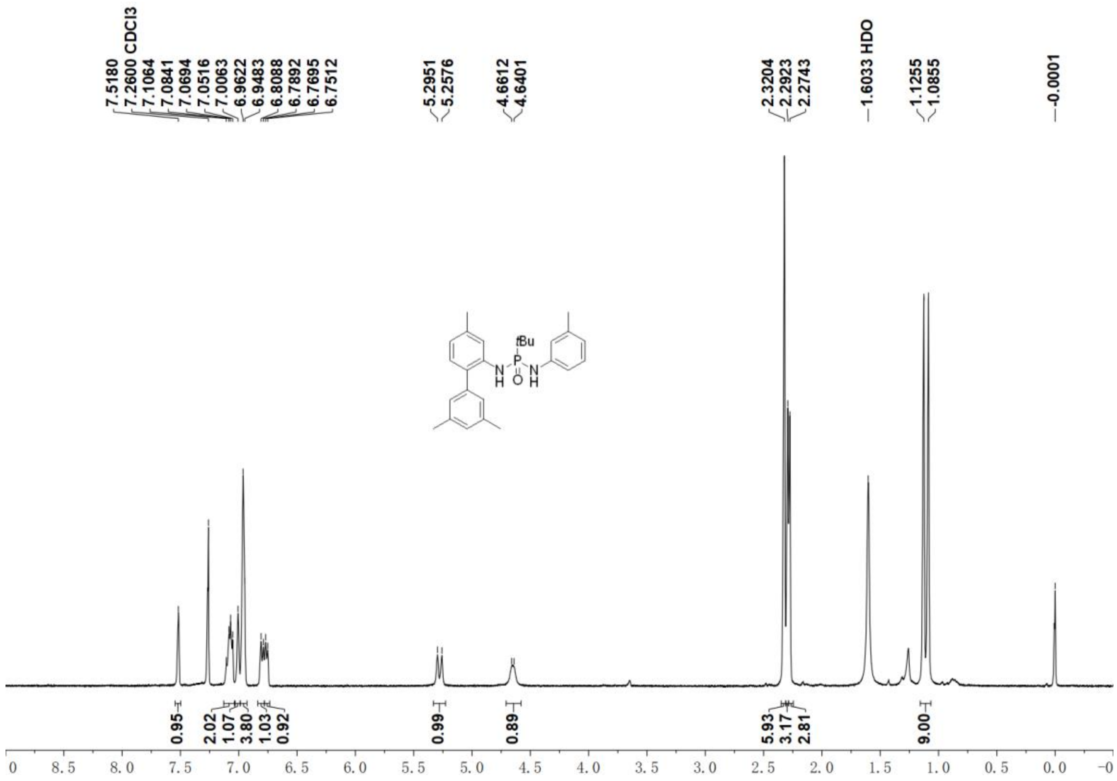

Supplementary Figure 98. ^{1}H NMR spectrum of **b18**

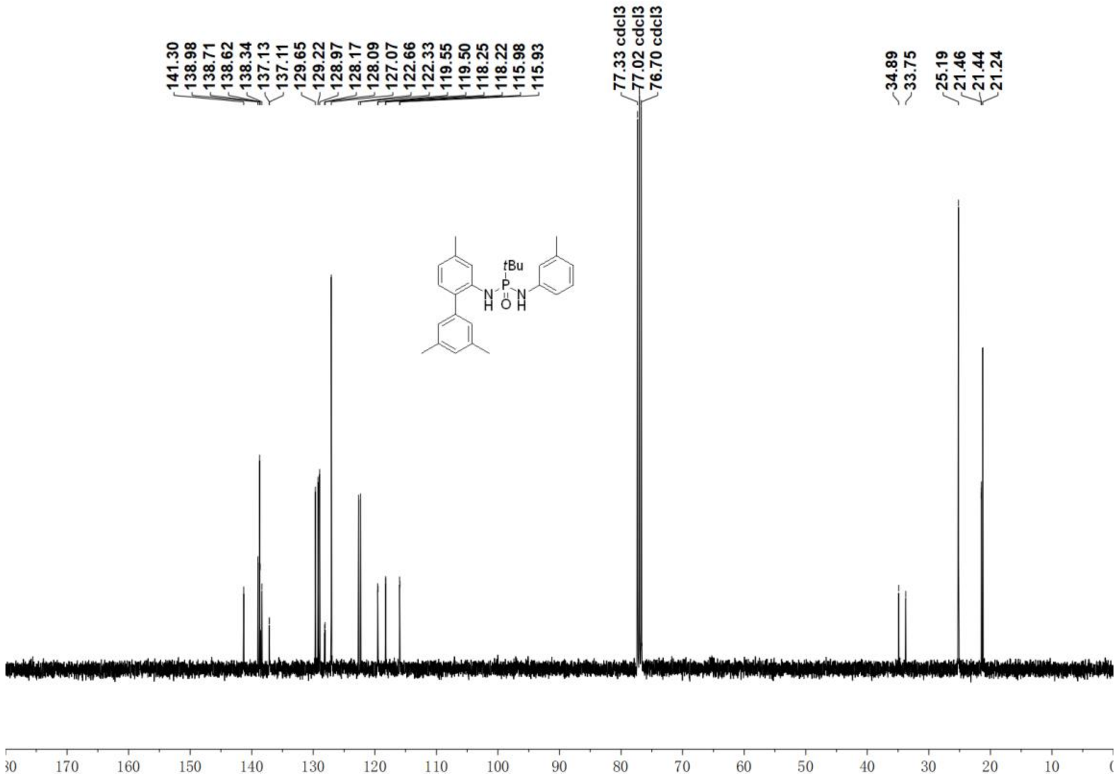

Supplementary Figure 99. ^{13}C NMR spectrum of **b18**

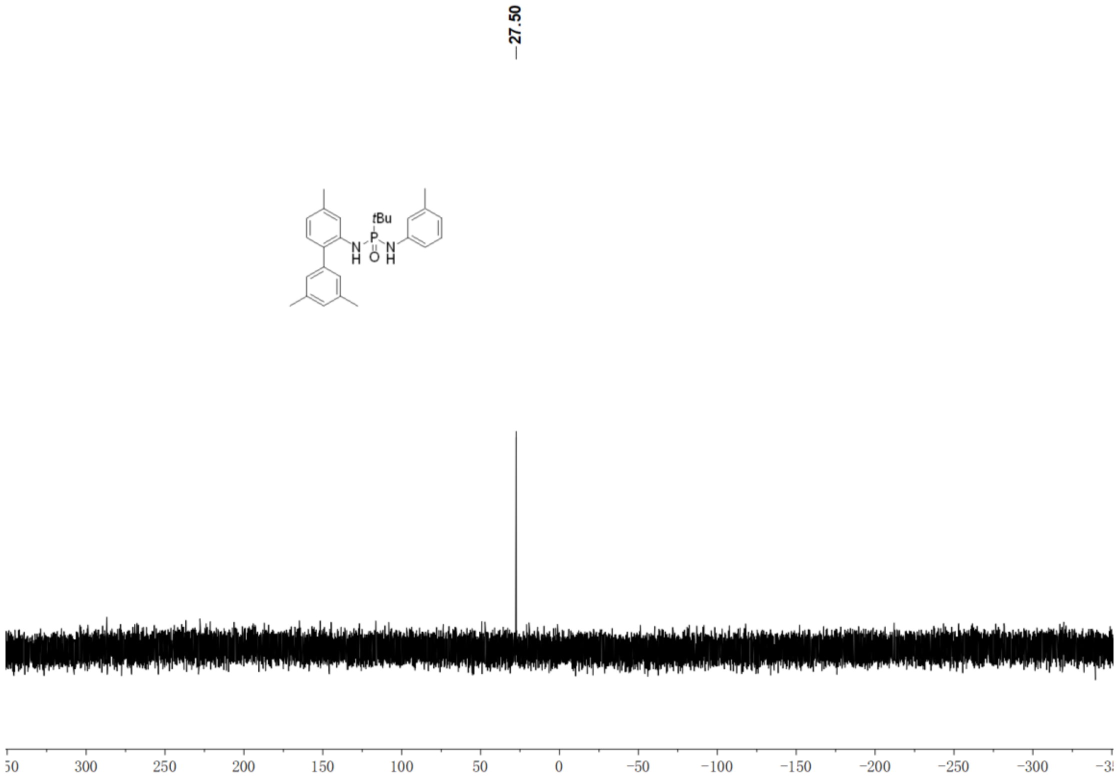

Supplementary Figure 100. ^{31}P NMR spectrum of **b18**

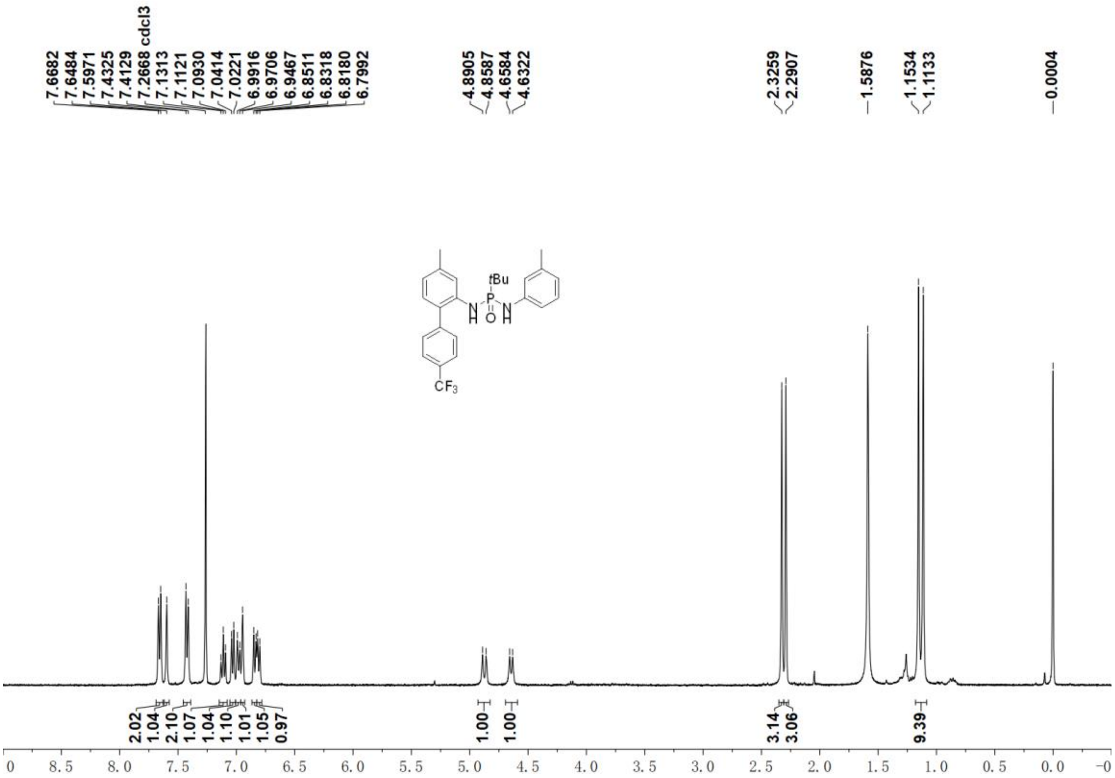

Supplementary Figure 101. ^{1}H NMR spectrum of **b19**

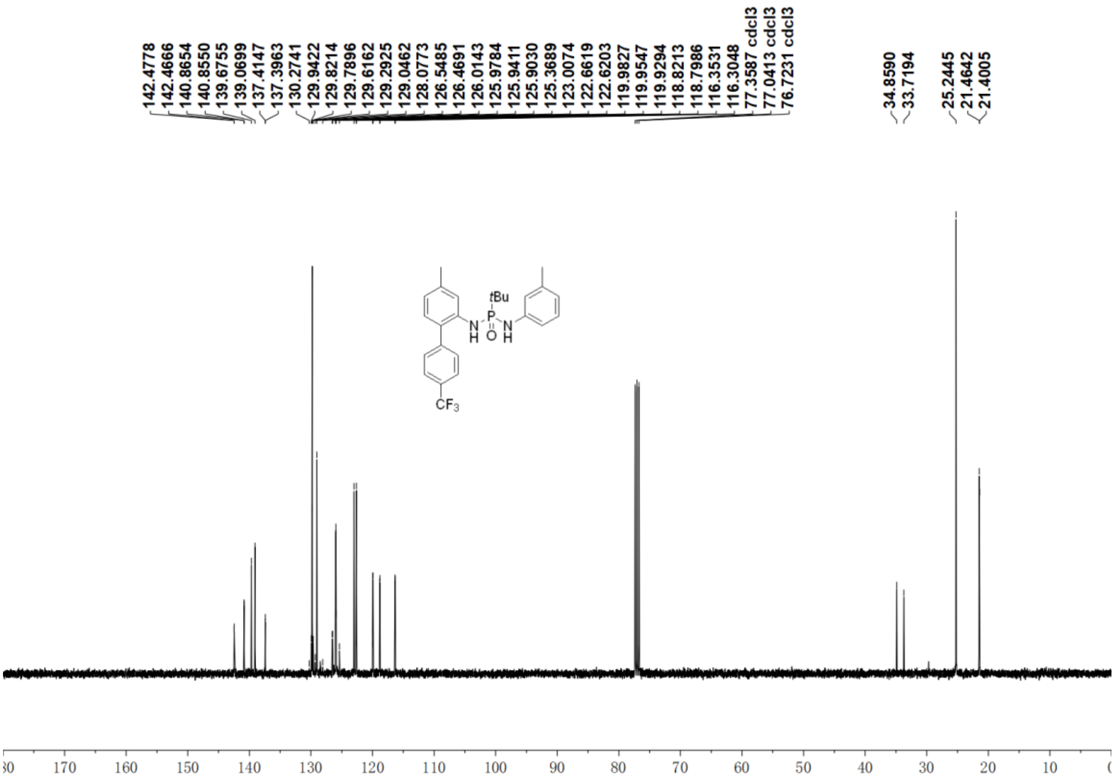

Supplementary Figure 102. ^{13}C NMR spectrum of **b19**

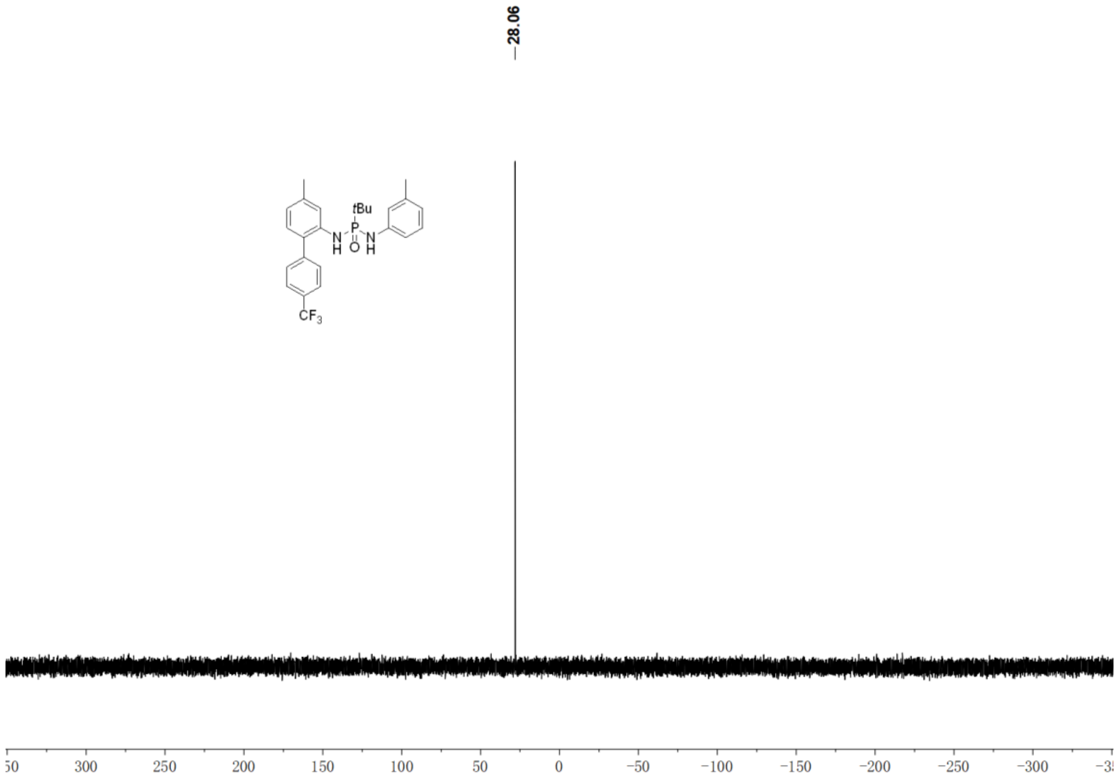

Supplementary Figure 103. ^{31}P NMR spectrum of **b19**

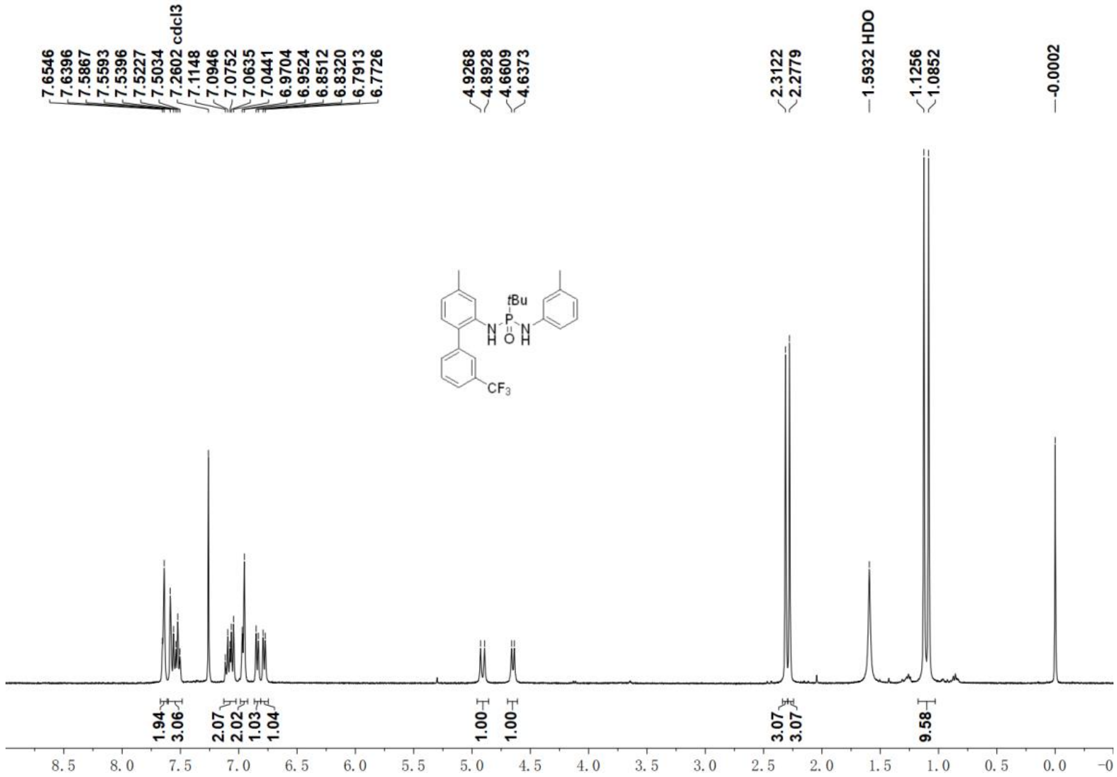

Supplementary Figure 104. ^{1}H NMR spectrum of **b20**

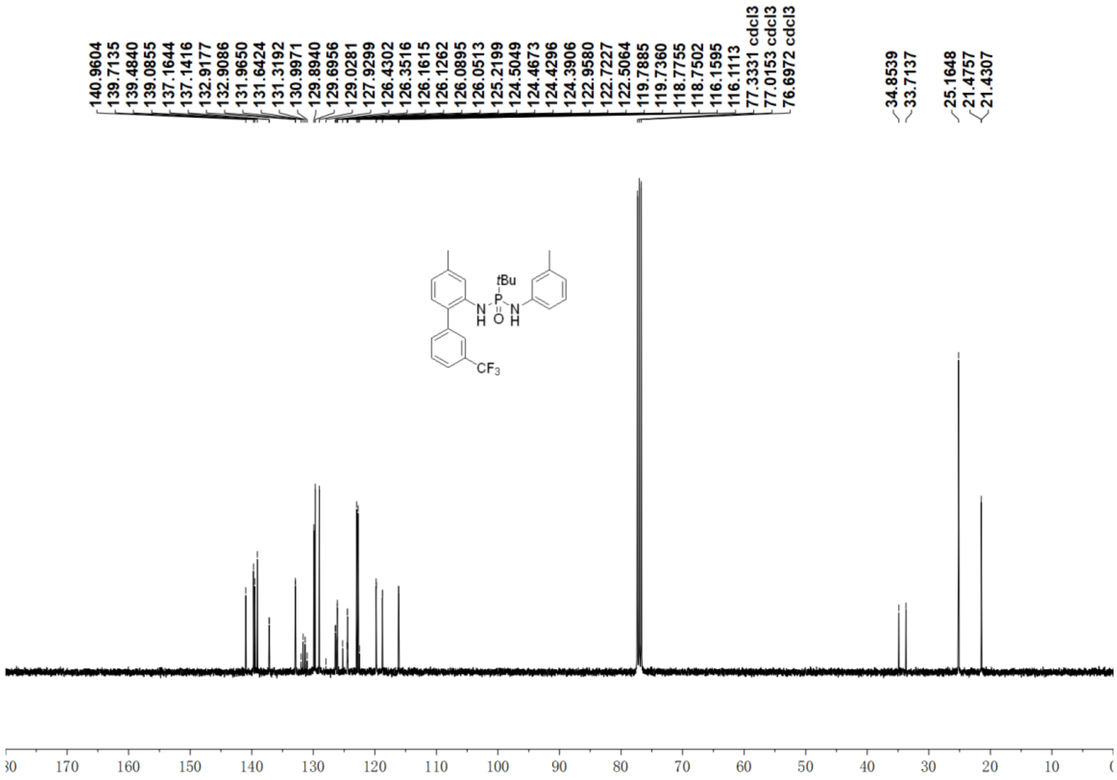

Supplementary Figure 105. ^{13}C NMR spectrum of **b20**

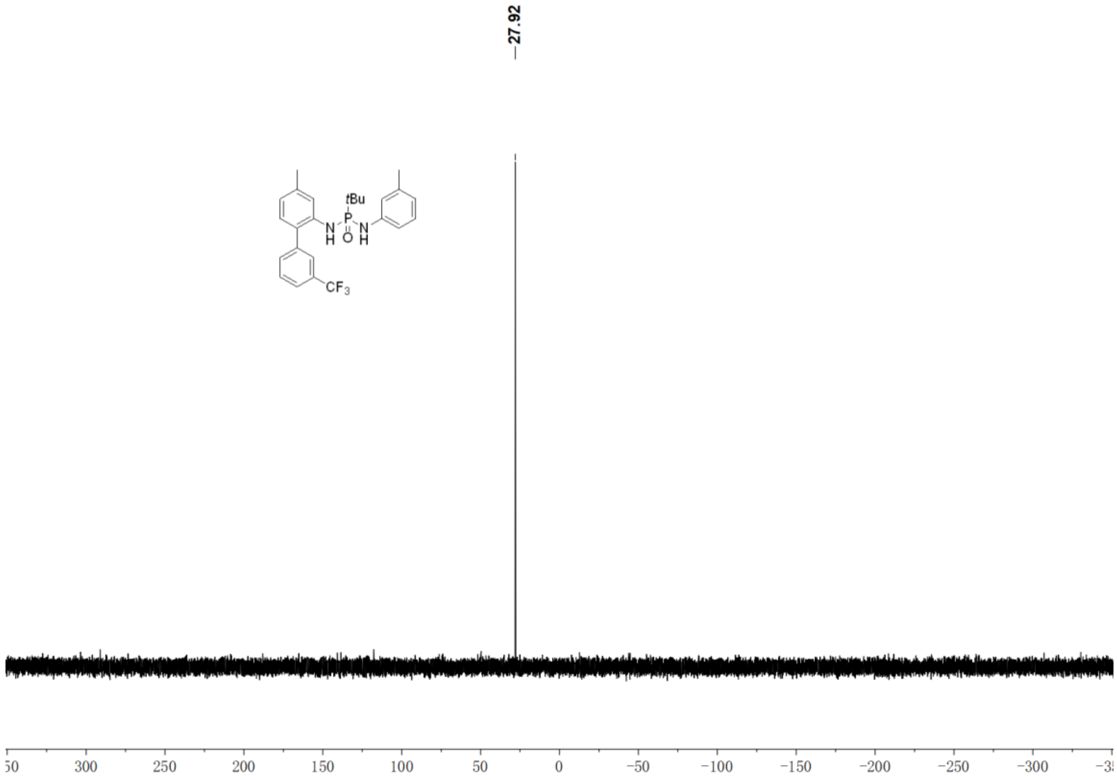

Supplementary Figure 106. ^{31}P NMR spectrum of **b20**

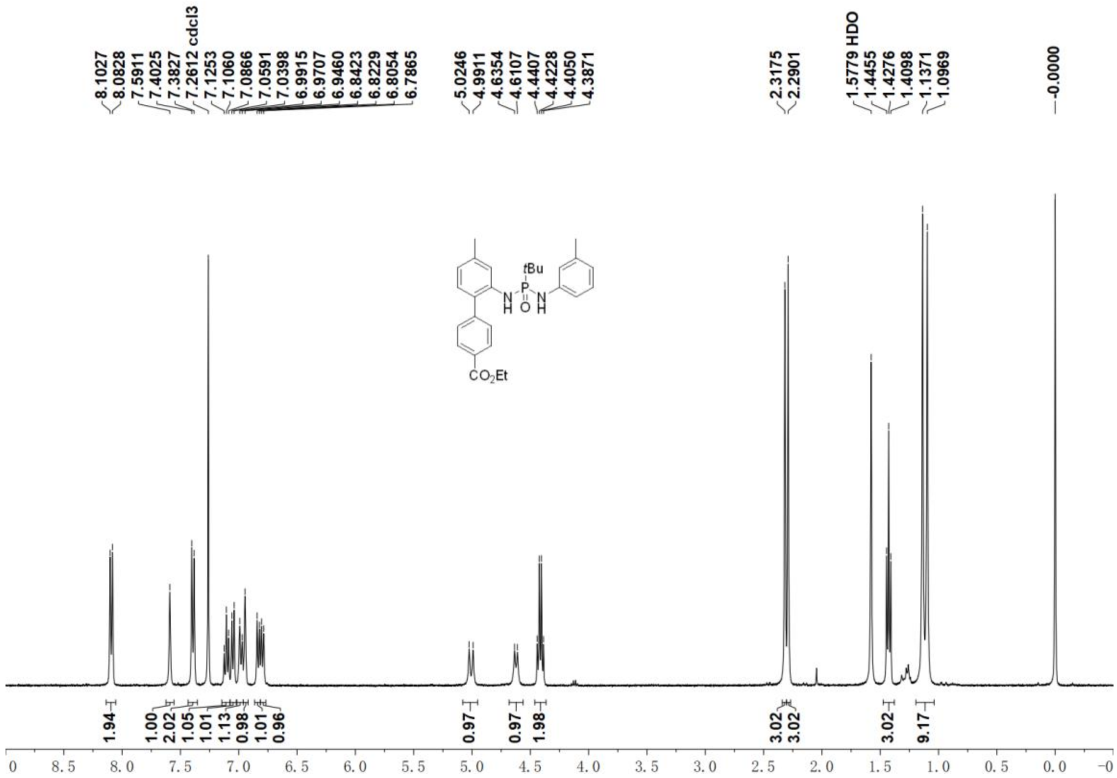

Supplementary Figure 107. ^{1}H NMR spectrum of **b21**

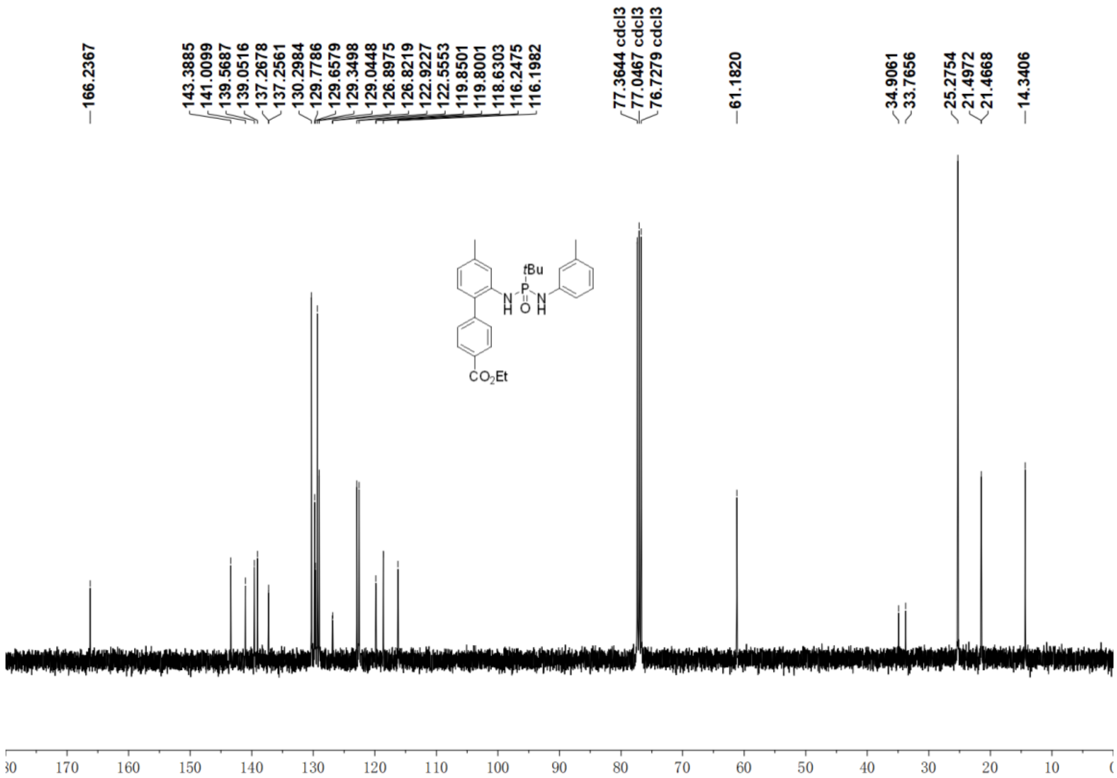

Supplementary Figure 108. ^{13}C NMR spectrum of **b21**

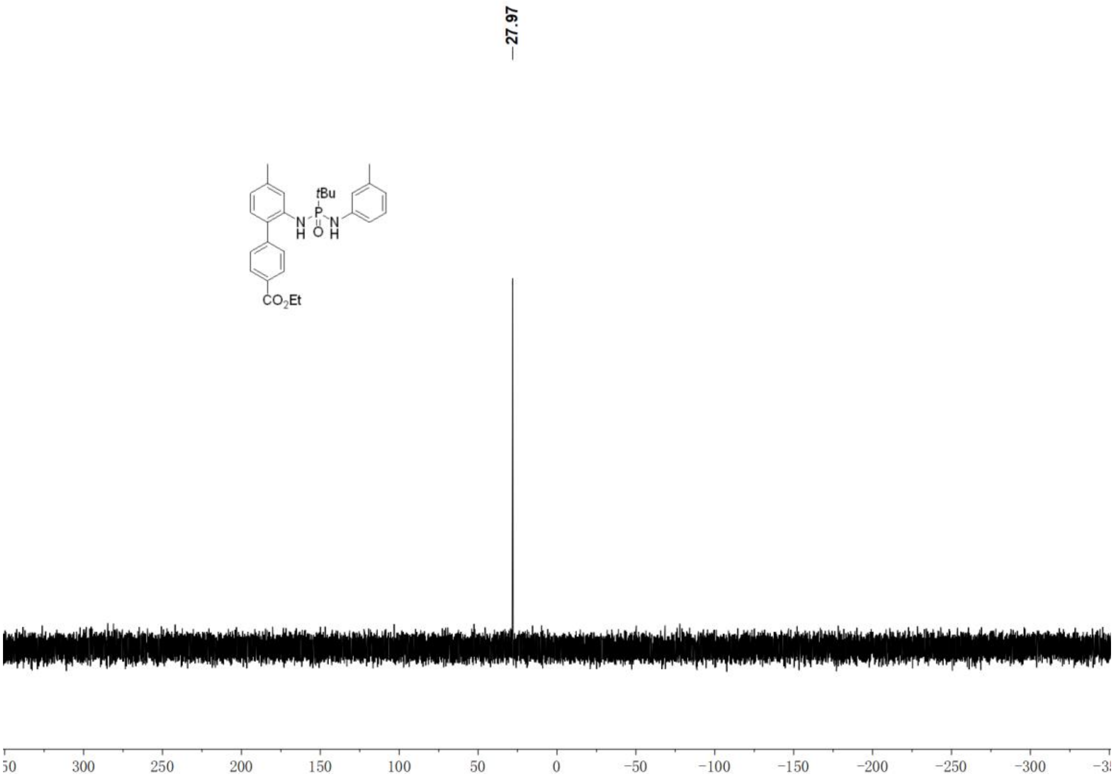

Supplementary Figure 109. ^{31}P NMR spectrum of **b21**

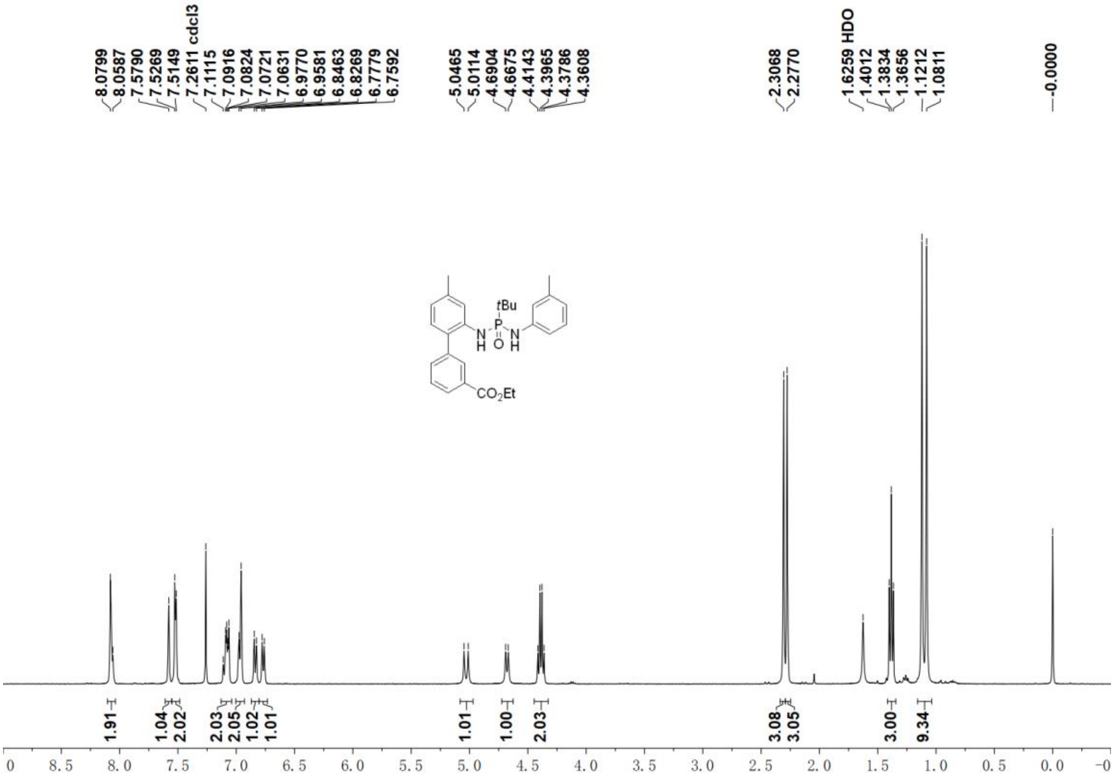

Supplementary Figure 110. ^{1}H NMR spectrum of **b22**

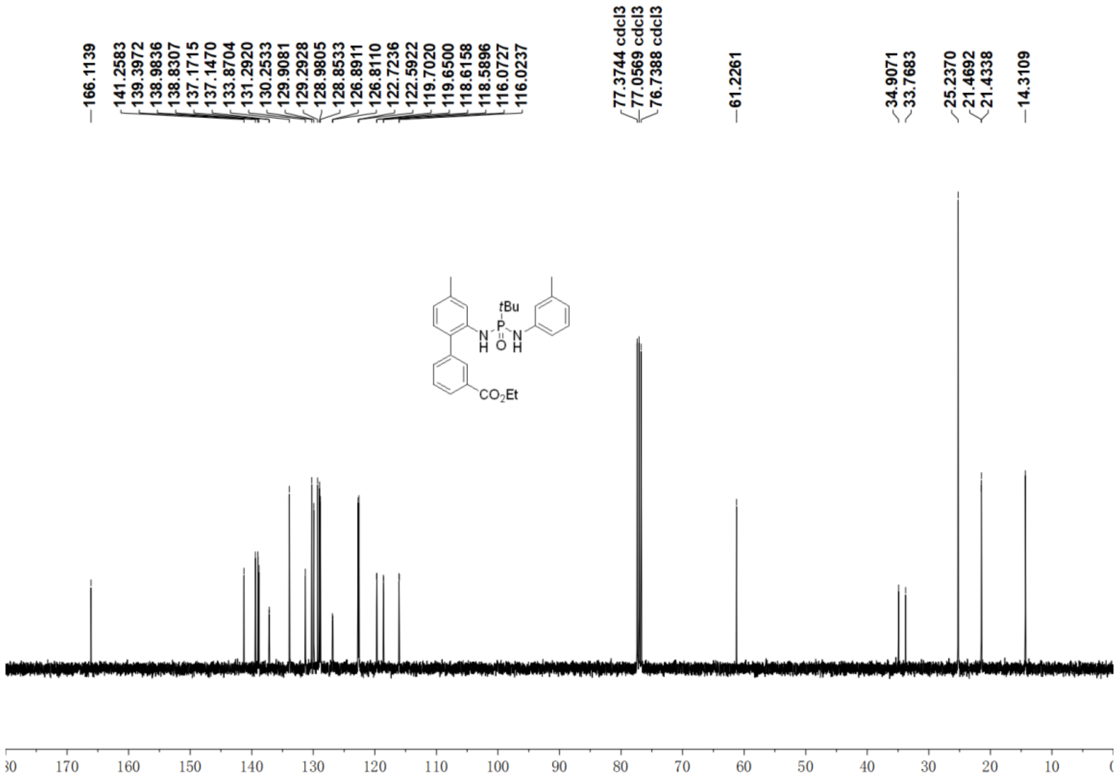

Supplementary Figure 111. ^{13}C NMR spectrum of **b22**

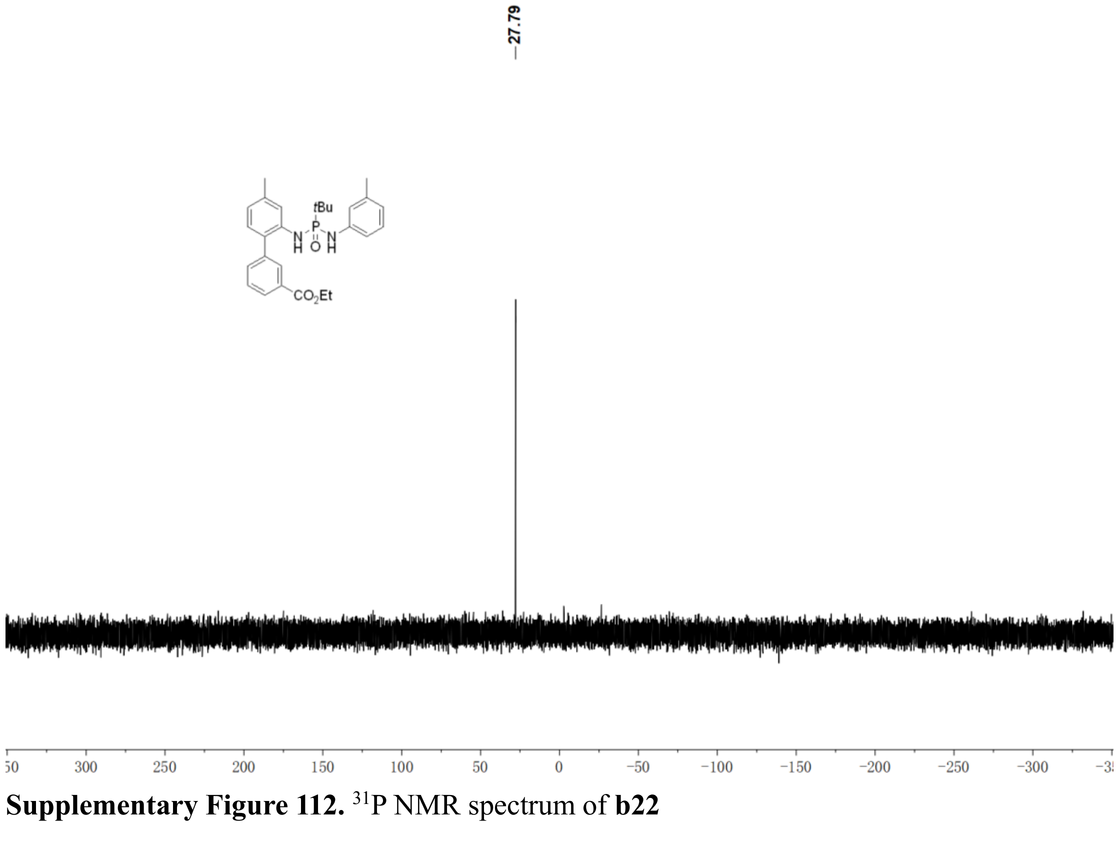

Supplementary Figure 112. ^{31}P NMR spectrum of **b22**

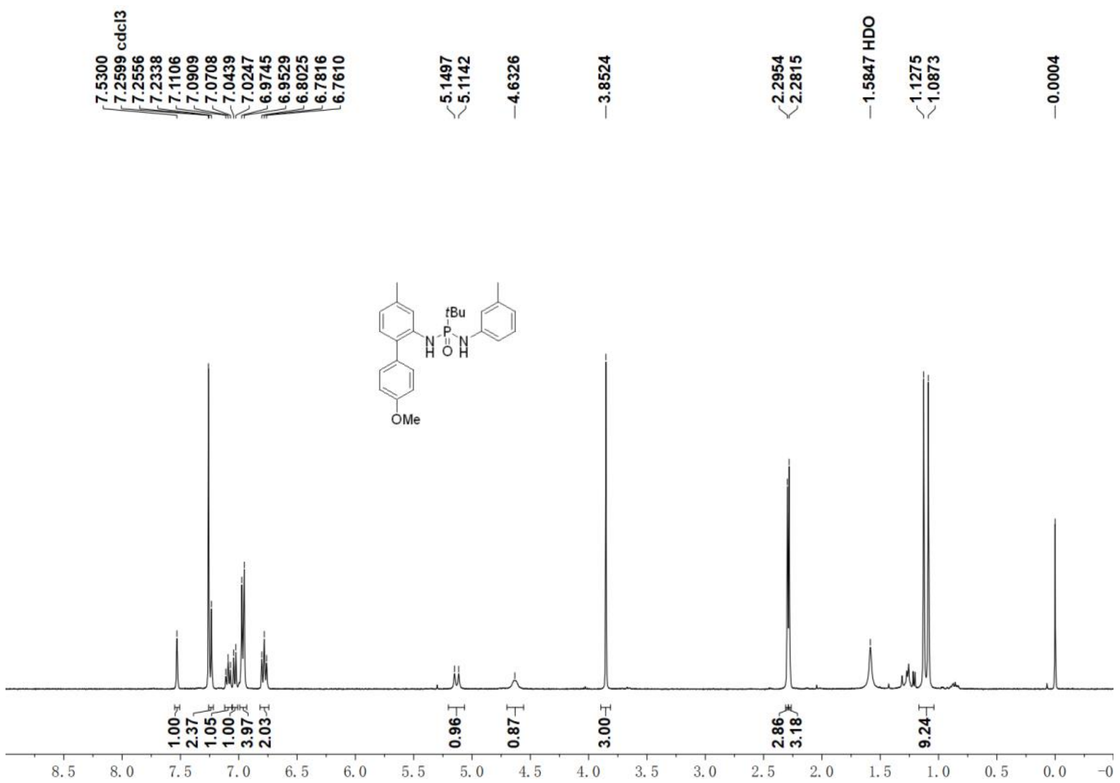

Supplementary Figure 113. ^{1}H NMR spectrum of **b23**

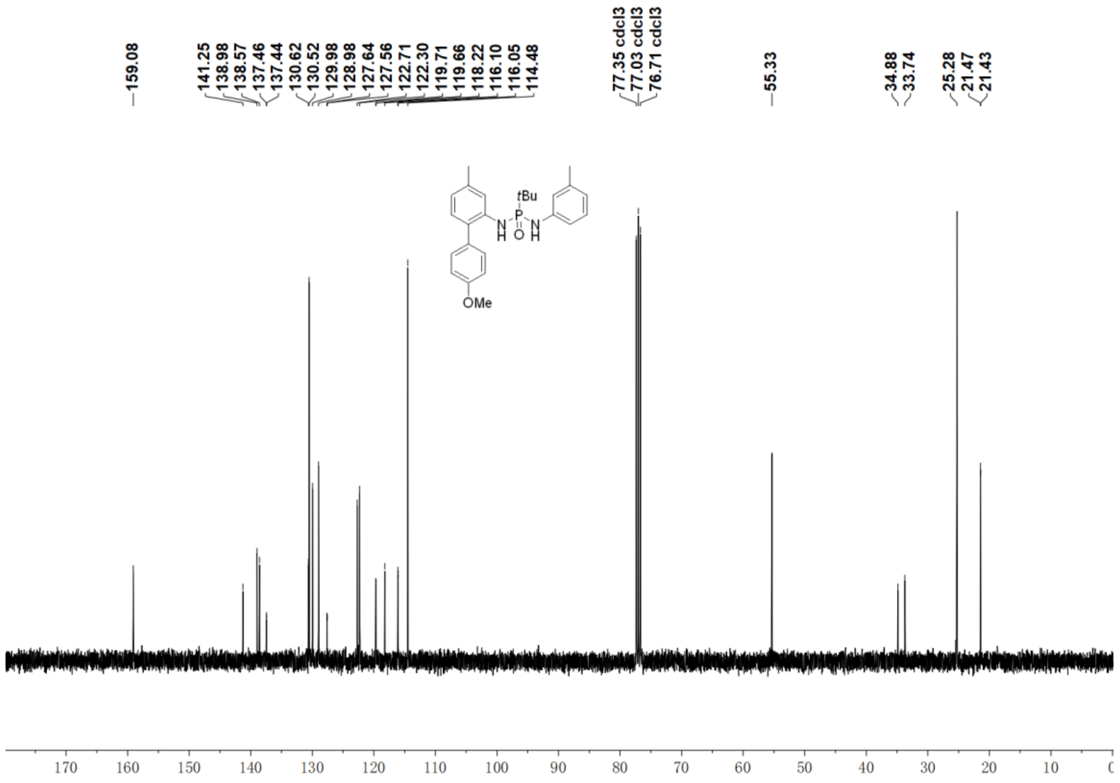

Supplementary Figure 114. ^{13}C NMR spectrum of **b23**

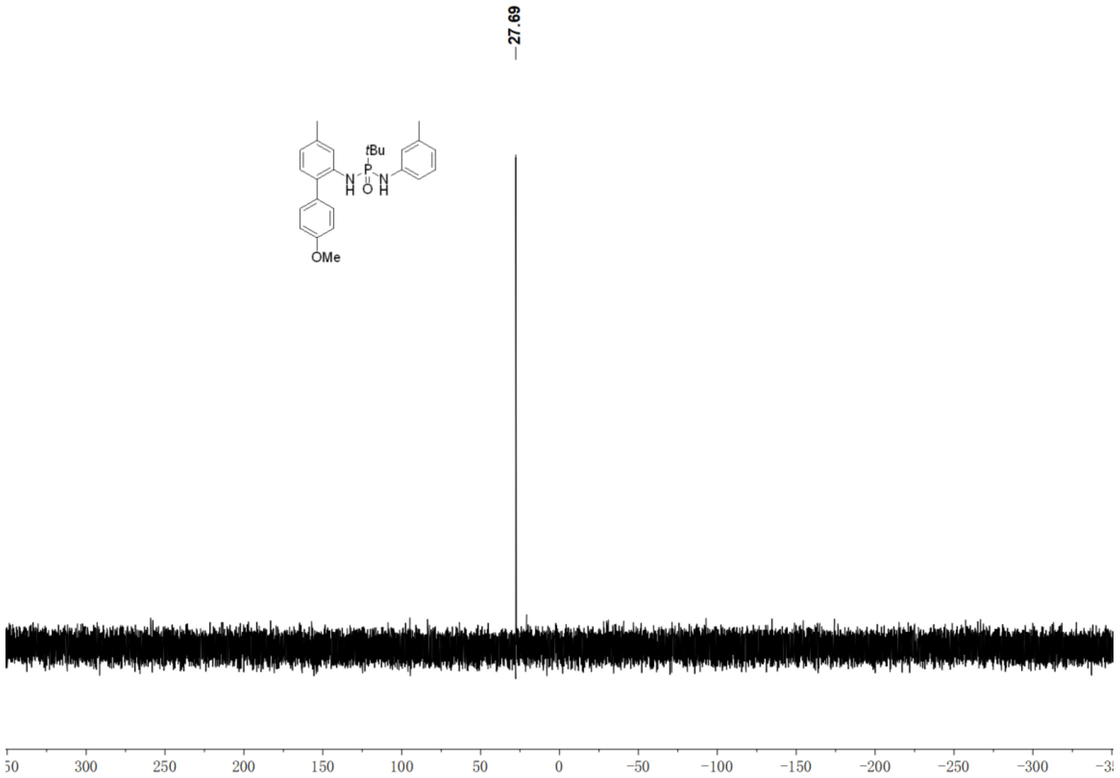

Supplementary Figure 115. ^{31}P NMR spectrum of **b23**

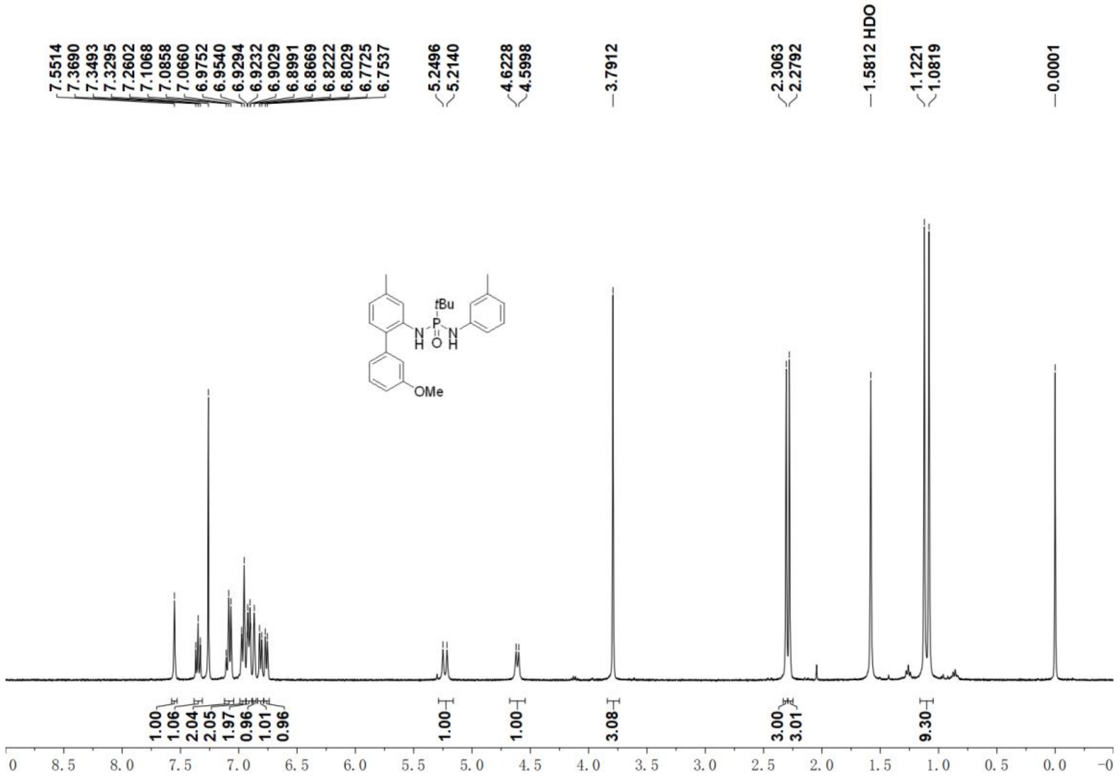

Supplementary Figure 116. ^{1}H NMR spectrum of **b24**

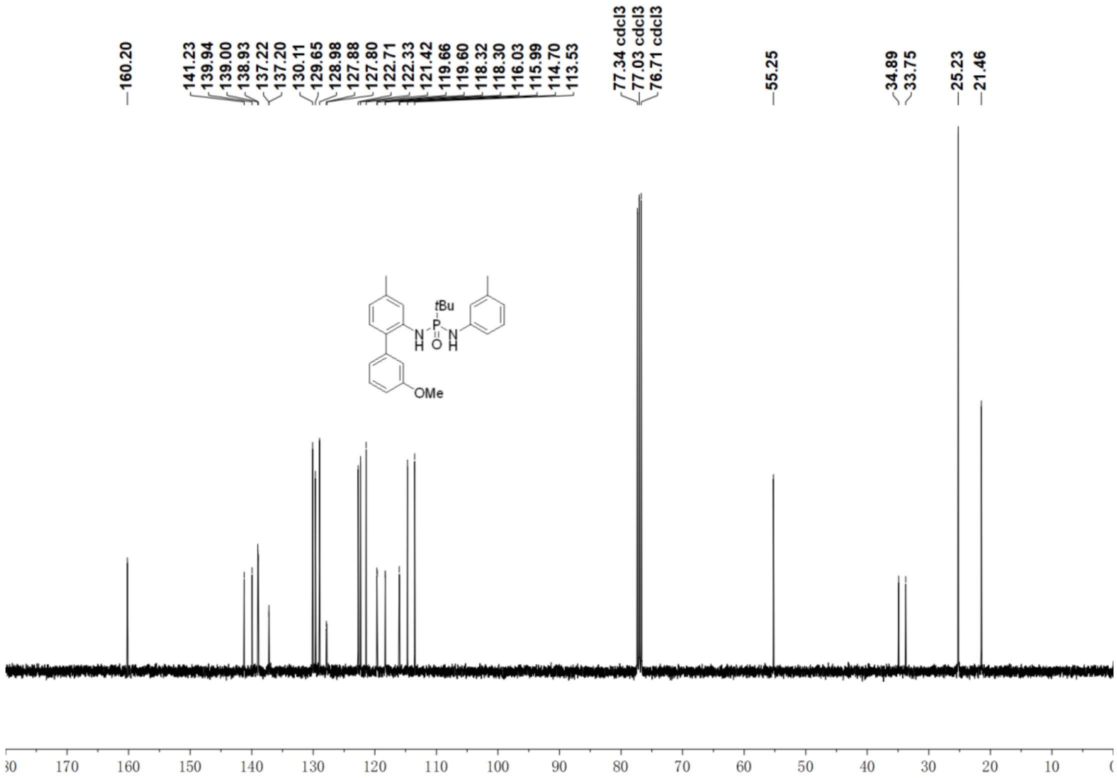

Supplementary Figure 117. ^{13}C NMR spectrum of **b24**

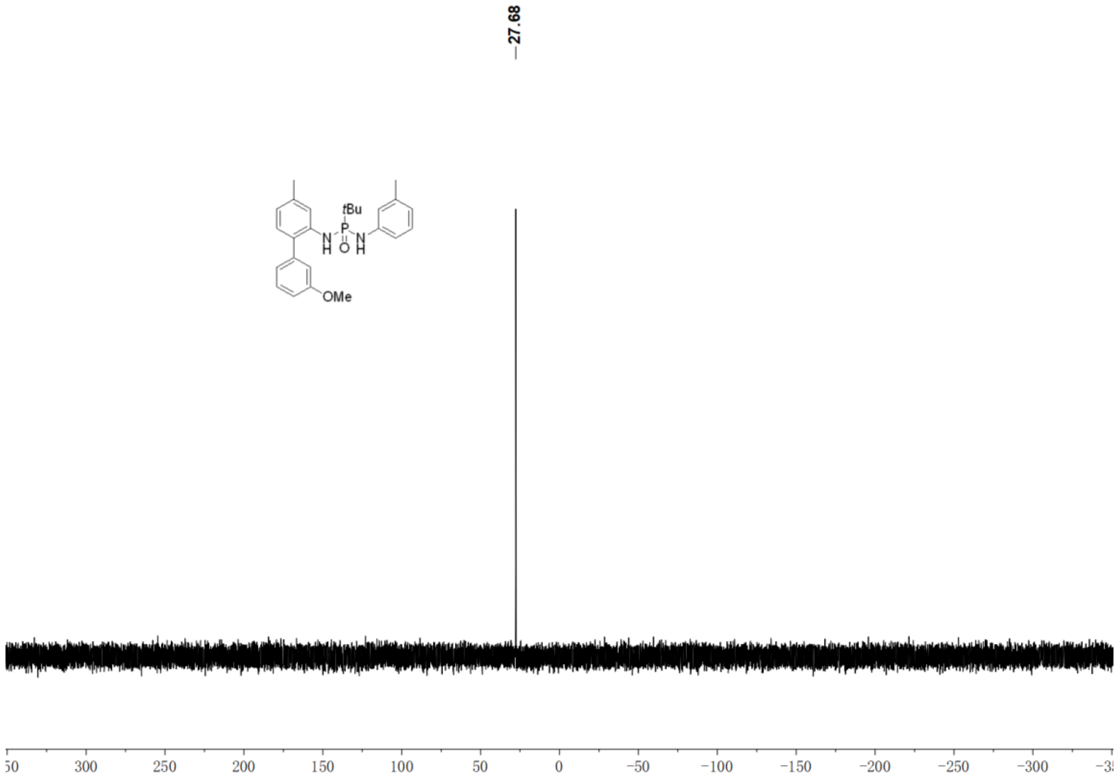

Supplementary Figure 118. ^{31}P NMR spectrum of **b24**

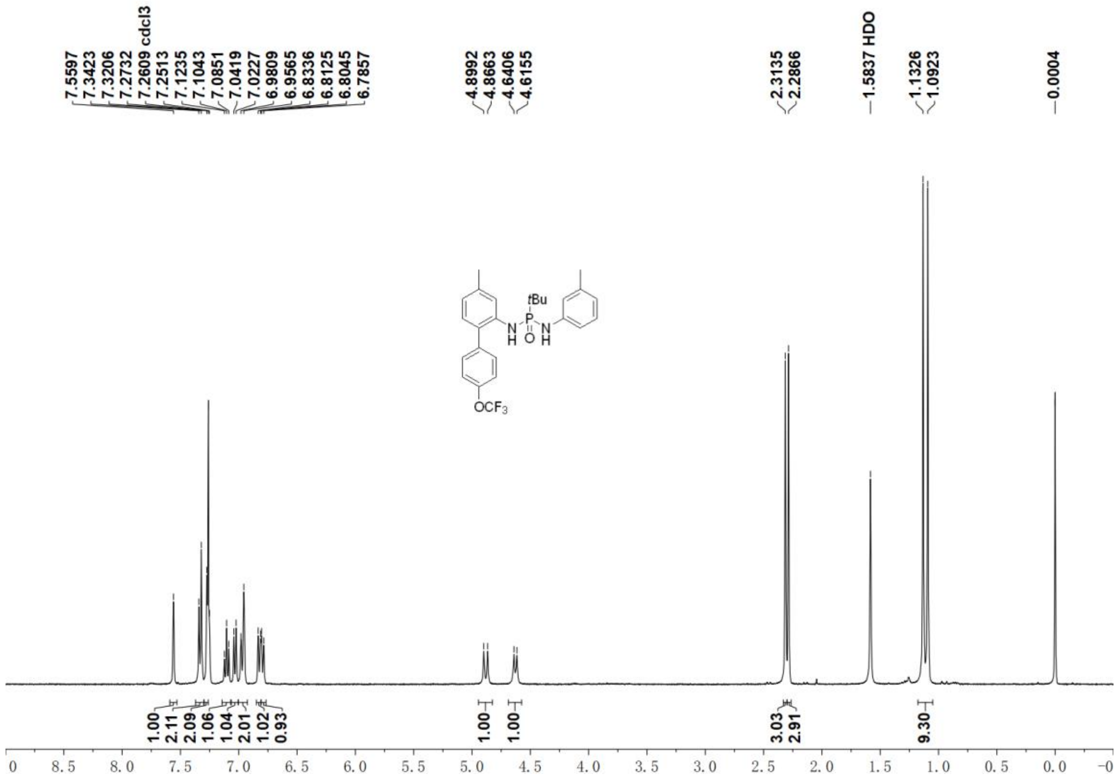

Supplementary Figure 119. ^{1}H NMR spectrum of **b25**

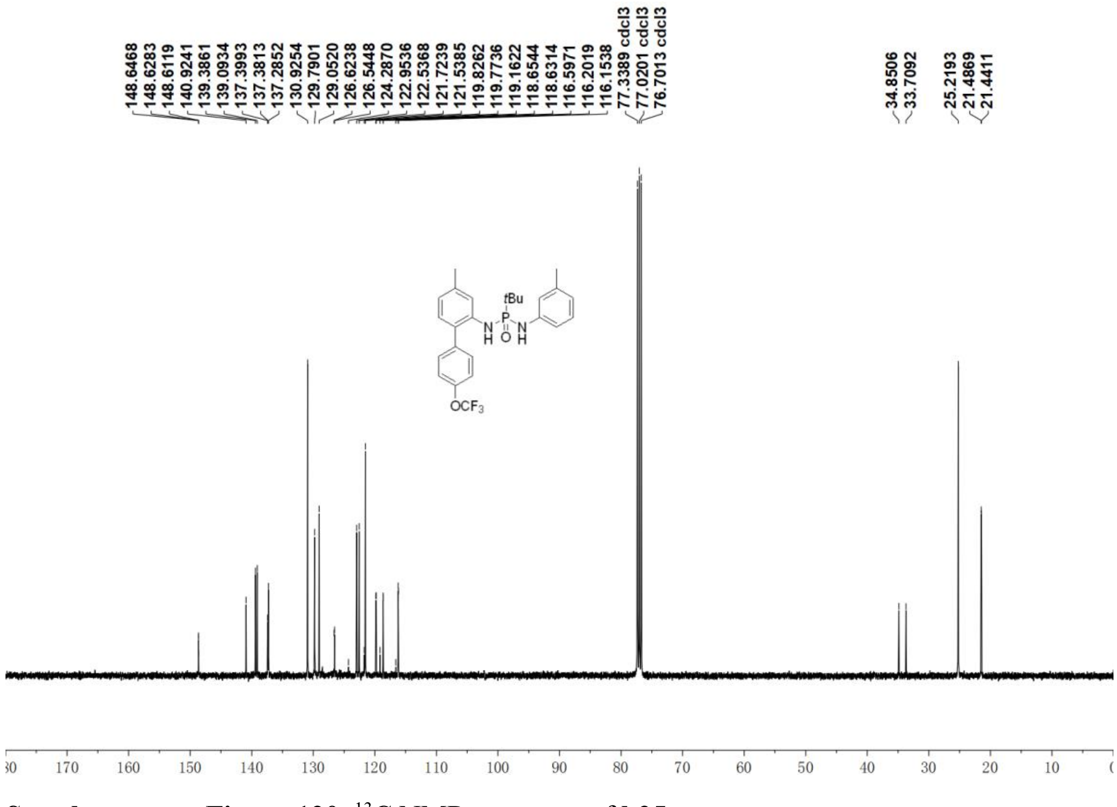

Supplementary Figure 120. ^{13}C NMR spectrum of **b25**

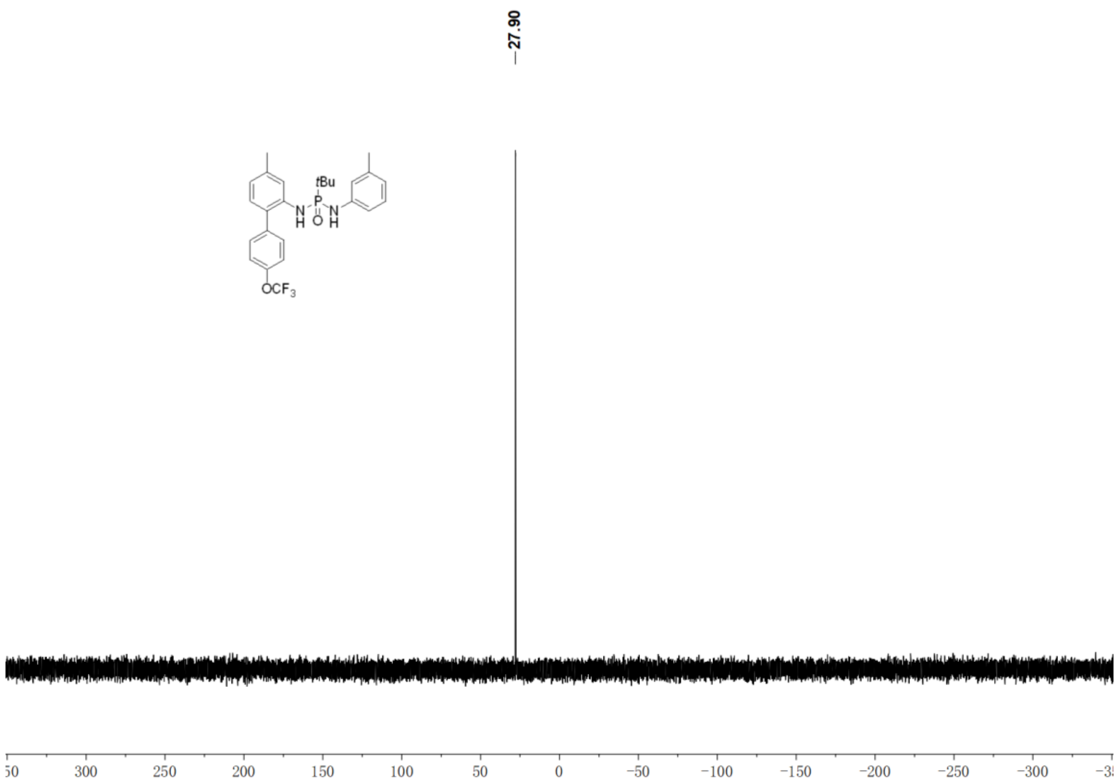

Supplementary Figure 121. ^{31}P NMR spectrum of **b25**

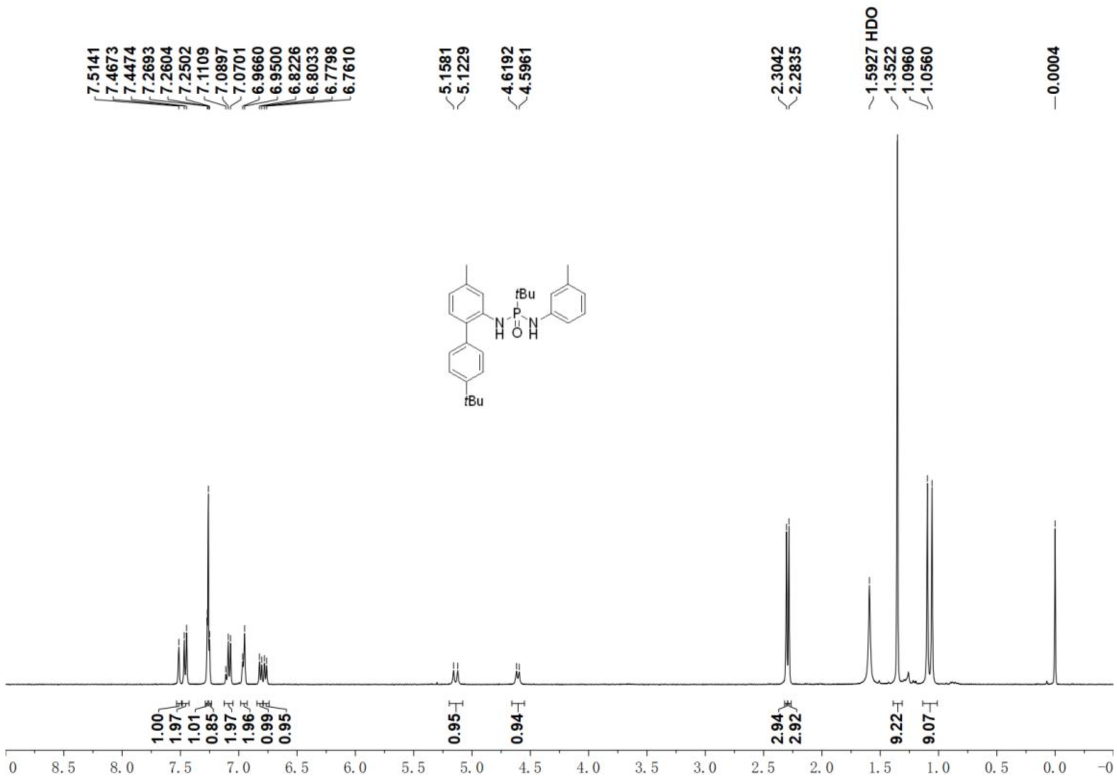

Supplementary Figure 122. ^{1}H NMR spectrum of **b26**

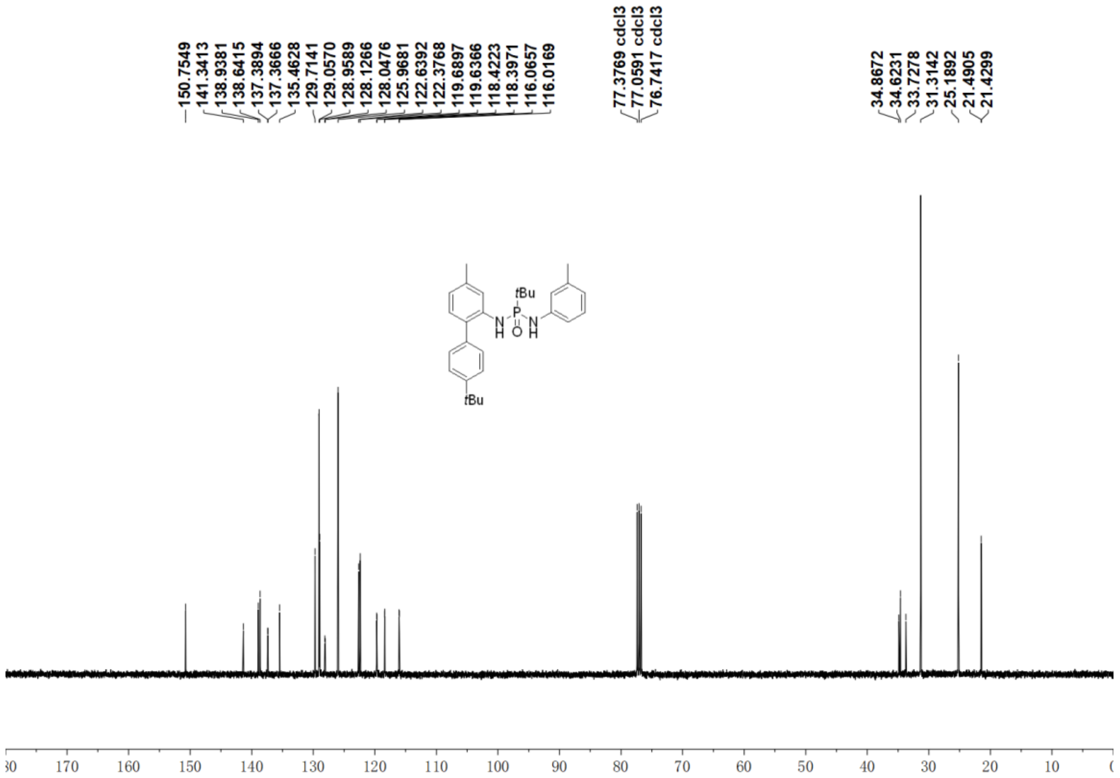

Supplementary Figure 123. ^{13}C NMR spectrum of **b26**

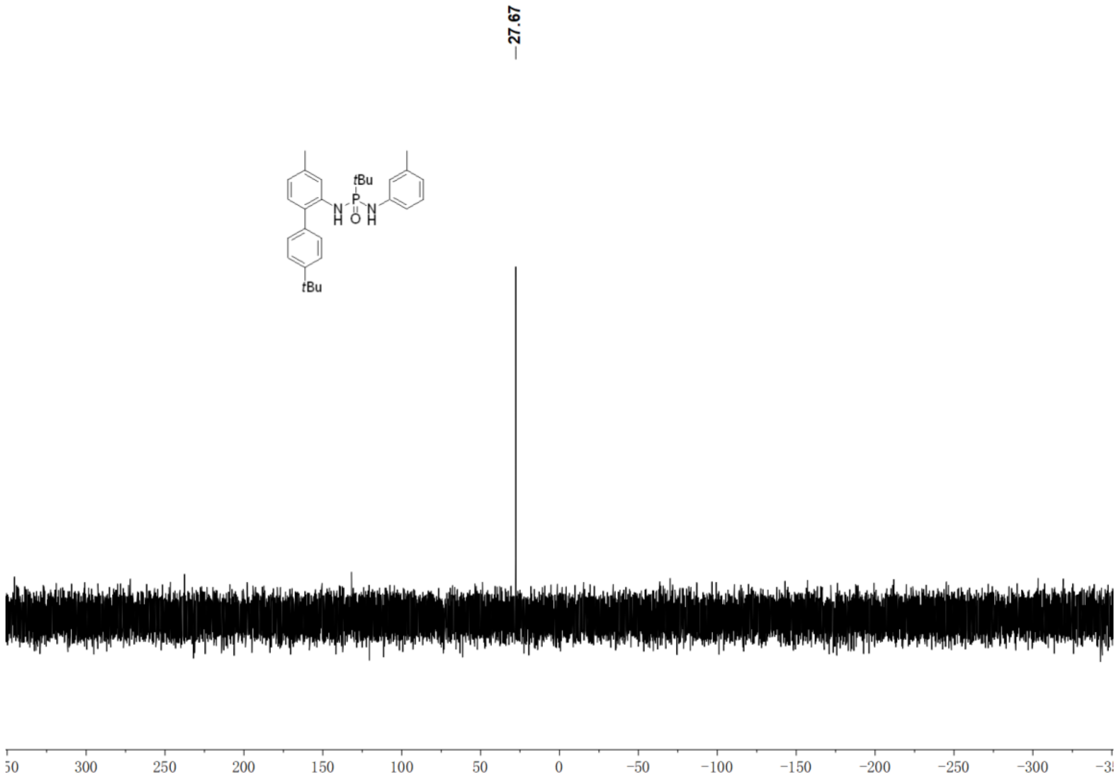

Supplementary Figure 124. ^{31}P NMR spectrum of **b26**

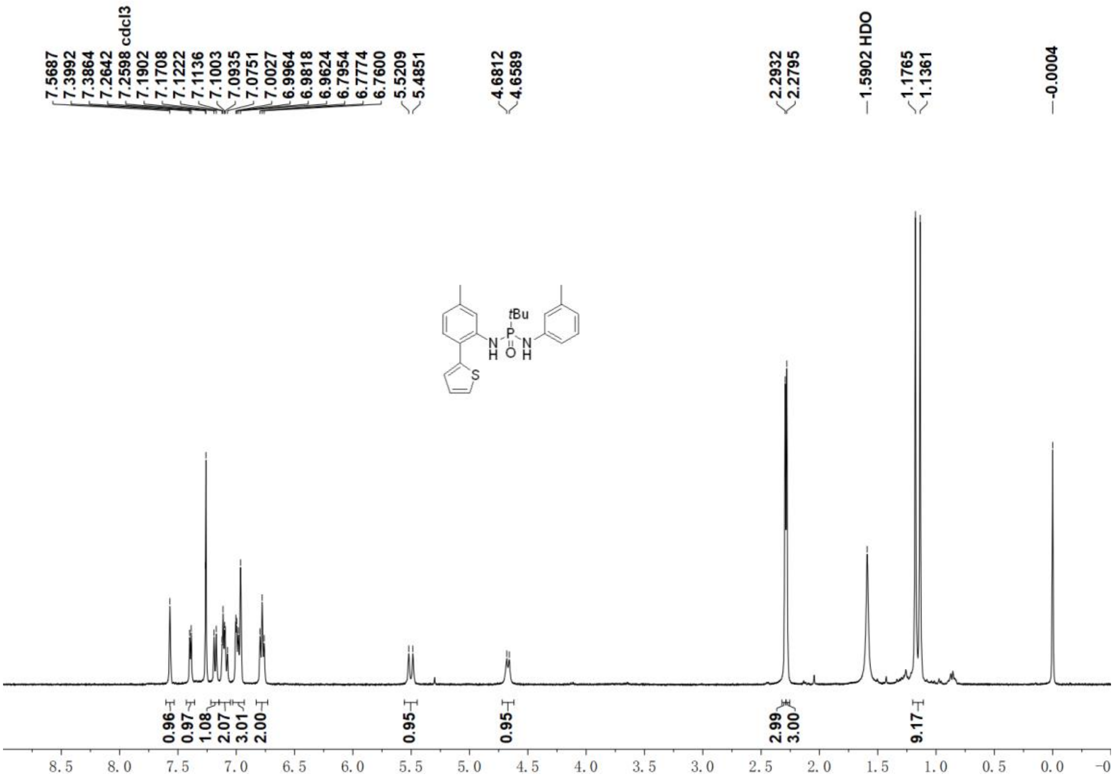

Supplementary Figure 125. ^{1}H NMR spectrum of **b27**

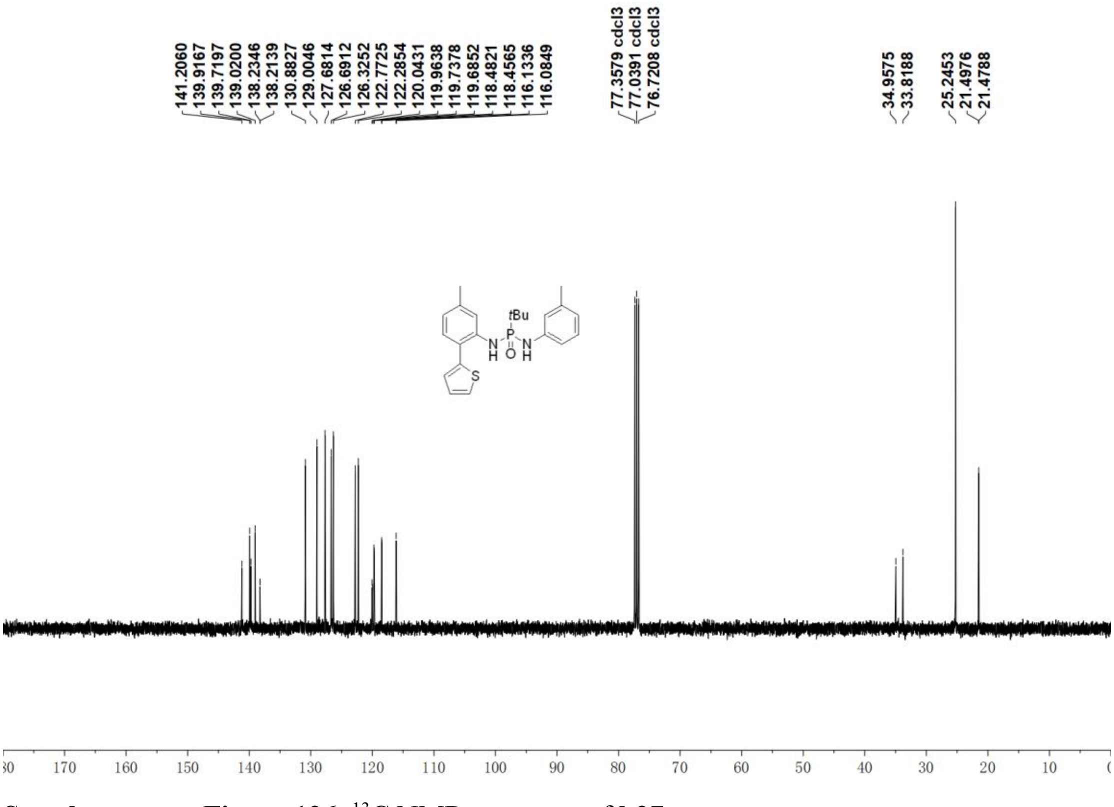

Supplementary Figure 126. ^{13}C NMR spectrum of **b27**

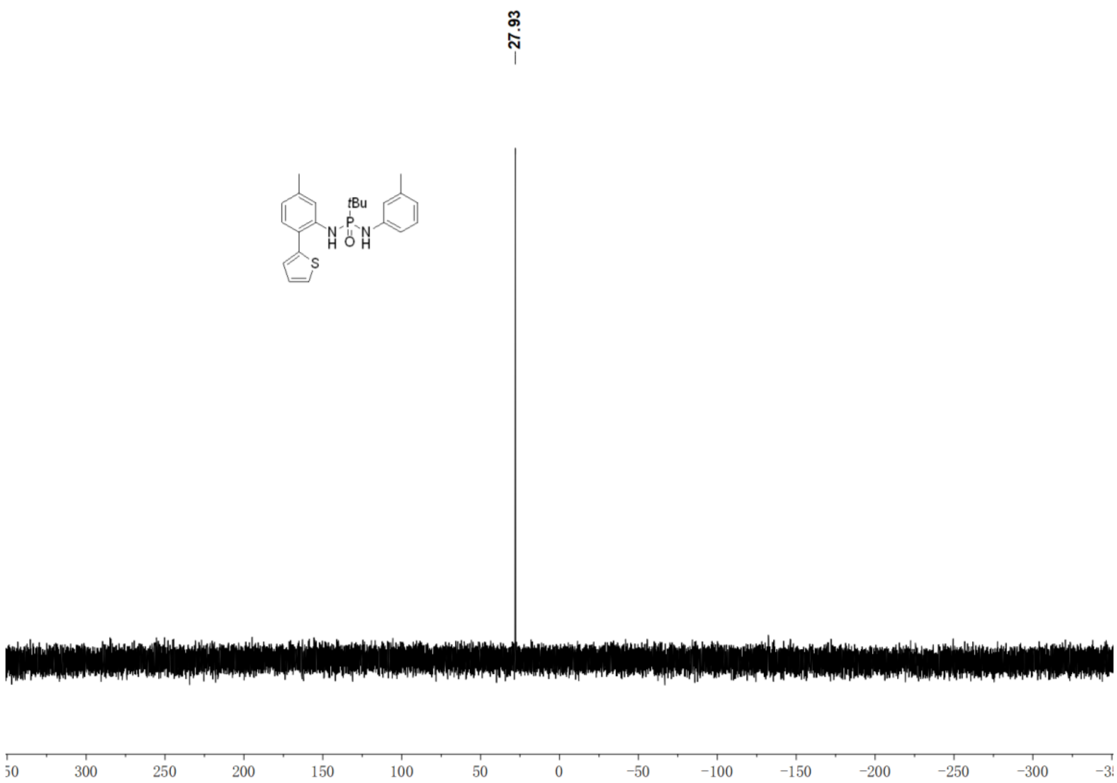

Supplementary Figure 127. ^{31}P NMR spectrum of **b27**

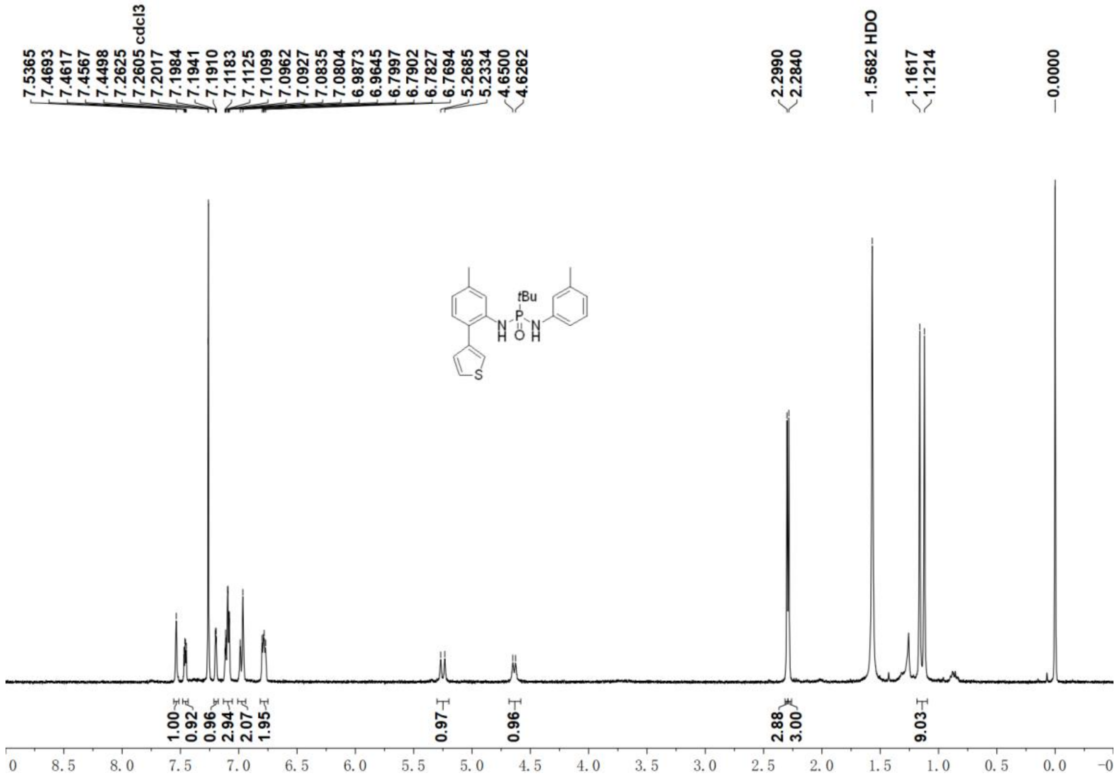

Supplementary Figure 128. ^{1}H NMR spectrum of **b28**

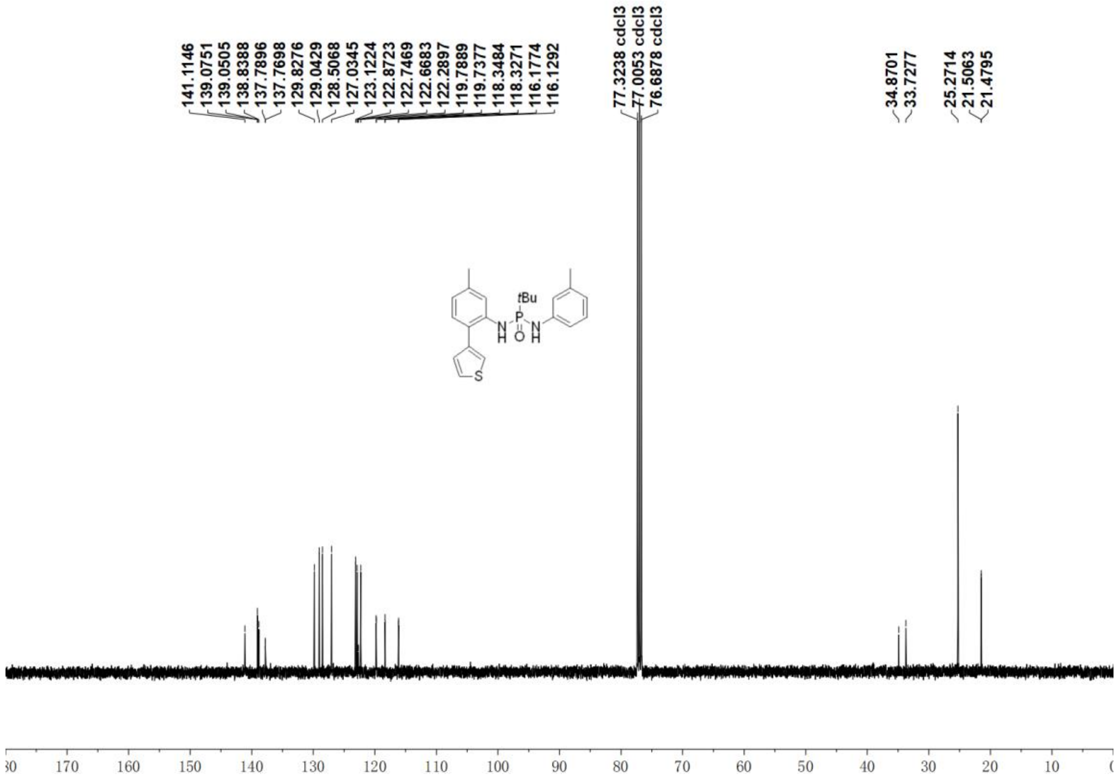

Supplementary Figure 129. ^{13}C NMR spectrum of **b28**

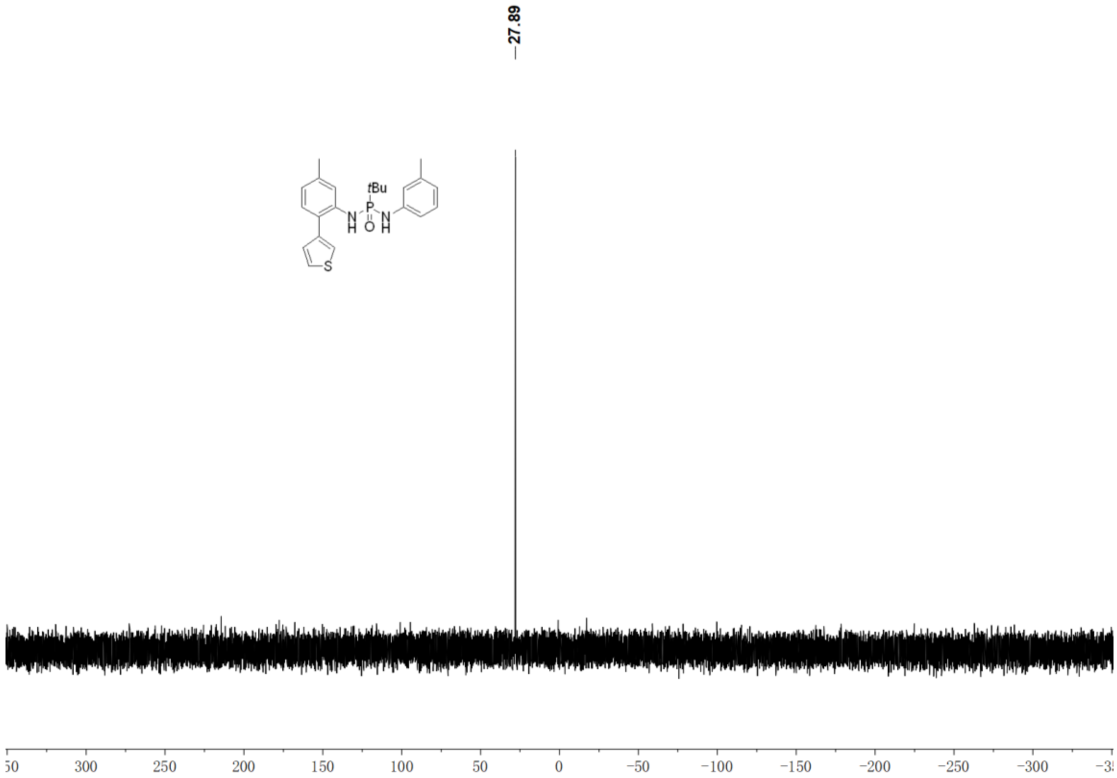

Supplementary Figure 130. ^{31}P NMR spectrum of **b28**

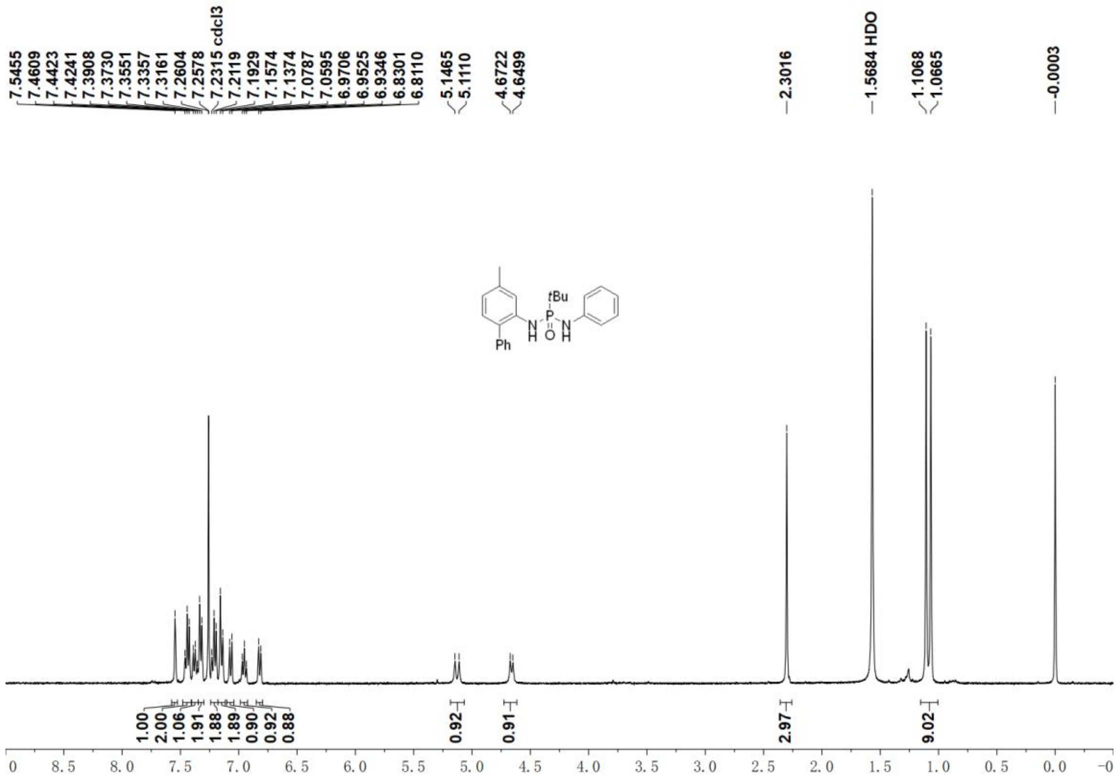

Supplementary Figure 131. ^{1}H NMR spectrum of **b29**

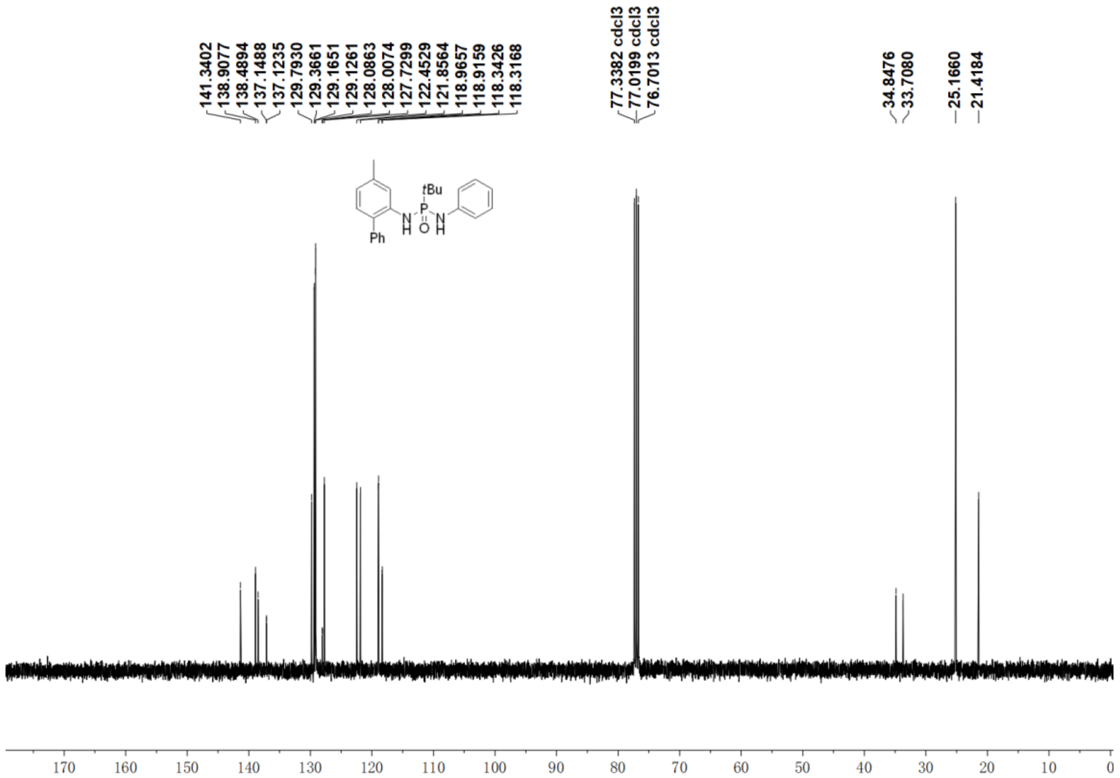

Supplementary Figure 132. ^{13}C NMR spectrum of **b29**

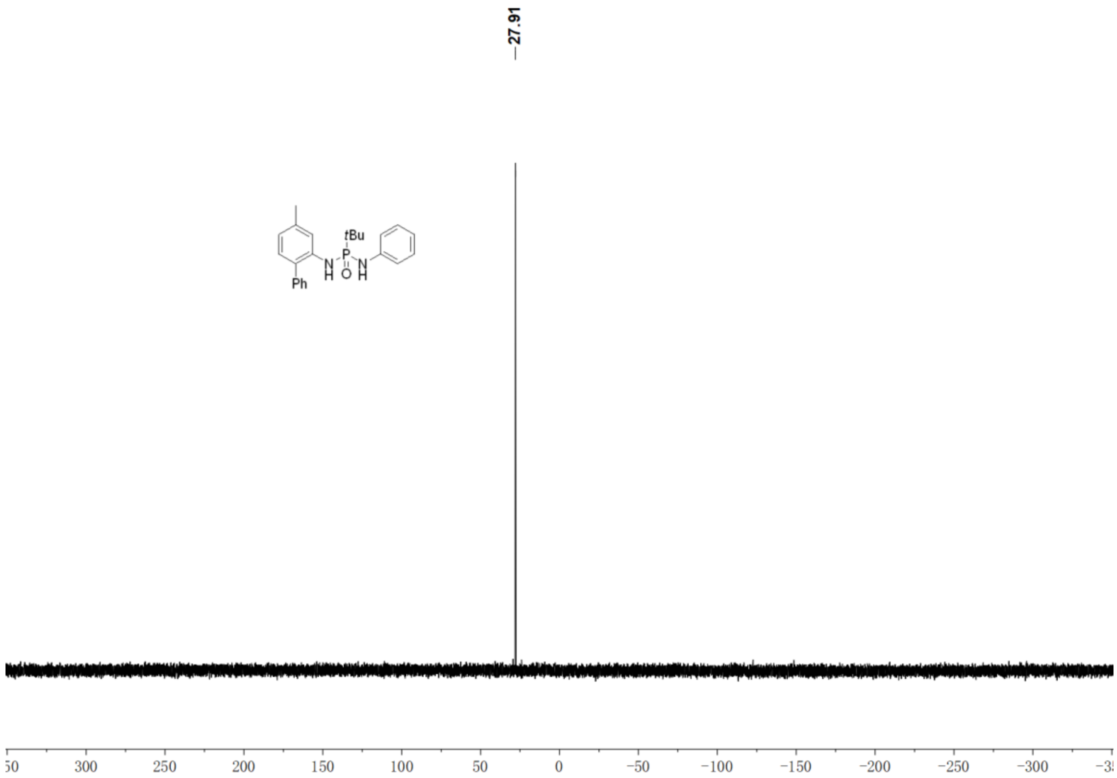

Supplementary Figure 133. ^{31}P NMR spectrum of **b29**

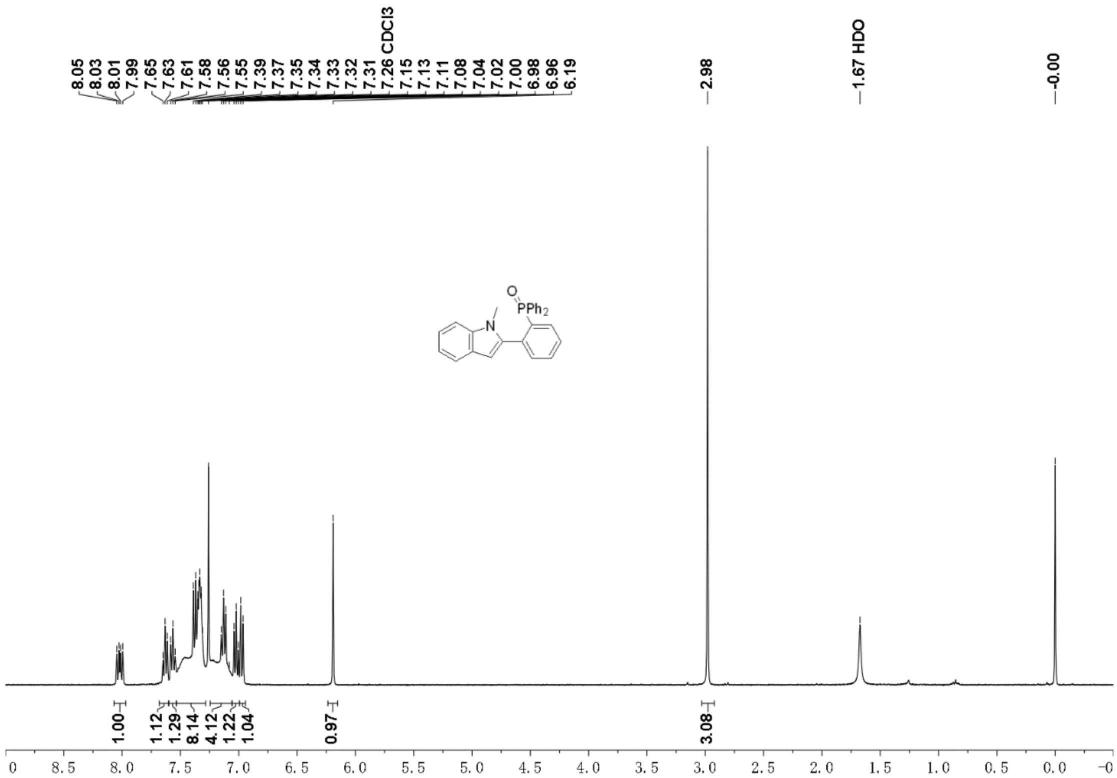

Supplementary Figure 134. ^{1}H NMR spectrum of **c1**

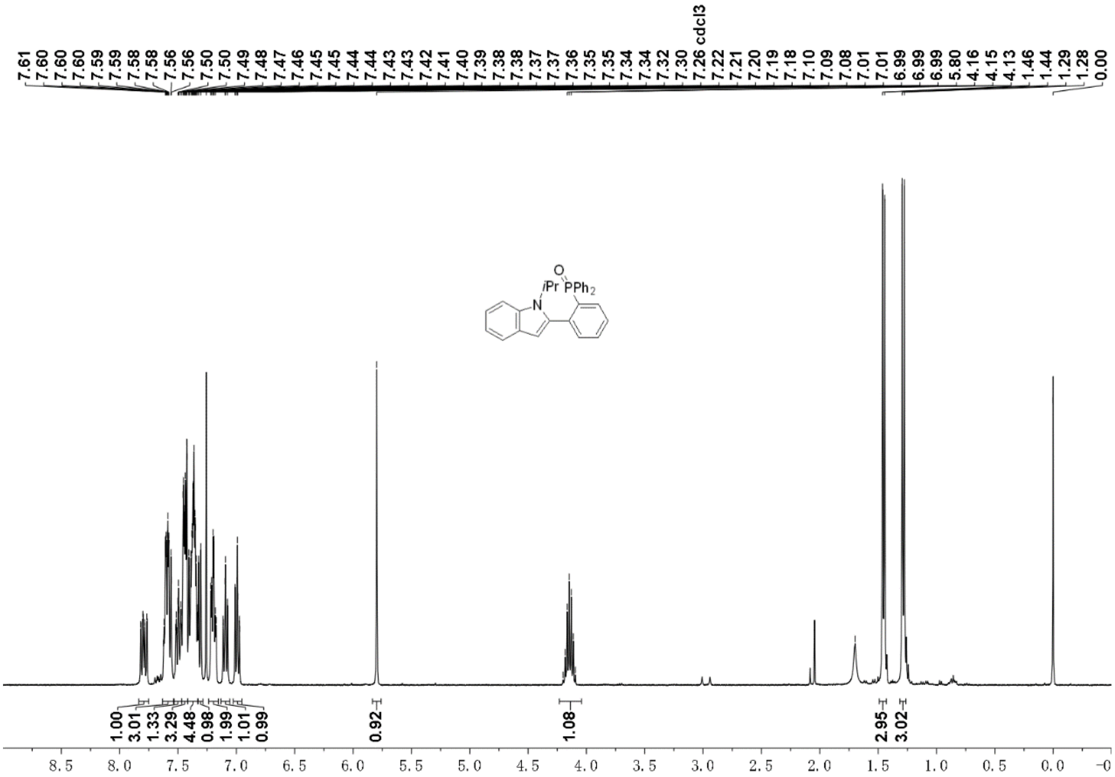

Supplementary Figure 135. ^{1}H NMR spectrum of **c2**

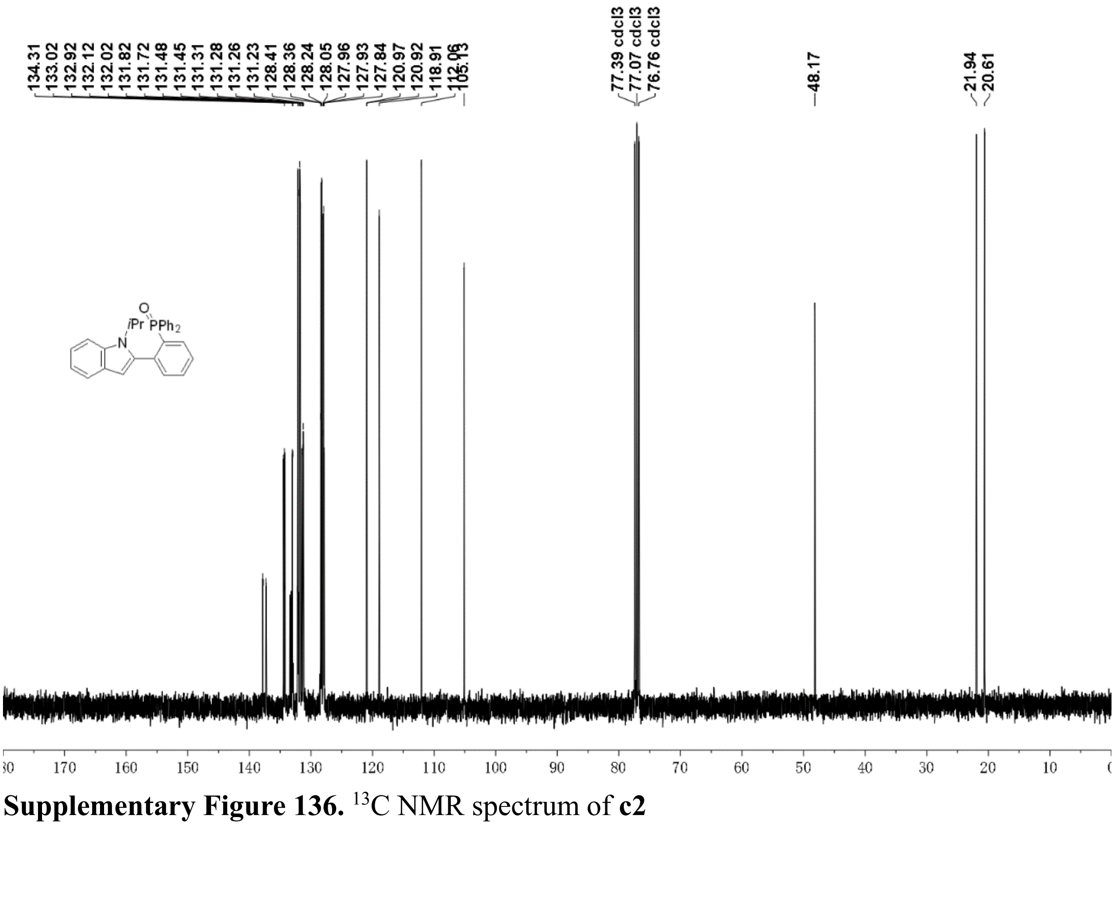

Supplementary Figure 136. ^{13}C NMR spectrum of **c2**

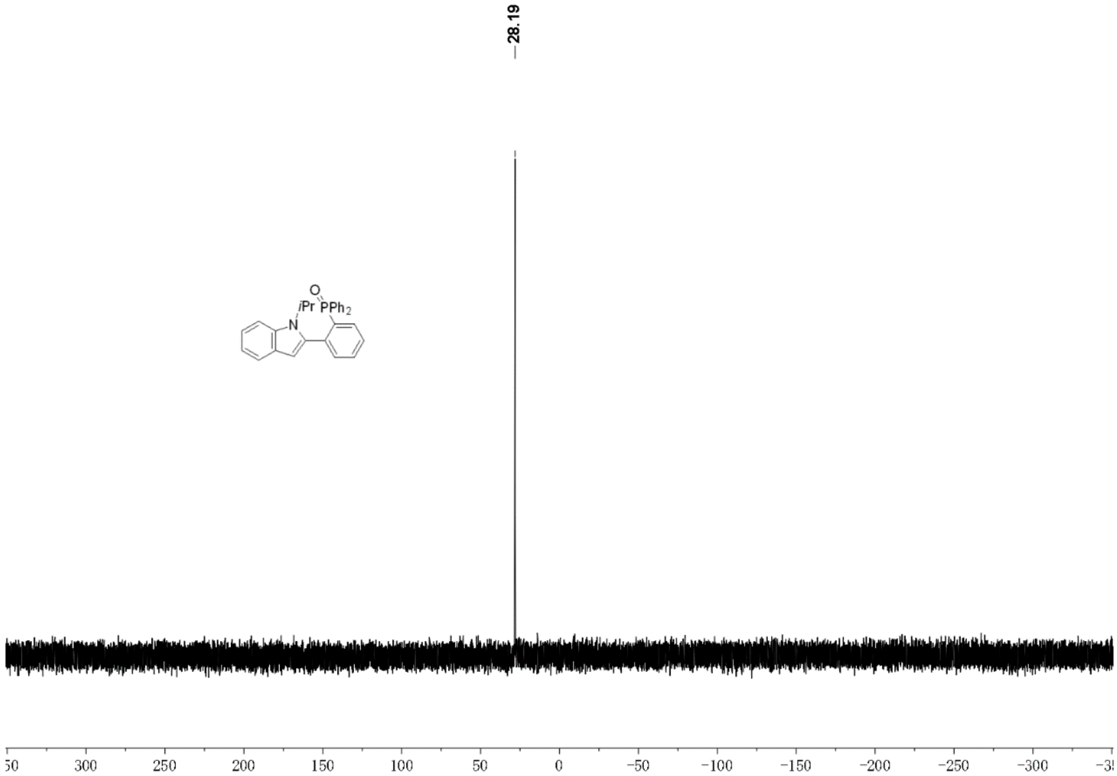

Supplementary Figure 137. ^{31}P NMR spectrum of **c2**

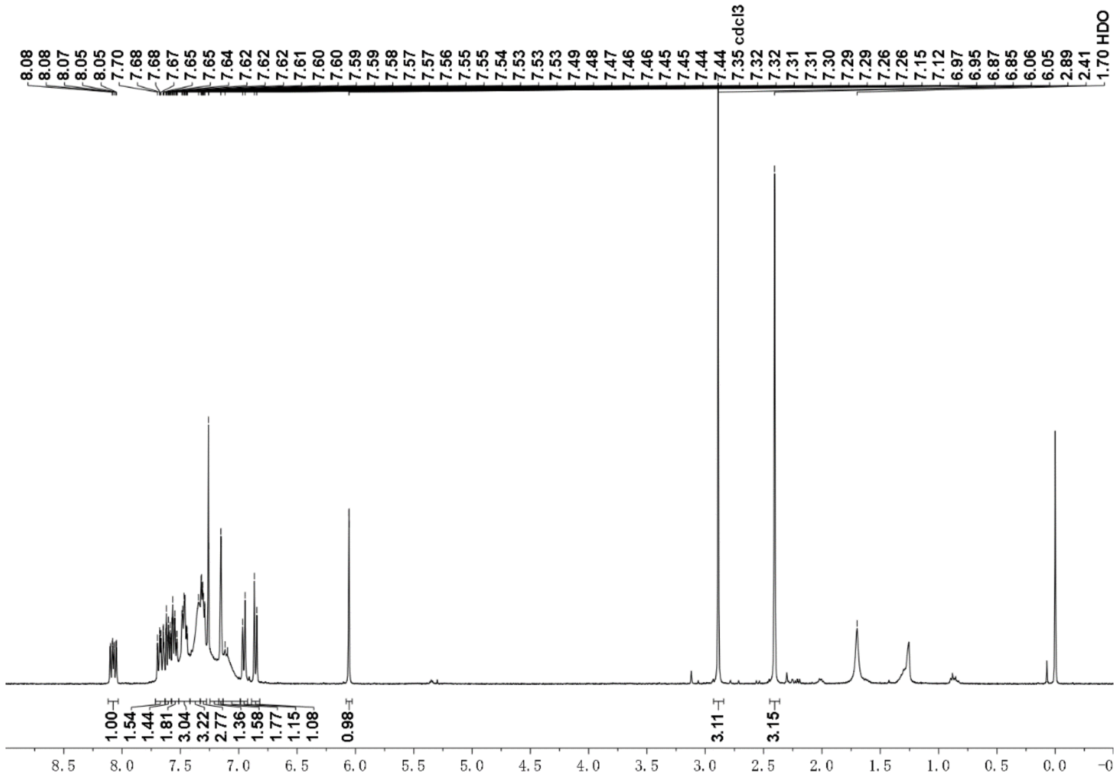

Supplementary Figure 138. ^{1}H NMR spectrum of **c3**

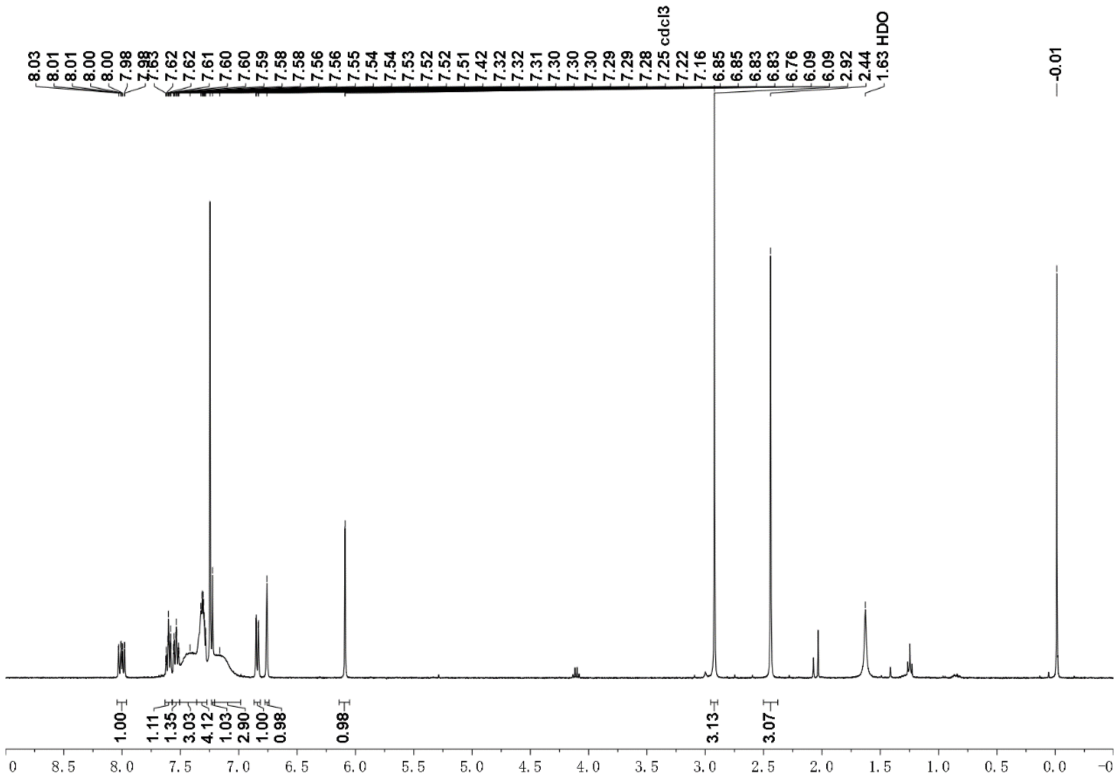

Supplementary Figure 139. ^{1}H NMR spectrum of **c4**

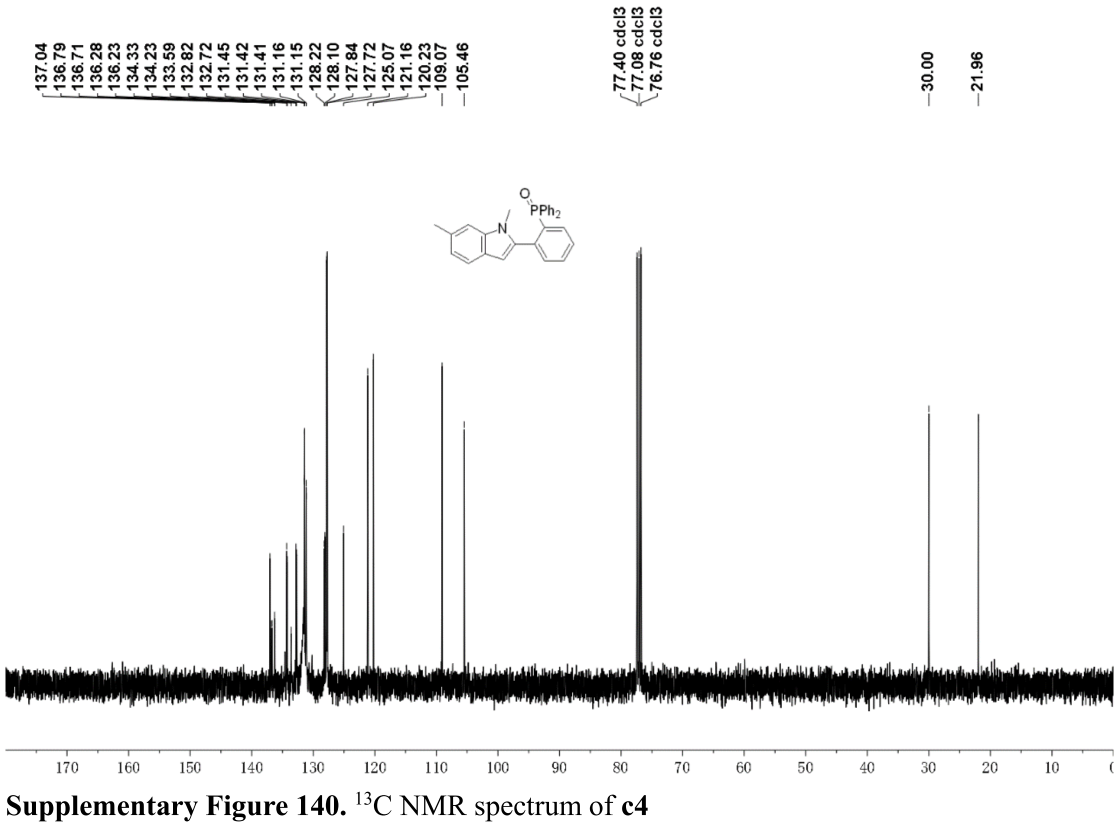

Supplementary Figure 140. ^{13}C NMR spectrum of **c4**

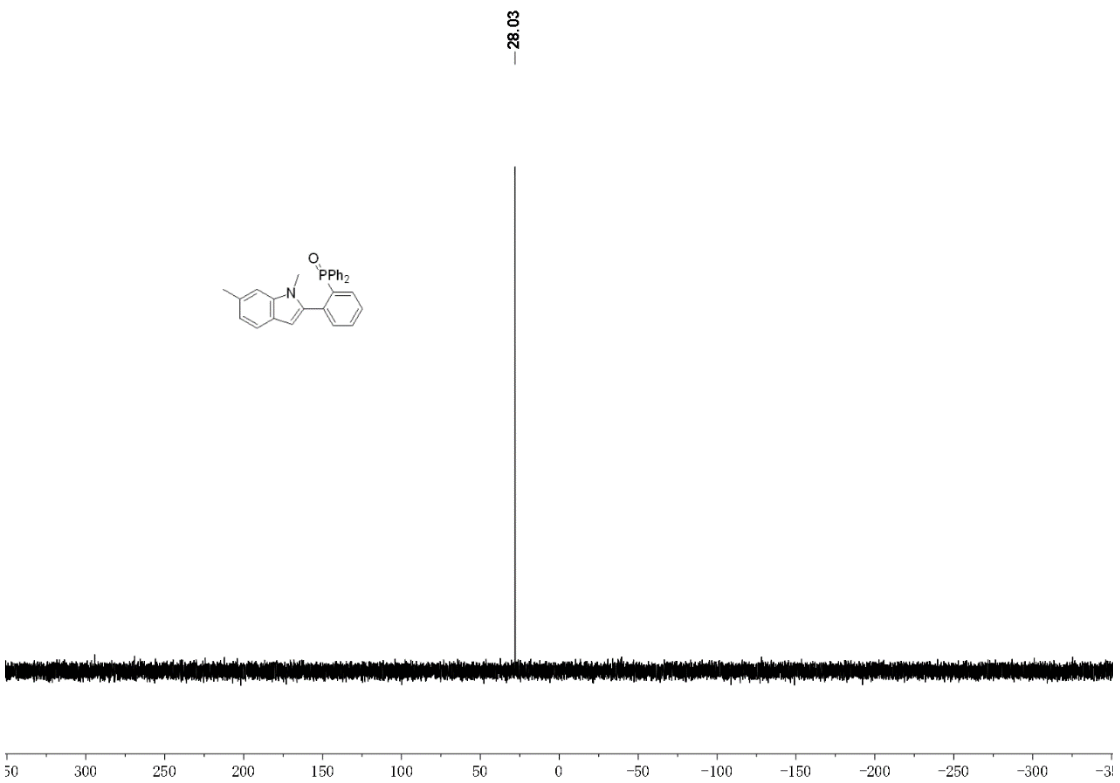

Supplementary Figure 141. ^{31}P NMR spectrum of **c4**

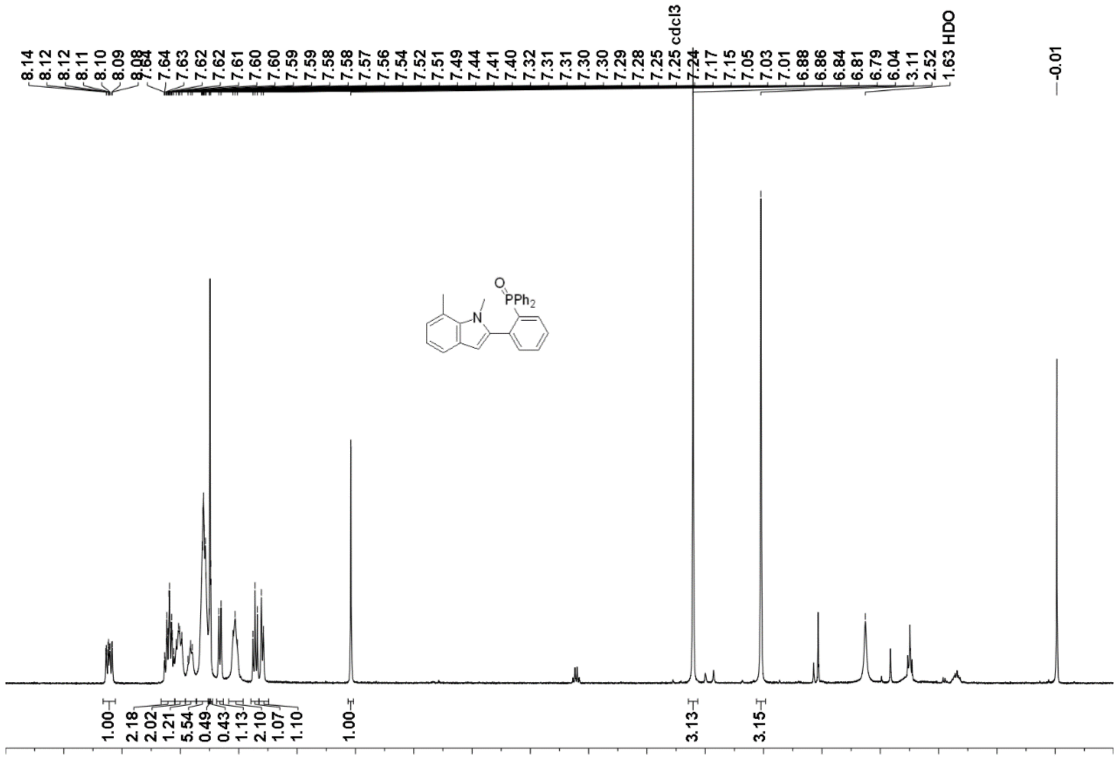

Supplementary Figure 142. ^{1}H NMR spectrum of **c5**

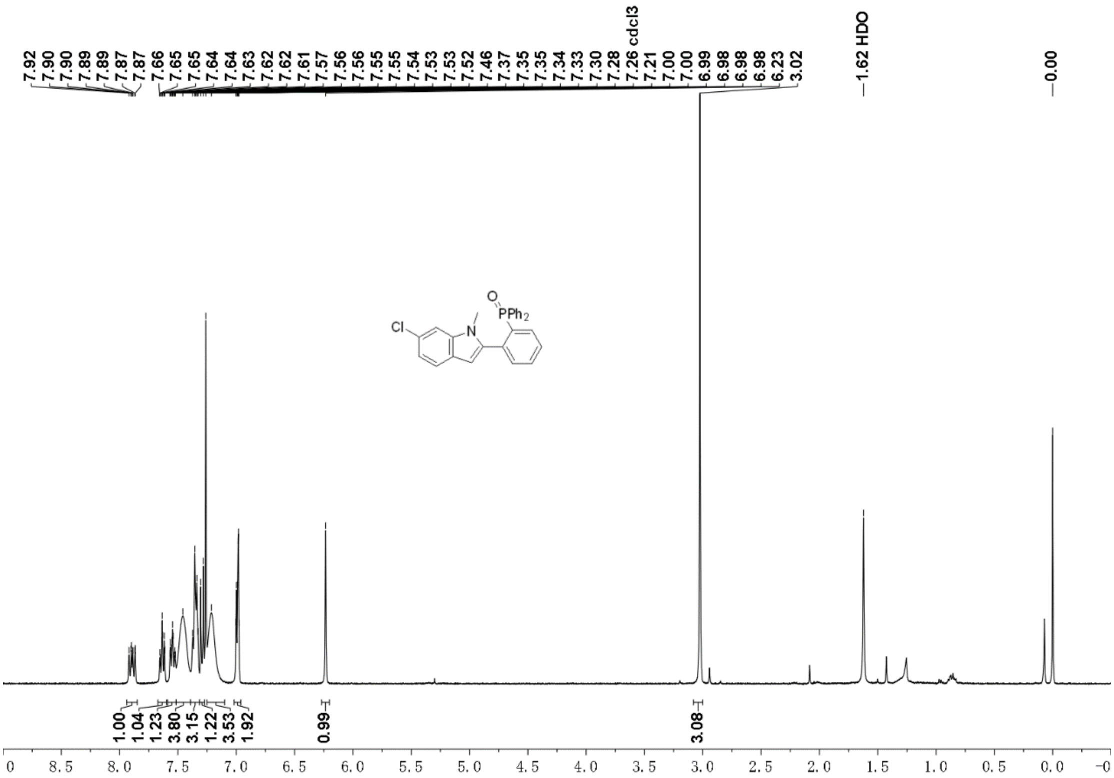

Supplementary Figure 143. ^{1}H NMR spectrum of **c6**

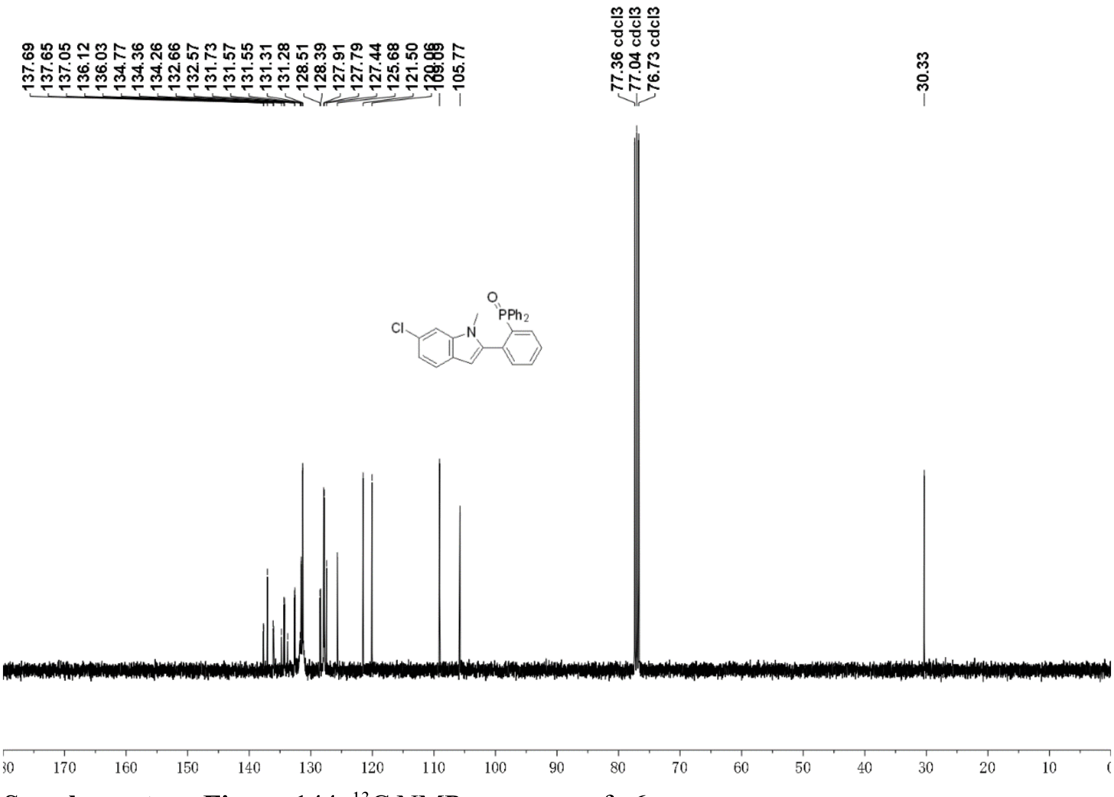

Supplementary Figure 144. ^{13}C NMR spectrum of **c6**

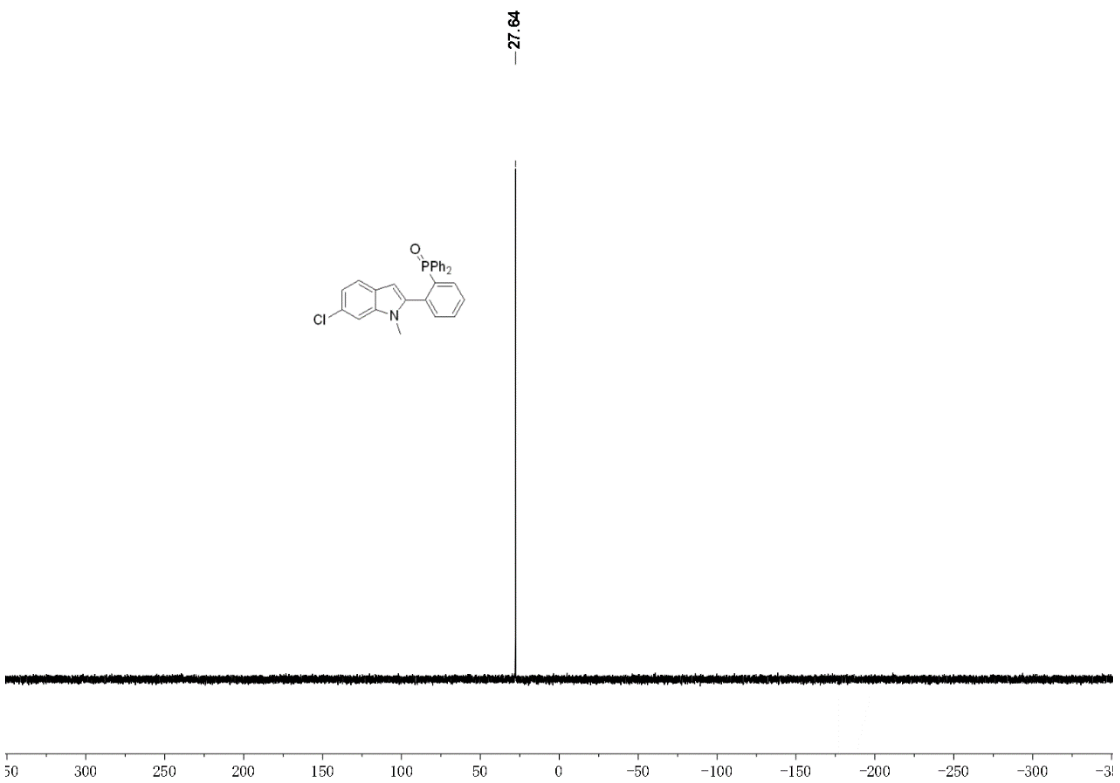

Supplementary Figure 145. ^{31}P NMR spectrum of **c6**

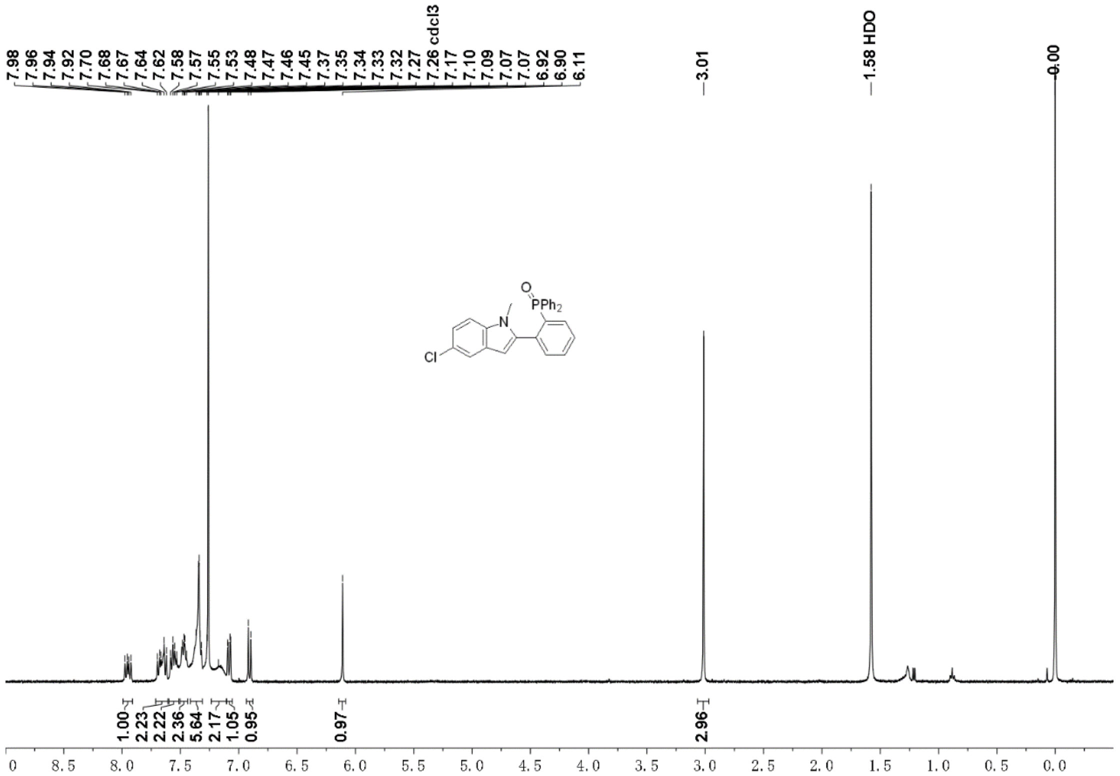

Supplementary Figure 146. ^{1}H NMR spectrum of **c7**

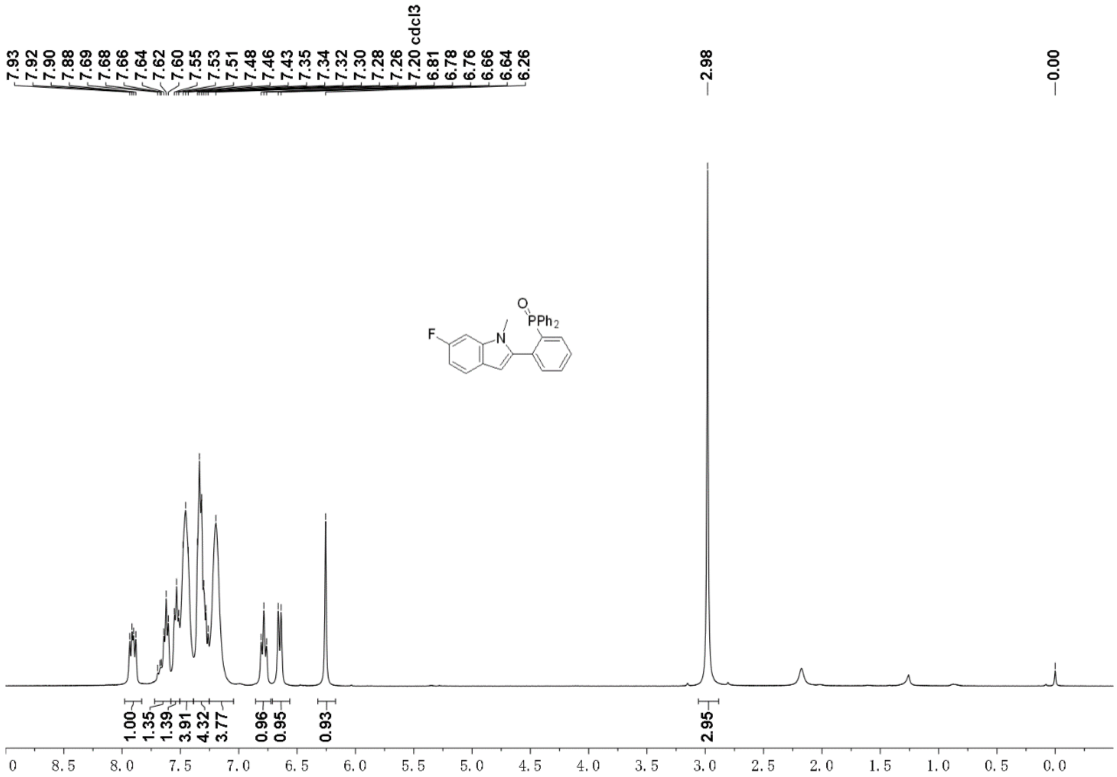

Supplementary Figure 147. ^{1}H NMR spectrum of **c8**

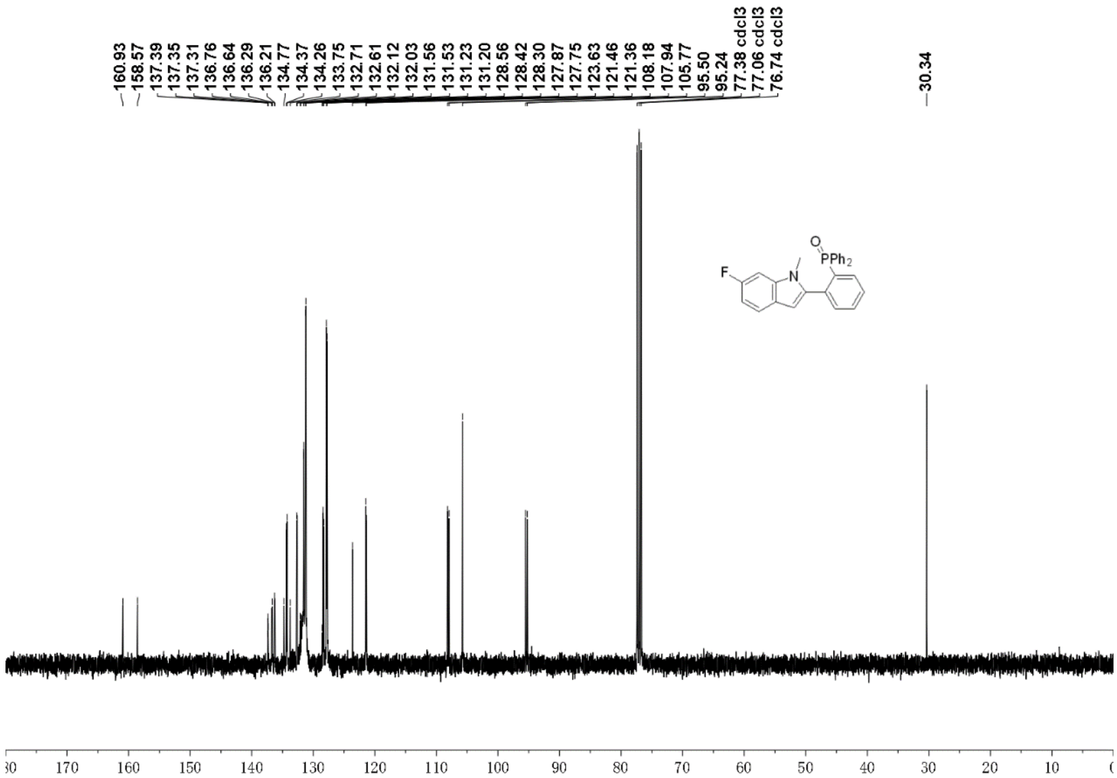

Supplementary Figure 148. ^{13}C NMR spectrum of **c8**

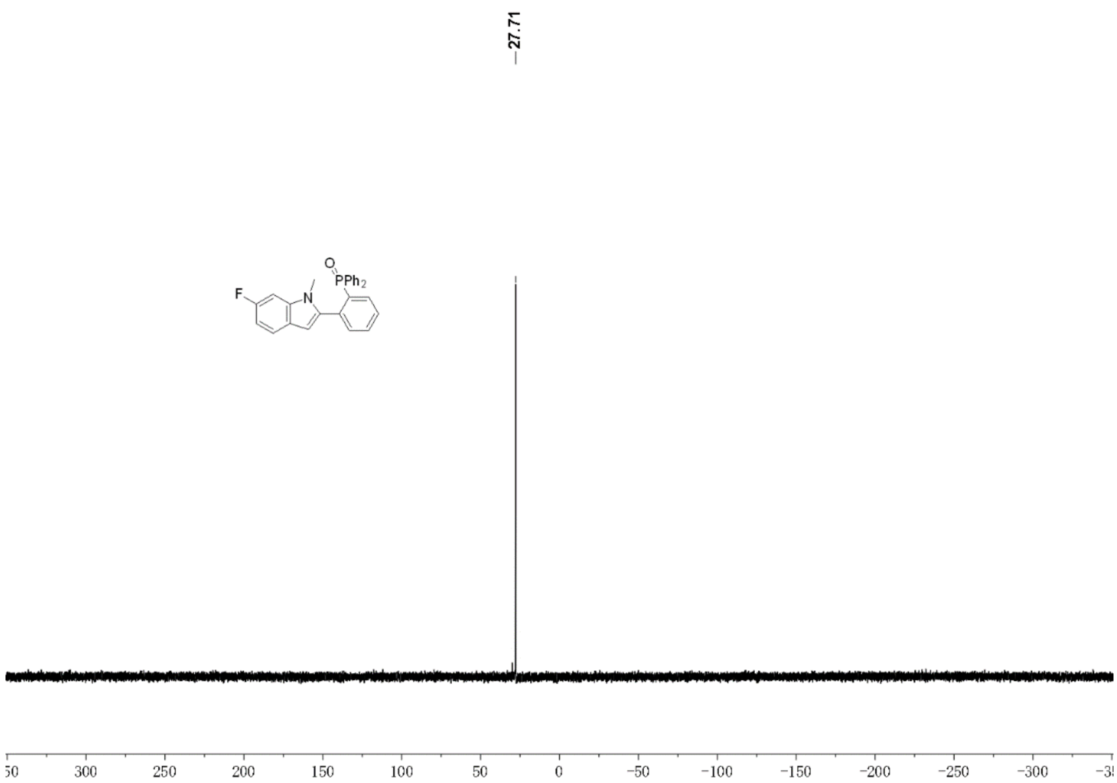

Supplementary Figure 149. ^{31}P NMR spectrum of **c8**

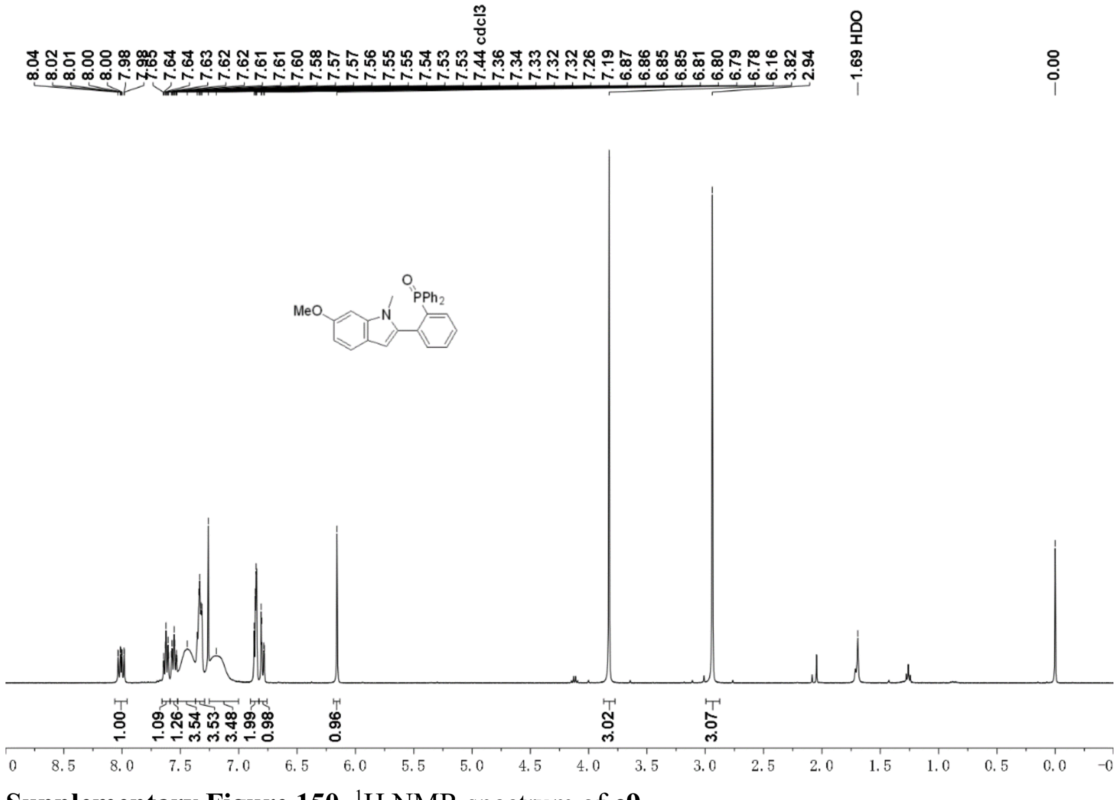

Supplementary Figure 150. ^{1}H NMR spectrum of **c9**

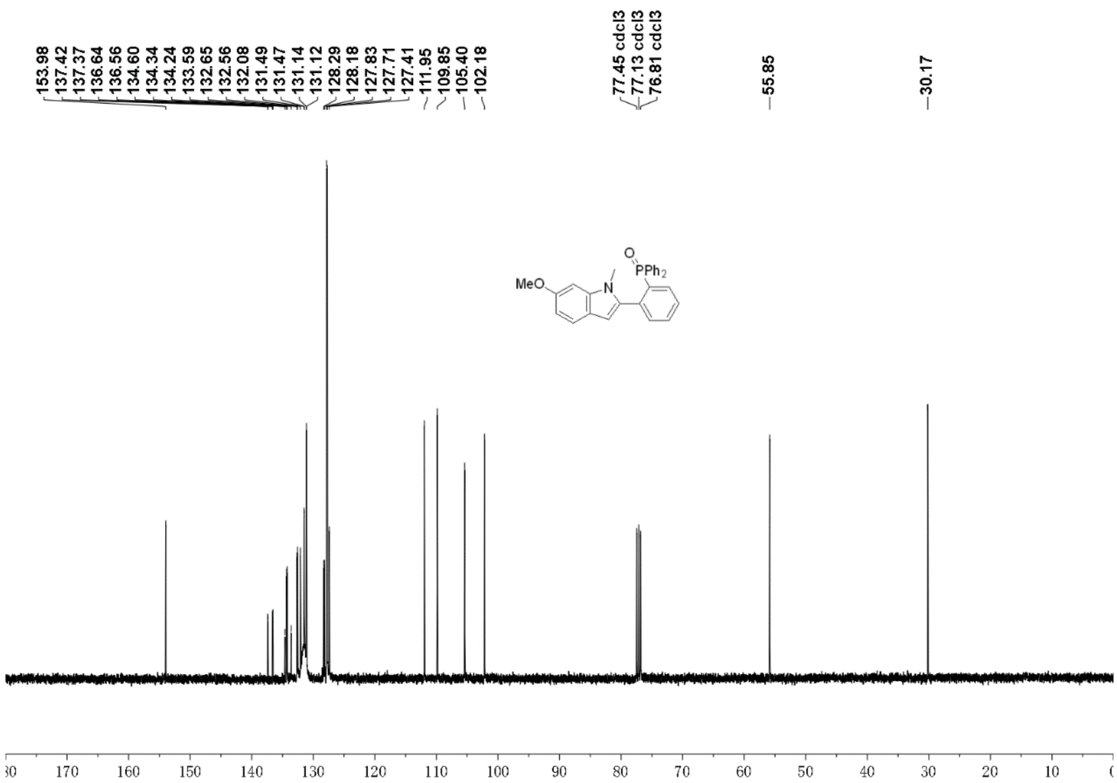

Supplementary Figure 151. ^{13}C NMR spectrum of **c9**

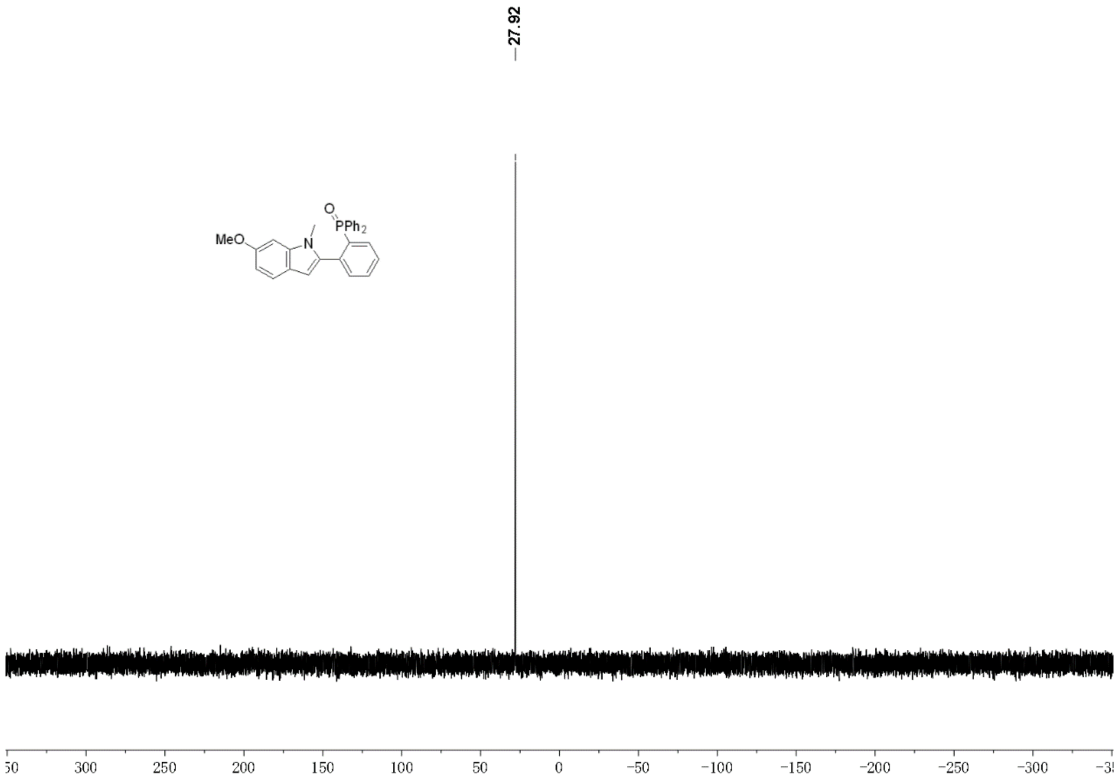

Supplementary Figure 152. ^{31}P NMR spectrum of **c9**

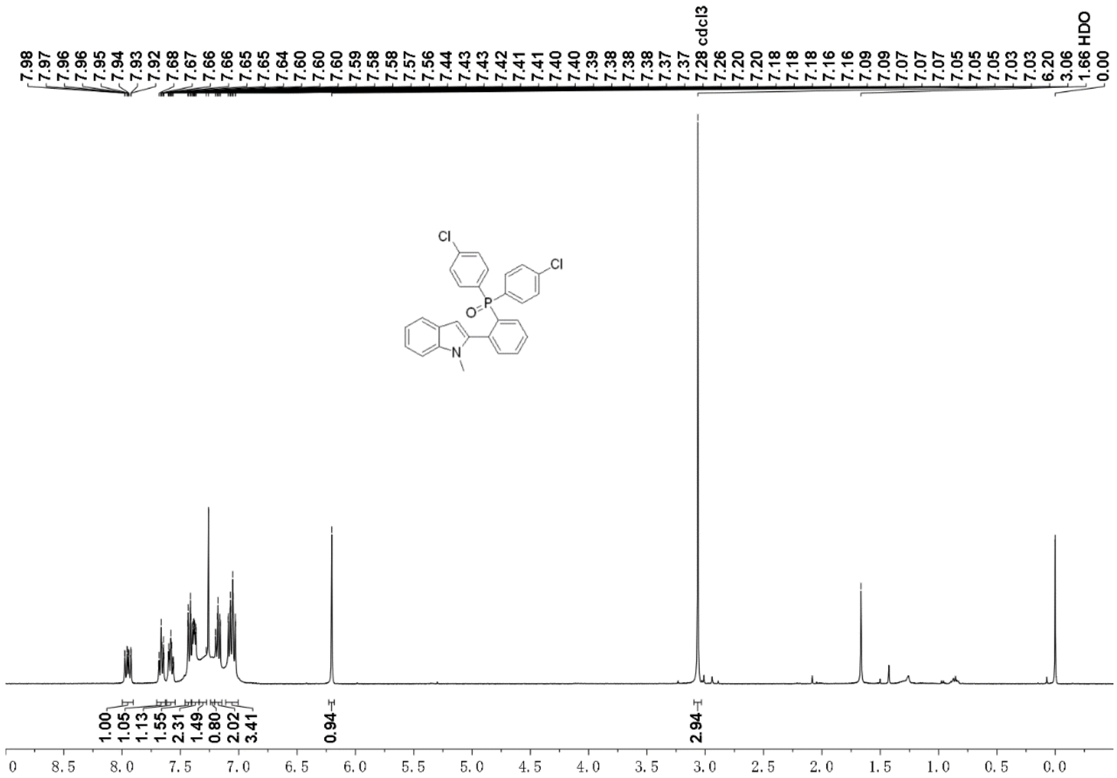

Supplementary Figure 153. ^{1}H NMR spectrum of **c10**

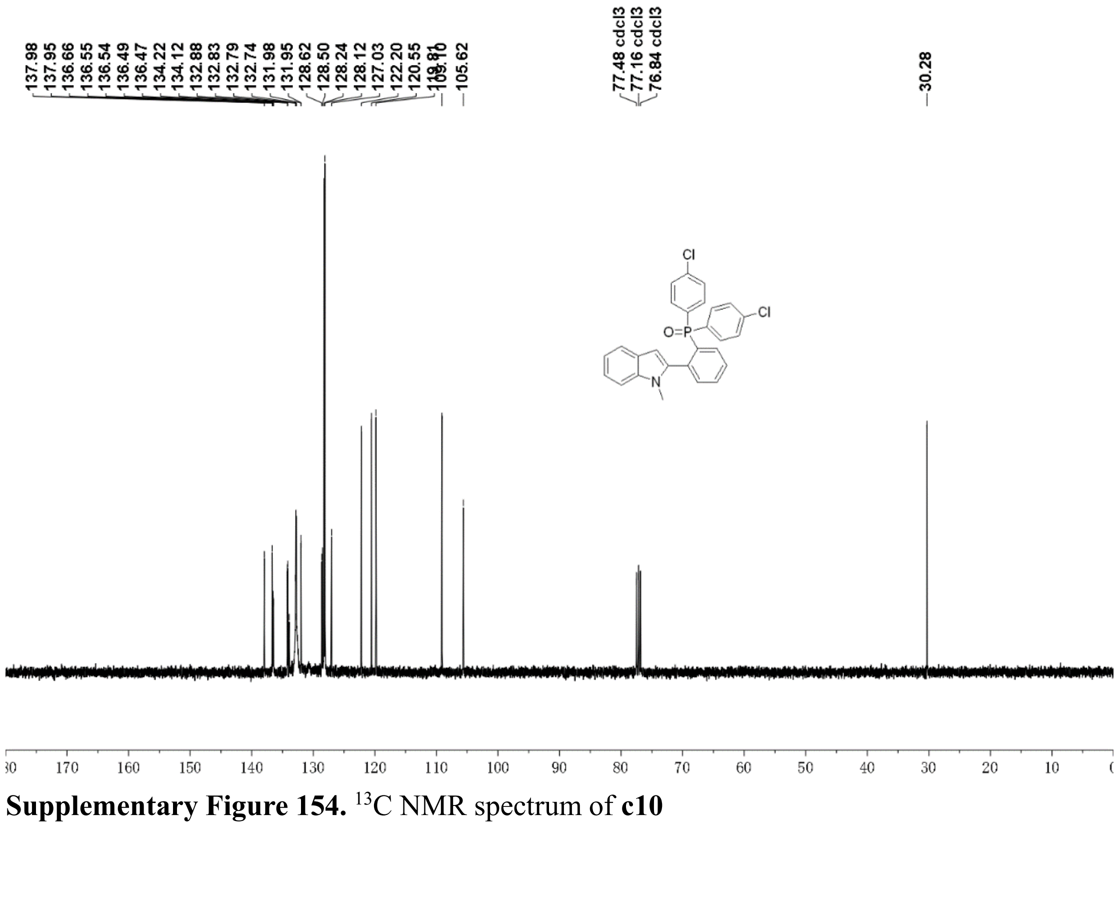

Supplementary Figure 154. ^{13}C NMR spectrum of **c10**

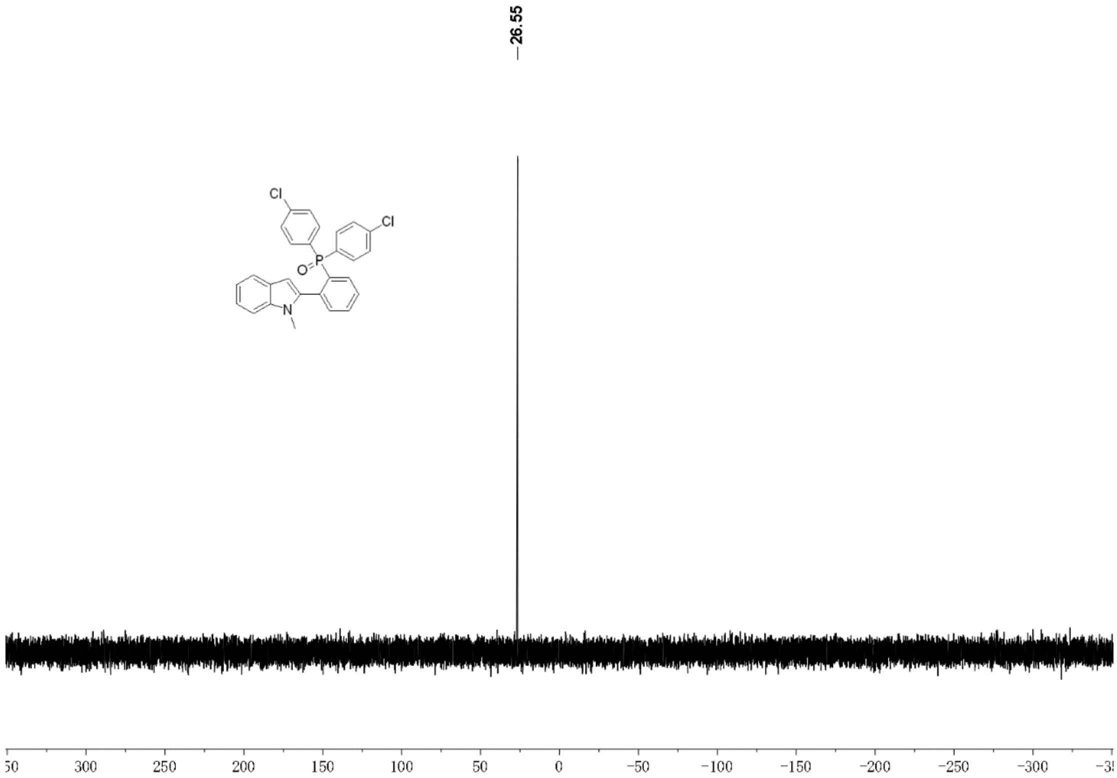

Supplementary Figure 155. ^{31}P NMR spectrum of **c10**

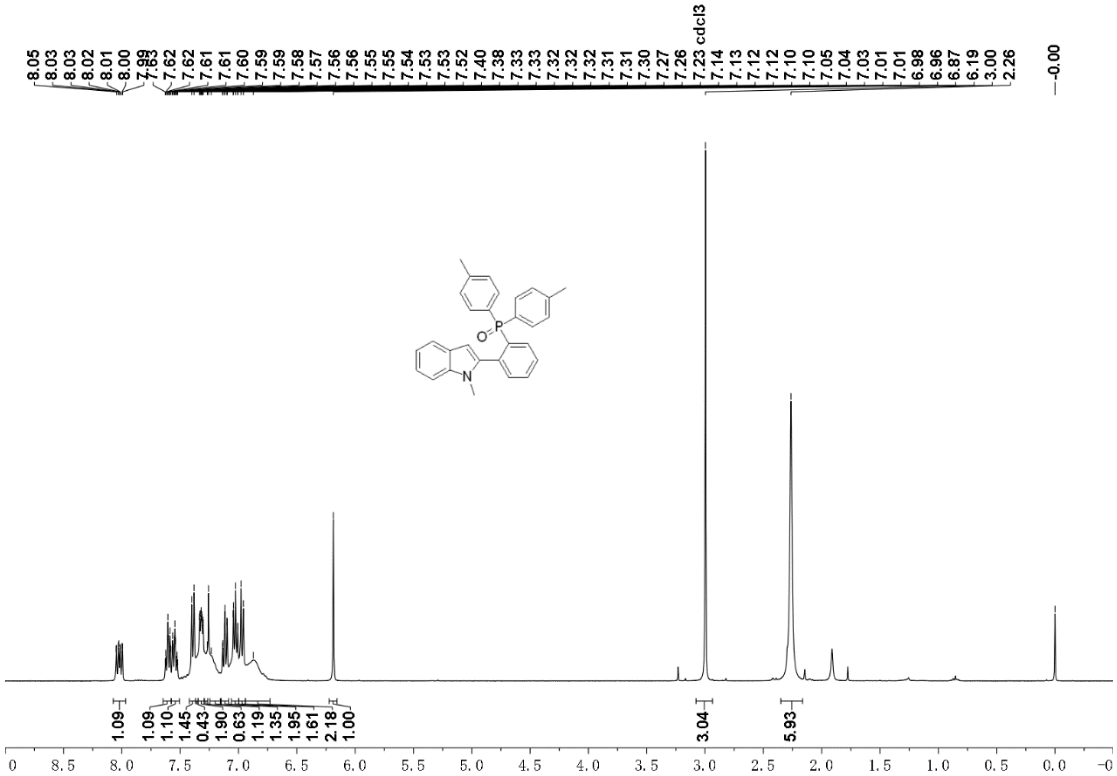

Supplementary Figure 156. ^{1}H NMR spectrum of **c11**

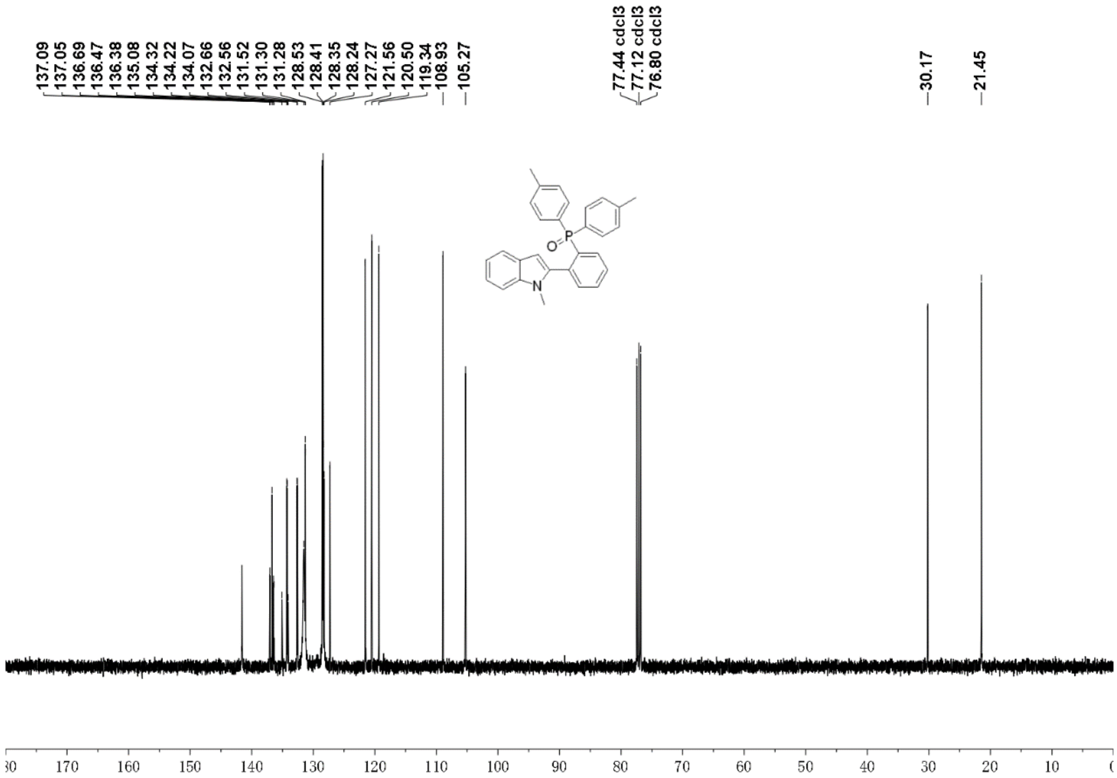

Supplementary Figure 157. ^{13}C NMR spectrum of **c11**

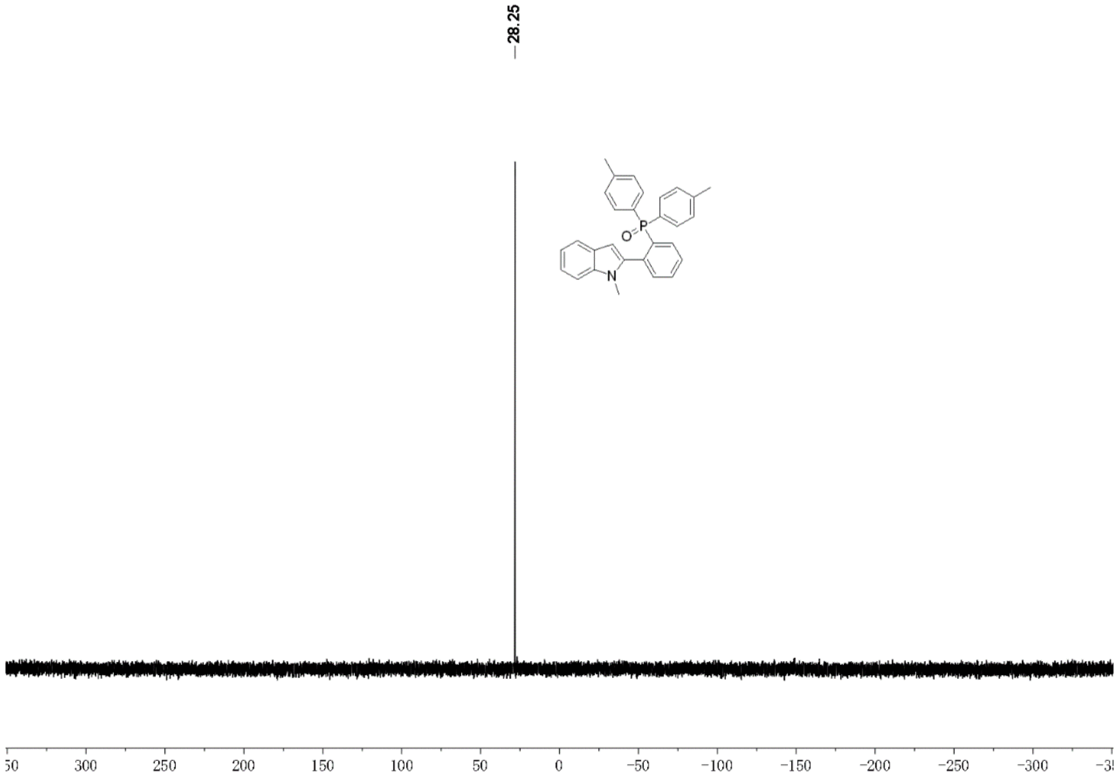

Supplementary Figure 158. ^{31}P NMR spectrum of **c11**

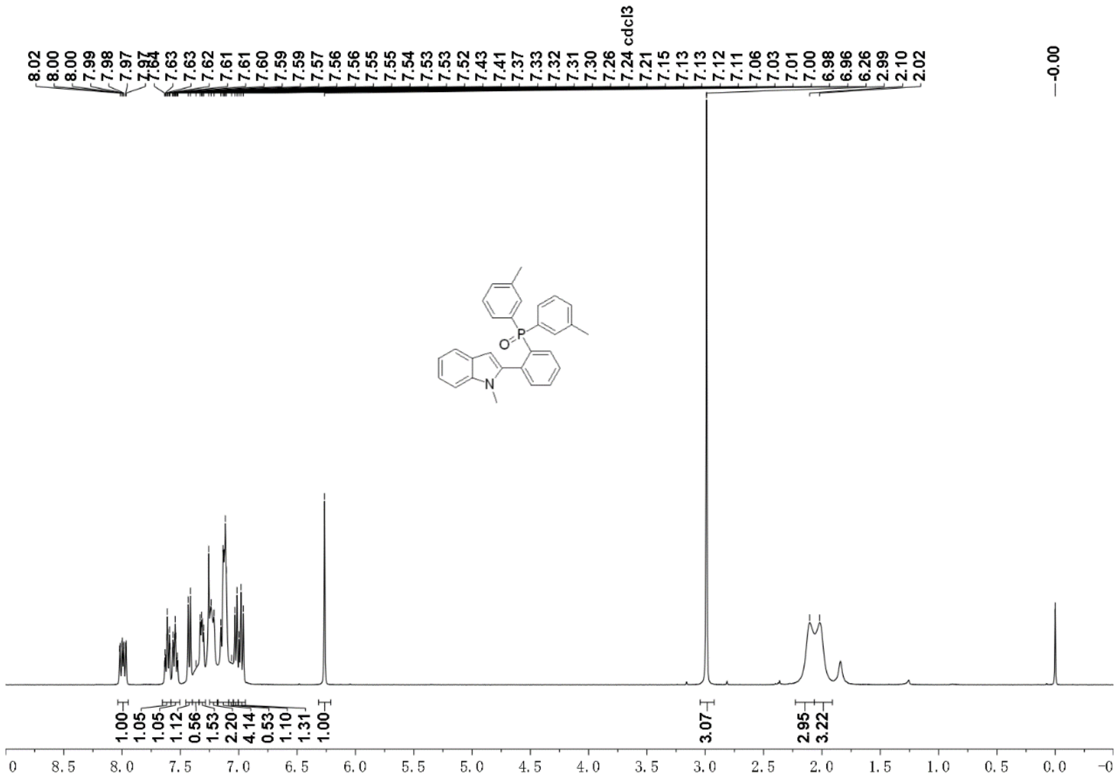

Supplementary Figure 159. ^{1}H NMR spectrum of **c12**

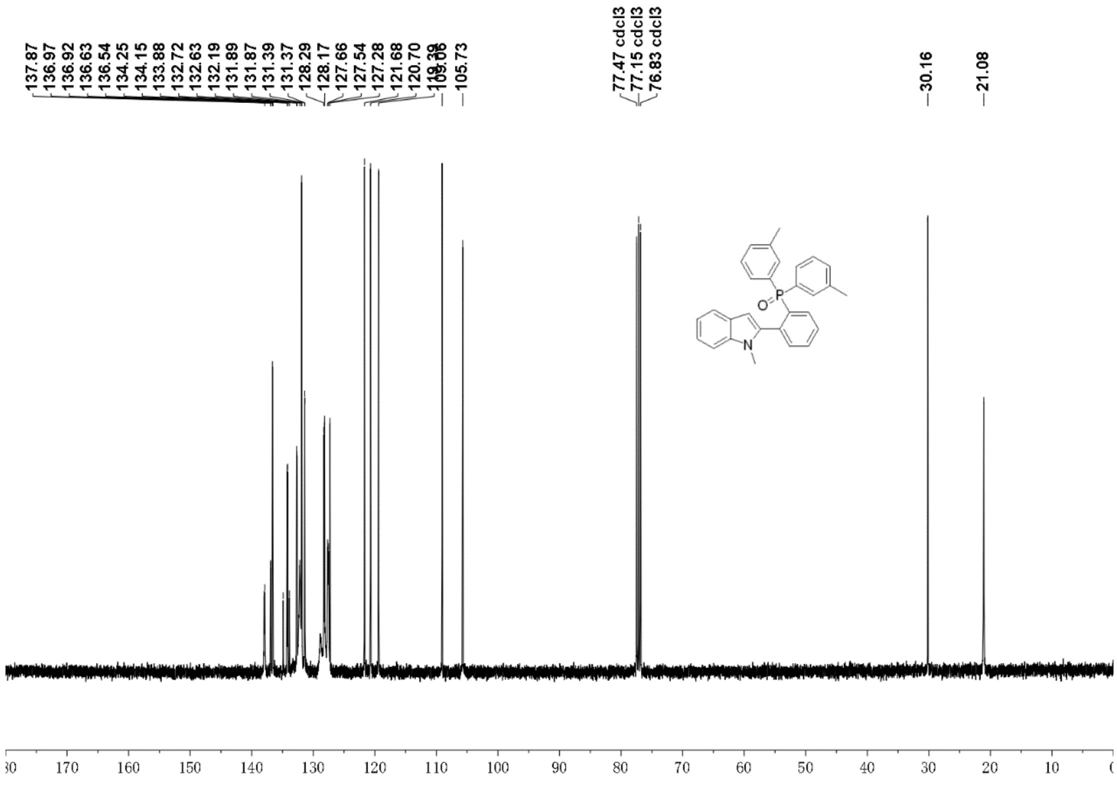

Supplementary Figure 160. ^{13}C NMR spectrum of **c12**

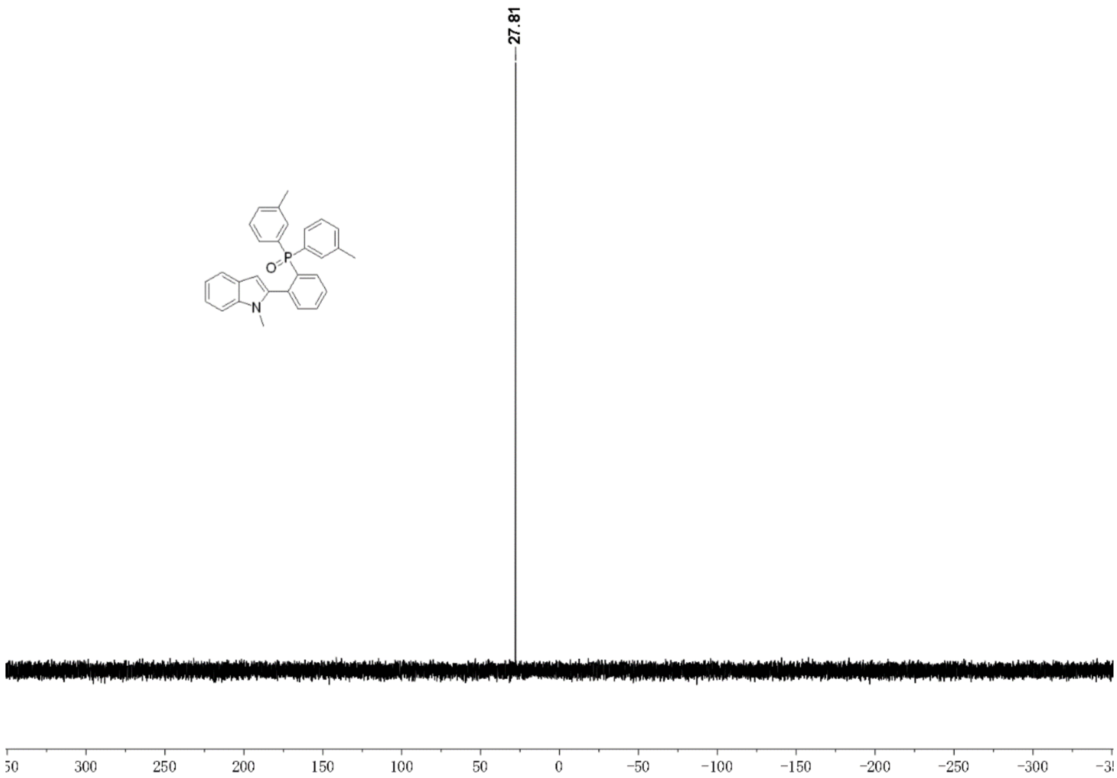

Supplementary Figure 161. ^{31}P NMR spectrum of **c12**

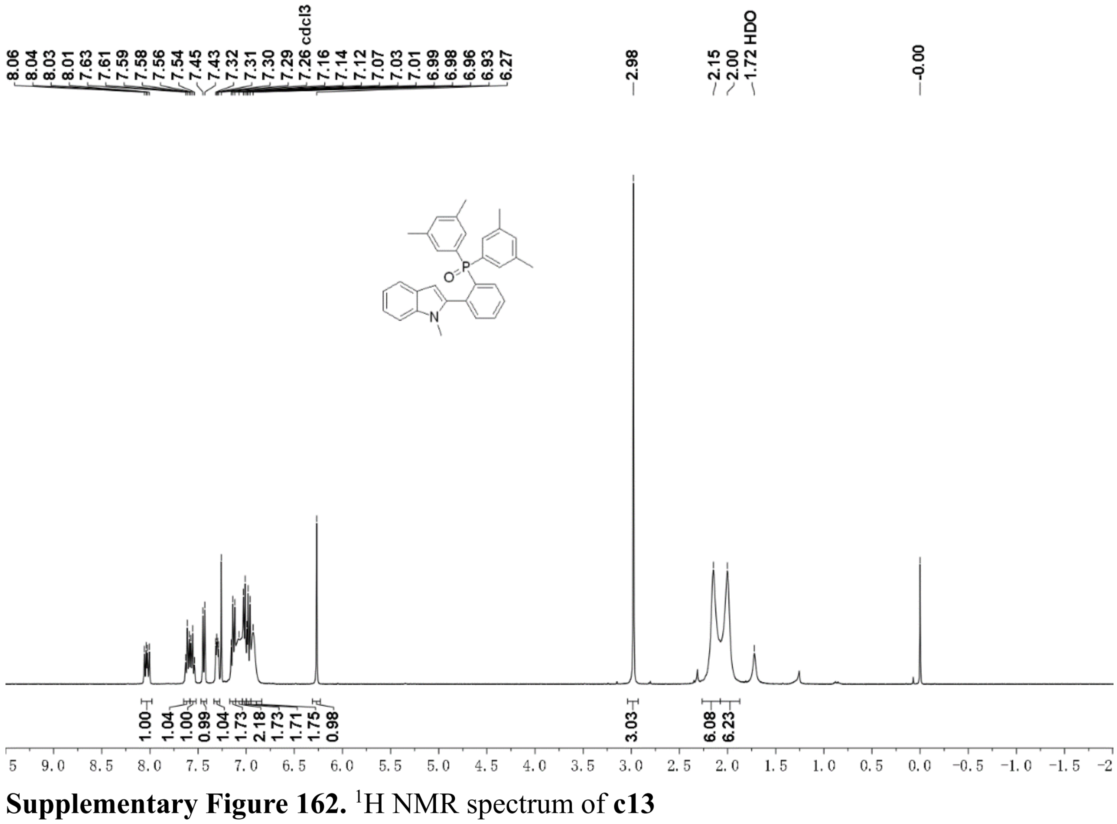

Supplementary Figure 162. ^{1}H NMR spectrum of **c13**

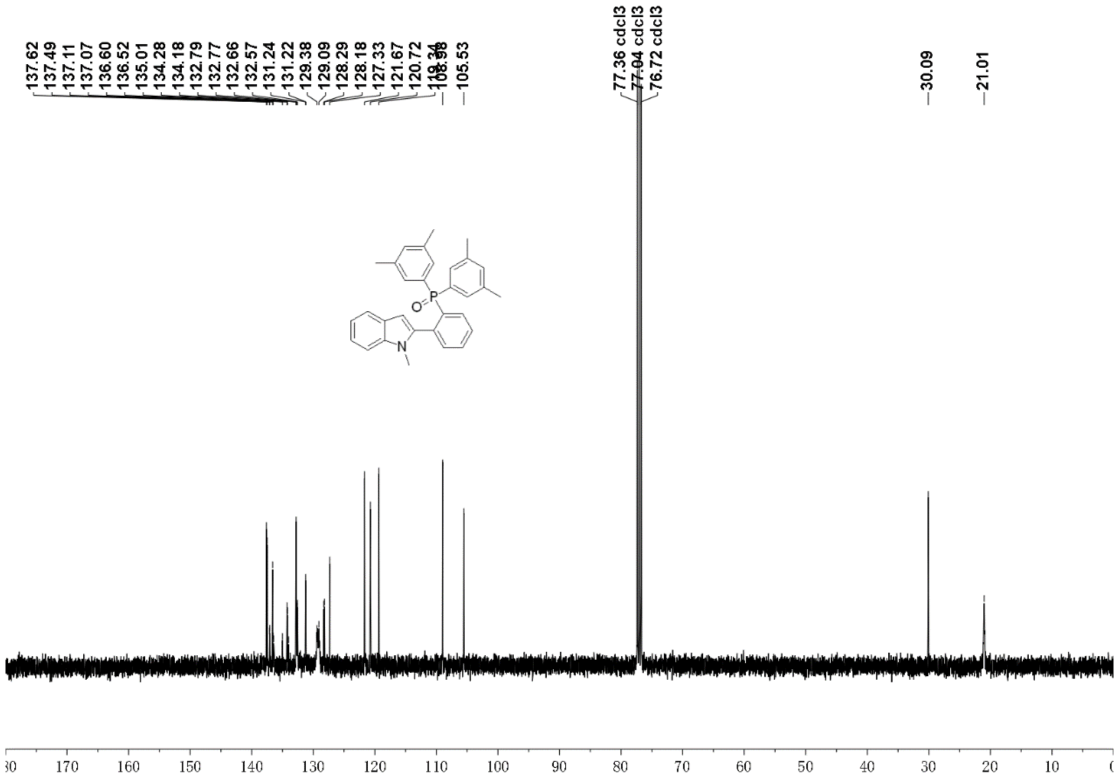

Supplementary Figure 163. ^{13}C NMR spectrum of **c13**

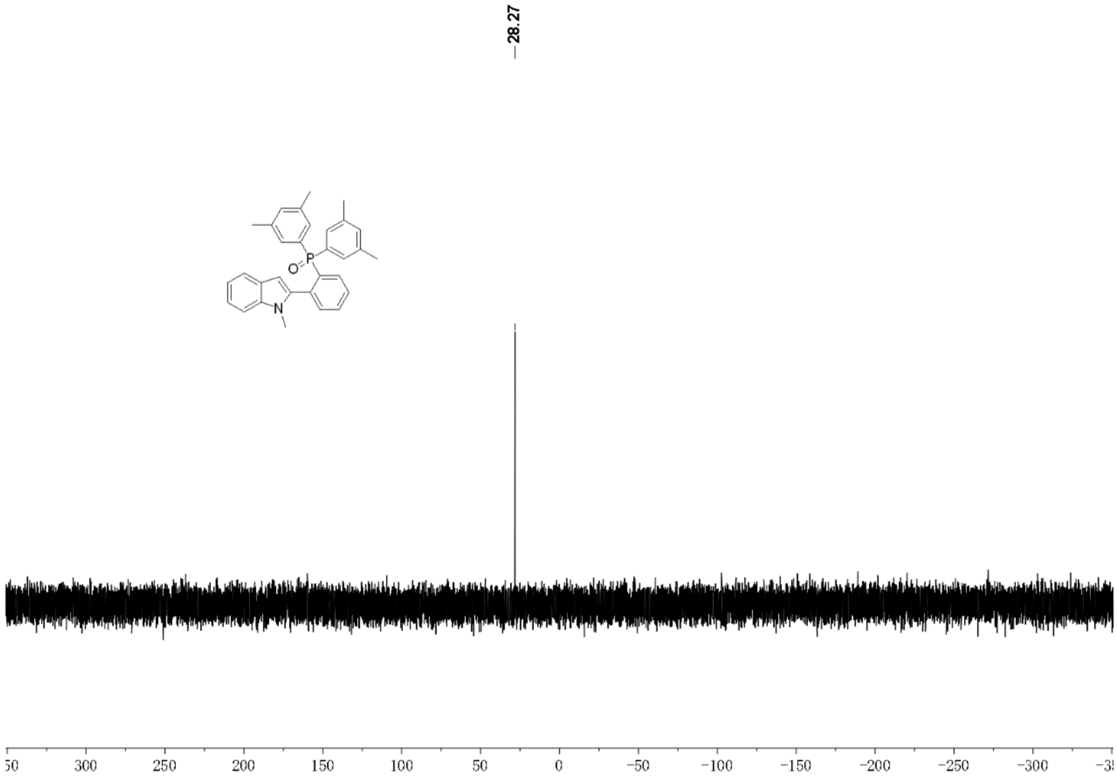

Supplementary Figure 164. ^{31}P NMR spectrum of **c13**

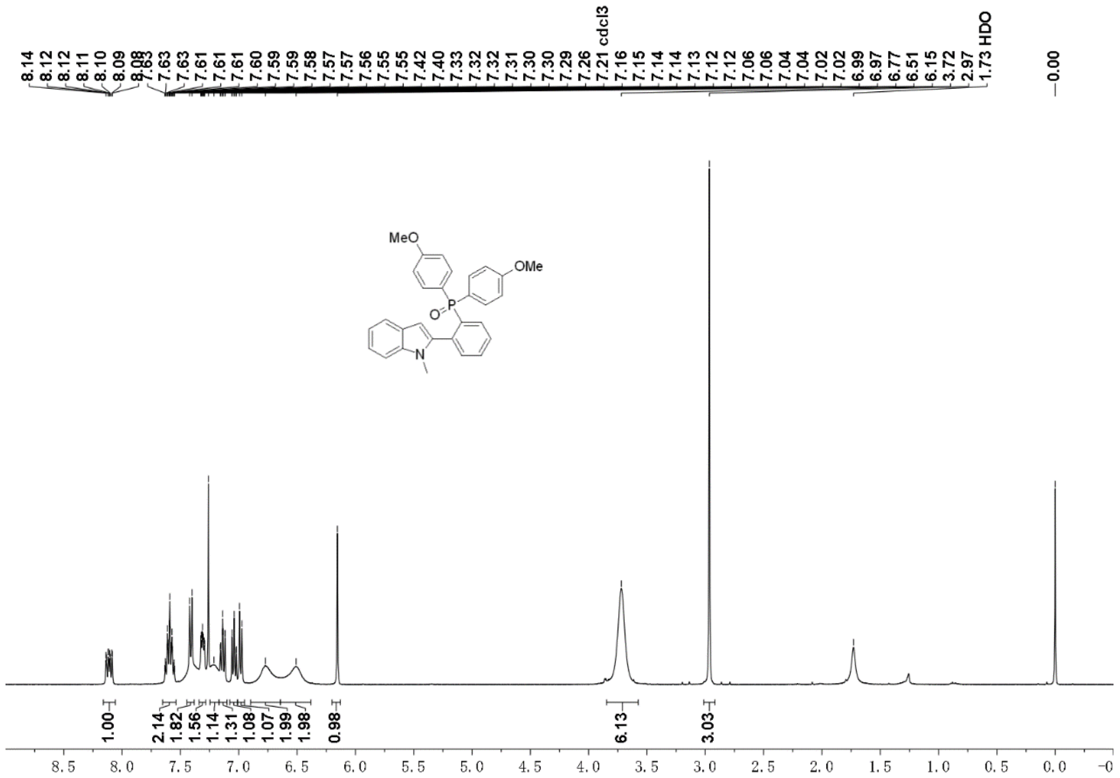

Supplementary Figure 165. ^{1}H NMR spectrum of **c14**

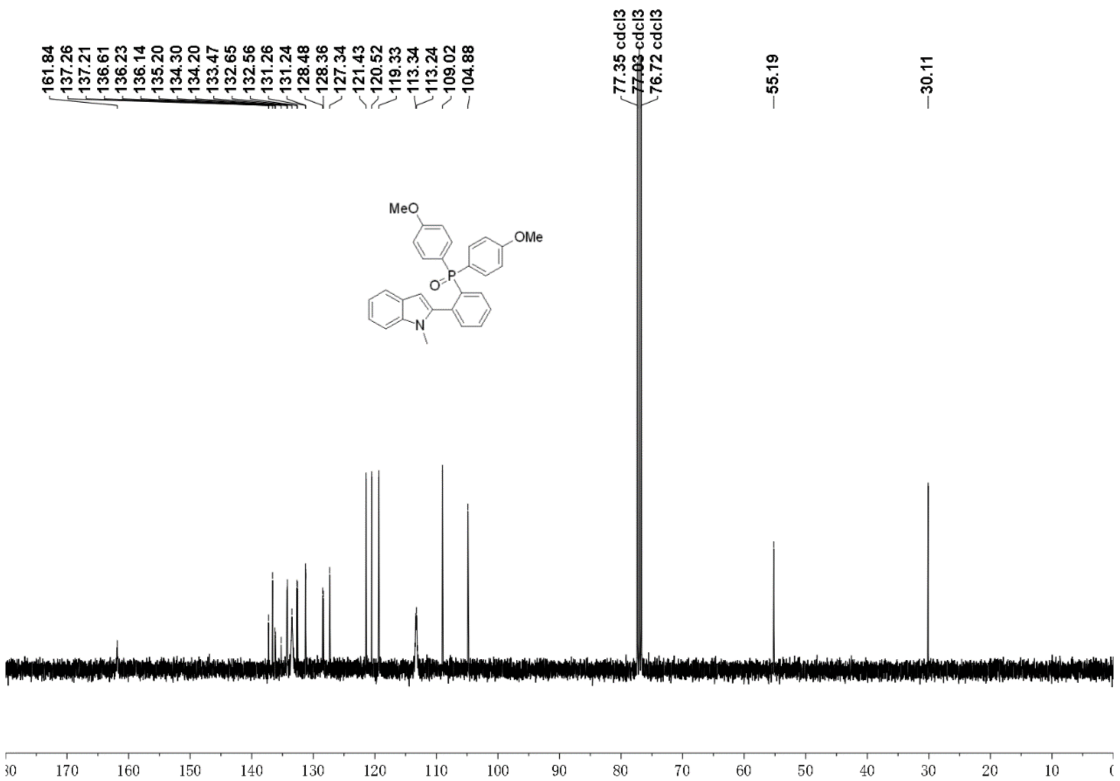

Supplementary Figure 166. ^{13}C NMR spectrum of **c14**

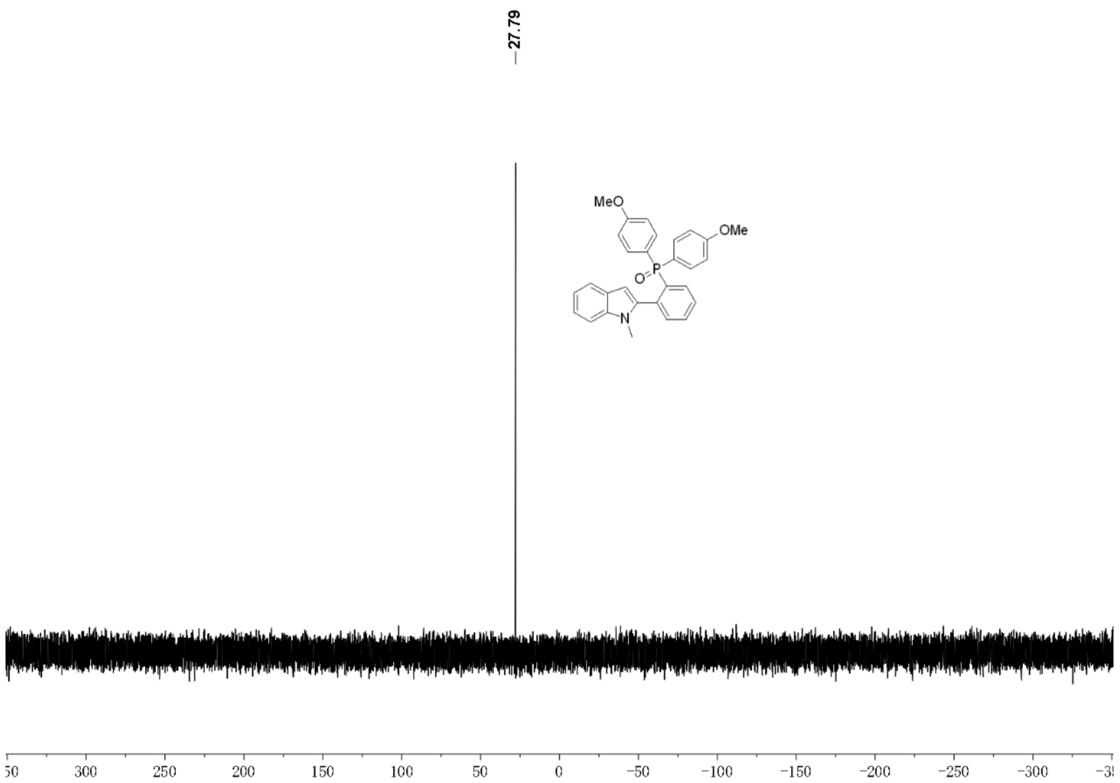

Supplementary Figure 167. ^{31}P NMR spectrum of **c14**

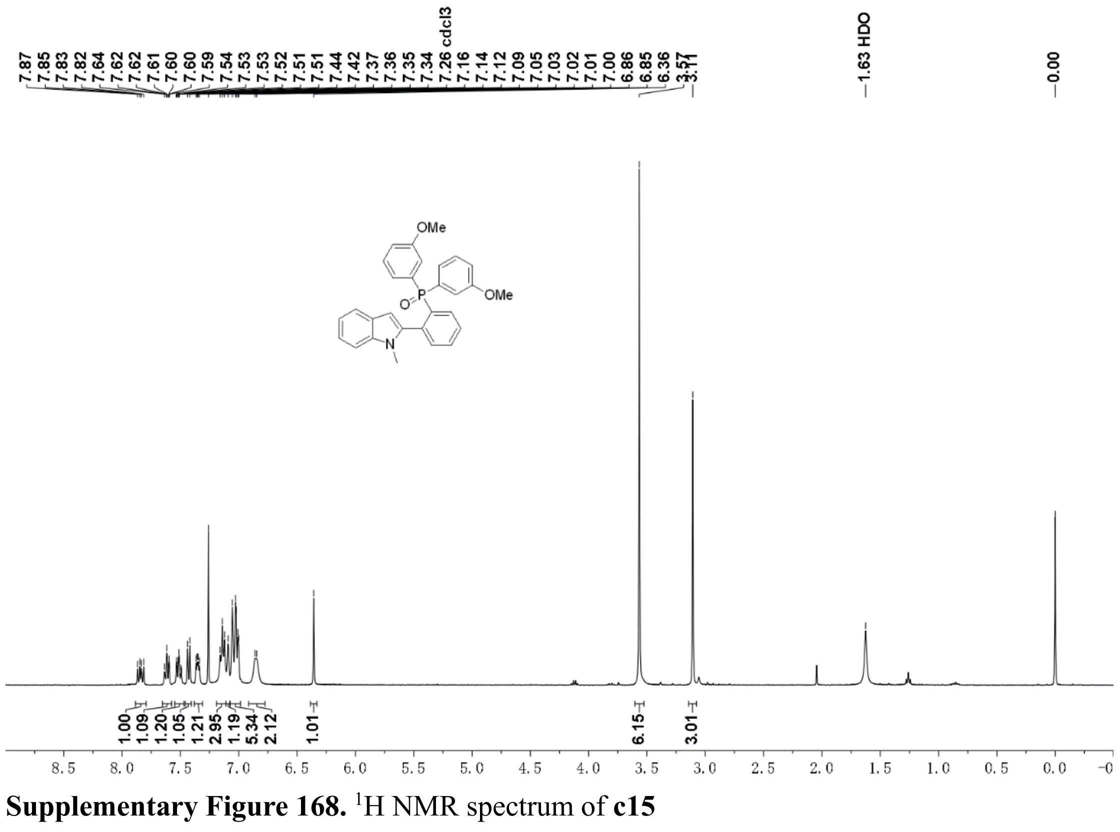

Supplementary Figure 168. ^{1}H NMR spectrum of **c15**

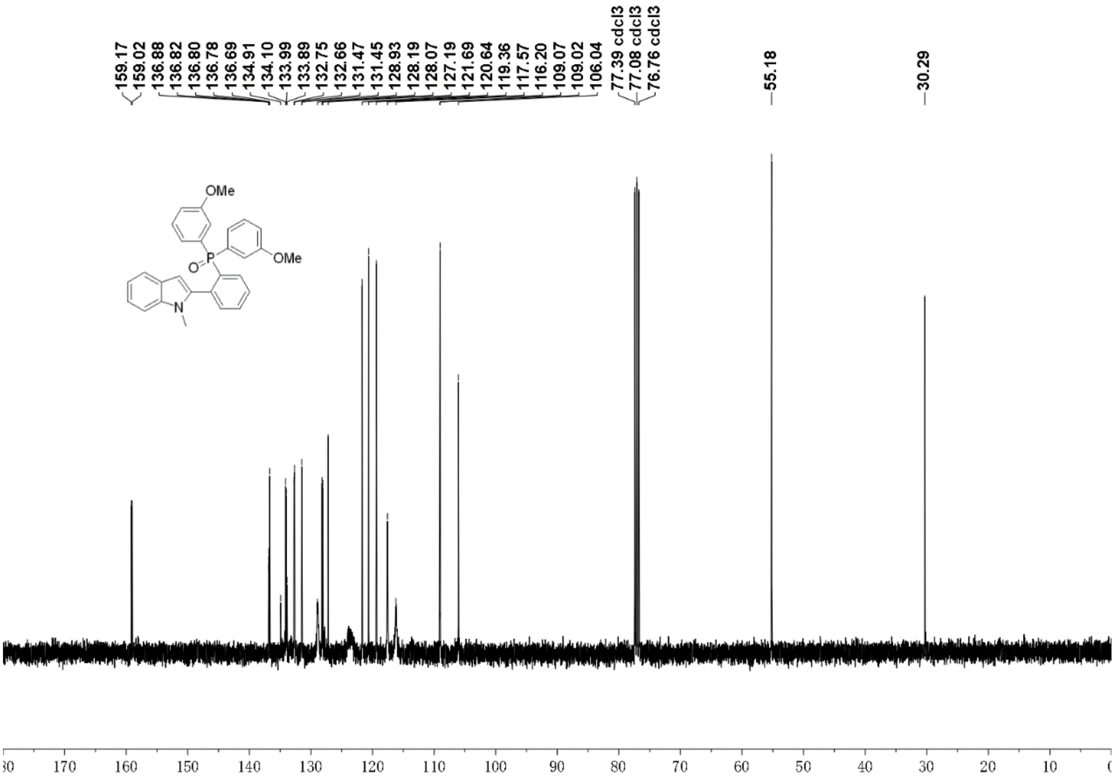

Supplementary Figure 169. ^{13}C NMR spectrum of **c15**

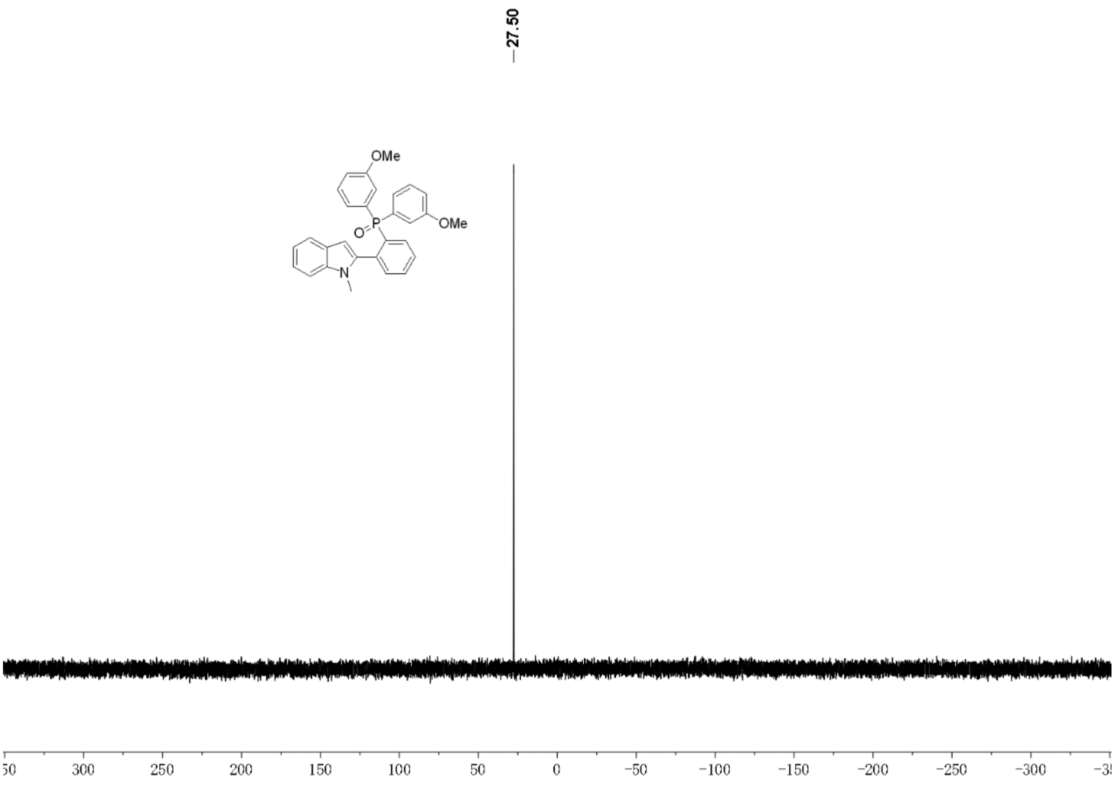

Supplementary Figure 170. ^{31}P NMR spectrum of **c15**

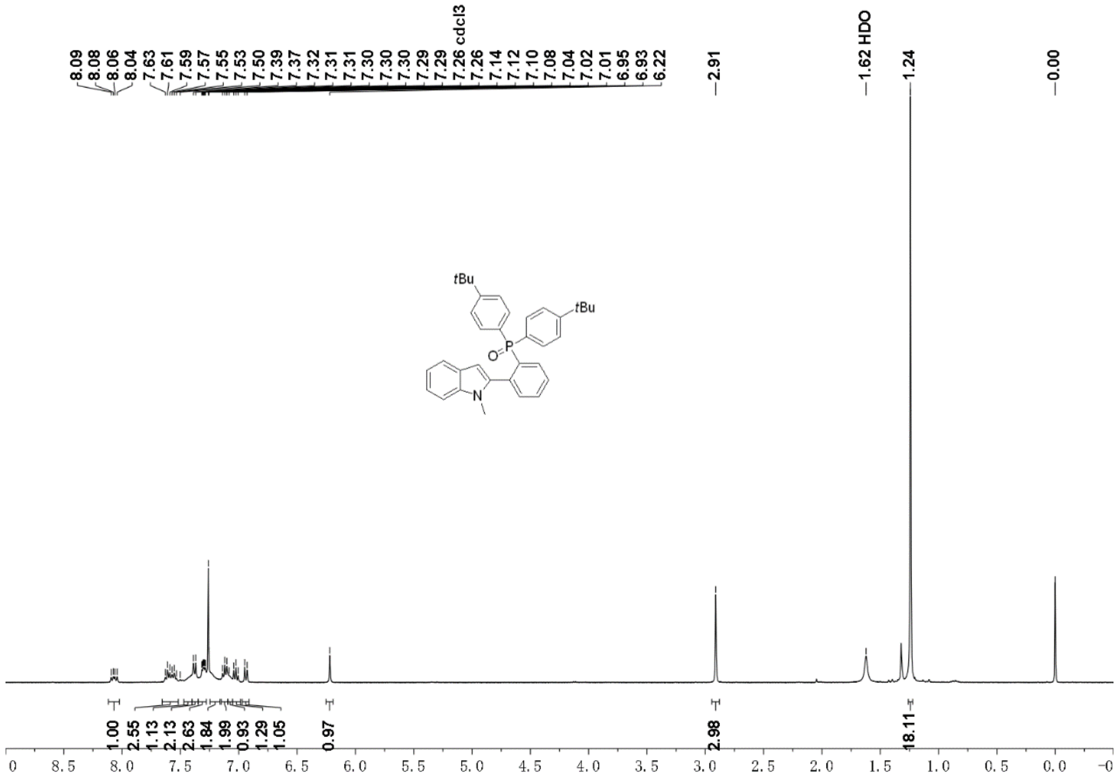

Supplementary Figure 171. ^{1}H NMR spectrum of **c16**

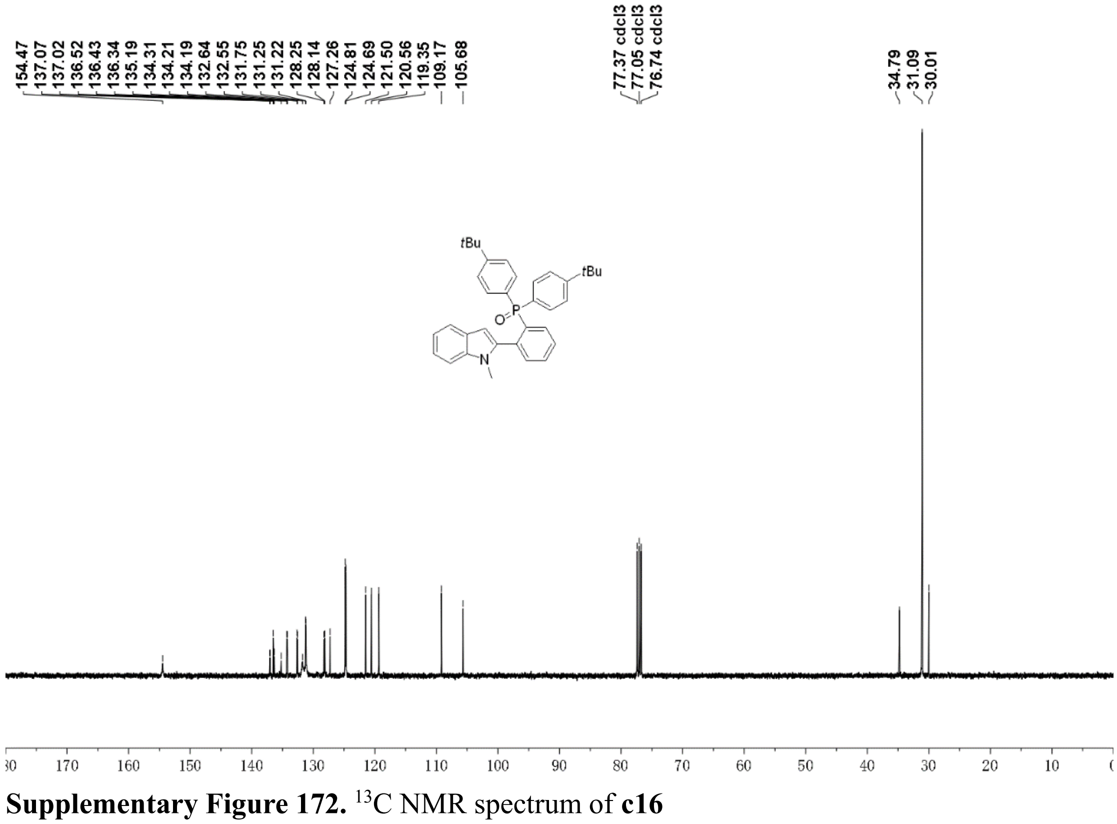

Supplementary Figure 172. ^{13}C NMR spectrum of **c16**

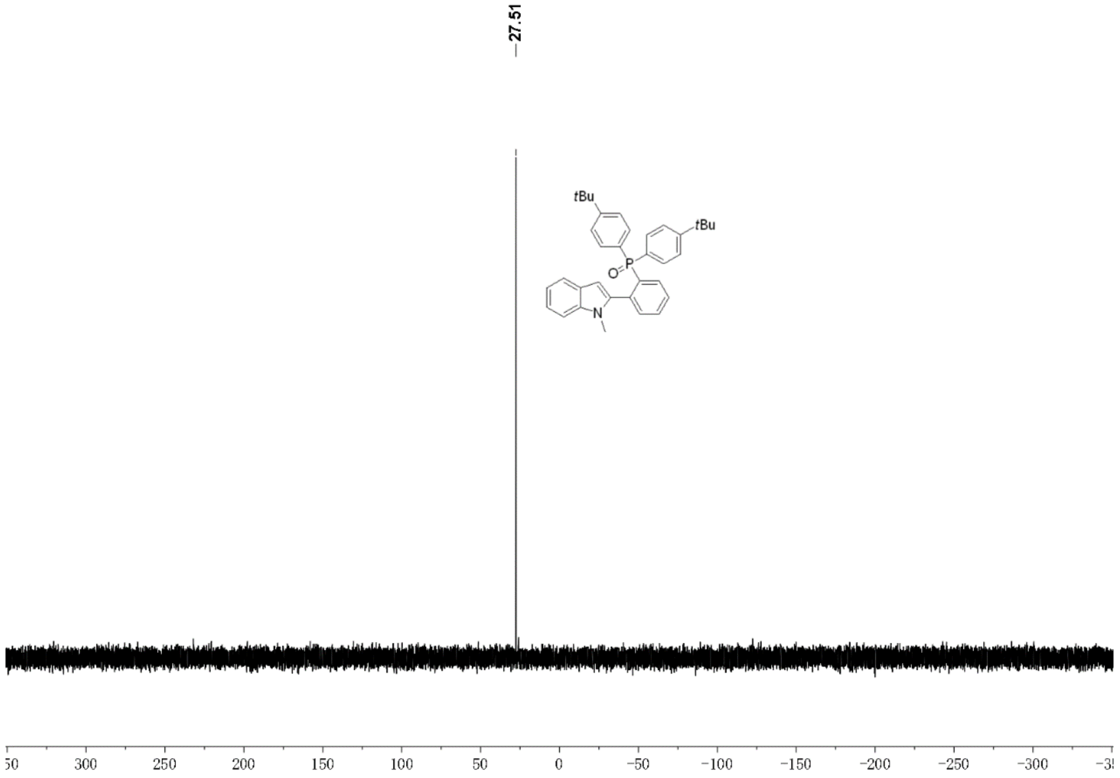

Supplementary Figure 173. ^{31}P NMR spectrum of **c16**

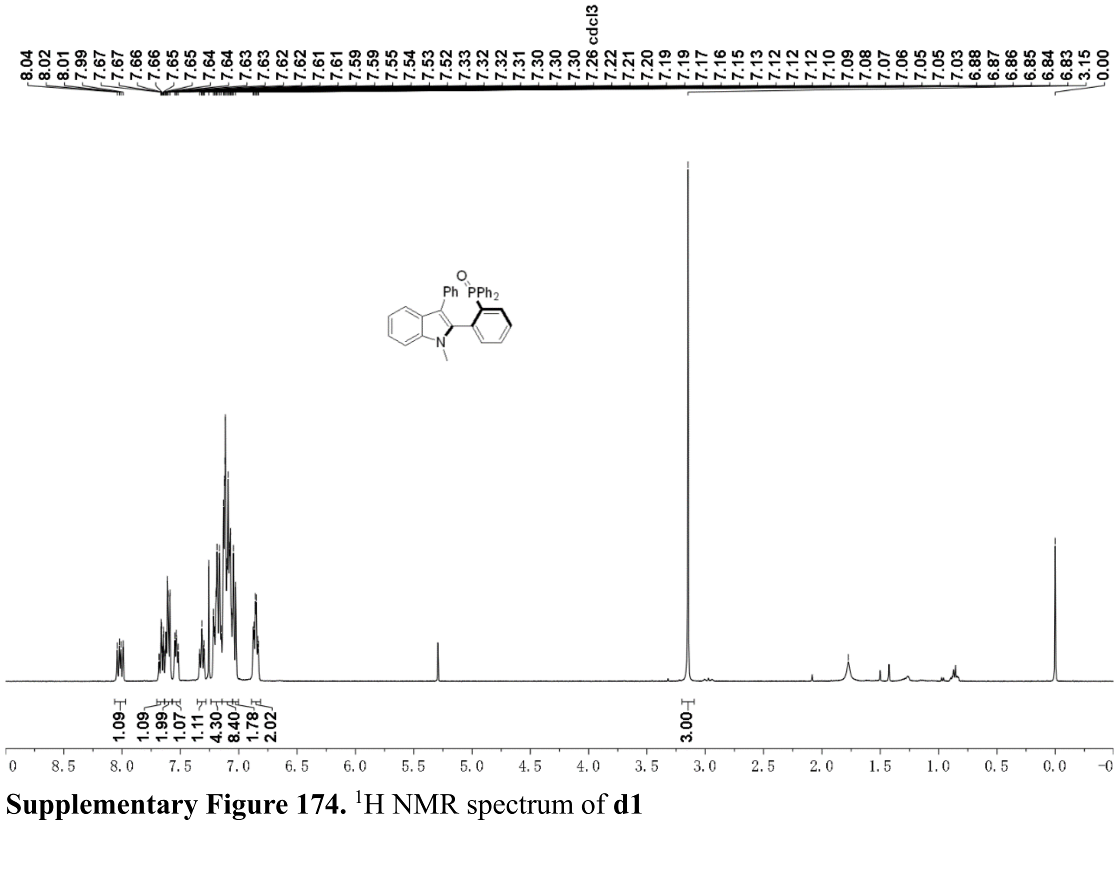

Supplementary Figure 174. ^{1}H NMR spectrum of **d1**

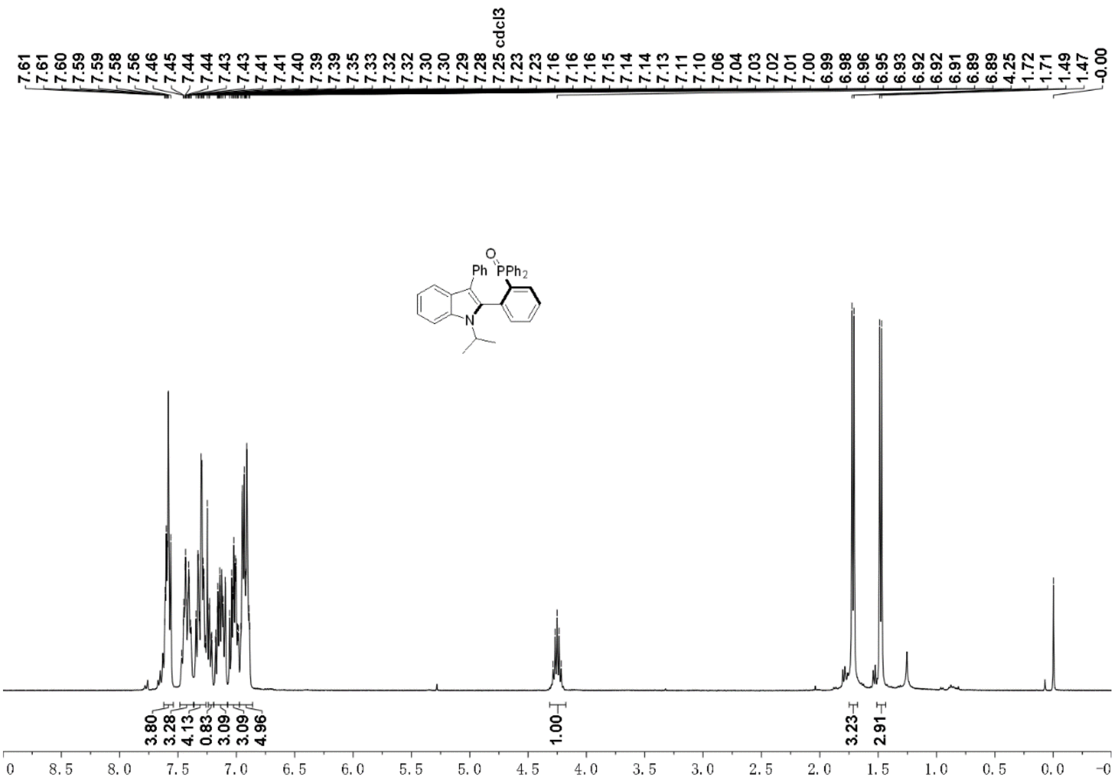

Supplementary Figure 175. ^{1}H NMR spectrum of **d2**

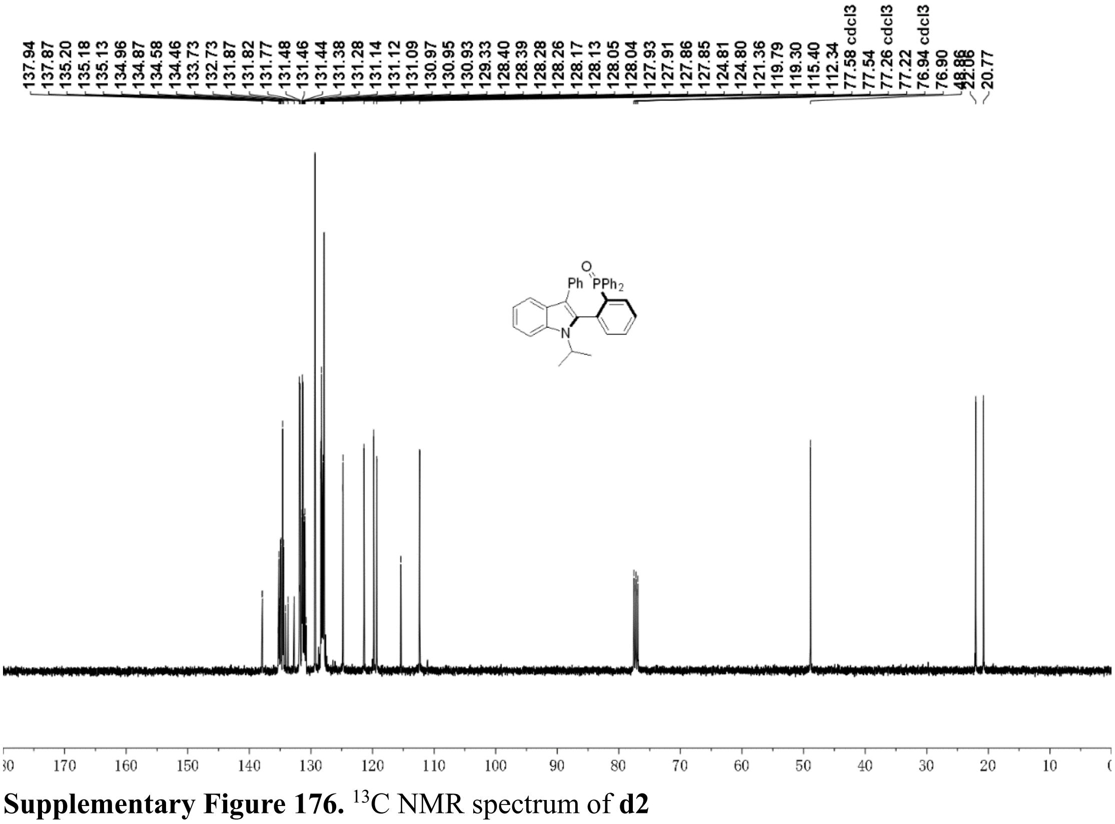

Supplementary Figure 176. ^{13}C NMR spectrum of **d2**

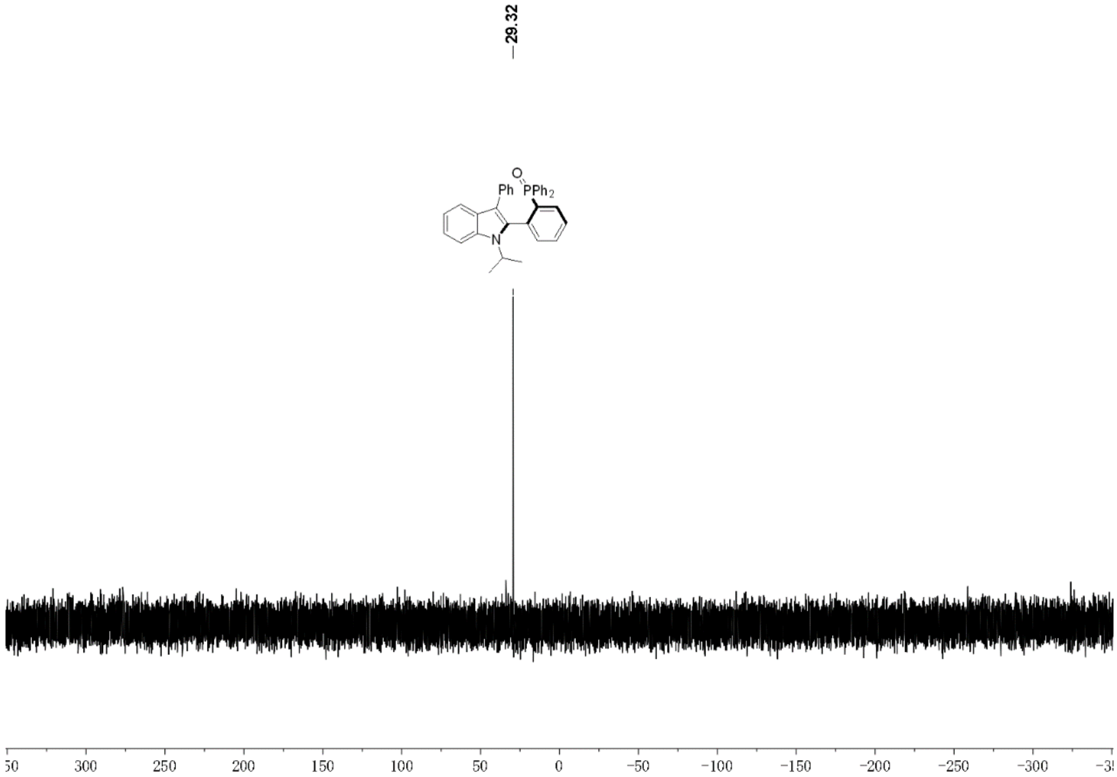

Supplementary Figure 177. ^{31}P NMR spectrum of **d2**

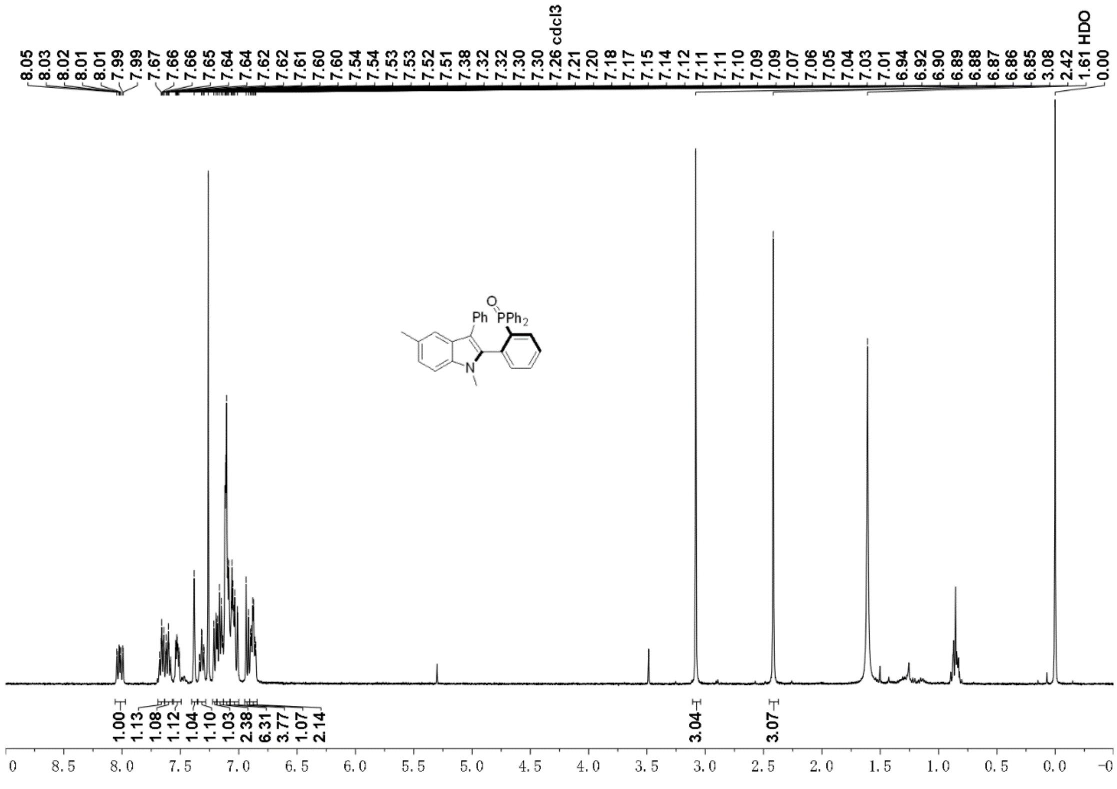

Supplementary Figure 178. ^{1}H NMR spectrum of **d3**

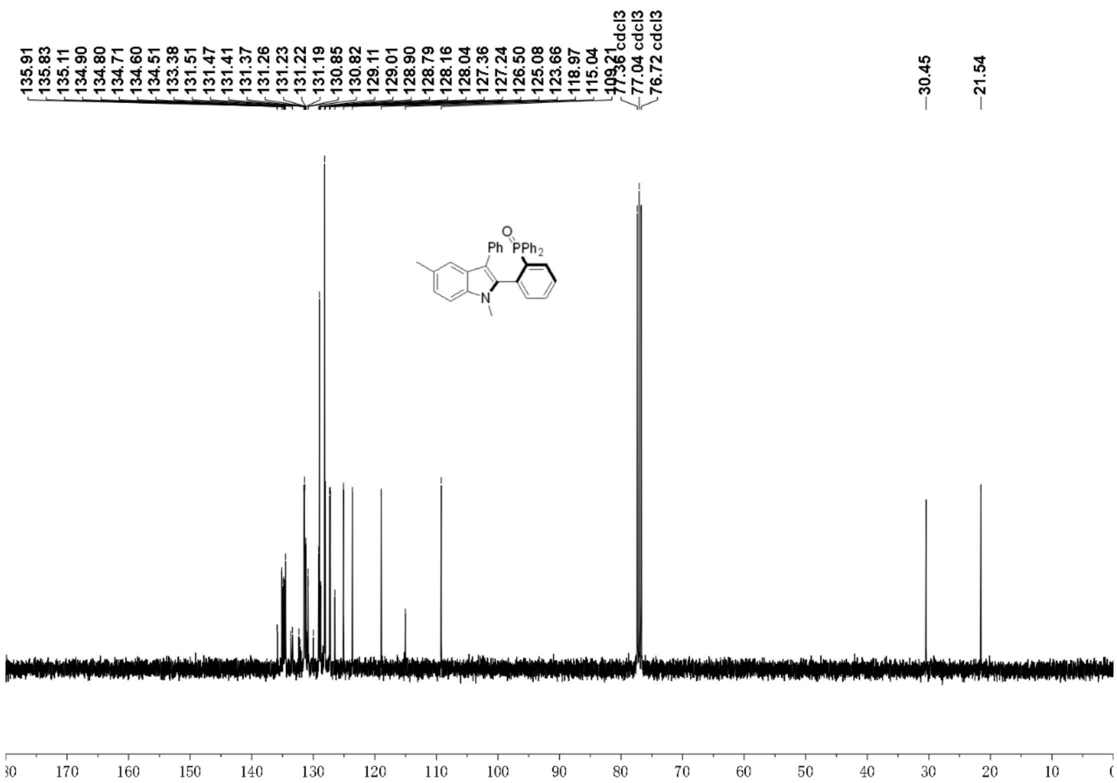

Supplementary Figure 179. ^{13}C NMR spectrum of **d3**

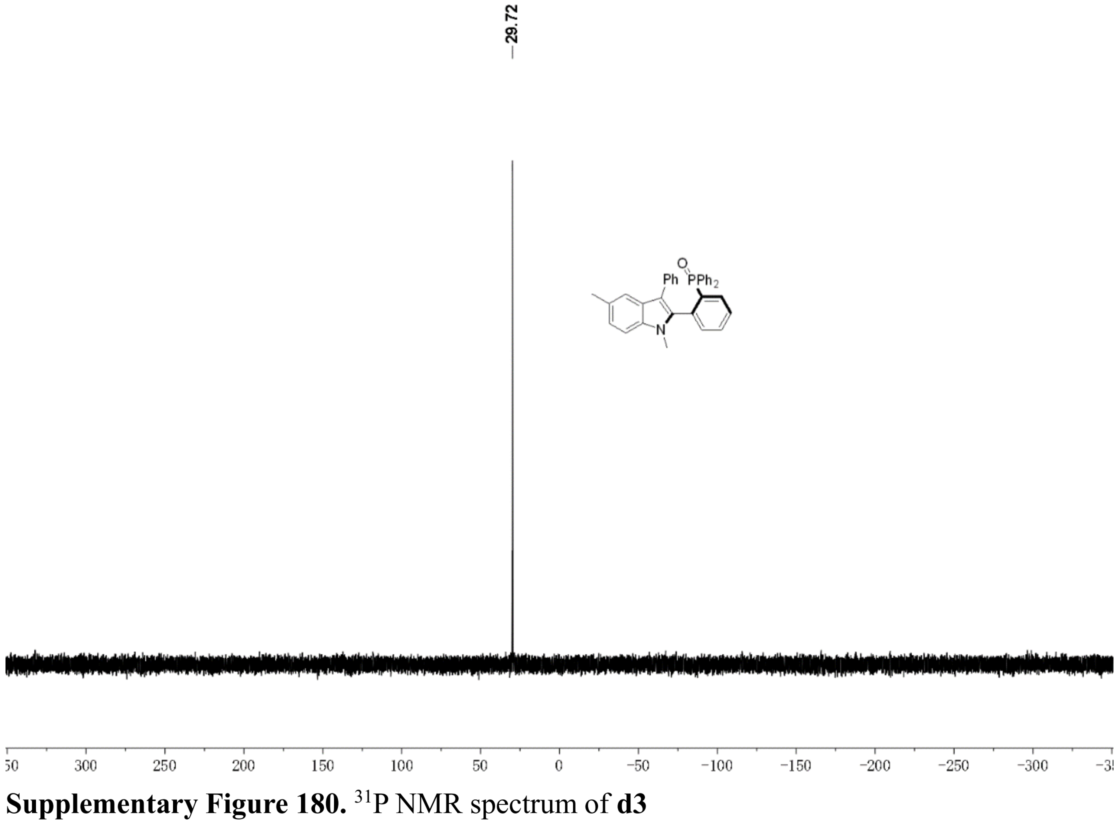

Supplementary Figure 180. ^{31}P NMR spectrum of **d3**

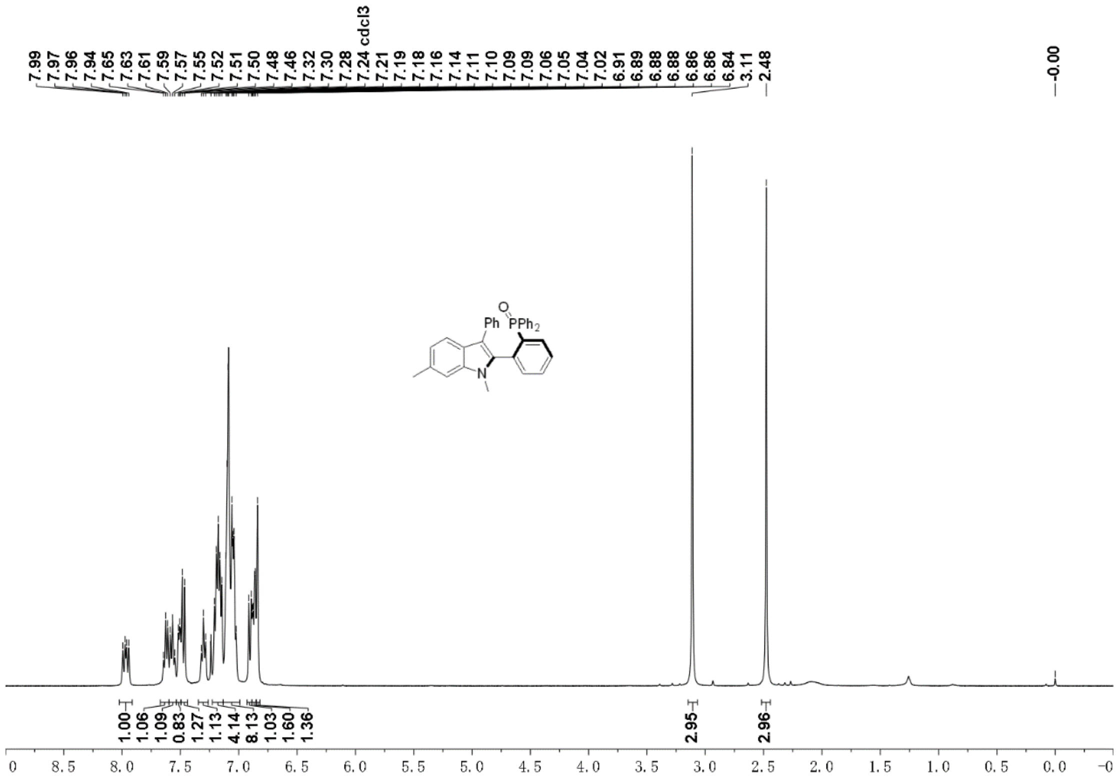

Supplementary Figure 181. ^{1}H NMR spectrum of **d4**

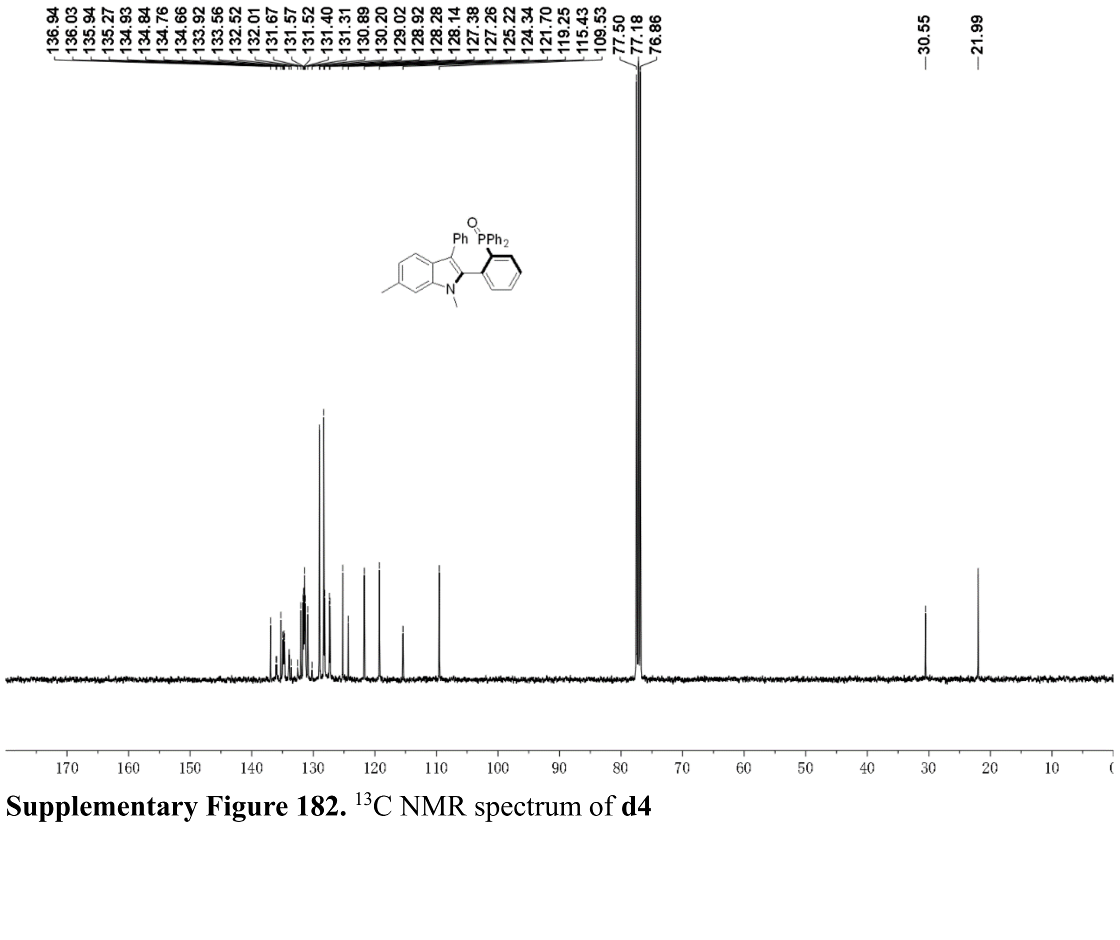

Supplementary Figure 182. ^{13}C NMR spectrum of **d4**

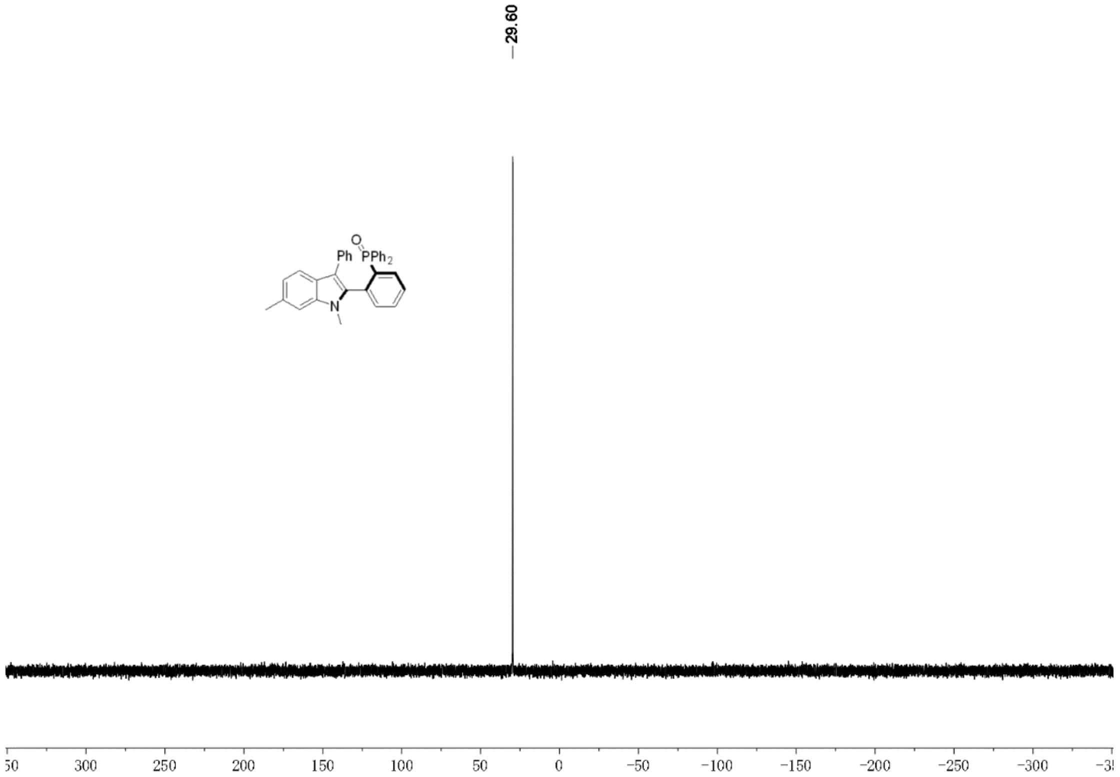

Supplementary Figure 183. ^{31}P NMR spectrum of **d4**

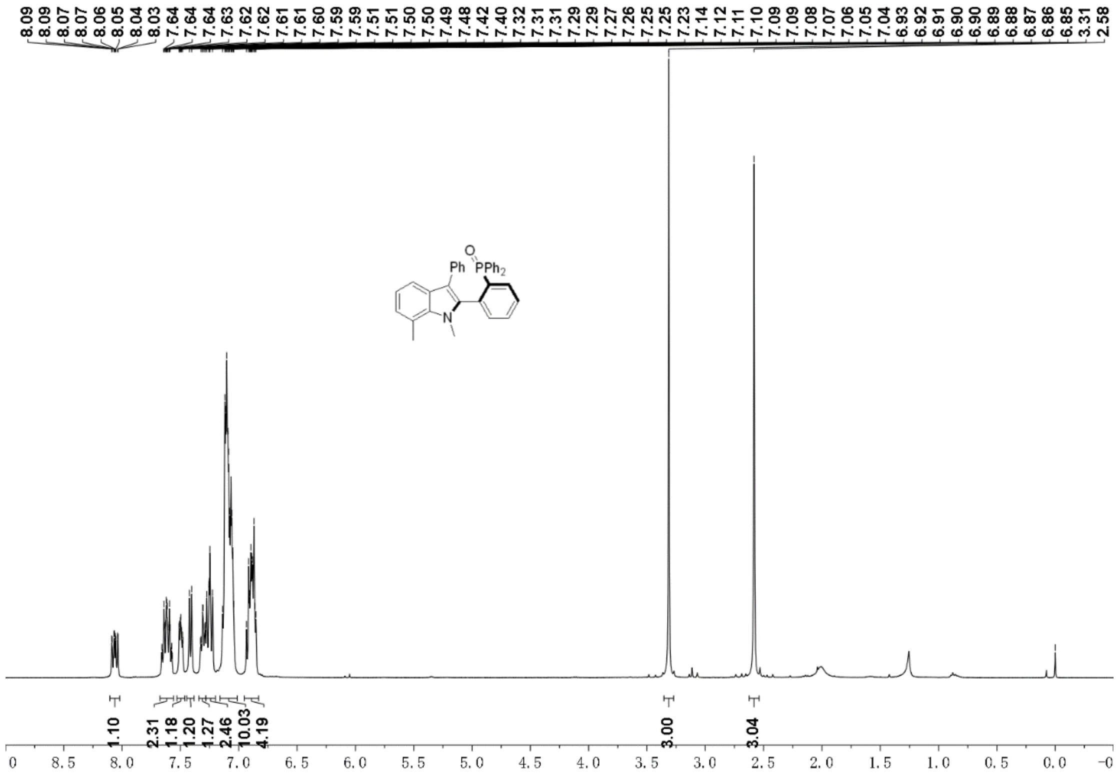

Supplementary Figure 184. ^{1}H NMR spectrum of **d5**

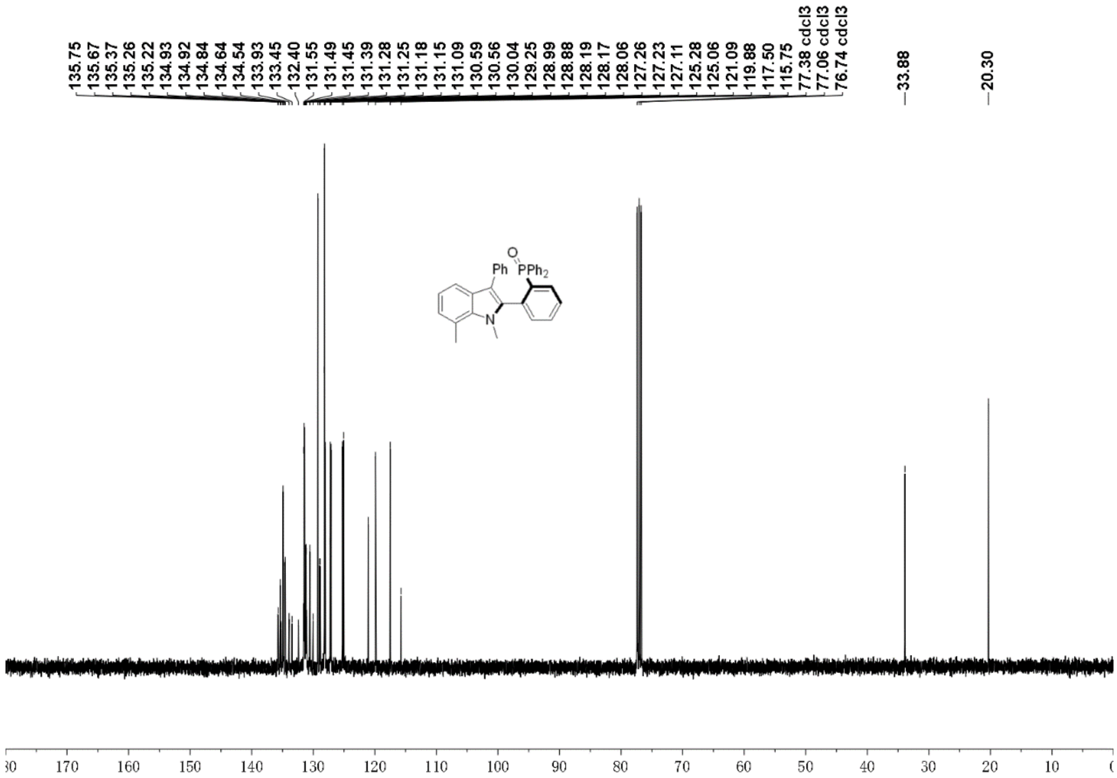

Supplementary Figure 185. ^{13}C NMR spectrum of **d5**

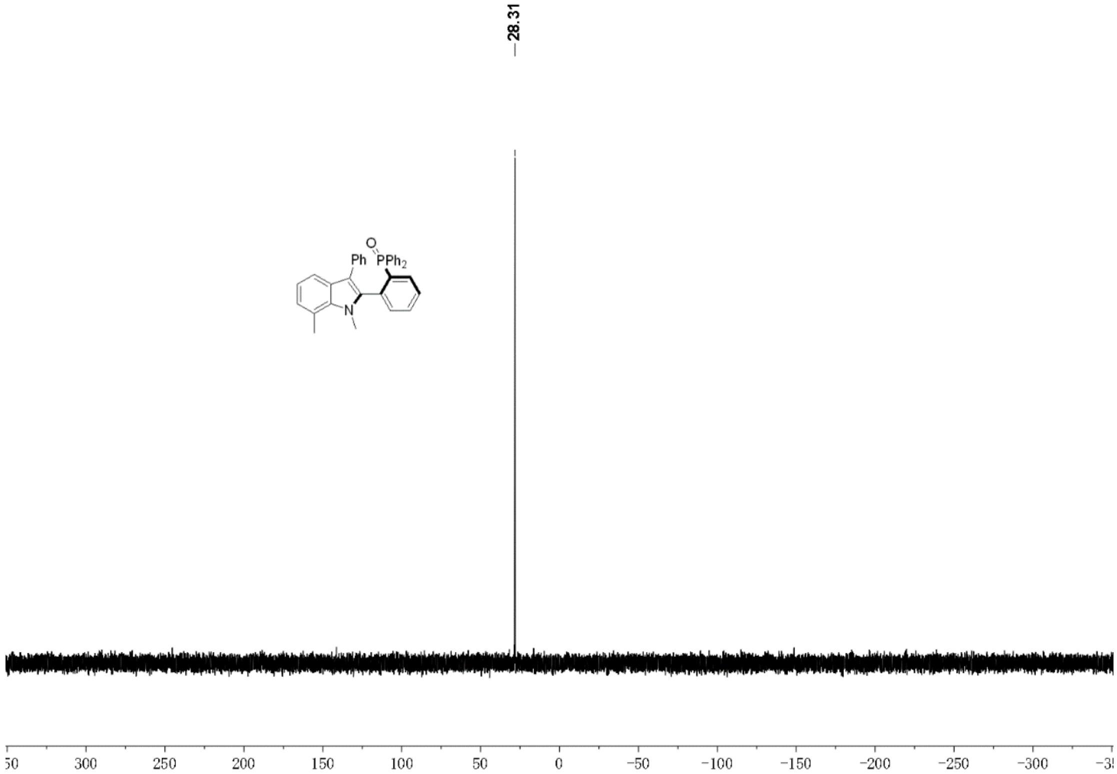

Supplementary Figure 186. ^{31}P NMR spectrum of **d5**

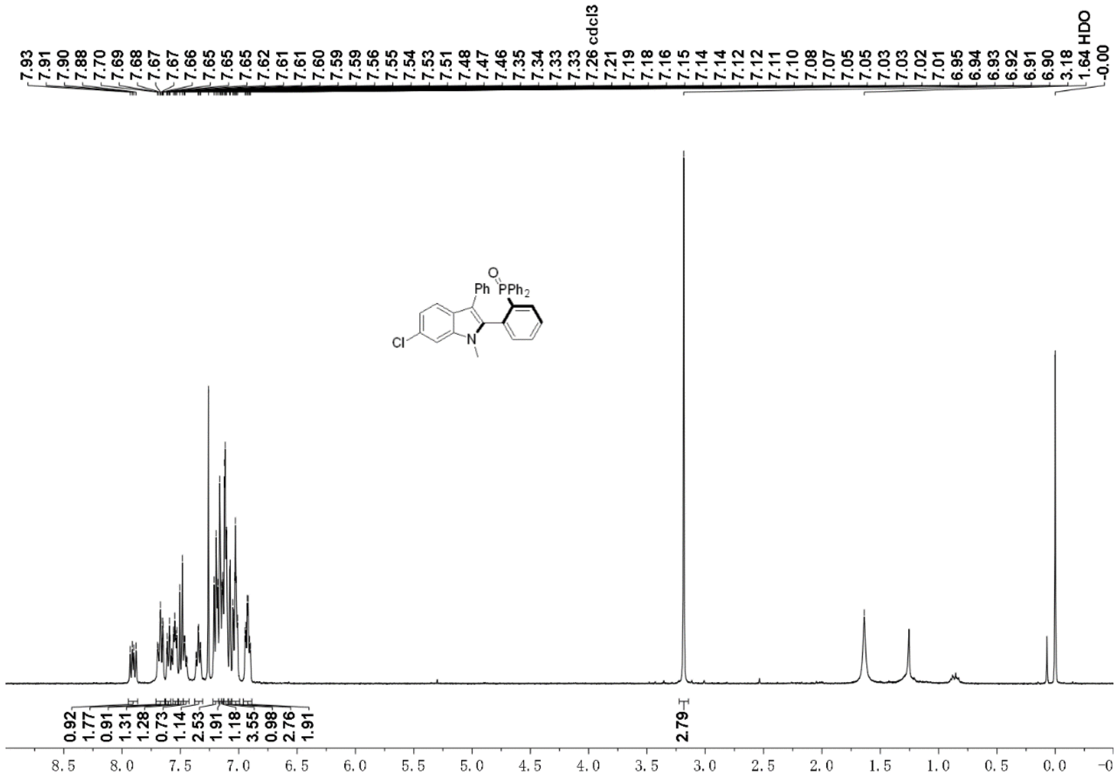

Supplementary Figure 187. ^{1}H NMR spectrum of **d6**

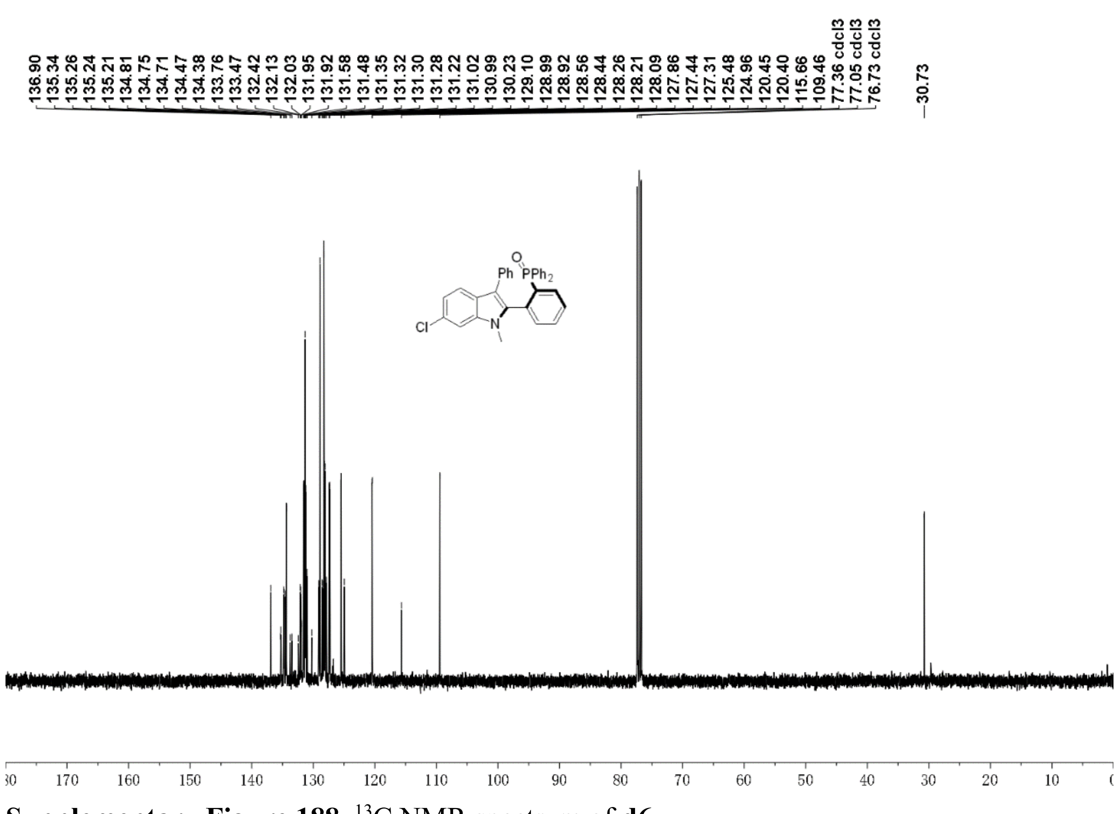

Supplementary Figure 188. ^{13}C NMR spectrum of **d6**

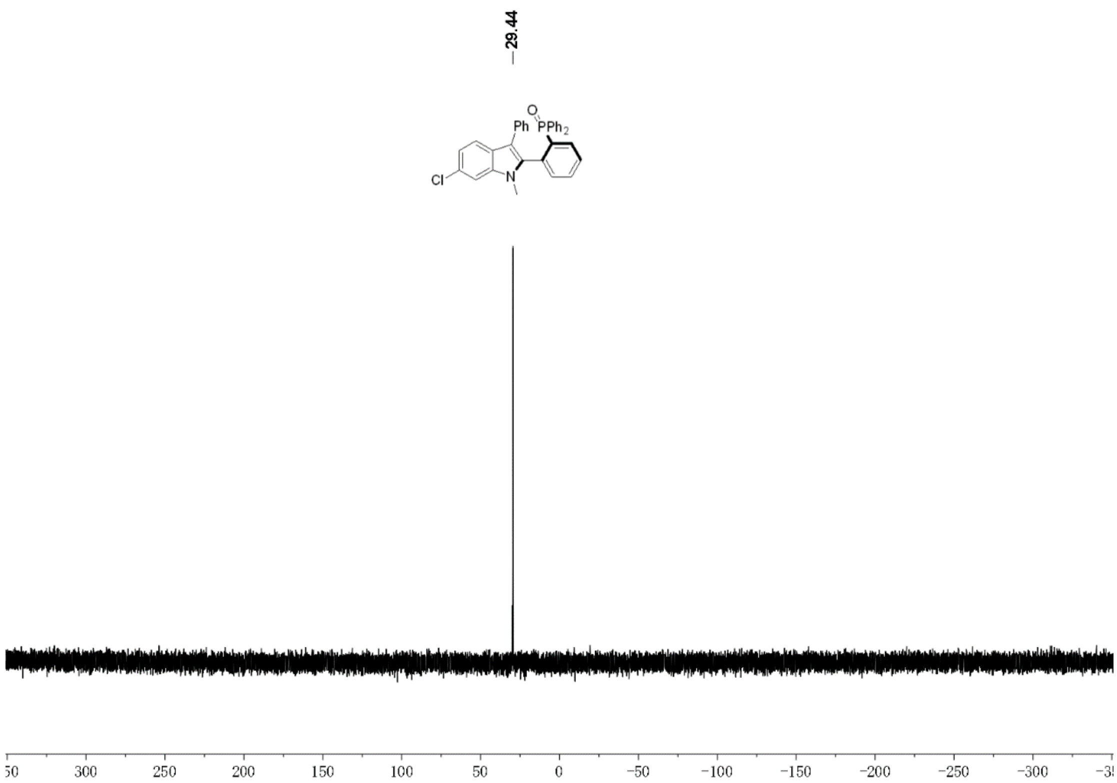

Supplementary Figure 189. ^{31}P NMR spectrum of **d6**

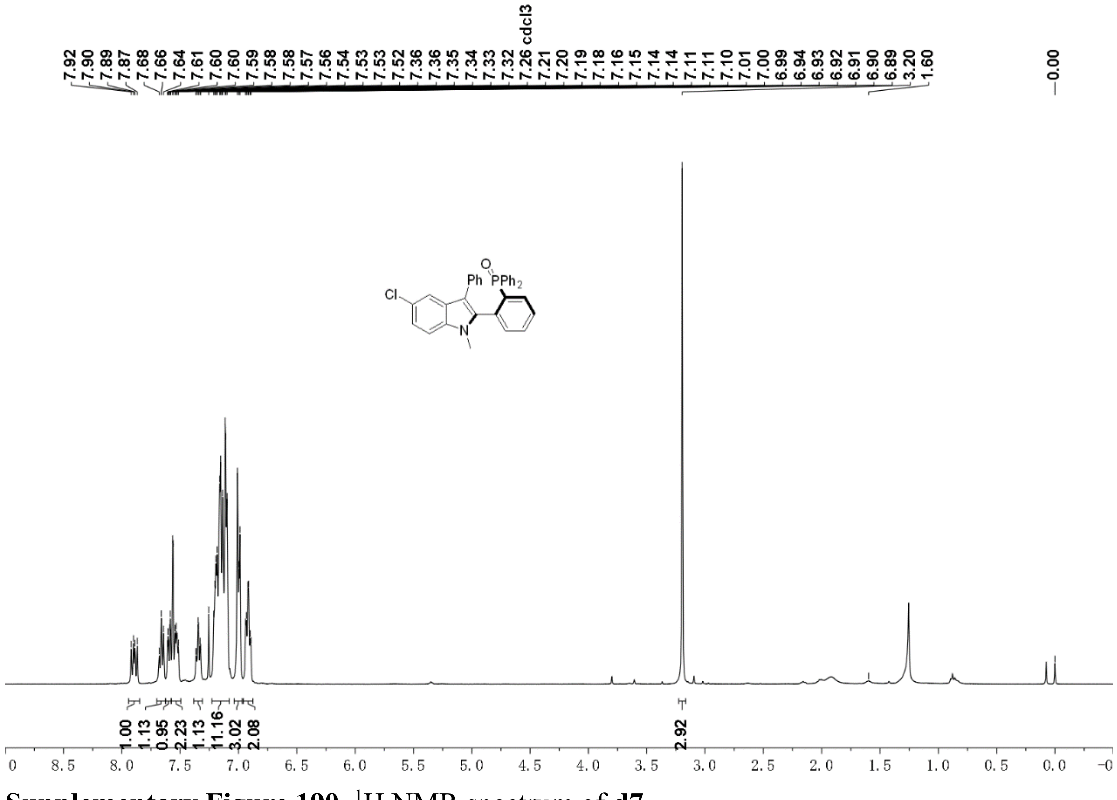

Supplementary Figure 190. ^{1}H NMR spectrum of **d7**

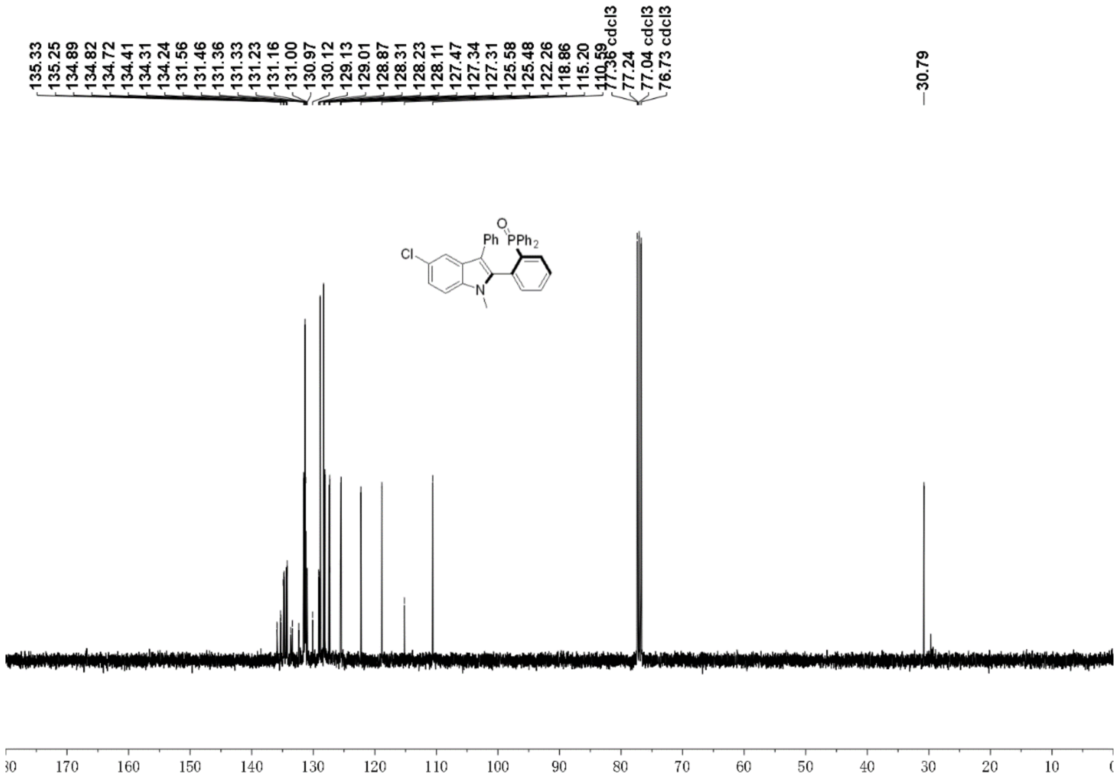

Supplementary Figure 191. ^{13}C NMR spectrum of **d7**

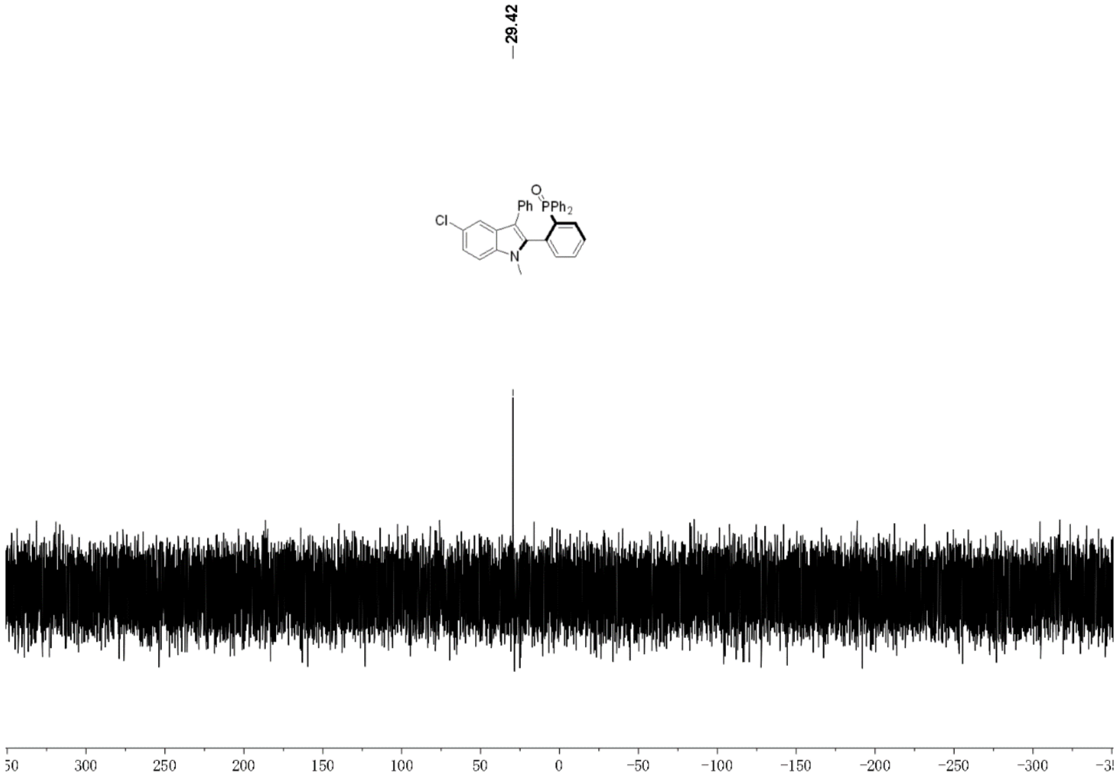

Supplementary Figure 192. ^{31}P NMR spectrum of **d7**

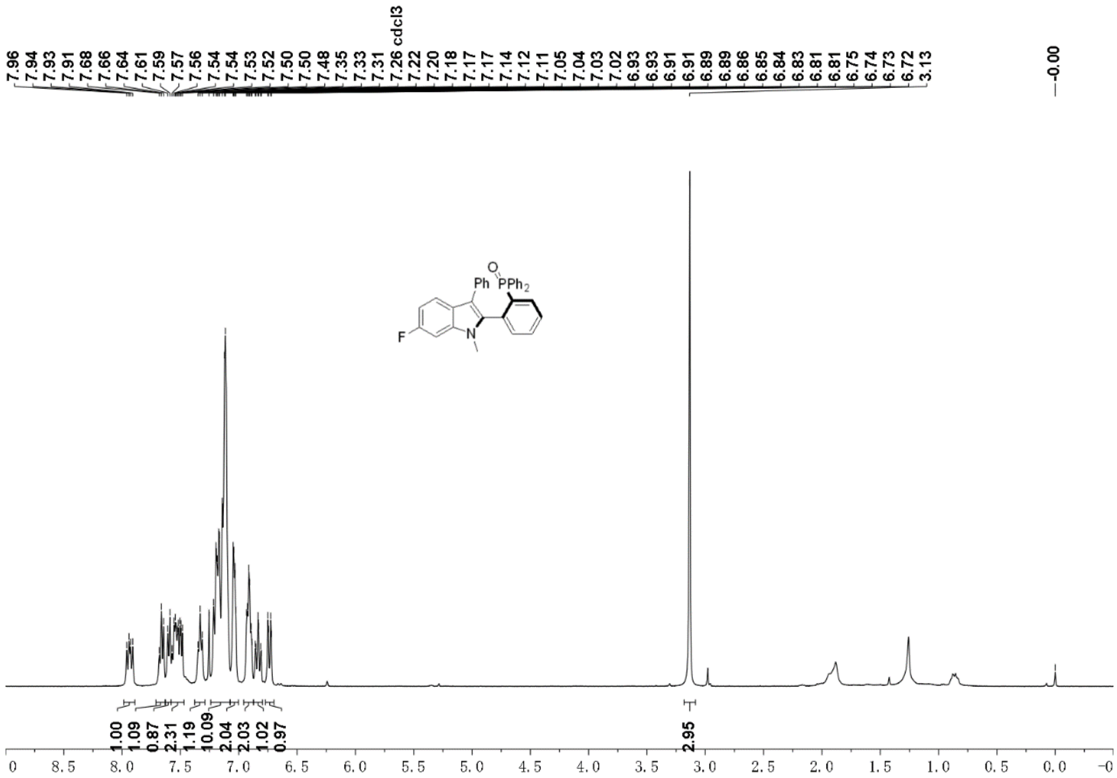

Supplementary Figure 193. ^{1}H NMR spectrum of **d8**

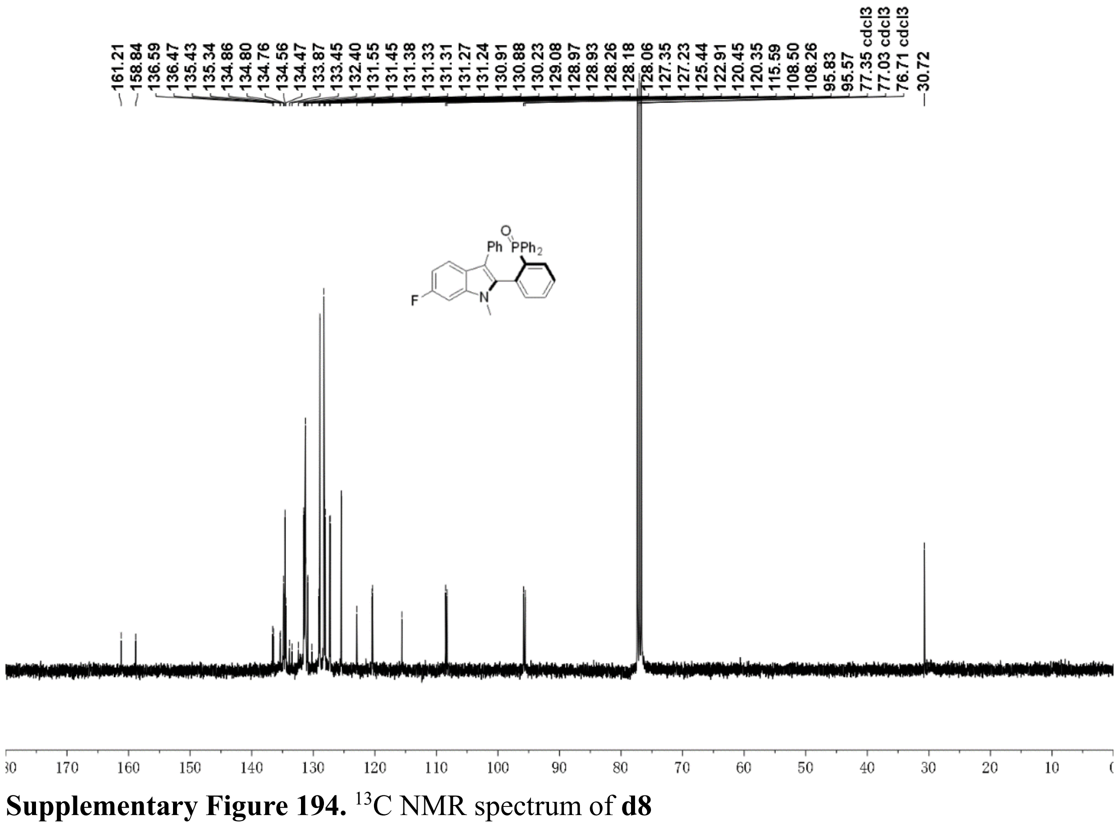

Supplementary Figure 194. ^{13}C NMR spectrum of **d8**

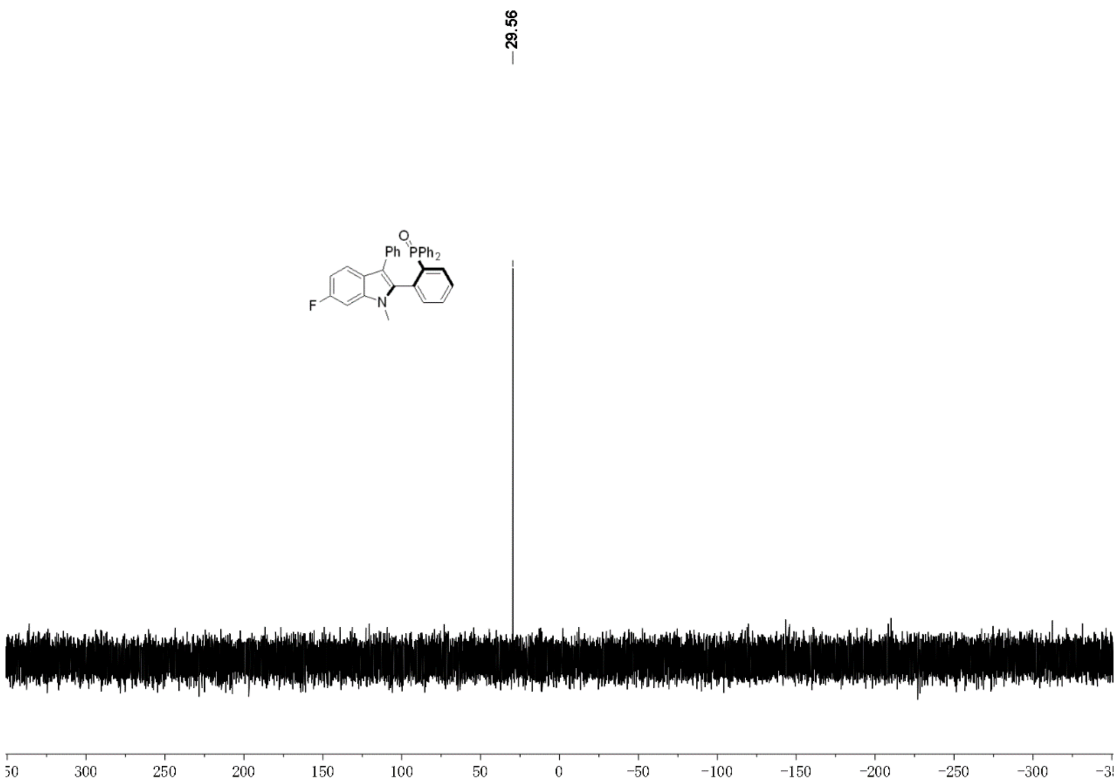

Supplementary Figure 195. ^{31}P NMR spectrum of **d8**

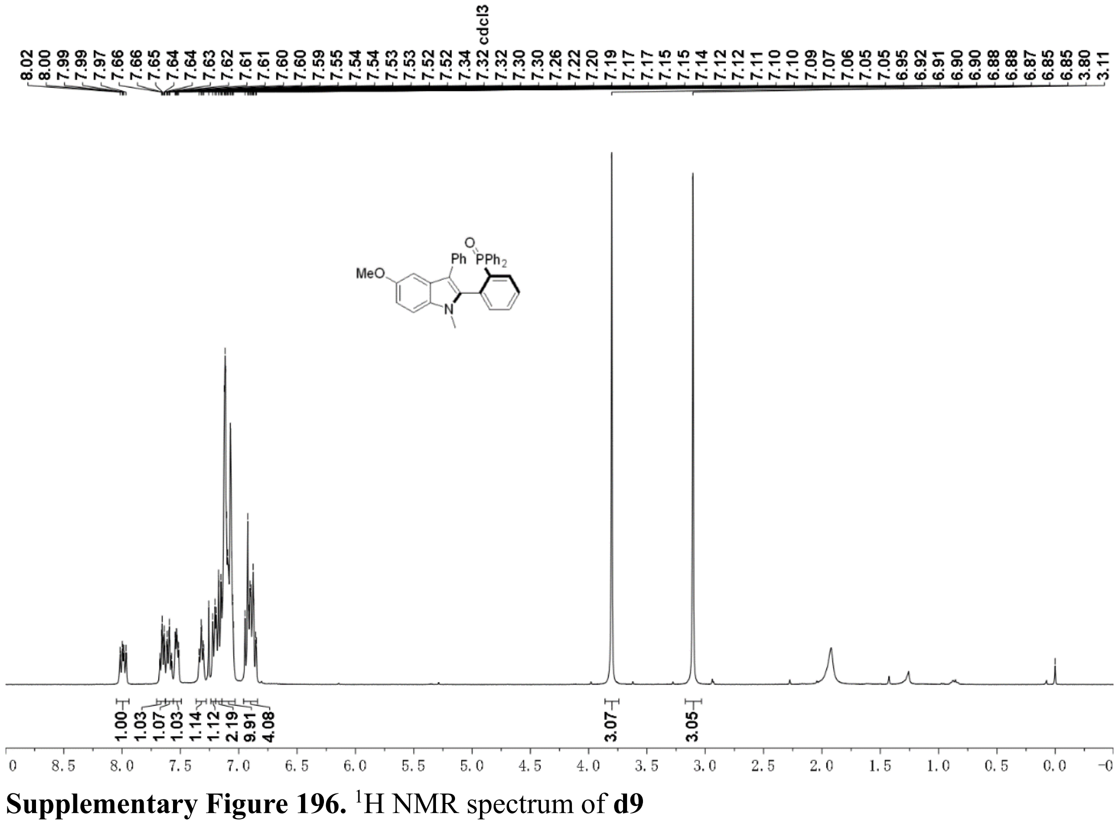

Supplementary Figure 196. ^{1}H NMR spectrum of **d9**

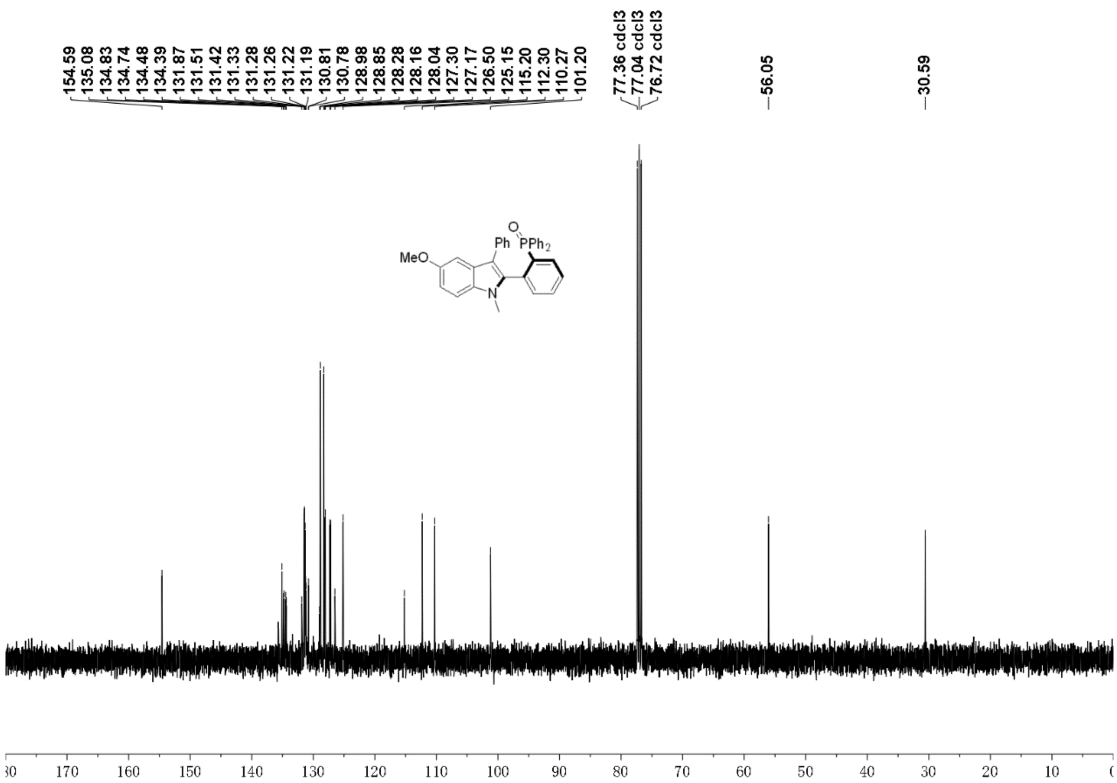

Supplementary Figure 197. ^{13}C NMR spectrum of **d9**

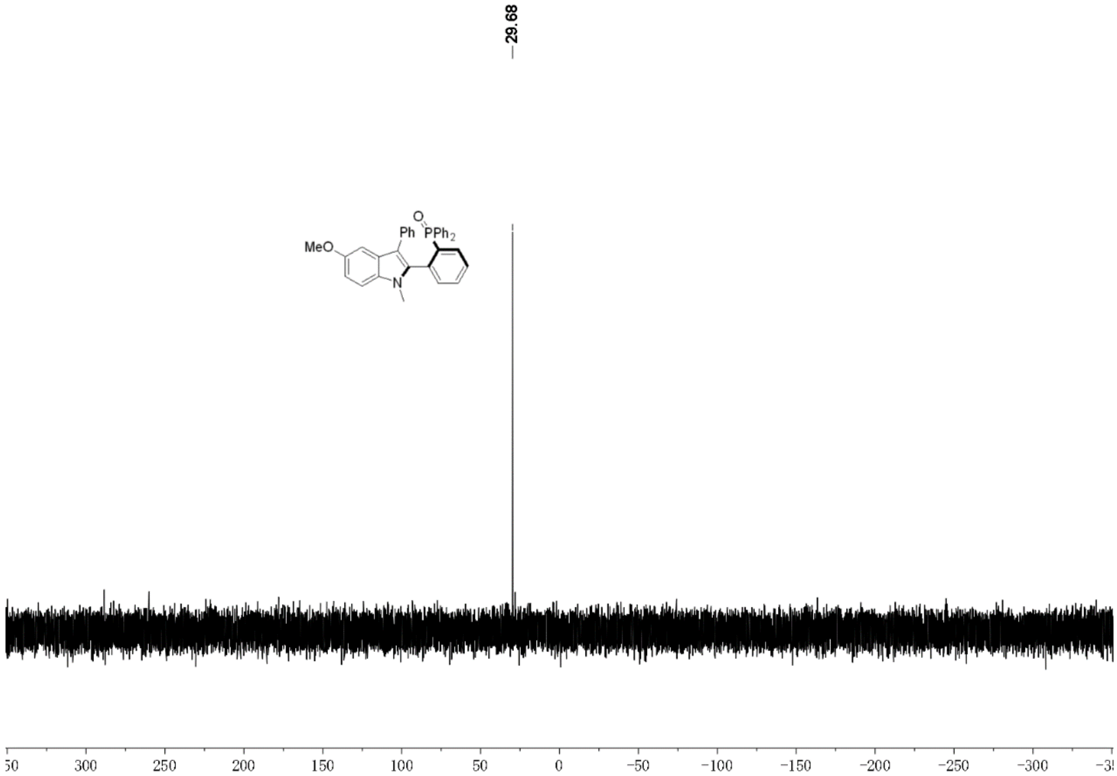

Supplementary Figure 198. ^{31}P NMR spectrum of **d9**

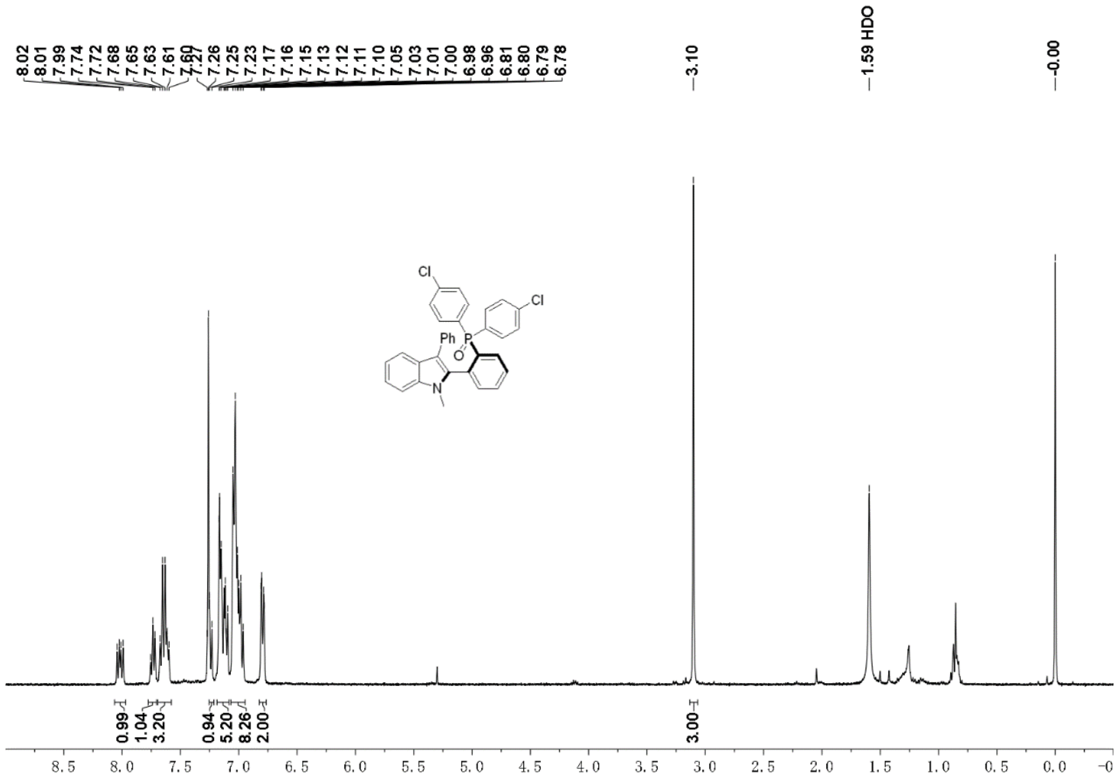

Supplementary Figure 199. ^{1}H NMR spectrum of **d10**

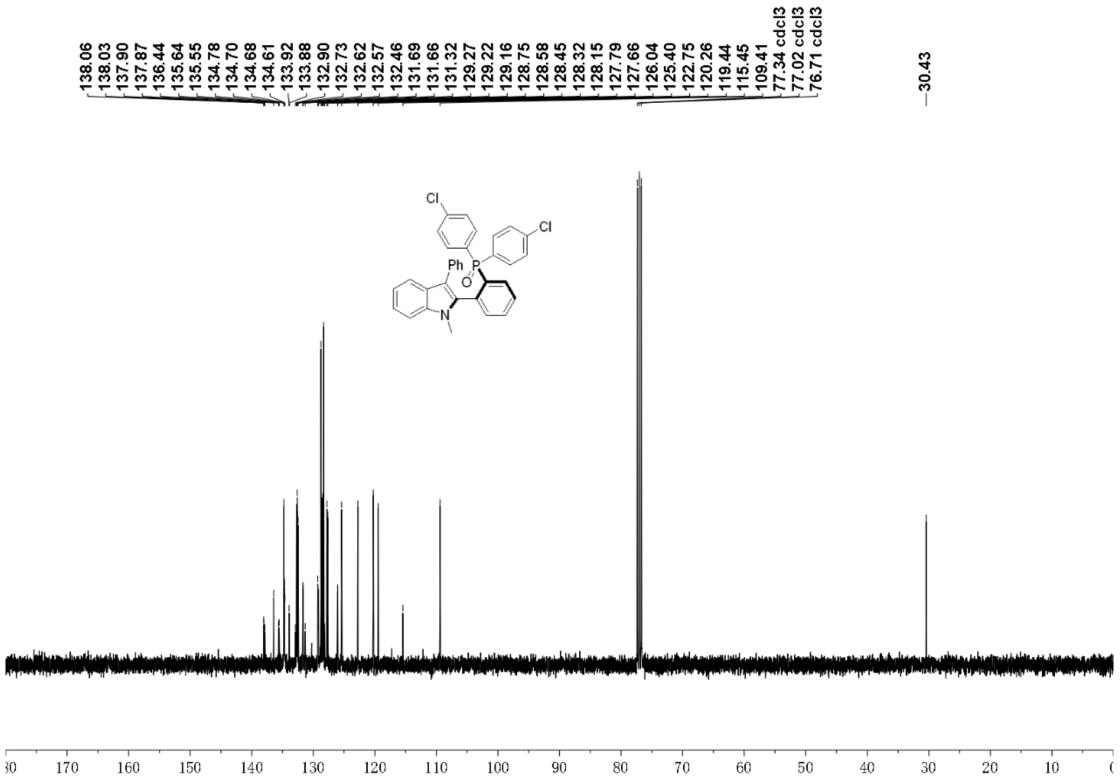

Supplementary Figure 200. ^{13}C NMR spectrum of **d10**

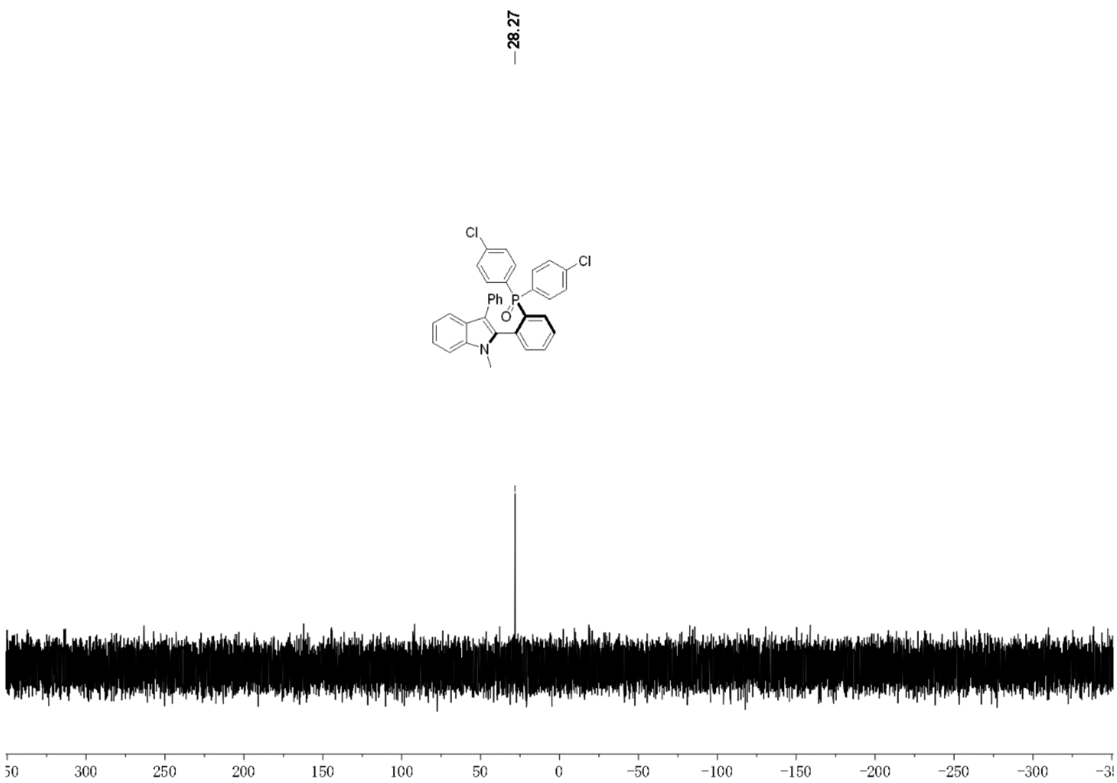

Supplementary Figure 201. ^{31}P NMR spectrum of **d10**

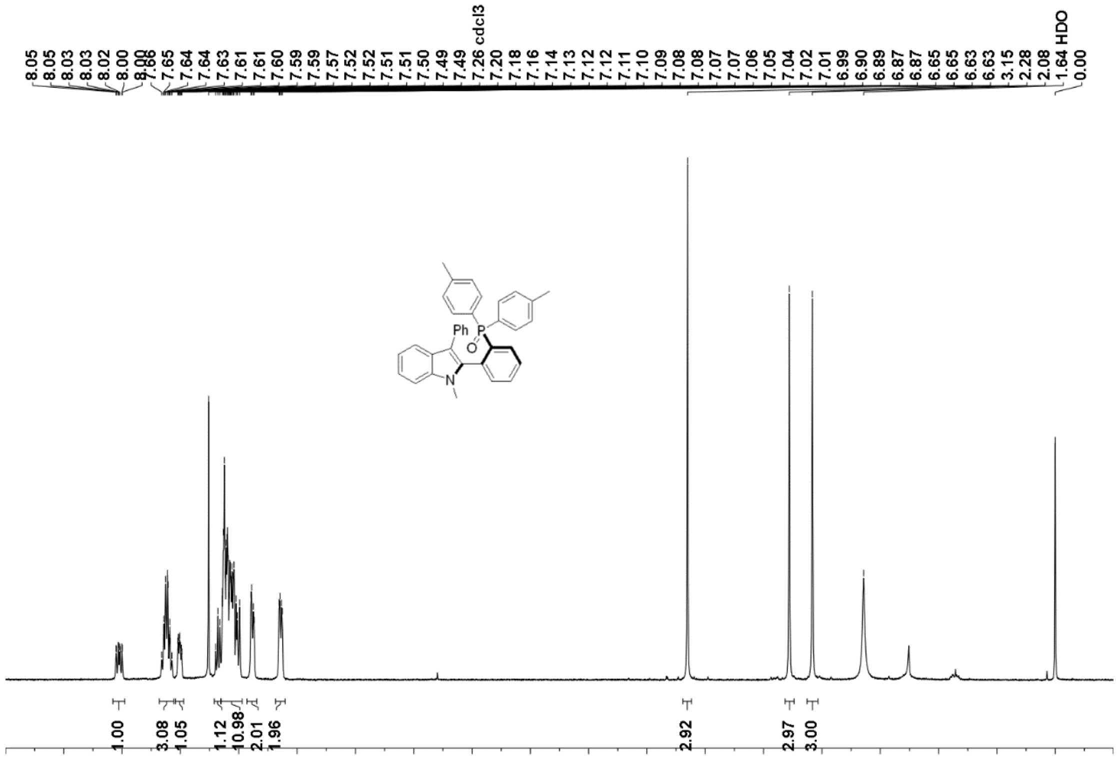

Supplementary Figure 202. ^{1}H NMR spectrum of **d11**

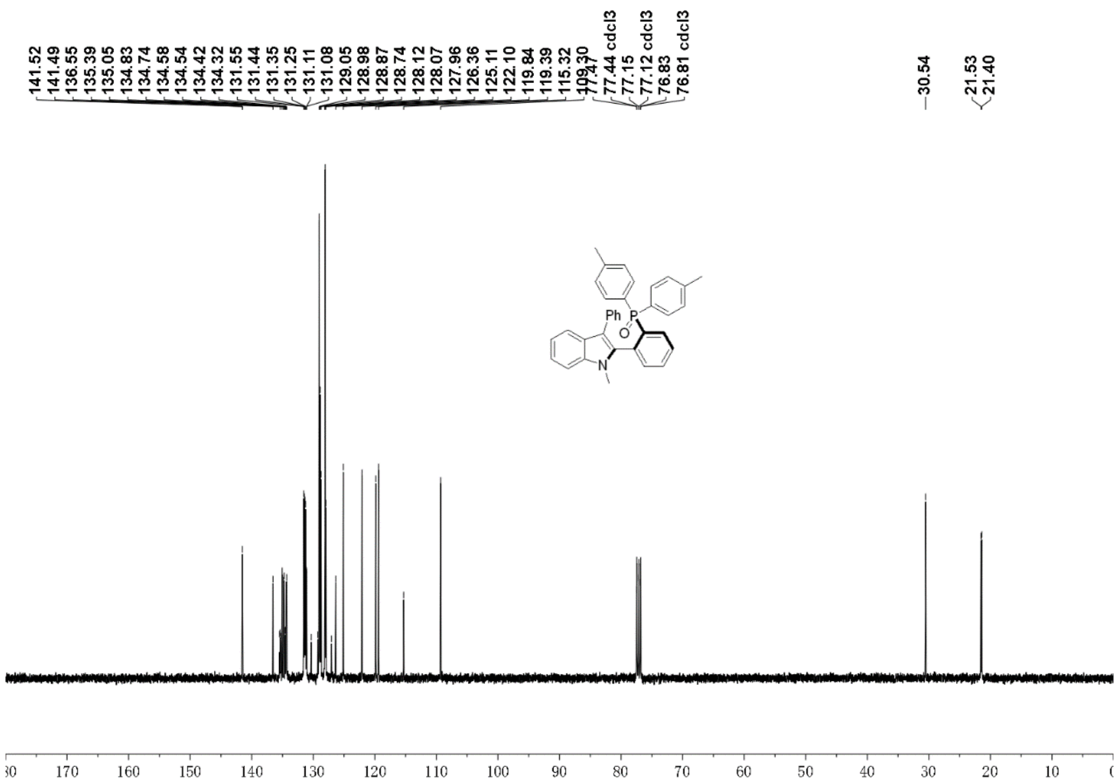

Supplementary Figure 203. ^{13}C NMR spectrum of **d11**

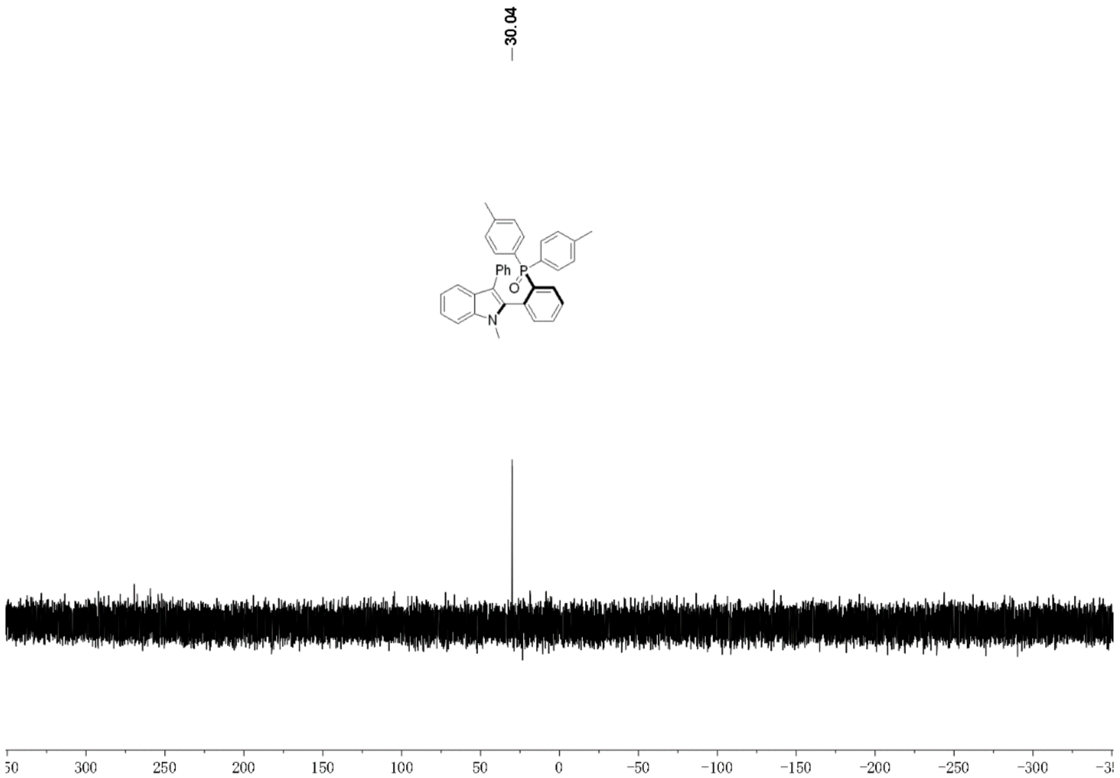

Supplementary Figure 204. ^{31}P NMR spectrum of **d11**

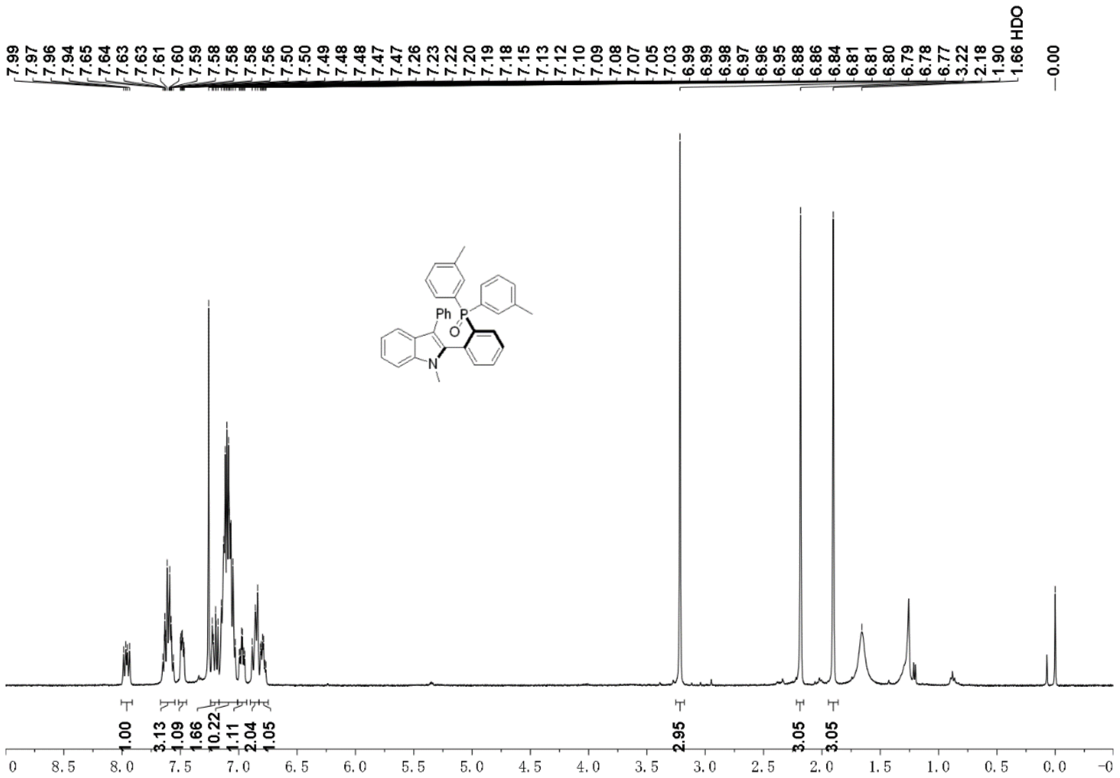

Supplementary Figure 205. ^{1}H NMR spectrum of **d12**

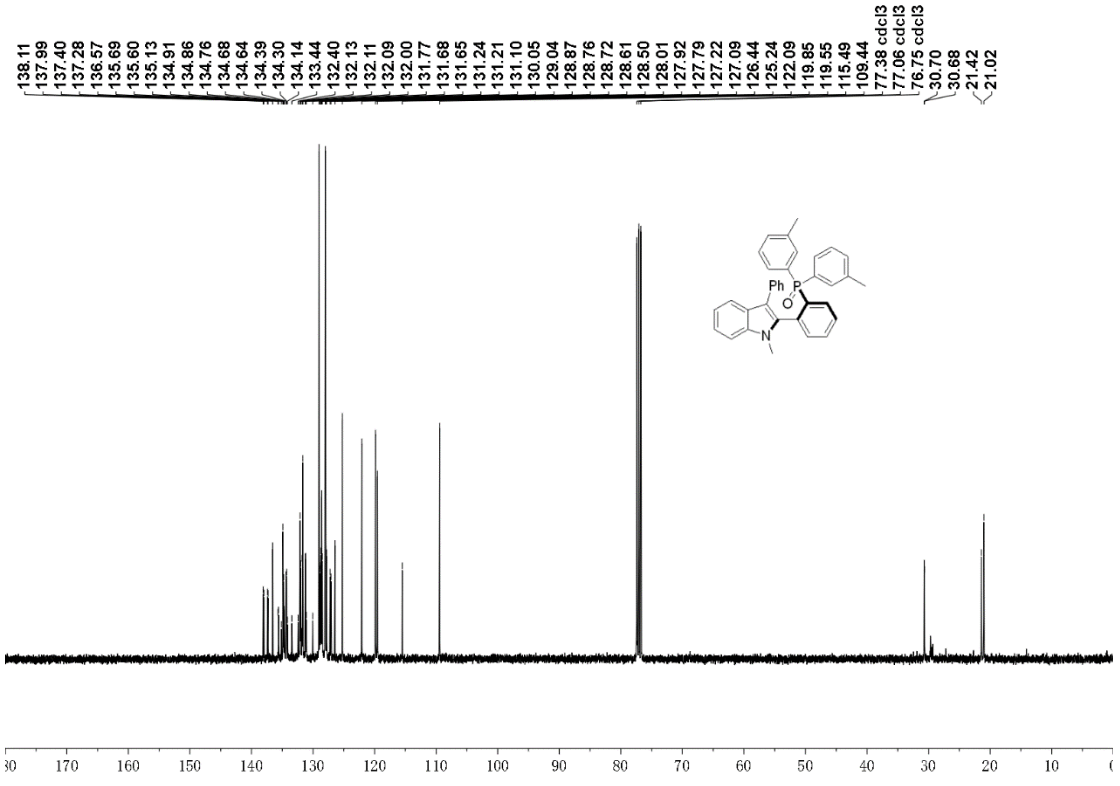

Supplementary Figure 206. ^{13}C NMR spectrum of **d12**

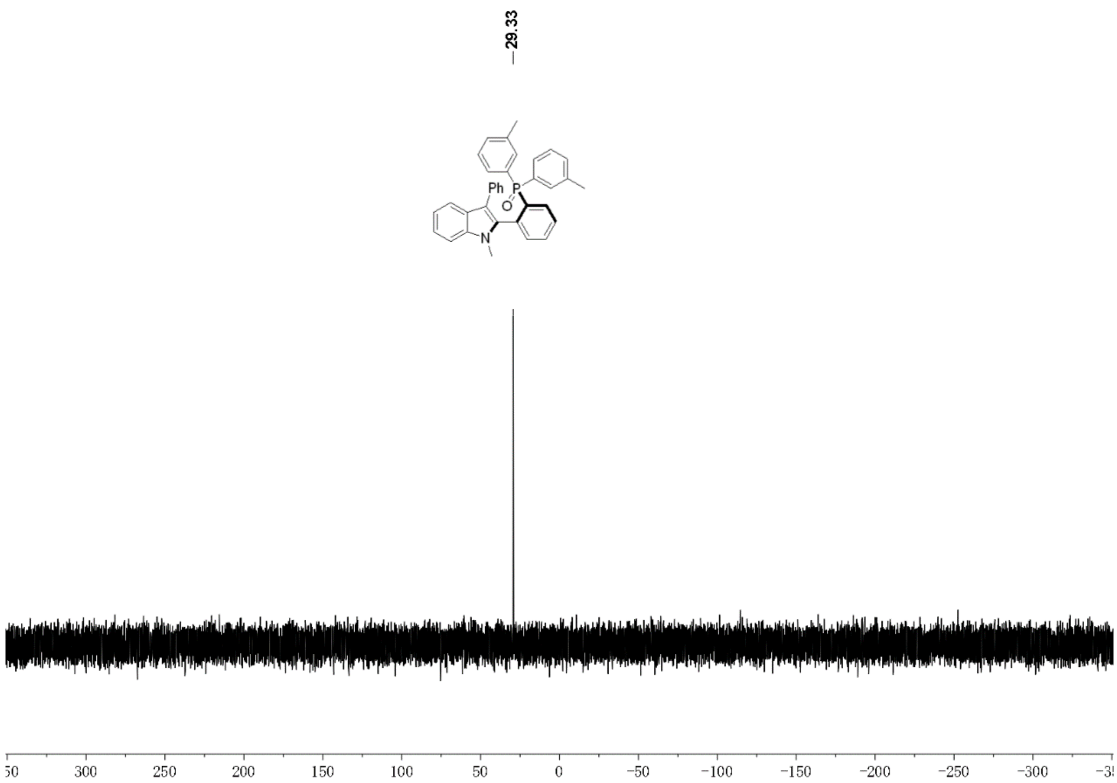

Supplementary Figure 207. ^{31}P NMR spectrum of **d12**

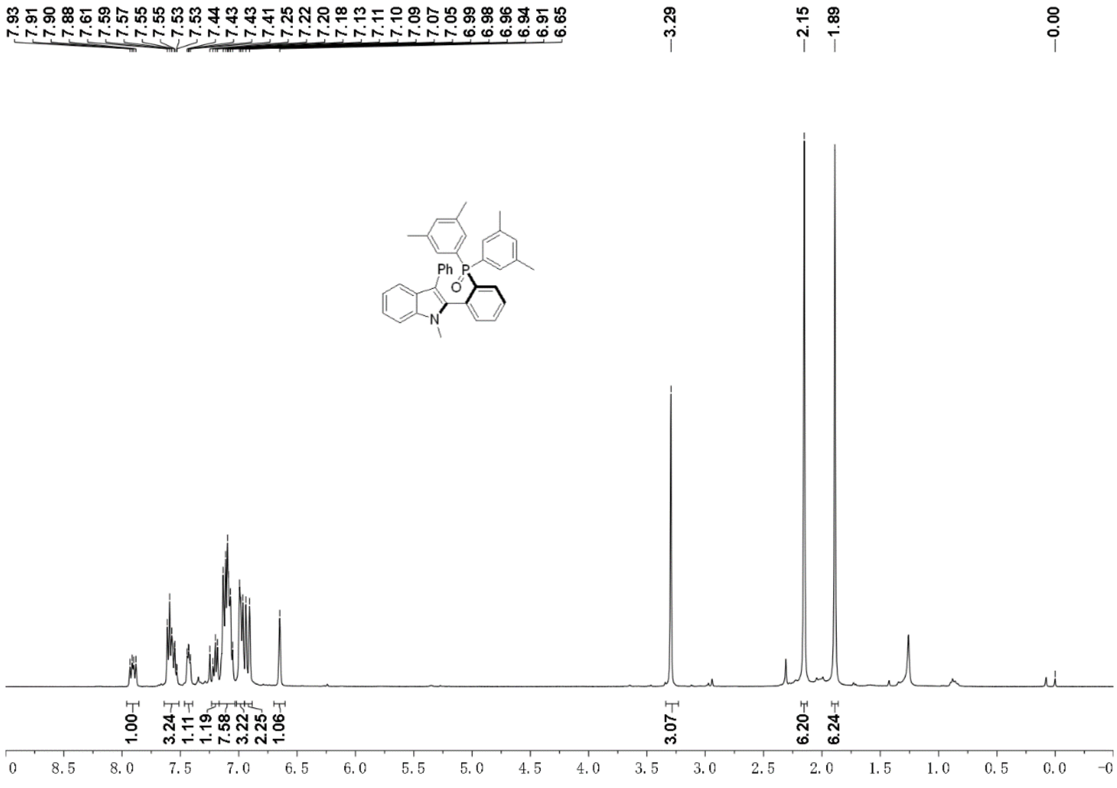

Supplementary Figure 208. ^{1}H NMR spectrum of **d13**

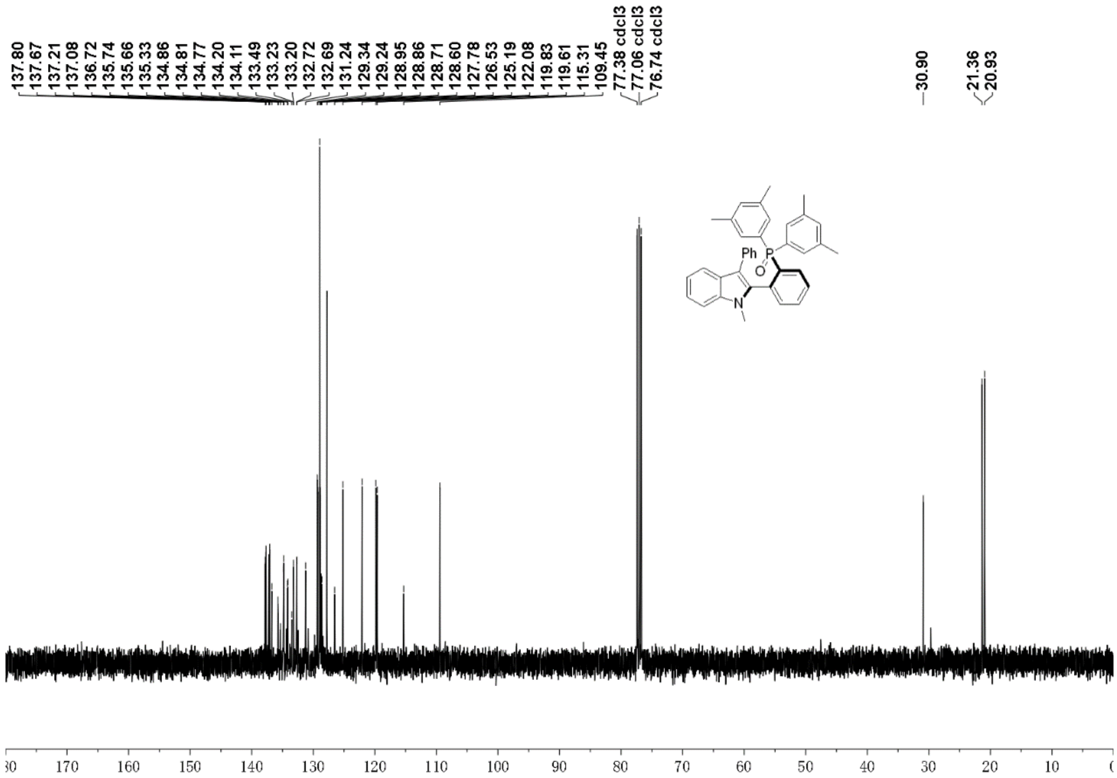

Supplementary Figure 209. ^{13}C NMR spectrum of **d13**

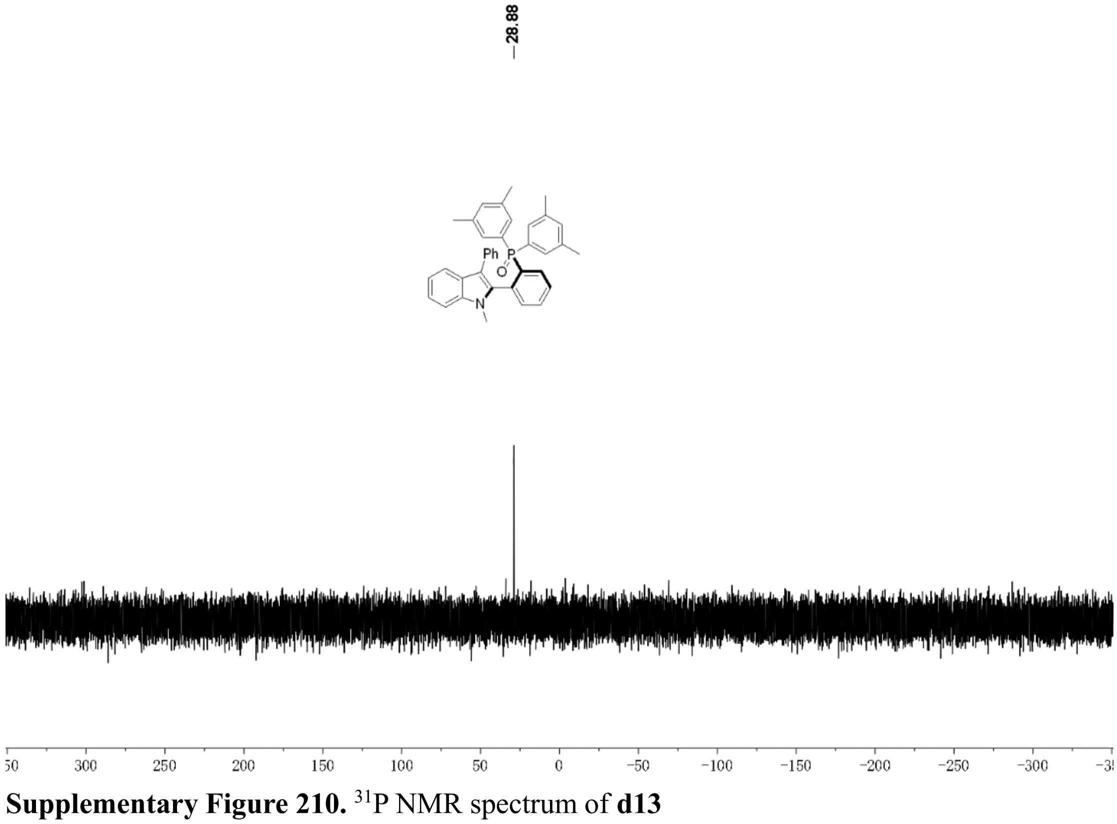

Supplementary Figure 210. ^{31}P NMR spectrum of **d13**

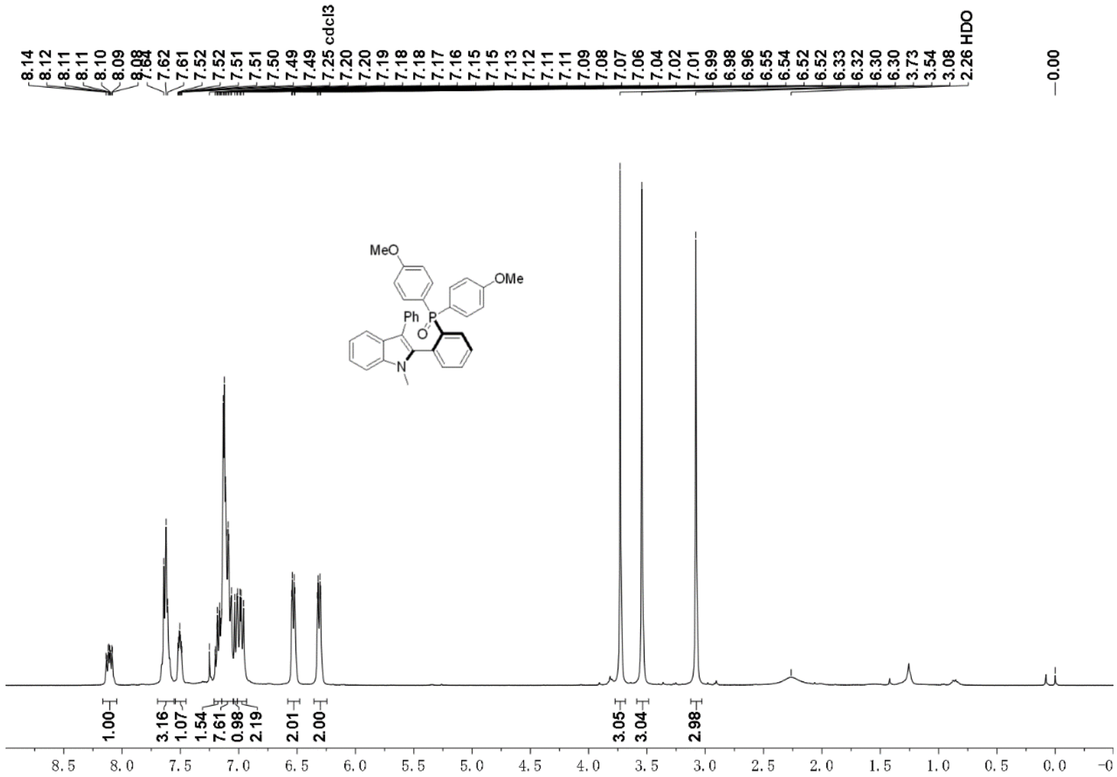

Supplementary Figure 211. ^{1}H NMR spectrum of **d14**

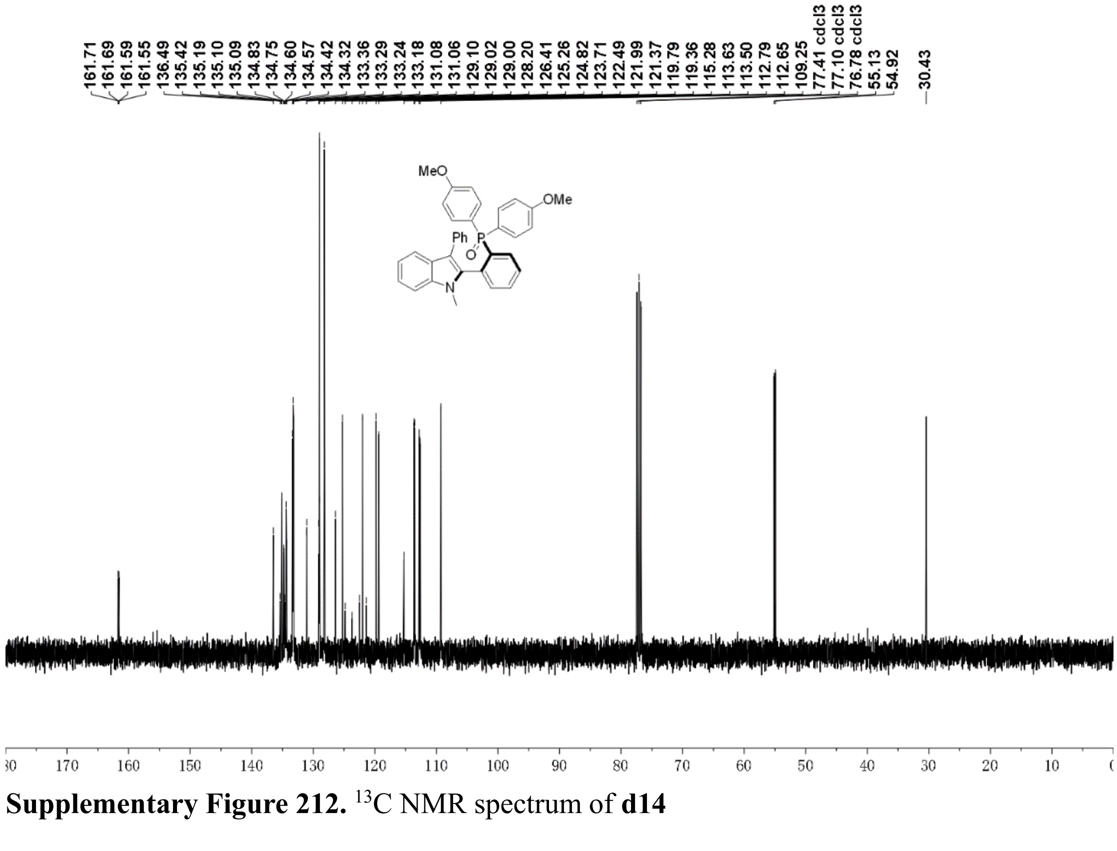

Supplementary Figure 212. ^{13}C NMR spectrum of **d14**

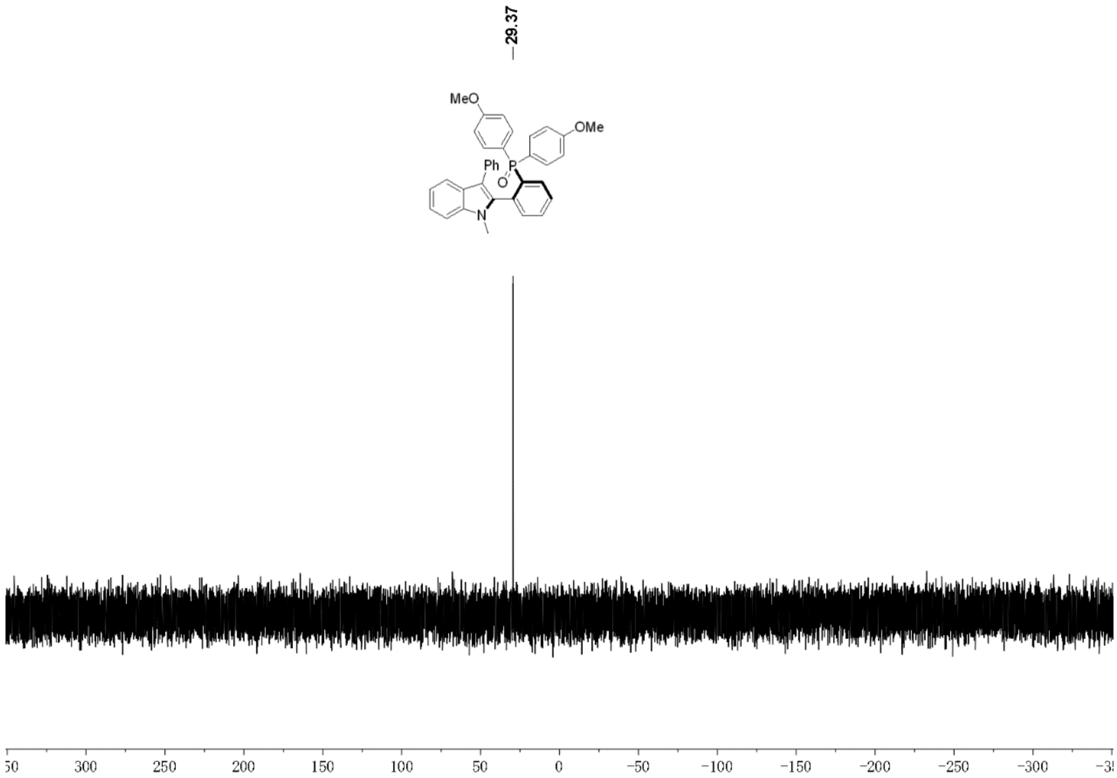

Supplementary Figure 213. ^{31}P NMR spectrum of **d14**

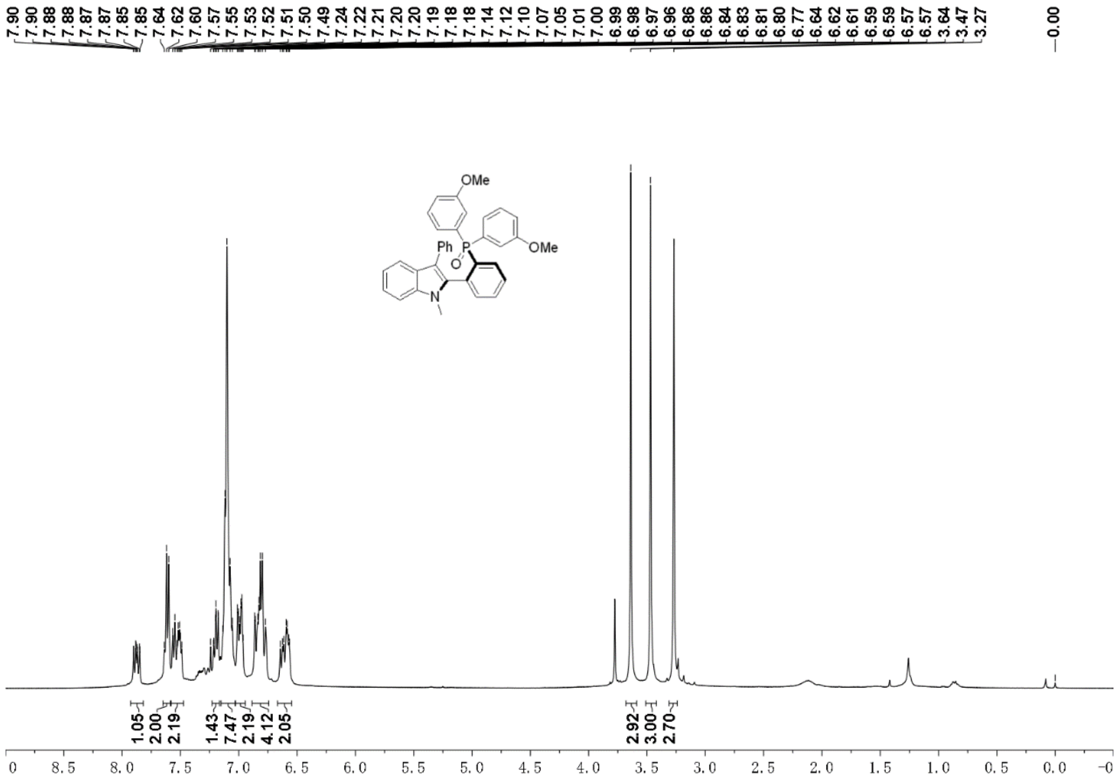

Supplementary Figure 214. ^{1}H NMR spectrum of **d15**

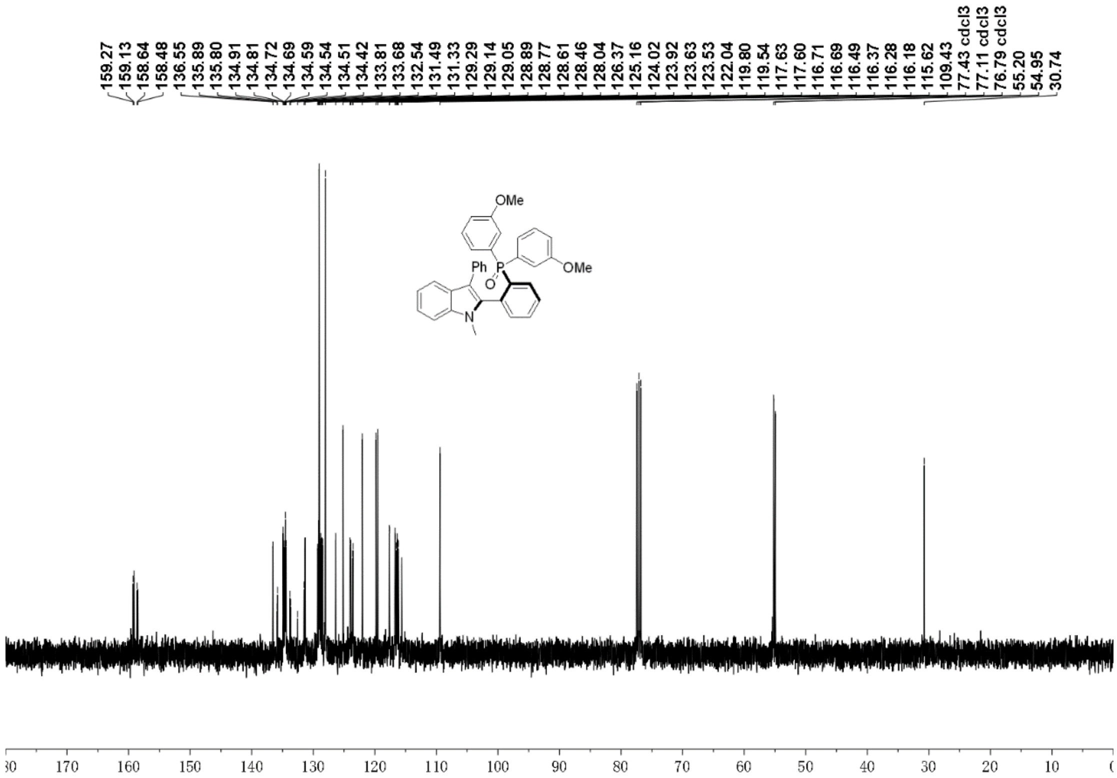

Supplementary Figure 215. ^{13}C NMR spectrum of **d15**

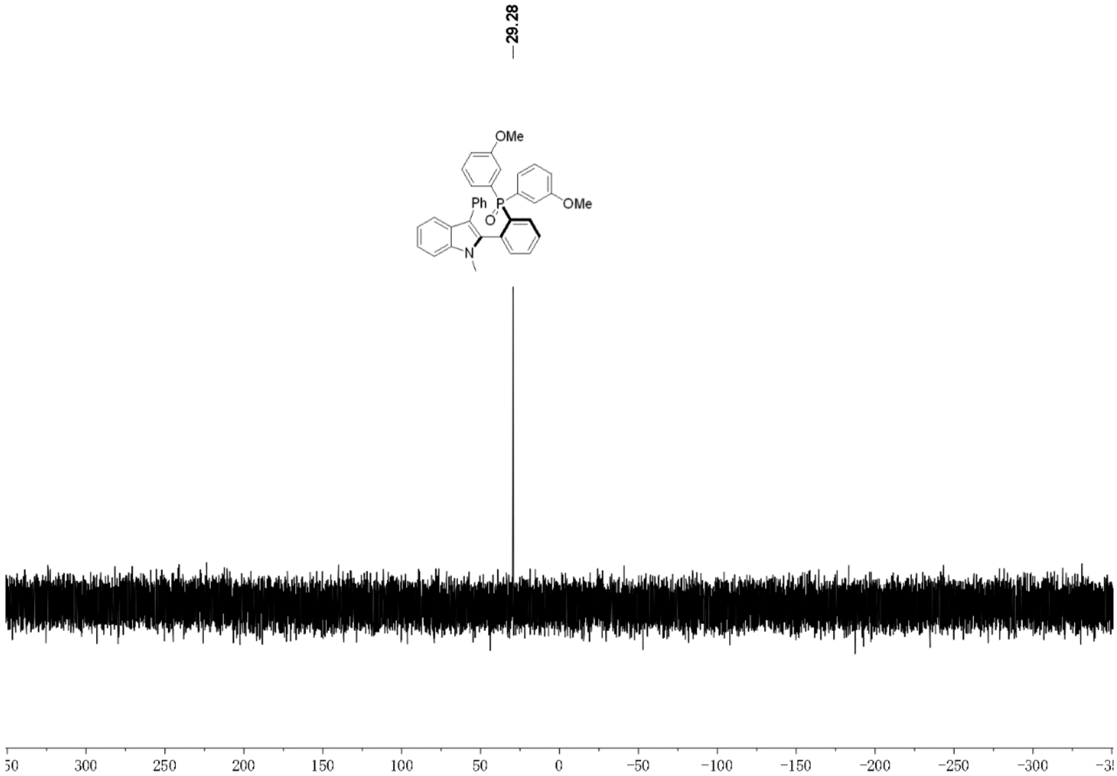

Supplementary Figure 216. ^{31}P NMR spectrum of **d15**

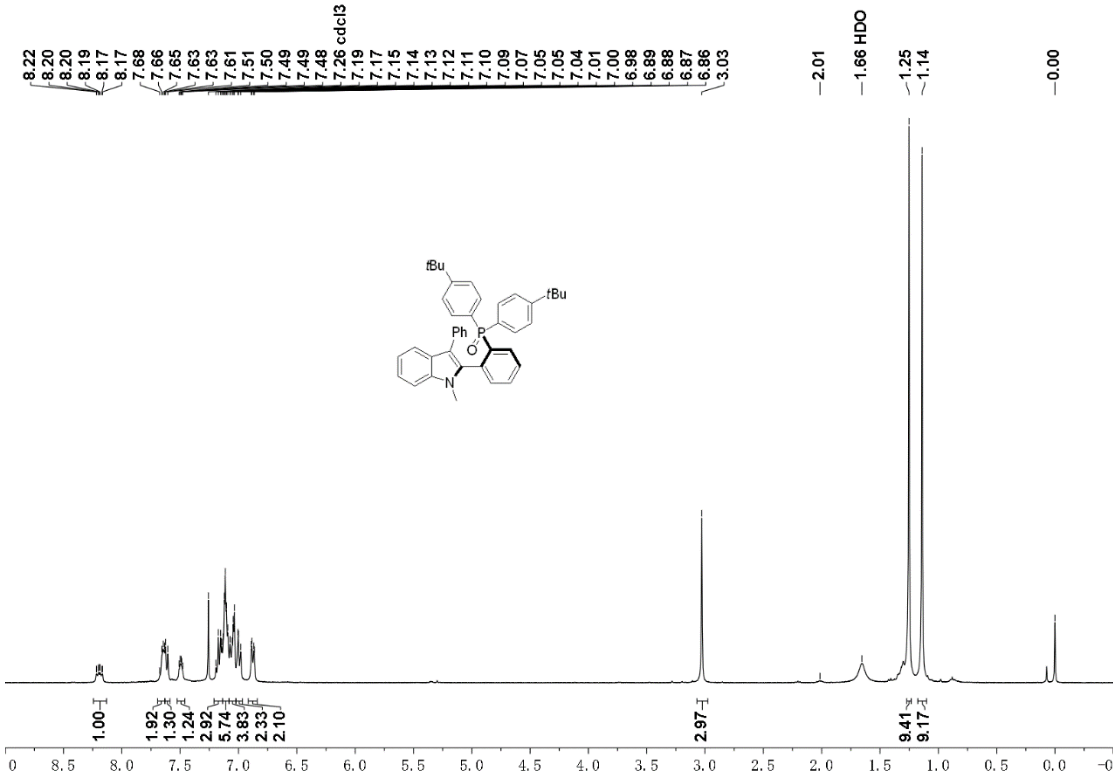

Supplementary Figure 217. ^{1}H NMR spectrum of **d16**

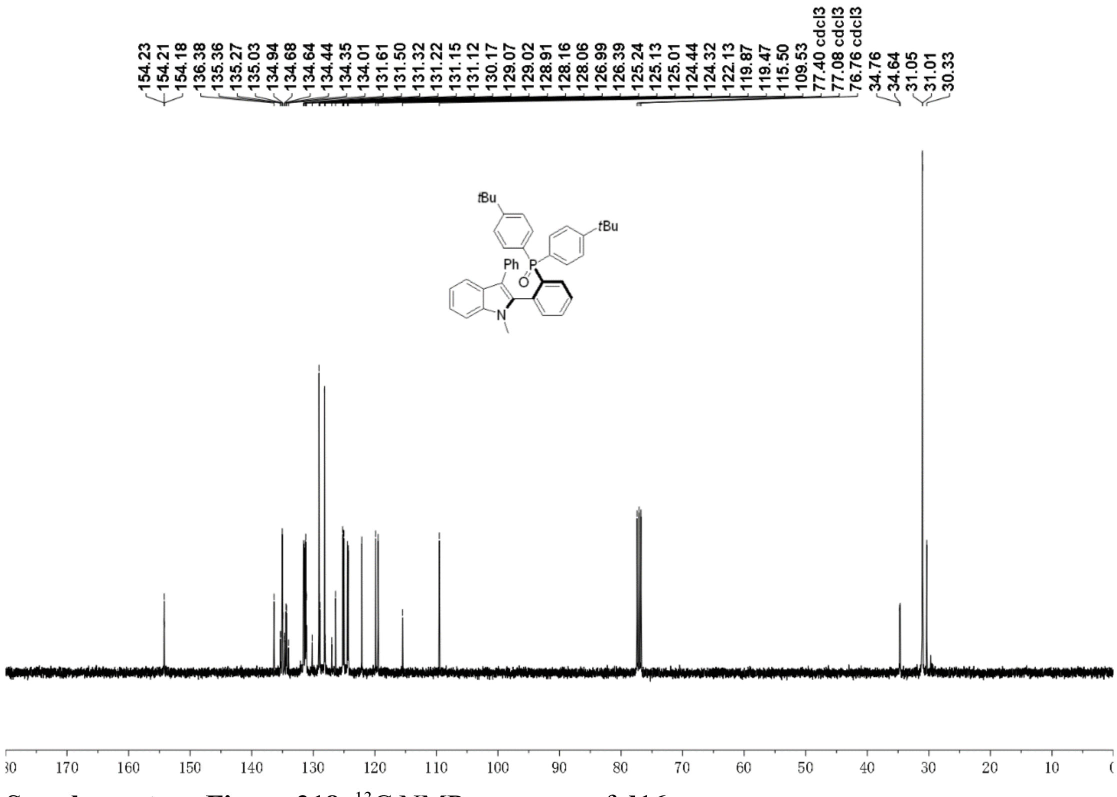

Supplementary Figure 218. ^{13}C NMR spectrum of **d16**

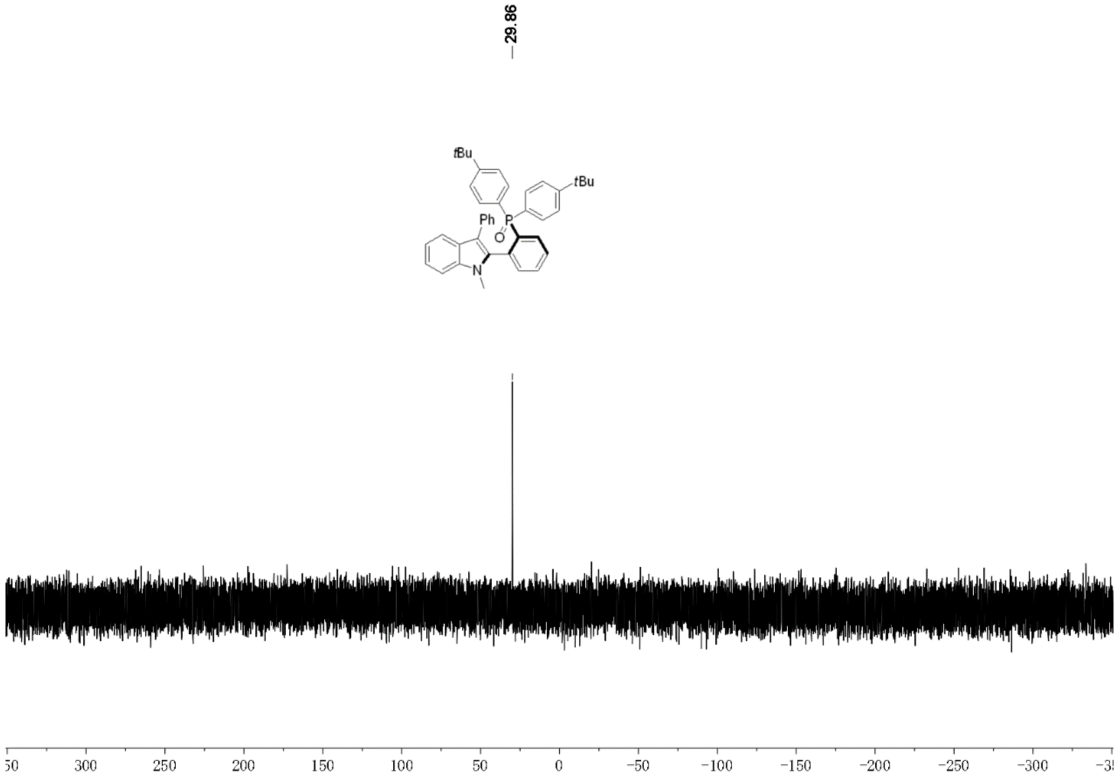

Supplementary Figure 219. ^{31}P NMR spectrum of **d16**

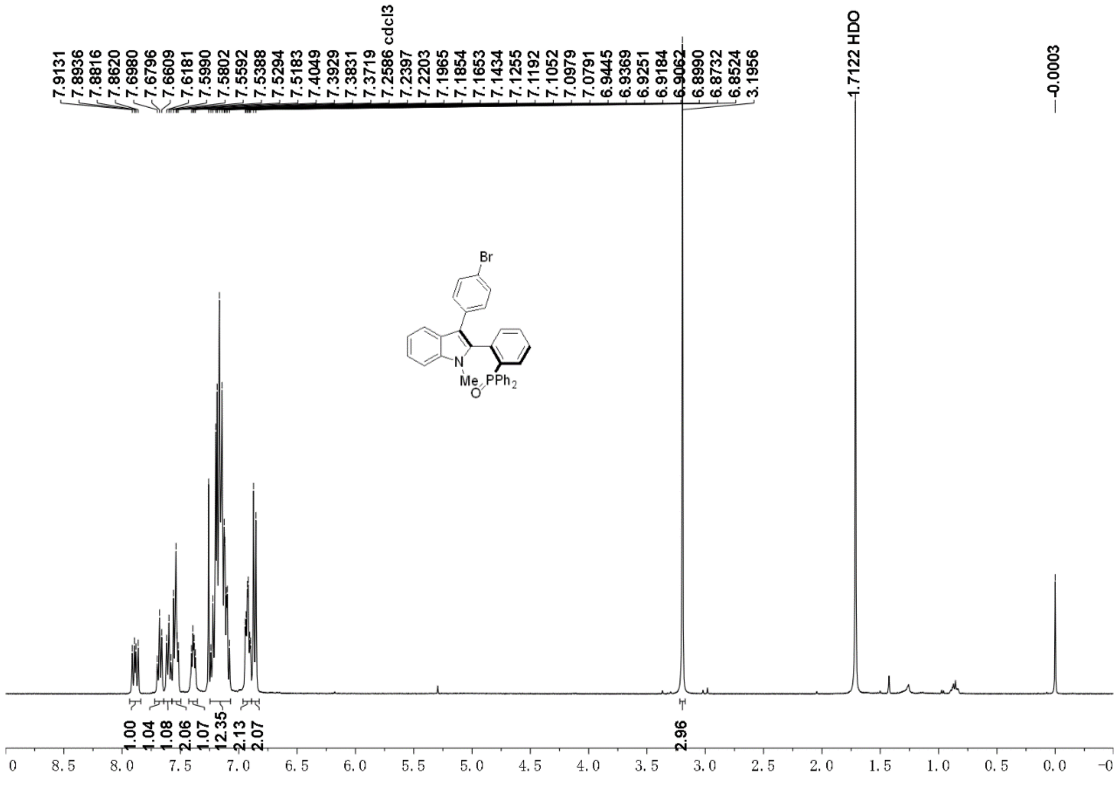

Supplementary Figure 220. ^{1}H NMR spectrum of **d17**

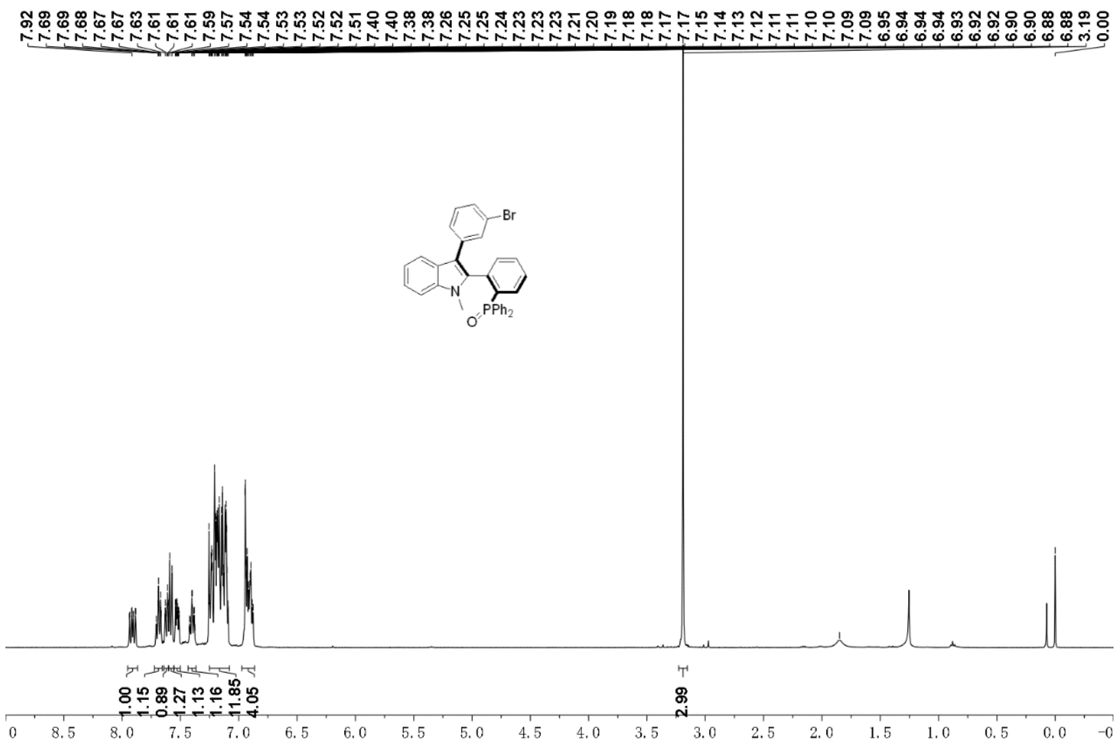

Supplementary Figure 221. ^{1}H NMR spectrum of **d18**

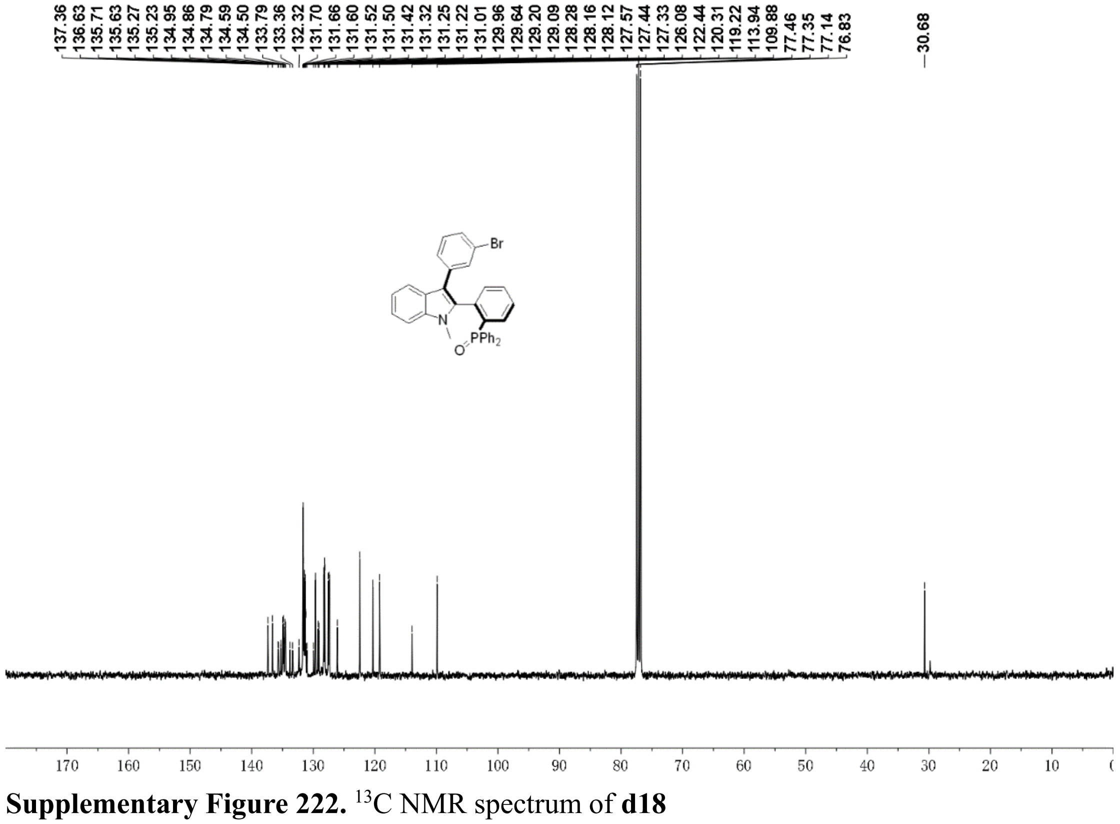

Supplementary Figure 222. ^{13}C NMR spectrum of **d18**

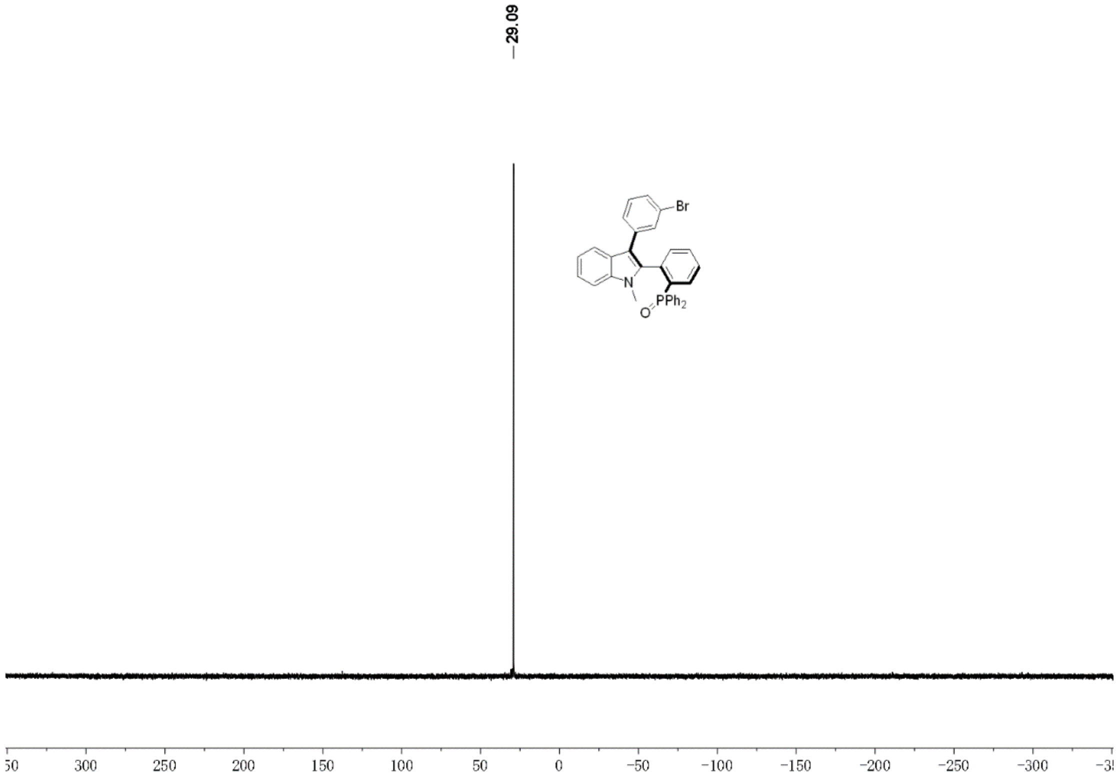

Supplementary Figure 223. ^{31}P NMR spectrum of **d18**

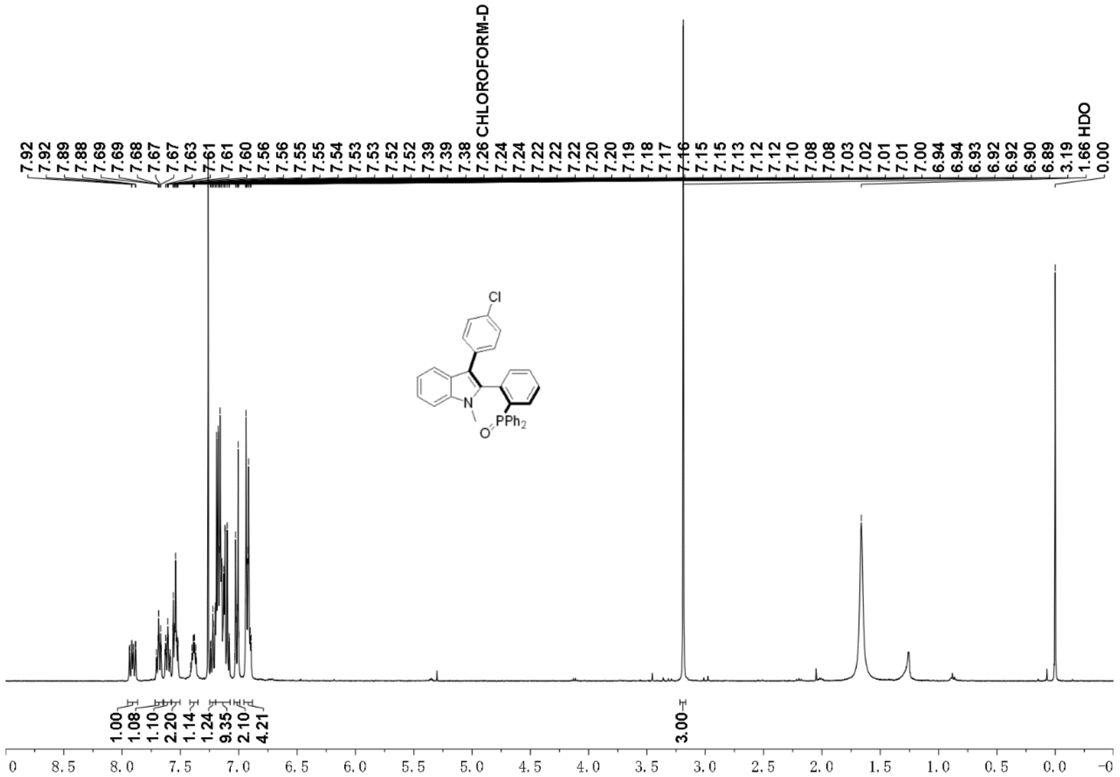

Supplementary Figure 224. ^{1}H NMR spectrum of **d19**

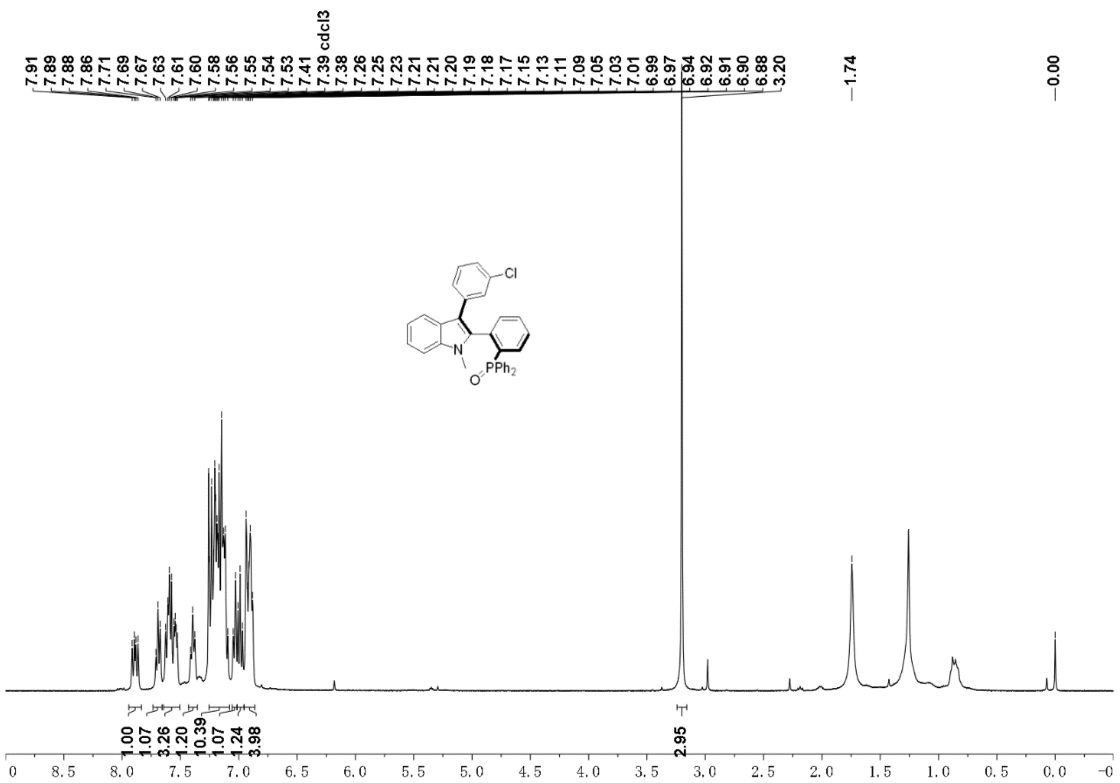

Supplementary Figure 225. ^{1}H NMR spectrum of **d20**

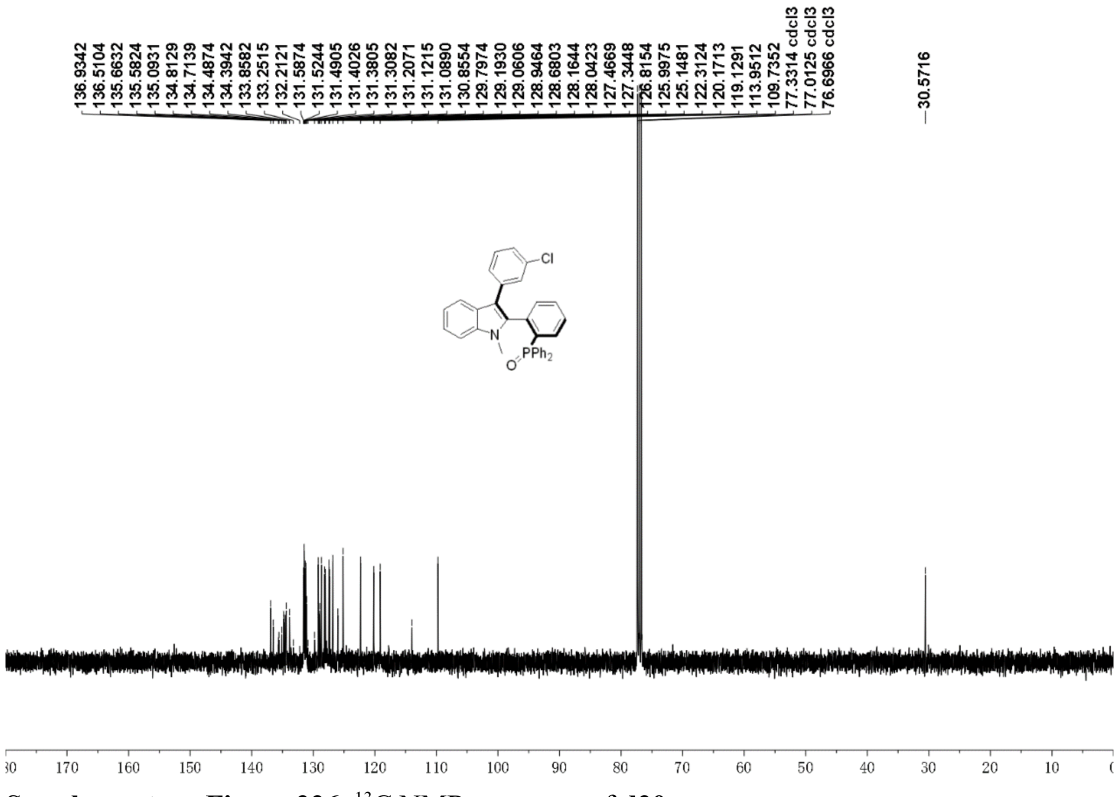

Supplementary Figure 226. ^{13}C NMR spectrum of **d20**

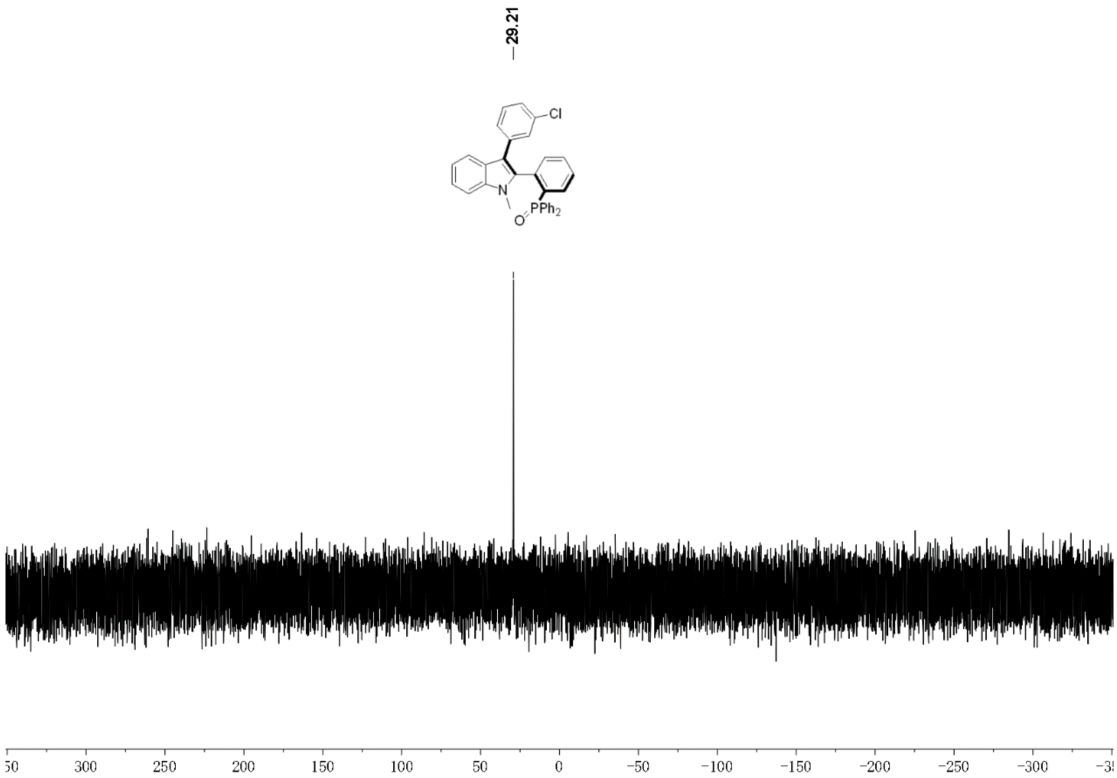

Supplementary Figure 227. ^{31}P NMR spectrum of **d20**

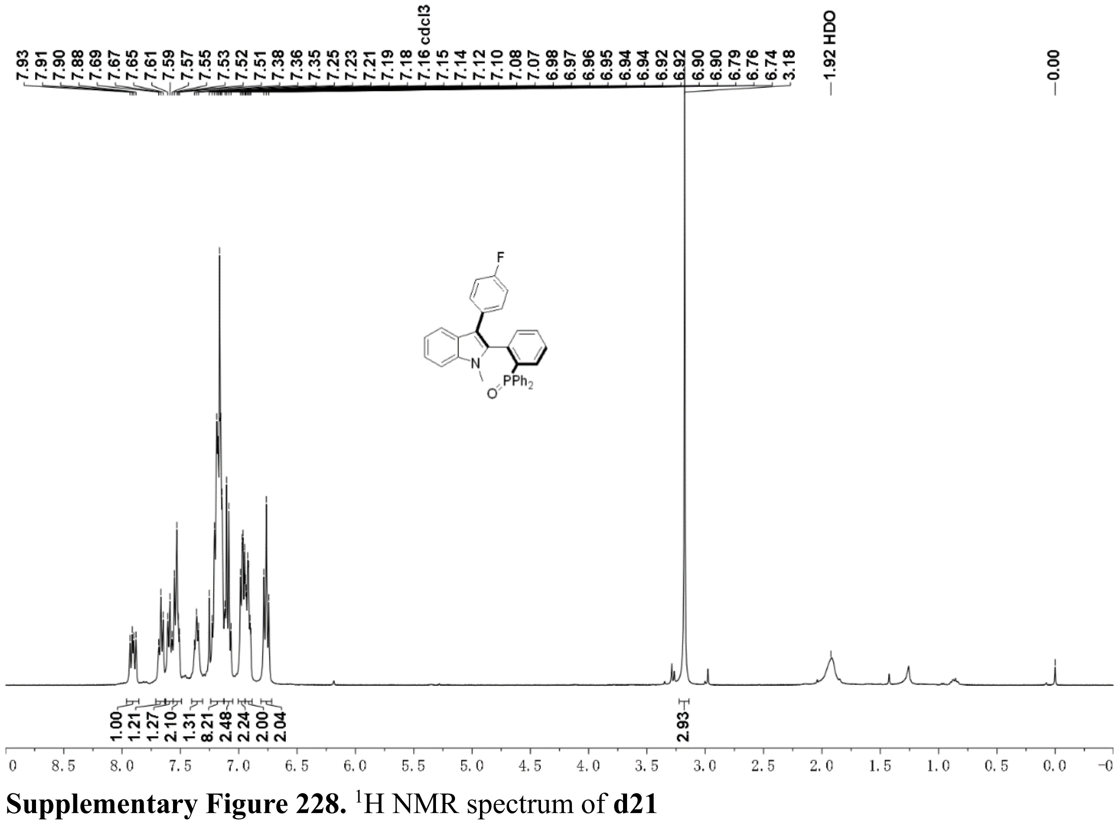

Supplementary Figure 228. ^{1}H NMR spectrum of **d21**

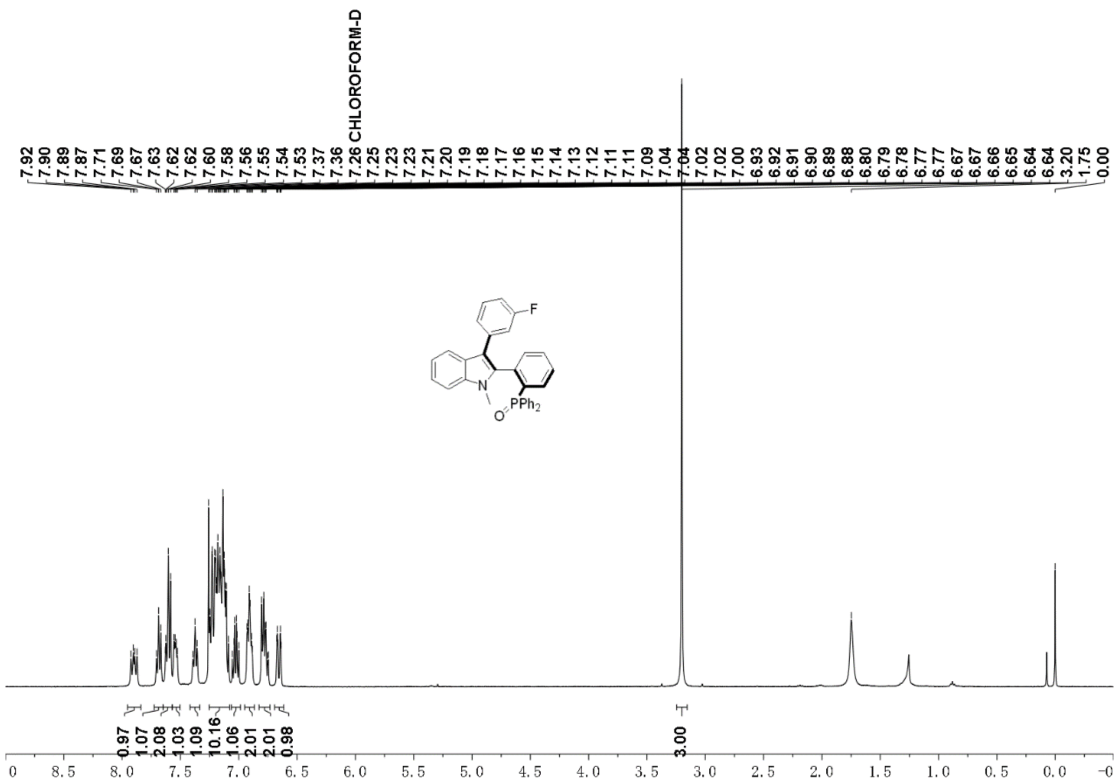

Supplementary Figure 229. ^{1}H NMR spectrum of **d22**

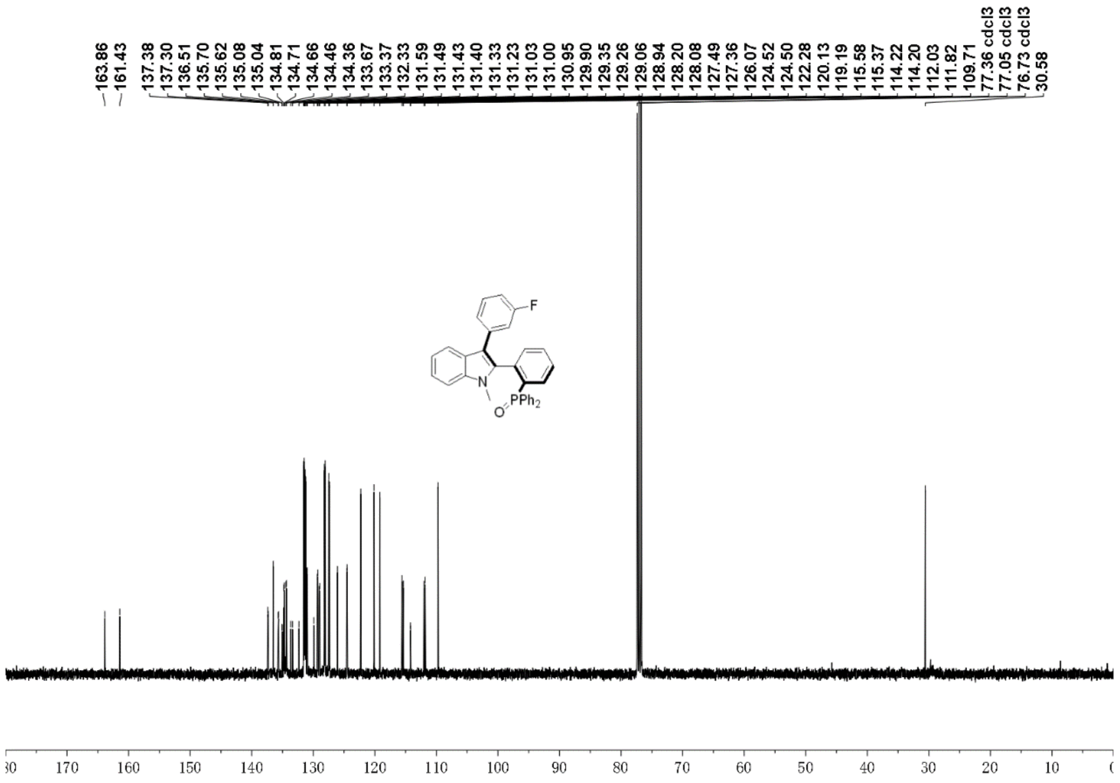

Supplementary Figure 230. ^{13}C NMR spectrum of **d22**

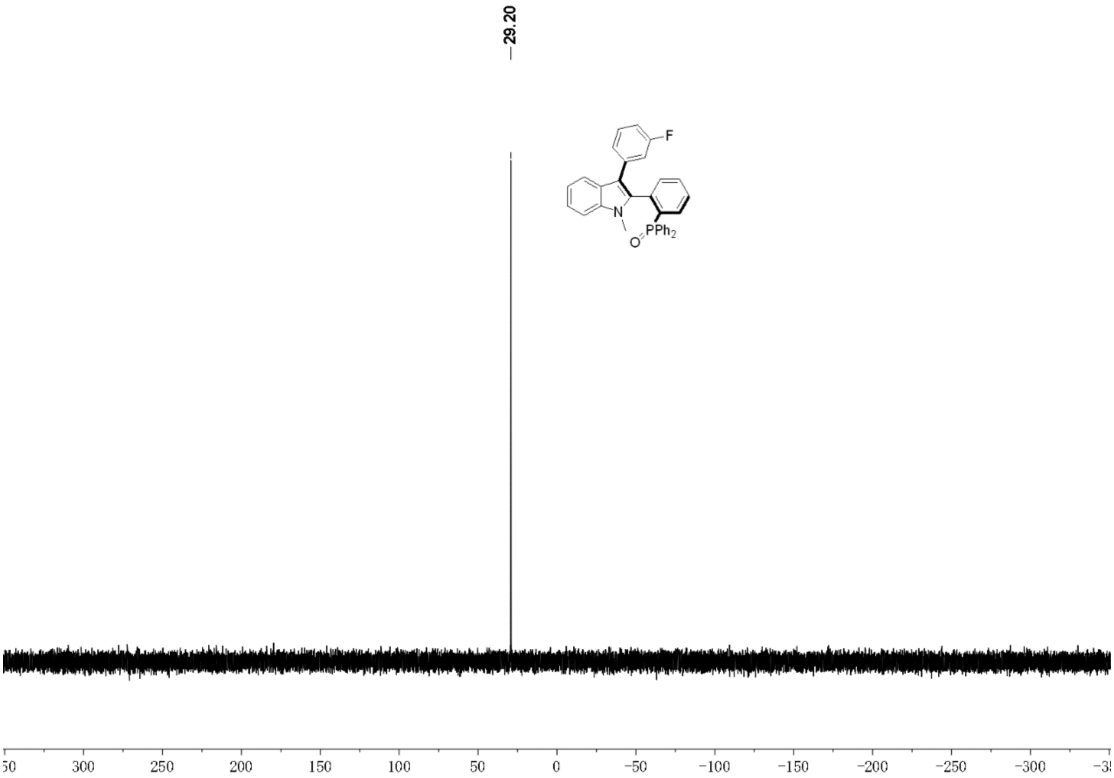

Supplementary Figure 231. ^{31}P NMR spectrum of **d22**

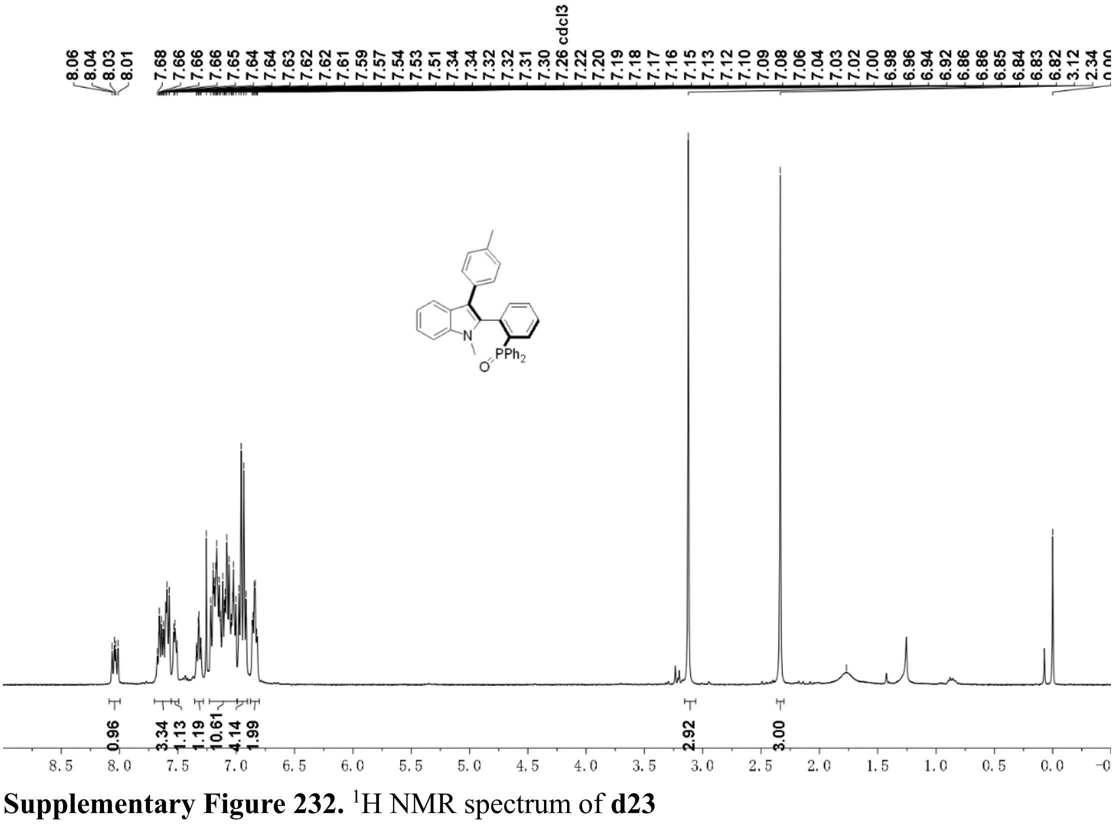

Supplementary Figure 232. ^{1}H NMR spectrum of **d23**

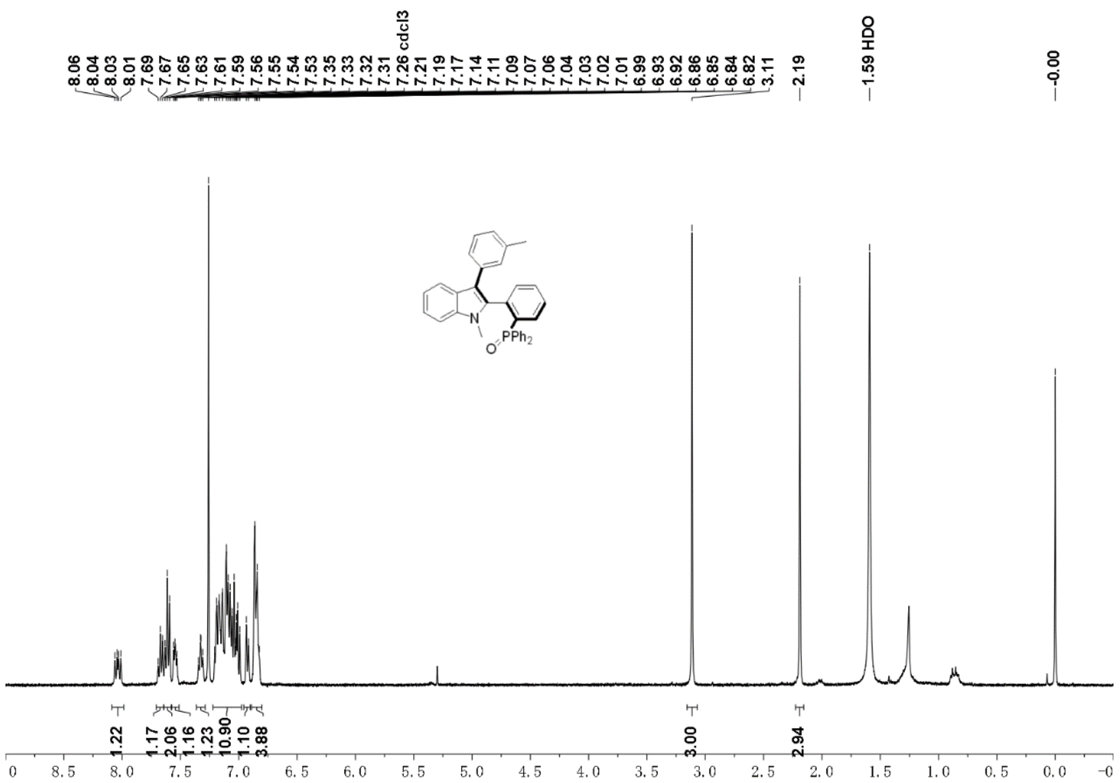

Supplementary Figure 233. ^{1}H NMR spectrum of **d24**

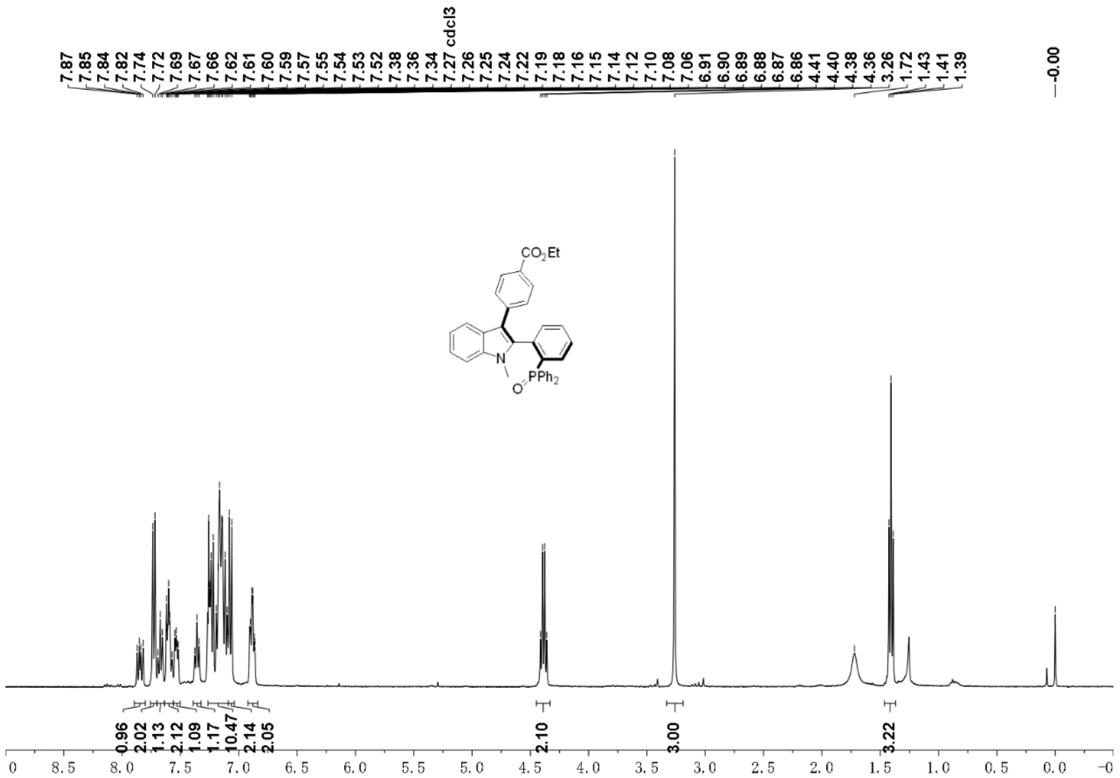

Supplementary Figure 234. ^{1}H NMR spectrum of **d25**

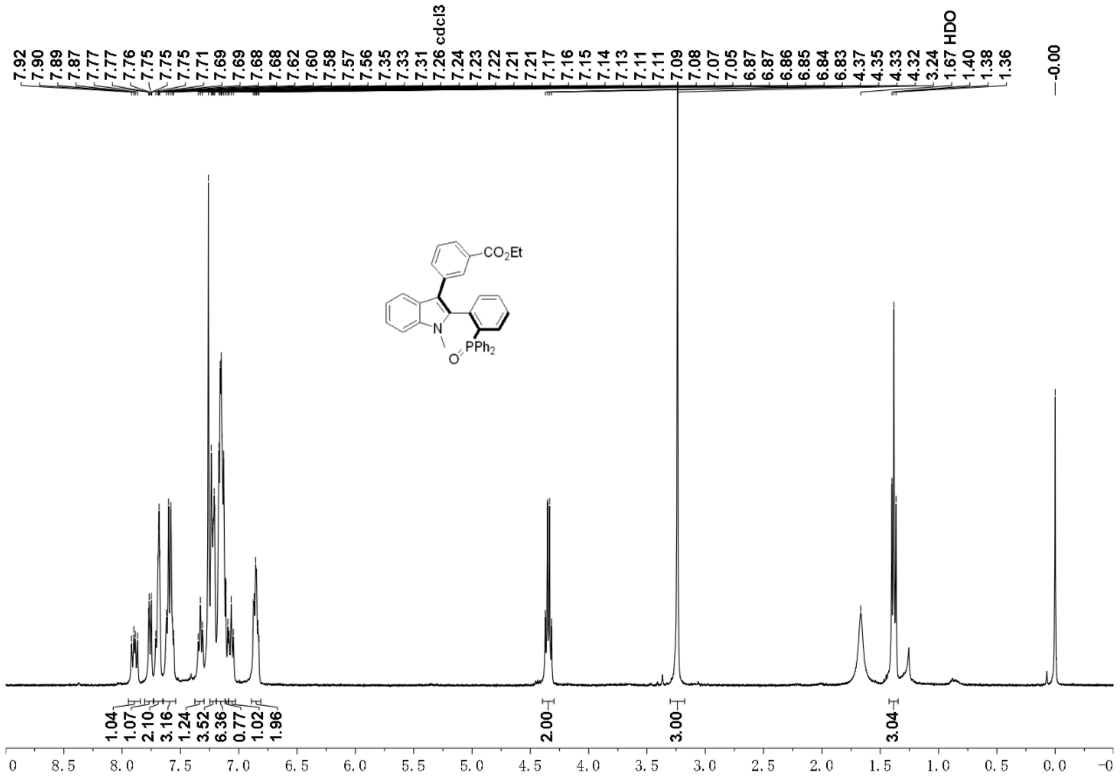

Supplementary Figure 235. ^{1}H NMR spectrum of **d26**

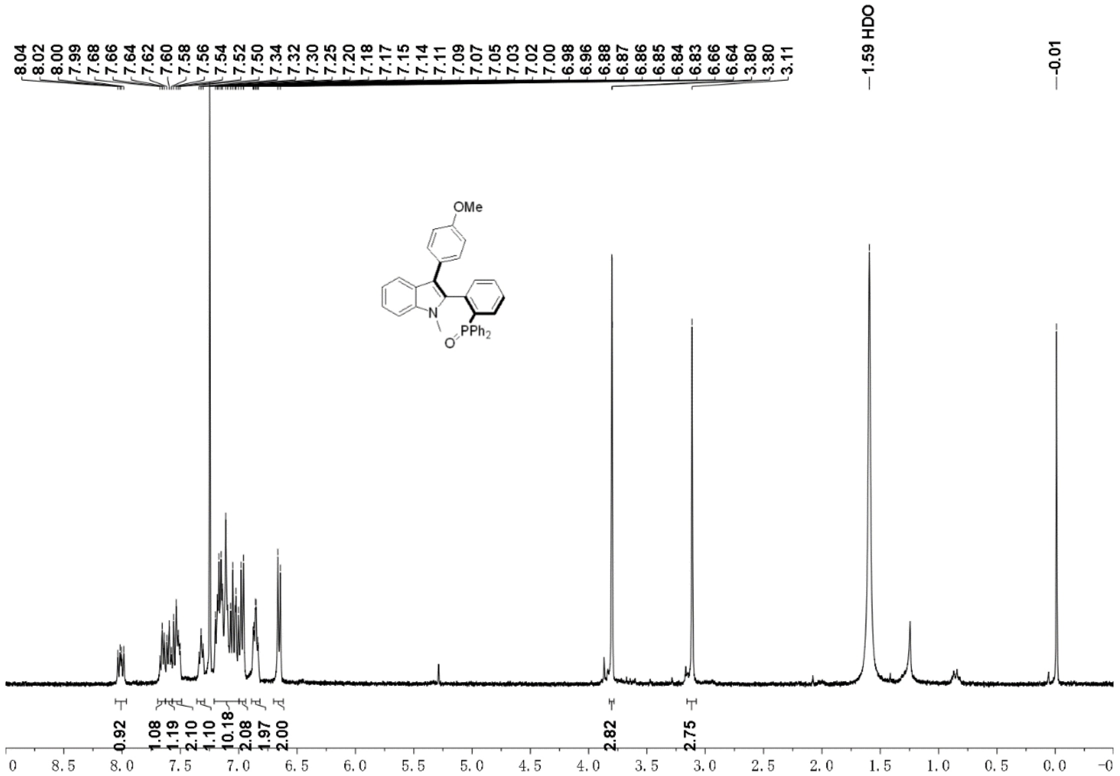

Supplementary Figure 236. ^{1}H NMR spectrum of **d27**

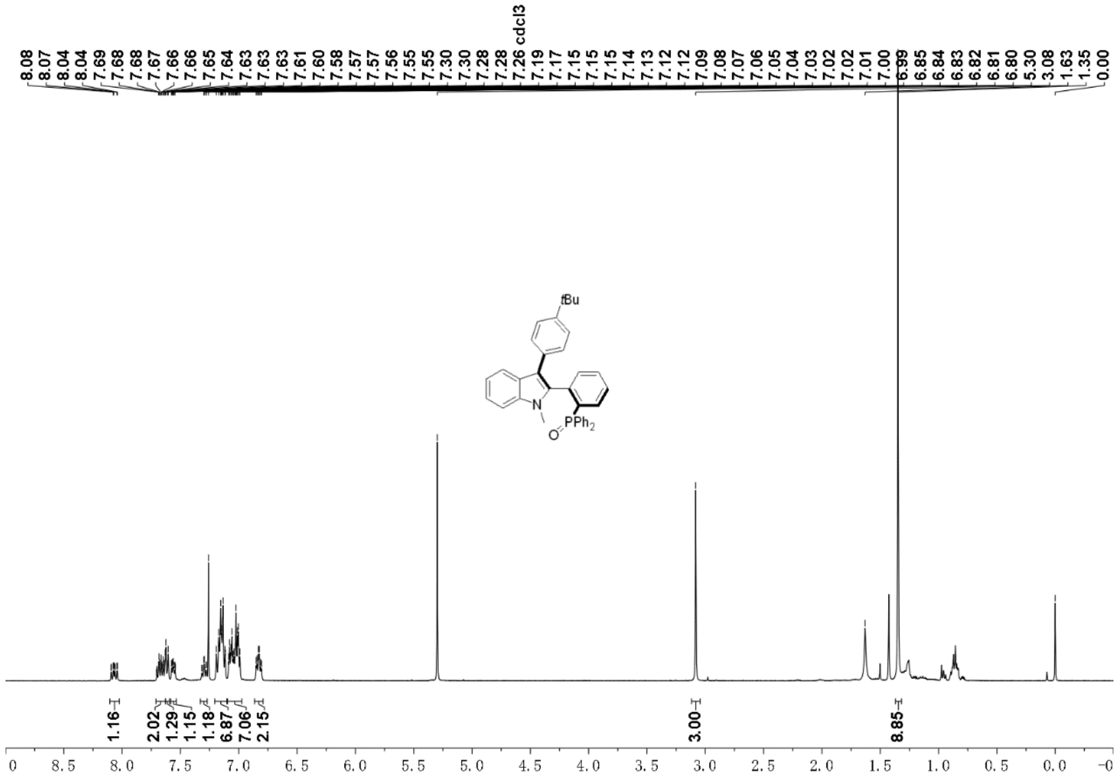

Supplementary Figure 237. ^{1}H NMR spectrum of **d28**

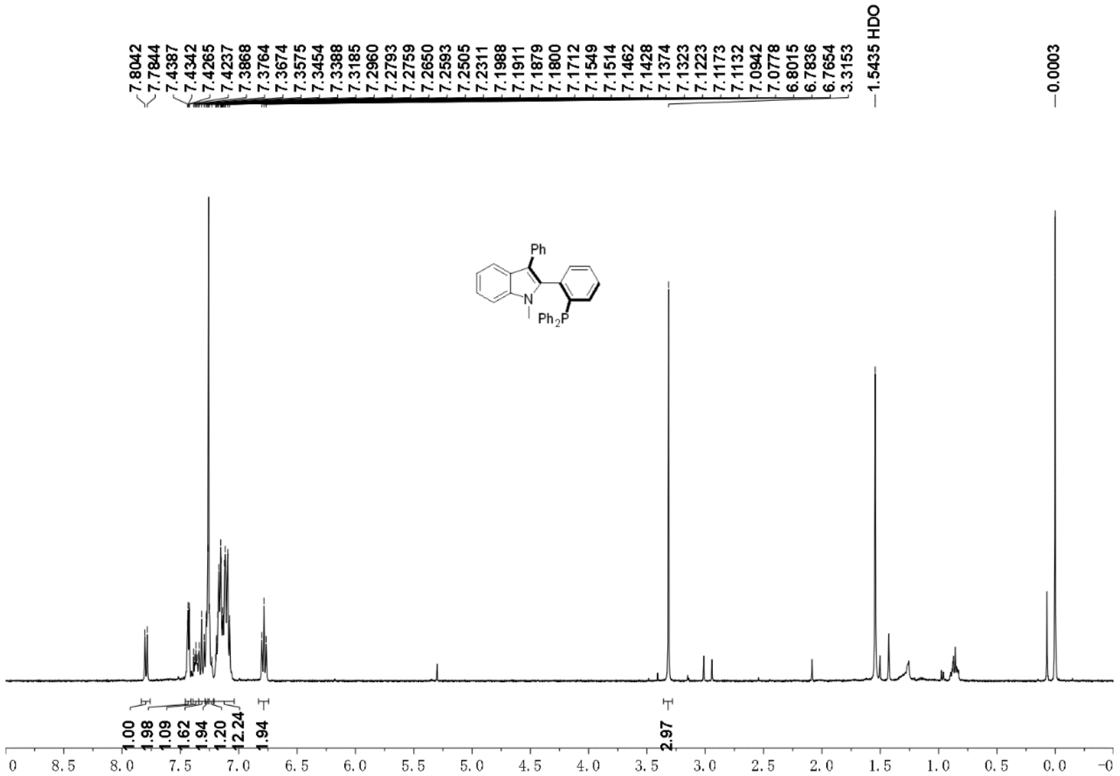

Supplementary Figure 238. ^{1}H NMR spectrum of **e1**

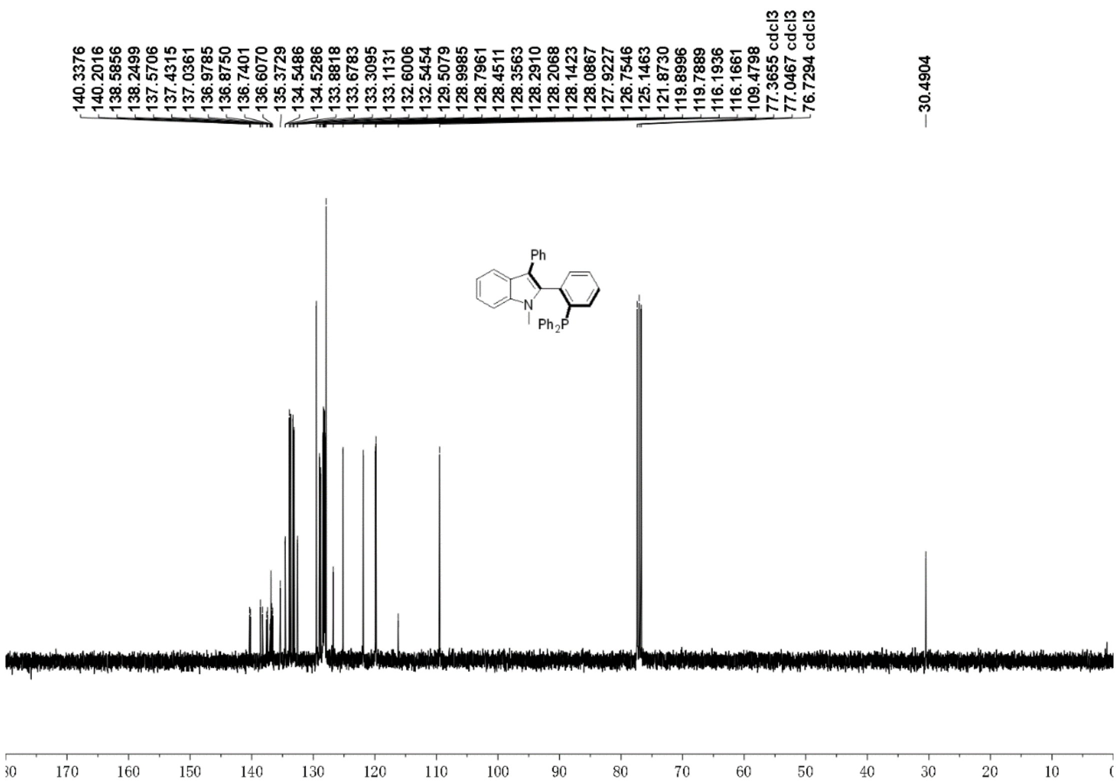

Supplementary Figure 239. ^{13}C NMR spectrum of **e1**

1.8 HPLC Charts

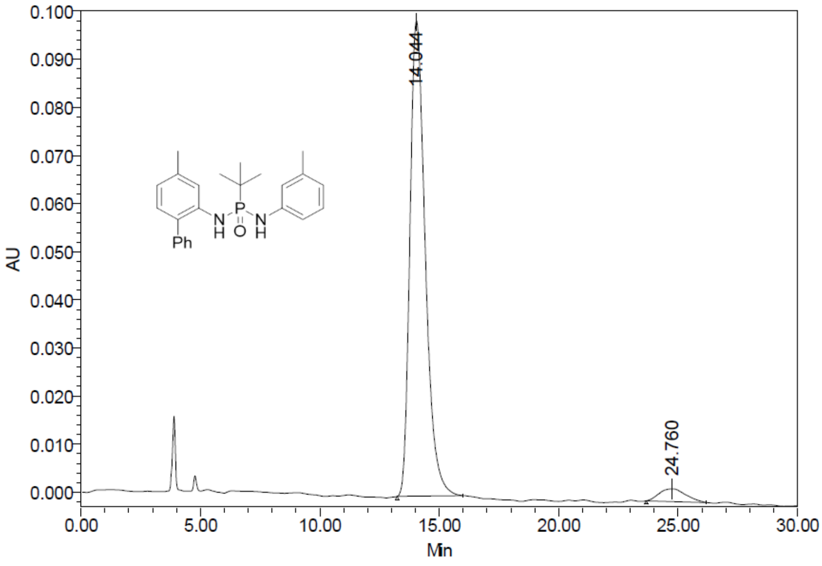

Peak Table

W2489 CHA 254

	Ret.Time	Height	Area	Area%
1	14.044	98806	4395866	95.545
2	24.760	2651	204984	4.455

Supplementary Figure 240. b1: OD-H, Hexane / *i*PrOH = 95/05, rate = 1.0 mL/min, 254 nm

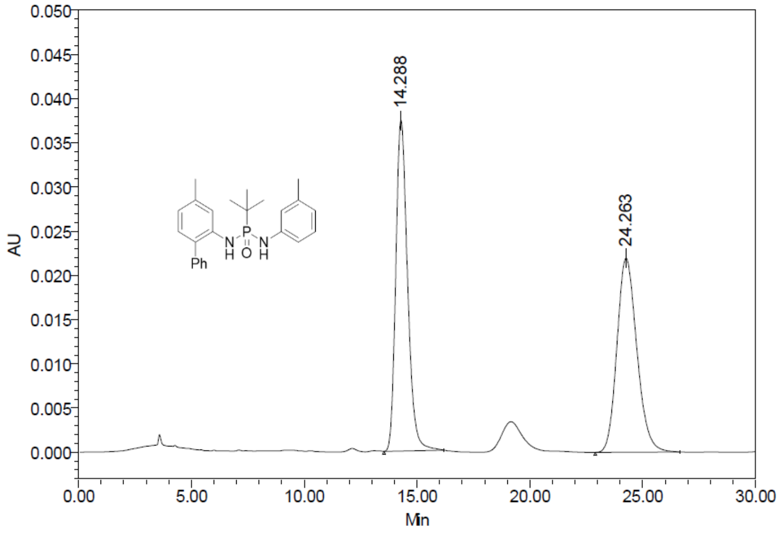

Peak Table

W2489 CHA 254

	Ret.Time	Height	Area	Area%
1	14.288	37426	1356314	50.158
2	24.263	21966	1347789	49.842

Supplementary Figure 241. rac-b1: OD-H, Hexane / *i*PrOH = 95/05, rate = 1.0 mL/min, 254 nm

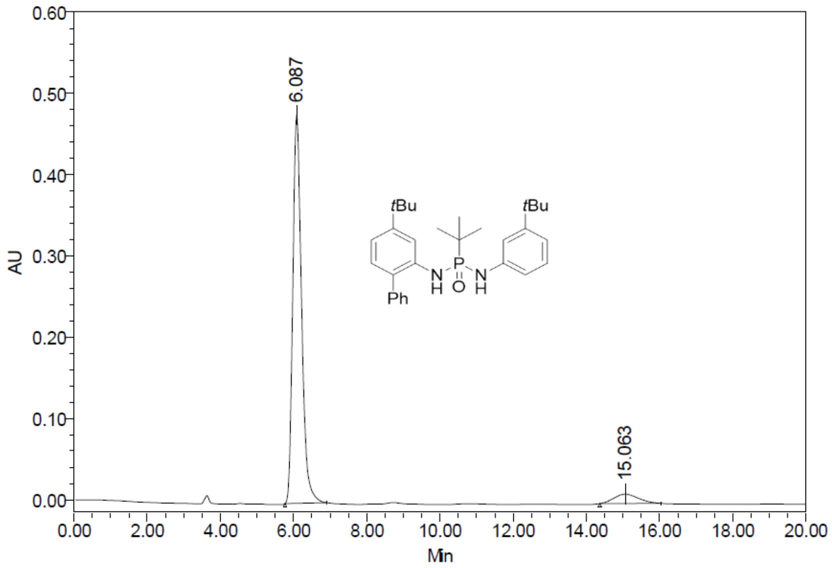

Peak Table

W2489 CHA 254

	Ret.Time	Height	Area	Area%
1	6.087	478430	7938610	93.647
2	15.063	11540	538599	6.353

Supplementary Figure 242. b2: OD-H, Hexane / *i*PrOH = 95/05, rate = 1.0 mL/min, 254 nm

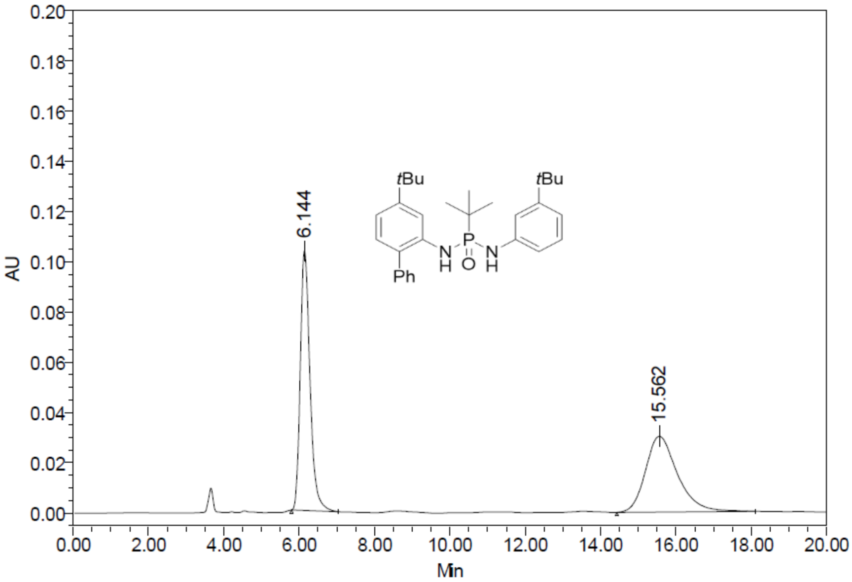

Peak Table

W2489 CHA 254

	Ret.Time	Height	Area	Area%
1	6.144	103639	1854306	50.953
2	15.562	30171	1784938	49.047

Supplementary Figure 243. rac-b2: OD-H, Hexane / *i*PrOH = 95/05, rate = 1.0 mL/min, 254 nm

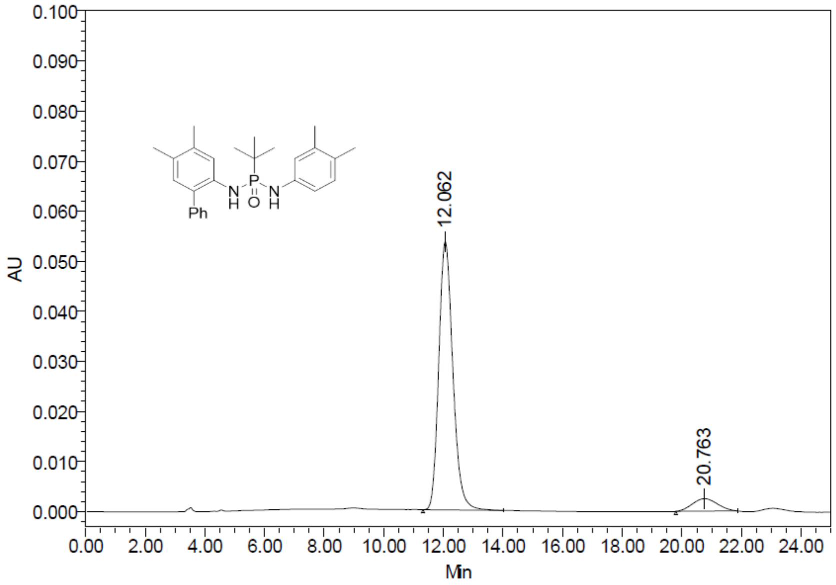

Peak Table

W2489 CHA 254

	Ret. Time	Height	Area	Area%
1	12.062	53575	1755049	92.303
2	20.763	2477	146359	7.697

Supplementary Figure 244. b3: OD-H, Hexane / *i*PrOH = 90/10, rate = 1.0 mL/min, 254 nm

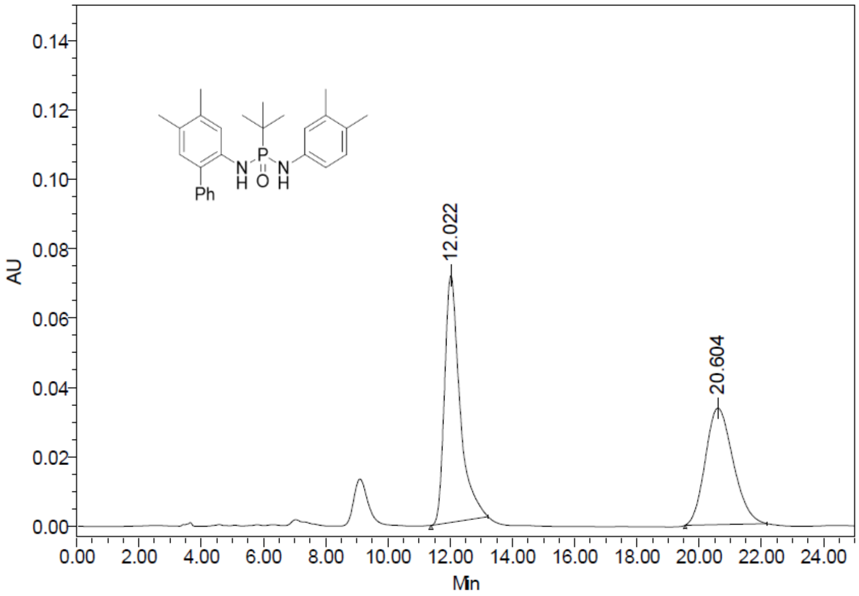

Peak Table

W2489 CHA 254

	Ret. Time	Height	Area	Area%
1	12.022	71171	2434228	53.870
2	20.604	33559	2084446	46.130

Supplementary Figure 245. rac-b3: OD-H, Hexane / *i*PrOH = 90/10, rate = 1.0 mL/min, 254 nm

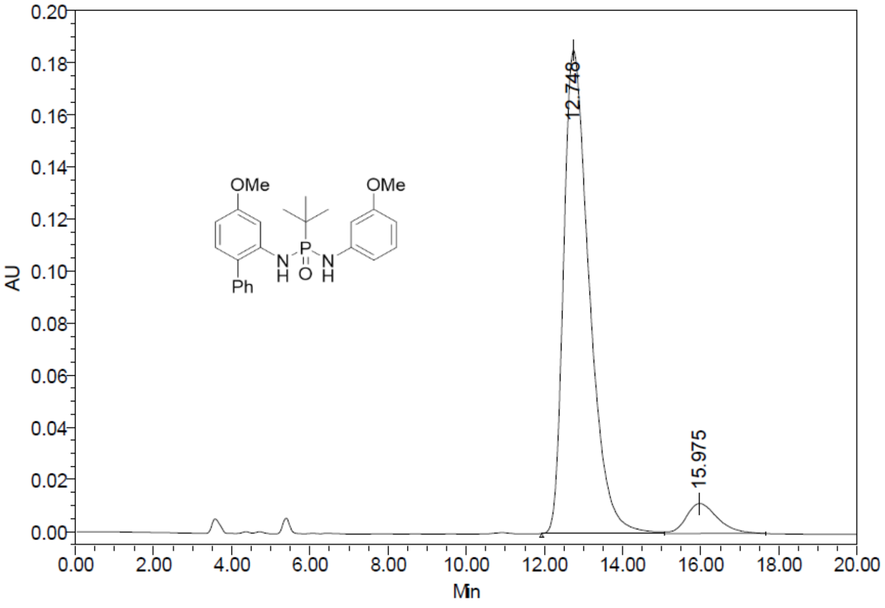

Peak Table

W2489 CHA 254

	Ret.Time	Height	Area	Area%
1	12.748	185671	8448634	92.907
2	15.975	11500	645003	7.093

Supplementary Figure 246. b4: OD-H, Hexane / *i*PrOH = 90/10, rate = 1.0 mL/min, 254 nm

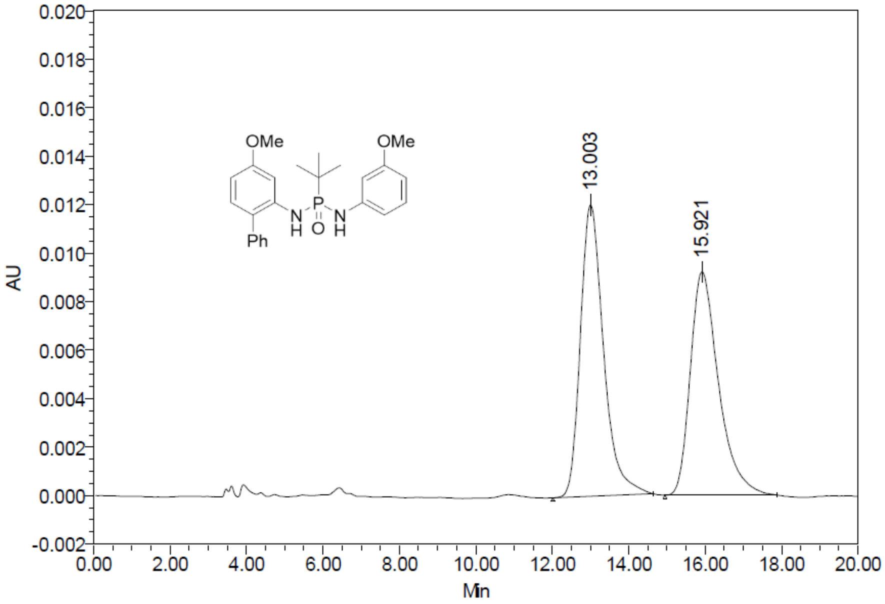

Peak Table

W2489 CHA 254

	Ret.Time	Height	Area	Area%
1	13.003	12019	506564	51.721
2	15.921	9211	472855	48.279

Supplementary Figure 247. rac-b4: OD-H, Hexane / *i*PrOH = 90/10, rate = 1.0 mL/min, 254 nm

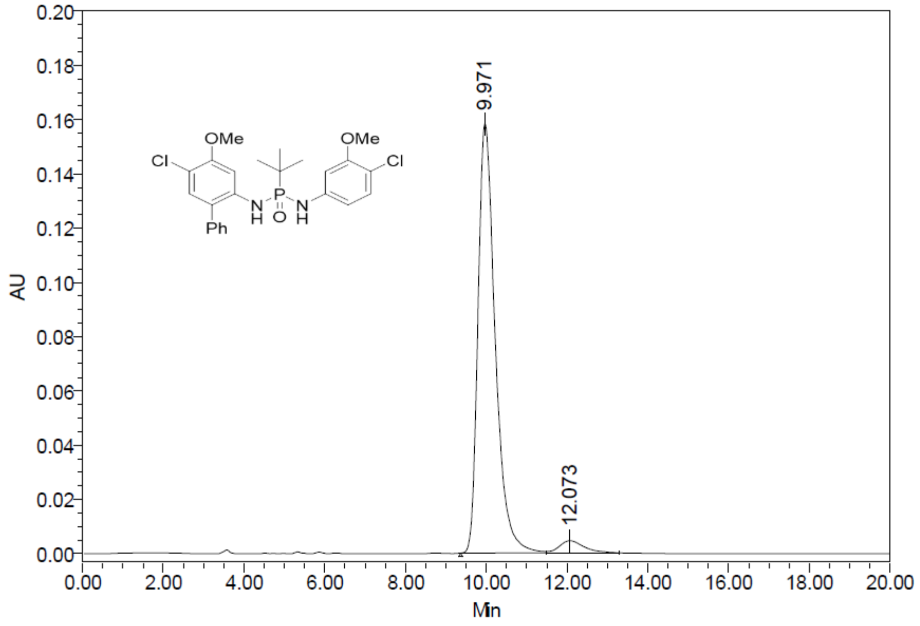

Peak Table

W2489 CHA 254

	Ret.Time	Height	Area	Area%
1	9.971	158291	4690045	96.052
2	12.073	4511	192768	3.948

Supplementary Figure 248. b5: OD-H, Hexane / *i*PrOH = 90/10, rate = 1.0 mL/min, 254 nm

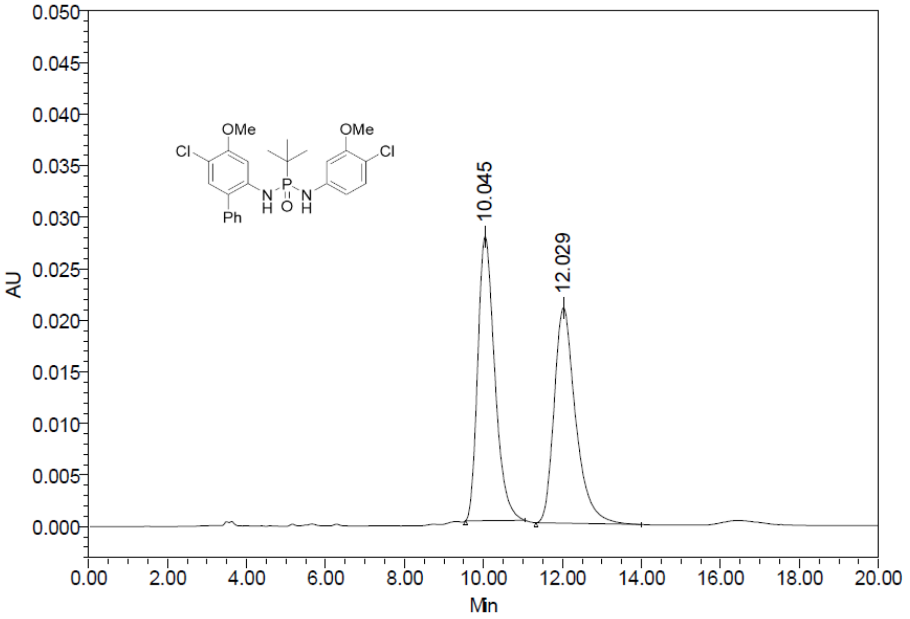

Peak Table

W2489 CHA 254

	Ret.Time	Height	Area	Area%
1	10.045	27542	822980	50.986
2	12.029	20895	791137	49.014

Supplementary Figure 249. rac-b5: OD-H, Hexane / *i*PrOH = 90/10, rate = 1.0 mL/min, 254 nm

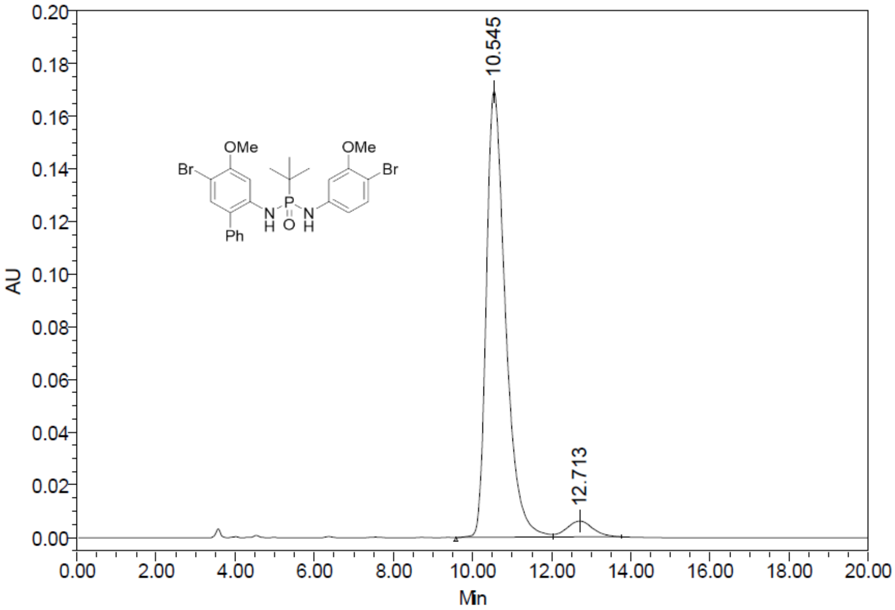

Peak Table

W2489 CHA 254

	Ret.Time	Height	Area	Area%
1	10.545	169448	5697605	95.332
2	12.713	6036	279014	4.668

Supplementary Figure 250. b6: OD-H, Hexane / *i*PrOH = 90/10, rate = 1.0 mL/min, 254 nm

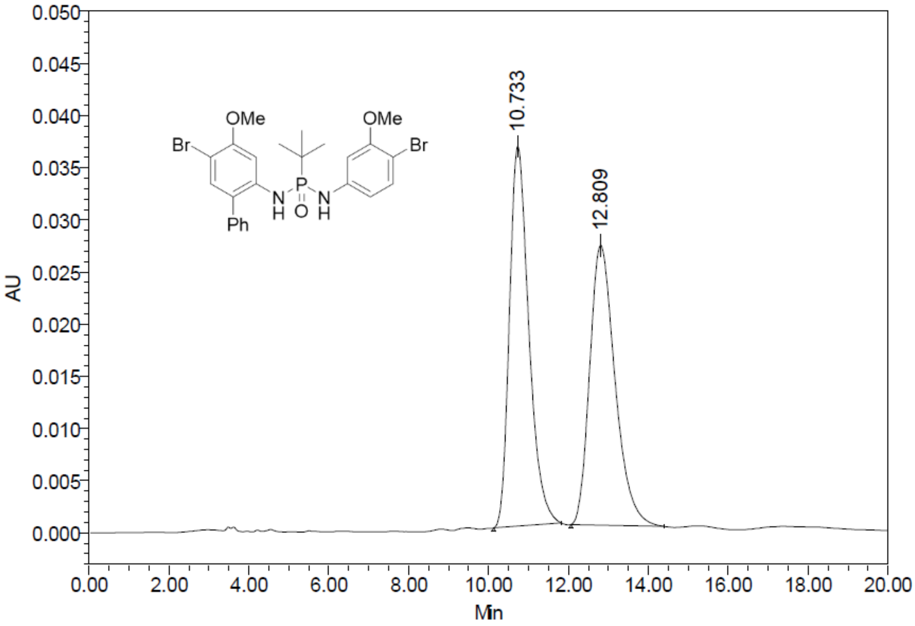

Peak Table

W2489 CHA 254

	Ret.Time	Height	Area	Area%
1	10.733	36411	1227128	50.935
2	12.809	26838	1182066	49.065

Supplementary Figure 251. rac-b6: OD-H, Hexane / *i*PrOH = 90/10, rate = 1.0 mL/min, 254 nm

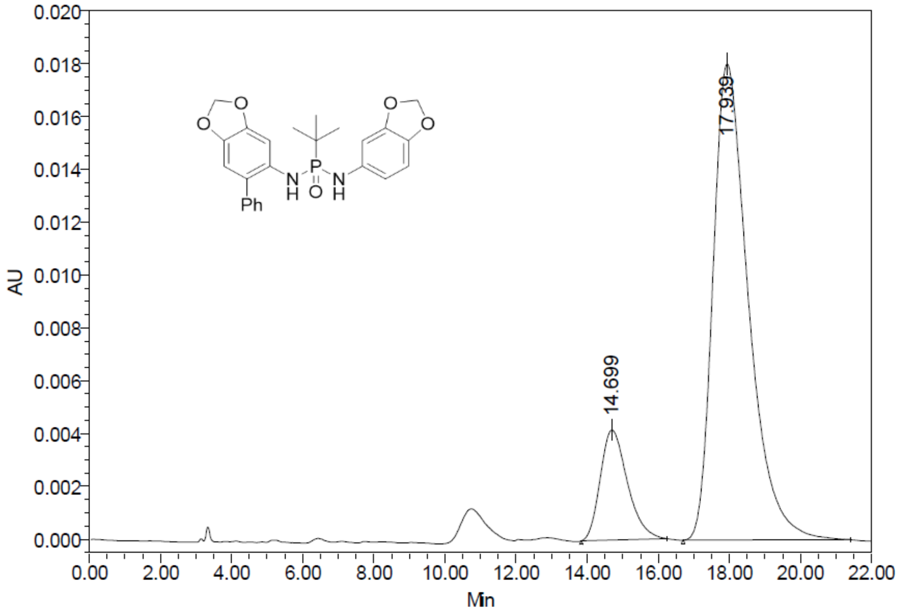

Peak Table

W2489 CHA 254

	Ret.Time	Height	Area	Area%
1	14.699	4169	221255	15.052
2	17.939	18028	1248706	84.948

Supplementary Figure 252. b7: OD-H, Hexane / *i*PrOH = 80/20, rate = 1.0 mL/min, 254 nm

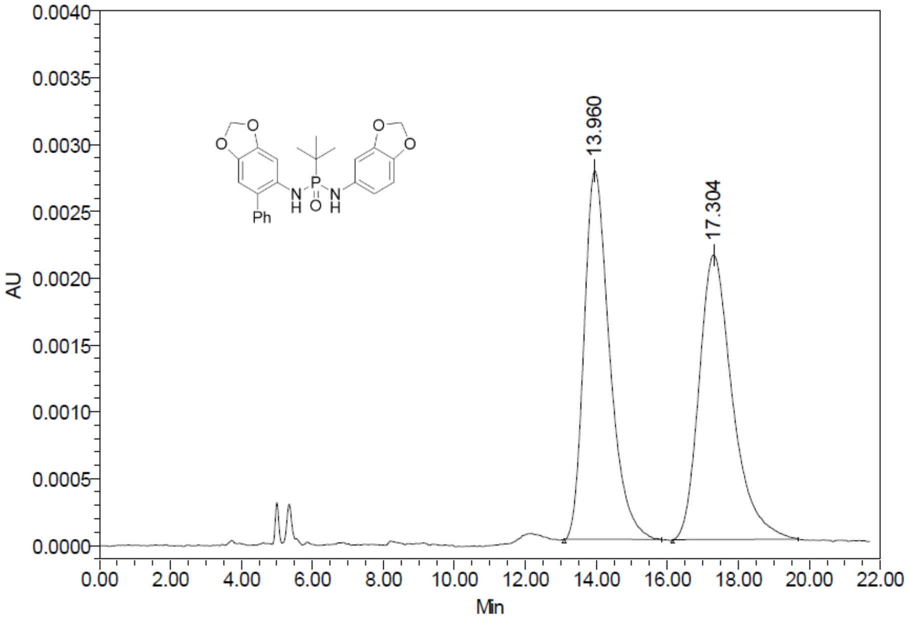

Peak Table

W2489 CHA 254

	Ret.Time	Height	Area	Area%
1	13.960	2759	137644	49.749
2	17.304	2130	139034	50.251

Supplementary Figure 253. rac-b7: OD-H, Hexane / *i*PrOH = 80/20, rate = 1.0 mL/min, 254 nm

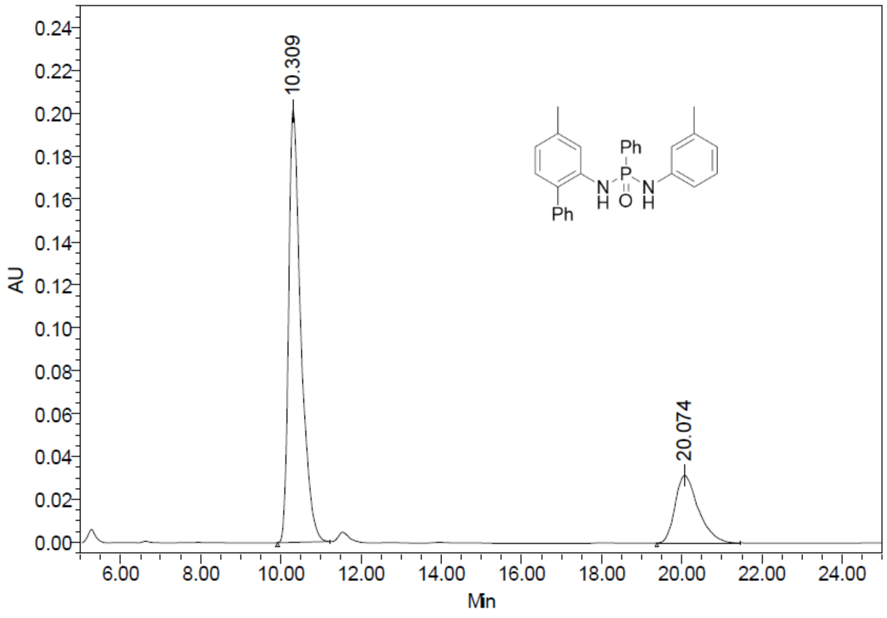

Peak Table

W2489 CHA 254

	Ret.Time	Height	Area	Area%
1	10.309	201894	4286343	77.140
2	20.074	31589	1270221	22.860

Supplementary Figure 254. b8: AD-H, Hexane / *i*PrOH = 80/20, rate = 1.0 mL/min, 254 nm

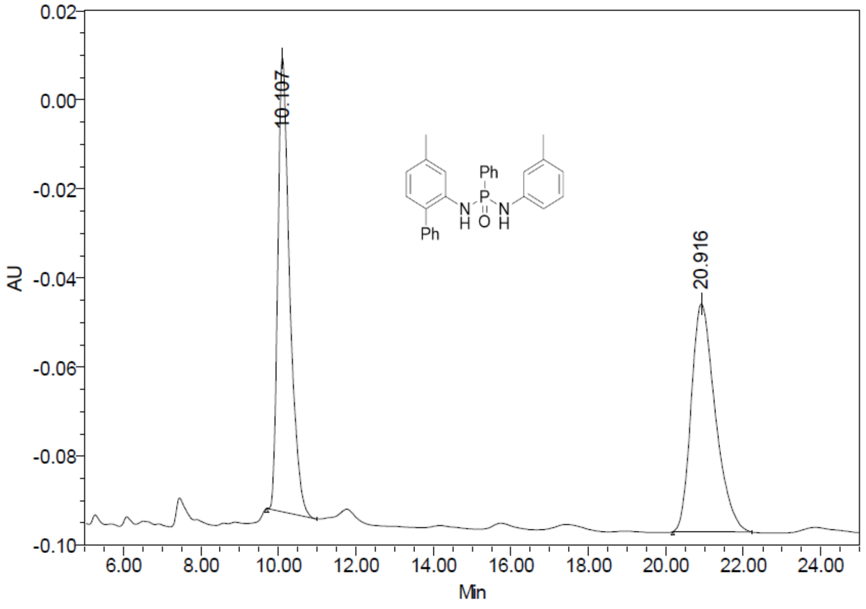

Peak Table

W2489 CHA 254

	Ret.Time	Height	Area	Area%
1	10.107	102117	2174172	49.551
2	20.916	51166	2213547	50.449

Supplementary Figure 255. rac-b8: AD-H, Hexane / *i*PrOH = 80/20, rate = 1.0 mL/min, 254 nm

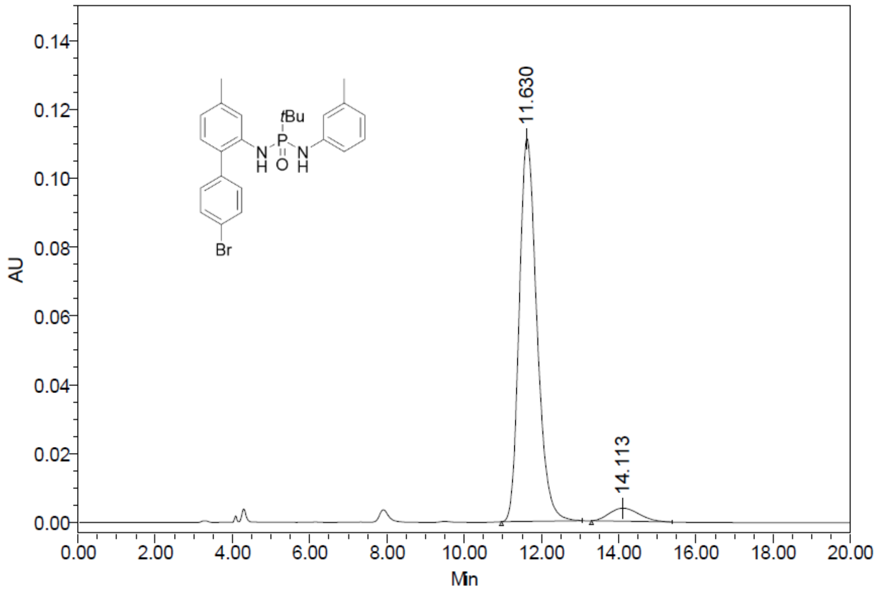

Peak Table

W2489 CHA 254

	Ret.Time	Height	Area	Area%
1	11.630	111193	3533369	94.517
2	14.113	3806	204978	5.483

Supplementary Figure 256. b9: OD-H, Hexane / *i*PrOH = 90/10, rate = 1.0 mL/min, 254 nm

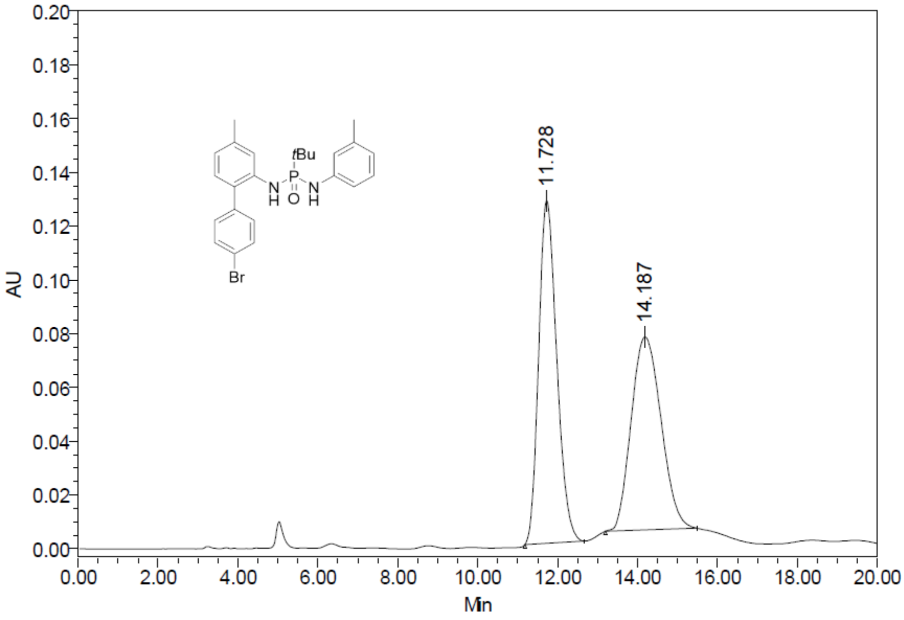

Peak Table

W2489 CHA 254

	Ret.Time	Height	Area	Area%
1	11.728	127427	4005813	51.789
2	14.187	71830	3729022	48.211

Supplementary Figure 257. rac-b9: OD-H, Hexane / *i*PrOH = 90/10, rate = 1.0 mL/min, 254 nm

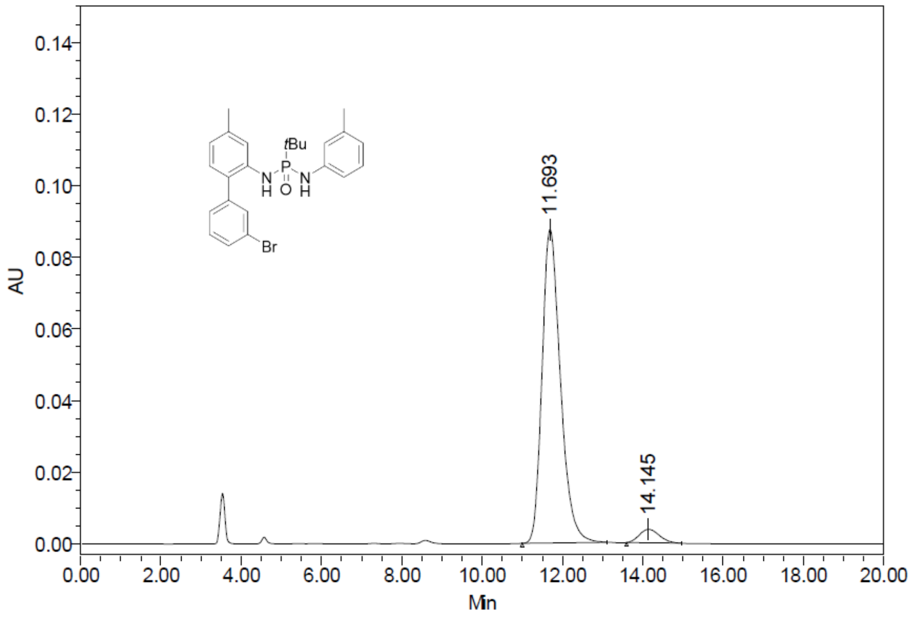

Peak Table

W2489 CHA 254

	Ret.Time	Height	Area	Area%
1	11.693	87537	2785091	95.546
2	14.145	3753	129830	4.454

Supplementary Figure 258. b10: OD-H, Hexane/*i*PrOH = 90/10, rate = 1.0 mL/min, 254 nm

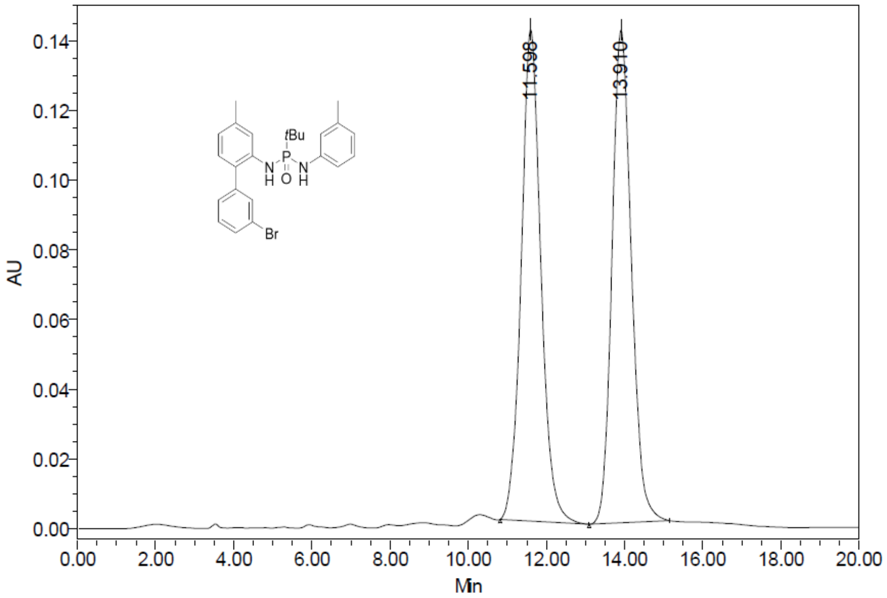

Peak Table

W2489 CHA 254

	Ret.Time	Height	Area	Area%
1	11.598	141044	4916879	50.323
2	13.910	141439	4853832	49.677

Supplementary Figure 259. rac-b10: OD-H, Hexane/*i*PrOH = 90/10, rate = 1.0 mL/min, 254 nm

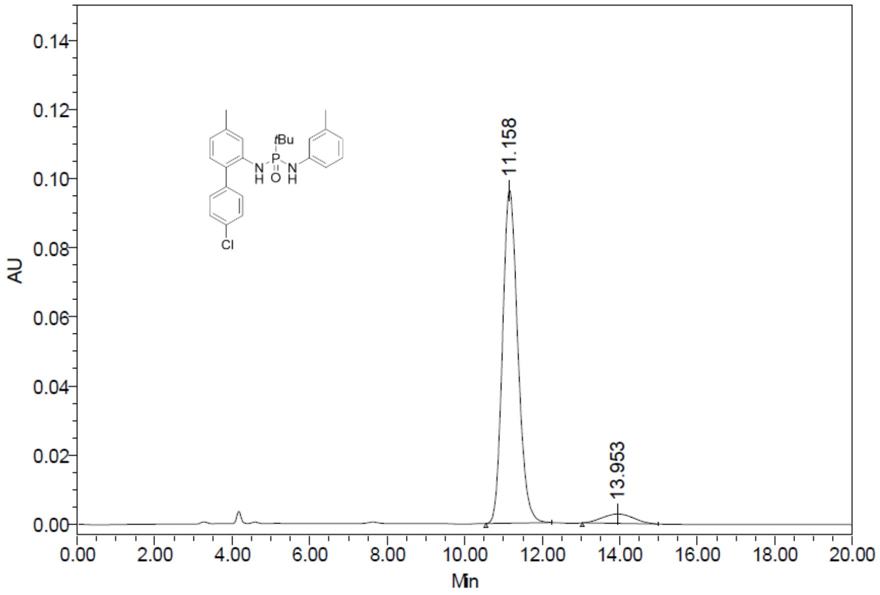

Peak Table

W2489 CHA 254

	Ret.Time	Height	Area	Area%
1	11.158	96268	2639436	94.566
2	13.953	2700	151669	5.434

Supplementary Figure 260. b11: OD-H, Hexane/*i*PrOH = 90/10, rate = 1.0 mL/min, 254 nm

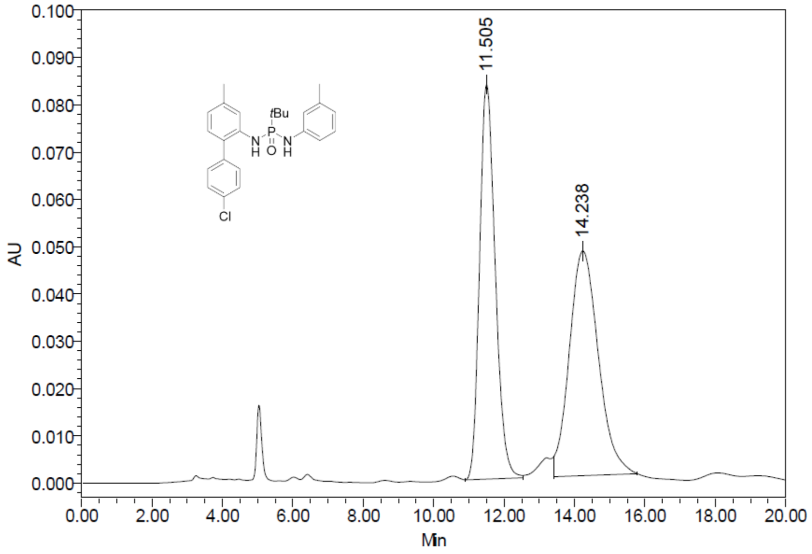

Peak Table

W2489 CHA 254

	Ret.Time	Height	Area	Area%
1	11.505	83454	2579989	48.683
2	14.238	47534	2719560	51.317

Supplementary Figure 261. rac-b11: OD-H, Hexane/*i*PrOH = 90/10, rate = 1.0 mL/min, 254 nm

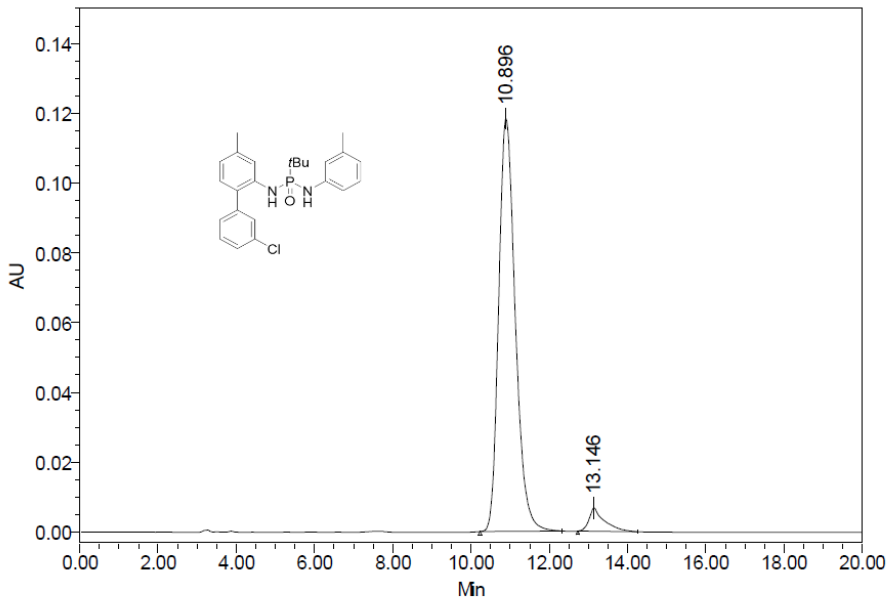

Peak Table

W2489 CHA 254

	Ret.Time	Height	Area	Area%
1	10.896	118268	3485639	95.103
2	13.146	6693	179477	4.897

Supplementary Figure 262. b12: OD-H, Hexane/*i*PrOH = 90/10, rate = 1.0 mL/min, 254 nm

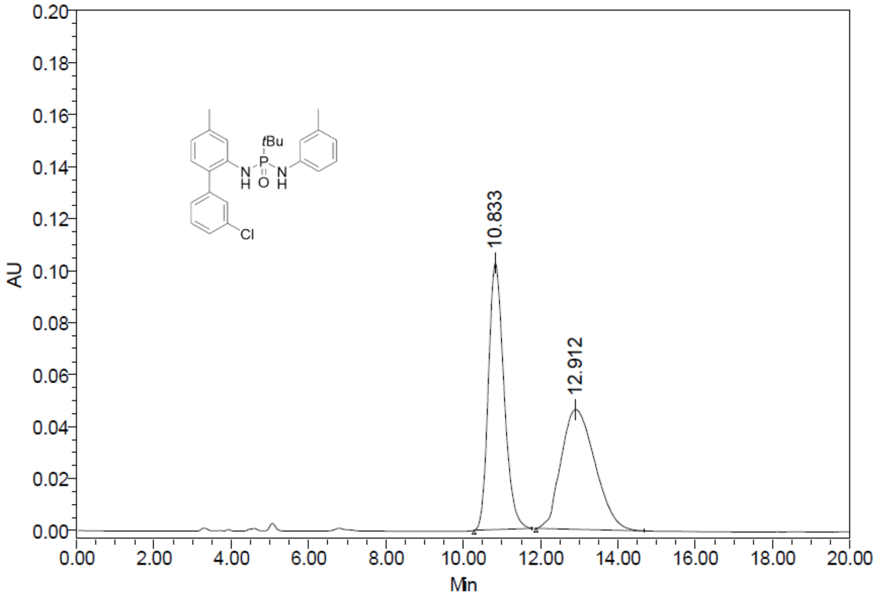

Peak Table

W2489 CHA 254

	Ret.Time	Height	Area	Area%
1	10.833	102498	2861584	50.464
2	12.912	46074	2808995	49.536

Supplementary Figure 263. rac-b12: OD-H, Hexane/*i*PrOH = 90/10, rate = 1.0 mL/min, 254 nm

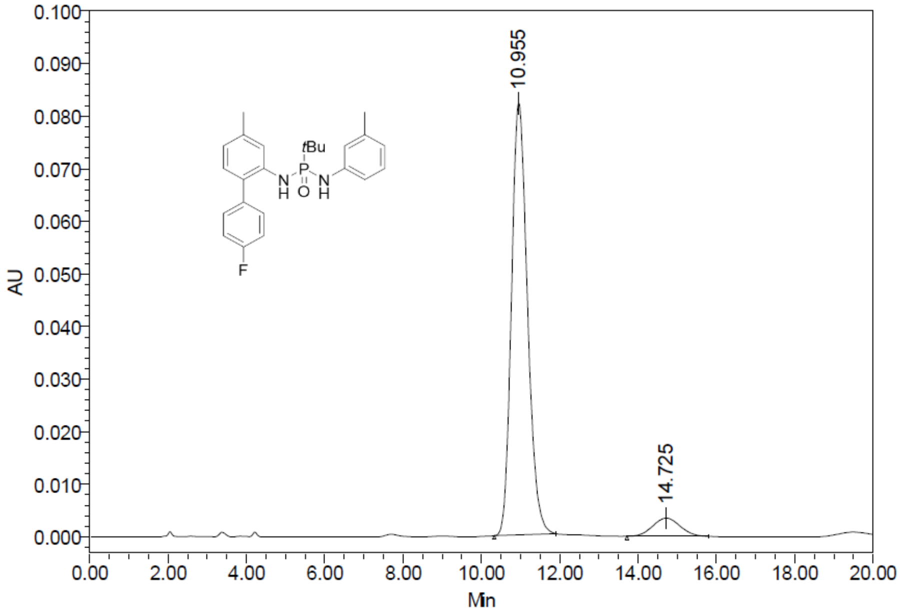

Peak Table

W2489 CHA 254

	Ret.Time	Height	Area	Area%
1	10.955	82153	2333263	93.289
2	14.725	3448	167838	6.711

Supplementary Figure 264. b13: OD-H, Hexane/*i*PrOH = 90/10, rate = 1.0 mL/min, 254 nm

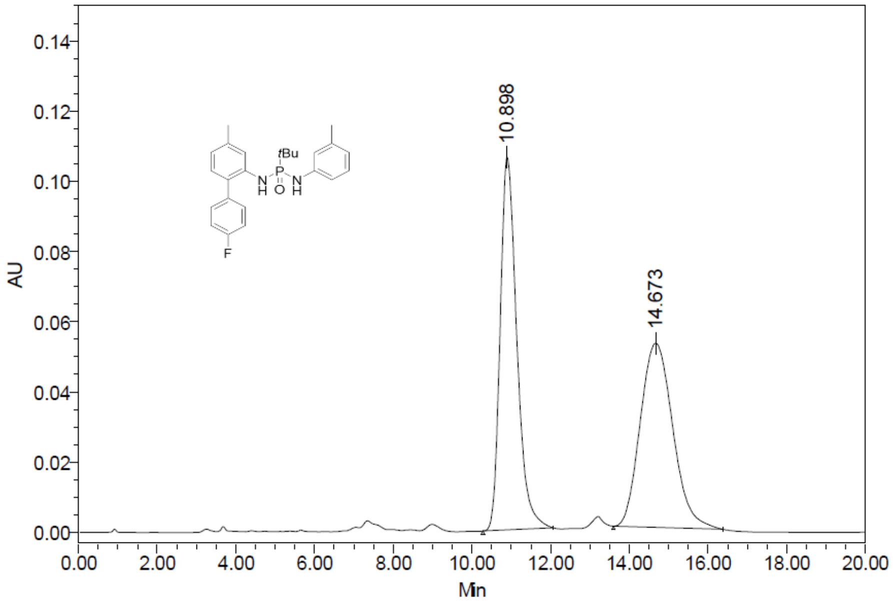

Peak Table

W2489 CHA 254

	Ret.Time	Height	Area	Area%
1	10.898	106186	3109199	50.717
2	14.673	52473	3021271	49.283

Supplementary Figure 265. rac-b13: OD-H, Hexane/*i*PrOH = 90/10, rate = 1.0 mL/min, 254 nm

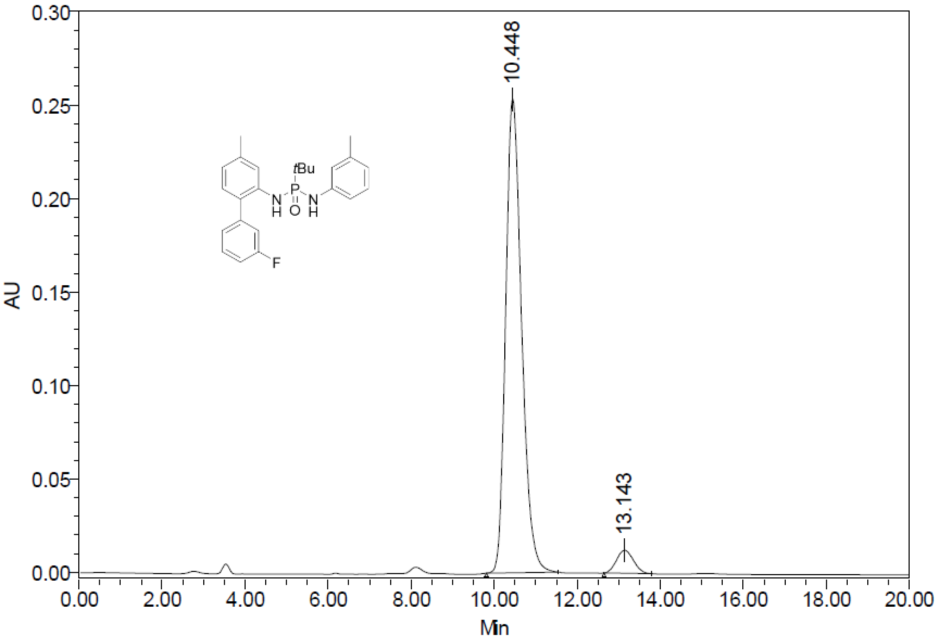

Peak Table

W2489 CHA 254

	Ret.Time	Height	Area	Area%
1	10.448	253270	6757437	95.060
2	13.143	12321	351151	4.940

Supplementary Figure 266. b14: OD-H, Hexane/*i*PrOH = 90/10, rate = 1.0 mL/min, 254 nm

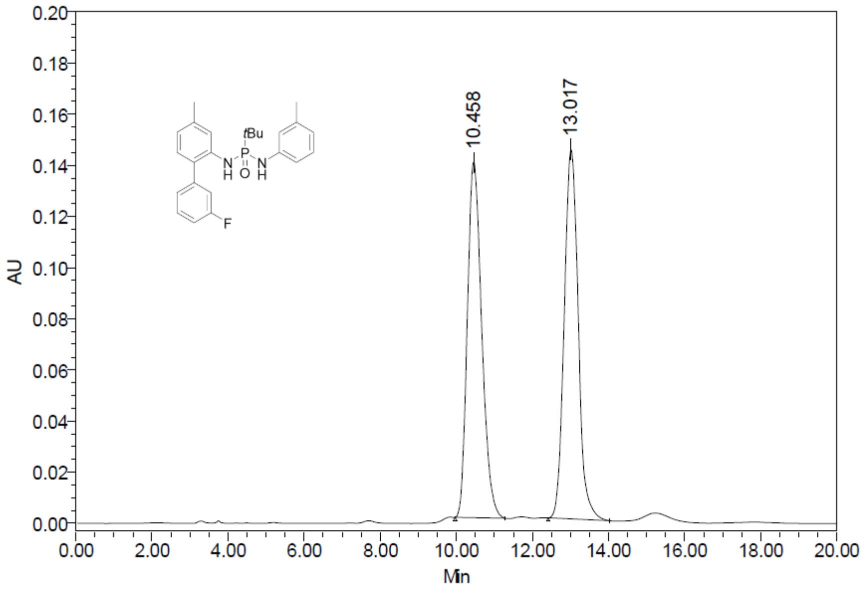

Peak Table

W2489 CHA 254

	Ret.Time	Height	Area	Area%
1	10.458	139132	3659429	49.808
2	13.017	144521	3687684	50.192

Supplementary Figure 267. rac-b14: OD-H, Hexane/*i*PrOH = 90/10, rate = 1.0 mL/min, 254 nm

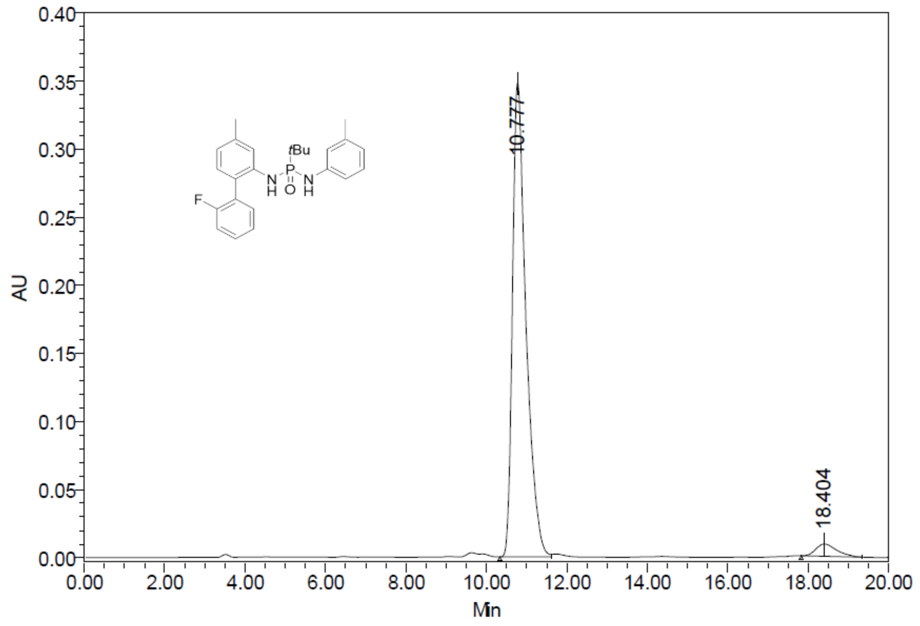

Peak Table

W2489 CHA 254

	Ret.Time	Height	Area	Area%
1	10.777	348245	8106838	96.126
2	18.404	9044	326734	3.874

Supplementary Figure 268. b15: AD-H, Hexane/*i*PrOH = 90/10, rate = 1.0 mL/min, 254 nm

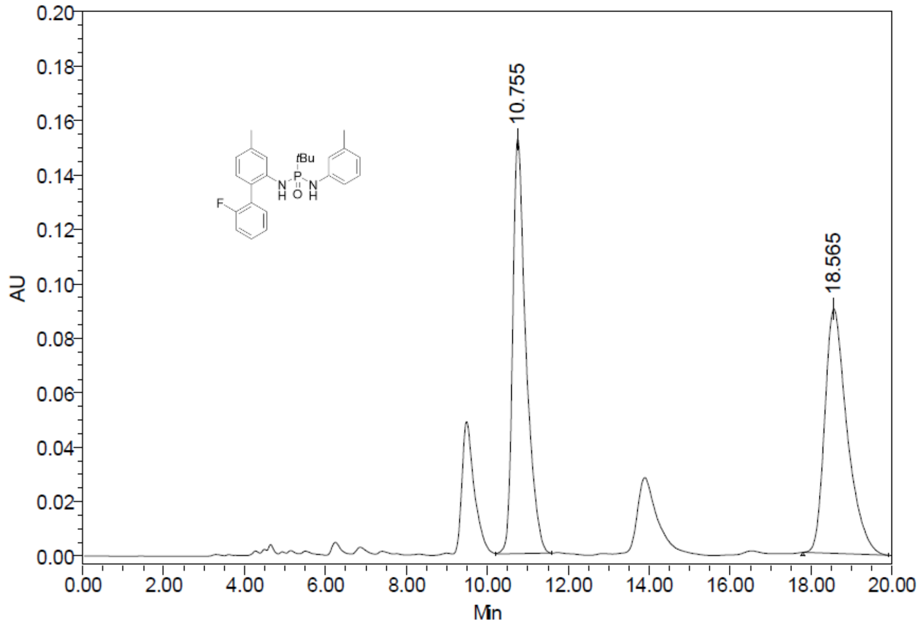

Peak Table

W2489 CHA 254

	Ret.Time	Height	Area	Area%
1	10.755	152585	3497086	50.204
2	18.565	89833	3468599	49.796

Supplementary Figure 269. rac-b15: AD-H, Hexane/*i*PrOH = 90/10, rate = 1.0 mL/min, 254 nm

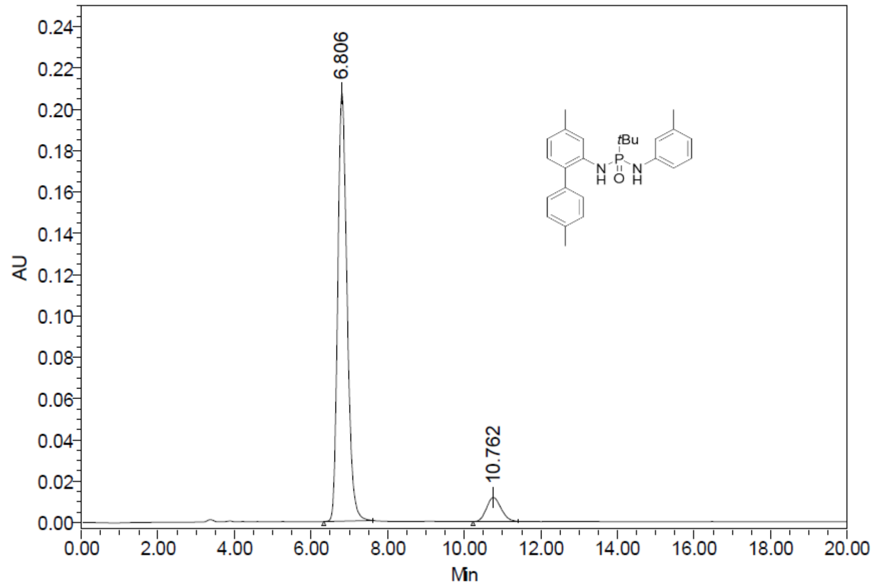

Peak Table

W2489 CHA 254

	Ret.Time	Height	Area	Area%
1	6.806	207756	3510826	91.962
2	10.762	11596	306887	8.038

Supplementary Figure 270. b16: OD-H, Hexane/*i*PrOH = 90/10, rate = 1.0 mL/min, 254 nm

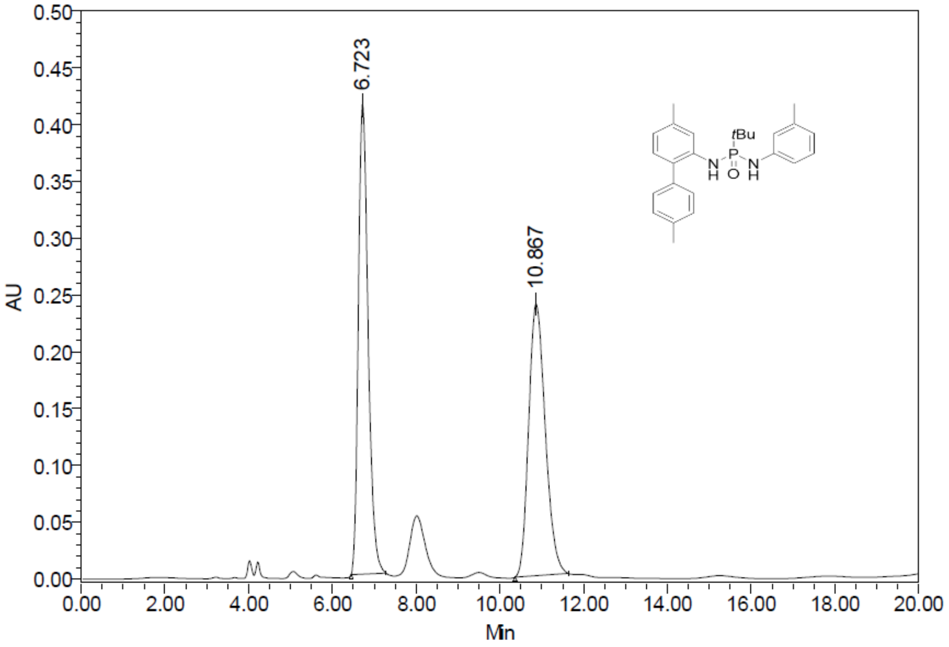

Peak Table

W2489 CHA 254

	Ret.Time	Height	Area	Area%
1	6.723	414027	6694758	50.429
2	10.867	239184	6580737	49.571

Supplementary Figure 271. rac-b16: OD-H, Hexane/*i*PrOH = 90/10, rate = 1.0 mL/min, 254 nm

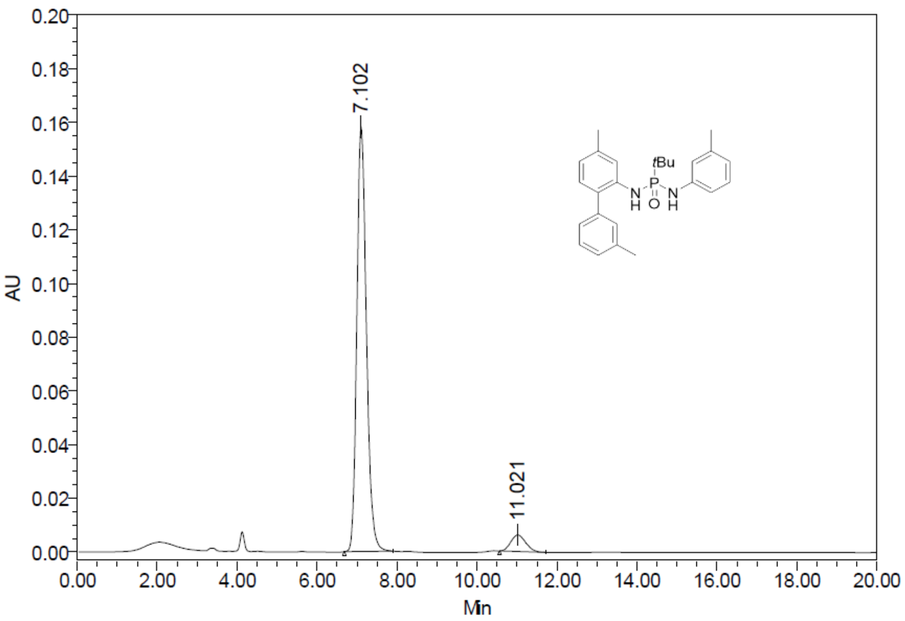

Peak Table

W2489 CHA 254

	Ret. Time	Height	Area	Area%
1	7.102	158374	2633107	94.252
2	11.021	6165	160581	5.748

Supplementary Figure 272. b17: OD-H, Hexane/*i*PrOH = 90/10, rate = 1.0 mL/min, 254 nm

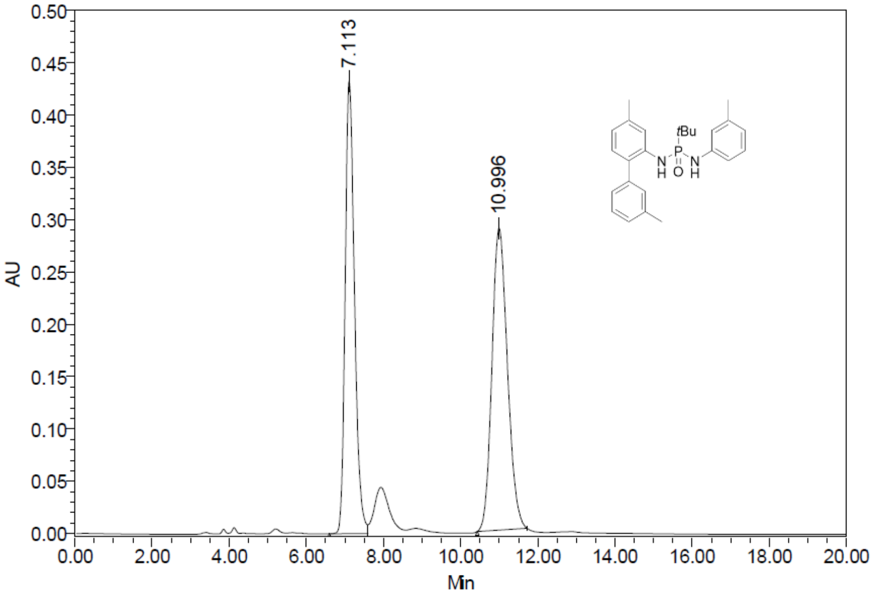

Peak Table

W2489 CHA 254

	Ret. Time	Height	Area	Area%
1	7.113	433198	7281990	47.540
2	10.996	288711	8035493	52.460

Supplementary Figure 273. rac-b17: OD-H, Hexane/*i*PrOH = 90/10, rate = 1.0 mL/min, 254 nm

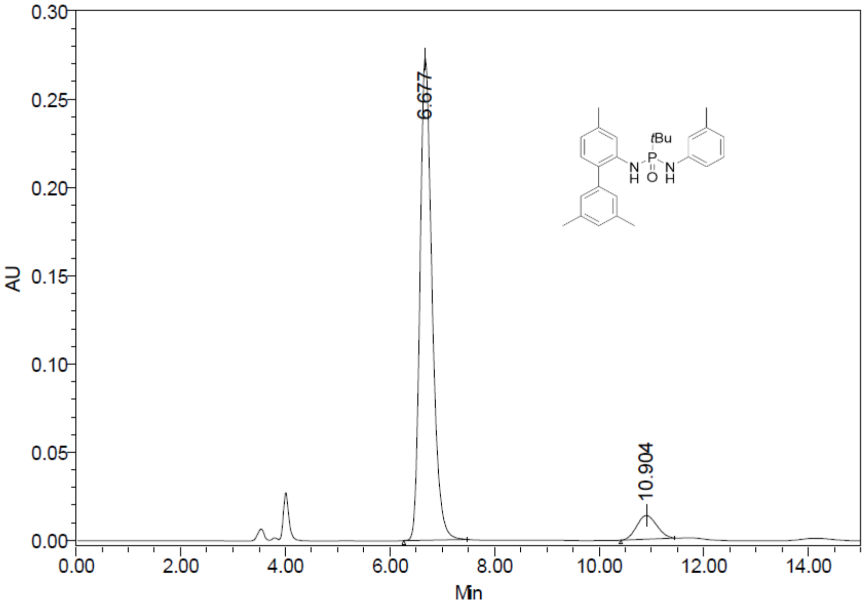

Peak Table

W2489 CHA 254

	Ret.Time	Height	Area	Area%
1	6.677	273191	4386796	92.493
2	10.904	13382	356048	7.507

Supplementary Figure 274. b18: OD-H, Hexane/*i*PrOH = 90/10, rate = 1.0 mL/min, 254 nm

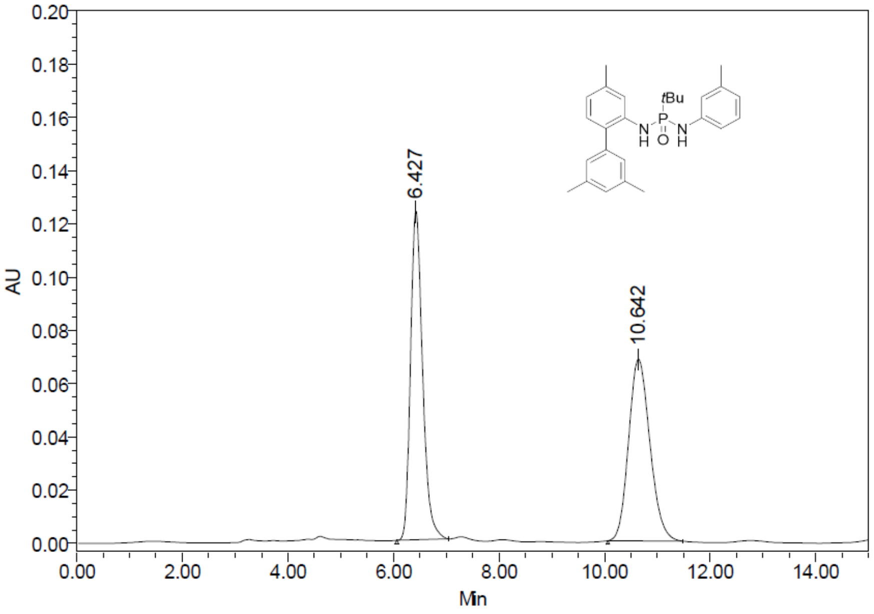

Peak Table

W2489 CHA 254

	Ret.Time	Height	Area	Area%
1	6.427	123470	1951615	50.470
2	10.642	68290	1915261	49.530

Supplementary Figure 275. rac-b18: OD-H, Hexane/*i*PrOH = 90/10, rate = 1.0 mL/min, 254 nm

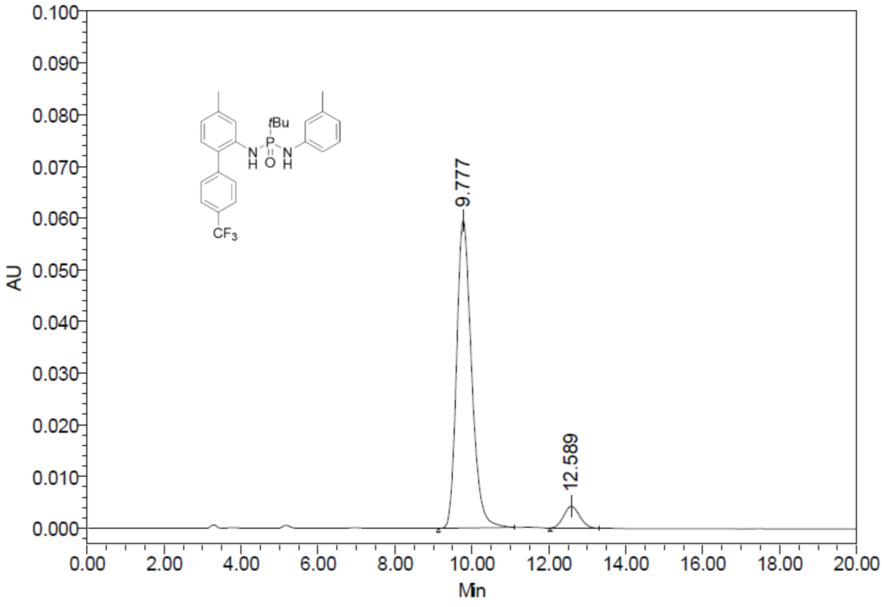

Peak Table

W2489 CHA 254

	Ret.Time	Height	Area	Area%
1	9.777	59525	1603672	92.885
2	12.589	4215	122842	7.115

Supplementary Figure 276. b19: OD-H, Hexane/*i*PrOH = 90/10, rate = 1.0 mL/min, 254 nm

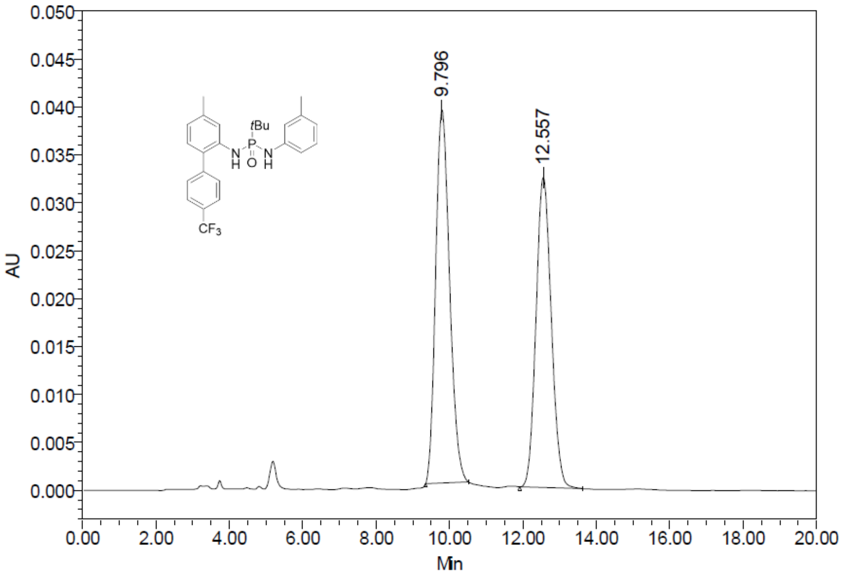

Peak Table

W2489 CHA 254

	Ret.Time	Height	Area	Area%
1	9.796	38953	1029448	52.450
2	12.557	32349	933284	47.550

Supplementary Figure 277. rac-b19: OD-H, Hexane/*i*PrOH = 90/10, rate = 1.0 mL/min, 254 nm

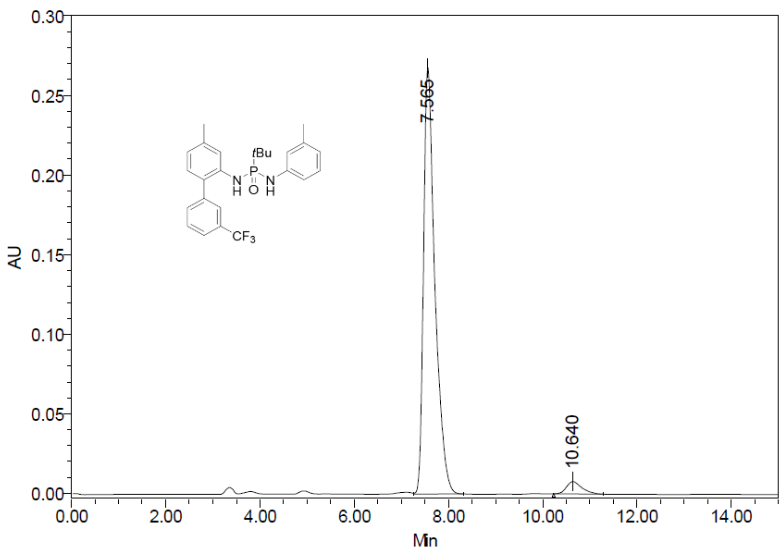

Peak Table

W2489 CHA 254

	Ret.Time	Height	Area	Area%
1	7.565	267774	4477774	96.246
2	10.640	7725	174648	3.754

Supplementary Figure 278. b20: AD-H, Hexane/*i*PrOH = 90/10, rate = 1.0 mL/min, 254 nm

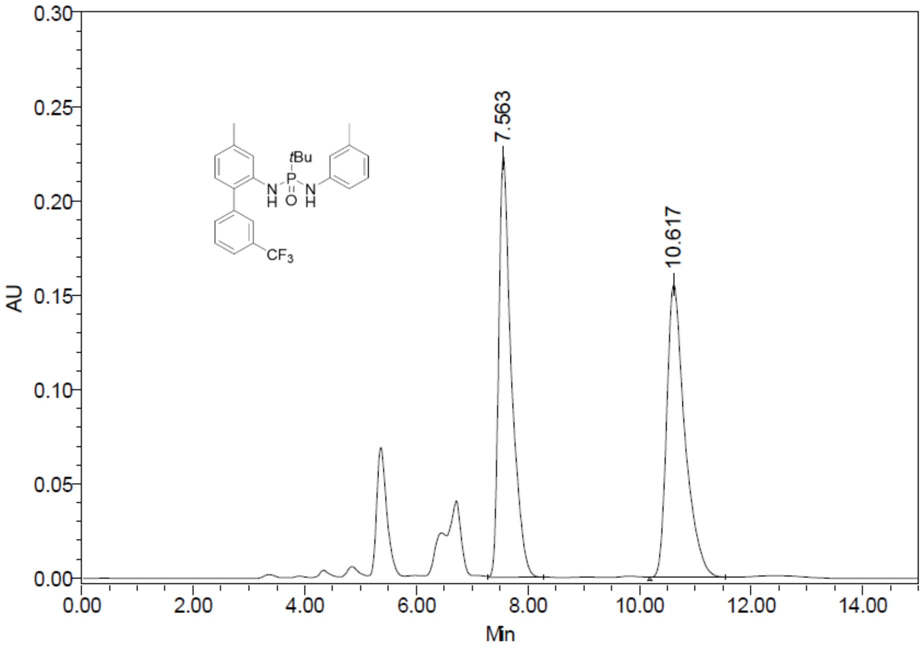

Peak Table

W2489 CHA 254

	Ret.Time	Height	Area	Area%
1	7.563	222676	3537354	50.260
2	10.617	155220	3500707	49.740

Supplementary Figure 279. rac-b20: AD-H, Hexane/*i*PrOH = 90/10, rate = 1.0 mL/min, 254 nm

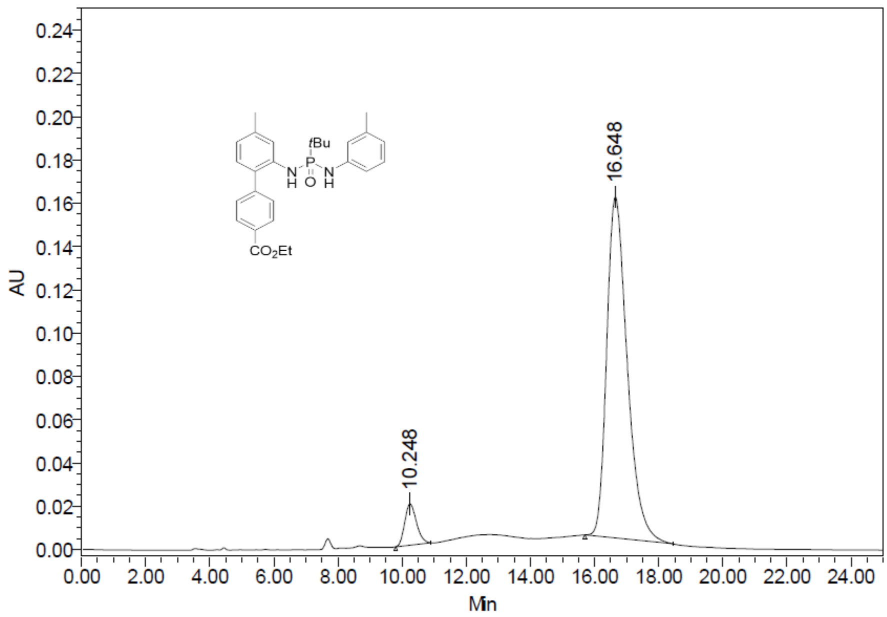

Peak Table

W2489 CHA 254

	Ret.Time	Height	Area	Area%
1	10.248	19040	483976	6.469
2	16.648	157586	6997342	93.531

Supplementary Figure 280. b21: IC, Hexane / *i*PrOH = 80/20, rate = 1.0 mL/min, 254 nm

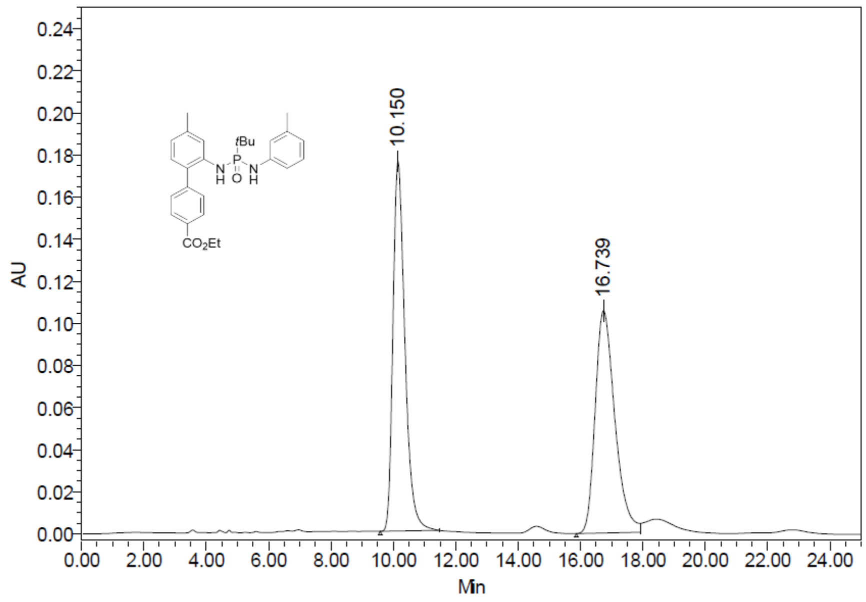

Peak Table

W2489 CHA 254

	Ret.Time	Height	Area	Area%
1	10.150	175695	4664063	50.248
2	16.739	105612	4618003	49.752

Supplementary Figure 281. rac-b21: IC, Hexane / *i*PrOH = 80/20, rate = 1.0 mL/min, 254 nm

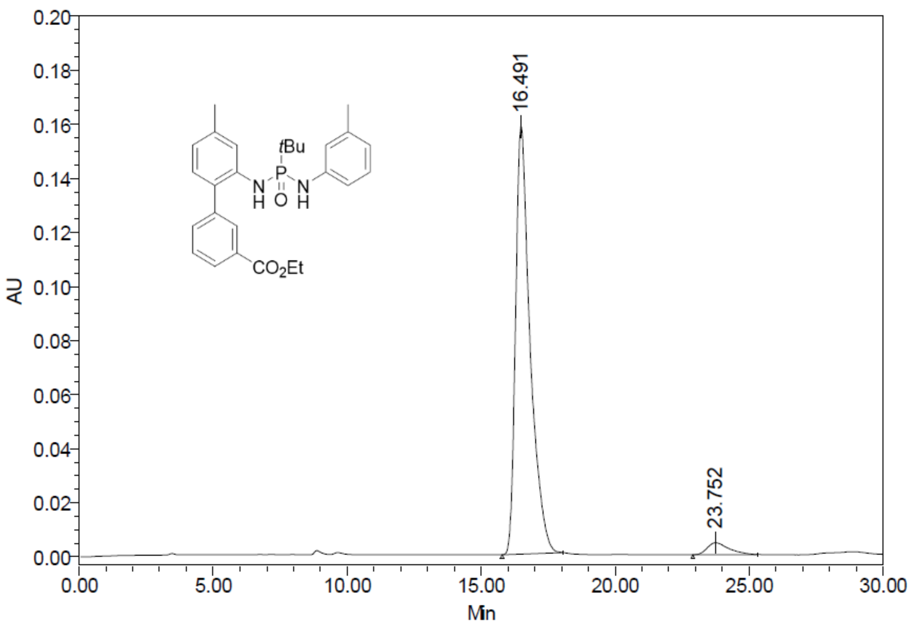

Peak Table

W2489 CHA 254

	Ret.Time	Height	Area	Area%
1	16.491	158303	5974975	95.932
2	23.752	4469	253354	4.068

Supplementary Figure 282. b22: AD-H, Hexane/*i*PrOH = 90/10, rate = 1.0 mL/min, 254 nm

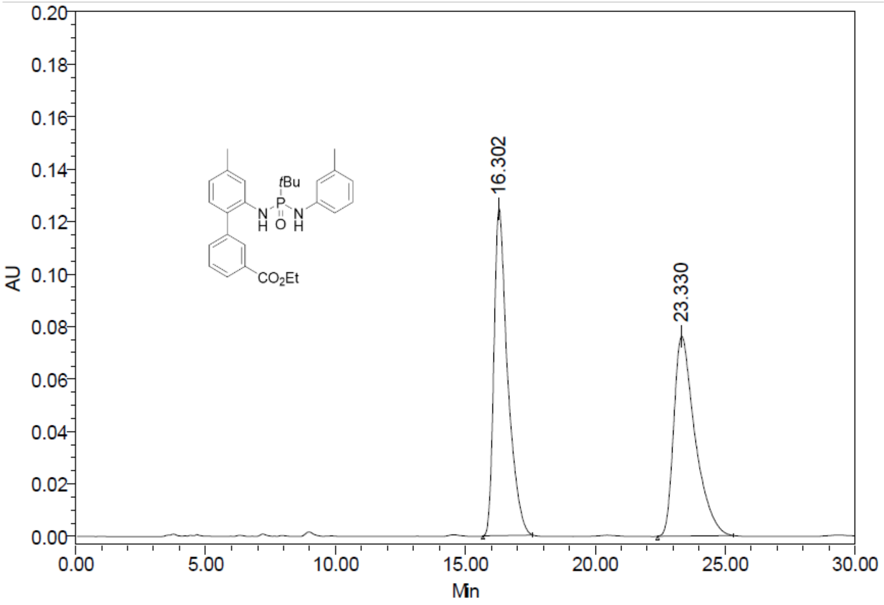

Peak Table

W2489 CHA 254

	Ret.Time	Height	Area	Area%
1	16.302	124631	4583008	51.424
2	23.330	76197	4329232	48.576

Supplementary Figure 283. rac-b22: AD-H, Hexane/*i*PrOH = 90/10, rate = 1.0 mL/min, 254 nm

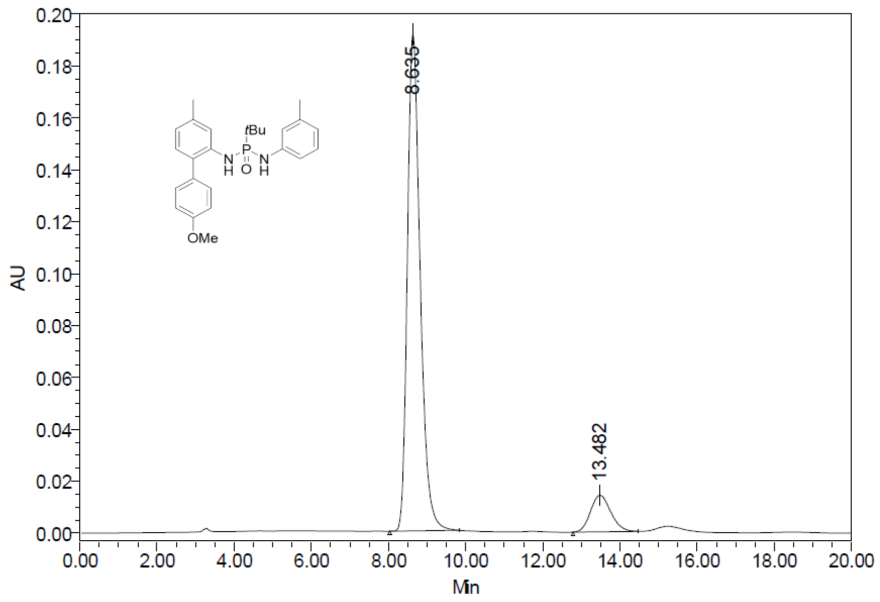

Peak Table

W2489 CHA 254

	Ret.Time	Height	Area	Area%
1	8.635	191409	4410221	89.549
2	13.482	14087	514710	10.451

Supplementary Figure 284. b23: OD-H, Hexane/*i*PrOH = 90/10, rate = 1.0 mL/min, 254 nm

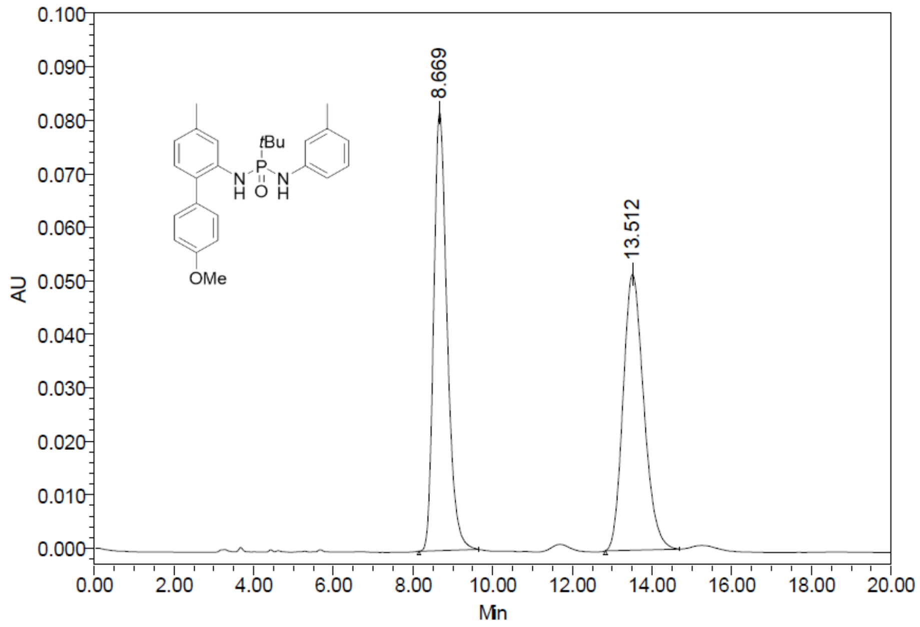

Peak Table

W2489 CHA 254

	Ret.Time	Height	Area	Area%
1	8.669	81924	1918406	50.743
2	13.512	51562	1862193	49.257

Supplementary Figure 285. rac-b23: OD-H, Hexane/*i*PrOH = 90/10, rate = 1.0 mL/min, 254 nm

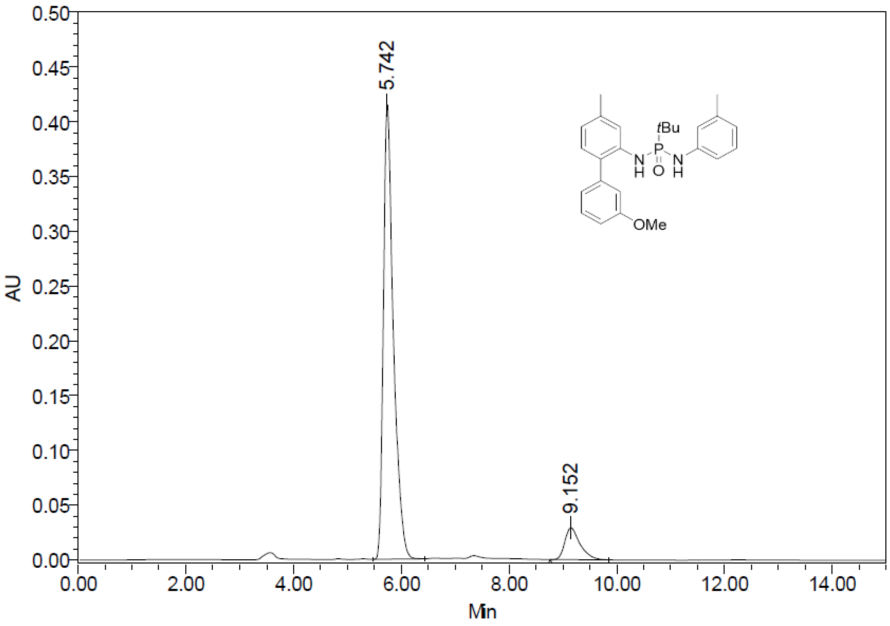

Peak Table

W2489 CHA 254

	Ret. Time	Height	Area	Area%
1	5.742	417624	5362002	90.381
2	9.152	29164	570687	9.619

Supplementary Figure 286. b24: AD-H, Hexane/*i*PrOH = 80/20, rate = 1.0 mL/min, 254 nm

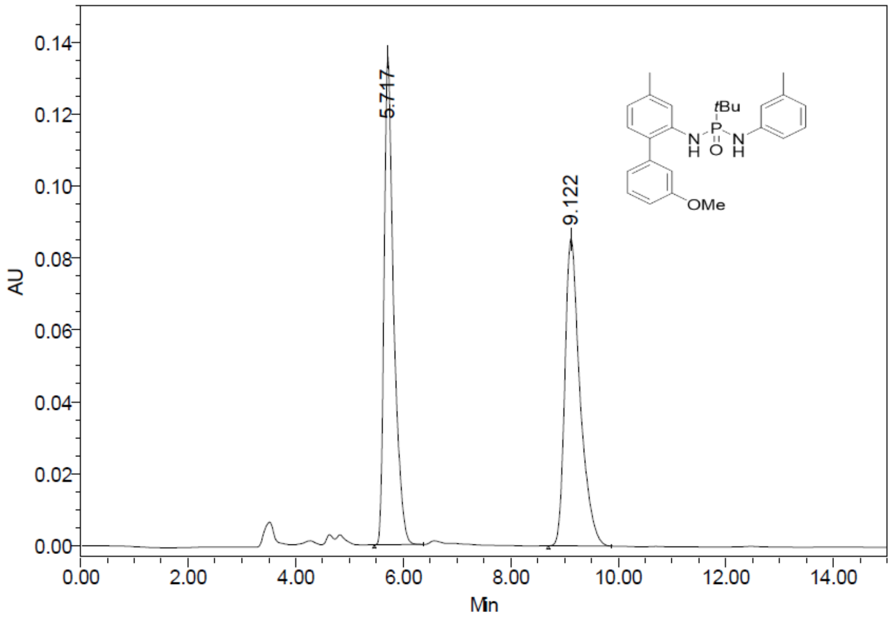

Peak Table

W2489 CHA 254

	Ret. Time	Height	Area	Area%
1	5.717	135500	1676285	49.982
2	9.122	85410	1677488	50.018

Supplementary Figure 287. rac-b24: AD-H, Hexane/*i*PrOH = 80/20, rate = 1.0 mL/min, 254 nm

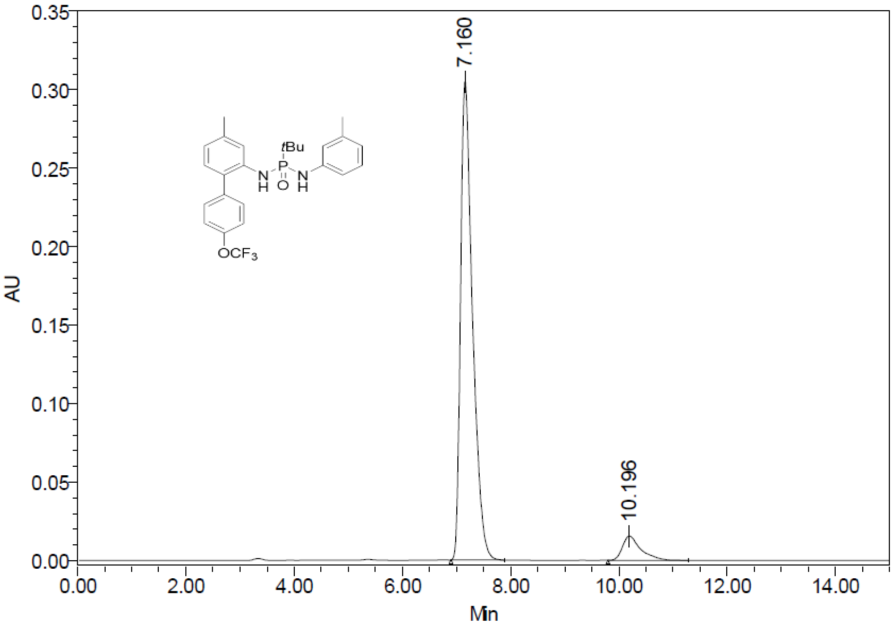

Peak Table

W2489 CHA 254

	Ret.Time	Height	Area	Area%
1	7.160	305409	4677078	92.709
2	10.196	15521	367798	7.291

Supplementary Figure 288. b25: AD-H, Hexane/*i*PrOH = 90/10, rate = 1.0 mL/min, 254 nm

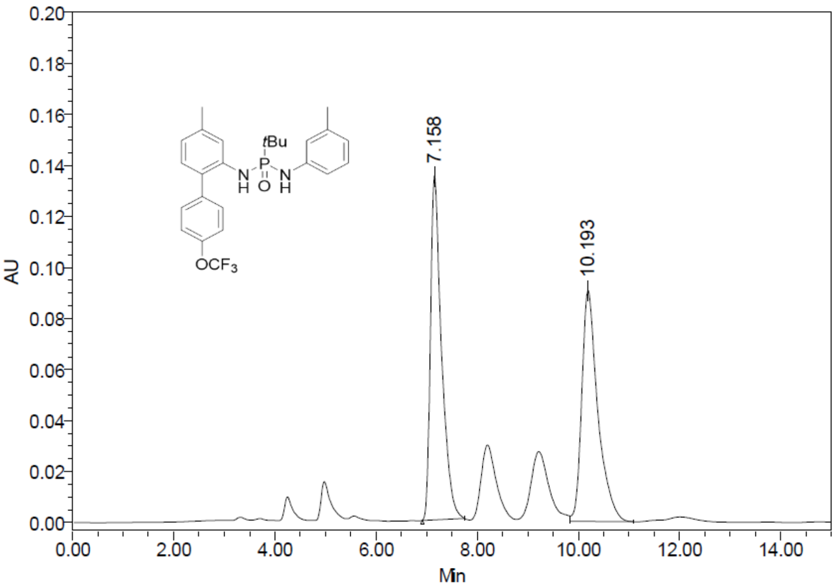

Peak Table

W2489 CHA 254

	Ret.Time	Height	Area	Area%
1	7.158	135239	2045477	50.586
2	10.193	90595	1998124	49.414

Supplementary Figure 289. rac-b25: AD-H, Hexane/*i*PrOH = 90/10, rate = 1.0 mL/min, 254 nm

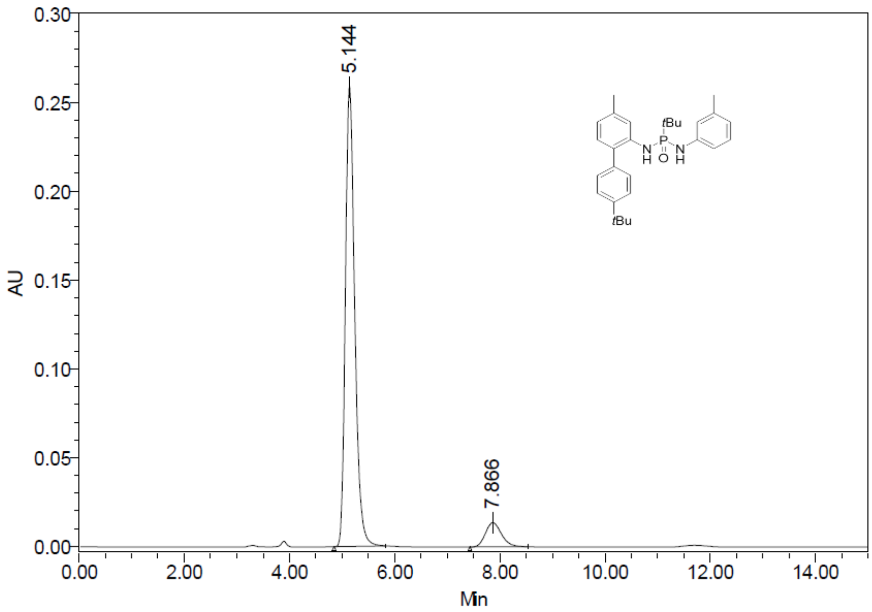

Peak Table

W2489 CHA 254

	Ret.Time	Height	Area	Area%
1	5.144	259310	3189217	91.694
2	7.866	13646	288877	8.306

Supplementary Figure 290. b26: OD-H, Hexane/*i*PrOH = 90/10, rate = 1.0 mL/min, 254 nm

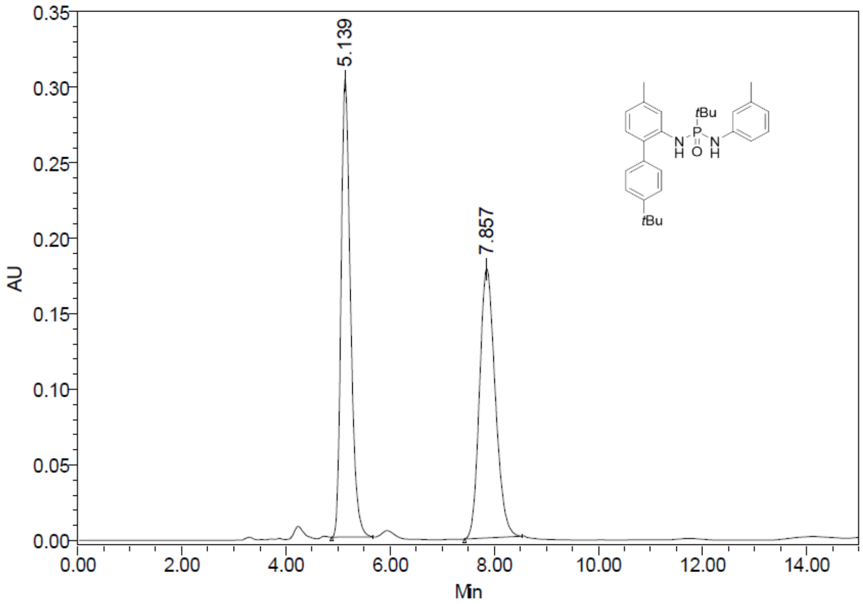

Peak Table

W2489 CHA 254

	Ret.Time	Height	Area	Area%
1	5.139	303609	3820766	50.539
2	7.857	178401	3739278	49.461

Supplementary Figure 291. rac-b26: OD-H, Hexane/*i*PrOH = 90/10, rate = 1.0 mL/min, 254 nm

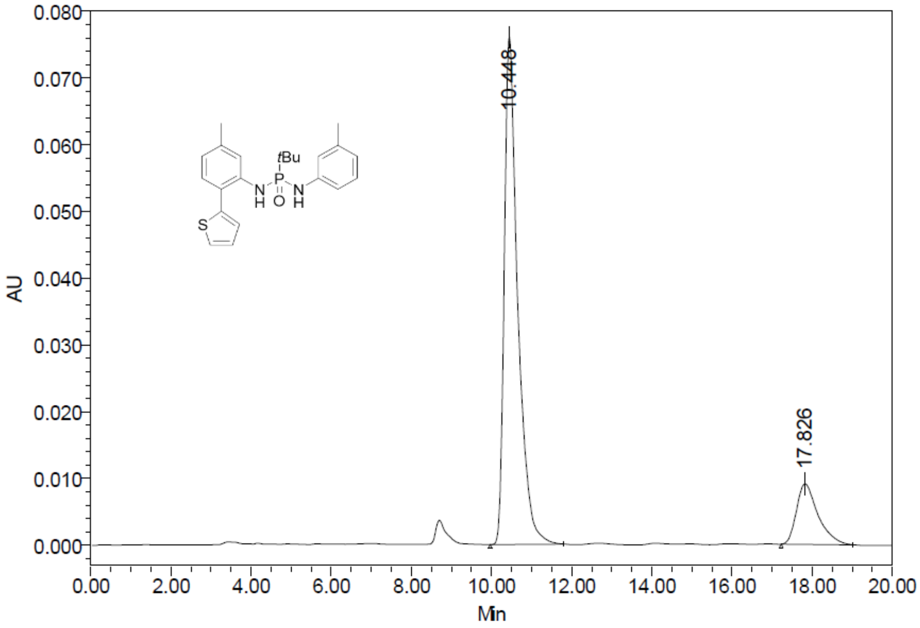

Peak Table

W2489 CHA 254

	Ret.Time	Height	Area	Area%
1	10.448	76046	1769405	83.949
2	17.826	9090	338309	16.051

Supplementary Figure 292. b27: AD-H, Hexane/*i*PrOH = 90/10, rate = 1.0 mL/min, 254 nm

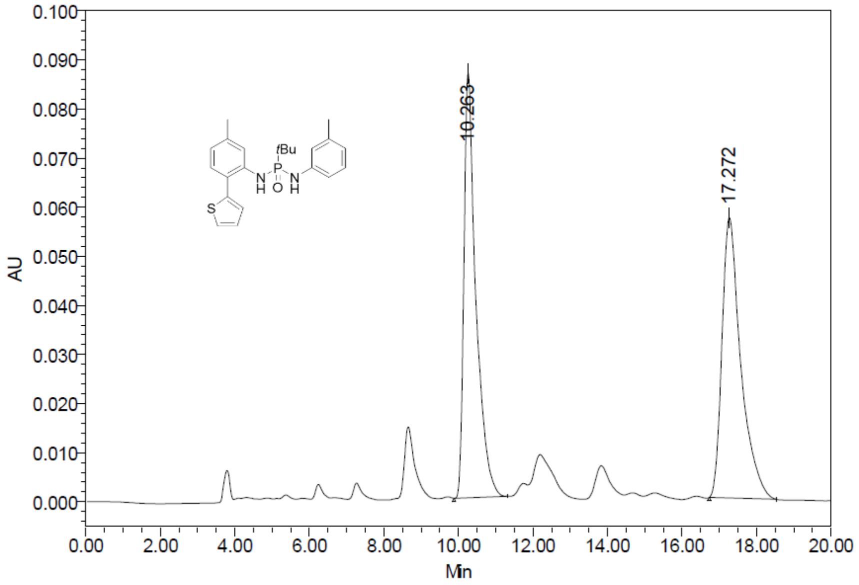

Peak Table

W2489 CHA 254

	Ret.Time	Height	Area	Area%
1	10.263	86560	1932588	49.090
2	17.272	57158	2004241	50.910

Supplementary Figure 293. rac-b27: AD-H, Hexane/*i*PrOH = 90/10, rate = 1.0 mL/min, 254 nm

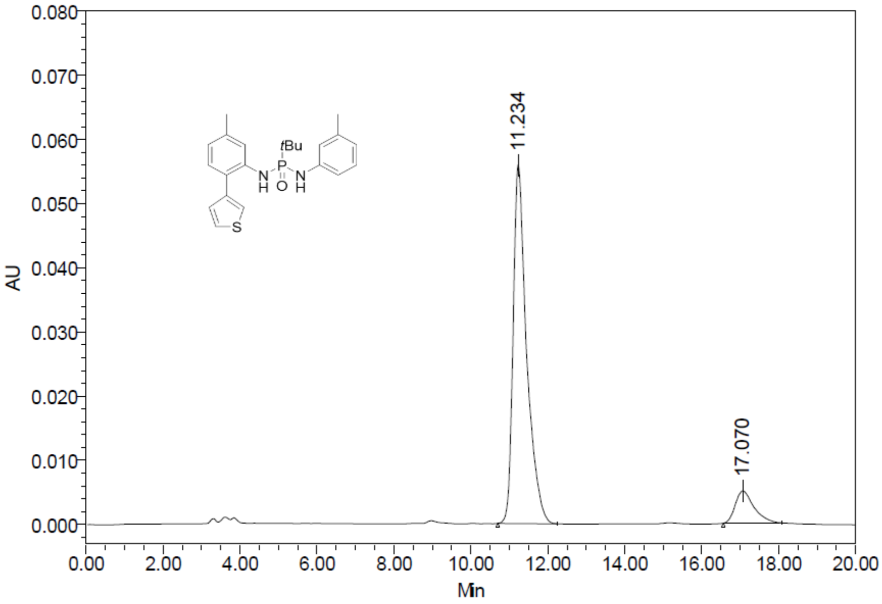

Peak Table

W2489 CHA 254

	Ret.Time	Height	Area	Area%
1	11.234	55907	1365408	88.933
2	17.070	5017	169908	11.067

Supplementary Figure 294. b28: AD-H, Hexane/*i*PrOH = 90/10, rate = 1.0 mL/min, 254 nm

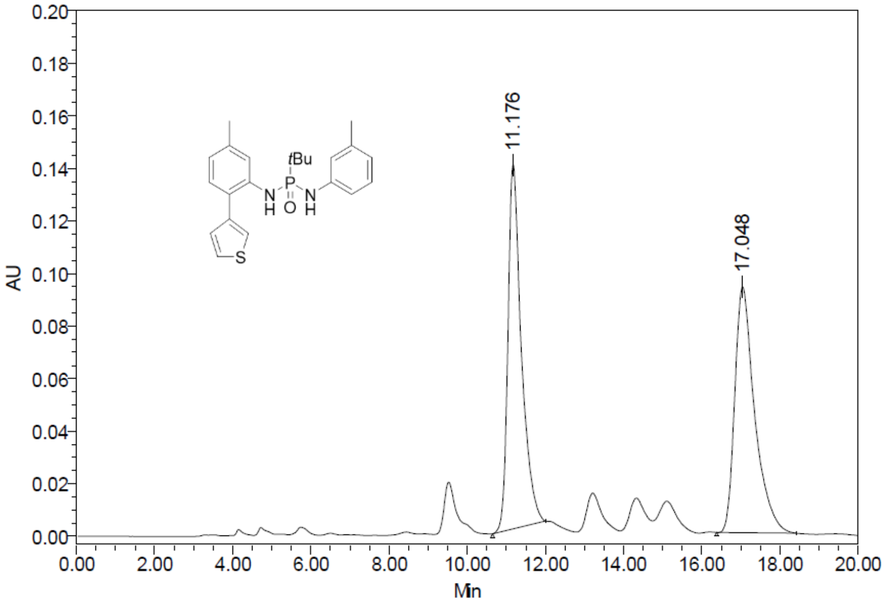

Peak Table

W2489 CHA 254

	Ret.Time	Height	Area	Area%
1	11.176	138611	3319078	50.043
2	17.048	93627	3313391	49.957

Supplementary Figure 295. rac-b28: AD-H, Hexane/*i*PrOH = 90/10, rate = 1.0 mL/min, 254 nm

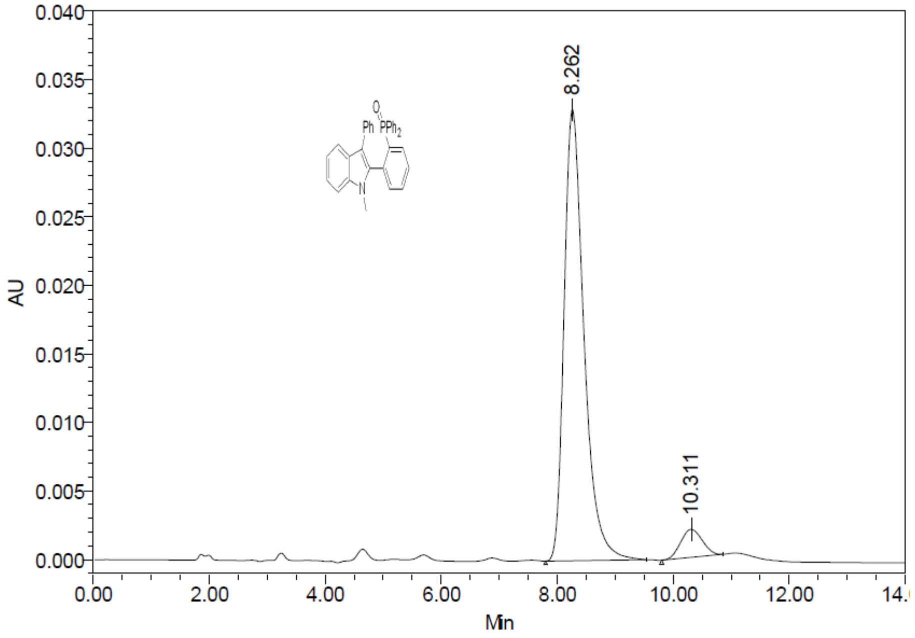

Peak Table

W2489 CHA 254

	Ret.Time	Height	Area	Area%
1	8.262	32940	782672	93.704
2	10.311	2034	52590	6.296

Supplementary Figure 296. d1: OD-H, Hexane/*i*PrOH = 90/10, rate = 1.0 mL/min, 254 nm

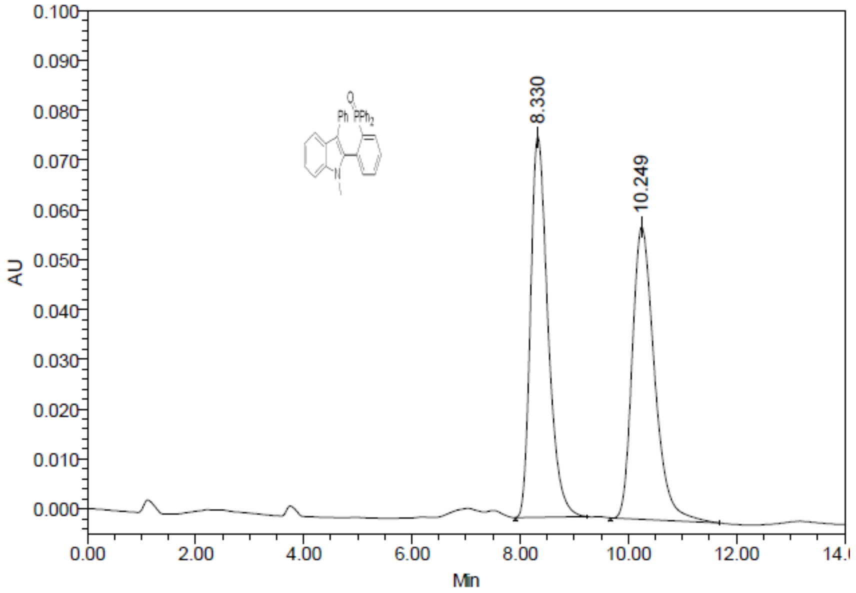

Peak Table

W2489 CHA 254

	Ret.Time	Height	Area	Area%
1	8.330	76371	1710337	50.513
2	10.249	58688	1675608	49.487

Supplementary Figure 297. rac-d1: OD-H, Hexane/*i*PrOH = 90/10, rate = 1.0 mL/min, 254 nm

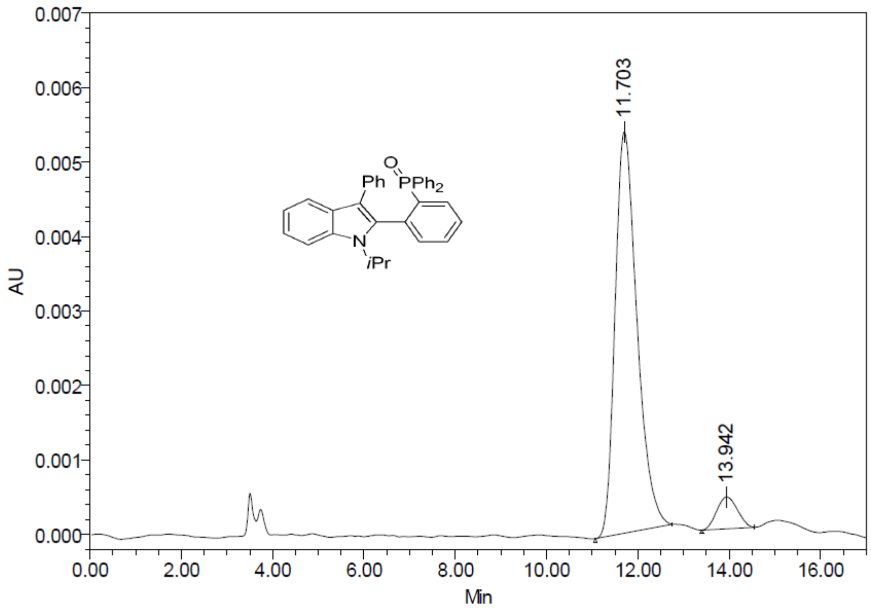

Peak Table

W2489 CHA 254

	Ret.Time	Height	Area	Area%
1	11.703	5389	179445	93.043
2	13.942	427	13417	6.957

Supplementary Figure 298. d2: OD-H, Hexane / *i*PrOH = 97/03, rate = 1.0 mL/min, 254 nm

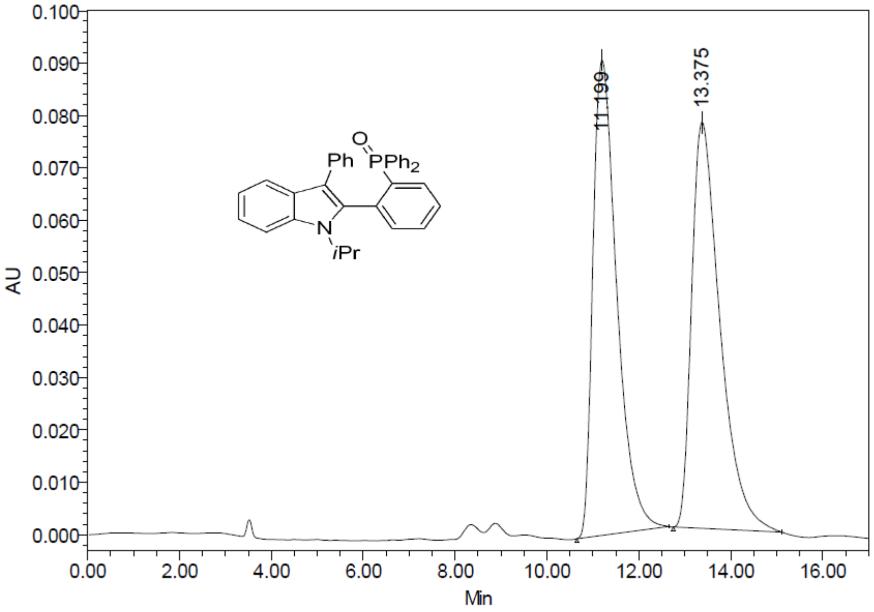

Peak Table

W2489 CHA 254

	Ret.Time	Height	Area	Area%
1	11.199	90698	3277794	49.648
2	13.375	77520	3324289	50.352

Supplementary Figure 299. rac-d2: OD-H, Hexane / *i*PrOH = 97/03, rate = 1.0 mL/min, 254 nm

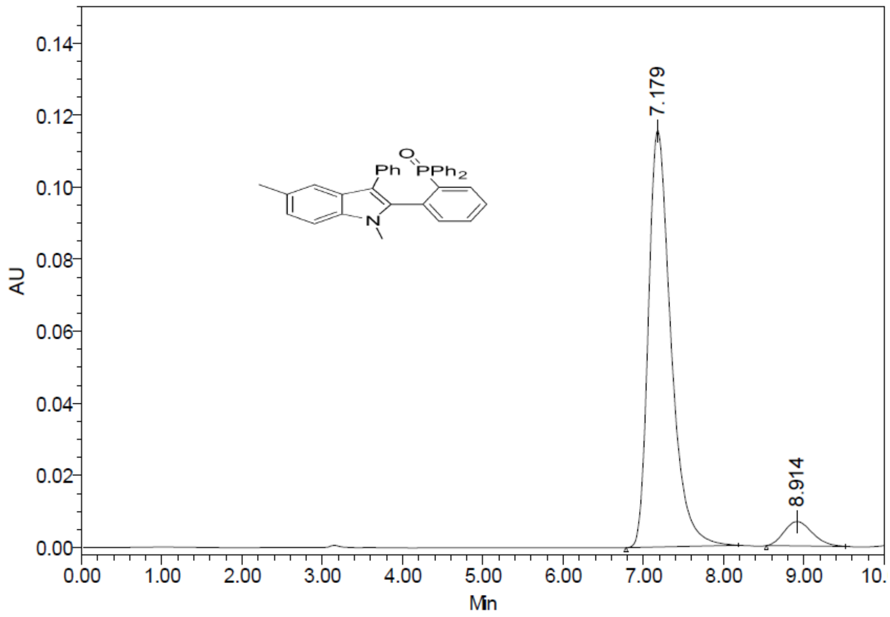

Peak Table

W2489 CHA 254

	Ret.Time	Height	Area	Area%
1	7.179	115610	2246588	93.142
2	8.914	6758	165406	6.858

Supplementary Figure 300. d3: OD-H, Hexane / *i*PrOH = 90/10, rate = 1.0 mL/min, 254 nm

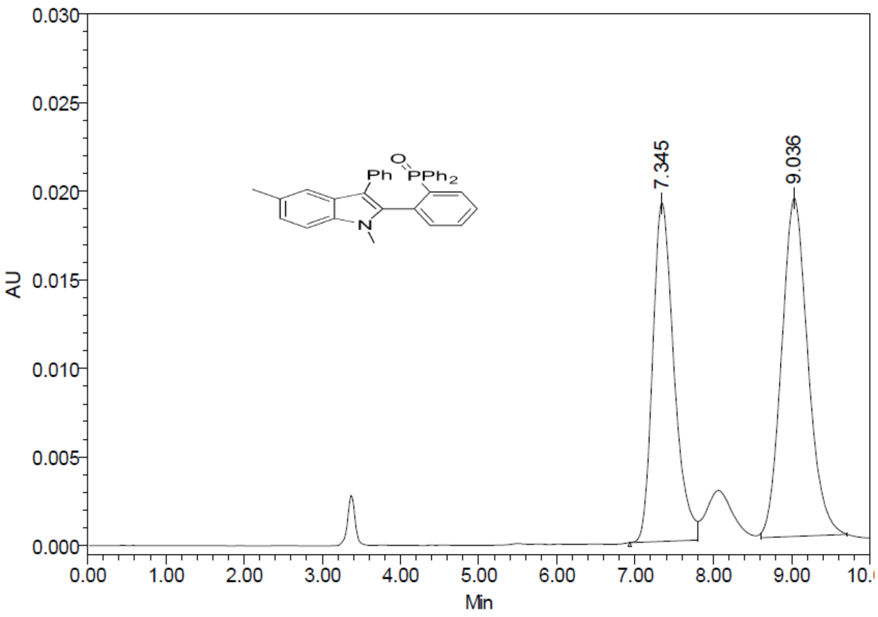

Peak Table

W2489 CHA 254

	Ret.Time	Height	Area	Area%
1	7.345	19089	359930	44.641
2	9.036	19104	446351	55.359

Supplementary Figure 301. rac-d3: OD-H, Hexane / *i*PrOH = 90/10, rate = 1.0 mL/min, 254 nm

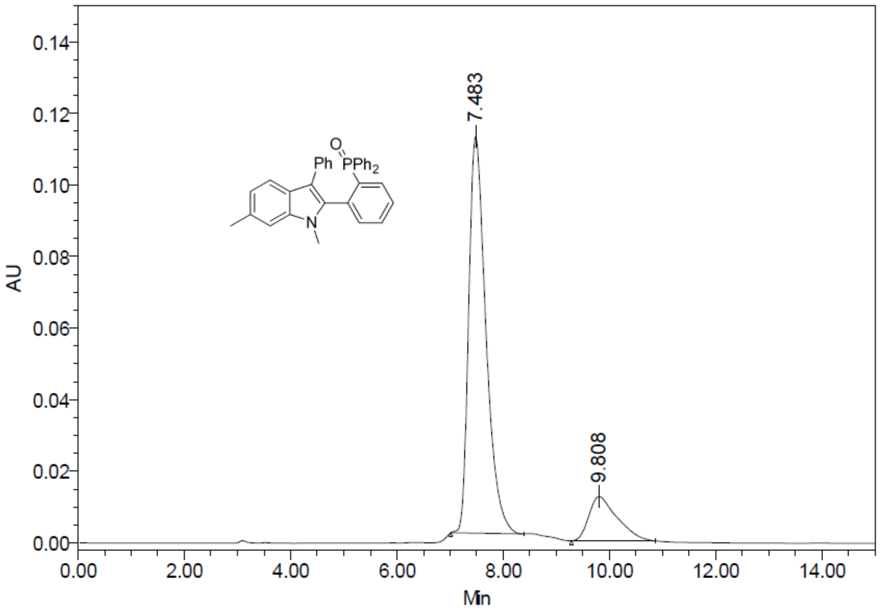

Peak Table

W2489 CHA 254

	Ret.Time	Height	Area	Area%
1	7.483	110872	2493956	84.510
2	9.808	12364	457122	15.490

Supplementary Figure 302. d4: OD-H, Hexane / *i*PrOH = 90/10, rate = 1.0 mL/min, 254 nm

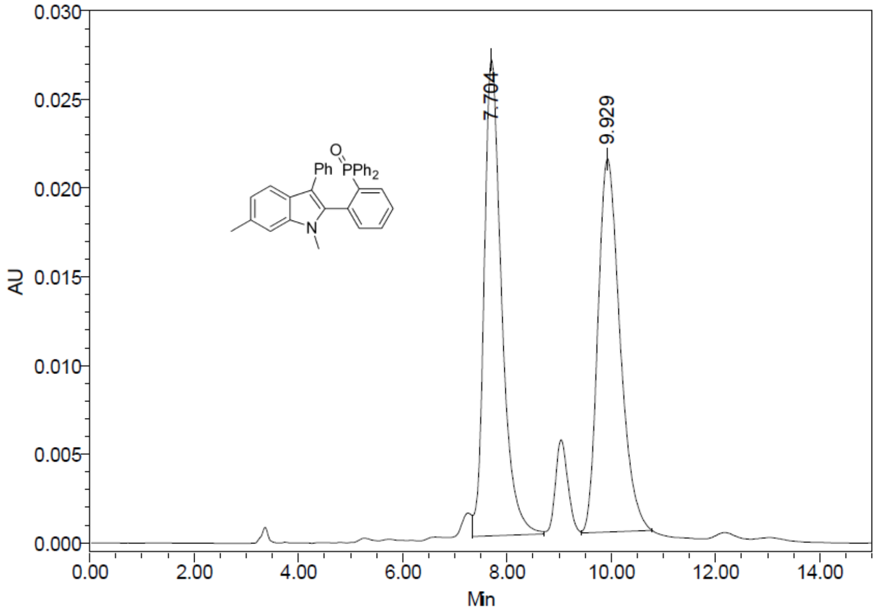

Peak Table

W2489 CHA 254

	Ret.Time	Height	Area	Area%
1	7.704	26891	626547	50.342
2	9.929	21047	618035	49.658

Supplementary Figure 303. rac-d4: OD-H, Hexane / *i*PrOH = 90/10, rate = 1.0 mL/min, 254 nm

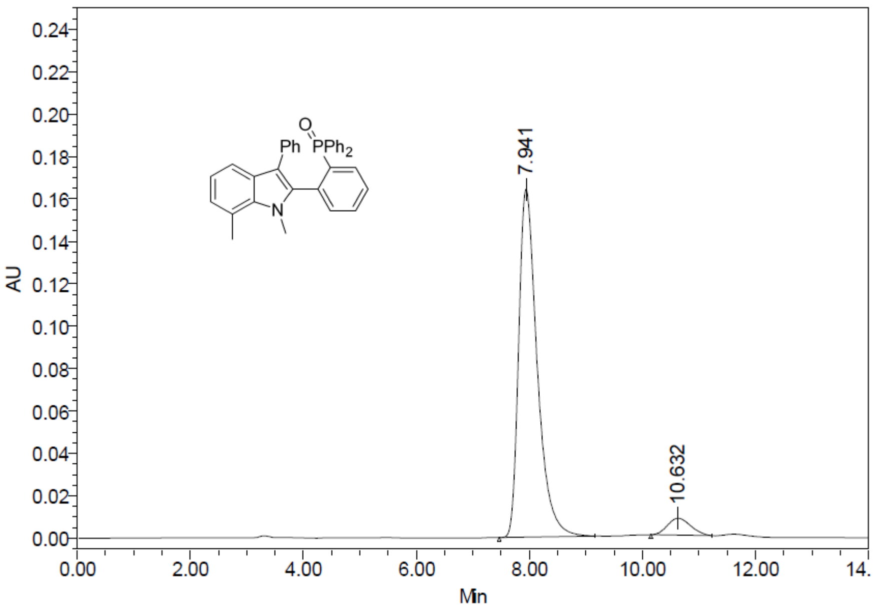

Peak Table

W2489 CHA 254

	Ret.Time	Height	Area	Area%
1	7.941	164308	3715006	94.266
2	10.632	7937	225959	5.734

Supplementary Figure 304. d5: OD-H, Hexane / *i*PrOH = 90/10, rate = 1.0 mL/min, 254 nm

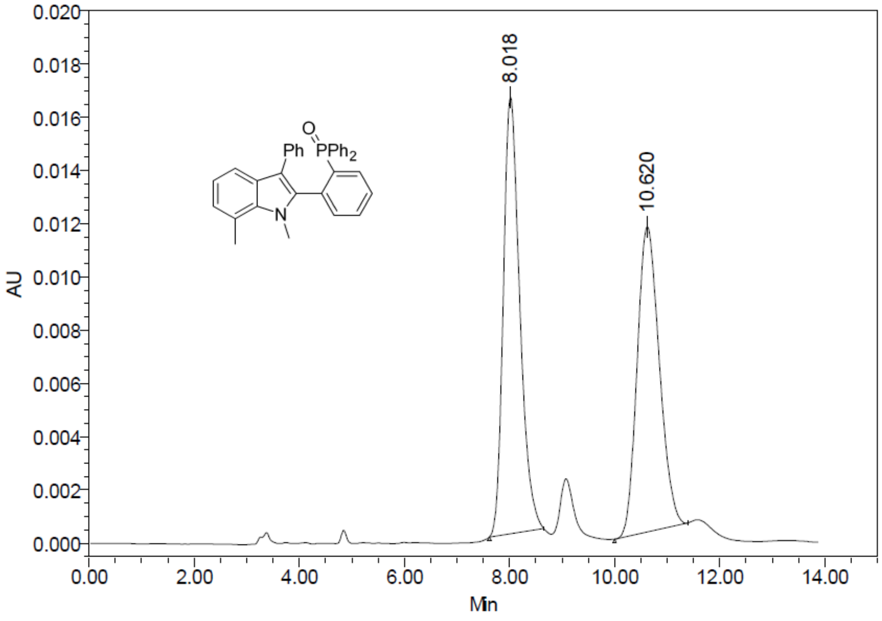

Peak Table

W2489 CHA 254

	Ret.Time	Height	Area	Area%
1	8.018	16410	357047	51.492
2	10.620	11472	336356	48.508

Supplementary Figure 305. rac-d5: OD-H, Hexane / *i*PrOH = 90/10, rate = 1.0 mL/min, 254 nm

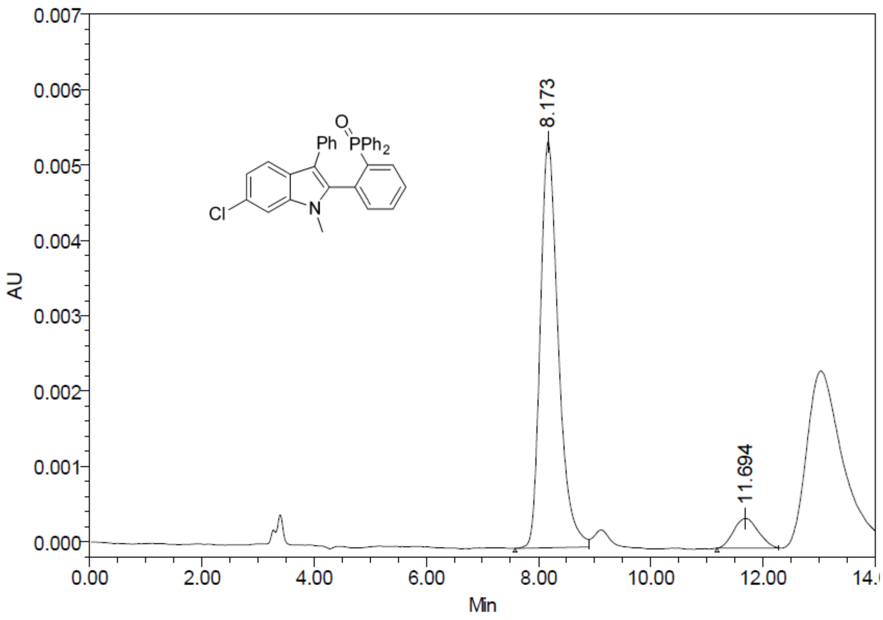

Peak Table

W2489 CHA 254

	Ret.Time	Height	Area	Area%
1	8.173	5393	120515	90.937
2	11.694	393	12010	9.063

Supplementary Figure 306. d6: OD-H, Hexane / *i*PrOH = 90/10, rate = 1.0 mL/min, 254 nm

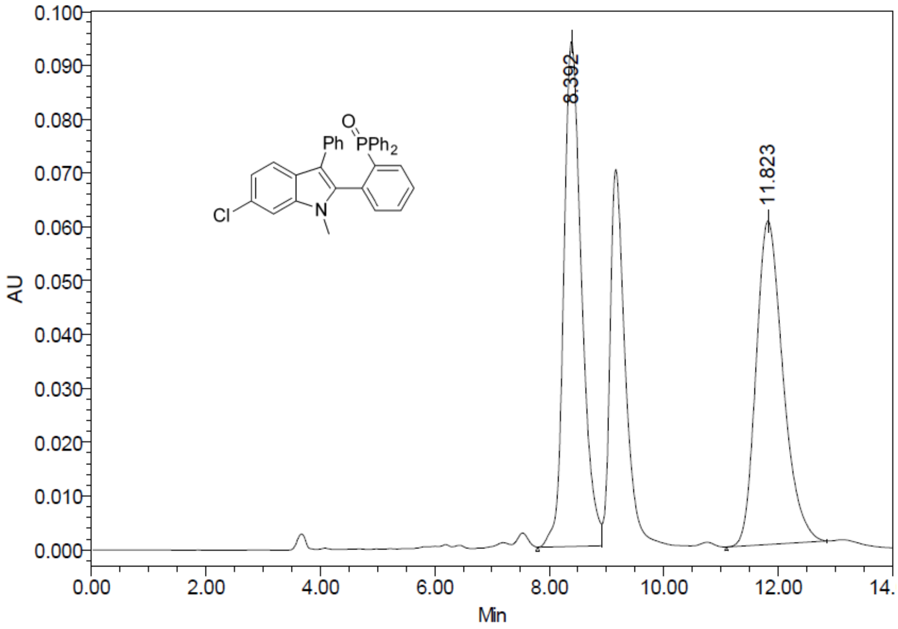

Peak Table

W2489 CHA 254

	Ret.Time	Height	Area	Area%
1	8.392	93963	2030430	50.796
2	11.823	60155	1966798	49.204

Supplementary Figure 307. rac-d6: OD-H, Hexane / *i*PrOH = 90/10, rate = 1.0 mL/min, 254 nm

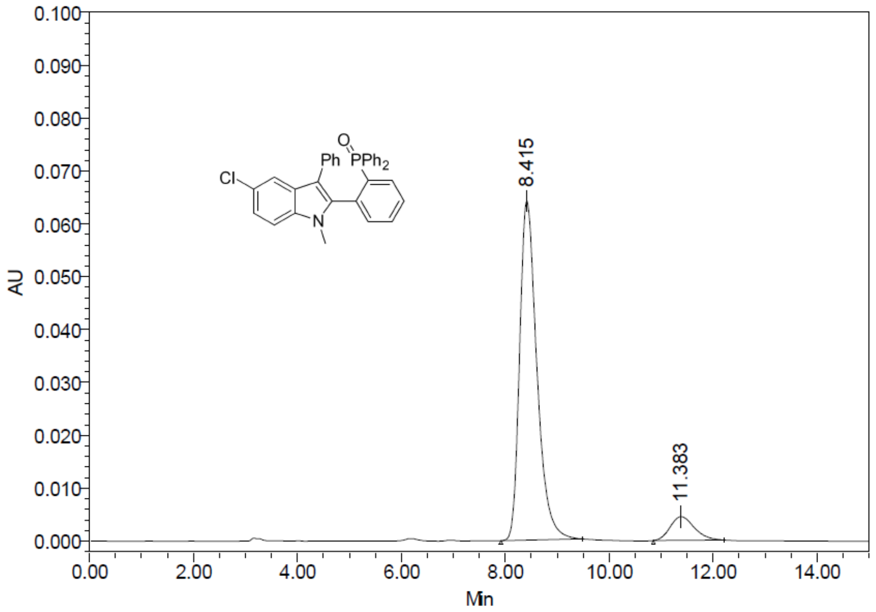

Peak Table

W2489 CHA 254

	Ret.Time	Height	Area	Area%
1	8.415	64187	1479014	91.087
2	11.383	4470	144722	8.913

Supplementary Figure 308. d7: OD-H, Hexane / *i*PrOH = 90/10, rate = 1.0 mL/min, 254 nm

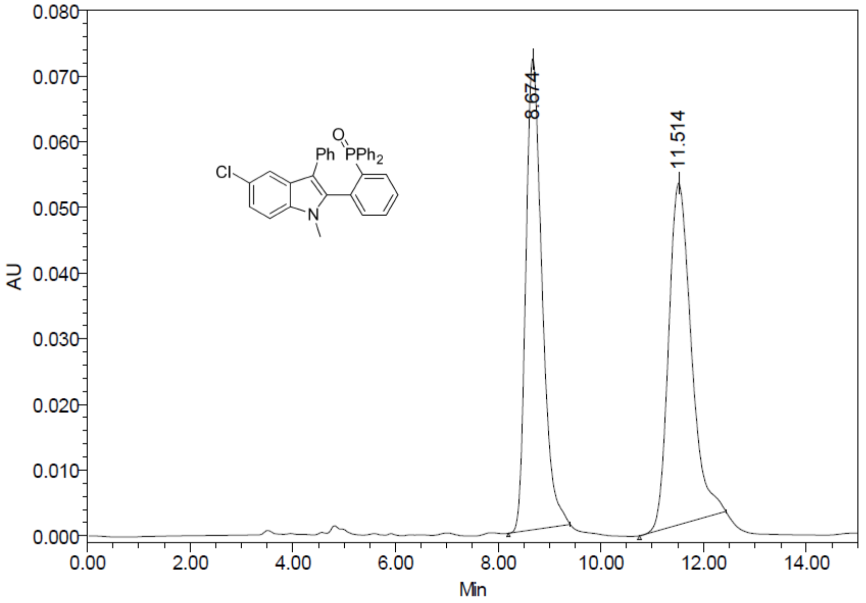

Peak Table

W2489 CHA 254

	Ret.Time	Height	Area	Area%
1	8.674	71759	1576139	49.705
2	11.514	52026	1594868	50.295

Supplementary Figure 309. rac-d7: OD-H, Hexane / *i*PrOH = 90/10, rate = 1.0 mL/min, 254 nm

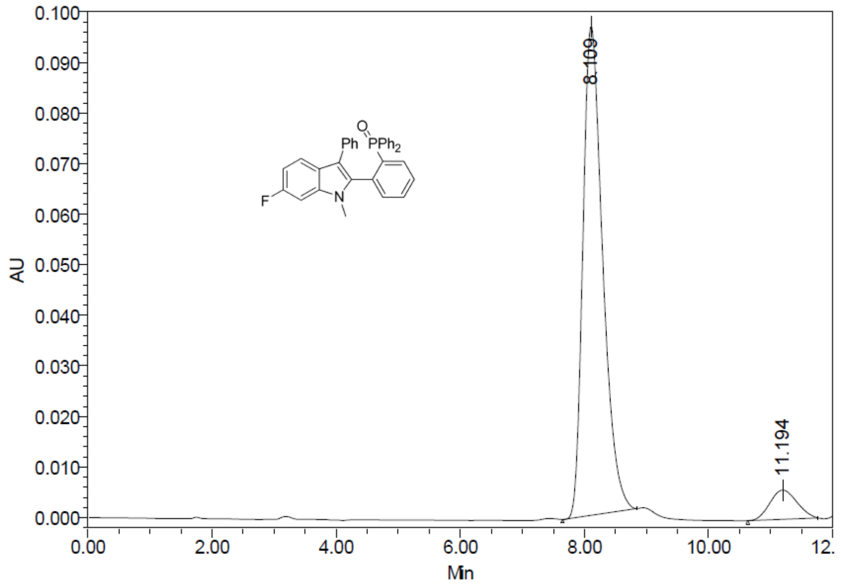

Peak Table

W2489 CHA 254

	Ret.Time	Height	Area	Area%
1	8.109	96837	2141690	92.440
2	11.194	5782	175163	7.560

Supplementary Figure 310. d8: OD-H, Hexane / *i*PrOH = 90/10, rate = 1.0 mL/min, 254 nm

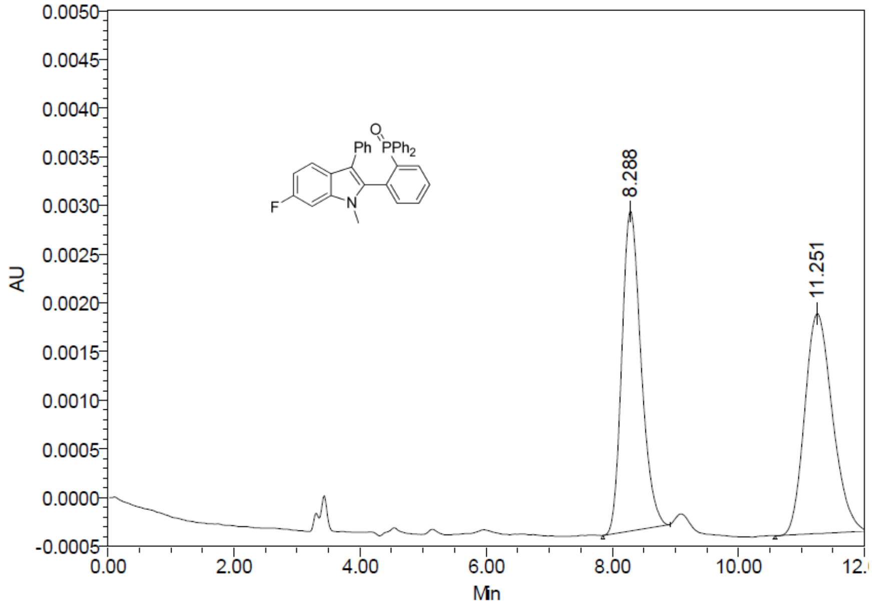

Peak Table

W2489 CHA 254

	Ret.Time	Height	Area	Area%
1	8.288	3288	69685	50.126
2	11.251	2258	69334	49.874

Supplementary Figure 311. rac-d8: OD-H, Hexane / *i*PrOH = 90/10, rate = 1.0 mL/min, 254 nm

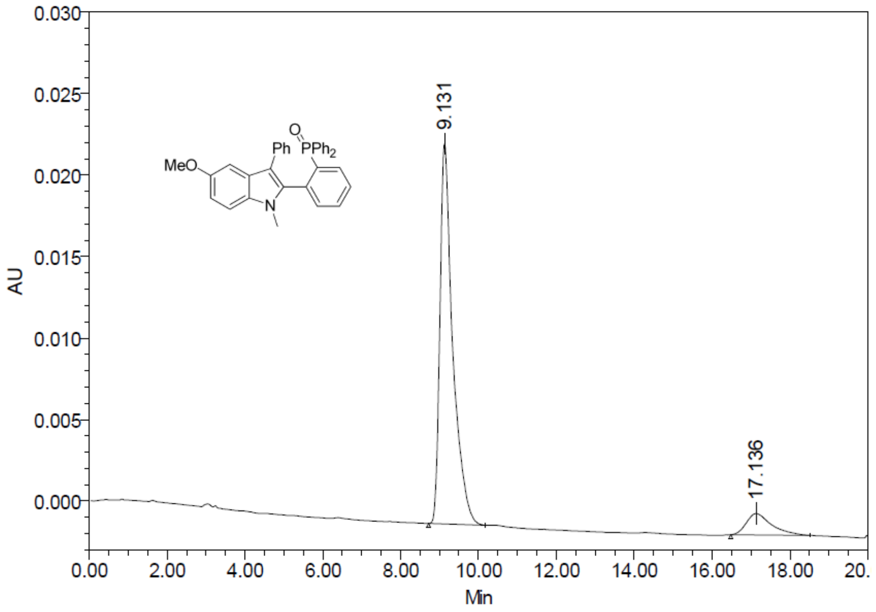

Peak Table

W2489 CHA 254

	Ret.Time	Height	Area	Area%
1	9.131	23345	534184	89.996
2	17.136	1313	59380	10.004

Supplementary Figure 312. d9: AD-H, Hexane / *i*PrOH = 85/15, rate = 1.0 mL/min, 254 nm

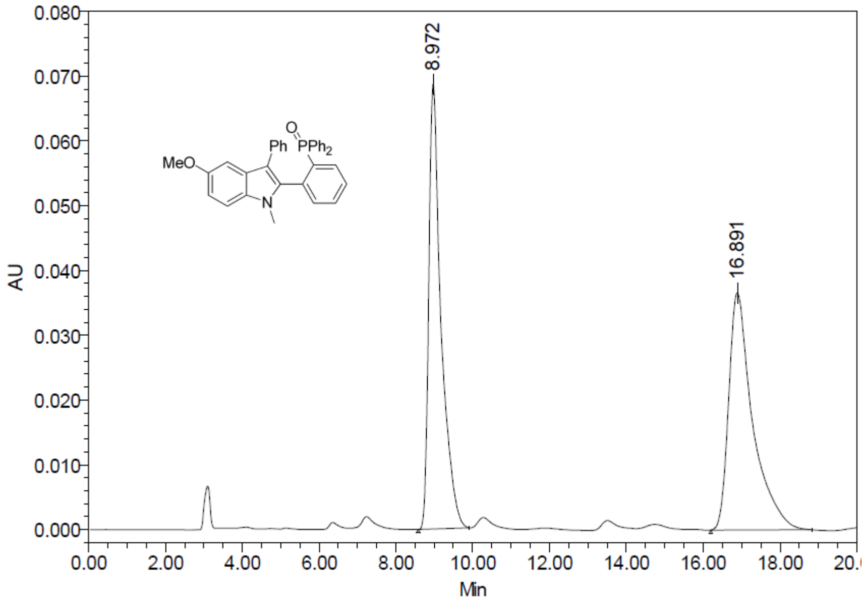

Peak Table

W2489 CHA 254

	Ret.Time	Height	Area	Area%
1	8.972	68768	1525710	49.057
2	16.891	36628	1584367	50.943

Supplementary Figure 313. rac-d9: AD-H, Hexane / *i*PrOH = 85/15, rate = 1.0 mL/min, 254 nm

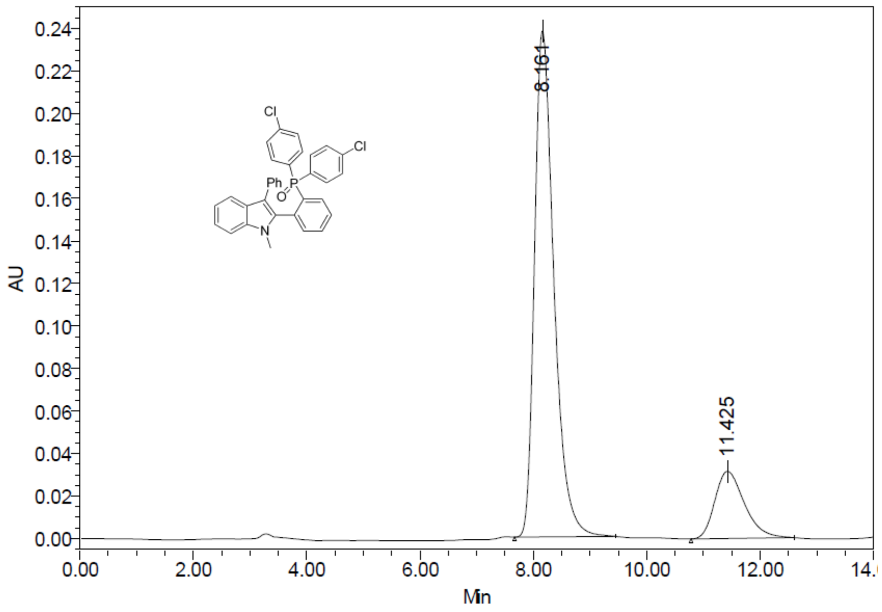

Peak Table

W2489 CHA 254

	Ret.Time	Height	Area	Area%
1	8.161	238274	5671095	83.149
2	11.425	31651	1149326	16.851

Supplementary Figure 314. d10: OD-H, Hexane/*i*PrOH = 90/10, rate = 1.0 mL/min, 254 nm

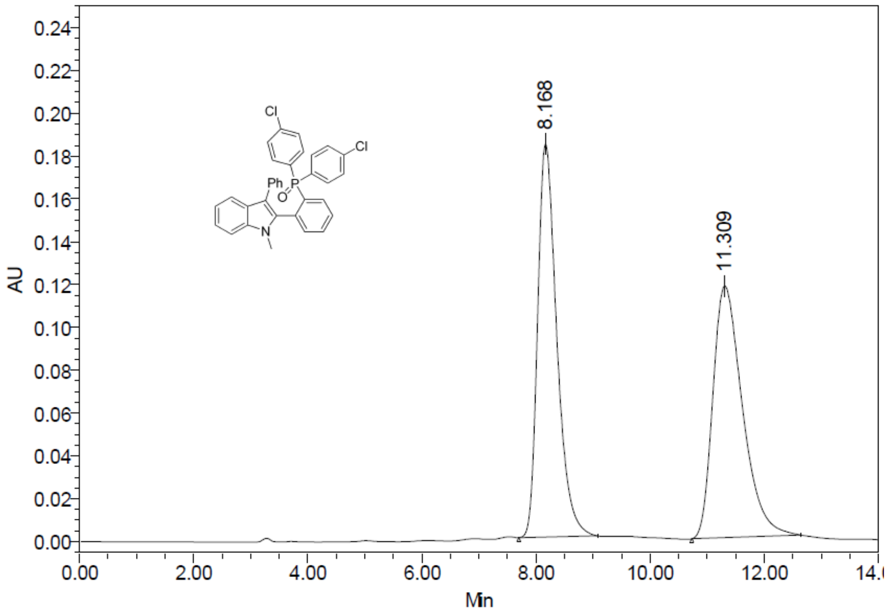

Peak Table

W2489 CHA 254

	Ret.Time	Height	Area	Area%
1	8.168	183747	4289102	50.324
2	11.309	117596	4233868	49.676

Supplementary Figure 315. rac-d10: OD-H, Hexane/*i*PrOH = 90/10, rate = 1.0 mL/min, 254 nm

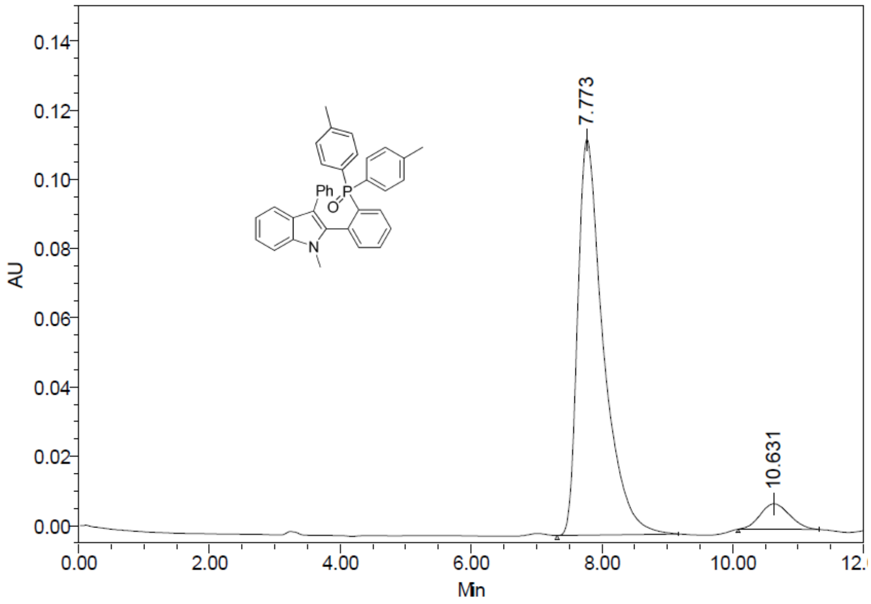

Peak Table

W2489 CHA 254

	Ret.Time	Height	Area	Area%
1	7.773	114366	3124393	93.101
2	10.631	7357	231509	6.899

Supplementary Figure 316. d11: OD-H, Hexane/*i*PrOH = 90/10, rate = 1.0 mL/min, 254 nm

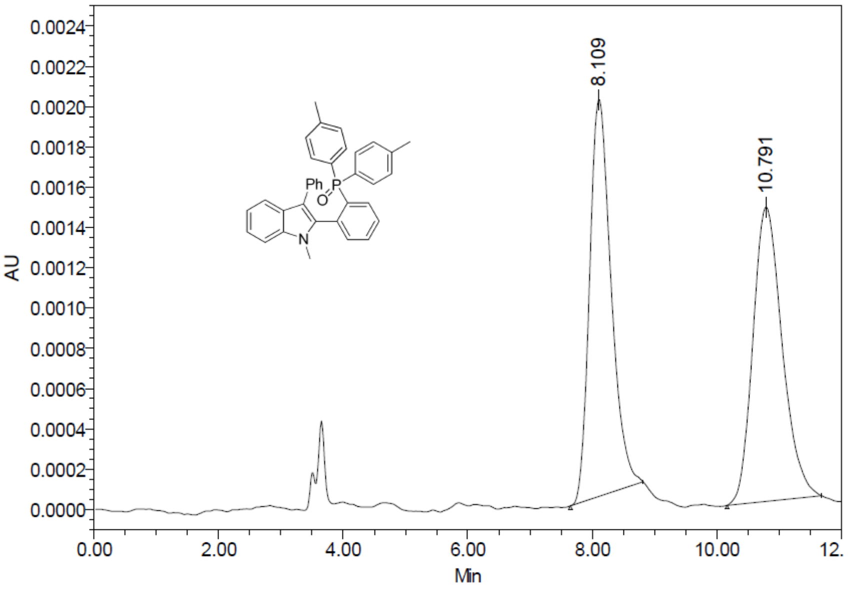

Peak Table

W2489 CHA 254

	Ret.Time	Height	Area	Area%
1	8.109	1972	47176	49.689
2	10.791	1461	47767	50.311

Supplementary Figure 317. rac-d11: OD-H, Hexane/*i*PrOH = 90/10, rate = 1.0 mL/min, 254 nm

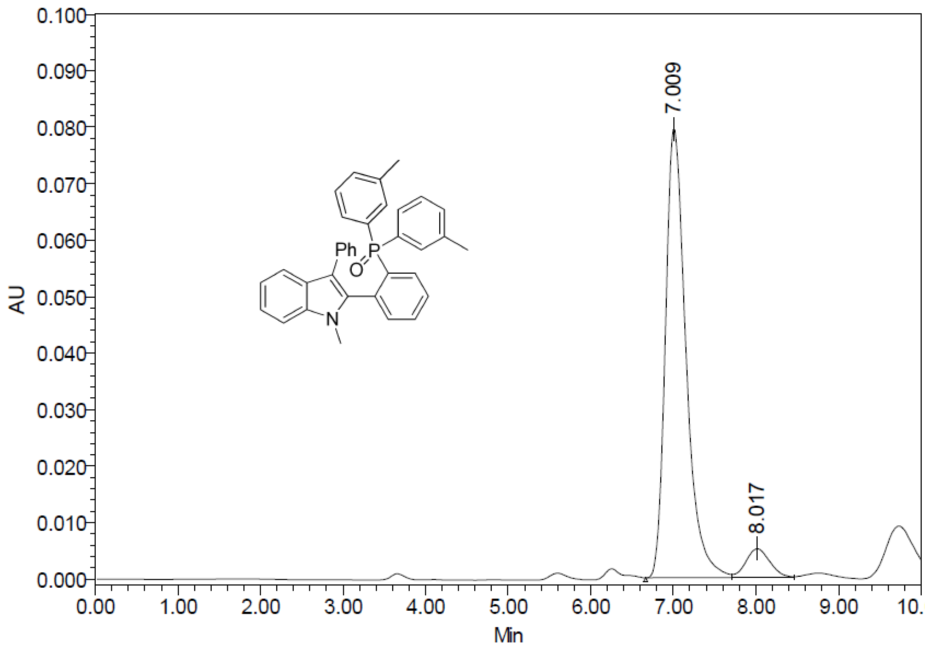

Peak Table

W2489 CHA 254

	Ret.Time	Height	Area	Area%
1	7.009	79690	1374115	93.060
2	8.017	5113	102483	6.940

Supplementary Figure 318. d12: OD-H, Hexane/*i*PrOH = 90/10, rate = 1.0 mL/min, 254 nm

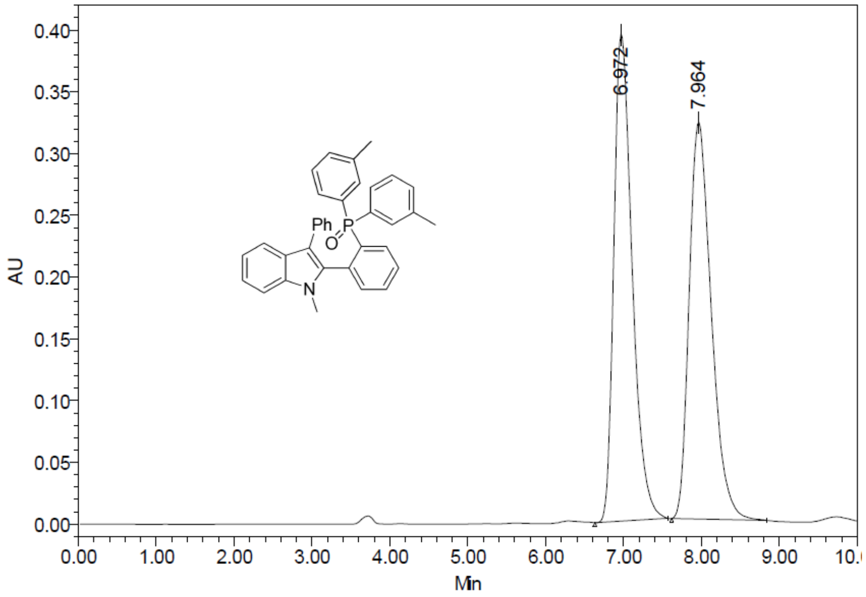

Peak Table

W2489 CHA 254

	Ret.Time	Height	Area	Area%
1	6.972	394901	6434871	50.398
2	7.964	321246	6333229	49.602

Supplementary Figure 319. rac-d12: OD-H, Hexane/*i*PrOH = 90/10, rate = 1.0 mL/min, 254 nm

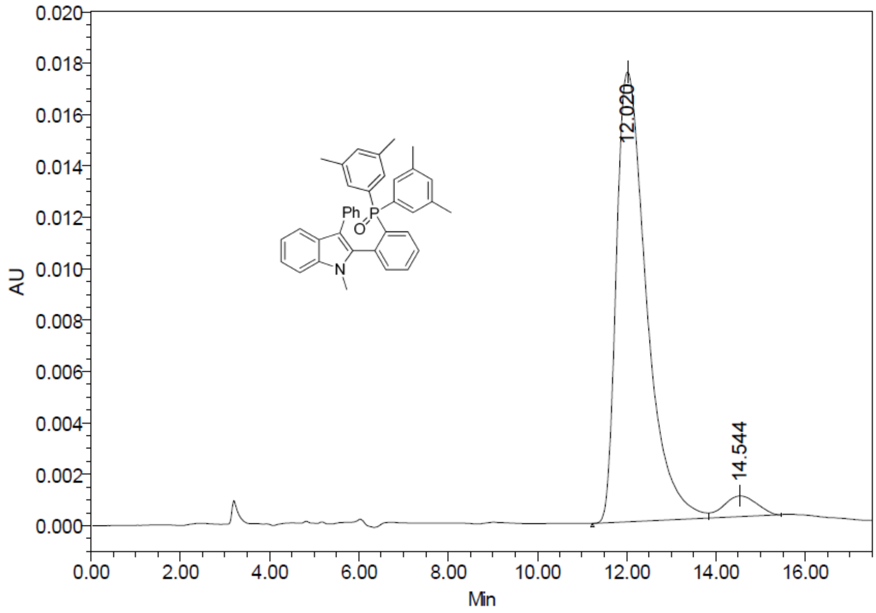

Peak Table

W2489 CHA 254

	Ret.Time	Height	Area	Area%
1	12.020	17501	819063	95.195
2	14.544	804	41338	4.805

Supplementary Figure 320. d13: OD-H, Hexane/*i*PrOH = 97/03, rate = 1.0 mL/min, 254 nm

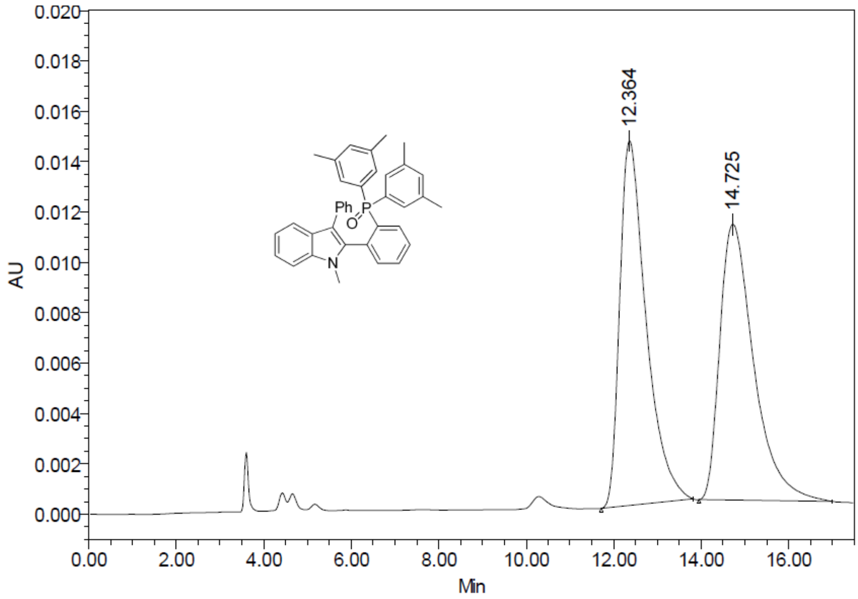

Peak Table

W2489 CHA 254

	Ret.Time	Height	Area	Area%
1	12.364	14467	606740	50.662
2	14.725	10950	590891	49.338

Supplementary Figure 321. rac-d13: OD-H, Hexane/*i*PrOH = 97/03, rate = 1.0 mL/min, 254 nm

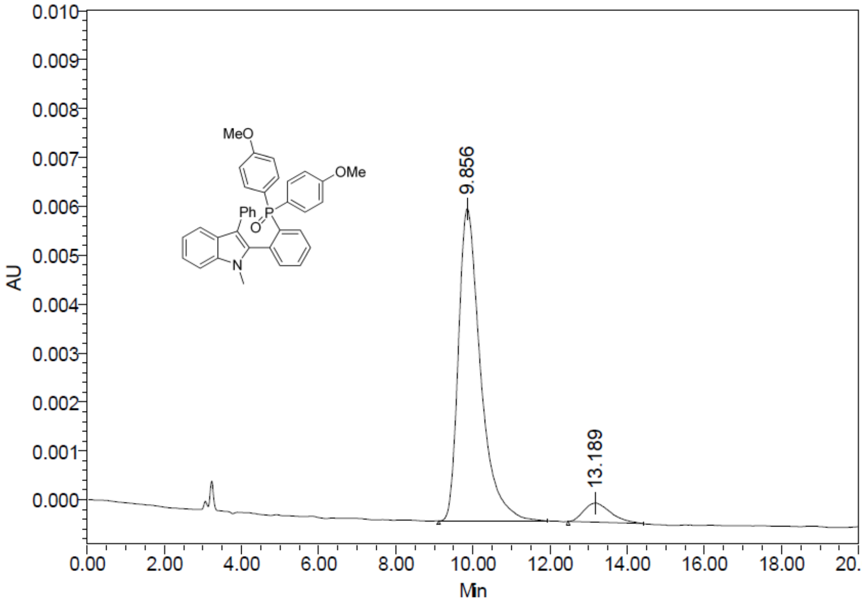

Peak Table

W2489 CHA 254

	Ret. Time	Height	Area	Area%
1	9.856	6385	253966	92.986
2	13.189	395	19156	7.014

Supplementary Figure 322. d14: OD-H, Hexane/*i*PrOH = 85/15, rate = 1.0 mL/min, 254 nm

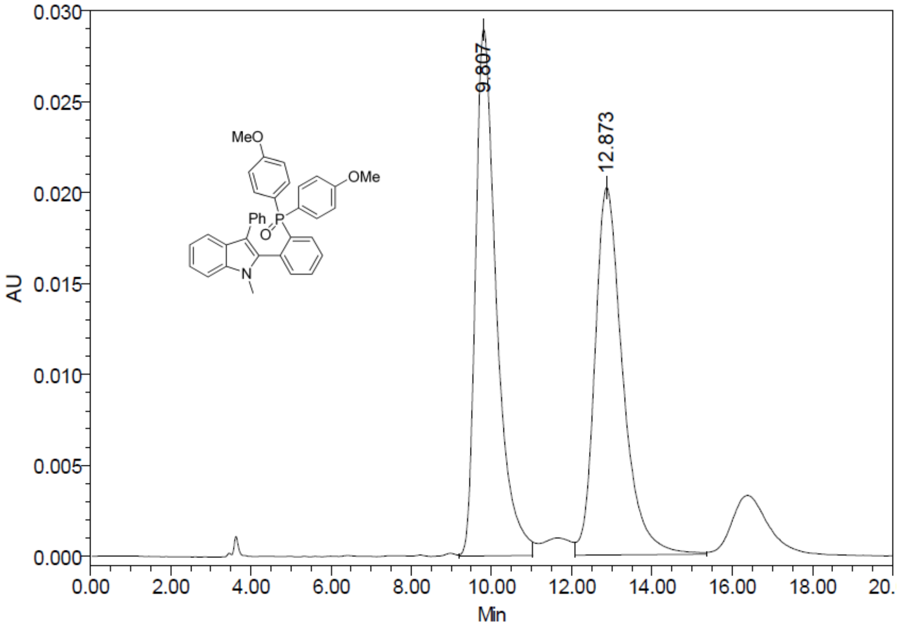

Peak Table

W2489 CHA 254

	Ret. Time	Height	Area	Area%
1	9.807	28954	1045560	51.704
2	12.873	20205	976657	48.296

Supplementary Figure 323. rac-d14: OD-H, Hexane/*i*PrOH = 85/15, rate = 1.0 mL/min, 254 nm

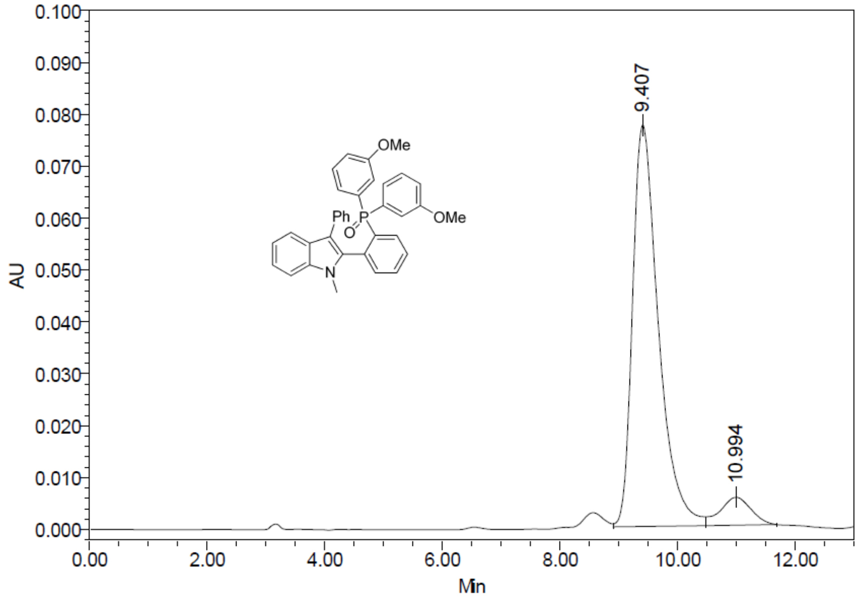

Peak Table

W2489 CHA 254

	Ret.Time	Height	Area	Area%
1	9.407	77416	2366476	92.360
2	10.994	5372	195746	7.640

Supplementary Figure 324. d15: OD-H, Hexane/*i*PrOH = 90/10, rate = 1.0 mL/min, 254 nm

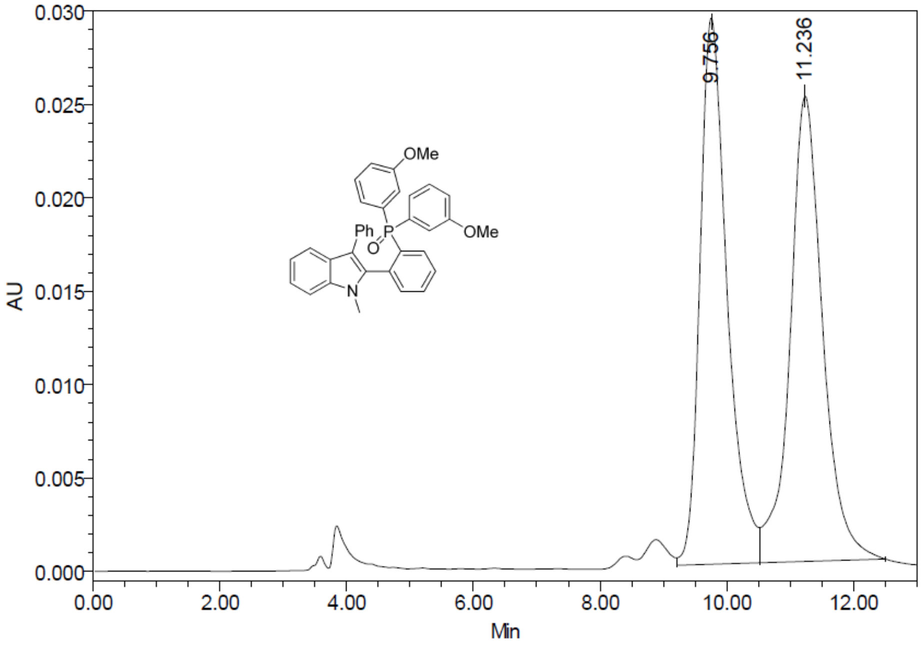

Peak Table

W2489 CHA 254

	Ret.Time	Height	Area	Area%
1	9.756	29247	872766	49.450
2	11.236	24913	892194	50.550

Supplementary Figure 325. rac-d15: OD-H, Hexane/*i*PrOH = 90/10, rate = 1.0 mL/min, 254 nm

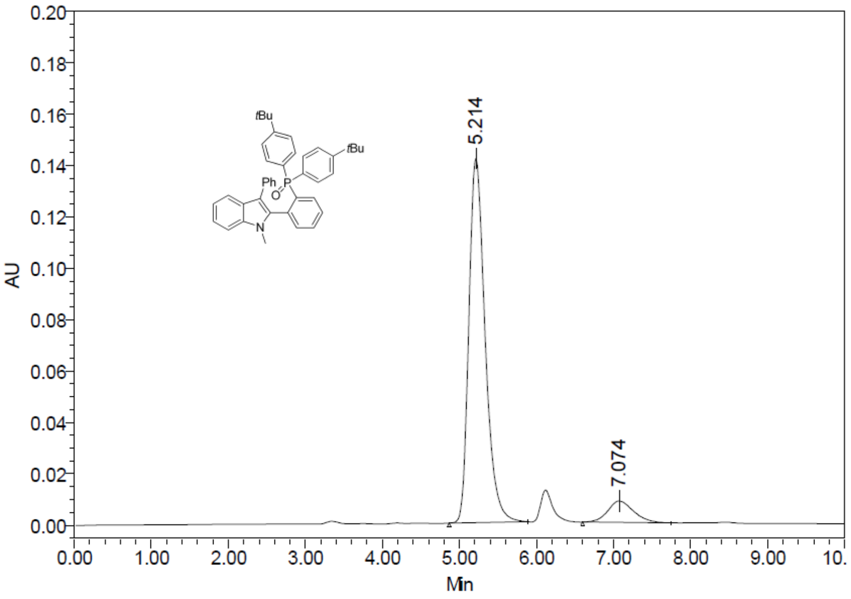

Peak Table

W2489 CHA 254

	Ret.Time	Height	Area	Area%
1	5.214	141953	2072677	91.405
2	7.074	8330	194891	8.595

Supplementary Figure 326. d16: OD-H, Hexane/*i*PrOH = 90/10, rate = 1.0 mL/min, 254 nm

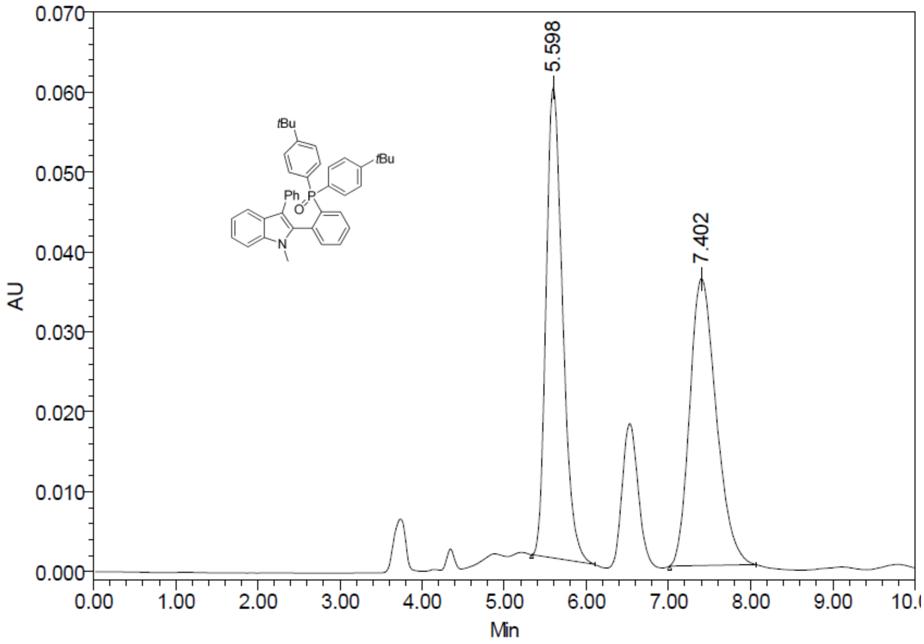

Peak Table

W2489 CHA 254

	Ret.Time	Height	Area	Area%
1	5.598	58946	824047	50.532
2	7.402	35894	806711	49.468

Supplementary Figure 327. rac-d16: OD-H, Hexane/*i*PrOH = 90/10, rate = 1.0 mL/min, 254 nm

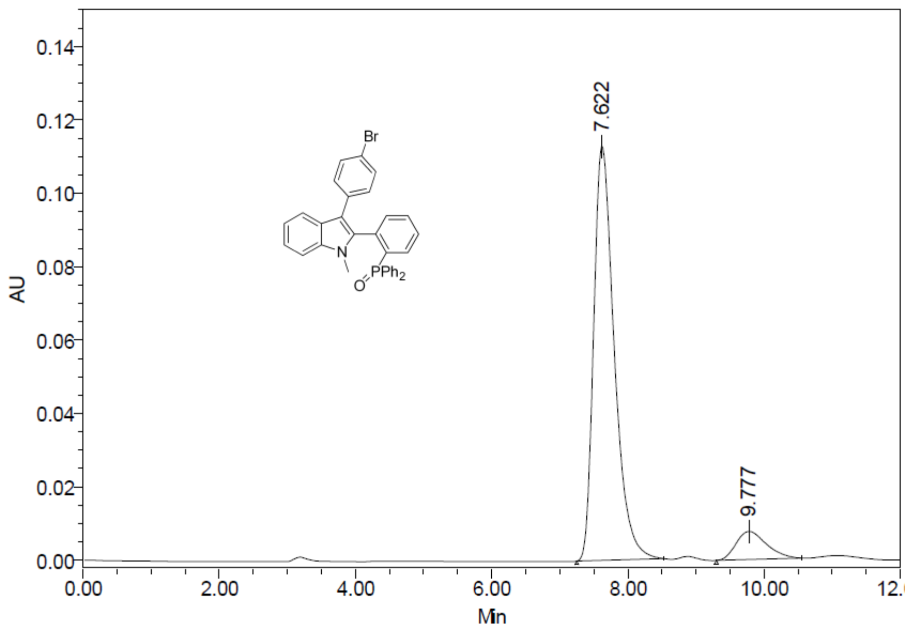

Peak Table

W2489 CHA 254

	Ret.Time	Height	Area	Area%
1	7.622	113011	2364921	90.842
2	9.777	7688	238405	9.158

Supplementary Figure 328. d17: OD-H, Hexane/*i*PrOH = 90/10, rate = 1.0 mL/min, 254 nm

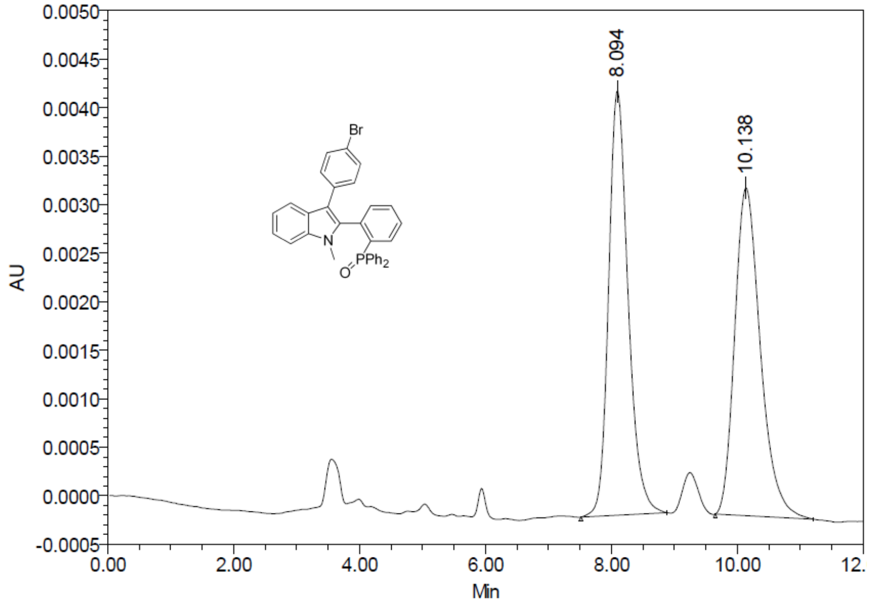

Peak Table

W2489 CHA 254

	Ret.Time	Height	Area	Area%
1	8.094	4374	93749	49.560
2	10.138	3379	95414	50.440

Supplementary Figure 329. rac-d17: OD-H, Hexane/*i*PrOH = 90/10, rate = 1.0 mL/min, 254 nm

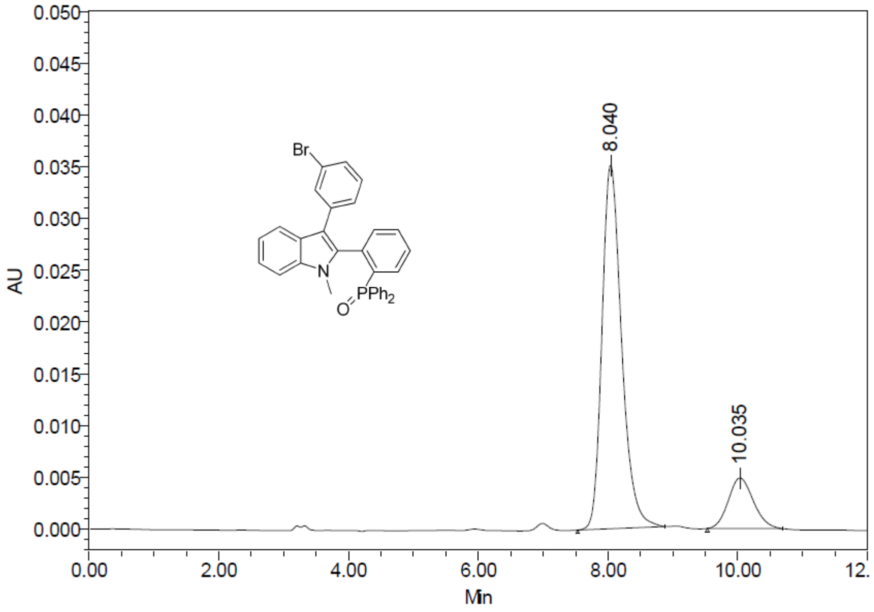

Peak Table

W2489 CHA 254

	Ret.Time	Height	Area	Area%
1	8.040	35163	737167	84.860
2	10.035	4873	131523	15.140

Supplementary Figure 330. d18: OD-H, Hexane/*i*PrOH = 90/10, rate = 1.0 mL/min, 254 nm

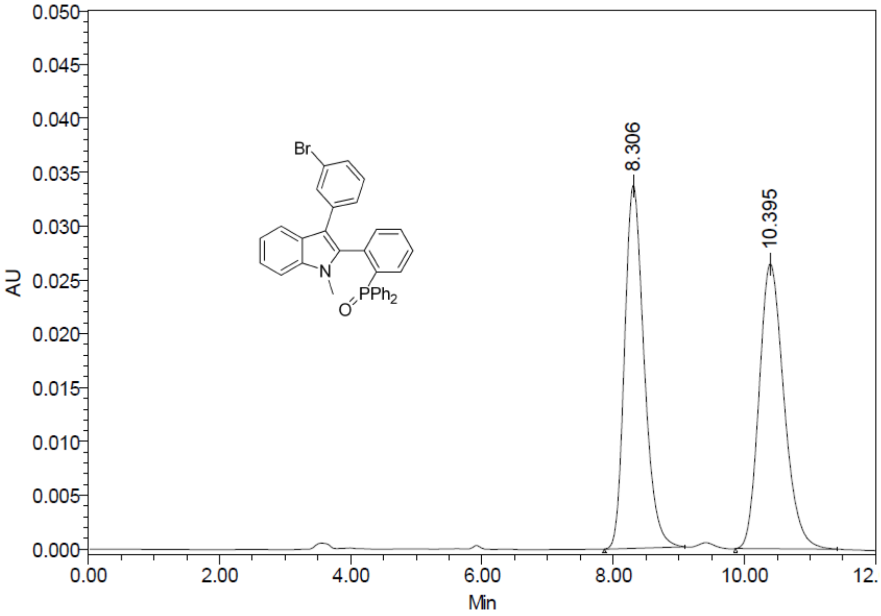

Peak Table

W2489 CHA 254

	Ret.Time	Height	Area	Area%
1	8.306	33714	695016	49.920
2	10.395	26459	697230	50.080

Supplementary Figure 331. rac-d18: OD-H, Hexane/*i*PrOH = 90/10, rate = 1.0 mL/min,

254 n

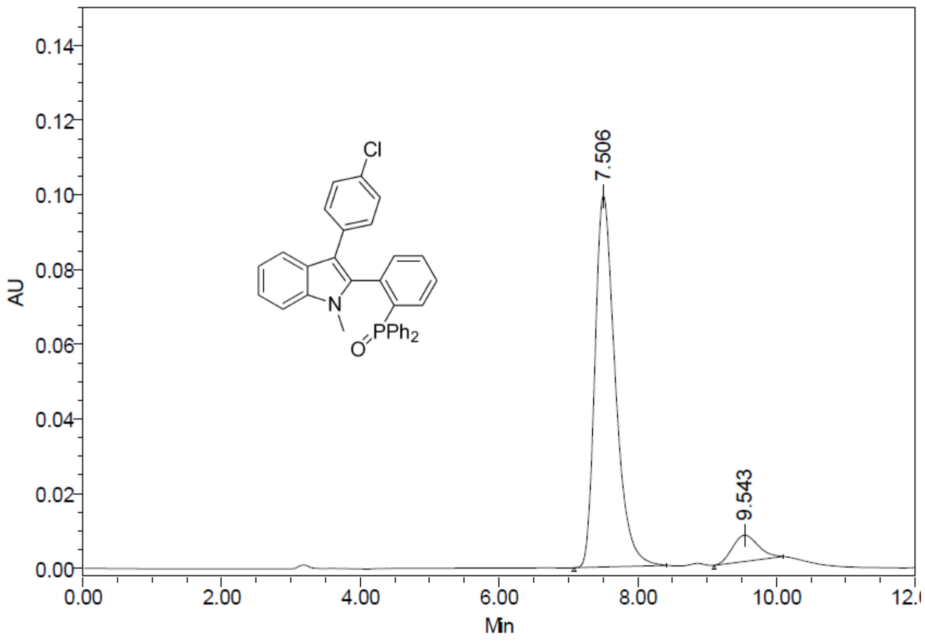

Peak Table

W2489 CHA 254

	Ret.Time	Height	Area	Area%
1	7.506	99456	2020021	91.772
2	9.543	7023	181117	8.228

Supplementary Figure 332. d19: OD-H, Hexane/*i*PrOH = 90/10, rate = 1.0 mL/min, 254 nm

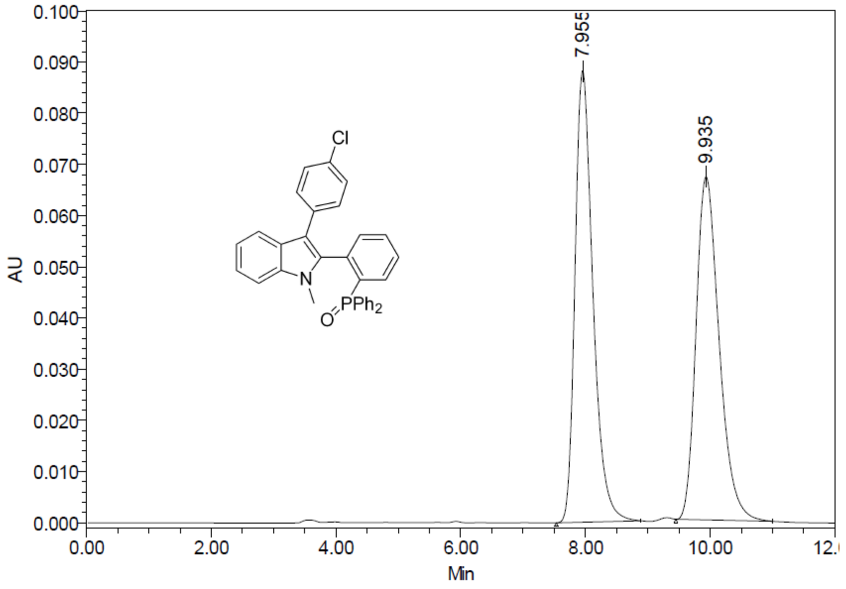

Peak Table

W2489 CHA 254

	Ret.Time	Height	Area	Area%
1	7.955	88219	1758893	50.426
2	9.935	67072	1729189	49.574

Supplementary Figure 333. rac-d19: OD-H, Hexane/*i*PrOH = 90/10, rate = 1.0 mL/min, 254 nm

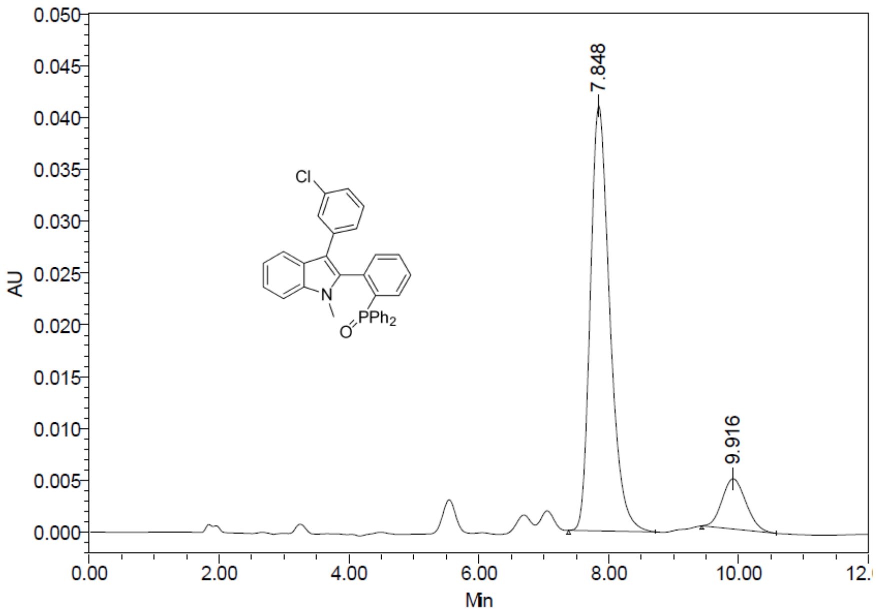

Peak Table

W2489 CHA 254

	Ret.Time	Height	Area	Area%
1	7.848	41004	855105	87.380
2	9.916	4847	123495	12.620

Supplementary Figure 334. d20: OD-H, Hexane/*i*PrOH = 90/10, rate = 1.0 mL/min, 254 nm

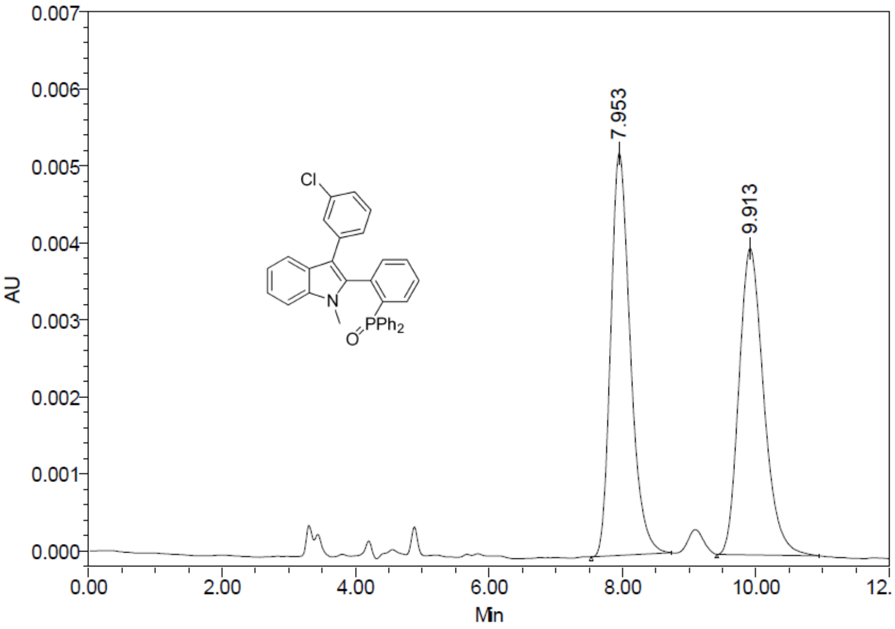

Peak Table

W2489 CHA 254

	Ret.Time	Height	Area	Area%
1	7.953	5229	106727	50.925
2	9.913	3982	102850	49.075

Supplementary Figure 335. rac-d20: OD-H, Hexane/*i*PrOH = 90/10, rate = 1.0 mL/min, 254 nm

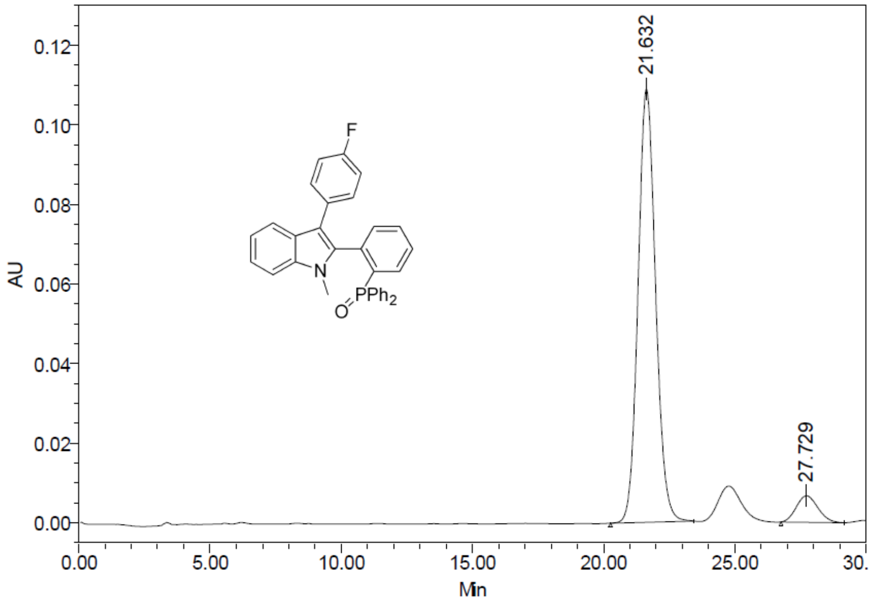

Peak Table

W2489 CHA 254

	Ret.Time	Height	Area	Area%
1	21.632	108972	5061846	93.003
2	27.729	6646	380815	6.997

Supplementary Figure 336. d21: IC, Hexane/*i*PrOH = 80/20, rate = 1.0 mL/min, 254 nm

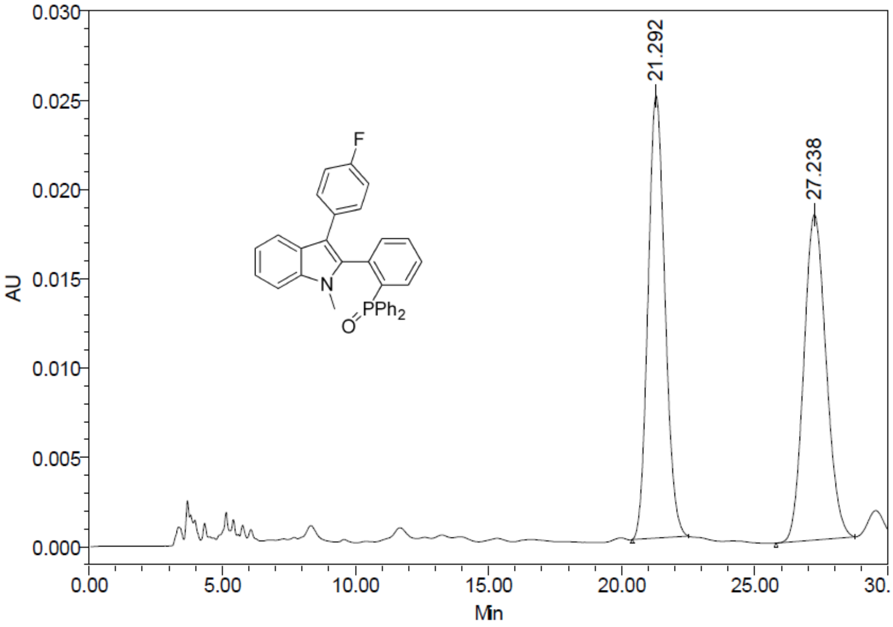

Peak Table

W2489 CHA 254

	Ret.Time	Height	Area	Area%
1	21.292	24792	1093756	50.713
2	27.238	18238	1062996	49.287

Supplementary Figure 337. rac-d21: IC, Hexane/*i*PrOH = 80/20, rate = 1.0 mL/min, 254 nm

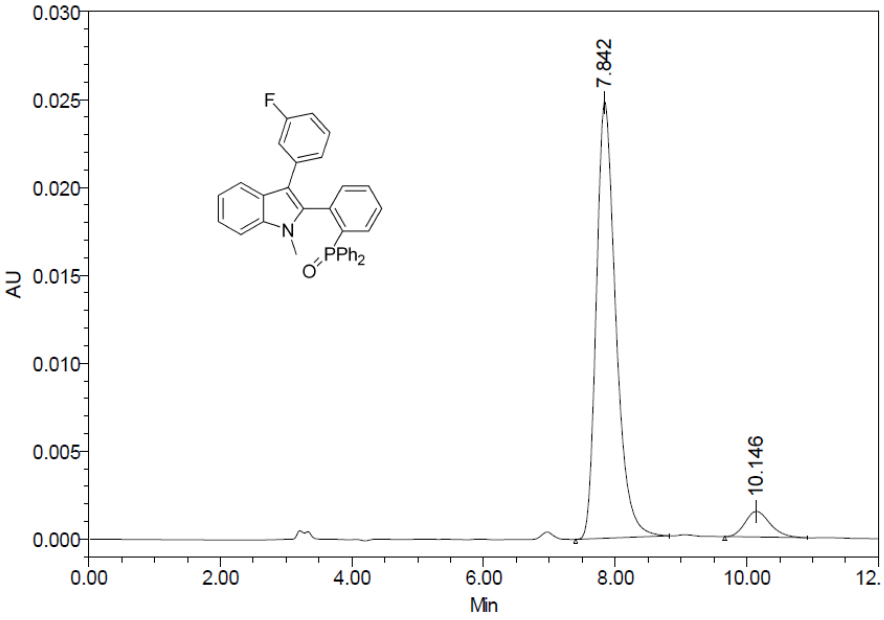

Peak Table

W2489 CHA 254

	Ret.Time	Height	Area	Area%
1	7.842	24842	508307	92.948
2	10.146	1456	38567	7.052

Supplementary Figure 338. d22: OD-H, Hexane/*i*PrOH = 90/10, rate = 1.0 mL/min, 254 nm

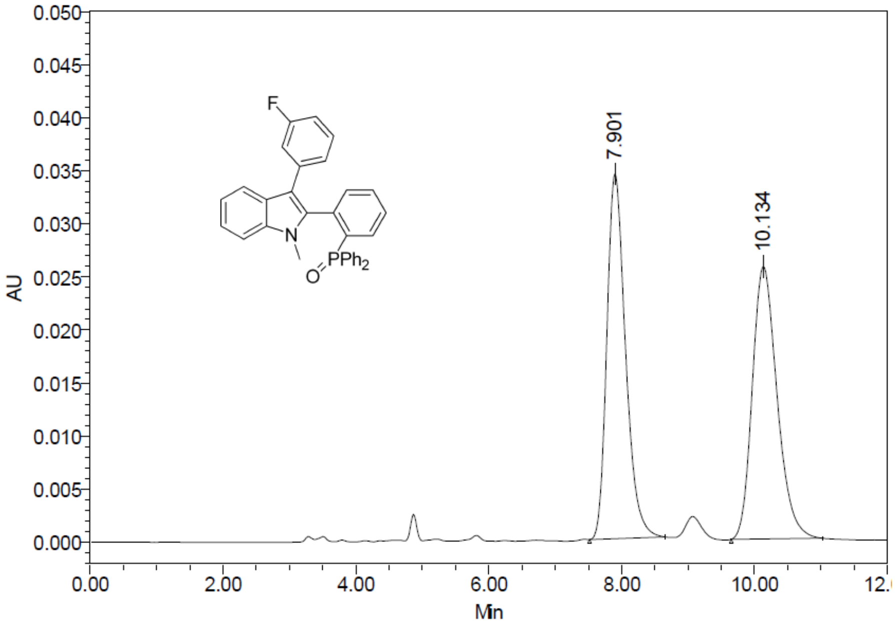

Peak Table

W2489 CHA 254

	Ret.Time	Height	Area	Area%
1	7.901	34391	670632	50.200
2	10.134	25675	665276	49.800

Supplementary Figure 339. rac-d22: OD-H, Hexane/*i*PrOH = 90/10, rate = 1.0 mL/min, 254 nm

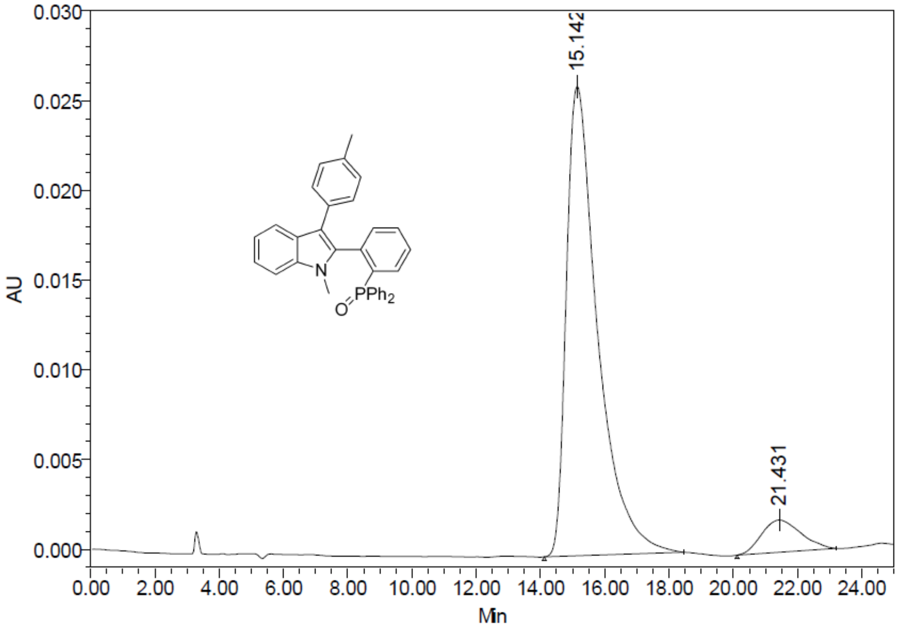

Peak Table

W2489 CHA 254

	Ret. Time	Height	Area	Area%
1	15.142	26164	1772108	92.074
2	21.431	1794	152550	7.926

Supplementary Figure 340. d23: OD-H, Hexane/*i*PrOH = 95/05, rate = 1.0 mL/min, 254 nm

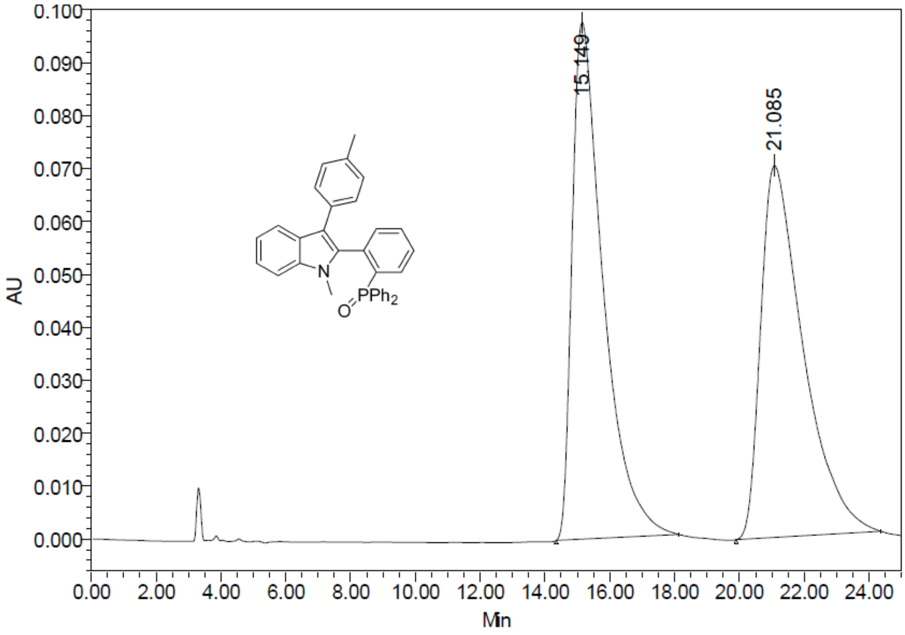

Peak Table

W2489 CHA 254

	Ret. Time	Height	Area	Area%
1	15.149	97712	6517149	50.434
2	21.085	70284	6405023	49.566

Supplementary Figure 341. rac-d23: OD-H, Hexane/*i*PrOH = 95/05, rate = 1.0 mL/min, 254 nm

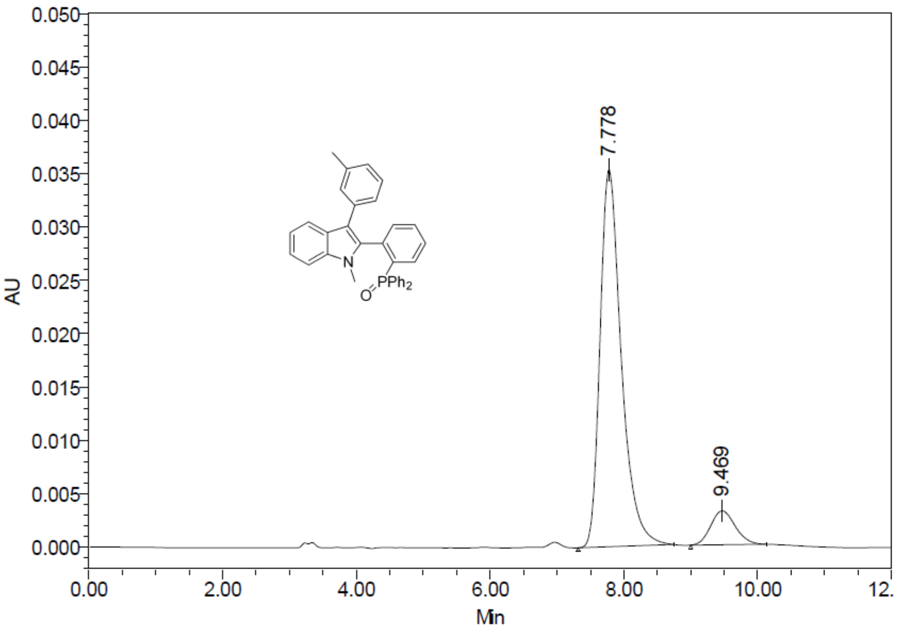

Peak Table

W2489 CHA 254

	Ret. Time	Height	Area	Area%
1	7.778	35346	781071	90.581
2	9.469	3176	81218	9.419

Supplementary Figure 342. d24: OD-H, Hexane/*i*PrOH = 90/10, rate = 1.0 mL/min, 254 nm

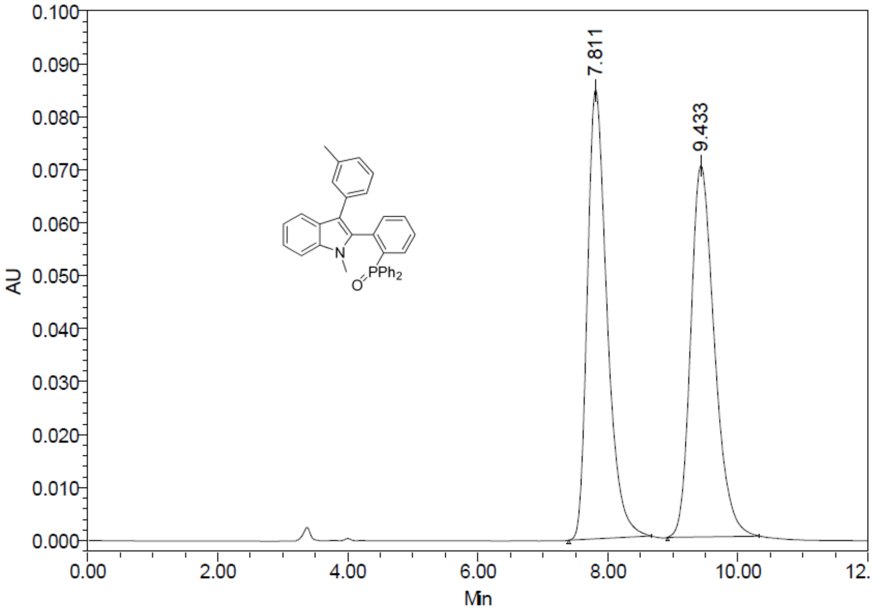

Peak Table

W2489 CHA 254

	Ret. Time	Height	Area	Area%
1	7.811	84781	1779739	50.049
2	9.433	70125	1776268	49.951

Supplementary Figure 343. rac-d24: OD-H, Hexane/*i*PrOH = 90/10, rate = 1.0 mL/min, 254 nm

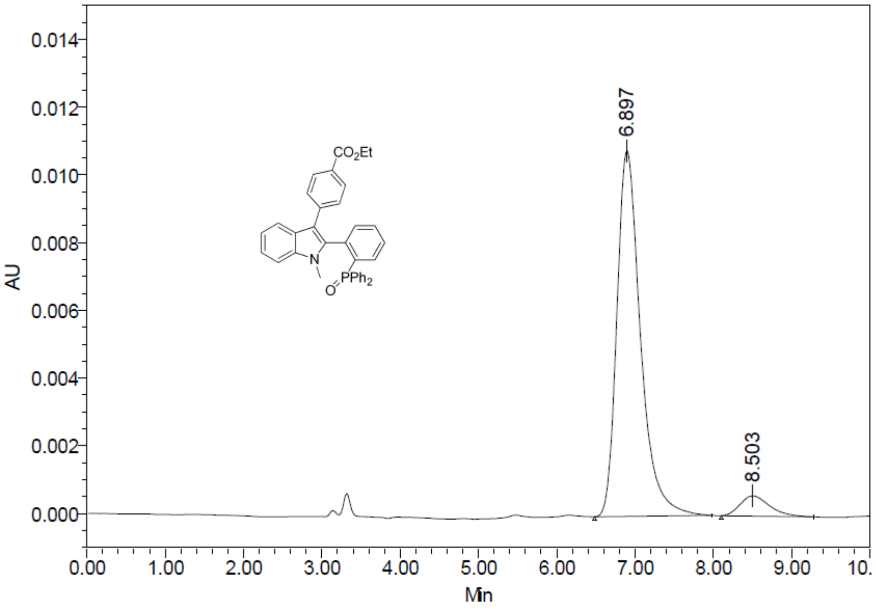

Peak Table

W2489 CHA 254

	Ret.Time	Height	Area	Area%
1	6.897	10804	226491	93.495
2	8.503	596	15757	6.505

Supplementary Figure 344. d25: OD-H, Hexane/*i*PrOH = 85/15, rate = 1.0 mL/min, 254 nm

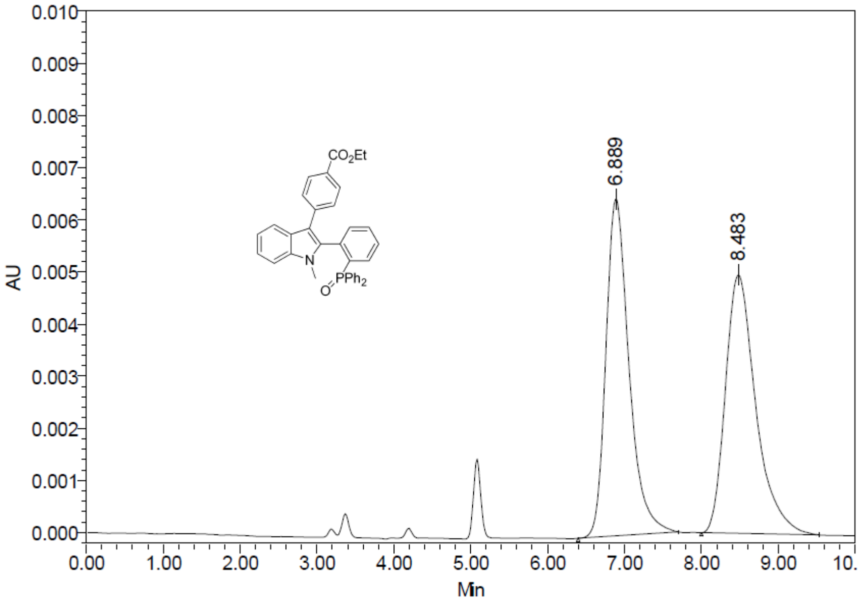

Peak Table

W2489 CHA 254

	Ret.Time	Height	Area	Area%
1	6.889	6465	134872	49.766
2	8.483	4957	136142	50.234

Supplementary Figure 345. rac-d25: OD-H, Hexane/*i*PrOH = 85/15, rate = 1.0 mL/min, 254 nm

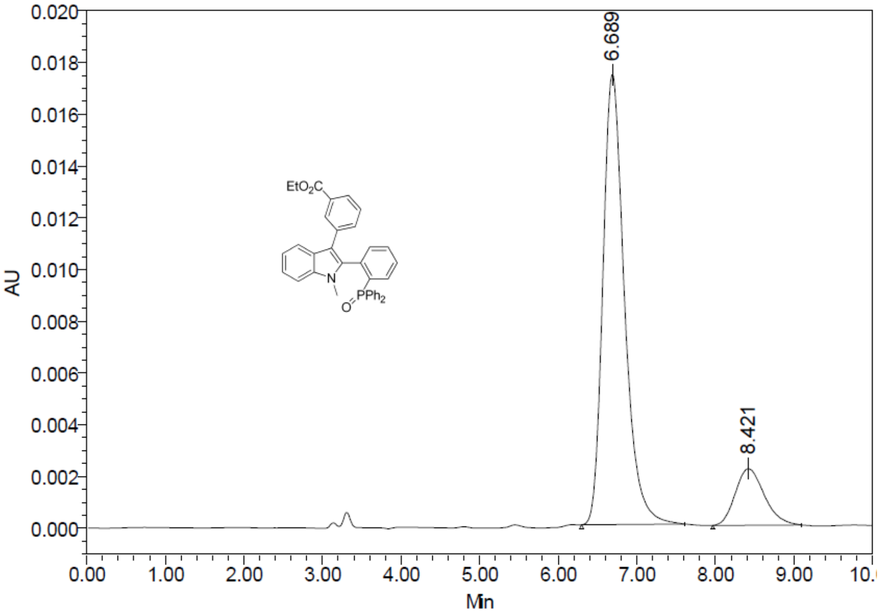

Peak Table

W2489 CHA 254

	Ret.Time	Height	Area	Area%
1	6.689	17427	331412	85.513
2	8.421	2188	56147	14.487

Supplementary Figure 346. d26: OD-H, Hexane/*i*PrOH = 85/15, rate = 1.0 mL/min, 254 nm

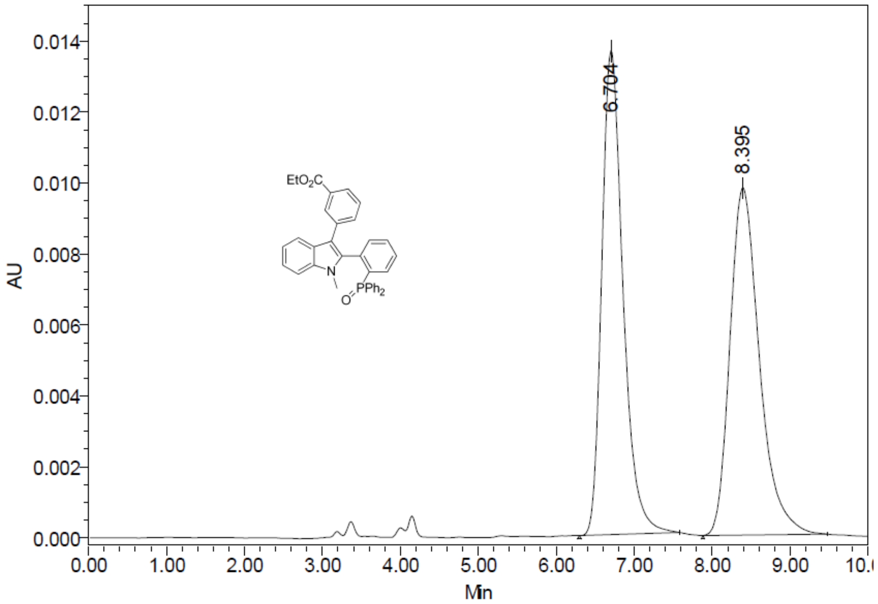

Peak Table

W2489 CHA 254

	Ret.Time	Height	Area	Area%
1	6.704	13655	257895	50.039
2	8.395	9789	257494	49.961

Supplementary Figure 347. rac-d26: OD-H, Hexane/*i*PrOH = 85/15, rate = 1.0 mL/min,

254 nm

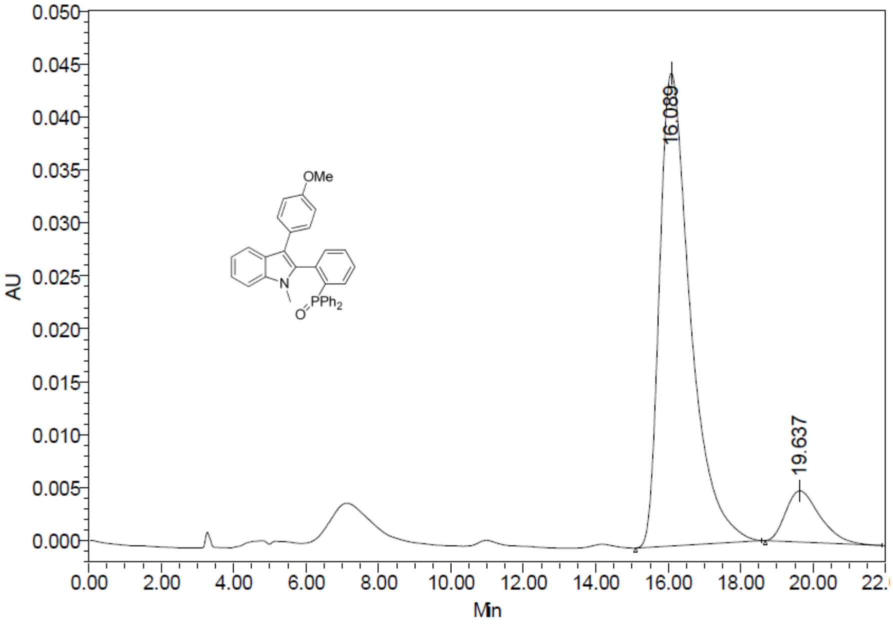

Peak Table

W2489 CHA 254

	Ret.Time	Height	Area	Area%
1	16.089	44627	2651243	89.329
2	19.637	4822	316716	10.671

Supplementary Figure 348. d27: OD-H, Hexane/*i*PrOH = 97/03, rate = 1.0 mL/min, 254 nm

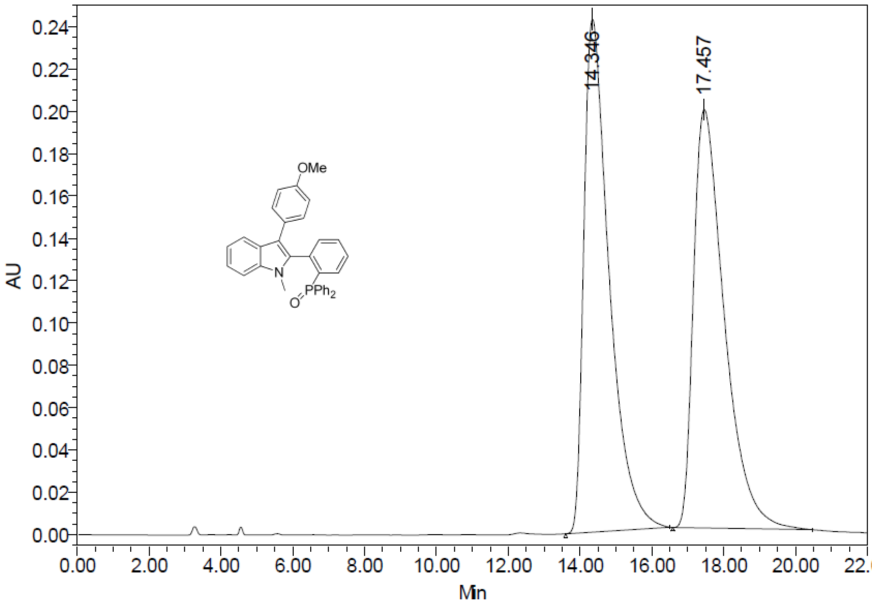

Peak Table

W2489 CHA 254

	Ret.Time	Height	Area	Area%
1	14.346	242462	11890119	50.206
2	17.457	197810	11792782	49.794

Supplementary Figure 349. rac-d27: OD-H, Hexane/*i*PrOH = 97/03, rate = 1.0 mL/min, 254 nm

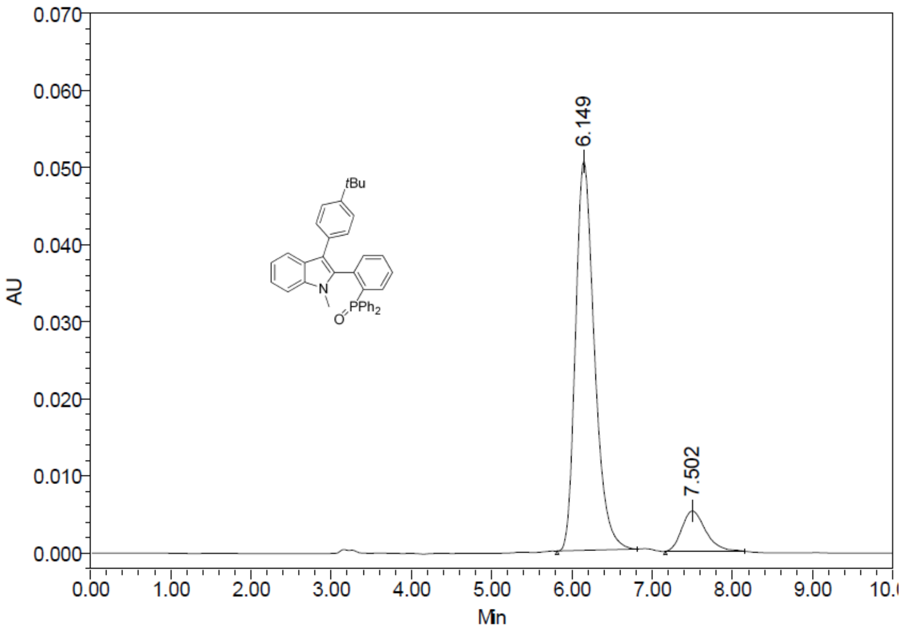

Peak Table

W2489 CHA 254

	Ret.Time	Height	Area	Area%
1	6.149	50518	826625	88.531
2	7.502	5219	107084	11.469

Supplementary Figure 350. d28: OD-H, Hexane/*i*PrOH = 90/10, rate = 1.0 mL/min, 254 nm

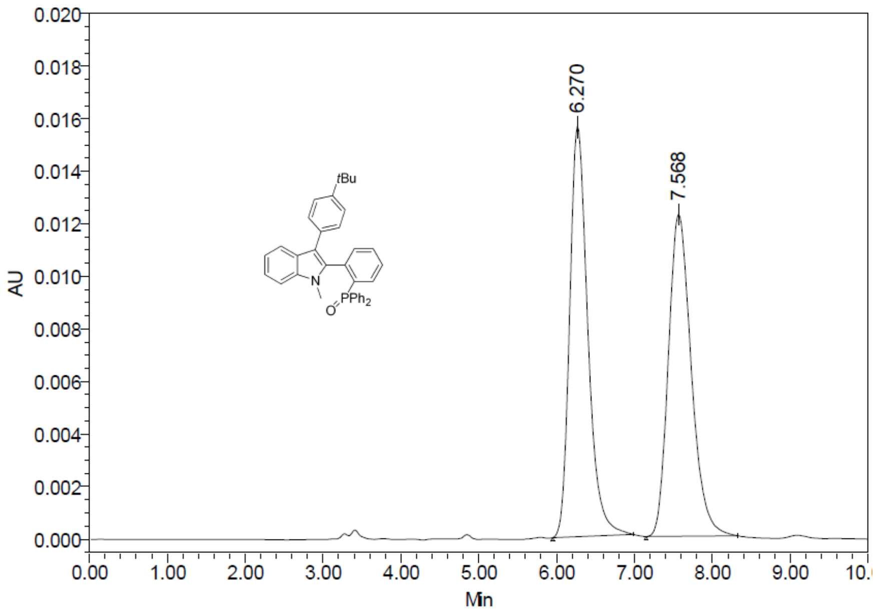

Peak Table

W2489 CHA 254

	Ret.Time	Height	Area	Area%
1	6.270	15624	252461	50.178
2	7.568	12257	250666	49.822

Supplementary Figure 351. rac-d28: OD-H, Hexane/*i*PrOH = 90/10, rate = 1.0 mL/min, 254 nm

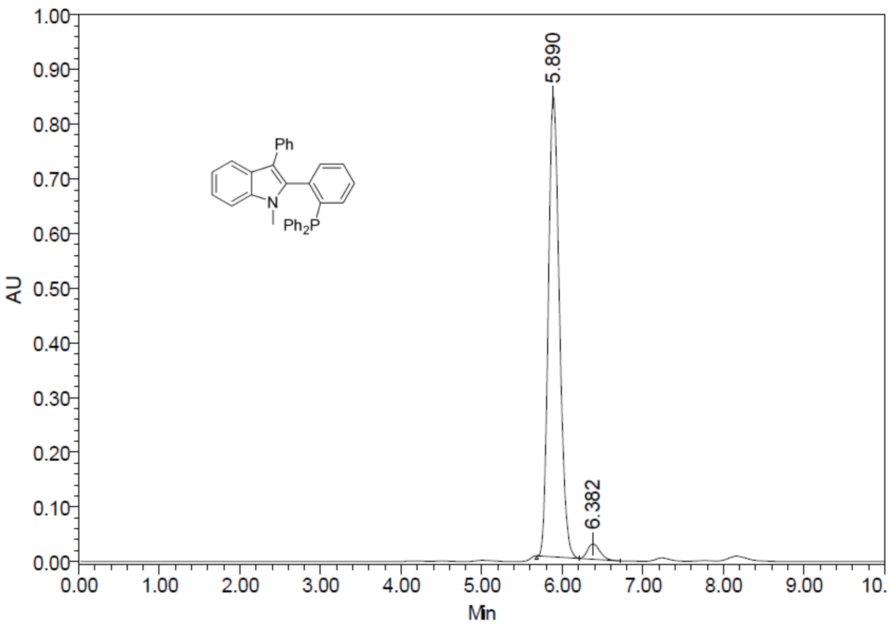

Peak Table

W2489 CHA 254

	Ret.Time	Height	Area	Area%
1	5.890	847439	8094026	96.406
2	6.382	28391	301739	3.594

Supplementary Figure 352. e1: IE, Hexane / *i*PrOH = 98/02, rate = 1.0 mL/min, 254 nm

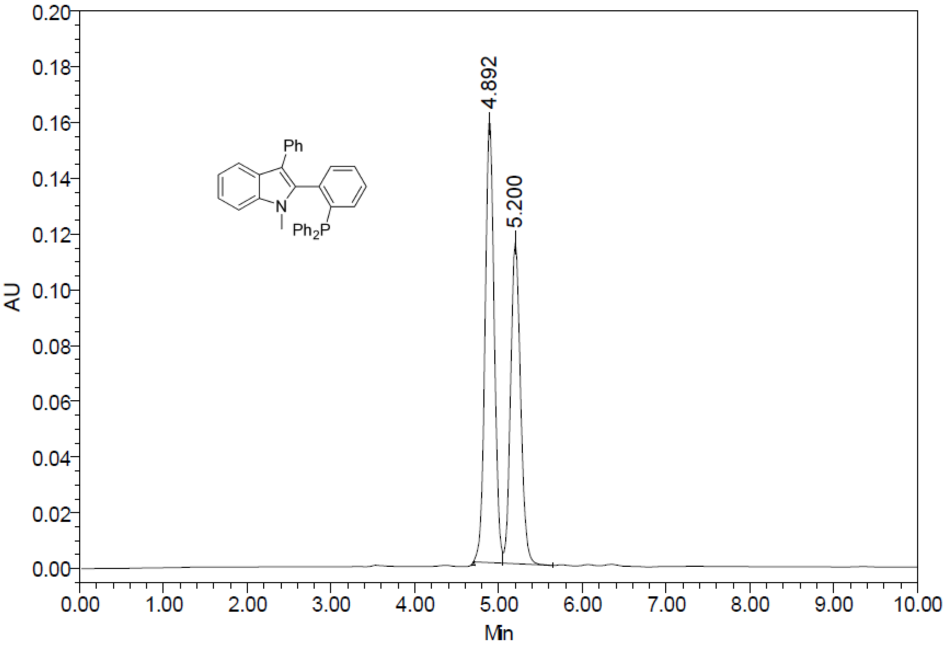

Peak Table

W2489 CHA 254

	Ret.Time	Height	Area	Area%
1	4.892	158821	1205199	55.912
2	5.200	115027	950322	44.088

Supplementary Figure 353. rac-e1: IE, Hexane / *i*PrOH = 98/02, rate = 1.0 mL/min, 254 nm

2. Supplementary References

1. a) Lou, S. & Fu, G. C.; *J. Am. Chem. Soc.* **132**, 1264–1266 (2010). b) Mao, J.-Y., Liu, F.-P., Wang, M., Wu, L., Zheng, B., Liu, S.-Z., Zhong, J.-C., Bian, Q.-H. & Walsh, P. J. *J. Am. Chem. Soc.* **136**, 17662–17668 (2014). c) Wu, H., Wang, Q. & Zhu, J.-P. *Angew. Chem. Int. Ed.* **57**, 2721–2725 (2018).

2. Bielawski, M., Aili, D. & Olofsson, B. *J. Org. Chem.* **73**, 4602–4607 (2008).

3. Phipps, R. J., Grimster, N. P. & Gaunt, M. J. *J. Am. Chem. Soc.* **130**, 8172–8174 (2008).

4. Ragazzon, G., Credi, A. & Colasson, B. *Chem. Eur. J.* **23**, 2149–2156 (2017).

5. Huang, X.-L., Li, C., Wang, J. & Yang, S.-D. *Synthesis* **54**, 4711–4720 (2022).

6. Chen, X.-Y., Liu, X.-Y., Zhu, H. & Wang, Z.-Q. *Tetrahedron* **81**, 131912 (2021).

7. Zhou, Y.-G., Zhang, X.-P., Liang, H.-Y., Cao, Z.-K., Zhao, X.-Y., He, Y.-W., Wang, S.-L., Pang, J.-Y., Zhou, Z.-Y., Ke, Z.-F. & Qiu, L.-Q. *ACS Catal.* **4**, 1390−1397 (2014).

8. Chen, Z.-G., Jiang, Q.-Z., Zhu, G.-X., Xiao, D.-M., Cao, P., Guo, C. & Zhang, X.-M. *J. Org. Chem.* **62**, 4521−4523 (1997).

9. Xue, F. & Hayashi, T. *Angew. Chem. Int. Ed.* **57**, 10368–10372 (2018).

10. Chi, Y., Zhang, W.-X. & Xi, Z.-F. *Org. Lett.* **16**, 6274–6277 (2014).
